# Enantioselective Iridium-Catalyzed Intramolecular Hydroarylation of (Hetero)Arene-Tethered Prochiral Diketones

**DOI:** 10.1021/jacs.6c05640

**Published:** 2026-05-07

**Authors:** Andrés Arribas, Carlos Lázaro-Milla, Martín Calvelo, José L. Mascareñas, Fernando López

**Affiliations:** † 16780 Centro Singular de Investigación en Química Biolóxica e Materiais Moleculares (CiQUS) and Departamento de Química Orgánica Universidade de Santiago de Compostela, 15782 Santiago de Compostela, Spain; ‡ Misión Biológica de Galicia (MBG), Consejo Superior de Investigaciones Científicas (CSIC), 36143 Pontevedra, Spain

## Abstract

The enantioselective iridium­(I)-catalyzed hydroarylation of C–C unsaturated bonds ranks among the most atom-efficient technologies in organic synthesis. We now report an enantioselective desymmetrizing cyclization that entails carbonyls instead of C=C partners, providing direct access to architecturally complex (hetero)­polycyclic frameworks bearing a tertiary alcohol and an adjacent all-carbon quaternary stereocenter at their ring junctions. The *in situ* dehydration of the initially formed alcohol was occasionally observed to yield products bearing an allylic quaternary stereocenter, which offers excellent opportunities for downstream functionalization. Mechanistically intriguing, we demonstrated that this dehydration can be suppressed by adding Et_3_SiH, an additive that also accelerates the overall transformation. Computational studies support a carbometalation/O–H reductive elimination pathway and shed light on the stereochemical origin of the enantioselectivity.

## Graphical abstract

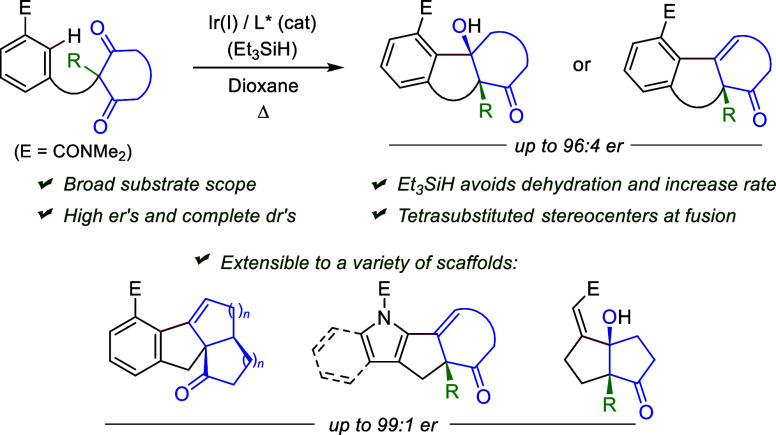

## Introduction

Over the past two decades, the transition-metal-catalyzed direct addition of inert C–H bonds across unsaturated moieties (i.e., hydrocarbonation reaction), has emerged as a powerful carbon–carbon bond-forming tool that enables a substantial increase of molecular complexity in an atom-economical manner.^1^ These reactions, which are usually triggered by an oxidative addition of the C–H bond to a low valent metal catalyst, have been thoroughly explored both in inter- and intramolecular contexts, with different metal catalysts.^2^ Moreover, a variety of enantioselective versions have also been successfully developed.^3^

Despite these significant advances, most enantioselective strategies remain limited to additions across C–C unsaturated partners (e.g., alkenes), whereas the use of carbonyl groups, which would open the door to the asymmetric synthesis of valuable chiral polycyclic alcohols, has been scarcely explored.^4,5^ A pioneering example disclosed by Shibata in 2009 illustrates one of the inherent challenges, as the initially formed chiral alcohols undergo *in situ* dehydration, ultimately delivering achiral alkene products (Scheme 1A, top).^6^ This elimination can be avoided by employing specifically designed α-keto carbonyl substrates that lack α-C–H bonds. However, this approach significantly narrows the scope of the method, which has so far remained restricted to the synthesis of chiral oxindoles (Scheme 1A, bottom).^6,7^

**Scheme 1:**
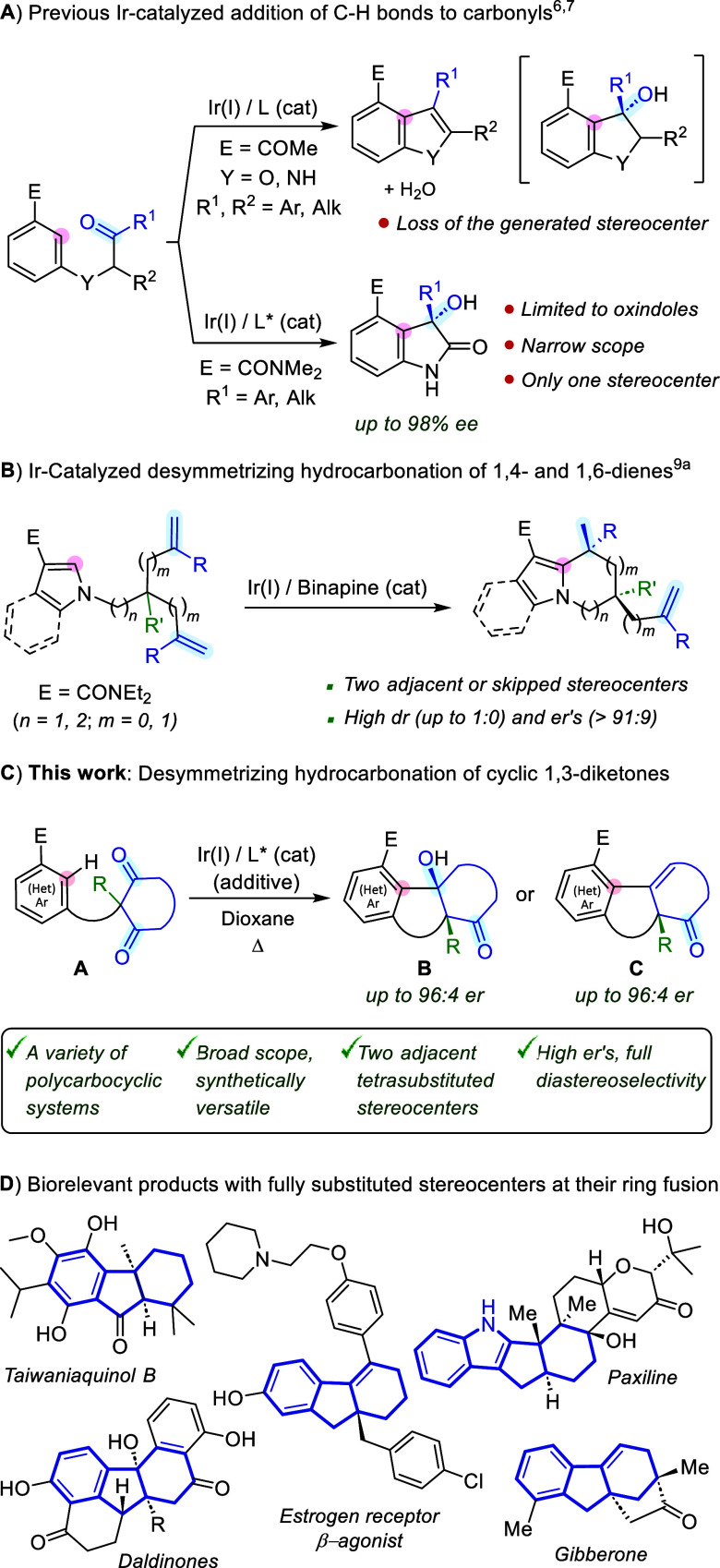
(A) Previous Ir-Catalyzed Intramolecular Hydrocarbonations of Carbonyls; (B) Desymmetrization of Prochiral 1,*n*-dienes; (C) Current Work; and (D) Biorelevant Products Bearing α-Chiral Tertiary Alcohols and/or Allylic Quaternary Stereocenters, at the Ring Fusion

Therefore, the development of general enantioselective cycloisomerization protocols involving the direct addition of inert C–H bonds across carbonyl groups to provide structurally complex polycarbocyclic chiral alcohols remains a highly appealing and yet unmet goal.^8^

Over the last few years, our group has developed a variety of iridium-catalyzed enantioselective hydrocarbonation reactions of C=C unsaturated systems,^9^ including an intramolecular desymmetrization of heteroaromatic systems bearing prochiral 1,*n*-dienes (Scheme 1B).^9a^ This reaction, promoted by an Ir­(I)/Binapine catalyst, delivers polyheterocyclic systems featuring either adjacent or skipped stereocenters, with high levels of diastereo- and enantioselectivity. Inspired by these developments, we reasoned that tethering cyclic 1,3-diketones to arene precursors through their C2 position would provide an excellent platform for performing iridium-catalyzed desymmetrizing carbonyl hydroarylations. Moreover, when these precursors contain a fully substituted prochiral C2-center, the cyclization would offer direct access to appealing polycyclic alcohols bearing a fused all-carbon quaternary stereocenter.

Herein, we report the successful implementation of this approach, a highly enantioselective desymmetrizing hydrocarbonation of aromatic systems tethered to prochiral cyclic 1,3-diones (**A**) (Scheme 1C).^10,11^ The process, promoted by an iridium­(I)/bisphosphine chiral catalyst, enables the construction of a broad range of polycyclic alcohols (products **B**) bearing tetrasubstituted stereocenters at their ring junctions. Depending on the substrate, an *in situ* dehydration to give alkene derivatives of type **C** may occur, but this pathway can be completely suppressed by the addition of Et_3_SiH, an additive that also accelerates the cyclization reaction. Both types of scaffolds (**B** and **C**) are highly relevant, as they constitute the core of many biorelevant compounds that are difficult to assemble in an enantioselective manner through alternative approaches (Scheme 1D).^12^ Finally, we also report experimental and computational mechanistic Density Functional Theory (DFT) studies that shed light on the elementary steps of the process and reveal key noncovalent interactions in the enantiodetermining transition states.

## Results and Discussion

At the outset, we selected prochiral benzamide **1a** as a model substrate to investigate the feasibility of the proposed hydrocarbonation process. Unfortunately, treatment of **1a** with the catalyst generated *in situ* from [Ir­(cod)_2_]­BAr^F^_4_ and of (*S*)-Binapine, which had provided excellent results in the Ir-catalyzed desymmetrizing hydrocarbonation of prochiral dienes,^9a^ led to the full recovery of starting material **1a** (Table 1, entry 1). However, using Binap as the ligand, we observed the formation of the tricyclic alkene **3a**, although in a poor 11% yield (entry 2). This product was assumed to result from the dehydration of putative tertiary alcohol intermediate **2a** (vide infra). Despite the low yield, the promising enantiomeric ratio of the product (86:14 er) led us to screen several other ligands, iridium­(I) sources, and reaction parameters, to improve both yield and selectivity (Tables 1 and S1–S3).^13^

**Table 1:**
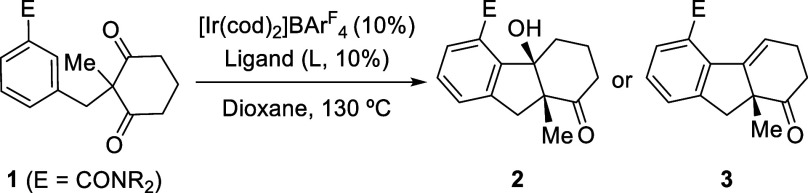
Preliminary Analysis of the Ir-Catalyzed Intramolecular Desymmetrization of Arene-Tethered Diketones **1** ^a^

entry	**1**, E	ligand (L)	**2**/**3**, yield (%)	er
1	**1a**, CONEt_2_	Binapine	**3a**, 0	–
2	**1a**, CONEt_2_	Binap	**3a**, 11	86:14
3	**1a**, CONEt_2_	Garphos	**3a**, 28	88:12
4	**1a**, CONEt_2_	MeO-Biphep	**3a**, 27	89:11
5	**1a**, CONEt_2_	Difluorphos	**3a**, 20	90:10
6	**1a**, CONEt_2_	C_3_-Tunephos	**3a**, 15	87:13
7	**1a**, CONEt_2_	SDP	**3a**, 31	58:42
8	**1a**, CONEt_2_	Segphos	**3a**, 32	91:9
9^b^	**1a**, CONEt_2_	Segphos	**3a**, 93	86:14
10	**1b**, CONMe_2_	Segphos	**3b**, 96	85:15
11	**1b**, CONMe_2_	DTBM-Segphos	**3b**, 94	90:10
12^c^	**1b**, CONMe_2_	DTBM-Segphos	**3b**, 84	90:10
13^d^	**1b**, CONMe_2_	DTBM-Segphos	**2b**, 63/**3b**, 32	90:10
14^c^ ^,^ ^e^	**1b**, CONMe_2_	DTBM-Segphos	**2b**, 76	92:8
15^e^ ^,^ ^f^	**1b**, CONMe_2_	DTBM-Segphos	**2b**, 81	91:9

a *Conditions*: **1** was added to a solution of [Ir­(cod)_2_]­BAr^F^_4_ (10%) and chiral ligand (L, 10%) in dioxane, and the mixture was heated at 130 °C for 16 h, unless otherwise noted; Yields (%) determined by NMR with an internal standard; er’s determined by HPLC.

b Reaction time: 96 h.

c Carried out with 5 mol % of [Ir­(cod)_2_]­BAr^F^_4_ / (*R*)-DTBM-Segphos.

d Carried out in the presence of 4 Å MS.

e Carried out in the presence of Et_3_SiH (1.25 equiv); Reaction time: 3 h.

f Carried out with 2.5 mol % of [Ir­(cod)_2_]­BAr^F^_4_/(*R*)-DTBM-Segphos; Reaction time 4 h.
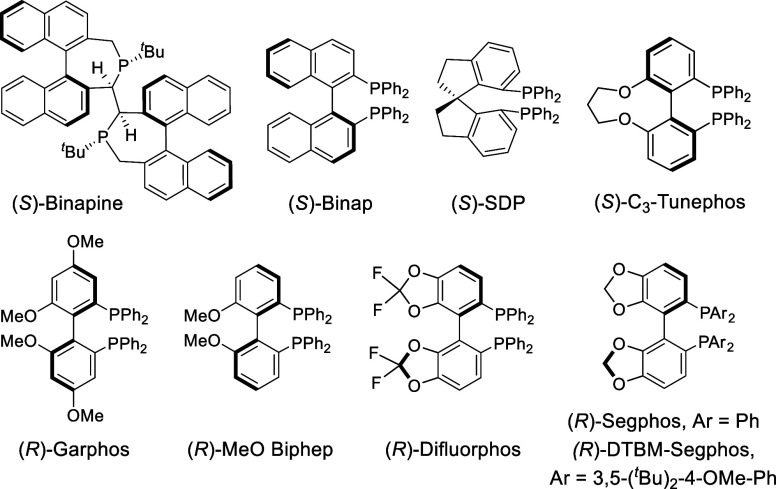

C2-symmetric bisphosphines like Binap, MeO-Biphep, Garphos, or Segphos, among others, did not significantly improve these values (entries 3–8 and Table S1), Segphos being the ligand that provided the best performance, a moderate 32% yield, and an excellent 91:9 er (entry 8). The yield could be increased by extending the reaction time up to 96 h (entry 9, 93% yield). Gratifyingly, after screening different directing groups (E), we found that the use of a dimethyl carboxamide (**1b**), instead of the diethyl derivative (**1a**), enabled the formation of the corresponding product (**3b**) in excellent yield while keeping the reaction time below 16 h (entry 10, 96% yield, 85:15 er). Moreover, by changing the ligand to the bulkier DTBM-Segphos, we could increase the er of **3b** up to 90:10 (94% yield, entry 11) and the catalyst loading could be reduced to 5 mol % with equally satisfying results (entry 12).

In some of the above reactions, we could detect non-dehydrated product **2b** in trace amounts (<3%). In this regard, we found that the proportion of alcohol **2b** can be increased by adding 4 Å MS to the reaction media (entry 13), suggesting that the presence of incidental water favors elimination to **3b**. More importantly, we were pleased to find that by using Et_3_SiH (1.25 equiv) as an additive, the reaction promoted by the Ir­(I)/DTBM-Segphos catalyst is significantly faster and exclusively delivers the expected alcohol, **2b**, which was isolated in 76% yield and 92:8 er (reaction time, 3 h, entry 14).^14^ Moreover, the catalyst loading could be further reduced down to 2.5 mol %, without significantly affecting the reaction rate, yield, or enantiomeric ratio of **2b** (entry 15). The *syn* stereochemistry of the product (**2b**), featuring a carbon quaternary stereocenter and the chiral tertiary alcohol at the ring fusion, was fully confirmed by X-ray analysis.^13^

At this point, we were in condition to explore the scope of the methodology, both in the absence and presence of Et_3_SiH (Scheme 2, A,B). As shown in Scheme 2A, precursors **1c**–**1g**, bearing linear or branched alkyl groups at the prochiral center, reacted smoothly with the Ir­(I)/DTBM-Segphos catalyst (5 mol %), without Et_3_SiH, to afford the corresponding dehydrated products of type **3** in good yields and with high enantiomeric ratios, ranging from 86:14 (**3e**) to 93:7 (**3g**). The er can be further improved by a simple recrystallization, as exemplified for **3f**, equipped with a benzyl group at the quaternary carbon center (97:3 er). Curiously, a precursor bearing a phenyl pendant at the prochiral center did not react under standard conditions; however, by replacing DTBM-Segphos with the less bulky Segphos ligand, the reactivity was restored, and product **3h** was obtained in 97% yield and with an 89:11 er. As evidenced by the reactions leading to products **3i**-**3l**, the method works very well with a variety of substituted prochiral cyclohexanediones, delivering the corresponding products in good yields and excellent enantiomeric ratios, which varied from 94:6 to 96:4. A precursor bearing a larger cycloheptane-1,3-dione also participated in the desymmetrization process, delivering the [6,5,7]-tricyclic product **3m** in 81% yield and with a 93:7 er. Likewise, the benzamide unit can also incorporate substituents at diverse positions without affecting the yield or the enantioselectivity (e.g., **3n**, **3o**).

**Scheme 2:**
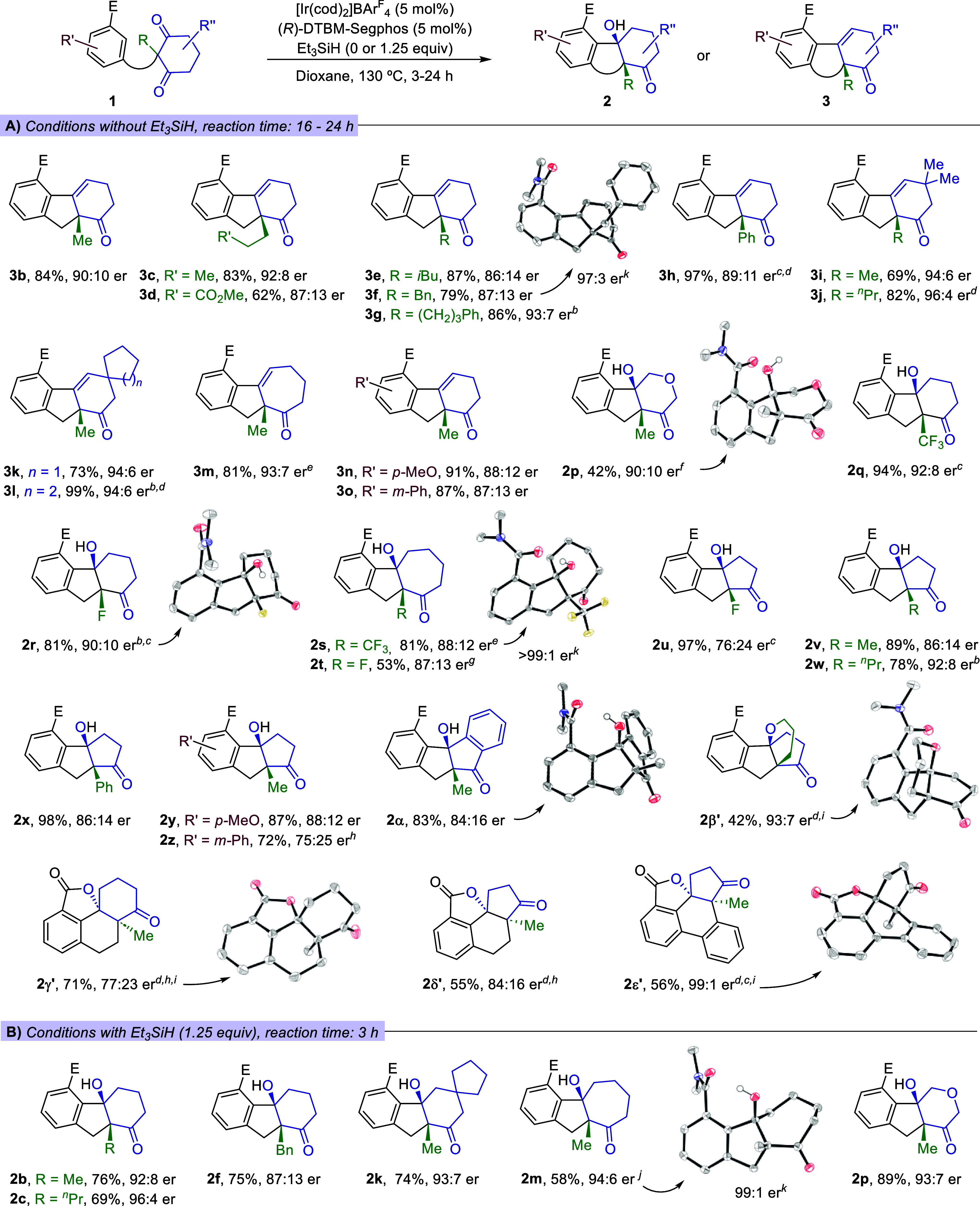
Scope of the Ir-Catalyzed Desymmetrizing Hydrocarbonations of Benzamide-Tethered 1,3-Diones. (A) Conditions without Et_3_SiH as additive. (B) Conditions using Et_3_SiH (1.25 equiv).[Fn s2fn1]–^k^

a
*Conditions*: A mixture of **1**, [Ir­(cod)_2_]­BAr^F^_4_ (5 mol %), and DTBM-Segphos (5 mol %) in dioxane, was heated at 130 °C for 16 h, unless otherwise noted; Isolated yields after chromatography; er’s determined by HPLC. E = CONMe_2_.
b Reaction time: 24 h.
c Carried out with Segphos instead of DTBM-Segphos.
d Carried out with 10 mol % catalyst.
e Carried out at 140 °C.
f 18% yield of **3p** (91:9 er) was also obtained.
g Carried out with Segphos at 110 °C for 16 h.
h Carried out with C_3_-Tunephos instead of DTBM-Segphos.
i Reaction time: 72 h.
j Reaction time: 6 h.
k Enantiomeric ratio obtained after recrystallization.

While in all the above examples, alcohols of type **2** were not observed, the hydrocarbonation of **1p**, which holds a prochiral pyran-dione unit, furnished chiral cyclopentanol **2p** in 42% yield (90:10 er), along with a lower amount of the dehydrated product **3p** (18% yield, 91:9 er). Importantly, in consonance with the result observed with **1b**, when carrying out this reaction in the presence of Et_3_SiH (1.25 equiv), chiral cyclic alcohol **2p** was obtained in 89% yield (after only 3 h), and with a 93:7 er. Likewise, in the presence of Et_3_SiH, other alcohols like **2c**, **2f**, **2k**, or **2m** were also obtained in good yields, complete *syn* selectivity and with equally high enantiomeric ratios (Scheme 2B).

Curiously, precursors that hold at the prochiral center electron-withdrawing fluorine or trifluoromethyl groups (**1q**-**1r**, R = F and CF_3_, respectively) led to the nondehydrated chiral products **2q** and **2r**, also in the absence of the additive (92:8 and 90:10 er, respectively). This effect of fluorine substituents was further confirmed with precursors bearing either a seven- or a five-membered prochiral 1,3-dione (**1s–1u**), which gave the corresponding alcohols (i.e., **2s–2u**), with complete *syn* selectivity, good yields an er’s up to 88:12. The er of **2s** was improved up to 97:3 by recrystallization and its absolute configuration could be confirmed by X-ray crystallographic analysis.^13^ Although the precise reasons why fluorine-containing substituents inhibit dehydration are not yet clear,^15^ these results are noteworthy from a synthetic perspective, as polycyclic scaffolds bearing fluorine-substituted stereocenters at their ring fusion are difficult to access through alternative enantioselective methods.^16^ Notably, we again observed in these cases a positive effect of Et_3_SiH in shortening the reaction times, which confirms that its beneficial impact on the reaction rate is general.^17^

Precursors bearing a five-membered diketone were, in general, less prone to dehydration. As a result, [6.5.5] ring-fused tricyclic alcohols such as **2v–2z** were obtained in excellent yields and high enantiomeric ratios, under the standard reaction conditions (without Et_3_SiH). On the other hand, a precursor bearing an indene-1,3-dione delivered the [6.5.5.6] ring-fused tetracyclic alcohol **2**α, in 83% yield and 85:15 er. A substrate equipped with a propanolyl mesylate substituent at the prochiral center of the diketone [**1**β, R = (CH_2_)_3_OMs] gave an alcohol (**2**β) that reacted *in situ* to provide the alkylated tetracyclic product **2**β**′** with a 92:8 er (54% yield).

The cyclization is also feasible in precursors that hold an additional methylene unit at the tether, which directly provide the lactones **2**γ**′** or **2**δ**′**, resulting from the *in situ* lactonization between the dimethyl amide and the newly formed alcohol. Moreover, substrates with phenyl rings as a constitutive part of the tether also gave the expected alcohols like **2**ε**′** in 56% yield and an excellent 99:1 er. Curiously, in these cases with a longer tether (**2**γ**′**, **2**δ**′**, **2**ε**′**), the absolute configuration of the major enantiomer is reversed, as confirmed by X-ray crystallography (**2**ε**′**).

We also explored the desymmetrization of alternative polycyclic prochiral diketones such as those shown in Scheme 3. Using the standard protocol with the Ir­(I)/DTBM-Segphos catalyst, we could assemble appealing tetracyclic alkenes like **5a**, in 91% yield and with an excellent 98:2 er (Scheme 3A, eq 1). Recrystallization of this product allowed one to increase further this value up to a 99.9:0.1 er, whereas a simple hydrogenation of **5a** led to the tetracyclic derivative **5a′** with complete stereoselectivity. The hydrocarbonation of an analogous bicyclo[3.3.0]­octane-based precursor (**4b**) provided the expected cyclization/elimination product **5b**, after 36 h at 130 °C (83% yield, 97:3 er, Scheme 3A, eq 2, right). Interestingly, when the reaction was carried out for just 4 h, alcohol **6b**, which bears three consecutive tetrasubstituted stereocenters, was obtained in 40% yield and with the same er (97:3), with a minor amount of **5b** (9% yield). As expected, performing this reaction in the presence of Et_3_SiH completely suppressed the dehydration, providing **6b** in 87% yield with 90:10 er. Note that product **6b** features an angular triquinane core, a structural motif that is present in a variety of natural sesquiterpenes with biologically relevant properties.^18^

**Scheme 3:**
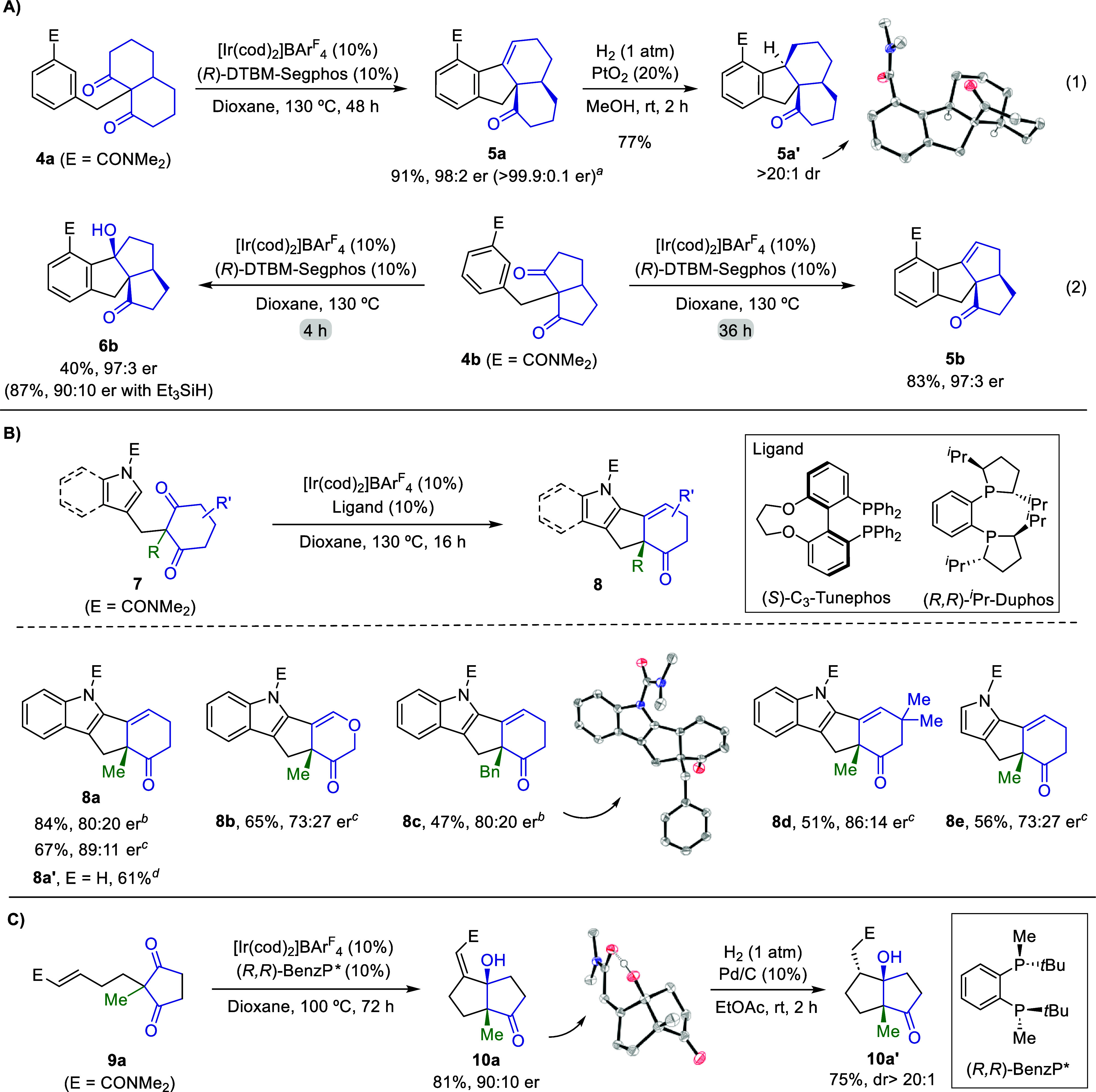
Application of the Enantioselective Desymmetrizing Process to Alternative Scaffolds: (A) Polycyclic Prochiral Diketones, (B) Indole-Tethered Diketones, and (C) Alkene-Tethered Prochiral Diketones^a^–[Fn s3fn4]

a Enantiomeric ratio obtained after recrystallization.
b Carried out with (*S*)-C_3_-Tunephos.
c Carried out with (*R*,*R*)-^*i*^Pr-Duphos.
d
*N*–H indole-product **8a′** was obtained from **8a** upon treatment with KOH (3.0 equiv), in EtOH (0.1 M) at 90 °C, 5 h.

Importantly, the desymmetrization process could be extended to azacyclic precursors of type **7**, enabling the assembly of exciting scaffolds that form the core of the paxiline and related indole diterpenes with intriguing biological properties (Scheme 3B).^19^ Although the reaction of the model precursor **7a** (R = Me) with the Ir­(I)/DTBM-Segphos gave modest results (**8a**, 52% yield, 60:40 er, Table S5),^13^ after a ligand screening we found that C_3_-Tunephos provides a more efficient process (84% yield, 80:20 er) and, particularly, ^*i*^Pr-Duphos further enhances the enantioselectivity up to 89:11 er (Scheme 3B). Notably, the dimethyl amide moiety used as a directing group at the indole nitrogen could be easily removed under basic conditions (Scheme 3B, footnote *d*). On the other hand, these Ir catalysts also promote the cyclization reaction of other indole-based precursors bearing different alkyl groups at the prochiral center, as well as of a pyrrole-based analogue precursor, enabling the access to a variety of heteropolycyclic scaffolds (**8a**–**8e**) featuring an endocyclic allylic quaternary center.

Next, we checked whether the method could also promote carbonyl hydrocarbonations through the activation of alkenyl, instead of aryl, C­(sp^2^)–H bonds, a transformation of high synthetic interest because of the relevance of the bicarbocyclic skeletons. Encouragingly, after a brief ligand screening, we found that the Ir­(I)/BenzP* complex promotes the cyclization of precursor **9a**, which bears an α,β-unsaturated carboxamide tethered to the prochiral 1,3-diketone. The corresponding bicyclic system **10a** is obtained in a good 81% yield, with an er of 90:10. Subsequent hydrogenation affords derivative **10a′**, bearing three consecutive stereocenters with almost complete stereoselectivity. This result constitutes the first example of an enantioselective hydroalkenylation of carbonyl compounds and further underscores the synthetic potential of this desymmetrizing method.

The dehydrated cyclization products have a rich functionality that can be exploited for diverse types of manipulations. The alkene, ketone, and dimethyl amide functional groups of model product **3b** could be selectively transformed through various reactions, providing a range of valuable derivatives with excellent chemo- and stereoselectivities (Scheme 4).

**Scheme 4:**
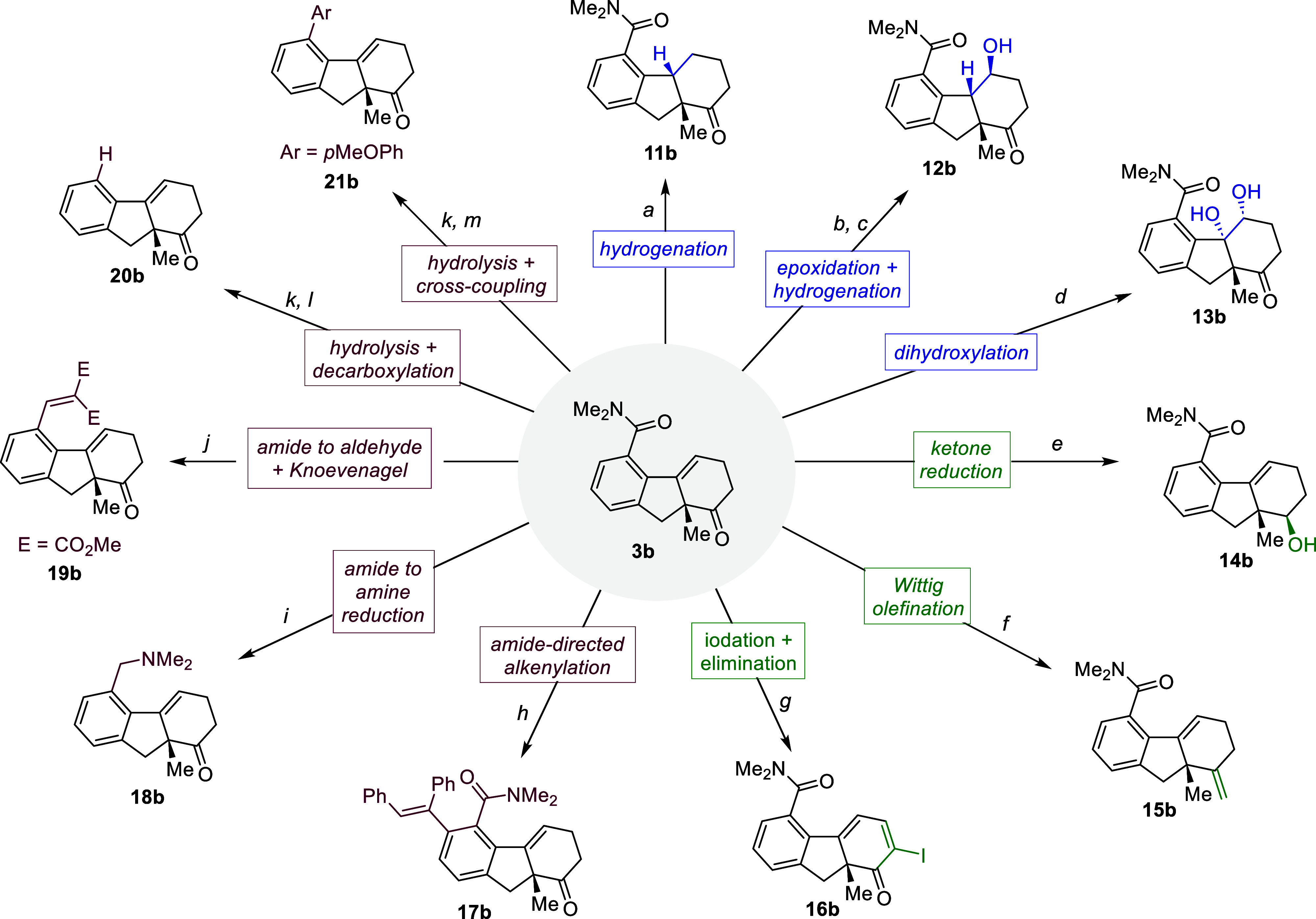
Preliminary Exploration of the Synthetic Potential of Products of Type **3** [Fn s4fn1] ^,^ ^b^

a
*Reagents and conditions:* (*a*) H_2_ (1 atm), Pd (C) (5 mol %), EtOAc, 65 °C (53% yield, dr = > 20:1). (*b*) *m*CPBA, CH_2_Cl_2_, 0 °C (42% yield). (*c*) H_2_, Pd­(C) (20%), MeOH, rt (39% yield, dr = > 20:1). (d) K_2_OsO_4_, NMO, MsNH_2_, acetone, H_2_O, rt (50% yield, dr = > 20:1). (*e*) Conditions: NaBH_4_, MeOH, 0 °C (65% yield, dr = > 20:1). (*f*) PPh_3_CH_3_,Br, tBuOK, THF, rt (57% yield). (*g*) I_2_, DBU, THF, rt (41% yield). (*h*) Diphenylacetylene, [Cp*RhCl_2_]_2_, AgSbF_6_, AcOH, DCE, 100 °C (MW) (71% yield). (*i*) Mo­(CO)_6_, TDMS, THF, 50 °C (62% yield) (*j*) Diethyl malonate, TMDS, Ir­(CO)­PPh_3_Cl, AcOH, toluene, rt (43% yield). (*k*) Me_3_OBF_4_, NaH_2_PO_4_, MeCN, then 1 M NaOH (84% yield). (*l*) Cu_2_O, 1,10-phenantroline, NMP, quinoline, 190 °C (MW) (69% yield). (*m*) *p*-MeOPhB­(OH)_2_, Pd­(OAc)_2_, dppb, Piv_2_O, Et_3_N, HBO_3_, dioxane, 160 °C (71% yield).
b Complete enantiospecificity was confirmed by HPLC.

As previously demonstrated for product **5a** (Scheme 3A, eq 1), catalytic hydrogenation was very effective, leading in this case to tricyclic product **11b** with complete stereoselectivity (conditions *a*, 53% yield). The absolute stereochemistry of this product could be confirmed by X-ray crystallographic analysis.^13^ On the other hand, epoxidation of **3b** with *m*CPBA, followed by a hydrogenative reductive ring opening afforded alcohol **12b**, as a single isomer. Likewise, dihydroxylation of **3b** furnished stereoselectively diol **13b** in 50% yield (conditions *d*). Reduction of the ketone moiety with NaBH_4_ was also stereoselective, providing **14b** in 65% yield, whereas its Wittig olefination delivered exocyclic alkene product **15b** in 57% yield. On the other hand, treatment of **3b** with I_2_ and DBU led to the α-iodo cyclohexadiene derivative **16b**, in 41% yield.^20^

Interestingly, the carboxamide group can be used to *ortho-*functionalize the aromatic ring of **3b**, as exemplified with a Rh-catalyzed alkyne hydroarylation that yields **17b** in 71% yield. The amide moiety can also be transformed selectively, without affecting the ketone moiety, either into its corresponding dimethyl amine derivative (**18b**) or into an aldehyde, which was trapped via a Knoevenagel condensation (**19b**, 45% yield). More importantly, the carboxamide can be readily converted into the corresponding acid (**3b′**, 84% yield), opening the door to a wide range of decarboxylative transformations.^21^ For instance, a Cu-catalyzed decarboxylative reaction affords unsubstituted aromatic product **20b** (69% yield), whereas a Pd-catalyzed decarboxylative Suzuki reaction affords **21b** (71% yield), which holds an aryl group at the former carboxamide position. Overall, these results confirm that the directing group provides a synthetic advantage rather than representing a burden, and further reinforces the synthetic relevance of the methodology.

To shed light on the mechanism of the cycloisomerization and the molecular origin of desymmetrization process, we performed DFT calculations using **1v** as a precursor and the iridium complex [(*R*-Segphos)­Ir]^+^ as a model catalyst.^22^ The coordination of the amide group to the catalyst enables the concomitant interaction of its *ortho* C–H bond with the iridium center (**I-1**, Figure 1). In consonance with previous calculations,^9,23^ a transition state with an accessible energy barrier of 18.5 kcal/mol (**TS-1**) delivers Ir­(III) hydride species **I-2**, which can coordinate either of the two carbonyl groups of the prochiral diketone moiety, leading to diastereomeric intermediates **I-3** ^***R***^ and **I-3** ^***S***^. Curiously, the energetic stabilization achieved by the carbonyl coordination (up to 1.5 kcal/mol) is lower compared to those values previously obtained for the related hydroarylations of C=C unsaturated partners (typically >5 kcal/mol)^9,23^ Intermediate **I-3** ^***R***^, which would eventually provide the experimentally observed (*R,R*) enantiomer **2v**, is 0.8 kcal/mol more stable than **I-3** ^***S***^.

**Figure 1:**
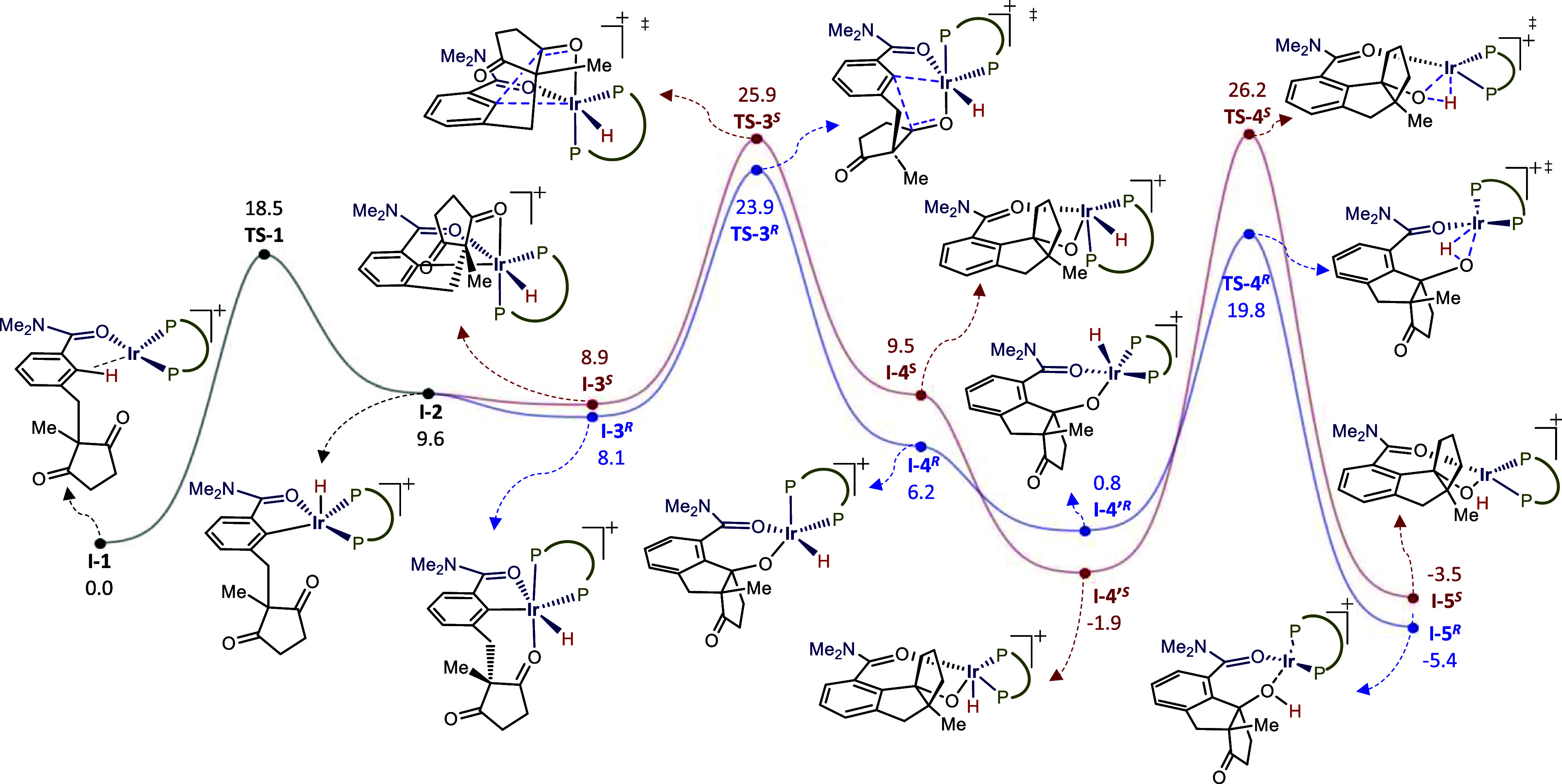
Energy profile ΔG^solv^ (kcal/mol) for the hydrocarbonation of **1v** ([Ir] = [Ir­(*R*-Segphos)]^+^); M06/6–311++g­(d,p) (SDD for Ir)//(B3LYP/6–31G­(d)) (LANL2DZ for Ir).

Importantly, the analysis of the carbonyl migratory insertions indicates that a carbometalation path is preferred over the alternative hydrometalation (Figure S16),^24^ with the activation barrier to **I-4** ^***R***^ (via **TS**-**3** ^***R***^) being lower (2.0 kcal/mol) than that leading to **I-4** ^***S***^. These findings are consistent with the experimentally observed major enantiomer and support the carbometalation as an enantiodetermining step. We also analyzed the subsequent O–Ir–H reductive elimination, as it is a scarcely explored elementary step in alkoxy iridium­(III) hydride complexes.^25^ As shown in Figure 1, intermediates **I-** **4ʼ** ^***R***^ and **I-4** **ʼ** ^***S***^ evolve to their more stable square pyramidal isomers (****I-4**ʼ** ^***R***^ and ****I-4**ʼ** ^***S***^), in which the hydride adopts an apical position, facilitating an effective orbital overlap between oxygen and hydrogen during the reductive elimination. The corresponding transition states differ markedly in energy, being the one leading to the (*R,R*)-**2v**, **TS-4** ^***R***^, 6.4 kcal/mol more stable than that delivering its (*S*,*S*)-counterpart (**TS-4** ^***S***^). According to the energetic span model,^26^ **TS-3** ^***R***^ is the TOF-determining transition state (TDTS) and **I-1** is the TOF-determining intermediate (TDI), resulting in an energetic span (δ*E*) of 23.9 kcal/mol, a value significantly higher than those obtained for related hydroarylations of C=C unsaturated partners (e.g., alkenes, alkynes or allenes, ΔΔG> 5.4 kcal/mol),^9^ in which the initial oxidative addition of the C–H bond to the Ir­(I) center was found to be the turnover-determining step of the overall process.^27^

To shed some light on the reasons behind the preference of the *R*-enantiomer, we performed an AIM analysis of transition states **TS-3** ^***S***^ and **TS-3** ^***R***^, to identify the noncovalent interactions (NCIs) involved in this enantiodetermining step, their bond paths, and bond critical points. This analysis indicates that preferred transition state **TS-3** ^***R***^ exhibits a significantly higher number of stabilizing NCIs. In particular, it features interactions between an aryl group of a diarylphosphine and the benzamide unit of **1b**, which are absent in the transition state leading to the minor enantiomer (Figure S18).^13^ This difference likely accounts for the relative stability of both transition states, as most of the remaining interactions are essentially equivalent, involving bonding contacts established by carbonyl groups, the iridium hydride, and the methyl group located at the prochiral center.

With regard to the dehydration step to form alkene products **3**, it may occur from intermediates **I-5** (Figure 1), due to the Lewis acid character of the Ir­(I) center. In consonance with this hypothesis, treatment of alcohol **2b** with the preformed precatalyst [(DTBM-Segphos)­Ir­(cod)]­BAr^F^_4_ (**Ir1**, 5 mol %, CCDC 2513813), in dioxane at 130 °C, quantitatively afforded alkene **3b** (Scheme 5A and Table S6). Likewise, the formation of **3b** was also observed when **2b** was heated in the presence of catalytic amounts of [Ir­(cod)_2_]­BAr^F^_4_ and different Lewis acids such as IrCl_3_ or ZnCl_2_.^28^ Curiously, when alcohol **2b** was treated with the iridium catalyst **Ir1** (10 mol %) in the presence of Et_3_SiH (1.25 equiv), the dehydrated product **3b** was predominantly formed after 3 h (ratio **2b**:**3b** = 1:3). This observation suggests that the absence of dehydration product **3b** in the standard cycloisomerization reaction with Et_3_SiH as an additive mainly results from a kinetic acceleration of the carbonyl hydroarylation, rather than from the inhibition of the alcohol elimination step.

**Scheme 5:**
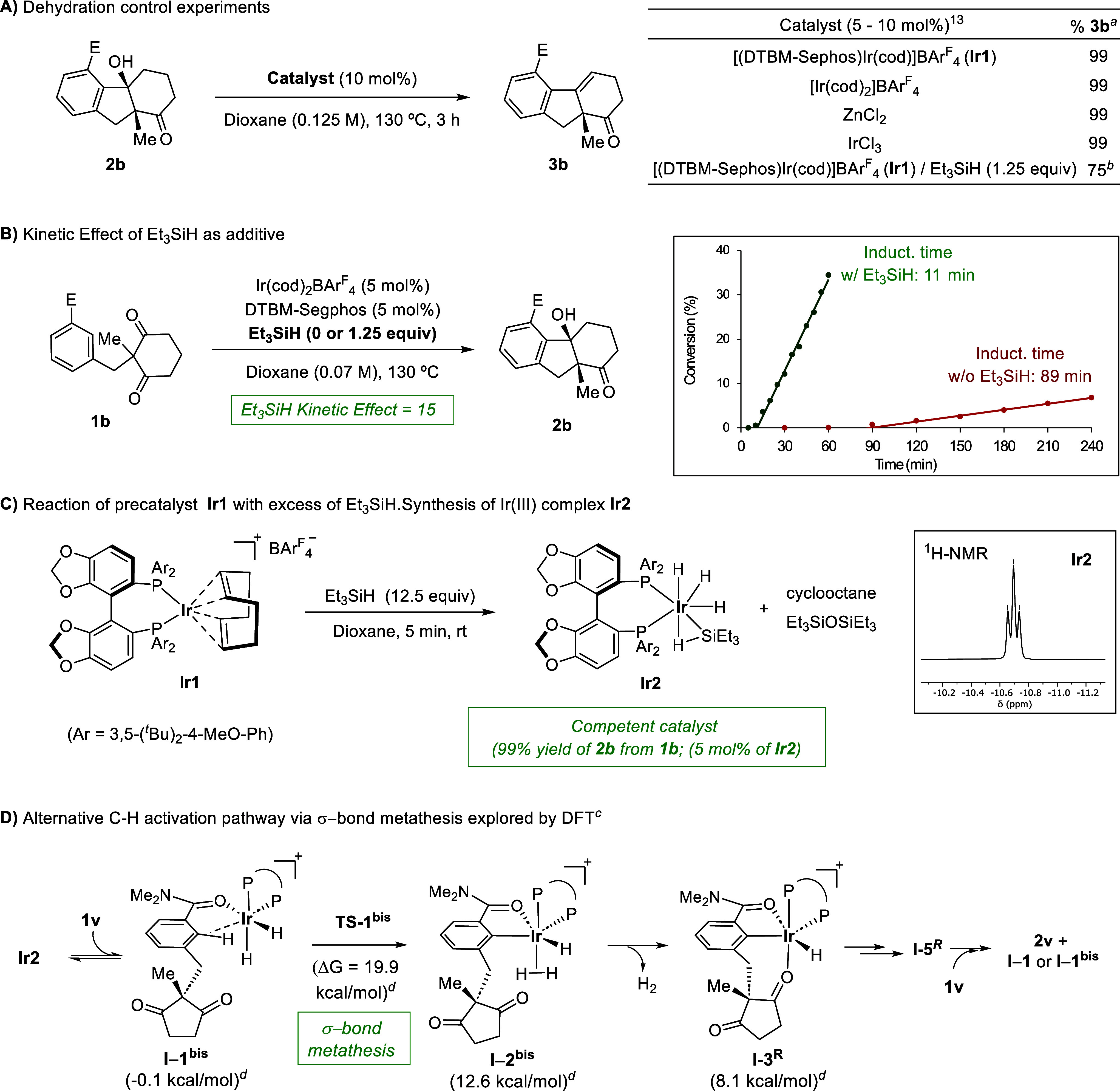
Mechanistic Studies. (A) Control Experiments on the Elimination of **2b**. (B) The Role of Et_3_SiH and Kinetic Data. (C) Stoichiometric Reaction between **Ir1** and Et_3_SiH, Synthesis of **Ir2**, off-Cycle Resting Species. (D) DFT Calculations with (*R*)-Segphos and **1v**. M06/6–311++g­(d,p) (SDD for Ir)//(B3LYP/6–31G­(d) (LANL2DZ for Ir)^a^–^d^

a Calculated by NMR with internal standard.
b 25% of **2b** was also observed (**2b**:**3b** = 1:3).
c DFT calculations carried out using (*R*)-Segphos and substrate **1v** as a model system, to allow direct comparisons with the energy profile of Figure 1.
d Energy reference: **I-1** (Figure 1).

Analysis of product distribution at low conversion in the presence and absence of Et_3_SiH further supports this interpretation. In the absence of Et_3_SiH, after a long induction period of ca. 90 min, the reaction proceeds slowly affording exclusively alcohol **2b**, at least during the first 3 h (conversion <10%, Scheme 5B). In contrast, with Et_3_SiH, the reaction begins after only ∼11 min and proceeds about 15 times faster at early stages, also exclusively producing **2b**, reaching 35% conversion in 1 h and full conversion within 3 h. The induction period observed without Et_3_SiH reflects the higher coordination affinity of COD relative to the substrate (**1b**) at the Ir­(I) center, which delays the formation of a substrate-bound intermediate of type **I-1** that initiates the catalytic cycle.^29^ Accordingly, control experiments showed that adding external COD to the reaction promoted by **Ir1** has a detrimental effect on the reaction rate (see Table S5).

To gain more information on the origin of the acceleration in the presence of Et_3_SiH, we monitored by NMR the reaction between freshly prepared precatalyst [(DTBM-Segphos)­Ir­(cod)]­BARF (**Ir1**) and an excess of Et_3_SiH. Interestingly, we observed a rapid conversion into a new Ir­(III) hydride species (**Ir2**), which displays a characteristic ^1^H NMR signal at −10.8 ppm (Scheme 5C).^13^ Consistent with precedents involving related achiral complexes,^30^ **Ir2** might arise from the reaction of **Ir1** with H_2_ generated via an Ir-promoted dehydrosilylation of adventitious water with Et_3_SiH.^31^ Indeed, both (Et_3_Si)_2_O and cyclooctane were detected by NMR in the reaction mixture. Importantly, heating a dioxane solution of **1b** with iridium hydride complex **Ir2** (10 mol %) and Et_3_SiH (1.25 equiv) at 130 °C for 3 h quantitatively afforded alcohol **2b** with the same er (92:8), confirming the catalytic competence of **Ir2** (Scheme 5C).^13^ Therefore, the coordinatively saturated Ir­(III) complex **Ir2** is likely an off-cycle species,^32^ in equilibrium with the catalytically relevant substrate-bound Ir­(I) species [(DTBM-Segphos) (**1b**)­Ir]^+^, which constitutes the entry point to the catalytic cycle (**I-1**, Figure 1).

Additionally, complexes of type **Ir2** could also be in equilibrium with Ir­(III) dihydride species such as **I-1** ^**bis**^ (Scheme 5D), which may also enter the catalytic cycle. Indeed, DFT calculations carried out using **1v** as a substrate and Segphos as a ligand indicate that **I-1** ^**bis**^, which was found to be almost isoenergetic with **I-1** (ΔG = −0.2 kcal/mol, see Figure S17 and Scheme 5D), can undergo a C–H activation through a σ-bond-metathesis-like pathway, with an energetically accessible barrier of ca. 20 kcal/mol, very similar to that calculated from **I-1** (ΔΔG = 1.4 kcal/mol, Figure S17). The resulting Ir­(III) species **I-2** ^**bis**^ would then evolve to the previously identified intermediate **I-3** ^***R***^, upon H_2_ decoordination (Scheme 5D). This alternative C–H activation path is analogous to that proposed for Ir-catalyzed hydrogen isotope exchange^33^ and is consistent with the essentially identical enantioselectivities observed with and without Et_3_SiH, since the enantiodetermining step (**I-3** → **I-4**) is common to both pathways. After the completion of the catalytic cycle, species **I–5** (Figure 1) could exchange product **2b** for substrate **1b** to restart the Ir­(I)/Ir­(III) catalytic cycle via **I-1**, or be oxidized to eventually regenerate the Ir­(III) dihydride species **I-1** ^**bis**^.

Taken together, these data indicate that Et_3_SiH plays several beneficial roles. On one hand, it accelerates the hydroarylation by removing COD from the reaction media (via hydrogenation to cyclooctane) and by enabling an alternative σ-bond metathesis C–H activation route, which likely increases the concentration of **I-3**.^34^ On the other hand, it eventually suppresses dehydration of **2b** by removing adventitious water through an Ir-catalyzed dehydrosilylation process. This interpretation is also consistent with the positive effect observed when 4 Å molecular sieves are used under the standard reaction conditions (Table 1, entry 13).

## Conclusion

In summary, we have developed the first enantioselective desymmetrizing hydroarylation of prochiral cyclic 1,3-diketones. The transformation, promoted by a chiral Ir­(I)-bisphosphine catalyst, delivers a variety of relevant polycyclic structures bearing two adjacent tetrasubstituted stereocenters at the ring junctions. The dehydration of the initially formed alcohol, which is occasionally observed, can be completely suppressed by the addition of Et_3_SiH. Notably, this additive significantly accelerates the overall transformation through the involvement of a chiral iridium­(III) hydride species that acts as an off-cycle intermediate. The method accommodates a variety of aromatic, heteroaromatic, and alkenyl C–H donors as well as diverse prochiral diketone precursors, providing a variety of scaffolds that exhibit a high synthetic value. DFT computational studies provided insights into the reaction mechanism and unveiled key noncovalent interactions responsible for the high levels of enantioselectivity observed.

## Supplementary Material



## Notes

Full experimental procedures, characterization data of all new compounds (including ^1^H-, ^13^C NMR spectra), computational details, and Cartesian coordinates (PDF).
