# Supplementary material for: Enantioselective Iridium-Catalyzed Intramolecular Hydroarylation of (Hetero)Arene-Tethered Prochiral Diketones
Source: J Am Chem Soc. 2026 May 7;148(19):20203–14. doi: 10.1021/jacs.6c05640 (PMC13195674; doi:10.1021/jacs.6c05640)
Supplement: Supplementary file 1 [file ja6c05640_si_001.pdf]

Supporting Information

Enantioselective Iridium-Catalyzed Intramolecular Hydroarylation of (Hetero)Arene-Tethered Prochiral Diketones

Andrés Arribas,[†,‡] Carlos Lázaro-Milla,[†,‡] Martín Calvelo,[†] José L. Mascareñas,[†] Fernando López*[†,§]

[†] Centro Singular de Investigación en Química Biolóxica e Materiais Moleculares (CIQUS) and Departamento de Química Orgánica Universidade de Santiago de Compostela, 15782, Santiago de Compostela (Spain).

[§] Misión Biológica de Galicia (MBG), Consejo Superior de Investigaciones Científicas (CSIC) 36143, Pontevedra (Spain).

‡ These authors contributed equally

Corresponding Author:

Dr. Fernando López: fernando.lopez@csic.es

Table of contents

1. General procedures 3

2. Optimization of the Ir-catalyzed intramolecular desymmetrizing hydrocarbonation of prochiral 1,3-diketones ... 4

3. Procedures for the synthesis of substrates 12

General Procedure A: Synthesis of 2-alkyl-1,3-diketone substrates (illustrated for **1b**) 12

General Procedure B: Synthesis of 2-aryl-1,3-diketone substrates (illustrated for **1h**) 19

General Procedure C: Preparation of 2-trifluoromethylated-1,3-diketone substrates (illustrated for **1q**) 21

General Procedure D: Preparation of 2-fluorinated-1,3-diketone substrates (illustrated for **1r**) 22

General Procedure E: Preparation of mesylated precursor 1β 23

General Procedure F: Synthesis of homologated-1,3-diketone 1γ 25

General Procedure G: Synthesis of homologated-1,3-diketone 1δ 26

General Procedure H: Synthesis of homologated-1,3-diketone 1ε 28

General Procedure I: Synthesis of fused-bicyclic-1,3-diketone substrates (illustrated for **4a**) 29

General Procedure J: Synthesis of indole-1,3-diketone substrates (illustrated for **7a**) 30

General Procedure K: Synthesis of alkenyl-1,3-diketone substrate 9a 33

4. Ir-catalyzed intramolecular hydrocarbonation (**General Procedure L**, illustrated for **3b**) 34

5. Dehydration control experiments 82

6. Transformations of the products obtained 84

7. Mechanistic studies with Et_3SiH 97

8. Kinetic studies 106

9. Single crystal X-ray diffractometry details 110

10. Computational studies 126

11. NMR spectra 163

12. References 279

1. General procedures

All reactions were conducted in dry solvents under Ar atmosphere unless otherwise stated. The abbreviation “rt” refers to reactions carried out at 20-25 °C. Reaction mixtures were stirred using Teflon-coated magnetic stir bars. Reaction temperatures were maintained using Thermowatch-controlled silicone oil baths. Dry solvents were obtained from a solvent purification system (Mbraun, SPS-5) or freshly distilled under Ar from an appropriate drying agent before use (toluene from Na / benzophenone, CH_2Cl_2 distilled form CaH_2). The water utilized in the reactions is sourced from a Milli-Q® water purification system. All other reagents for the synthesis of precursors were purchased from Aldrich. Thin-layer chromatography (TLC) was performed on pre-coated silica gel F_{254} plates and were visualized by observation under UV light, or by treating the plates with either vanillin, *p*-anisaldehyde, ninhydrin, potassium permanganate or cerium nitrate solutions, followed by heating. Dryings were performed with anhydrous Na_2SO_4. Concentration refers to the removal of volatile solvents via distillation using a Buchi rotary evaporator, followed by residual solvent removal under high vacuum. In reactions carried out in sealed tubes, the mixture must be at rt before sealing, to avoid overpressure at high temperatures. Flash chromatography was carried out on silica gel (40-60 µm) unless otherwise stated. Flash MPLC chromatography was performed in a CombiFlash NextGen 300+ using 4g or 12g normal phase SiO_2 RediSep® Flash Columns (Gold and Bronze). Crude residues were transferred to the columns through RediSep® solid load cartridges (5g or 25g). Flow rates used from 13 to 30 mL/min.

^{1}H, ^{13}C and ^{19}F spectra were recorded in $CDCl_3$, CD_2Cl_2, or C_6D_6 at Varian Mercury 300 MHz or Bruker AVIII 500 MHz spectrometers. Carbon types were determined from DEPT-NMR experiments. NMR spectra were analyzed using MestreNova© processing software (http://www.mestrelab.com). 1,3,5-Trimethoxybenzene was used as internal standard for analyzing the reaction crudes by NMR. ^{1}H-NMR spectral data are reported as follows: chemical shift (δ ppm), integration, multiplicity (s = singlet, d = doublet, t = triplet, q = quartet, dd = double doublet, td = triple doublet, m = multiplet, br = broad). Data for ^{13}C are reported in terms of chemical shift relative to the residual solvent peak.

Mass spectra were acquired using chemical ionization (CI) or electron impact techniques (EI) and were recorded using IT-MS Bruker AmaZon SL at the CACTUS facility of the USC.

X-ray crystallographic analysis[1] was performed at the CACTUS facility of the USC in a Rigaku XtaLAB Synergy, Dualflex, HyPix-Arc 150 using a microfocus sealed tube as diffraction source. Determination of melting points was achieved with Buchi M-560 melting point system and are uncorrected.

Enantiomeric ratios (er) were determined on an Agilent HPLC 1100 Series or on a Jasco SFC 4000 series by using commercially available chiral columns.

Bis(cyclooctadiene)iridium(I) tetrakis(3,5-bis(trifluoromethyl) phenyl) borate (abbreviated as $[Ir(COD)_2]BAr^F_4$, CAS: 666826-16-0) was used freshly prepared.[2] Bis(1,5-cyclooctadiene) Iridium(I) tetrafluoroborate (abbreviated as $[Ir(COD)_2]BF_4$, CAS: 35138-23-9) and Bis(1,5-cyclooctadiene)Iridium(I) trifluoromethanesulfonate (abbreviated as $[Ir(COD)_2]OTf$, CAS: 413621-65-5) were purchased from Aldrich and BLD. Other bisphosphines were purchased from Aldrich, Strem-Chemicals or BLD. All other reagents used were bought from Aldrich, BLD or TCI and used without further purification.

2. Optimization of the Ir-catalyzed intramolecular desymmetrizing hydrocarbonation of prochiral 1,3-diketones

Table S1. Evaluation of different ligands, temperatures and reaction times in **1a** as model substrate.[a]

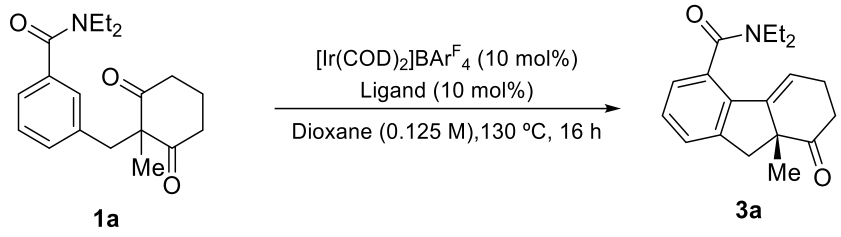

Entry	Ligand	Yield **3a** (%)	er
1	d^{F}ppe	0	-
2	(*S*)-Binap	11	86:14
3	(*S*)-Binapine	0	-
4	(*R,R*)-QuinoxP*	0	-
5	(*S*)-Phanephos	0	-
6	(*S*)-SDP	31	58:42
7	(*R*)-Monophos	0	-
8	(*S,S*)-Chiraphos	17	67:13
9	(*R*)-BTFM-Garphos	34	77:23
10	(*R*)-Xyl-Garphos	15	85:15
11	(*R*)-Garphos	28	88:12
12	(*R*)-DTBM-Garphos	0	-
13	(*R*)-MeO-Biphep	27	89:11
14	(*R*)-Fur-Biphep	18	83:17
15	(*S*)-C_3-Tunephos	15	87:13
16	(*S*)-Synphos	42	88:12
17	(*R*)-Segphos	32	90:10
18[b]	(*R*)-Segphos	93	86:14
19[b,c]	(*R*)-Segphos	36	91:9
20	(*R*)-Difluorphos	20	91:9
21	(*R*)-DTBM-Segphos	0	-

[a] Conditions: A solution of **1a** (0.1 mmol), $[Ir(COD)_2]BAr^F{}_4$ (10 mol%), Ligand (10 mol%) and dioxane (0.125 M) was heated at the indicated temperature and time following the General Procedure L. [b] Reaction time: 96 h. [c] Reaction temperature: 100 ˚C

Table S2. Evaluation of different directing groups and ligands.[a]

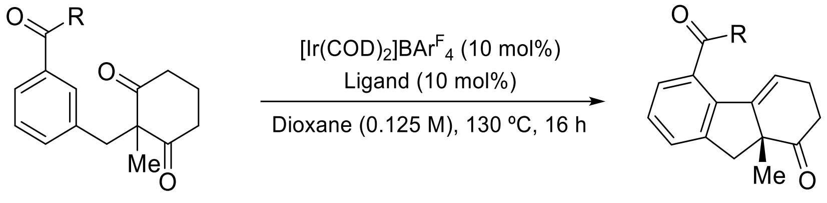

Entry	R	Ligand	Yield **3** (%)	er
1	NEt_2 (**1a**)	*rac*-Binap	11 (**3a**)	-
2	NEt_2 (**1a**)	(*R*)-Segphos	32 (**3a**)	90:10
3	NEt_2 (**1a**)	(*R*)-DTBM-Segphos	0 (**3a**)	-
4	pyrrolidinyl (**1a^{I}**)	*rac*-Binap	78 (**3a^{I}**)	-
5	pyrrolidinyl (**1a^{I}**)	(*R*)-Segphos	99 (**3a^{I}**)	86:14
6	pyrrolidinyl (**1a^{I}**)	(*R*)-DTBM-Segphos	99 (**3a^{I}**)	88:12
7	N(OMe)Me (**1a^{II}**)	*rac*-Binap	Traces (**3a^{II}**)	-
8	N(OMe)Me (**1a^{II}**)	(*R*)-DTBM-Segphos	0 (**3a^{II}**)	-
9	OMe (**1a^{III}**)	*rac*-Binap	16 (**3a^{III}**)	-
10	OMe (**1a^{III}**)	(*R*)-DTBM-Segphos	31 (**3a^{III}**)	74:26
11	Me (**1a^{IV}**)	*rac*-Binap	40 (**3a^{IV}**)	-
12	Me (**1a^{IV}**)	(*R*)-DTBM-Segphos	51 (**3a^{IV}**)	65:35
13	NMe_2 (**1b**)	*rac*-Binap	88 (**3b**)	-
14	NMe_2 (**1b**)	(*R*)-Segphos	96 (**3b**)	85:15
**15**	**NMe_2 (1b)**	**(*R*)-DTBM-Segphos**	**94 (3b)**	**90:10**

[a] Conditions: A solution of **1a** (0.1 mmol), $[Ir(COD)_2]BAr^F_4$ (10 mol%), Ligand (10 mol%) and dioxane (0.125 M) was heated at 130 ˚C, 16 h following General Procedure L.

Table S3. Evaluation of different ligands, catalyst counterions, additives, solvents, employing **1b** as substrate.[a]

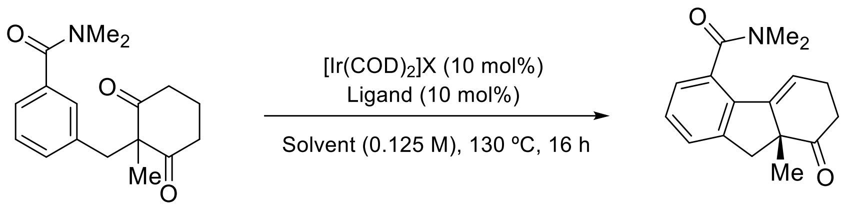

Entry	Ligand	X	Solvent	Yield **3b** (%)	er
1	*rac*-Binap	BAr^F_4	Dioxane	88	-
2	(*R*)-Segphos	BAr^F_4	Dioxane	96	85:15
3	(*R*)-DTBM-Garphos	BAr^F_4	Dioxane	34	84:16
4	(*R*)-DTBM-Binap	BAr^F_4	Dioxane	75	85:15
5	(*S*)-DTBM-Synphos	BAr^F_4	Dioxane	27	85:15
6	(*R*)-DTMS-Segphos	BAr^F_4	Dioxane	98	86:14
7	(*R*)-DTMS-Garphos	BAr^F_4	Dioxane	88	85:15
8	(*R*)-Sunphos	BAr^F_4	Dioxane	91	71:29
**9**	(*R*)-DTBM-Segphos	BAr^F_4	Dioxane	94	90:10
10	(*R*)-DTBM-Segphos	BF_4	Dioxane	<5	-
11	(*R*)-DTBM-Segphos	OTf	Dioxane	<5	-
12	(*R*)-DTBM-Segphos	BAr^F_4	Toluene	<5	-
13	(*R*)-DTBM-Segphos	BAr^F_4	Fluorobenzene	<5	-
14	(*R*)-DTBM-Segphos	BAr^F_4	DCE	<5	-
15	(*R*)-DTBM-Segphos	BAr^F_4	DME	<5	-
16	(*R*)-DTBM-Segphos	BAr^F_4	2-MeTHF	<5	-

[a] Conditions: A solution of **1b** (0.1 mmol), $[Ir(COD)_2]X$ (10 mol%), Ligand (10 mol%), Solvent (0.125 M) and Additive (1.5 equiv) was heated at 130 ˚C, 16 h following the General Procedure L.

Table S4. Evaluation of additives employing **1b** as model substrate and Ir/DTBM-Segphos as catalyst.[a]

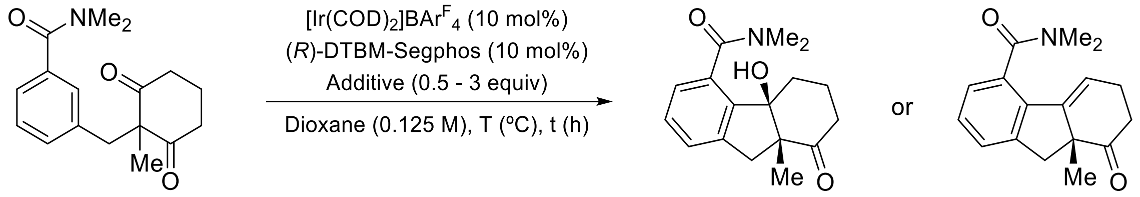

1b **2b** **3b**

Entry	Additive	Equiv	T (°C)	t (h)	Yield **2b** (%)	er **2b**	Yield **3b** (%)	er **3b**
1	TMSCl	1.5	130	16	0	-	0	-
2	AcCl	1.5	130	16	0	-	0	-
3	H_2O	1.5	130	16	0	-	0	-
4	Mol. Sieves 4Å	0.2 g/mmol	130	3	<5	-	<5	-
5	Mol. Sieves 4Å	0.2 g/mmol	130	16	63	n.d.	32	n.d.
6	Et_3SiH	1.25	130	16	30	91:9	70	91:9
7	Et_3SiH	1.25	130	3	78	92:8	0	-
8	Et_3SiH	0.5	130	5	28	92:8	42	92:8
9[c]	Et_3SiH	1.25	130	3	76	92:8	0	-
10[d]	Et_3SiH	1.25	130	3	63	91:9	0	-
11	Et_3SiH	3	100	18	0[e]	-	0	-
12	Et_3SiH	1.25	115	5	0[f]	-	0	-
13	$BnMe_2SiH$	1.25	130	3	72	92:8	0	-
14	$^{i}Pr_3SiH$	1.25	130	3	63	n.d.	36	n.d.
15	$^{t}BuMe_2SiH$	1.25	130	3	0	n.d.	<10	n.d.
16[f]	B_2Pin_2	1.25	130	3	0	-	0	-
17	HBPin	1.25	130	3	45	92:8	47	90:10

[a] Conditions: A solution of **1b** (0.1 mmol), [Ir(COD)$_2$]BAr$^F{}_4$ (10 mol%), Ligand (10 mol%), Dioxane (0.125 M) and Additive (0.5-3 equiv) was heated at the indicated temperature and time following the General Procedure L. [b] **1b** was recovered. [c] Carried out with 5 mol% of catalyst. [d] Carried out with 2.5 mol% of catalyst. [e] Complex mixture. [f] **1b** was recovered together with a complex mixture.

Table S5. Evaluation of different Iridium (I) precatalysts, employing **1b** as substrate and a reaction time of 3h. Effect of adding external COD.[a]

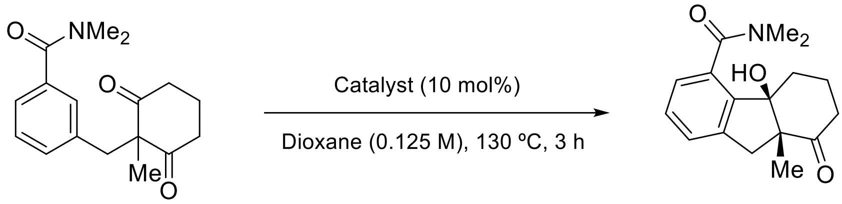

1b **2b**

Entry	Catalyst	Yield **2b** (%)
1	Ir(**COD**)$_2$BAr$^F{}_4$ (10%) + (*R*)-DTBM-Segphos (10%)	7[b]
2	Ir(**COD**)$_2$BAr$^F{}_4$ (10%) + (*R*)-DTBM-Segphos (10%) + Et_3SiH (1.25 equiv)	78
3	[Ir(**COD**)Cl]$_2$ (5%) + (*R*)-DTBM-Segphos (10%) + NaBAr$^F{}_4$ (10%)	14[b]
4	Ir(**COD**)$_2$BAr$^F{}_4$ (10%) + (*R*)-DTBM-Segphos (10%) + **COD** (20%)	0[b]
5	[Ir(**COD**)Cl]$_2$ (5%) + (*R*)-DTBM-Segphos (10%) + NaBAr$^F{}_4$ (10%) + **COD** (20%)	0[b]

[a] Conditions: A solution of **1b** (0.1 mmol), Iridium catalyst (5 or 10 mol%), Solvent (0.125 M) and was heated at 130 ˚C, 3 h following the General Procedure L. [b] Determined by GS-MS of the crude reaction mixture.

Table S6. Optimization of the hydroarylation of precursors of type **7**.[a]

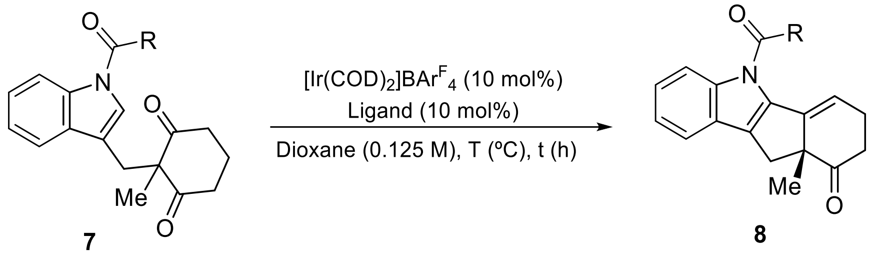

7 **8**

Entry	R	Ligand	T (°C)	t (h)	Yield **8** (%)	er
1	-NMe_2 (**7a**)	(*R*)-DTBM-Segphos	130	48	52	58:42
2	-NMe_2 (**7a**)	(*S*)-Difluorphos	130	16	38	63:37
3	-NMe_2 (**7a**)	(*S*)-H8-Binap	130	16	60	65:35
4	-NMe_2 (**7a**)	(*R*)-Ph-Garphos	130	16	49	74:26
5	-NMe_2 (**7a**)	(*S*)-BTFM-Garphos	130	16	39	60:40
6	-NMe_2 (**7a**)	(*R*)-MeO-Biphep	130	16	68	74:26
7	-NMe_2 (**7a**)	(*S*)-C_1-Tunephos	130	16	92	72:28
8	-NMe_2 (**7a**)	(*S*)-C_2-Tunephos	130	16	92	73:27
9	-NMe_2 (**7a**)	(*S*)-C_3-Tunephos	130	16	84	80:20
10	-NMe_2 (**7a**)	(*S*)-C_5-Tunephos	130	16	78	75:25
11	-NMe_2 (**7a**)	(*R,R*)-QuinoxP*	130	16	59	68:32
12	-NMe_2 (**7a**)	(*R,R*)-BenzP	130	16	84	86:14
13	-NMe_2 (**7a**)	(*R,R*)-BenzP	115	16	31	93:7
14	-NMe_2 (**7a**)	(*R,R*)-BenzP	100	16	0	-
15[b]	-NMe_2 (**7a**)	(*R,R*)-BenzP	130	16	0	-
**16**	-NMe_2 (**7a**)	**(*R,R*)-*i*Pr-Duphos**	**130**	**16**	**67**	**89:11**
17	-NMe_2 (**7a**)	(*R,R*)-Et-Duphos	130	16	71	62:38
18	-NMe_2 (**7a**)	(*R,R*)-Binaphene	130	16	0	-
19	-NMe_2 (**7a**)	Josiphos SL-J002-1	130	16	16	73:27
20	-NMe_2 (**7a**)	Josiphos SL-J502-2	130	16	36	75:25
21	-NMe_2 (**7a**)	(*S,S*)-f-Binaphane	130	16	22	66:34
22	-NMe_2 (**7a**)	(*S,S*)-Chiraphos	130	16	0	-
23	-NMe_2 (**7a**)	(*S*)-Xyl-SDP	130	16	0	-
24	-NMe_2 (**7a**)	(*S*)-Binapine	130	16	0	-
25	Piperidinyl (**7a**^{I})	*rac*-Binap	130	16	98 (**8a**^{I})	-
26	Piperidinyl (**7a**^{I})	(*R,R*)-BenzP	130	16	80 (**8a**^{I})	66:34

[a] Conditions: A solution of **7** (0.1 mmol), $[Ir(COD)_2]BAr^F_4$ (10 mol%), Ligand (10 mol%) and Dioxane (0.125 M) was heated at the indicated temperature and time following the General Procedure L. [b] Toluene as solvent.

Table S7. Optimization of the hydroarylation of precursor **9a**.[a]

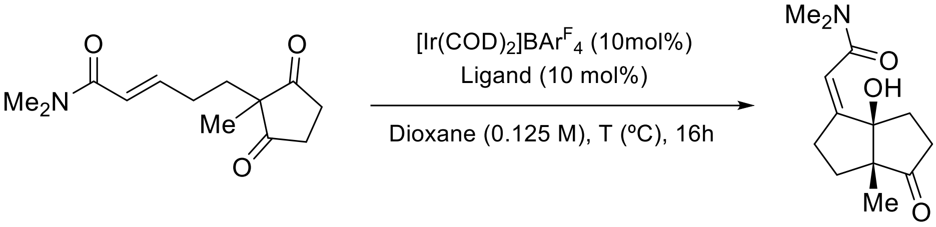

9a **10a**

Entry	Ligand	T (°C)	Yield **9a** (%)	er
1	(*R*)-DTBM-Segphos	130	44	58:12
2	(*R*)-Binap	130	85	77:23
3	(*S*)-C_3-Tunephos	130	83	72:28
4	(*S*)-Binapine	130	0	-
5	(*R,R*)-*i*Pr-Duphos	130	91	69:31
6	(*R,R*)-QuinoxP*	130	74	76:24
7	(*R,R*)-BenzP*	130	99	81:19
8	(*R,R*)-BenzP*	100	44	89:11
9 [b]	(*R,R*)-BenzP*	100	81	90:10

[a] Conditions: A solution of **7** (0.1 mmol), [Ir(COD)$_2$]BAr$^F{}_4$ (10 mol%), Ligand (10 mol%) and Dioxane (0.125 M) was heated at the indicated temperature and time following the General Procedure L. [b] 3 days of reaction time.

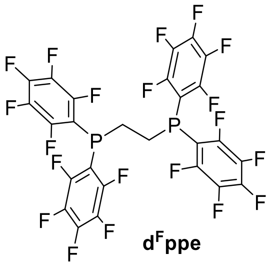

d^Fppe

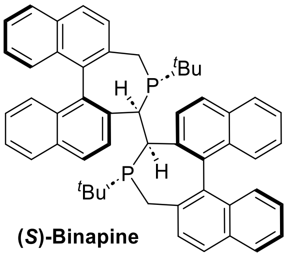

(*S*)-Binapine

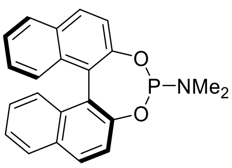

(*R*)-Monophos

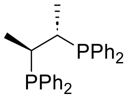

(*S,S*)-Chiraphos

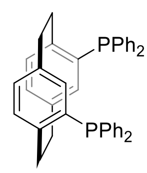

(*S*)-Phanephos

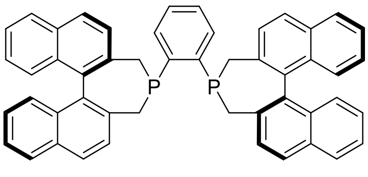

(*R,R*)-Binaphene

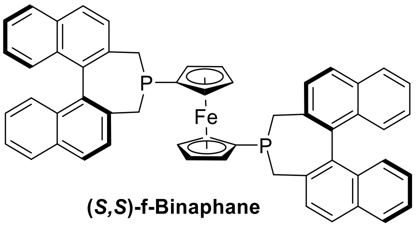

(*S,S*)-f-Binaphane

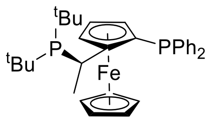

Josiphos SL-J002-1

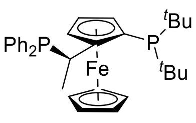

Josiphos SL-J502-2

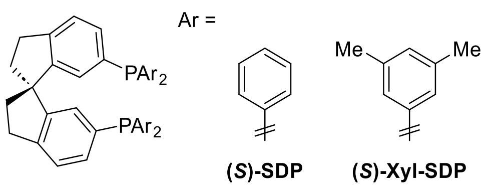

(*S*)-SDP (*S*)-Xyl-SDP

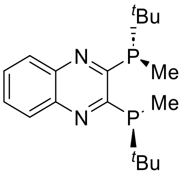

(*R,R*)-QuinoxP*

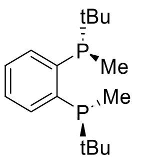

(*R,R*)-BenzP

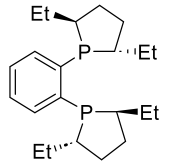

(*R,R*)-Et-Duphos

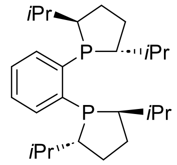

(*R,R*)-*i*Pr-Duphos

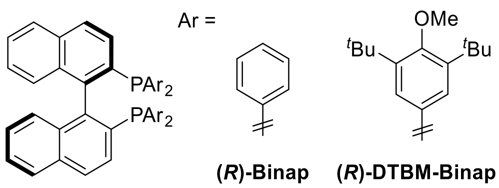

(*R*)-Binap (*R*)-DTBM-Binap

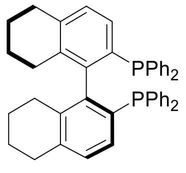

(*S*)-H8-Binap

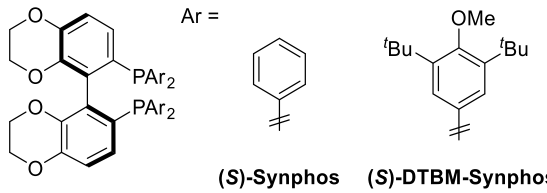

(*S*)-Synphos (*S*)-DTBM-Synphos

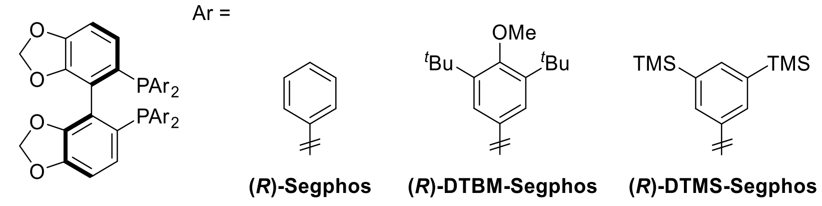

(*R*)-Segphos (*R*)-DTBM-Segphos (*R*)-DTMS-Segphos

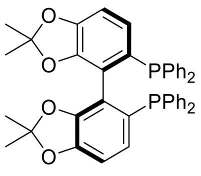

(*R*)-Sunphos

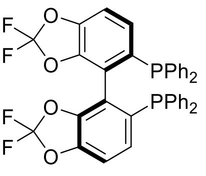

(*R*)-Difluorphos

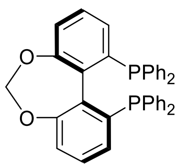

(*S*)-C_1-Tunephos

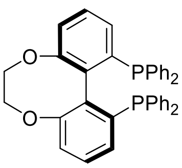

(*S*)-C_2-Tunephos

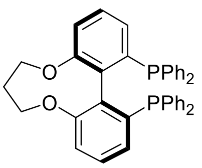

(*S*)-C_3-Tunephos

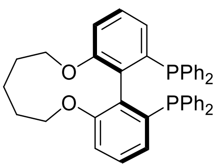

(*S*)-C_5-Tunephos

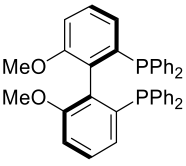

(*R*)-MeO-Biphep

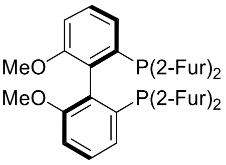

(*R*)-Fur-Biphep

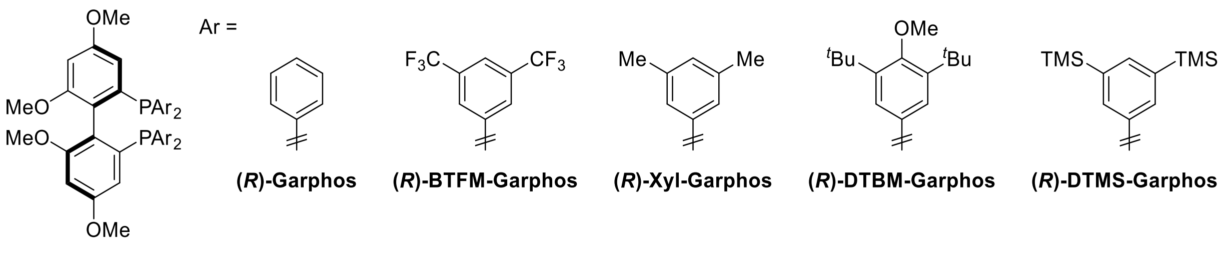

(*R*)-Garphos (*R*)-BTFM-Garphos (*R*)-Xyl-Garphos (*R*)-DTBM-Garphos (*R*)-DTMS-Garphos

3. Procedures for the synthesis of substrates

General Procedure A: Synthesis of 2-alkyl-1,3-diketone substrates (illustrated for **1b**)

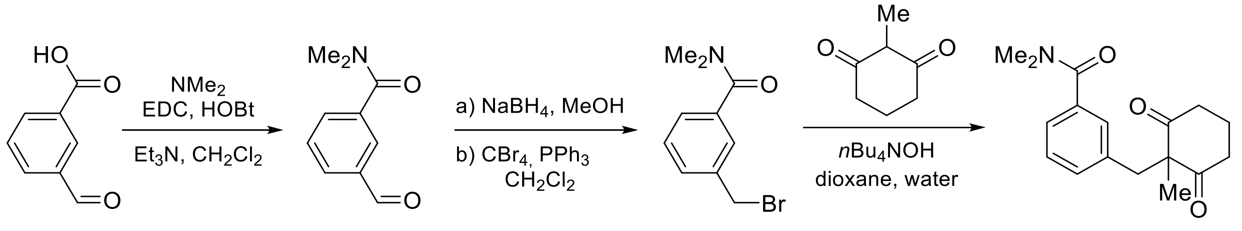

Step 1: Me_2NH (19.1 mL, 38.3 mmol, 2 M in THF, 1.15 equiv) was added to a solution of 3-Formyl-benzoic acid (5.0 g, 33.3 mmol, 1.0 equiv), EDC·HCl (9.6 g, 49.6 mmol, 1.5 equiv), HOBt (5.3 g, 33.3 mmol 1.equiv) and Et_3N (14 mL, 10.1 mol, 3.0 equiv) in CH_2Cl_2 (133 mL) at rt. The resulting mixture was stirred overnight and then quenched with water. The layers were separated, and the aqueous phase was extracted with CH_2Cl_2. The combined organic layers were washed with brine dried, and the solvent was evaporated to afford 5.9 g of 3-Formyl-*N,N*-dimethylbenzamide **S1** (86% yield) as a pale-yellow oil that was used in the next step without further purification.

Step 2a: $NaBH_4$ (1.05 g, 27.8 mmol, 1.5 equiv) was added to a solution of 3-formyl-*N,N*-dimethylbenzamide **S1** (3.28 g, 18.5 mmol, 1.0 equiv) in MeOH (74 mL) at 0 °C. The reaction was stirred at the same temperature until complete consumption of starting material as indicated by TLC. The reaction was quenched with a saturated solution of NH_4Cl in water. The reaction mixture was extracted with EtOAc and the combined organic layers were washed with brine, dried over anhydrous Na_2SO_4, filtered and evaporated under reduced pressure to afford 3.15 g of 3-(hydroxymethyl)-*N,N*-dimethylbenzamide (95% yield) that was used in the next step without further purification.

Step 2b: To a solution of PPh_3 (3.51 g, 13.4 mmol, 1.2 equiv) and 3-(hydroxymethyl)-*N,N*-dimethylbenzamide (2.0 g, 11.2 mmol, 1.0 equiv) in CH_2Cl_2 (56 mL) at rt, CBr_4 (1.3 mL, 13.4 mmol, 1.2 equiv) was added portionwise. The reaction was stirred at the same temperature until complete consumption of starting material as indicated by TLC. Upon completion, the reaction mixture was concentrated, and the resulting crude was purified by flash chromatography on silica gel (9:1 → 8:2 CH_2Cl_2/Et_2O) to afford 2.0 g of 3-(bromomethyl)-*N,N*-dimethylbenzamide **S2** (74% yield) as yellow oil.

Step 3:[3] To a stirred solution of 2-methylcyclohexane-1,3-dione (200 mg, 1.59 mmol, 1.0 equiv) in dioxane (0.8 mL) was added aqueous *n*Bu_4NOH solution (0.59 mL, 1.59 mmol, 1.54 M in water, 1.0 equiv). The mixture was stirred for 5 min at rt. Then a solution of 3-(bromomethyl)-*N,N*-dimethylbenzamide **S2** (461 mg g, 1.90 mmol) in dioxane (0.8 mL) was added and the reaction mixture was stirred until complete consumption of starting material as indicated by TLC (12-36 h). Upon completion, the reaction was quenched with saturated aqueous NH_4Cl solution and extracted with EtOAc. The combined organic layers were dried over anhydrous Na_2SO_4 and evaporated under reduced pressure. The crude residue was purified by flash chromatography (SiO_2-12g, 30 mL/min, 0 → 70% hexane/EtOAc) to give 164 mg of ***N,N*-dimethyl-3-((1-methyl-2,6-dioxocyclohexyl)methyl)benzamide 1b** (36% yield) as white solid. **^{1}H NMR** (300 MHz, $CDCl_3$) δ 7.20 – 7.07 (m, 2H), 7.04 – 6.88 (m, 2H), 3.04 (s, 2H), 2.97 (br s, 3H), 2.83 (br s, 3H), 2.55 – 2.40 (m, 2H), 2.34

– 2.19 (m, 2H), 1.76 – 1.61 (m, 1H), 1.56 – 1.41 (m, 1H), 1.18 (s, 3H) ppm. **^{13}C NMR** (75 MHz, $CDCl_3$) δ 210.72 (CO), 171.09 (CO), 136.74 (C), 136.38 (C), 131.06 (CH), 128.40 (CH), 128.33 (CH), 125.63 (CH), 65.22 (C), 42.53 (CH_2), 39.37 (CH_3 amide), 38.89 (CH_2), 35.18 (CH_3 amide), 22.20 (CH_3), 16.61 (CH_2) ppm. **HRMS** [APCI]: *m/z* calculated for $C_{17}H_{22}NO_3$ $[M + H]^+$: 288.1594, found 288.1597

***N,N*-Diethyl-3-((1-methyl-2,6-dioxocyclohexyl)methyl)benzamide (1a)**

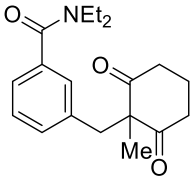

Prepared following General Procedure A, obtained in 24% yield as pale-yellow solid. **^{1}H NMR** (300 MHz, $CDCl_3$) δ 7.31 – 7.17 (m, 2H), 7.09 – 7.02 (m, 1H), 3.52 (br m, 2H), 3.22 (br m, 2H), 3.15 (s, 2H), 2.65 – 2.49 (m, 2H), 2.44 – 2.28 (m, 2H), 1.88 – 1.70 (m, 1H), 1.67 – 1.50 (m, 1H), 1.30 (s, 3H), 1.17 (br m, 6H) ppm. **^{13}C NMR** (75 MHz, $CDCl_3$) δ 210.99 (CO), 170.96 (CO), 137.62 (C), 137.06 (C), 130.89 (CH), 128.63 (CH), 127.84 (CH), 125.23 (CH), 65.41 (C), 43.00 (CH_2), 39.20 (CH_2), 22.50 (CH_3), 16.82 (CH_2) ppm. Note: Amide signals are not visible due to rotamerism. **HRMS** [APCI]: *m/z* calculated for $C_{19}H_{26}NO_3$ $[M + H]^+$: 316.1907, found 316.1909.

2-Methyl-2-(3-(pyrrolidine-1-carbonyl)benzyl)cyclohexane-1,3-dione (1a^I)

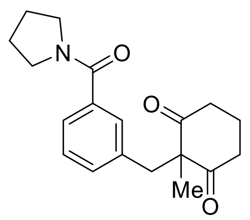

Prepared following General Procedure A, obtained in 17% yield as colorless solid. **^{1}H NMR** (300 MHz, $CDCl_3$) δ 7.36 (d, *J* = 7.6 Hz, 1H), 7.26 (t, *J* = 7.6 Hz, 1H), 7.16 (s, 1H), 7.09 (d, *J* = 7.5 Hz, 1H), 3.61 (t, *J* = 6.8 Hz, 2H), 3.36 (t, *J* = 6.5 Hz, 2H), 3.15 (s, 2H), 2.63 – 2.48 (m, 2H), 2.41 – 2.29 (m, 2H), 2.00 – 1.74 (m, 5H), 1.65 – 1.49 (m, 1H), 1.29 (s, 3H) ppm. **^{13}C NMR** (75 MHz, $CDCl_3$) δ 210.95 (CO), 169.32 (CO), 137.46 (C), 136.73 (C), 131.43 (CH), 128.47 (CH), 125.91 (CH), 65.32 (C), 49.47 (CH_2), 46.16 (CH_2), 42.79 (CH_2), 39.10 (CH_2), 26.41 (CH_2), 24.44 (CH_2), 22.49 (CH_3), 16.71 (CH_2) ppm. **HRMS** [APCI]: *m/z* calculated for $C_{19}H_{24}NO_3$ $[M + H]^+$: 314.1751, found 314.1753.

***N*-Methoxy-*N*-methyl-3-((1-methyl-2,6-dioxocyclohexyl)methyl)benzamide (1a^{II})**

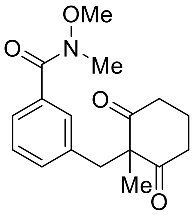

Prepared following General Procedure A, obtained in 56% yield as colorless oil. **^{1}H NMR** (300 MHz, $CDCl_3$) δ 7.45 (d, *J* = 7.9 Hz, 1H), 7.28 (s, 1H), 7.23 (t, *J* = 7.4 Hz, 1H), 7.09 (d, *J* = 7.9 Hz, 1H), 3.50 (s, 3H), 3.28 (s, 3H), 3.12 (s, 2H), 2.59 – 2.47 (m, 2H), 2.40 – 2.26 (m, 2H), 1.85 – 1.67 (m, 1H), 1.62 – 1.48 (m, 1H), 1.25 (s, 3H) ppm. **^{13}C NMR** (75 MHz, $CDCl_3$) δ 210.84 (CO), 169.66 (CO), 136.51 (C), 134.36 (C), 132.21 (CH), 129.47 (CH), 128.20 (CH), 126.90 (CH), 65.38 (C), 61.08 (CH_3), 42.90 (CH_2), 39.07 (CH_2), 33.69 (CH_3), 22.29 (CH_3), 16.75 (CH_2) ppm. **HRMS** [APCI]: *m/z* calculated for $C_{17}H_{22}NO_4$ $[M + H]^+$: 304.1543, found 304.1533.

Methyl 3-((1-methyl-2,6-dioxocyclohexyl)methyl)benzoate (1a^{III})

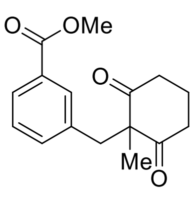

Prepared following General Procedure A, from commercial methyl 3-(bromomethyl)benzoate. Obtained in 73% yield as white solid. **^{1}H NMR** (300 MHz, $CDCl_3$) δ 7.84 (dd, *J* = 7.5, 1.7 Hz, 1H), 7.68 (s, 1H), 7.33 – 7.16 (m, 2H), 3.87 (s, 3H), 3.16 (s, 2H), 2.57 (m, 2H), 2.34 (m, 2H), 1.79 (m, 1H), 1.54 (m, 1H), 1.28 (s, 3H) ppm. **^{13}C NMR** (75 MHz, $CDCl_3$) δ 210.79 (CO), 166.94 (CO), 137.30 (C), 134.63

(CH), 130.99 (CH), 130.36 (C), 128.50 (CH), 128.27 (CH), 65.37 (C), 52.21(CH_3), 42.45 (CH_2), 39.05 (CH_2), 22.72 (CH_3), 16.79 (CH_2) ppm. **HRMS** [APCI]: *m/z* calculated for $C_{16}H_{19}O_4$ $[M + H]^+$: 275.1278, found 275.1271.

2-(3-Acetylbenzyl)-2-methylcyclohexane-1,3-dione (1a^{IV})

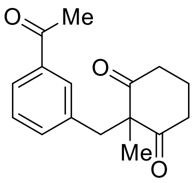

Prepared following General Procedure A, from commercial 1-(3-(chloromethyl)phenyl)ethan-1-one. Obtained in 21% yield as colorless oil. **^{1}H NMR** (300 MHz, $CDCl_3$) δ 7.76 (d, *J* = 7.6 Hz, 1H), 7.61 (s, 1H), 7.36 – 7.22 (m, 2H), 3.19 (s, 2H), 2.66 – 2.56 (m, 2H), 2.54 (s, 3H), 2.43 – 2.27 (m, 2H), 1.90 – 1.72 (m, 1H), 1.65 – 1.47 (m, 1H), 1.30 (s, 3H) ppm. **^{13}C NMR** (75 MHz, $CDCl_3$) δ 210.74 (CO), 197.97 (CO), 137.51 (C), 137.24 (C), 134.76 (CH), 130.00 (CH), 128.66 (CH), 126.81 (CH), 65.43 (C), 42.21 (CH_2), 38.98 (CH_2), 26.69 (CH_3), 22.84 (CH_3), 16.77 (CH_2) ppm. **HRMS** [APCI]: *m/z* calculated for $C_{16}H_{19}O_3$ $[M + H]^+$: 259.1329, found 259.1324.

3-((2,6-Dioxo-1-propylcyclohexyl)methyl)-*N,N*-dimethylbenzamide (1c)

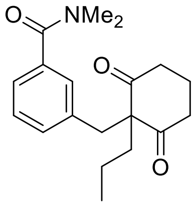

Prepared following General Procedure A, obtained in 93% yield as white solid. **^{1}H NMR** (300 MHz, $CDCl_3$) δ 7.18 – 7.04 (m, 2H), 6.97 – 6.84 (m, 2H), 2.95 (br s, *J* = 4.9 Hz, 6H), 2.79 (br s, 3H), 2.30 (ddd, *J* = 17.2, 8.5, 4.9 Hz, 2H), 2.01 (ddd, *J* = 17.2, 8.3, 4.9 Hz, 2H), 1.76 – 1.66 (m, 2H), 1.66 – 1.50 (m, 1H), 1.24 – 1.10 (m, 1H), 0.96 (dq, *J* = 14.7, 7.3 Hz, 2H), 0.71 (t, *J* = 7.2 Hz, 3H) ppm. **^{13}C NMR** (75 MHz, $CDCl_3$) δ 212.00 (CO), 170.94 (CO), 136.81 (C), 136.54 (C), 130.88 (CH), 128.47 (CH), 128.15 (CH), 125.63 (CH), 69.15 (C), 43.75 (CH_2), 41.03 (CH_2), 40.69 (CH_2), 39.27 (CH_3 amide), 35.10 (CH_3 amide), 18.64 (CH_2), 15.64 (C)H2, 14.33 (CH_3) ppm. **HRMS** [APCI]: *m/z* calculated for $C_{19}H_{26}NO_3$ $[M + H]^+$: 316.1907, found 316.1905.

Methyl 3-(1-(3-(dimethylcarbamoyl)benzyl)-2,6-dioxocyclohexyl)propanoate (1d)

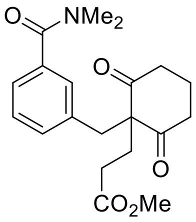

Prepared following General Procedure A, obtained in 23% yield as pale-yellow solid. **^{1}H NMR** (300 MHz, $CDCl_3$) δ 7.31 – 7.21 (m, 2H), 7.06 – 6.96 (m, 2H), 3.61 (s, 2H), 3.07 (br s, 5H), 2.91 (br s, 3H), 2.58 – 2.40 (m, 3H), 2.32 – 2.18 (m, 2H), 2.16 (s, 3H), 1.83 – 1.66 (m, 1H), 1.53 – 1.33 (m, 1H) ppm. **^{13}C NMR** (75 MHz, $CDCl_3$) δ 210.87 (CO), 172.84 (CO), 171.15 (C), 136.80 (C), 136.12 (CH), 131.23 (CH), 128.78 (CH), 128.48 (CH), 126.11 (CH), 68.26 (C), 51.78 (CH_3), 43.73 (CH_2), 40.20 (CH_2), 39.54 (CH_3 amide), 35.36 (CH_3 amide), 31.44 (CH_2), 29.63 (CH_2), 16.15 (CH_2) ppm. **HRMS** [APCI]: *m/z* calculated for $C_{20}H_{26}NO_5$ $[M + H]^+$: 360.1805, found 360.1808.

3-((1-*iso*Butyl-2,6-dioxocyclohexyl)methyl)-*N,N*-dimethylbenzamide (1e)

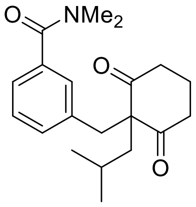

Prepared following General Procedure A, obtained in 66% yield as white solid. **^{1}H NMR** (300 MHz, $CDCl_3$) δ 7.23 – 7.10 (m, 2H), 6.99 – 6.86 (m, 2H), 2.97 (br m, 5H), 2.83 (br s, 3H), 2.47 – 2.32 (m, 2H), 2.07 – 1.94 (m, 2H), 1.90 – 1.75 (m, 2H), 1.75 – 1.57 (m, 1H), 1.45 – 1.28 (m, 1H), 1.28 – 1.09 (m, 1H), 0.81 – 0.62 (m, 6H) ppm. **^{13}C NMR** (75 MHz, $CDCl_3$) δ 212.19 (CO), 170.98 (CO), 136.69 (C), 136.57 (C), 131.01 (CH), 128.62 (CH), 128.26 (CH), 125.87 (CH), 68.56 (C), 47.78 (CH_2), 45.95 (CH_2),

40.74 (CH_2), 39.36 (CH_3 amide), 35.20 (CH_3 amide), 25.43 (CH), 24.04 (CH_3), 15.77 (CH_2) ppm. **HRMS** [APCI]: *m/z* calculated for $C_{20}H_{28}NO_3$ $[M + H]^+$: 330.2064, found 330.2064.

3-((1-Benzyl-2,6-dioxocyclohexyl)methyl)-*N,N*-dimethylbenzamide (1f)

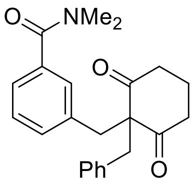

Prepared following General Procedure A, obtained in 61% yield as white solid. **^{1}H NMR** (300 MHz, $CDCl_3$) δ 7.23 – 7.07 (m, 5H), 7.07 – 6.85 (m, 4H), 3.18 (dd, *J* = 11.7, 3.8 Hz, 4H), 3.01 (br s, 3H), 2.85 (br s, 3H), 1.93 – 1.69 (m, 4H), 0.89 – 0.72 (m, 2H) ppm. **^{13}C NMR** (75 MHz, $CDCl_3$) δ 212.62 (CO), 171.00 (CO), 136.78 (C), 136.54 (C), 136.08 (C), 131.05 (CH), 129.86 (CH), 128.73 (CH), 128.58 (CH), 128.30 (CH_2), 127.13 (CH_2), 125.90 (CH_2), 70.58 (C), 45.39 (CH_2), 44.37 (CH_2), 41.47 (CH_2), 39.38 (CH_3 amide), 35.25 (CH_3 amide), 14.64 (CH_2) ppm. **HRMS** [APCI]: *m/z* calculated for $C_{23}H_{26}NO_3$ $[M + H]^+$: 364.1907, found 364.1908.

3-((2,6-dioxo-1-(3-phenylpropyl)cyclohexyl)methyl)-*N,N*-dimethylbenzamide (1g)

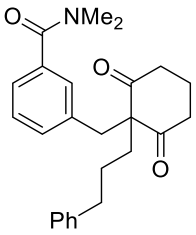

Prepared following General Procedure A, obtained in 56% yield as colorless oil. **^{1}H NMR** (300 MHz, $CDCl_3$) δ 7.27 – 7.18 (m, 4H), 7.17 – 7.11 (m, 1H), 7.11 – 7.04 (m, 2H), 7.04 – 6.96 (m, 2H), 3.05 (s, 5H), 2.87 (s, 3H), 2.49 (t, *J* = 7.7 Hz, 2H), 2.44 – 2.29 (m, 2H), 2.18 – 2.03 (m, 2H), 1.97 – 1.85 (m, 2H), 1.71 – 1.55 (m, 1H), 1.44 – 1.17 (m, 3H) ppm. **^{13}C NMR** (75 MHz, $CDCl_3$) 211.95 (CO), 171.03 (CO), 141.28 (C), 136.74 (C), 136.71 (C), 131.01 (CH), 128.63 (CH), 128.34 (CH), 128.26 (CH), 125.93 (CH), 125.84 (CH), 69.09 (C), 43.97 (CH_2), 40.75 (CH_2), 39.37 (CH_3 amide), 38.38 (CH_2), 36.12 (CH_2), 35.24 (CH_3 amide), 27.21 (CH_2), 15.75 (CH_2) ppm. **HRMS** [APCI]: *m/z* calculated for $C_{25}H_{30}NO_3$ $[M + H]^+$: 392.2220, found 392.2234.

***N,N*-Dimethyl-3-((1,4,4-trimethyl-2,6-dioxocyclohexyl)methyl)benzamide (1i)**

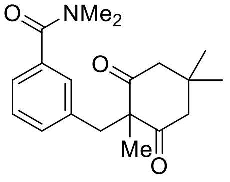

Prepared following General Procedure A, obtained in 72% yield as pale-yellow solid. **^{1}H NMR** (300 MHz, $CDCl_3$) δ 7.21 – 7.10 (m, 2H), 7.09 – 6.97 (m, 2H), 3.01 (br m, 5H), 2.84 (s, 3H), 2.42 (d, *J* = 2.5 Hz, 4H), 1.16 (d, *J* = 3.8 Hz, 3H), 0.81 (d, *J* = 3.2 Hz, 3H), 0.67 (d, *J* = 3.1 Hz, 3H) ppm. **^{13}C NMR** (75 MHz, $CDCl_3$) δ 209.97 (CO), 171.08 (CO), 136.23 (C), 131.59 (CH), 128.88 (CH), 128.18 (CH), 125.66 (CH), 64.75 (C), 52.08 (CH_2), 42.35 (CH_2), 39.35 (CH_3 amide), 35.15 (CH_3 amide), 30.24 (C), 28.50 (CH_3), 28.27 (CH_3), 20.96 (CH_3) ppm. **HRMS** [APCI]: *m/z* calculated for $C_{19}H_{26}NO_3$ $[M + H]^+$: 316.1907, found 316.1907.

3-((4,4-Dimethyl-2,6-dioxo-1-propylcyclohexyl)methyl)-*N,N*-diethylbenzamide (1j)

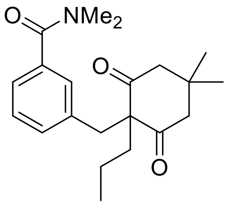

Prepared following General Procedure A, obtained in 80% yield as white solid. **^{1}H NMR** (300 MHz, $CDCl_3$) δ 7.20 – 7.04 (m, 4H), 3.03 (d, *J* = 4.1 Hz, 2H), 2.98 (br s, 3H), 2.83 (br s, 3H), 2.44 (dd, *J* = 15.5, 3.6 Hz, 2H), 2.29 (dd, *J* = 15.4, 3.4 Hz, 2H), 1.67 – 1.58 (m, 2H), 1.19 – 1.03 (m, 2H), 0.92 – 0.71 (m, 6H), 0.40 (d, *J* = 4.0 Hz, 3H) ppm. **^{13}C NMR** (75 MHz, $CDCl_3$) δ 209.50 (CO), 209.48 (CO), 171.26 (C), 136.80 (C), 135.89 (C), 132.36 (CH), 129.74 (CH), 127.93 (CH), 125.42 (CH), 69.32 (C), 52.18 (CH_2), 39.38 (CH_2), 39.34 (CH_2), 35.16 (CH_3 amide), 30.11 (C), 29.20 (CH_3), 27.50 (CH_3), 17.83 (CH_2), 14.26 (CH_3) ppm. **HRMS** [APCI]: *m/z* calculated for $C_{21}H_{30}NO_3$ $[M + H]^+$: 344.2220, found 344.2221.

_N,N_-dimethyl-3-((8-methyl-7,9-dioxospiro[4.5]decan-8-yl)methyl)benzamide (1k)

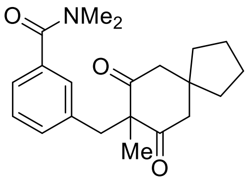

Prepared following General Procedure A, obtained in 49% yield as colorless oil. **^{1}H NMR** (300 MHz, $CDCl_3$) δ 7.23 – 7.14 (m, 2H), 7.08 – 6.99 (m, 2H), 3.04 (s, 2H), 3.01 (s, 3H), 2.85 (s, 3H), 2.51 (d, *J* = 15.3 Hz, 2H), 2.41 (d, *J* = 15.3 Hz, 2H), 1.61 – 1.42 (m, 4H), 1.23 (t, *J* = 7.2 Hz, 2H), 1.18 (s, 3H), 1.04 (t, *J* = 7.0 Hz, 2H) ppm. **^{13}C NMR** (75 MHz, $CDCl_3$) δ 210.11 (CO), 171.20 (CO), 136.48 (C), 136.34 (C), 131.55 (CH), 128.87 (CH), 128.29 (CH), 125.75 (CH), 64.88 (C), 50.81 (CH_2), 42.71 (CH_2), 40.06 (C), 39.44 (CH_3 amide), 38.16 (CH_2), 37.70 (CH_2), 35.26 (CH_3 amide), 24.17 (CH_2), 23.94 (CH_2), 21.40 (CH_3) ppm. **HRMS** [APCI]: *m/z* calculated for $C_{21}H_{28}NO_3$ $[M + H]^+$: 342.2064, found 342.2066.

_N,N_-Dimethyl-3-((3-methyl-2,4-dioxospiro[5.5]undecan-3-yl)methyl)benzamide (1l)

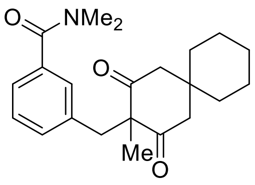

Prepared following General Procedure A, obtained in 90% yield as colorless oil. **^{1}H NMR** (300 MHz, $CDCl_3$) δ 7.20 – 7.07 (m, 2H), 7.05 – 6.93 (m, 2H), 2.96 (br m, 5H), 2.81 (br s, 3H), 2.49 (dd, *J* = 15.0, 2.7 Hz, 2H), 2.37 (dd, *J* = 15.0, 1.9 Hz, 2H), 1.38 – 1.14 (m, 6H), 1.14 – 1.00 (m, 5H), 0.97 – 0.79 (m, 2H) ppm. **^{13}C NMR** (75 MHz, $CDCl_3$) δ 209.90 (CO), 170.95 (CO), 136.15 (C), 131.44 (CH), 128.79 (CH), 128.03 (CH), 125.55 (CH), 65.14 (C), 49.75 (CH_2), 42.40 (CH_2), 39.28 (CH_3 amide), 36.65 (CH_2), 36.26 (CH_2), 35.06 (CH_3 amide), 33.00 (C), 25.01 (CH_2), 20.98 (CH_2), 20.95 (CH_2), 20.70 (CH_3) ppm. **HRMS** [APCI]: *m/z* calculated for $C_{22}H_{30}NO_3$ $[M + H]^+$: 356.2220, found 356.2222.

_N,N_-Dimethyl-3-((1-methyl-2,7-dioxocycloheptyl)methyl)benzamide (1m)

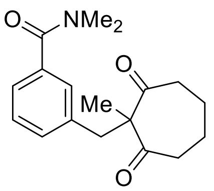

Prepared following General Procedure A, obtained in 48% yield as white solid. **^{1}H NMR** (300 MHz, $CDCl_3$) δ 7.25 – 7.14 (m, 2H), 7.07 – 6.93 (m, 2H), 3.08 (br s, *J* = 2.2 Hz, 2H), 3.02 (br s, 3H), 2.88 (br s, 3H), 2.45 – 2.32 (m, 4H), 1.89 – 1.72 (m, 4H), 1.10 (br d, *J* = 2.1 Hz, 3H) ppm. **^{13}C NMR** (75 MHz, $CDCl_3$) δ 211.66 (CO), 171.26 (CO), 136.43 (C), 136.35 (C), 131.26 (CH), 128.53 (CH), 128.32 (CH), 125.59 (CH), 65.89 (C), 41.75 (CH_2), 39.69 (CH_2), 39.47 (CH_3 amide), 35.26 (CH_3 amide), 27.87 (CH_2), 18.92 (CH_3) ppm. **HRMS** [APCI]: *m/z* calculated for $C_{18}H_{24}NO_3$ $[M + H]^+$: 302.1751, found 302.1755.

4-methoxy-_N,N_-dimethyl-3-((1-methyl-2,6-dioxocyclohexyl)methyl)benzamide (1n)

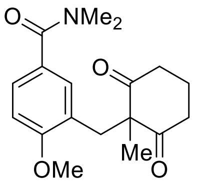

Prepared following General Procedure A, obtained in 42% yield as pale-yellow solid. **^{1}H NMR** (300 MHz, $CDCl_3$) δ 7.27 (d, *J* = 8.5 Hz, 1H), 7.00 (s, 1H), 6.74 (d, *J* = 8.4 Hz, 1H), 3.67 (s, 3H), 3.00 (s, 2H), 2.95 (s, 6H), 2.84 – 2.70 (m, 2H), 2.60 – 2.42 (m, 2H), 2.08 – 1.90 (m, 1H), 1.77 – 1.56 (m, 1H), 1.02 (s, 3H) ppm. **^{13}C NMR** (75 MHz, $CDCl_3$) δ 209.47 (CO), 171.08 (CO), 158.28 (C), 131.42 (CH), 128.38 (CH), 128.15 (C), 123.74 (C), 110.11 (CH), 65.53 (C), 54.90 (CH_3), 39.72 (CH_3 amide), 38.89 (CH_2), 38.19 (CH_2), 35.54 (CH_3 amide), 17.68 (CH_2), 17.17 (CH_3) ppm. **HRMS** [APCI]: *m/z* calculated for $C_{18}H_{24}NO_4$ $[M + H]^+$: 318.1700, found 318.1699.

_N,N_-dimethyl-5-((1-methyl-2,6-dioxocyclohexyl)methyl)-[1,1'-biphenyl]-3-carboxamide (1o)

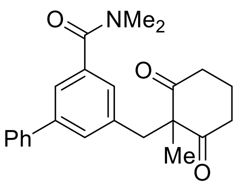

Prepared following General Procedure A , obtained in 57% yield white foam. **^{1}H NMR** (300 MHz, $CDCl_3$) δ 7.52 – 7.43 (m, 3H), 7.41 – 7.32 (m, 2H), 7.32 – 7.24 (m, 2H), 6.98 (s, 1H), 3.16 (s, 2H), 3.04 (s, 3H), 2.90 (s, 3H), 2.62 – 2.45 (m, 2H), 2.41 – 2.24 (m, 2H), 1.85 – 1.65 (m, 1H), 1.63 – 1.46 (m, 1H), 1.26 (s, 3H) ppm. **^{13}C NMR** (75 MHz, $CDCl_3$) δ 210.76 (CO), 171.09 (CO), 141.46 (C), 139.90 (C), 137.41 (C), 137.04 (C), 129.85 (CH), 128.83 (CH), 127.72 (CH), 127.16 (CH), 127.03 (CH), 124.44 (CH), 65.32 (C), 42.62 (CH_2), 39.48 (CH_3 amide), 39.01 (CH_2), 35.28 (CH_3 amide), 22.51 (CH_3), 16.71 (CH_2) ppm. **HRMS** [APCI]: *m/z* calculated for $C_{23}H_{26}NO_3$ $[M + H]^+$: 364.1907, found 364.1916.

_N,N_-Dimethyl-3-((4-methyl-3,5-dioxotetrahydro-2H-pyran-4-yl)methyl)benzamide (1p)

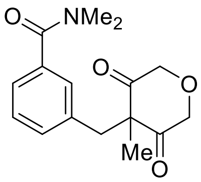

Prepared following General Procedure A, obtained in 75% yield as colorless oil. **^{1}H NMR** (300 MHz, $CDCl_3$) δ 7.33 – 7.22 (m, 2H), 7.14 – 7.03 (m, 2H), 4.18 (d, *J* = 3.5 Hz, 4H), 3.24 (d, *J* = 4.3 Hz, 2H), 3.06 (br s, 3H), 2.92 (br s, 3H), 1.28 (d, *J* = 5.2 Hz, 3H) ppm. **^{13}C NMR** (75 MHz, $CDCl_3$) δ 207.49 (CO), 171.11 (CO), 136.52 (C), 135.69 (C), 131.24 (CH), 128.58 (CH), 128.50 (CH), 125.91 (CH), 73.52 (C), 64.69 (CH_2), 40.90 (CH_2), 39.43 (CH_3 amide), 35.26 (CH_3 amide), 18.37 (CH_3) ppm. **HRMS** [APCI]: *m/z* calculated for $C_{16}H_{20}NO_4$ $[M + H]^+$: 290.1387, found 290.1392.

_N,N_-Dimethyl-3-((1-methyl-2,5-dioxocyclopentyl)methyl)benzamide (1v)

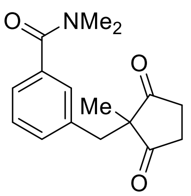

Prepared following General Procedure A, obtained in 50% yield as colorless oil. **^{1}H NMR** (300 MHz, $CDCl_3$) δ 7.27 – 7.16 (m, 2H), 7.06 – 6.97 (m, 2H), 3.02 (br s, 3H), 2.89 (br d, *J* = 12.9 Hz, 5H), 2.63 – 2.49 (m, 2H), 2.16 – 2.05 (m, 2H), 1.14 (s, 3H) ppm. **^{13}C NMR** (75 MHz, $CDCl_3$) δ 216.65 (CO), 170.98 (CO), 136.68 (C), 135.90 (C), 130.81 (CH), 128.73 (CH), 128.10 (CH), 126.06 (CH), 58.06 (C), 41.92 (CH_2), 39.41 (CH_3 amide), 35.63 (CH_2), 35.28 (CH_3 amide), 20.24 (CH_3) ppm. **HRMS** [APCI]: *m/z* calculated for $C_{16}H_{20}NO_3$ $[M + H]^+$: 274.1438, found 274.1442.

3-((2,5-Dioxo-1-propylcyclopentyl)methyl)-_N,N_-dimethylbenzamide (1w)

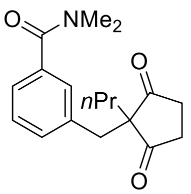

Prepared following General Procedure A, obtained in 44% yield as colorless oil. **^{1}H NMR** (300 MHz, $CDCl_3$) δ 7.33 – 7.20 (m, 2H), 7.13 – 7.01 (m, 2H), 3.09 (s, 3H), 2.96 (s, 2H), 2.92 (s, 3H), 2.61 – 2.39 (m, 2H), 2.19 – 1.94 (m, 2H), 1.79 – 1.68 (m, 2H), 1.25 – 1.06 (m, 2H), 0.85 (t, *J* = 7.2 Hz, 3H) ppm. **^{13}C NMR** (75 MHz, $CDCl_3$) δ 217.73 (CO), 171.14 (CO), 136.89 (C), 135.90 (C), 131.01 (CH), 128.93 (CH), 128.25 (CH), 126.25 (CH), 63.11 (C), 42.03 (CH_2), 39.56 (CH_3 amide), 38.82 (CH_3), 36.78 (CH_2), 35.45 (CH_3 amide), 18.34 (CH_2), 14.53 (CH_3) ppm. **HRMS** [APCI]: *m/z* calculated for $C_{18}H_{24}NO_3$ $[M + H]^+$: 302.1751, found 302.1751.

4-methoxy-*N,N*-dimethyl-3-((1-methyl-2,5-dioxocyclopentyl)methyl)benzamide (1y)

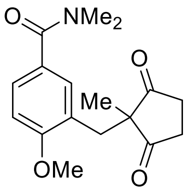

Prepared following General Procedure A, obtained in 30% yield as white solid. **^{1}H NMR** (300 MHz, $CDCl_3$) δ 7.35 – 7.24 (m, 1H), 7.09 – 7.01 (m, 1H), 6.82 – 6.72 (m, 1H), 3.70 (s, 3H), 2.99 (s, 6H), 2.87 (s, 2H), 2.55 (s, 4H), 1.06 (s, 3H) ppm. **^{13}C NMR** (75 MHz, $CDCl_3$) δ 215.16 (CO), 170.98 (CO), 158.23 (C), 131.12 (CH), 128.51 (CH), 128.30 (C), 123.43 (C), 110.11 (CH), 57.00 (C), 54.98 (CH_3), 39.49 (CH_3 amide), 37.06 (CH_2), 35.58 (CH_3 amide), 35.18 (CH_2), 17.52 (CH_3) ppm. **HRMS** [APCI]: *m/z* calculated for $C_{17}H_{22}NO_4$ $[M + H]^+$: 304.1543, found 304.1548.

***N,N*-dimethyl-5-((1-methyl-2,5-dioxocyclopentyl)methyl)-[1,1'-biphenyl]-3-carboxamide (1z)**

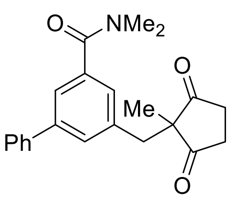

Prepared following General Procedure A, obtained in 33% yield as colorless oil. **^{1}H NMR** (300 MHz, $CDCl_3$) δ 7.54 – 7.44 (m, 3H), 7.42 – 7.34 (m, 2H), 7.34 – 7.27 (m, 2H), 6.99 (s, 1H), 3.05 (s, 3H), 3.00 (s, 2H), 2.92 (s, 3H), 2.72 – 2.47 (m, 2H), 2.30 – 2.04 (m, 2H), 1.18 (s, 3H) ppm. **^{13}C NMR** (75 MHz, $CDCl_3$) δ 216.57 (CO), 170.91 (CO), 141.71 (C), 139.68 (C), 137.24 (C), 136.48 (C) 129.48 (CH), 128.88 (CH), 127.83 (CH), 127.02 (CH), 126.85 (CH), 124.76 (CH), 58.12 (C), 41.90 (CH_2), 39.45 (CH_3 amide), 35.66 (CH_2), 35.31 (CH_3 amide), 20.36 (CH_3) ppm. **HRMS** [APCI]: *m/z* calculated for $C_{22}H_{24}NO_3$ $[M + H]^+$: 350.1751, found 350.1753.

***N,N*-Dimethyl-3-((2-methyl-1,3-dioxo-2,3-dihydro-1*H*-inden-2-yl)methyl)benzamide (1α)**

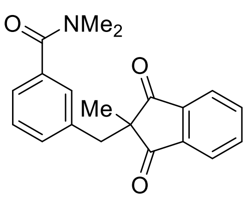

Prepared following General Procedure A, obtained in 77% yield as pale-yellow solid. **^{1}H NMR** (300 MHz, $CDCl_3$) δ 7.82 – 7.72 (m, 2H), 7.68 (ddd, *J* = 4.8, 3.2, 1.5 Hz, 2H), 7.07 – 6.94 (m, 3H), 3.14 (s, 2H), 3.01 (br s, 3H), 2.68 (br s, 3H), 1.36 (s, 3H) ppm. **^{13}C NMR** (75 MHz, $CDCl_3$) δ 203.77 (CO), 171.25 (CO), 141.48 (C), 136.19 (C), 135.90 (C), 135.80 (CH), 131.04 (CH), 128.55 (CH), 128.14 (CH), 125.86 (CH), 123.17 (CH), 55.84 (C), 41.16 (CH_2), 39.33 (CH_3 amide), 35.36 (CH_3 amide), 20.39 (CH_3) ppm. **HRMS** [APCI]: *m/z* calculated for $C_{20}H_{20}NO_3$ $[M + H]^+$: 322.1438, found 322.1437.

General Procedure B: Synthesis of 2-aryl-1,3-diketone substrates (illustrated for 1h)

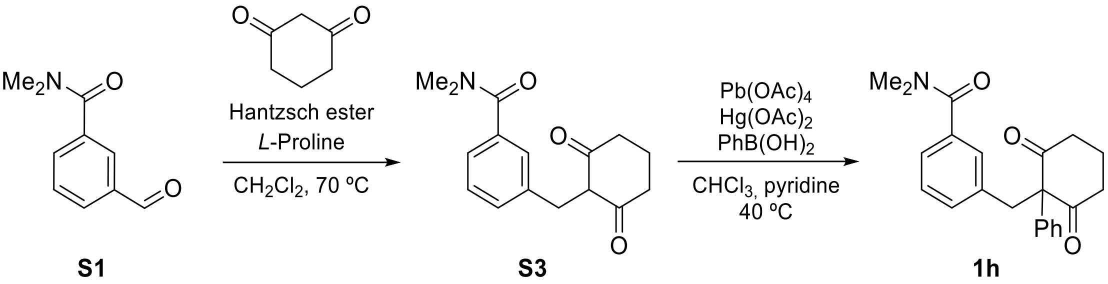

Step 1:[4] *L*-Proline (107 mg, 0.93 mmol, 20 mol%) was added to a sealed tube containing a solution of 1,3-cyclohexadione (520 mg, 4.638 mmol, 1.0 equiv), Hantzsch ester (1.17 g, 4.6 mmol, 1.0 equiv), and the 3-formyl-*N,N*-dimethylbenzamide **S1** (904 mg, 5.1 mmol, 1.1 equiv) in dichloromethane (9 mL). The reaction mixture was stirred at 70 °C until complete consumption of starting material as indicated by TLC. The solvent was removed under reduced pressure, and the crude residue was purified by flash chromatography (SiO_2-12g, 30 mL/min, 0 → 70% hexane/EtOAc), to afford 0.96 g of 3-((2,6-dioxocyclohexyl)methyl)-*N,N*-dimethylbenzamide **S3** (75% yield) as a white foam. **^{1}H NMR** (300 MHz, $CDCl_3$) δ 10.61 (s, 1H), 7.27 (d, *J* = 8.4 Hz, 2H), 7.18 (t, *J* = 7.5 Hz, 1H), 7.09 (d, *J* = 7.5 Hz, 1H), 3.58 (s, 2H), 3.05 (s, 3H), 2.92 (s, 3H), 2.28 (t, *J* = 6.3 Hz, 4H), 1.91 – 1.66 (m, 2H) ppm. **^{13}C NMR** (75 MHz, $CDCl_3$) δ 172.39 (CO), 142.19 (C), 134.66 (C), 130.16 (CH), 127.79 (CH), 127.03 (CH), 123.73 (CH), 114.77 (C), 39.52 (CH_3 amide), 35.30 (CH_3 amide), 32.59 (CH_2), 27.34 (CH_2), 20.57 (CH_2) ppm. **HRMS** [APCI]: *m/z* calculated for $C_{16}H_{20}NO_3$ $[M + H]^+$: 274.1438, found 274.1438.

Step 2:[5] To a stirred suspension of $Pd(OAc)_4$ (182 mg, 0.4 mmol, 1.0 equiv) and $Hg(OAc)_2$ (13 mg, 0.04 mmol, 0.1 equiv) in chloroform (0.6 mL), phenyl boronic acid (50 mg, 0.4 mmol, 1.0 equiv) was added over 15 min. The mixture was stirred 1 h at 40 °C, and a solution of 3-((2,6-dioxocyclohexyl)methyl)-*N,N*-dimethylbenzamide **S3** (100 mg, 0.4 mmol, 1.0 equiv) in pyridine (0.5 mL) was added. The reaction mixture was stirred 1h at 40 °C, followed by 16h at rt. The resulting suspension was diluted with chloroform (100 mL), filtered through celite, concentrated under reduced pressure and purified by flash chromatography (SiO_2-12g, 30 mL/min, 0 → 100%), to afford 80 mg **3-((2,6-dioxo-1-phenylcyclohexyl)methyl)-*N,N*-dimethylbenzamide 1h** (57% yield) as a brown oil. **^{1}H NMR** (300 MHz, $CDCl_3$) δ 7.53 – 7.36 (m, 3H), 7.36 – 7.20 (m, 4H), 7.19 – 7.06 (m, 2H), 3.61 (s, 2H), 3.23 (s, 3H), 3.04 (s, 3H), 2.98 – 2.82 (m, 2H), 2.72 – 2.57 (m, 2H), 2.11 – 1.92 (m, 1H), 1.89 – 1.69 (m, 1H) ppm. **^{13}C NMR** (75 MHz, $CDCl_3$) δ 207.3 (CO), 171.8 (CO), 137.7 (C), 137.3 (C), 135.7 (C), 132.5 (CH), 130.0 (CH), 129.4 (CH), 128.0 (CH), 127.8 (CH), 127.3 (CH), 125.1 (CH), 76.7 (C), 39.6 (CH_2), 39.5 (CH_2), 35.4 (2x CH_3 amide), 17.2 (CH_2) ppm. **HRMS** [APCI]: *m/z* calculated for $C_{22}H_{24}NO_3$ $[M + H]^+$: 350.1751, found 350.1748.

3-((2,5-Dioxo-1-phenylcyclopentyl)methyl)-*N,N*-dimethylbenzamide (1x)

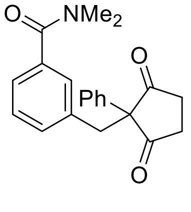

Prepared following General Procedure B, obtained in 27% yield as a brown oil. **^{1}H NMR** (300 MHz, $CDCl_3$) δ 7.38 – 7.16 (m, 9H), 3.36 (s, 2H), 3.09 (s, 3H), 2.93 (s, 3H), 2.81 – 2.66 (m, 2H), 2.36 – 2.23 (m, 2H) ppm. **^{13}C NMR** (75 MHz, $CDCl_3$) δ 213.17 (CO), 171.36 (C), 136.43 (C), 136.11 (C), 135.44 (C), 131.95 (CH), 129.42 (CH), 129.25 (CH), 128.67 (CH), 128.34 (CH), 126.92 (CH), 126.11 (CH), 67.18 (C),

41.95 (CH_2), 39.62 (CH_3 amide), 36.11 (CH_2), 35.44 (CH_3 amide) ppm. **HRMS** [APCI]: *m/z* calculated for $C_{21}H_{22}NO_3$ $[M + H]^+$: 336.1594, found 336.1586. Note: After Step 2, the desired compound was recovered together with 3-((2,5-dioxo-1-phenylcyclopent-3-en-1-yl)methyl)-*N,N*-dimethylbenzamide, that can be hydrogenated using Pd/C as catalyst to afford pure 3-((2,5-dioxo-1-phenylcyclopentyl)methyl)-*N,N*-dimethylbenzamide.

General Procedure C: Preparation of 2-trifluoromethylated-1,3-diketone substrates (illustrated for **1q**)

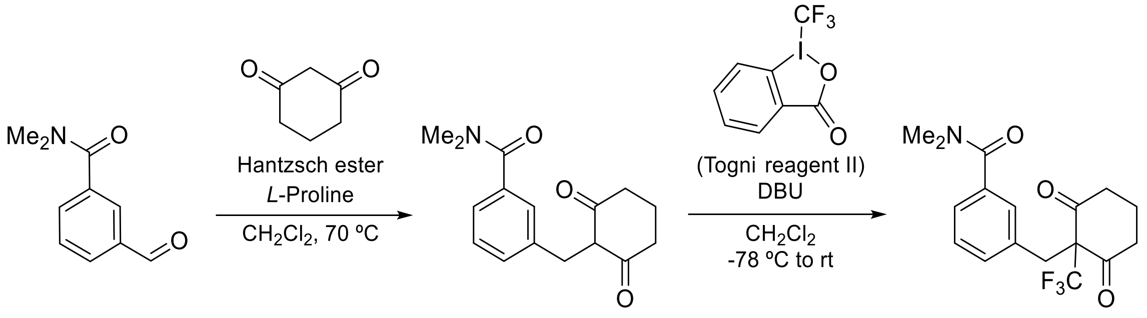

Step 1: See Step 1 of General Procedure B for the synthesis of 3-((2,6-dioxocyclohexyl)methyl)-*N,N*-dimethylbenzamide **S3**.

Step 2:[6] A solution of 3-((2,6-dioxocyclohexyl)methyl)-*N,N*-dimethylbenzamide **S3** (300 mg, 1.1 mmol, 1.0 equiv) in CH_2Cl_2 (10 mL) was added to a stirred suspension of Togni reagent II (1-trifluoromethyl-1,2-benziodoxol-3(1*H*)-one, 520 mg, 1.6 mmol, 1.5 equiv) in CH_2Cl_2 (10 mL). The mixture was stirred 30 min at rt and then cooled to -78 °C. DBU (0.32 mL, 2.2 mmol, 2.0 equiv) was added, and the reaction was stirred for 1 h at -78 °C and then at rt until complete consumption of starting material as indicated by TLC (16 h). The crude reaction mixture was concentrated and purified by flash chromatography (SiO_2-12g, 30 mL/min, 0 → 100% hexane/EtOAc) to afford 313 mg of **3-((2,6-dioxo-1-(trifluoromethyl)cyclohexyl)methyl)-*N,N*-dimethylbenzamide 1q** (83 yield) as a white solid. **^{1}H NMR** (300 MHz, $CDCl_3$) δ 7.35 – 7.25 (m, 2H), 7.17 – 7.00 (m, 2H), 3.52 (s, 2H), 3.08 (s, 3H), 2.92 (s, 3H), 2.73 – 2.57 (m, 2H), 2.46 – 2.30 (m, 2H), 1.92 (m, *J* = 14.2, 5.9 Hz, 1H), 1.37 – 1.21 (m, 1H) ppm. **^{13}C NMR** (75 MHz, $CDCl_3$) δ 202.2 (CO), 171.1 (CO), 136.8 (C), 134.3 (C), 132.2 (CH), 129.4 (CH), 128.9 (CH), 126.5 (CH), 122.42 (CF_3, q, *J* = 287.4 Hz), 71.25 (C, q, *J* = 21.2 Hz), 40.5 (CH_2), 39.6 (CH_3 amide), 35.4 (CH_3 amide), 34.7(CH_2), 15.3 (CH_2) ppm. **^{19}F NMR** (282 MHz, $CDCl_3$) δ -66.97 (s, 3F) ppm. **HRMS** [APCI]: *m/z* calculated for $C_{17}H_{19}F_3NO_3$ $[M + H]^+$: 342.1312, found 342.1309.

3-((2,7-dioxo-1-(trifluoromethyl)cycloheptyl)methyl)-*N,N*-dimethylbenzamide (1s)

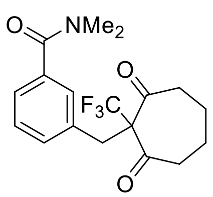

Synthesized by following General Procedure C, obtained in 82% yield as white solid. **^{1}H NMR** (300 MHz, $CDCl_3$) δ 7.36 – 7.33 (m, 2H), 7.27 – 7.23 (m, 2H), 3.51 (s, 2H), 3.13 (s, 3H), 2.97 (s, 3H), 2.55 (ddd, *J* = 12.0, 9.3, 2.4 Hz, 2H), 2.30 (ddd, *J* = 12.1, 9.3, 2.4 Hz, 2H), 1.99 – 1.87 (m, 2H), 1.87 – 1.74 (m, 2H) ppm. **^{13}C NMR** (126 MHz, $CDCl_3$) δ 203.4 (CO), 171.0 (CO), 136.8 (C), 134.0 (C), 131.7 (CH), 129.1 (CH), 128.8 (CH), 126.4 (CH), 123.35 (CF_3, d, *J* = 285.5 Hz), 73.39 (C, q, *J* = 21.3 Hz), 43.99 (CH_2, q, *J* = 2.2 Hz), 39.4 (CH_3 amide), 35.3 (CH_3 amide), 34.76 (CH_2, q, *J* = 2.4 Hz), 27.9 (CH_2) ppm. **^{19}F NMR** (471 MHz, $CDCl_3$) δ -64.8 (s, 3F) ppm. **HRMS** [APCI]: *m/z* calculated for $C_{18}H_{21}F_3NO_3$ $[M + H]^+$: 356.1468, found 356.1465.

General Procedure D: Preparation of 2-fluorinated-1,3-diketone substrates (illustrated for **1r**)

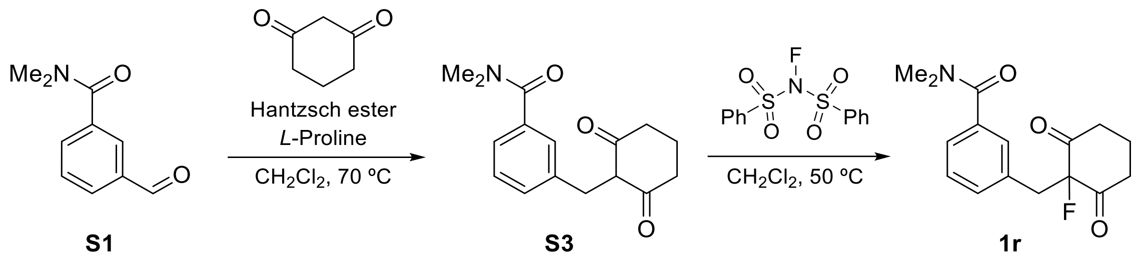

Step 1: See Step 1 of General Procedure B for the synthesis of 3-((2,6-dioxocyclohexyl)methyl)-*N,N*-dimethylbenzamide **S3**.

Step 2: *N*-fluorobenzenesulfonimide (1.0 g, 3.3 mmol, 1.5 equiv) was added to a solution of 3-((2,6-dioxocyclohexyl)methyl)-*N,N*-dimethylbenzamide **S3** (600 mg, 2.2 mmol, 1.0 equiv) in CH_2Cl_2 (8.8 mL). The resulting mixture was stirred at 50 °C for 16 h. The crude reaction mixture was concentrated under pressure and purified by flash chromatography (SiO_2-12g, 30 mL/min, 0 → 100% hexane/EtOAc, then 0 → 5% MeOH/EtOAc) to afford **3-((1-fluoro-2,6-dioxocyclohexyl)methyl)-*N,N*-dimethylbenzamide 1r** (93% yield) as a white solid. **^{1}H NMR** (300 MHz, $CDCl_3$) δ 7.37 – 7.14 (m, 4H), 3.35 (d, *J* = 24.3 Hz, 2H), 3.07 (s, 3H), 2.93 (s, 3H), 2.87 – 2.71 (m, 4H), 2.15 – 2.01 (m, 1H), 1.86 – 1.61 (m, 1H) ppm. **^{13}C NMR** (75 MHz, $CDCl_3$) δ 200.70 (CO, d, *J* = 17.5 Hz),171.17 (CO), 136.72 (C), 132.34 (C), 131.25 (CH), 128.88 (CH), 128.59 (CH), 126.71 (CH), 103.57 (CF, d, *J* = 205.3 Hz), 40.88 (CH_2, d, *J* = 22.5 Hz), 39.66 (CH_3 amide), 38.57 (CH_2), 35.47 (CH_3 amide), 18.16 (CH_2) ppm. **^{19}F NMR** (282 MHz, $CDCl_3$) δ -169.62 (s, 1F) ppm. **HRMS** [APCI]: *m/z* calculated for $C_{16}H_{19}FNO_3$ $[M + H]^+$: 292.1343, found 292.1338.

3-((1-fluoro-2,7-dioxocycloheptyl)methyl)-*N,N*-dimethylbenzamide (1t)

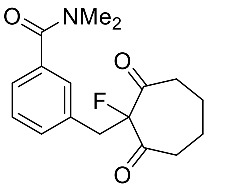

Synthesized by following General Procedure D, obtained in 21% yield as brown oil. **^{1}H NMR** (300 MHz, $CDCl_3$) δ 7.32 – 7.27 (m, 2H), 7.27 – 7.20 (m, 2H), 3.37 (d, *J* = 24.4 Hz, 2H), 3.09 (s, 3H), 2.94 (s, 3H), 2.87 – 2.73 (m, 2H), 2.45 – 2.33 (m, 2H), 2.25 – 2.08 (m, 2H), 1.81 – 1.67 (m, 2H) ppm. **^{13}C NMR** (75 MHz, $CDCl_3$) δ 204.58 (CO, d, *J* = 25.5 Hz), 171.4 (CO), 136.3 (C), 133.8 (C), 131.9 (CH), 129.3 (CH), 128.5 (CH), 126.0 (CH), 105.65 (CF, d, *J* = 194.6 Hz), 40.7 (CH_2), 39.6 (CH_3 amide), 36.59 (CH_2, d, *J* = 20.6 Hz), 35.4 (CH_3 amide), 24.65 (CH_2, d, *J* = 2.1 Hz) ppm. **^{19}F NMR** (282 MHz, $CDCl_3$) δ -161.9 (s, 1F) ppm. **HRMS** [APCI]: *m/z* calculated for $C_{17}H_{21}FNO_3$ $[M + H]^+$: 306.1500, found 306.15025.

3-((1-fluoro-2,5-dioxocyclopentyl)methyl)-*N,N*-dimethylbenzamide (1u)

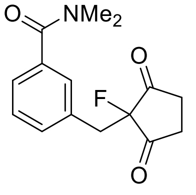

Synthesized by following General Procedure D, obtained in 49% yield as yellow solid. **^{1}H NMR** (300 MHz, $CDCl_3$) δ 7.42 – 7.25 (m, 1H), 7.21 – 7.08 (m, 1H), 3.23 (d, *J* = 17.9 Hz, 1H), 3.07 (s, 1H), 2.93 (s, 2H), 2.76 – 2.47 (m, 2H) ppm. **^{13}C NMR** (75 MHz, $CDCl_3$) δ 206.09 (CO, d, *J* = 15.1 Hz), 170.85 (CO), 137.09 (C), 131.33 (CH), 130.98 (C), 129.16 (CH), 128.64 (CH), 126.97 (CH), 93.52 (CF, d, *J* = 207.8 Hz), 39.93 (CH_2, d, *J* = 25.5 Hz), 35.46 (CH_3 amide), 34.06 (CH_3 amide), 34.02 (CH_2) ppm. **^{19}F NMR** (282 MHz, $CDCl_3$) δ -171.75 (s, 1F) ppm. **HRMS** [APCI]: *m/z* calculated for $C_{15}H_{17}FNO_3$ $[M + H]^+$: 278.1187, found 278.1185.

General Procedure E:[7] Preparation of mesylated precursor 1β

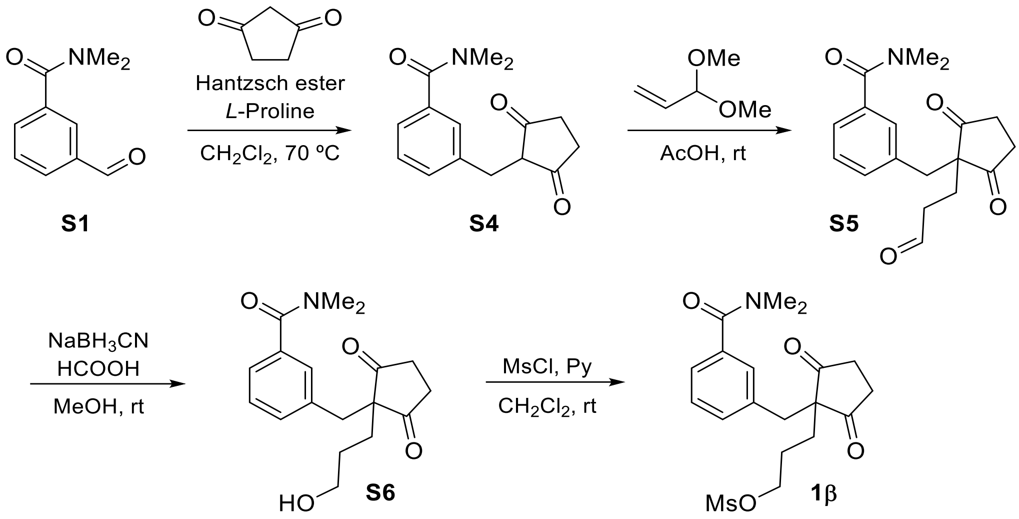

Step 1: *L*-Proline (110 mg, 0.96 mmol, 20 mol%) was added to a sealed tube containing a solution of 1,3-cyclopentadione (0.47 g, 4.8 mmol, 1.0 equiv), Hantzsch ester (1.21 g, 4.8 mmol, 1.0 equiv), and the 3-formyl-*N,N*-dimethylbenzamide **S1** (934 mg, 5.27 mmol, 1.1 equiv) in dichloromethane (12 mL). The reaction mixture was stirred at 70 °C until complete consumption of starting material as indicated by TLC. The solvent was removed under reduced pressure, and the crude residue was purified by flash chromatography (SiO_2-12g, 30 mL/min, 0 → 5% → 10% CH_2Cl_2/MeOH), to afford 660 mg of 3-((2,5-dioxocyclopentyl)methyl)-*N,N*-dimethylbenzamide **S4** (53% yield) as a pale-yellow foam.

Step 2: To a stirred solution of 3-((2,5-dioxocyclopentyl)methyl)-*N,N*-dimethylbenzamide **S4** (100 mg, 0.386 mmol, 1.0 equiv) in 10% aqueous AcOH (1 mL) was added acrolein dimethyl acetal (59 mg, 0.578 mmol, 1.5 equiv) at rt. The reaction mixture was stirred at same temperature until complete consumption of starting material as indicated by TLC (48 h). The reaction was concentrated to give 100 mg crude aldehyde **S5** (82% yield) as a colorless oil, that was used without in the next step without further purification.

Step 3: $NaBH_3CN$ (24 mg, 0.38 mmol, 1.0 equiv) was added to a suspension of aldehyde **S5** (120 mg, 0.38 mmol, 1.0 equiv) in MeOH (2 mL), and formic acid (88 mg, 1.9 mmol, 5.0 equiv) was added subsequently. The white suspension was stirred at rt until complete consumption of starting material as indicated by TLC. The reaction mixture was diluted with toluene, washed with water and saturated aqueous $NaHCO_3$ solution, dried over anhydrous Na_2SO_4, filtered and concentrated in vacuo to afford 120 mg crude alcohol **S6** (99% yield) that was used for the next step without further purification.

Step 4: The crude product **S6** was dissolved in dry CH_2Cl_2 (2.0 mL), then pyridine (179 mg, 2.7 mmol, 6.0 equiv) and MsCl (108 mg, 0.95 mmol, 2.5 equiv) were added successively under an atmosphere of argon. The reaction mixture was stirred at rt. After the starting material disappeared as indicated by TLC, the reaction mixture was diluted with diethyl ether and washed with the saturated aqueous $CuSO_4$ solution, dried over anhydrous Na_2SO_4, filtered and concentrated in vacuum. The crude residue was purified by flash chromatography (SiO_2-4g, 13 mL/min, 0 → 70% →

100% hexane/EtOAc) to give 94 mg of **3-(1-(3-(dimethylcarbamoyl)benzyl)-2,5-dioxocyclopentyl)propyl methanesulfonate 1β** (63% yield) as colorless oil. **^{1}H NMR** (300 MHz, $CDCl_3$) δ 7.33 – 7.26 (m, 2H), 7.13 – 7.02 (m, 2H), 4.14 (t, *J* = 6.1 Hz, 2H), 3.09 (s, 3H), 3.01 (s, 3H), 2.97 (s, 2H), 2.93 (s, 3H), 2.62 – 2.47 (m, 2H), 2.17 – 2.07 (m, 2H), 1.91 – 1.80 (m, 2H), 1.64 (m, 2H) ppm. **^{13}C NMR** (75 MHz, $CDCl_3$) δ 216.73 (CO), 170.97 (CO), 137.01 (C), 135.24 (C), 130.94 (CH), 128.98 (CH), 128.23 (CH), 126.41 (CH), 69.15 (C), 61.86 (CH_2), 42.14 (CH_2), 39.53 (CH_3 amide), 37.57 (CH_2), 36.55 (CH_2), 35.43 (CH_3 amide), 31.27 (CH_2), 24.47 (CH_2) ppm. **HRMS** [APCI]: *m/z* calculated for $C_{19}H_{26}NO_6S$ $[M + H]^+$: 396.1475, found 396.1475.

<u>General Procedure F</u>: Synthesis of homologated-1,3-diketone 1γ

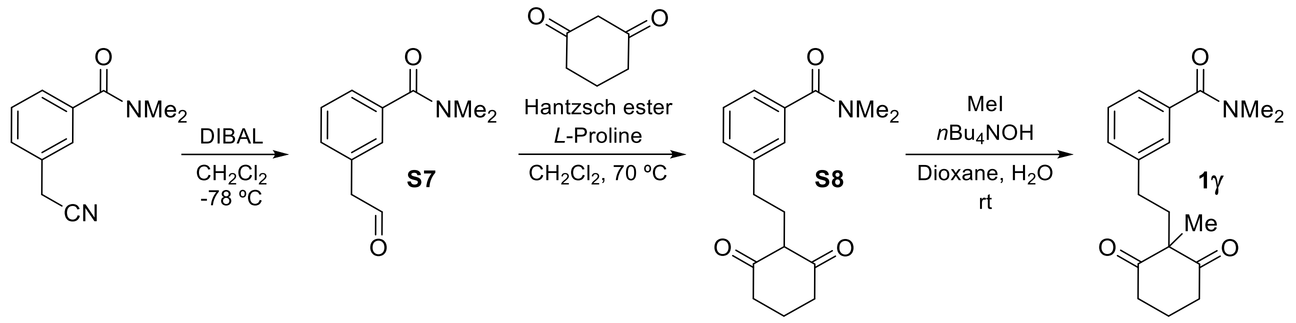

Step 1: To a solution of 3-(cyanomethyl)-*N,N*-dimethylbenzamide (0.92 g, 4.88 mmol) in anhydrous CH_2Cl_2 (32 mL) were slowly added DIBAL-H (1.0 M in THF, 5.9 ml, 5.86 mmol, 1.2 equiv) under argon atmosphere at -78 °C. The reaction mixture was stirred at same temperature until complete consumption of starting material as indicated by TLC (30 min) and quenched by dropwise addition of EtOAc. The cooling bath was removed, and saturated Rochelle Salt solution, H_2O and HCl (2.0 M in H_2O) were added. The mixture was stirred at 25 °C until two clear layers formed. The organic phase was separated, and the aqueous phase was extracted with CH_2Cl_2. The combined organic phase was dried over anhydrous Na_2SO_4 and filtered. The solvent was removed under reduced pressure, and the crude residue was purified by flash chromatography (SiO_2-12g, 30 mL/min, 0 $\rightarrow$ 100% hexane/EtOAc), to afford 290 mg of ***N,N*-dimethyl-3-(2-oxoethyl)benzamide S7** (31% yield) as a colorless oil.

Step 2: *L*-Proline (31.8 mg, 0.27 mmol, 20 mol%) was added to a sealed tube containing a solution of 1,3-cyclohexadione (155 mg, 1.38 mmol, 1.0 equiv), Hantzsch ester (350 mg, 1.38 mmol, 1.0 equiv), and the *N,N*-dimethyl-3-(2-oxoethyl)benzamide **S7** (290 mg, 1.52 mmol, 1.1 equiv) in dichloromethane (3 mL). The reaction mixture was stirred at 70 °C until complete consumption of starting material as indicated by TLC. The solvent was removed under reduced pressure, and the crude residue was purified by flash chromatography (SiO_2-12g, 30 mL/min, 0 $\rightarrow$ 100% hexane/EtOAc), to afford 380 mg of **3-(2-(2,6-dioxocyclohexyl)ethyl)-*N,N*-dimethylbenzamide S8** (96% yield) as a colorless oil.

Step 3: To a solution of 3-(2-(2,6-dioxocyclohexyl)ethyl)-*N,N*-dimethylbenzamide **S8** (0.255 g, 0.89 mmol, 1.0 equiv) in dioxane (0.9 mL) was added aqueous *n*Bu_4NOH (0.6 mL, 0.89 mmol, 1.54 M in water, 1.0 equiv). The mixture was stirred for 5 min at rt. Then MeI (0.25 g, 1.77 mmol, 2.0 equiv) was added and the reaction mixture was stirred until complete consumption of starting material as indicated by TLC (12-36 h). Upon completion, the reaction was quenched with saturated aqueous NH_4Cl solution and extracted with EtOAc. The organics were combined, dried over anhydrous Na_2SO_4, filtered and concentrated under reduced pressure. The crude residue was purified by flash chromatography (SiO_2-12g, 30 mL/min, 0 $\rightarrow$ 70% $\rightarrow$ 85% hexane/EtOAc) to give 152 mg of ***N,N*-dimethyl-3-(2-(1-methyl-2,6-dioxocyclohexyl)ethyl)benzamide 1γ** (57% yield) as a colorless oil. **^{1}H NMR** (300 MHz, $CDCl_3$) δ 7.38 – 7.29 (m, 1H), 7.28 – 7.18 (m, 3H), 3.13 (s, 3H), 3.00 (s, 3H), 2.80 – 2.67 (m, 4H), 2.54 – 2.42 (m, 2H), 2.19 – 2.08 (m, 2H), 2.07 – 1.89 (m, 2H), 1.35 (s, 3H) ppm. **^{13}C NMR** (75 MHz, $CDCl_3$) δ 209.98 (CO), 171.56 (CO), 141.56 (C), 136.66 (C), 129.52 (CH), 128.43 (CH), 126.94 (CH), 124.75 (CH), 65.38 (C), 39.53 (CH_3 amide), 38.27 (CH_2), 37.99 (CH_2), 35.28 (CH_3 amide), 31.00 (CH_2), 20.47 (CH_3), 17.64 (CH_2) ppm. **HRMS** [APCI]: *m/z* calculated for $C_{18}H_{24}NO_3$ $[M + H]^+$: 302.1751, found 302.1743.

General Procedure G: Synthesis of homologated-1,3-diketone 1δ

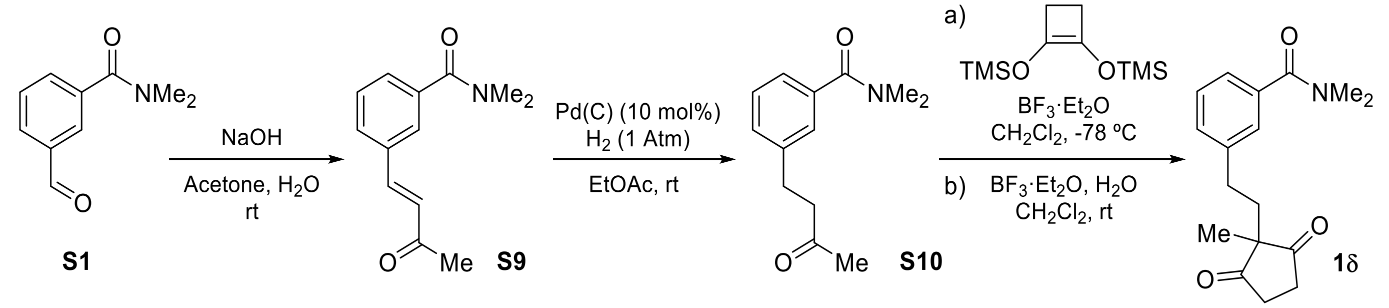

Step 1: To a suspension of 3-formyl-*N,N*-dimethylbenzamide **S1** (0.600 g, 3.38 mmol, 1.0 equiv) in a mixture of acetone and water (1:1, 3 mL), NaOH (3 mL, 40 mg, 0.85 mmol, 1% aqueous solution, 0.25 equiv) was added dropwise to this mixture at rt and the reaction mixture was stirred until complete consumption of starting material as indicated by TLC. After completion, the reaction mixture was diluted with H_2O and extracted with CH_2Cl_2. The organics were combined, dried over anhydrous Na_2SO_4, filtered and concentrated under reduced pressure. The crude residue was purified by flash chromatography (SiO_2-12g, 30 mL/min, 0 → 85% hexane/EtOAc) to give 236 mg of **(*E*)-*N,N*-dimethyl-3-(3-oxobut-1-en-1-yl)benzamide S9** (32% yield) as a pale-yellow oil.

Step 2: Pd/C (10% w/w, 115 mg, 10 mol%) was added to a solution of (*E*)-*N,N*-dimethyl-3-(3-oxobut-1-en-1-yl)benzamide **S9** (236 mg, 1.09 mmol, 1.0 equiv) in EtOAc (11 mL) under argon. The resulting black suspension was purged with H_2 for 1 minute and then stirred at rt under hydrogen atmosphere (balloon) until complete consumption of starting material as indicated by TLC. The catalyst was removed by filtration through a Celite pad, and the solids were washed with EtOAc. The filtrate was concentrated under reduced pressure, and the crude residue was purified by flash chromatography (SiO_2-12g, 30 mL/min, 0 → 80% hexane/EtOAc) to afford 170 mg of ***N,N*-dimethyl-3-(3-oxobutyl)benzamide S10** as a colorless oil (71% yield). **^{1}H NMR** (300 MHz, $CDCl_3$) δ 7.22 – 7.05 (m, 4H), 3.07 – 2.75 (m, 8H), 2.65 (t, *J* = 7.2 Hz, 2H), 2.01 (s, 3H) ppm. **^{13}C NMR** (75 MHz, $CDCl_3$) δ 207.27 (CO), 171.37 (CO), 141.15 (C), 136.39 (C), 129.31 (CH), 128.25 (CH), 126.75 (CH), 124.55 (CH), 44.56 (CH_2), 39.32 (CH_3 amide), 35.07 (CH_3 amide), 29.83 (CH_3), 29.25 (CH_2) ppm. **HRMS** [APCI]: *m/z* calculated for $C_{13}H_{18}NO_2$ $[M + H]^+$: 220.1332, found 220.1327.

Step 3:[8] $BF_3{\cdot}OEt_2$ (1.45 g, 1.27 mL, 10.3 mmol, 15 equiv) was added to a solution of *N,N*-dimethyl-3-(3-oxobutyl)benzamide **S10** (150 mg, 0.68 mmol, 1 equiv) in CH_2Cl_2 (4.5 mL) at -78 °C. The solution was stirred at this temperature for 15 min. Neat bis((trimethylsilyl)oxy)cyclobut-1-ene (0.47 g, 2.05 mmol, 3.0 equiv) was added dropwise at -78 °C, and the mixture was stirred for 1 h. Then, the mixture was warmed to rt for 24 h. The mixture was cooled to 0 °C and was treated sequentially with H_2O (0.4 mL, 21.9 mmol, 32 equiv) and $BF_3{\cdot}OEt_2$ (1.45 g, 1.27 mL, 10.3 mmol, 15 equiv). The mixture was allowed to warm to rt. After 24 h TLC revealed that rearrangement was complete. The solution was poured into saturated aqueous solution of $NaHCO_3$. The aqueous layer was extracted thoroughly with CH_2Cl_2. The organics were combined, dried over anhydrous Na_2SO_4, filtered and concentrated under reduced pressure. The crude residue was purified by flash chromatography (SiO_2-4g, 13 mL/min, 0 → 70% hexane/EtOAc) to give 29 mg of ***N,N*-dimethyl-3-(2-(1-methyl-2,5-dioxocyclopentyl)ethyl)benzamide 1δ** (15% yield) as a colorless oil. **^{1}H NMR** (300 MHz, $CDCl_3$) δ 7.32 – 7.12 (m, 4H), 3.10 (s, 3H), 2.96 (s, 3H), 2.85 – 2.64 (m, 4H), 2.54 – 2.47 (m, 2H), 2.02 – 1.91 (m,

2H), 1.16 (s, 3H) ppm. **^{13}C NMR** (75 MHz, $CDCl_3$) δ 216.23 (CO), 171.67 (CO), 141.05 (C), 136.69 (C), 129.73 (CH), 128.57 (CH), 127.16 (CH), 125.02 (CH), 56.57 (C), 39.65 (CH_3 amide), 36.61 (CH_2), 35.43 (CH_3 amide), 35.19 (CH_2), 30.80 (CH_2), 19.99 (CH_3) ppm. **HRMS** [APCI]: *m/z* calculated for $C_{17}H_{22}NO_3$ $[M + H]^+$: 288.1594, found 288.1588.

General Procedure H:[9] Synthesis of homologated-1,3-diketone 1ε

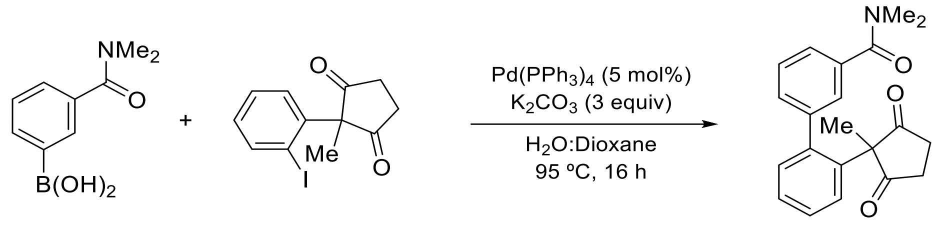

Compound 2-(2-iodophenyl)-2-methylcyclopentane-1,3-dione **S11** was prepared following reported procedure.[10]

Commercially available 3-(dimethylcarbamoyl)-phenyl boronic acid (831 mg, 4.31 mmol, 1.4 equiv), 2-(2-iodophenyl)-2-methylcyclopentane-1,3-dione **S11** (902 mg, 2.88 mmol, 1.0 equiv), $Pd(PPh_3)_4$ (166 mg, 0.14 mmol, 5 mol%) and K_2CO_3 (1.19 g, 8.61 mmol, 3.0 equiv) were suspended in a 9:1 mixture of Dioxane:H_2O (44 mL). The mixture was stirred overnight at 95 ˚C. Then, the solvents were removed under reduced pressure, water (100 mL) was added and then extracted three times with 75 mL of EtOAc. The combined organic phases were dried over anhydrous Na_2SO_4, filtered and concentrated under reduced pressure. The crude residue was purified by flash chromatography (SiO_2-12g, 24 mL/min, 0 → 100% hexane/EtOAc) to give 811 mg of ***N,N*-dimethyl-2'-(1-methyl-2,5-dioxocyclopentyl)-[1,1'-biphenyl]-3-carboxamide 1ε** (84% yield) as a brown solid. **^{1}H NMR** (300 MHz, $CDCl_3$) δ 7.66 – 7.26 (m, 5H), 7.10 (dd, *J* = 7.3, 1.5 Hz, 1H), 7.03 (dd, *J* = 6.9, 1.9 Hz, 1H), 6.99 – 6.91 (m, 1H), 3.04 (s, 3H), 2.92 (s, 3H), 2.69 – 2.46 (m, 2H), 2.23 – 2.06 (m, 1H), 1.62 (td, *J* = 17.2, 7.6 Hz, 1H), 1.45 (s, 3H) ppm. **^{13}C NMR** (75 MHz, $CDCl_3$) δ 215.4 (CO), 170.4 (CO), 141.5 (C), 140.5 (C), 136.2 (C), 136.0 (C), 132.4 (CH), 130.4 (CH), 129.1 (CH), 128.3 (CH), 128.2 (CH), 128.0 (CH), 127.6 (CH), 126.3 (CH), 61.8 (CH), 39.3 (CH_3 amide), 35.1 (CH_3 amide), 34.4 (CH_2), 34.2 (CH_2), 21.2 (CH_3) ppm. **HRMS** [APCI]: *m/z* calculated for $C_{21}H_{22}NO_3$ $[M + H]^+$: 336.1594, found 336.1594.

<u>General Procedure I</u>: Synthesis of fused-bicyclic-1,3-diketone substrates (illustrated for **4a**)

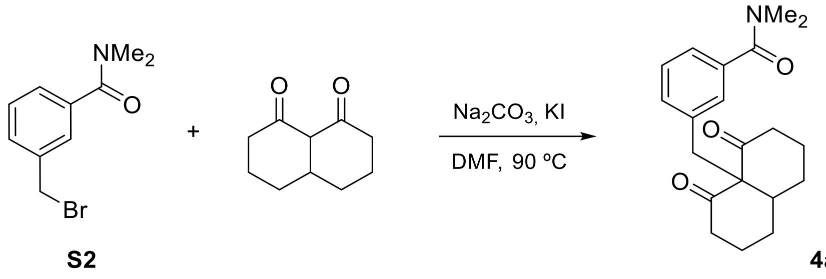

To a stirred solution of 3-(bromomethyl)-*N,N*-dimethylbenzamide **S2** (100 mg, 0.41 mmol, 1.0 equiv) in DMF (0.9 mL) was added Na_2CO_3 (88 mg, 0.83 mmol, 2.0 equiv), KI (14 mg, 0.08 mmol, 0.2 mmol) and hexahydronaphthalene-1,8(2*H*,5*H*)-dione (206 mg, 1.24 mmol, 3.0 equiv). The reaction was stirred at 90 °C until complete consumption of starting materials as indicated by TLC (16h). Upon completion, the crude was diluted with EtOAc, and water was added. After being extracted with EtOAc, the combined organic layers were washed with brine, dried over anhydrous Na_2SO_4 and evaporated under reduced pressure. The crude residue was purified by flash chromatography (SiO_2-12g, 30 mL/min, 0 $\rightarrow$ 70% hexane/EtOAc) to give 77 mg of **3-((4,5-dioxooctahydronaphthalen-4a(2*H*)-yl)methyl)-*N,N*-dimethylbenzamide 4a** (57% yield) as a white solid. **^{1}H NMR** (300 MHz, $CDCl_3$) δ 7.32 – 7.25 (m, 4H), 3.36 (s, 2H), 3.08 (s, 3H), 2.95 (s, 3H), 2.61 – 2.49 (m, 2H), 2.44 – 2.32 (m, 2H), 2.23 – 2.14 (m, 1H), 2.07 – 1.86 (m, 4H), 1.81 – 1.62 (m, 4H) ppm. **^{13}C NMR** (75 MHz, $CDCl_3$) δ 210.2 (CO), 137.1 (C), 136.3 (C), 132.0 (CH), 129.3 (CH), 128.5 (CH), 125.7 (CH), 69.8 (C), 43.0 (CH), 39.8 (CH_2), 36.4 (CH_2), 27.0 (CH_2), 23.3 (CH_2) ppm. <u>Note</u>: Amide ^{13}C signals do not appear due to rotamerism. **HRMS** [APCI]: *m/z* calculated for $C_{20}H_{26}NO_3$ $[M + H]^+$: 328.1907, found 328.1904.

3-((3,4-dioxohexahydropentalen-3a(1*H*)-yl)methyl)-*N,N*-dimethylbenzamide (4b)

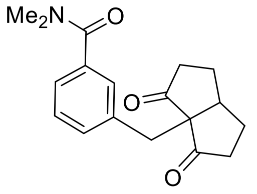

Prepared following <u>General Procedure I</u>, obtained in 71% yield as a pale-yellow solid. **^{1}H NMR** (300 MHz, $CDCl_3$) δ 7.30 – 7.26 (m, 2H), 7.14 – 7.09 (m, 2H), 3.06 (s, 3H), 3.02 (s, 3H), 2.92 (s, 3H), 2.84 (p, *J* = 6.4 Hz, 1H), 2.33 (ddd, *J* = 18.9, 8.7, 6.5 Hz, 2H), 2.10 (ddd, *J* = 18.8, 9.1, 7.0 Hz, 2H), 1.99 – 1.83 (m, 2H), 1.72 – 1.57 (m, 2H) ppm. **^{13}C NMR** (75 MHz, $CDCl_3$) δ 211.5 (CO), 170.9 (CO), 136.6 (C), 136.4 (C), 130.7 (CH), 128.6 (CH), 128.0 (CH), 125.7 (CH), 70.4 (C), 42.2 (CH), 39.3 (CH_3 amide), 38.9 (CH_2), 37.1 (CH_2), 35.1 (CH_3 amide), 24.4 (CH_2) ppm. **HRMS** [APCI]: *m/z* calculated for $C_{18}H_{22}NO_3$ $[M + H]^+$: 300.1594, found 300.1591.

General Procedure J: **Synthesis of indole-1,3-diketone substrates** (illustrated for **7a**)

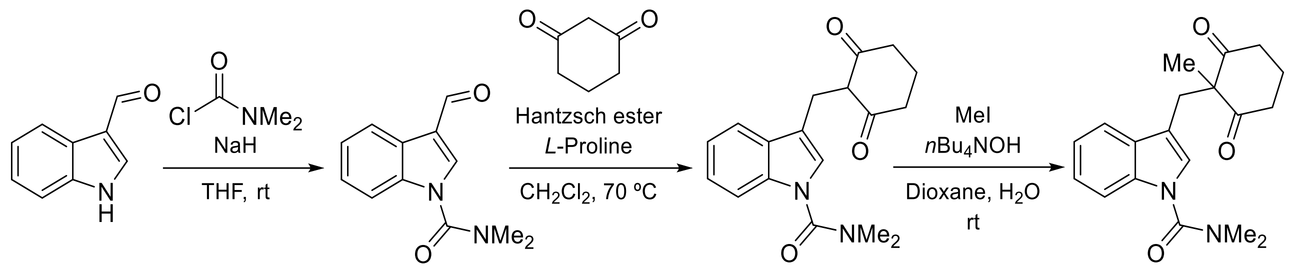

Step 1: Indole-3-carboxaldehyde (5 g, 34.4 mmol, 1.0 equiv) was dissolved in anhydrous THF and cooled to 0 °C. Then NaH (60%, 2.07 g, 51.67 mmol, 1.5 equiv) was added and stir for 15 min. After this *N,N*-dimethylcarbamoyl chloride (5.55 g, 51.67 mmol, 1.5 equiv) was added to the reaction mixture and stirred at rt. Once the starting material was fully converted as indicated by TLC, the reaction mixture was quenched at 0 °C with saturated aqueous NH_4Cl solution. Then it was extracted with EtOAc, washed with water, brine and dried over anhydrous Na_2SO_4, filtered and concentrated under reduced pressure to afford 7.4 g of **3-formyl-*N,N*-dimethylbenzamide S12** (99% yield) as a pale-orange solid that was used without in the next step without further purification.

Step 2: *L*-Proline (72 mg, 0.62 mmol, 20 mol%) was added to a sealed tube containing a solution of 1,3-cyclohexanedione (0.35 g, 3.12 mmol, 1.0 equiv), Hantzsch ester (0.79 g, 3.12 mmol, 1.0 equiv), and the 3-formyl-*N,N*-dimethyl-1*H*-indole-1-carboxamide **S12** (810 mg, 3.74 mmol, 1.2 equiv) in dichloromethane (6.2 mL). The reaction mixture was stirred at 70 °C until complete consumption of starting material as indicated by TLC. The solvent was removed under reduced pressure, and the crude residue was purified by flash chromatography (SiO_2-12g, 30 mL/min, 0 $\rightarrow$ 100% hexane/EtOAc), to afford 515 mg of **3-((2,6-dioxocyclohexyl)methyl)-*N,N*-dimethyl-1*H*-indole-1-carboxamide S13** (60% yield) as a white solid.

Step 3: To a solution of 3-((2,6-dioxocyclohexyl)methyl)-*N,N*-dimethyl-1*H*-indole-1-carboxamide **S13** (0.51 g, 1.65 mmol, 1.0 equiv) in dioxane (1.6 mL) was added aqueous nBu$_4$NOH (1.1 mL, 1.65 mmol, 1.54 M in water, 1.0 equiv). The mixture was stirred for 5 min at rt. Then MeI (0.47 g, 3.3 mmol, 2.0 equiv) was added and the reaction mixture was stirred until complete consumption of starting material as indicated by TLC (12-36 h). Upon completion, the reaction was quenched with NH_4Cl (sat) and extracted with EtOAc. The organics were combined, dried over anhydrous Na_2SO_4, filtered and concentrated under reduced pressure. The crude residue was purified by flash chromatography (SiO_2-12g, 30 mL/min, 0 $\rightarrow$ 50% $\rightarrow$ 70% hexane/EtOAc) to give 260 mg of ***N,N*-dimethyl-3-((1-methyl-2,6-dioxocyclohexyl)methyl)-1*H*-indole-1-carboxamide 7a** (48% yield) as pale-yellow solid. **^{1}H NMR** (300 MHz, $CDCl_3$) δ 7.60 – 7.53 (m, 1H), 7.52 – 7.45 (m, 1H), 7.24 – 7.08 (m, 2H), 6.98 – 6.90 (m, 1H), 3.23 – 3.17 (m, 2H), 3.02 – 2.95 (m, 6H), 2.57 – 2.41 (m, 2H), 2.33 – 2.16 (m, 2H), 1.79 – 1.63 (m, 1H), 1.59 – 1.43 (m, 1H), 1.34 – 1.27 (m, 3H) ppm. **^{13}C NMR** (75 MHz, $CDCl_3$) δ 211.40 (CO), 154.73 (CO), 135.37 (C), 128.98 (C), 125.45 (CH), 123.69 (CH), 121.71 (CH), 119.65 (CH), 114.29 (C), 113.35 (CH), 64.95 (C), 38.94 (CH_2), 38.31 (CH_3 amide), 32.22 (CH_2), 22.51 (CH_3), 16.71 (CH_2) ppm. **HRMS** [APCI]: *m/z* calculated for $C_{19}H_{23}N_2O_3$ $[M + H]^+$: 327.1703, found 327.1699.

2-methyl-2-((1-(piperidine-1-carbonyl)-1*H*-indol-3-yl)methyl)cyclohexane-1,3-dione (7a^{I})

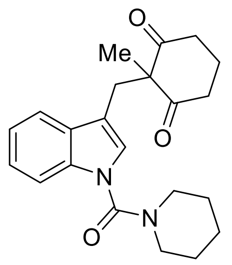

Prepared following General Procedure J, from piperidine-1-carbonyl chloride instead *N,N*-dimethylcarbamoyl chloride, obtained in 55 % yield as white solid. **^{1}H NMR** (300 MHz, $CDCl_3$) δ 7.60 (ddd, *J* = 8.2, 1.3, 0.8 Hz, 1H), 7.50 (ddd, *J* = 7.7, 1.5, 0.8 Hz, 1H), 7.25 – 7.11 (m, 2H), 6.93 (s, 1H), 3.47 – 3.40 (m, 4H), 3.21 (s, 2H), 2.59 – 2.44 (m, 2H), 2.36 – 2.19 (m, 2H), 1.87 – 1.44 (m, 8H), 1.32 (s, 3H) ppm. **^{13}C NMR** (75 MHz, $CDCl_3$) δ 211.41 (CO), 153.95 (CO), 135.36 (C), 129.13 (C), 125.58 (CH), 123.68 (CH), 121.70 (CH), 119.6 (CH)5, 114.29 (C), 113.16 (CH), 65.05 (C), 47.59 (CH_2), 38.96 (CH_2), 32.25 (CH_2) (CH_2), 25.85 (CH_2), 24.35 (CH_2), 22.51 (CH_3), 16.77 (CH_2) ppm. **HRMS** [APCI]: *m/z* calculated for $C_{22}H_{27}N_2O_3$ $[M + H]^+$: 367.2016, found 367.2020.

***N,N*-dimethyl-3-((4-methyl-3,5-dioxotetrahydro-2*H*-pyran-4-yl)methyl)-1*H*-indole-1-carboxamide (7b)**

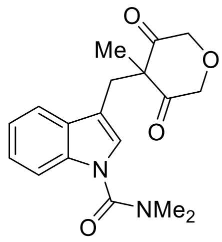

Prepared following General Procedure J, obtained in 69% yield as white solid. **^{1}H NMR** (300 MHz, $CDCl_3$) δ 7.65 – 7.53 (m, 2H), 7.32 – 7.13 (m, 2H), 7.04 (s, 1H), 4.21 (d, *J* = 17.9 Hz, 2H), 4.10 (d, *J* = 17.9 Hz, 2H), 3.37 (s, 2H), 3.05 (s, 6H), 1.42 (s, 3H) ppm. **^{13}C NMR** (75 MHz, $CDCl_3$) δ 208.11 (CO), 154.93 (CO), 135.54 (C), 129.36 (C), 125.71 (CH), 123.96 (CH), 121.91 (CH), 119.43 (CH), 113.59 (CH), 113.36 (C), 73.72 (CH_2), 64.65 (C), 38.53 (CH_3 amide), 30.58 (CH_2), 19.43 (CH_3) ppm. **HRMS** [APCI]: *m/z* calculated for $C_{18}H_{21}N_2O_4$ $[M + H]^+$: 329.1496, found 329.1493.

3-((1-benzyl-2,6-dioxocyclohexyl)methyl)-*N,N*-dimethyl-1*H*-indole-1-carboxamide (7c)

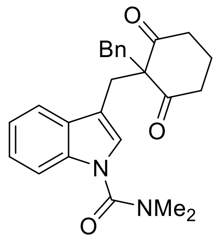

Prepared following General Procedure J, from benzyl bromide instead MeI, obtained in 65% yield as white solid. **^{1}H NMR** (300 MHz, $CDCl_3$) δ 7.62 – 7.55 (m, 1H), 7.54 – 7.43 (m, 1H), 7.27 – 7.13 (m, 5H), 7.07 – 6.98 (m, 2H), 6.96 (s, 1H), 3.38 (s, 2H), 3.29 (s, 2H), 3.04 (s, 6H), 1.91 – 1.76 (m, 4H), 1.05 – 0.70 (m, 2H) ppm. **^{13}C NMR** (75 MHz, $CDCl_3$) δ 213.52 (CO), 154.85 (CO), 136.56 (C), 135.69 (C), 130.01 (CH), 128.70 (CH), 128.45 (C), 127.20 (CH), 125.89 (CH), 124.03 (CH), 122.09 (CH), 120.12 (CH), 114.13 (C), 113.50 (CH), 70.14 (C), 45.19 (CH_2), 41.49 (CH_2), 38.49 (CH_3 amide), 34.59 (CH_2), 14.97 (CH_2) ppm. **HRMS** [APCI]: *m/z* calculated for $C_{25}H_{27}N_2O_3$ $[M + H]^+$: 403.2016, found 403.2010.

***N,N*-dimethyl-3-((1,4,4-trimethyl-2,6-dioxocyclohexyl)methyl)-1*H*-indole-1-carboxamide (7d)**

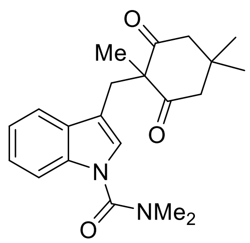

Prepared following General Procedure J, obtained in 71% yield as yellow oil. **^{1}H NMR** (300 MHz, $CDCl_3$) δ 7.62 – 7.49 (m, 2H), 7.29 – 7.09 (m, 2H), 7.01 (s, 1H), 3.18 (s, 2H), 3.01 (s, 6H), 2.49 (d, *J* = 15.0 Hz, 2H), 2.35 (d, *J* = 15.1 Hz, 2H), 1.31 (s, 3H), 0.87 (s, 3H), 0.72 (s, 3H) ppm. **^{13}C NMR** (75 MHz, $CDCl_3$) δ 210.81 (CO), 154.80 (CO), 135.33 (C), 129.45 (C), 125.89 (CH), 123.70 (CH), 121.73 (CH), 119.65 (CH), 113.85 (C), 113.43 (CH), 64.63 (C), 52.37 (CH_2), 38.39 (CH_3 amide), 32.17 (CH_2), 30.21 (C), 28.69 (CH_3), 28.54 (CH_3), 21.52 (CH_3) ppm. **HRMS** [APCI]: *m/z* calculated for $C_{21}H_{27}N_2O_3$ $[M + H]^+$: 355.2016, found 355.2020.

_N,N_-dimethyl-3-((1-methyl-2,6-dioxocyclohexyl)methyl)-1_H_-pyrrole-1-carboxamide (7e)

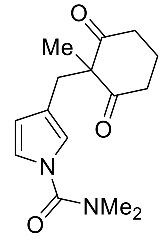

Prepared following General Procedure J, obtained in 52% yield as colorless oil. **^{1}H NMR** (300 MHz, $CDCl_3$) δ 6.87 (t, *J* = 2.7 Hz, 1H), 6.66 (s, 1H), 5.89 (dd, *J* = 3.1, 1.7 Hz, 1H), 3.01 (s, 6H), 2.93 (s, 2H), 2.63 – 2.47 (m, 2H), 2.47 – 2.33 (m, 2H), 1.90 – 1.55 (m, 2H), 1.24 (s, 3H) ppm. **^{13}C NMR** (75 MHz, $CDCl_3$) δ 211.45 (CO), 154.55 (CO), 120.80 (C), 120.75 (CH), 119.63 (CH), 112.23 (CH), 65.41 (C), 39.07 (CH_2), 38.66 (CH_3 amide), 34.68 (CH_2), 22.25 (CH_3), 16.93 (CH_2) ppm. **HRMS** [APCI]: *m/z* calculated for $C_{15}H_{21}N_2O_3$ $[M + H]^+$: 277.1547, found 277.1545.

General Procedure K: Synthesis of alkenyl-1,3-diketone substrate 9a

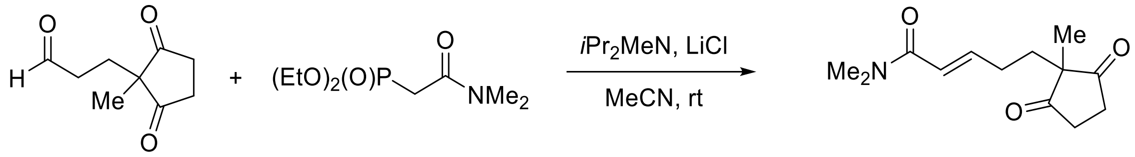

Aldehyde **S14** was prepared following reported procedure.[11] LiCl (151.2 mg, 3.6 mmol) in a 50 mL flask was dried by a heat gun under reduced pressure. Then, diethyl (2-(dimethylamino)-2-oxoethyl)phosphonate (437 mg, 2.0 mmol) in MeCN (21 mL) and *N,N*-diisopropylethylamine (621 µL, 3.6 mmol) were added at 0 °C. The mixture was stirred at 0 °C for 20 min, and then aldehyde **S14** (300 mg, 1.8 mmol) was added. The mixture was stirred at room temperature for 24 h, and water (50 mL) was added. The mixture was extracted with ethyl acetate (50 mL × 3) and washed with brine (50 mL). The combined extracts were dried over Na_2SO_4. The organic phase was concentrated under reduced pressure. The crude residue was purified by flash chromatography (SiO_2-12g, 30 mL/min, 0 → 100% hexane/EtOAc) to give 123 mg of **(*E*)-*N,N*-dimethyl-5-(1-methyl-2,5-dioxocyclopentyl)pent-2-enamide 9a** (29% yield) as colorless oil. **^{1}H NMR** (500 MHz, $CDCl_3$) δ 6.67 (dt, *J* = 15.1, 6.9 Hz, 1H), 6.19 (d, *J* = 15.1 Hz, 1H), 3.00 (s, 6H), 2.83 – 2.68 (m, 4H), 2.12 – 2.04 (m, 2H), 1.81 – 1.74 (m, 2H), 1.13 (s, 3H) ppm. **^{13}C NMR** (126 MHz, $CDCl_3$) δ 216.2 (CO), 166.5 (CO), 143.7 (CH), 121.6 (CH), 56.3 (C), 35.2 (CH_2), 33.5 (CH_2), 27.6 (CH_2), 19.7 (CH_3) ppm. **HRMS** [APCI]: *m/z* calculated for $C_{13}H_{20}NO_3$ $[M + H]^+$: 238.1438, found 238.1439.

4. Ir-catalyzed intramolecular hydrocarbonation (General Procedure L, illustrated for **3b**)

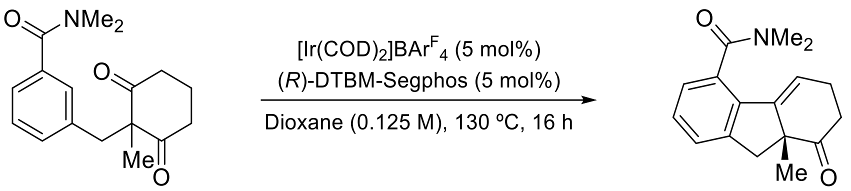

1b **3b**

Freshly prepared[2] [Ir(COD)$_2$]BAr$^F{}_4$ (6.35 mg, 0.005 mmol), (*R*)-DTBM-Segphos (5.9 mg, 0.005 mmol), prochiral diketone **1b** (28.7 mg, 0.1 mmol) and dioxane (0.8 mL, 0.125 M) were sequentially added to a sealed tube under argon atmosphere and the reaction mixture was stirred at 130 ˚C until complete consumption of starting material as indicated by TLC (typically 16 h). The mixture was concentrated, and the crude residue was purified by flash chromatography (SiO$_2$-4g, 13 mL/min, 0 → 100% hexane/CH$_2$Cl$_2$ then 0 → 70% hexane/EtOAc) to give 22 mg of **(*R*)-*N,N*,9a-trimethyl-1-oxo-2,3,9,9a-tetrahydro-1*H*-fluorene-5-carboxamide 3b** (84% yield) as a yellow oil. **^{1}H NMR** (500 MHz, CDCl$_3$) δ 7.26 – 7.14 (m, 2H), 7.08 – 6.95 (m, 1H), 6.05 (d x2, *J* = 88.4, 6.8, 2.8 Hz, 1H), 3.29 (d x2, *J* = 41.4, 16.6 Hz, 1H), 3.11 (s x2, 3H), 2.78 (d, *J* = 3.0 Hz, 3H), 2.75 – 2.60 (m, 3H), 2.57 – 2.48 (m, 1H), 2.32 – 2.23 (m, 1H), 1.25 (s x2, 3H) ppm. **^{13}C NMR** (126 MHz, CDCl$_3$) δ 215.15 (CO), 170.72 (CO), 145.43 (C), 145.27 (C), 143.49 (C), 142.79 (C), 133.37 (C), 133.25 (C), 131.80 (C), 131.53 (C), 128.82 (CH), 128.51 (CH), 126.42 (CH), 126.34 (CH), 124.88 (CH), 124.54 (CH), 119.64 (CH), 118.97 (CH), 55.28 (C), 54.99 (C), 40.17 (CH$_2$), 40.00 (CH$_2$), 38.49 (CH$_3$ amide), 38.28 (CH$_3$ amide), 35.62 (CH$_2$), 35.27 (CH$_2$), 34.90 (CH$_3$ amide), 34.63 (CH$_3$ amide), 24.58 (CH$_3$), 24.08 (CH$_3$), 23.88 (CH$_2$) ppm. Note: most carbon signals are duplicated due to rotamerism. **HRMS** [APCI]: *m/z* calculated for $C_{17}H_{20}NO_2$ [M + H]$^+$: 270.1489, found 270.1492.

Enantioselectivity was determined by chiral HPLC analysis on a Chiralpack IG3 column at rt (Hexane:*i*PrOH = 90:10, 1 mL/min).

A: Racemic sample prepared using [Ir(COD)$_2$]BAr$^F{}_4$ / *rac*-Binap (10 mol%) at 130 ˚C (Table S3, entry 1, 96% yield).

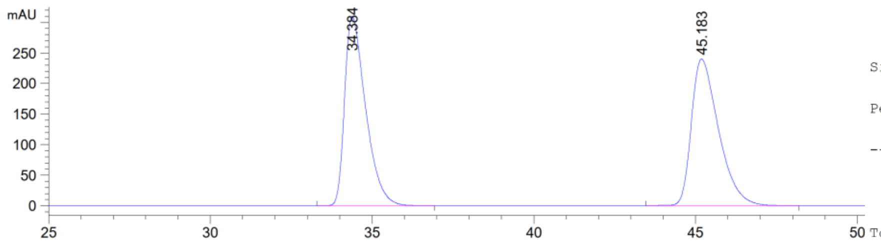

Signal 2: DAD1 B, Sig=254,4 Ref=off

Peak #	RetTime [min]	Type	Width [min]	Area [mAU*s]	Height [mAU]	Area %
1	34.384	BB	0.6841	1.39710e4	309.82864	49.9253
2	45.183	BB	0.8859	1.40129e4	239.74489	50.0747
Totals :				2.79839e4	549.57353	

B: Asymmetric sample prepared using [Ir(COD)$_2$]BAr$^F{}_4$ / *(R)*-Segphos (10 mol%) at 130 ˚C (Table S3, entry 2, 96% yield, 85:15 er).

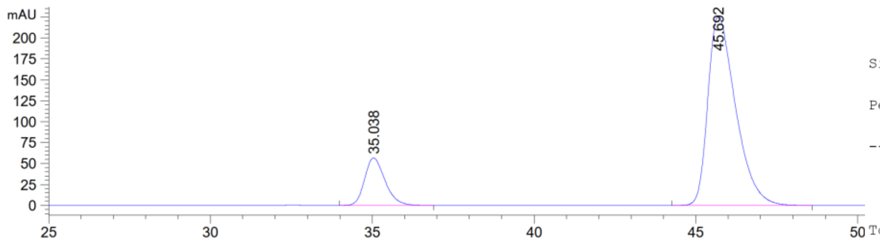

Signal 2: DAD1 B, Sig=254,4 Ref=off

Peak #	RetTime [min]	Type	Width [min]	Area [mAU*s]	Height [mAU]	Area %
1	35.038	BB	0.6669	2477.19238	56.56292	15.5466
2	45.692	BB	0.9019	1.34568e4	226.25812	84.4534
Totals :				1.59340e4	282.82104	

C: Asymmetric sample prepared using $[Ir(COD)_2]BAr^F_4$ / *(R)*-DTBM-Garphos (10 mol%) at 130 ˚C (Table S3, entry 3, 34% yield, 84:16 er).

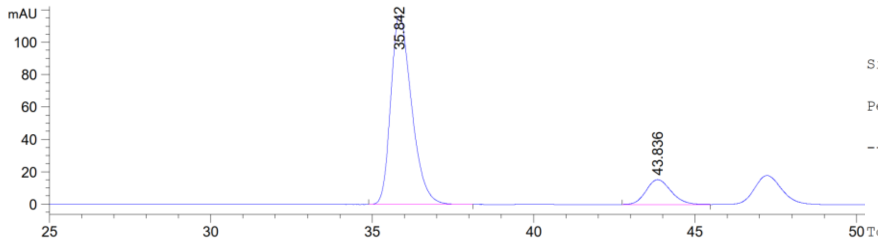

Signal 2: DAD1 B, Sig=254,4 Ref=off

Peak #	RetTime [min]	Type	Width [min]	Area [mAU*s]	Height [mAU]	Area %
1	35.842	BB	0.6892	5233.57715	116.70697	86.4011
2	43.836	BB	0.7880	823.72748	15.35051	13.5989
Totals :				6057.30463	132.05748	

D: Asymmetric sample prepared using $[Ir(COD)_2]BAr^F_4$ / *(R)*-DTBM-Binap (10 mol%) at 130 ˚C (Table S3, entry 4, 75% yield, 85:15 er).

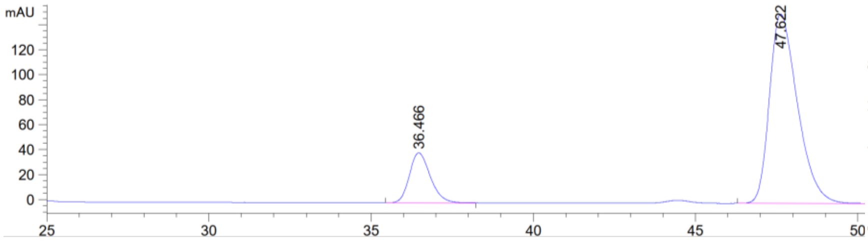

Signal 2: DAD1 B, Sig=254,4 Ref=off

Peak #	RetTime [min]	Type	Width [min]	Area [mAU*s]	Height [mAU]	Area %
1	36.466	BB	0.6826	1789.56519	39.95393	16.4475
2	47.622	BB	0.9063	9090.91504	151.44569	83.5525
Totals :				1.08805e4	191.39962	

E: Asymmetric sample prepared using $[Ir(COD)_2]BAr^F_4$ / *(S)*-DTBM-Synphos (10 mol%) at 130 ˚C (Table S3, entry 5, 27% yield, 85:15er).

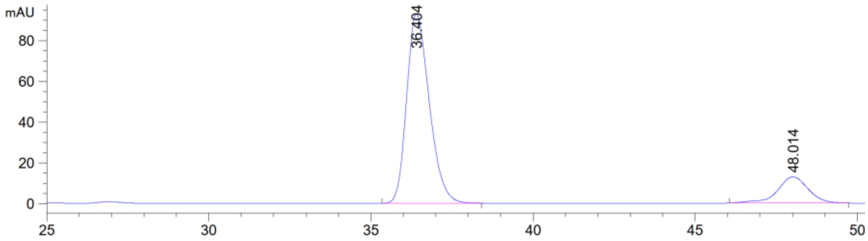

Signal 2: DAD1 B, Sig=254,4 Ref=off

Peak #	RetTime [min]	Type	Width [min]	Area [mAU*s]	Height [mAU]	Area %
1	36.404	BB	0.7567	4513.62988	92.91423	84.2187
2	48.014	BB	0.8087	845.78650	12.83587	15.7813
Totals :				5359.41638	105.75010	

F: Asymmetric sample prepared using $[Ir(COD)_2]BAr^F_4$ / *(R)*-DTMS-Segphos (10 mol%) at 130 ˚C (Table S3, entry 6, 98% yield, 86:14 er).

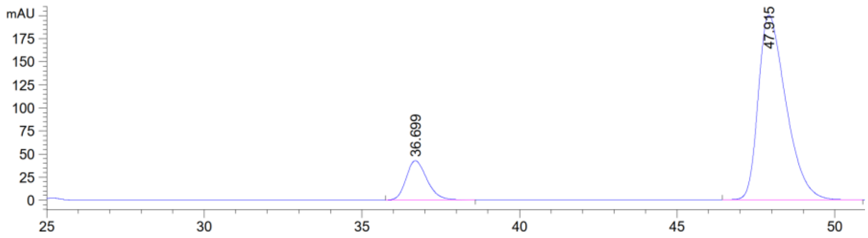

Signal 2: DAD1 B, Sig=254,4 Ref=off

Peak #	RetTime [min]	Type	Width [min]	Area [mAU*s]	Height [mAU]	Area %
1	36.699	BB	0.7039	1946.85693	42.53896	13.7417
2	47.915	BB	0.9309	1.22206e4	199.99457	86.2583
Totals :				1.41675e4	242.53353	

G: Asymmetric sample prepared using $[Ir(COD)_2]BAr^F_4$ / *(R)*-DTMS-Garphos (10 mol%) at 130 ˚C (Table S3, entry 7, 88% yield, 85:15 er).

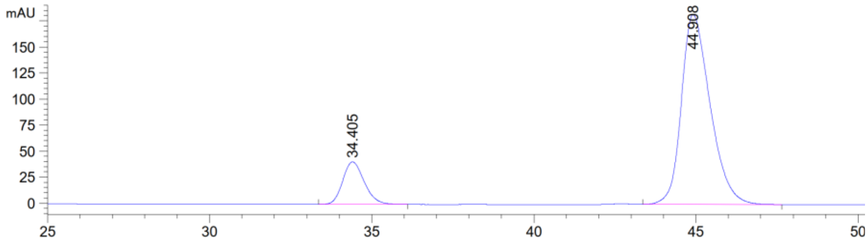

Signal 2: DAD1 B, Sig=254,4 Ref=off

Peak #	RetTime [min]	Type	Width [min]	Area [mAU*s]	Height [mAU]	Area %
1	34.405	BB	0.7096	1883.53687	40.87844	14.3572
2	44.908	BB	0.9351	1.12356e4	182.28778	85.6428
Totals :				1.31191e4	223.16622	

H: Asymmetric sample prepared using [Ir(COD)$_2$]BAr$^F{}_4$ / *(R)*-Sunphos (10 mol%) at 130 ˚C (Table S3, entry 8, 91% yield, 71:29 er).

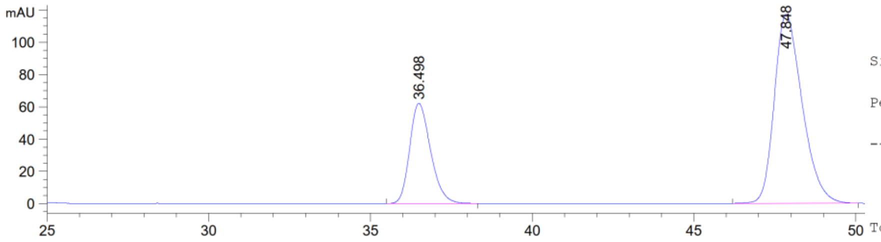

Signal 2: DAD1 B, Sig=254,4 Ref=off

Peak #	RetTime [min]	Type	Width [min]	Area [mAU*s]	Height [mAU]	Area %
1	36.498	BB	0.6881	2791.11890	62.13311	28.4429
2	47.848	BB	0.9097	7021.92773	117.08994	71.5571
Totals :				9813.04663	179.22304	

I: Asymmetric sample prepared using [Ir(COD)$_2$]BAr$^F{}_4$ / *(R)*-DTBM-Segphos (10 mol%) at 130 ˚C (Table S3, entry 9, 94% yield, 90:10 er).

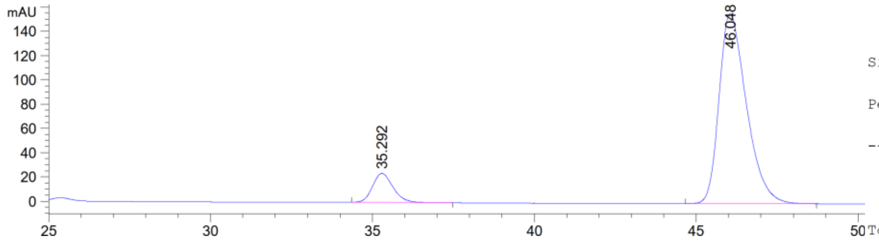

Signal 2: DAD1 B, Sig=254,4 Ref=off

Peak #	RetTime [min]	Type	Width [min]	Area [mAU*s]	Height [mAU]	Area %
1	35.292	BB	0.6694	1044.03687	23.81981	10.1981
2	46.048	BB	0.9013	9193.52148	156.50128	89.8019
Totals :				1.02376e4	180.32109	

J: Asymmetric sample prepared using [Ir(COD)$_2$]BAr$^F{}_4$ / *(R)*-DTBM-Segphos (10 mol%) in the presence of 1.25 equiv of Et_3SiH at 130 ˚C for 16 h (Table S4, entry 6, 70% yield, 91:9 er).

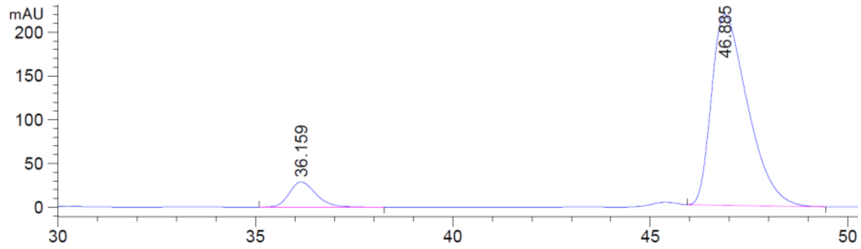

Signal 2: DAD1 B, Sig=254,4 Ref=off

Peak #	RetTime [min]	Type	Width [min]	Area [mAU*s]	Height [mAU]	Area %
1	36.159	BB	0.7138	1340.95496	28.76830	8.7968
2	46.885	BB	0.9767	1.39028e4	218.38797	91.2032
Totals :				1.52437e4	247.15627	

K: Asymmetric sample prepared using [Ir(COD)$_2$]BAr$^F{}_4$ / *(R)*-DTBM-Segphos (10 mol%) in the presence of 0.5 equiv of Et_3SiH at 130 ˚C for 5 h (Table S4, entry 8, 42% yield, 92:8 er).

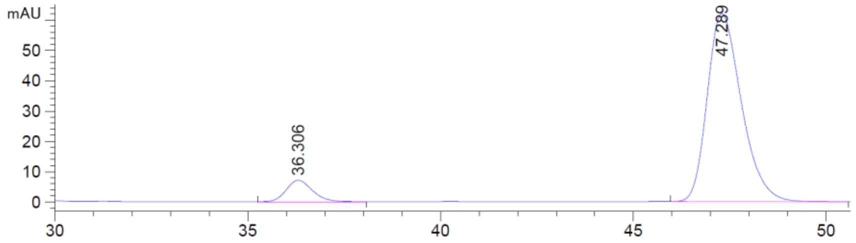

Signal 2: DAD1 B, Sig=254,4 Ref=off

Peak #	RetTime [min]	Type	Width [min]	Area [mAU*s]	Height [mAU]	Area %
1	36.306	BB	0.7015	334.72919	7.08074	8.0628
2	47.289	BB	0.9368	3816.79248	61.94770	91.9372
Totals :				4151.52167	69.02844	

L: Asymmetric sample prepared using [Ir(COD)$_2$]BAr$^F{}_4$ / *(R)*-DTBM-Segphos (10 mol%) in the presence of 1.25 equiv of HBPin at 130 ˚C for 3 h (Table S4, entry 17, 47% yield, 90:10 er).

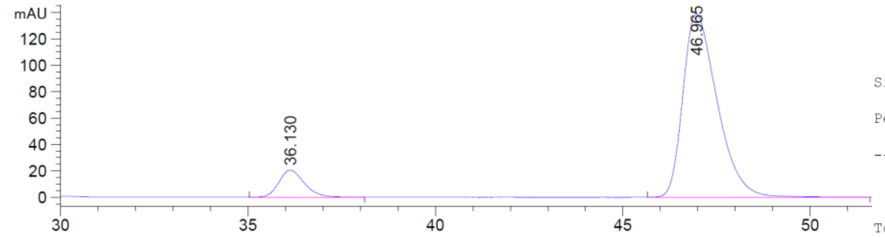

Signal 2: DAD1 B, Sig=254,4 Ref=off

Peak #	RetTime [min]	Type	Width [min]	Area [mAU*s]	Height [mAU]	Area %
1	36.130	BB	0.7110	960.11011	20.47669	9.8356
2	46.965	BB	0.9727	8801.47852	138.63393	90.1644
Totals :				9761.58862	159.11062	

(4a*R*,9a*R*)-4a-hydroxy-*N*,*N*,9a-trimethyl-1-oxo-2,3,4,4a,9,9a-hexahydro-1*H*-fluorene-5-carboxamide (2b)

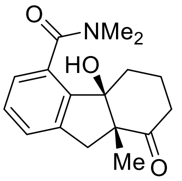

Prepared following General Procedure L from prochiral diketone **1b** in the presence of Et_3SiH (or other additives when noted). Reaction time: 3 hours. Obtained as yellow oil. **^{1}H NMR** (500 MHz, $CDCl_3$) δ 7.28 – 7.24 (m, 1H), 7.22 (t, *J* = 7.5 Hz, 1H), 7.06 (d, *J* = 7.4 Hz, 1H), 3.82 (br s, 1H), 3.53 (d, *J* = 15.6 Hz, 1H), 3.15 (s, 3H), 3.03 (s, 3H), 2.72 (d, *J* = 15.6 Hz, 1H), 2.57 – 2.47 (m, 1H), 2.39 – 2.28 (m, 1H), 2.21 – 2.10 (m, 2H), 2.05 – 1.95 (m, 1H), 1.49 – 1.39 (m, 1H), 1.30 (s, 3H) ppm. **^{13}C NMR** (126 MHz, $CDCl_3$) δ 214.0 (CO), 171.6 (CO), 144.1 (C), 143.7 (C), 131.6 (C), 127.9 (CH), 126.7 (CH), 125.2 (CH), 85.3 (COH), 61.3 (C), 39.9 (CH_2), 39.8 (CH_3 amide), 37.9 (CH_2), 35.3 (CH_3 amide), 34.0 (CH_2), 20.0 (CH_2), 18.0 (CH_3) ppm. **HRMS** [APCI]: *m/z* calculated for $C_{17}H_{22}NO_3$ $[M + H]^+$: 288.1594, found 288.1597.

Enantioselectivity was determined by chiral HPLC analysis on a Chiralpack IE3 column at rt (Hexane:*i*PrOH = 90:10, 1 mL/min).

A: Racemic sample prepared using $[Ir(COD)_2]BAr^F{}_4$ / *rac*-DTBM-Segphos (10 mol%) at 130 ˚C in the presence of 1.25 equiv of Et_3SiH for 3 h.

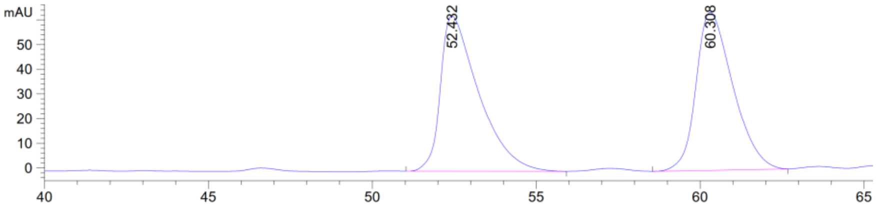

Signal 1: DAD1 A, Sig=220,4 Ref=off

Peak #	RetTime [min]	Type	Width [min]	Area [mAU*s]	Height [mAU]	Area %
1	52.432	BB	1.1718	5124.37744	62.94258	50.5360
2	60.308	BB	1.1525	5015.66846	63.98815	49.4640
Totals :				1.01400e4	126.93073	

B: Asymmetric sample prepared using $[Ir(COD)_2]BAr^F{}_4$ / *(R)*-DTBM-Segphos (10 mol%) at 130 ˚C in the presence of 1.25 equiv of Et_3SiH for 16 h. (Table S4, entry 6, 30% yield, 91:9 er).

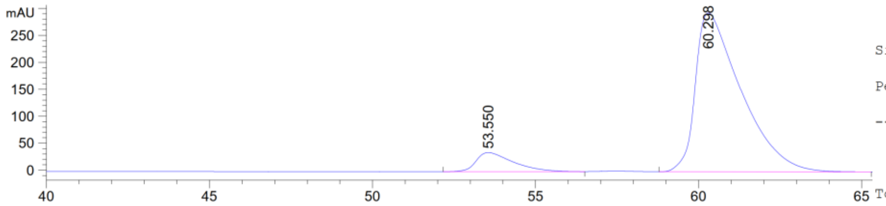

Signal 1: DAD1 A, Sig=220,4 Ref=off

Peak #	RetTime [min]	Type	Width [min]	Area [mAU*s]	Height [mAU]	Area %
1	53.550	BB	1.1363	2788.01563	35.34257	8.8497
2	60.298	BB	1.3910	2.87161e4	294.65335	91.1503
Totals :				3.15041e4	329.99592	

C: Asymmetric sample prepared using $[Ir(COD)_2]BAr^F{}_4$ / *(R)*-DTBM-Segphos (10 mol%) at 130 ˚C in the presence of 1.25 equiv of Et_3SiH for 3 h. (Table S4, entry 7, 78% yield, 92:8 er).

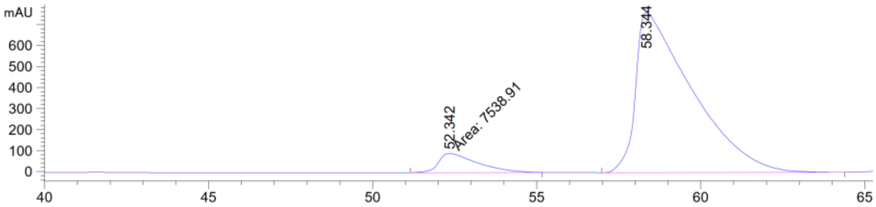

Signal 1: DAD1 A, Sig=220,4 Ref=off

Peak #	RetTime [min]	Type	Width [min]	Area [mAU*s]	Height [mAU]	Area %
1	52.342	MM	1.3743	7538.90820	91.43021	7.6384
2	58.344	BB	1.6437	9.11591e4	760.92499	92.3616
Totals :				9.86980e4	852.35520	

D: Asymmetric sample prepared using of $[Ir(COD)_2]BAr^F{}_4$ / *(R)*-DTBM-Segphos (10 mol%) at 130 ˚C in the presence of 0.5 equiv of Et_3SiH for 5 h. (Table S4, entry 8, 28% yield, 92:8 er).

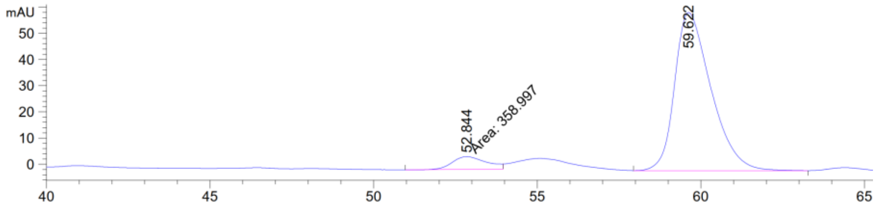

Signal 1: DAD1 A, Sig=220,4 Ref=off

Peak #	RetTime [min]	Type	Width [min]	Area [mAU*s]	Height [mAU]	Area %
1	52.844	MM	1.2261	358.99722	4.88004	7.1606
2	59.622	BB	1.1436	4654.52441	60.36646	92.8394
Totals :				5013.52164	65.24650	

E: Asymmetric sample prepared using 5 mol% of $[Ir(COD)_2]BAr^F{}_4$ / *(R)*-DTBM-Segphos at 130 ˚C in the presence of 1.25 equiv of Et_3SiH for 3 h. (Table S4, entry 9, 76% yield, 92:8 er).

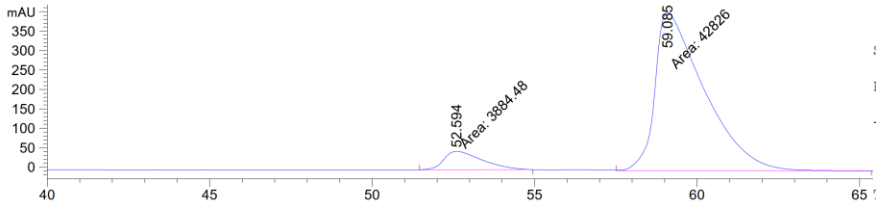

Signal 1: DAD1 A, Sig=220,4 Ref=off

Peak #	RetTime [min]	Type	Width [min]	Area [mAU*s]	Height [mAU]	Area %
1	52.594	MM	1.3664	3884.48389	47.38160	8.3161
2	59.085	MM	1.7563	4.28260e4	406.40744	91.6839
Totals :				4.67105e4	453.78904	

F: Asymmetric sample prepared using 2.5 mol% of $[Ir(COD)_2]BAr^F{}_4$ / *(R)*-DTBM-Segphos at 130 ˚C in the presence of 1.25 equiv of Et_3SiH for 3 h. (Table S4, entry 10, 63% yield, 91:9 er).

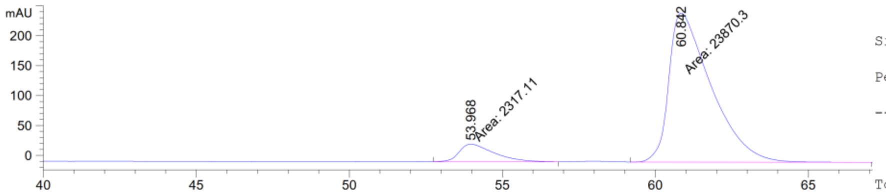

Signal 1: DAD1 A, Sig=220,4 Ref=off

Peak #	RetTime [min]	Type	Width [min]	Area [mAU*s]	Height [mAU]	Area %
1	53.968	MM	1.3129	2317.10547	29.41351	8.8482
2	60.842	MM	1.5995	2.38703e4	248.72136	91.1518
Totals :				2.61874e4	278.13487	

G: Asymmetric sample prepared using 10 mol% of $[Ir(COD)_2]BAr^F{}_4$ / *(R)*-DTBM-Segphos at 130 ˚C in the presence of 1.25 equiv of $BnMe_2SiH$ for 3 h. (Table S4, entry 13, 72% yield, 92:8 er).

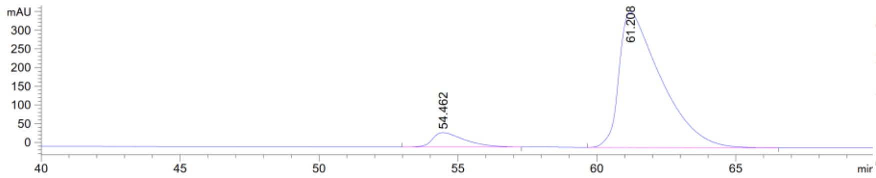

Signal 1: DAD1 A, Sig=220,4 Ref=off

Peak #	RetTime [min]	Type	Width [min]	Area [mAU*s]	Height [mAU]	Area %
1	54.462	BB	1.1340	3068.82568	38.41680	7.4945
2	61.208	BB	1.5061	3.78788e4	361.59390	92.5055
Totals :				4.09476e4	400.01070	

H: Asymmetric sample prepared using 10 mol% of $[Ir(COD)_2]BAr^F{}_4$ / *(R)*-DTBM-Segphos at 130 ˚C in the presence of 1.25 equiv of HBPin for 3h. (Table S4, entry 17, 45% yield, 90:10 er).

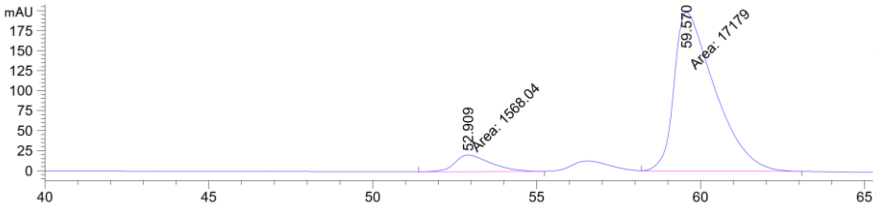

Signal 1: DAD1 A, Sig=220,4 Ref=off

Peak #	RetTime [min]	Type	Width [min]	Area [mAU*s]	Height [mAU]	Area %
1	52.909	MM	1.2607	1568.04407	20.72902	8.3642
2	59.570	MM	1.4415	1.71790e4	198.61937	91.6358
Totals :				1.87470e4	219.34839	

(R)-N,N-Diethyl-9a-methyl-1-oxo-2,3,9,9a-tetrahydro-1*H*-fluorene-5-carboxamide (3a)

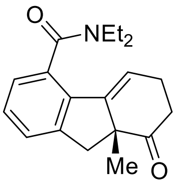

Prepared following General Procedure L from prochiral diketone **1a**. Obtained as colorless oil. **^{1}H NMR** (500 MHz, $CDCl_3$, 0 ˚C) δ 7.30 – 7.22 (m, 2H), 7.10 – 7.04 (m, 1H), 6.34 – 6.14 (m, 1H), 3.85 – 3.65 (m, 1H), 3.59 – 3.43 (m, 1H), 3.36 (2 x d, J = 16.6 Hz, 1H), 3.22 – 3.08 (m, 2H), 2.85 – 2.65 (m, 3H), 2.64 – 2.50 (m, 1H), 2.42 – 2.29 (m, 1H), 1.35 – 1.27 (m, 6H), 1.06 – 1.00 (m, 3H) ppm. **^{13}C NMR** (126 MHz, $CDCl_3$) δ 215.24 (CO), 170.18 (CO), 170.13 (CO), 145.31 (C), 145.06 (C), 143.41 (C), 142.96 (C), 133.24 (C), 133.09 (C), 132.31 (C), 132.05 (C), 128.55 (CH), 128.25 (CH), 126.16 (CH), 126.13 (CH), 124.69 (CH), 124.54 (CH), 120.27 (CH), 119.29 (CH), 55.18 (C), 54.99 (C), 42.91 (CH_2), 42.69 (CH_2), 40.22 (CH_2), 40.01 (CH_2), 38.91 (CH_2), 38.85 (CH_2), 35.56 (CH_2), 35.28 (CH_2), 24.35 (CH_3), 24.09 (CH_3), 23.75 (CH_2), 23.66 (CH_2), 14.07 (CH_3 amide), 13.93 (CH_3 amide), 12.85 (CH_3 amide), 12.74 (CH_3 amide) ppm. Note: most of the signals are duplicated due to rotamerism. **HRMS** [APCI]: m/z calculated for $C_{19}H_{24}NO_2$ $[M + H]^+$: 298.1802, found 298.1801.

Enantioselectivity was determined by chiral HPLC analysis on a Chiralpack IG3 column at rt (Hexane:*i*PrOH = 85:15, 1 mL/min).

A: Racemic sample prepared using [Ir(COD)$_2$]BAr$^F{}_4$ / *rac*-Binap (10 mol%) at 130 ˚C.

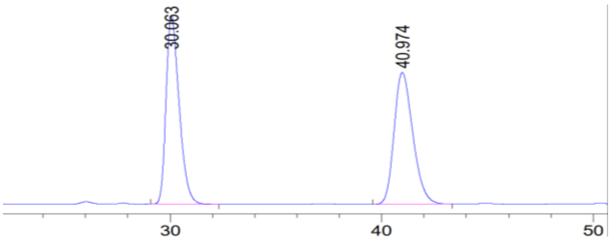

Signal 2: DAD1 B, Sig=254,4 Ref=off

Peak #	RetTime [min]	Type	Width [min]	Area [mAU*s]	Height [mAU]	Area %
1	30.063	BB	0.6568	9070.25098	211.32121	49.9914
2	40.974	BB	0.9391	9073.36230	147.61282	50.0086
Totals :				1.81436e4	358.93404	

B: Asymmetric sample prepared using [Ir(COD)$_2$]BAr$^F{}_4$ / *(S)*-Binap (10 mol%) at 130 ˚C (Table S1, entry 2, 11% yield, 86:14 er).

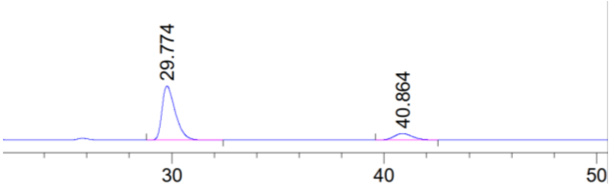

Signal 2: DAD1 B, Sig=254,4 Ref=off

Peak #	RetTime [min]	Type	Width [min]	Area [mAU*s]	Height [mAU]	Area %
1	29.774	BB	0.6569	1.60965e4	371.95630	85.9019
2	40.864	BB	0.9004	2641.71826	44.38205	14.0981
Totals :				1.87382e4	416.33835	

C: Asymmetric sample prepared using [Ir(COD)$_2$]BAr$^F{}_4$ / *(S)*-SDP (10 mol%) at 130 ˚C (Table S1, entry 6, 31% yield, 58:42 er).

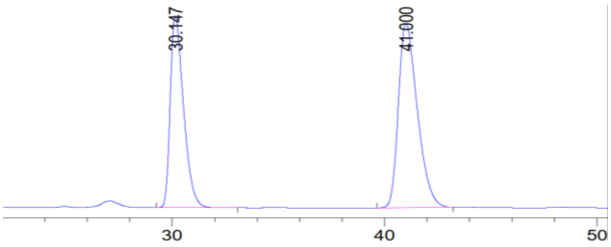

Signal 2: DAD1 B, Sig=254,4 Ref=off

Peak #	RetTime [min]	Type	Width [min]	Area [mAU*s]	Height [mAU]	Area %
1	30.147	BB	0.6646	1.40568e4	323.69339	42.2042
2	41.000	BB	0.9445	1.92498e4	310.82568	57.7958
Totals :				3.33066e4	634.51907	

D: Asymmetric sample prepared using [Ir(COD)$_2$]BAr$^F{}_4$ / *(S,S)*-Chiraphos (10 mol%) at 130 ˚C (Table S1, entry 8, 17% yield, 67:13 er).

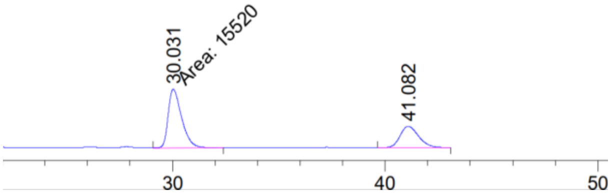

Signal 2: DAD1 B, Sig=254,4 Ref=off

Peak #	RetTime [min]	Type	Width [min]	Area [mAU*s]	Height [mAU]	Area %
1	30.031	MM	0.7349	1.55200e4	351.95459	66.8506
2	41.082	BBA	0.9233	7695.95605	127.29644	33.1494
Totals :				2.32160e4	479.25103	

E: Asymmetric sample prepared using [Ir(COD)$_2$]BAr$^F{}_4$ / *(R)*-BTFM-Garphos (10 mol%) at 130 ˚C (Table S1, entry 9, 34% yield, 77:23 er).

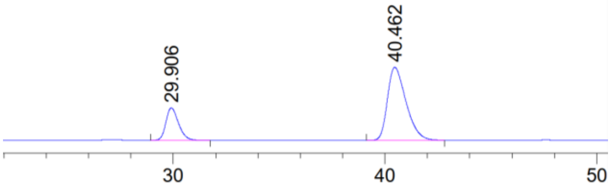

Signal 2: DAD1 B, Sig=254,4 Ref=off

Peak #	RetTime [min]	Type	Width [min]	Area [mAU*s]	Height [mAU]	Area %
1	29.906	BB	0.6364	5643.30176	136.48903	23.2501
2	40.462	BB	0.9319	1.86288e4	306.13339	76.7499
Totals :				2.42721e4	442.62242	

F: Asymmetric sample prepared using $[Ir(COD)_2]BAr^F{}_4$ / *(R)*-Xyl-Garphos (10 mol%) at 130 ˚C (Table S1, entry 10, 15% yield, 85:15 er).

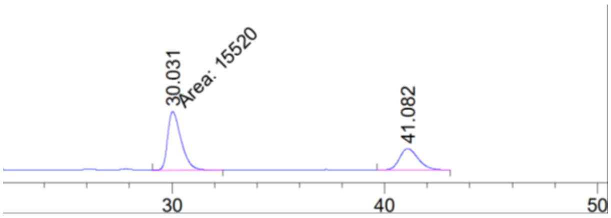

Signal 2: DAD1 B, Sig=254,4 Ref=off

Peak #	RetTime [min]	Type	Width [min]	Area [mAU*s]	Height [mAU]	Area %
1	30.031	MM	0.7349	1.55200e4	351.95459	66.8506
2	41.082	BBA	0.9233	7695.95605	127.29644	33.1494
Totals :				2.32160e4	479.25103	

G: Asymmetric sample prepared using $[Ir(COD)_2]BAr^F{}_4$ / *(R)*-Garphos (10 mol%) at 130 ˚C (Table S1, entry 11, 28% yield, 88:12 er).

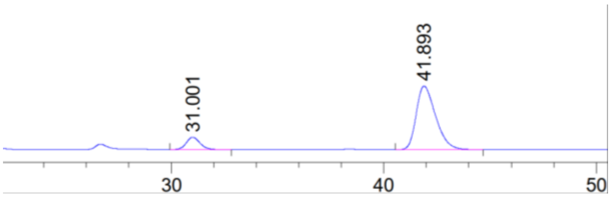

Signal 2: DAD1 B, Sig=254,4 Ref=off

Peak #	RetTime [min]	Type	Width [min]	Area [mAU*s]	Height [mAU]	Area %
1	31.001	BB	0.6696	2142.04834	48.09890	12.0759
2	41.893	BB	0.9764	1.55962e4	245.75458	87.9241
Totals :				1.77382e4	293.85348	

H: Asymmetric sample prepared using $[Ir(COD)_2]BAr^F{}_4$ / *(R)*-MeO-Biphep (10 mol%) at 130 ˚C (Table S1, entry 13, 27% yield, 89:11 er).

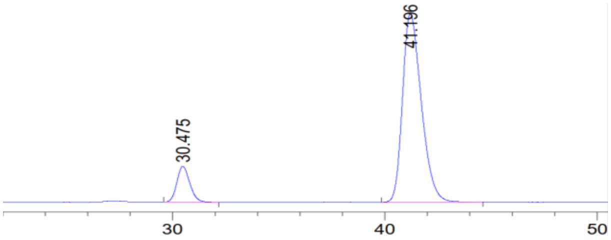

Signal 2: DAD1 B, Sig=254,4 Ref=off

Peak #	RetTime [min]	Type	Width [min]	Area [mAU*s]	Height [mAU]	Area %
1	30.475	BB	0.6493	1874.00647	44.50229	11.4669
2	41.196	BB	0.9499	1.44688e4	235.13129	88.5331
Totals :				1.63428e4	279.63357	

I: Asymmetric sample prepared using $[Ir(COD)_2]BAr^F{}_4$ / *(R)*-Fur-Biphep (10 mol%) at 130 ˚C (Table S1, entry 14, 18% yield, 83:17 er).

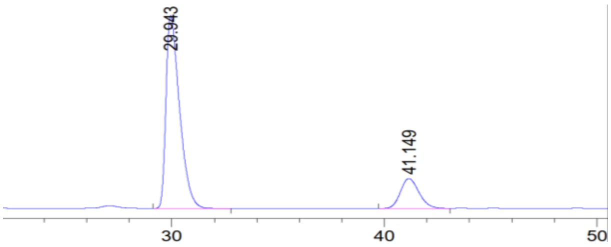

Signal 2: DAD1 B, Sig=254,4 Ref=off

Peak #	RetTime [min]	Type	Width [min]	Area [mAU*s]	Height [mAU]	Area %
1	29.943	BB	0.6710	2.27459e4	513.33368	82.5609
2	41.149	BB	0.9118	4804.55176	79.64384	17.4391
Totals :				2.75505e4	592.97752	

J: Asymmetric sample prepared using $[Ir(COD)_2]BAr^F{}_4$ / *(S)*-C_3-Tunephos (10 mol%) at 130 ˚C (Table S1, entry 15, 15% yield, 87:13 er).

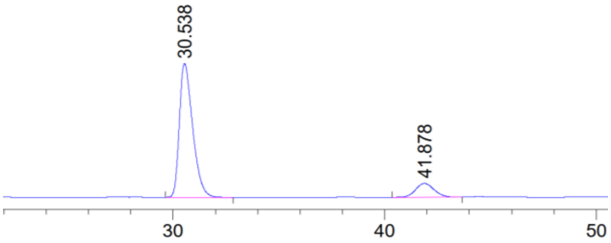

Signal 2: DAD1 B, Sig=254,4 Ref=off

Peak #	RetTime [min]	Type	Width [min]	Area [mAU*s]	Height [mAU]	Area %
1	30.538	BB	0.6695	1.19513e4	274.73361	87.3839
2	41.878	BB	0.9181	1725.47815	28.26974	12.6161
Totals :				1.36768e4	303.00335	

K: Asymmetric sample prepared using [Ir(COD)$_2$]BAr$^F{}_4$ / *(S)*-Synphos (10 mol%) at 130 ˚C (Table S1, entry 16, 42% yield, 88:12 er).

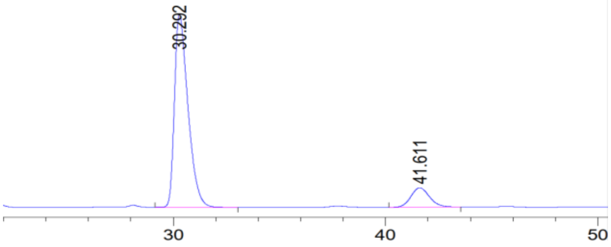

Signal 2: DAD1 B, Sig=254,4 Ref=off

Peak #	RetTime [min]	Type	Width [min]	Area [mAU*s]	Height [mAU]	Area %
1	30.292	BB	0.6701	1.64804e4	373.99960	87.8005
2	41.611	BB	0.9090	2289.87793	37.57164	12.1995
Totals :				1.87702e4	411.57124	

L: Asymmetric sample prepared using [Ir(COD)$_2$]BAr$^F{}_4$ / *(R)*-Segphos (10 mol%) at 130 ˚C (Table S1, entry 17, 32% yield, 90:10 er).

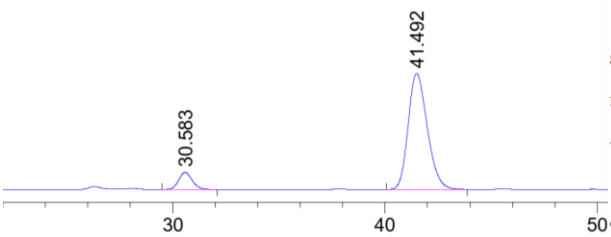

Signal 2: DAD1 B, Sig=254,4 Ref=off

Peak #	RetTime [min]	Type	Width [min]	Area [mAU*s]	Height [mAU]	Area %
1	30.583	BB	0.6567	877.47595	19.81256	9.4351
2	41.492	BB	0.9621	8422.65625	134.59262	90.5649
Totals :				9300.13220	154.40518	

M: Asymmetric sample prepared using [Ir(COD)$_2$]BAr$^F{}_4$ / *(R)*-Segphos (10 mol%) at 130 ˚C for 96 h (Table S1, entry 18, 93% yield, 86:14 er).

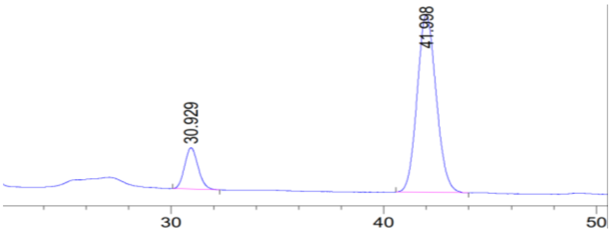

Signal 1: DAD1 A, Sig=220,4 Ref=off

Peak #	RetTime [min]	Type	Width [min]	Area [mAU*s]	Height [mAU]	Area %
1	30.928	BB	0.6223	420.58707	9.46682	14.2089
2	41.998	BB	0.9368	2539.43555	40.53845	85.7911
Totals :				2960.02261	50.00527	

N: Asymmetric sample prepared using [Ir(COD)$_2$]BAr$^F{}_4$ / *(R)*-Segphos (10 mol%) at 100 ˚C for 96 h (Table S1, entry 19, 36% yield, 91:9 er).

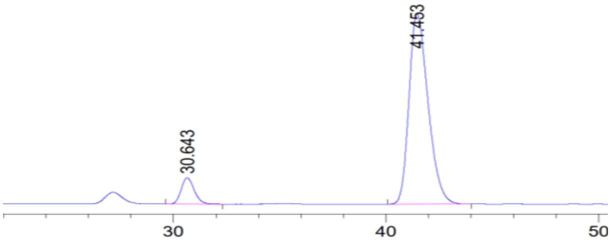

Signal 2: DAD1 B, Sig=254,4 Ref=off

Peak #	RetTime [min]	Type	Width [min]	Area [mAU*s]	Height [mAU]	Area %
1	30.643	BB	0.6487	1210.91858	28.44452	8.6409
2	41.453	BB	0.9533	1.28029e4	205.93584	91.3591
Totals :				1.40138e4	234.38036	

O: Asymmetric sample prepared using [Ir(COD)$_2$]BAr$^F{}_4$ / *(R)*-Difluorphos (10 mol%) at 130 ˚C (Table S1, entry 20, 20% yield, 91:9 er).

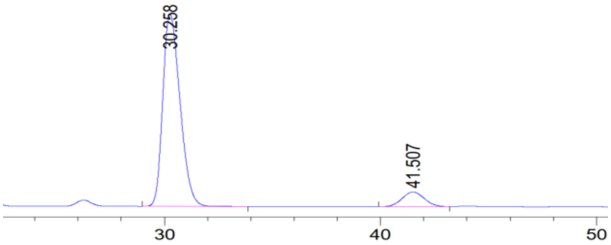

Signal 2: DAD1 B, Sig=254,4 Ref=off

Peak #	RetTime [min]	Type	Width [min]	Area [mAU*s]	Height [mAU]	Area %
1	30.258	BB	0.8660	1.79027e4	323.32187	91.1247
2	41.507	BB	1.0517	1743.67542	24.45950	8.8753
Totals :				1.96464e4	347.78137	

(*R*)-9a-Methyl-5-(pyrrolidine-1-carbonyl)-2,3,9,9a-tetrahydro-1*H*-fluoren-1-one (3a^{I})

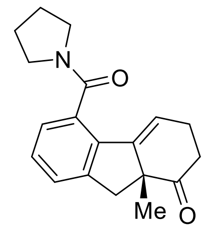

Prepared following General Procedure L from prochiral diketone **1a^{I}**. Obtained as yellow oil. **^{1}H NMR** (300 MHz, $CDCl_3$) δ 7.28 – 7.19 (m, 2H), 7.15 – 7.05 (m, 1H), 6.20 (dd, *J* = 6.9, 2.8 Hz, 1H), 3.66 (hept, *J* = 6.2 Hz, 2H), 3.34 (d, *J* = 16.7 Hz, 1H), 3.13 (t, *J* = 6.3 Hz, 2H), 2.79 – 2.65 (m, 3H), 2.61 – 2.49 (m, 1H), 2.40 – 2.26 (m, 1H), 1.92 (dp, *J* = 25.3, 6.5 Hz, 4H), 1.31 (s, 3H) ppm. **^{13}C NMR** (75 MHz, $CDCl_3$) δ 210.95 (CO), 169.32 (CO), 137.46 (C), 136.73 (C), 131.43 (CH), 128.47 (CH), 125.91 (CH), 65.32 (C), 49.47 (CH_2), 46.16 (CH_2), 42.79 (CH_2), 39.10 (CH_2), 26.41 (CH_2), 24.44 (CH_2), 22.49 (CH_3), 16.71 (CH_2) ppm. **HRMS** [APCI]: *m/z* calculated for $C_{19}H_{22}NO_2$ $[M + H]^+$: 296.1645, found 296.1654.

Enantioselectivity was determined by chiral HPLC analysis on a Chiralpack IG3 column at rt (Hexane:*i*PrOH = 85:15, 1 mL/min).

A: Racemic sample prepared using $[Ir(COD)_2]BAr^F_4$ / *rac*-Binap (10 mol%) at 130 ˚C.

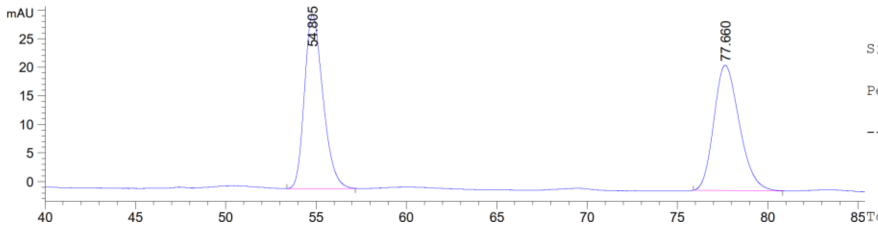

Signal 2: DAD1 B, Sig=254,4 Ref=off

Peak #	RetTime [min]	Type	Width [min]	Area [mAU*s]	Height [mAU]	Area %
1	54.805	BB	1.0133	2163.95337	30.36803	50.1148
2	77.660	BB	1.1650	2154.04321	21.90953	49.8852
Totals :				4317.99658	52.27755	

B: Asymmetric sample prepared using $[Ir(COD)_2]BAr^F_4$ / *(R)*-Segphos (10 mol%) at 130 ˚C (Table S2, entry 5, 99% yield, 86:14 er).

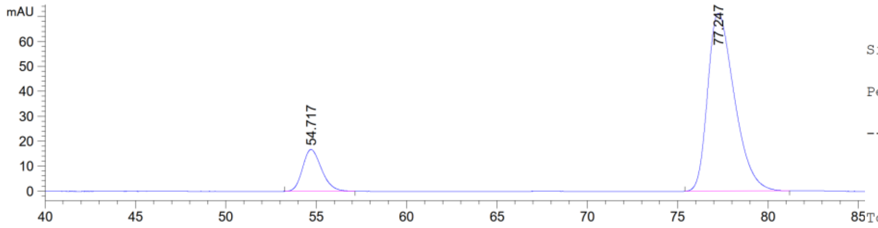

Signal 2: DAD1 B, Sig=254,4 Ref=off

Peak #	RetTime [min]	Type	Width [min]	Area [mAU*s]	Height [mAU]	Area %
1	54.717	BB	1.0390	1225.31812	16.75934	14.3093
2	77.247	BB	1.4367	7337.76758	71.29932	85.6907
Totals :				8563.08569	88.05866	

C: Asymmetric sample prepared using $[Ir(COD)_2]BAr^F_4$ / *(R)*-DTBM-Segphos (10 mol%) at 130 ˚C (Table S2, entry 6, 90% yield, 88:12 er).

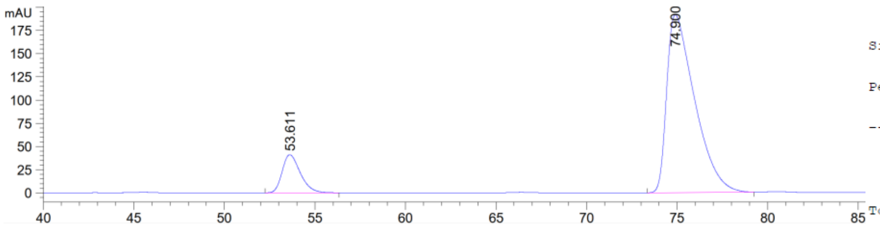

Signal 2: DAD1 B, Sig=254,4 Ref=off

Peak #	RetTime [min]	Type	Width [min]	Area [mAU*s]	Height [mAU]	Area %
1	53.611	BB	1.0422	2852.89722	41.08267	12.2058
2	74.900	BB	1.5488	2.05204e4	191.70538	87.7942
Totals :				2.33733e4	232.78805	

Methyl (*R*)-9a-methyl-1-oxo-2,3,9,9a-tetrahydro-1*H*-fluorene-5-carboxylate (3a^{III})

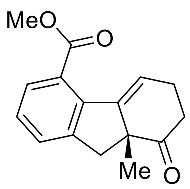

Prepared following General Procedure L from prochiral diketone **1a^{III}**. Obtained as colorless oil. **^{1}H NMR** (500 MHz, $CDCl_3$) δ 7.63 (d, *J* = 7.7 Hz, 1H), 7.40 (dd, *J* = 7.6, 1.0 Hz, 1H), 7.24 (d, *J* = 7.6 Hz, 1H), 6.77 (dd, *J* = 6.3, 3.1 Hz, 1H), 3.93 (s, 3H), 3.33 (d, *J* = 16.5 Hz, 1H), 2.88 – 2.72 (m, 3H), 2.68 – 2.55 (m, 1H), 2.42 – 2.29 (m, 1H), 1.34 (s, 3H) ppm. **^{13}C NMR** (126 MHz, $CDCl_3$) δ 214.80 (CO), 168.58 (CO), 144.49 (C), 143.79 (C), 137.35 (C), 129.40 (CH), 128.93 (CH), 127.76 (CH), 127.47 (C), 123.57 (CH), 55.01 (C), 52.33

(CH$_3$), 40.32 (CH$_2$), 35.15 (CH$_2$), 24.98 (CH$_3$), 24.67 (CH$_2$) ppm. **HRMS** [APCI]: *m/z* calculated for $C_{16}H_{17}O_3$ [M + H]$^+$: 257.1172, found 257.1163.

Enantioselectivity was determined by chiral HPLC analysis on a Chiralpack IF3 column at rt (Hexane:*i*PrOH = 98:2, 1 mL/min).

A: Racemic sample prepared using [Ir(COD)$_2$]BAr$^F{}_4$ / *rac*-Binap (10 mol%) at 130 ˚C.

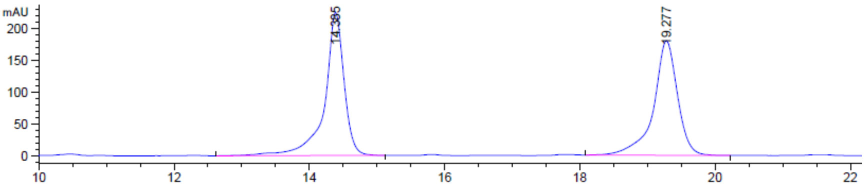

Signal 1: DAD1 A, Sig=220,4 Ref=off

Peak #	RetTime [min]	Type	Width [min]	Area [mAU*s]	Height [mAU]	Area %
1	14.385	BB	0.2882	4497.55420	224.07028	50.4825
2	19.277	BB	0.3609	4411.57275	179.42569	49.5175
Totals :				8909.12695	403.49597	

B: Asymmetric sample prepared using [Ir(COD)$_2$]BAr$^F{}_4$ / *(R)*-DTBM-Segphos (10 mol%) at 130 ˚C (Table S2, entry 10, 31% yield, 74:26 er).

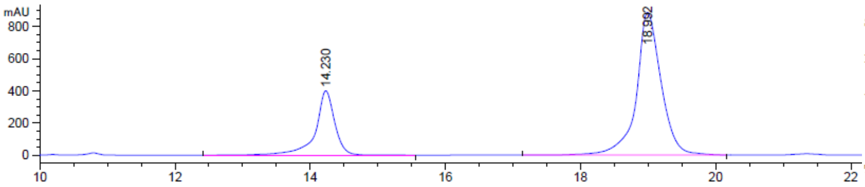

Signal 1: DAD1 A, Sig=220,4 Ref=off

Peak #	RetTime [min]	Type	Width [min]	Area [mAU*s]	Height [mAU]	Area %
1	14.230	BB	0.2907	8050.19287	400.18585	26.3058
2	18.992	BB	0.3751	2.25522e4	885.56976	73.6942
Totals :				3.06024e4	1285.75562	

(*R*)-5-acetyl-9a-methyl-2,3,9,9a-tetrahydro-1*H*-fluoren-1-one (3a^{IV})

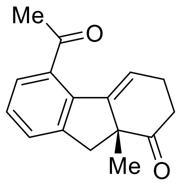

Prepared following General Procedure L from prochiral diketone **1a^{IV}**. Obtained as colorless oil. **^{1}H NMR** (500 MHz, CDCl$_3$) δ 7.41 – 7.30 (m, 1H), 7.29 – 7.23 (m, 1H), 6.40 (dd, *J* = 6.3, 3.0 Hz, 1H), 3.33 (d, *J* = 16.5 Hz, 1H), 2.84 – 2.71 (m, 3H), 2.60 (s, 3H), 2.64 – 2.55 (m, 1H), 2.39 – 2.31 (m, 1H), 1.35 (s, 3H) ppm. **^{13}C NMR** (126 MHz, CDCl$_3$) δ 214.49 (CO), 203.60 (CO), 144.93 (C), 143.68 (C), 137.12 (C), 135.36 (C), 128.57 (CH), 128.03 (CH), 126.47 (CH), 122.84 (CH), 54.93 (C), 40.33 (CH$_2$), 35.11 (CH$_2$), 30.24 (CH$_3$), 24.88 (CH$_3$), 24.59 (CH$_2$) ppm. **HRMS** [APCI]: *m/z* calculated for $C_{16}H_{17}O_2$ [M + H]$^+$: 241.1223, found 241.1222.

Enantioselectivity was determined by chiral HPLC analysis on a Chiralpack IF3 column at rt (Hexane:*i*PrOH = 98:2, 1 mL/min).

A: Racemic sample prepared using [Ir(COD)$_2$]BAr$^F{}_4$ / *rac*-Binap (10 mol%) at 130 ˚C.

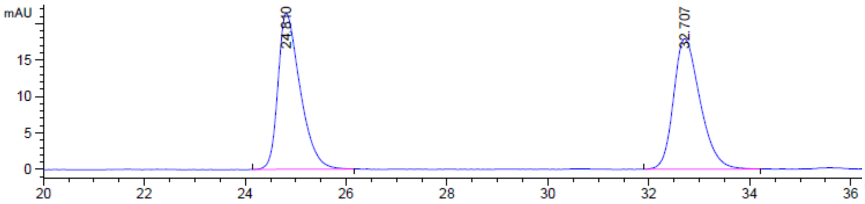

Signal 4: DAD1 D, Sig=300,4 Ref=off

Peak #	RetTime [min]	Type	Width [min]	Area [mAU*s]	Height [mAU]	Area %
1	24.810	BB	0.4519	652.80096	21.40009	49.9840
2	32.707	BB	0.5501	653.22015	17.88713	50.0160
Totals :				1306.02112	39.28723	

B: Asymmetric sample prepared using $[Ir(COD)_2]BAr^F{}_4$ / *(R)*-DTBM-Segphos (10 mol%) at 130 ˚C (Table S2, entry 12, 51% yield, 64:36 er).

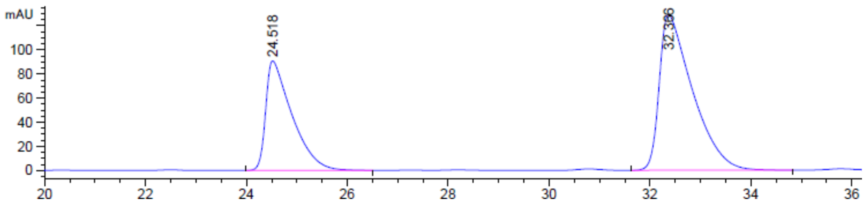

Signal 4: DAD1 D, Sig=300,4 Ref=off

Peak #	RetTime [min]	Type	Width [min]	Area [mAU*s]	Height [mAU]	Area %
1	24.518	BB	0.5206	3274.86035	90.83945	35.7263
2	32.366	BB	0.6647	5891.67383	129.03223	64.2737
Totals :				9166.53418	219.87167	

(4a*R*,9a*R*)-4a-Hydroxy-*N,N*-dimethyl-1-oxo-9a-propyl-2,3,4,4a,9,9*a*-hexahydro-1*H*-fluorene-5-carboxamide (2c)

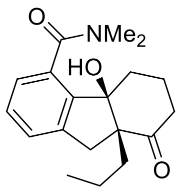

Prepared following General Procedure L from prochiral diketone **1c** in the presence of 1.25 equiv of Et_3SiH. Reaction time: 3 h. Obtained as pale-yellow oil. **^{1}H NMR** (500 MHz, $CDCl_3$) δ ^{1}H NMR (500 MHz, $CDCl_3$) δ 7.29 (d, *J* = 7.5 Hz, 1H), 7.21 (t, *J* = 7.5 Hz, 1H), 7.01 (d, *J* = 7.5 Hz, 1H), 3.74 (d, *J* = 15.3 Hz, 1H), 3.15 (s, 3H), 3.06 (s, 3H), 2.69 (d, *J* = 15.3 Hz, 1H), 2.51 (ddd, *J* = 14.2, 12.4, 6.5 Hz, 1H), 2.32 – 2.27 (m, 1H), 2.24 – 2.16 (m, 2H), 2.05 – 1.97 (m, 1H), 1.97 – 1.91 (m, 1H), 1.86 – 1.78 (m, 1H), 1.29 – 1.18 (m, 2H), 1.11 – 1.02 (m, 1H), 0.93 (t, *J* = 7.3 Hz, 3H) ppm. **^{13}C NMR** (125 MHz, $CDCl_3$) δ ^{13}C NMR (126 MHz, $CDCl_3$) δ 212.8 (CO), 171.6 (CO), 145.9 (C), 142.9 (C), 131.5 (C), 128.2 (CH), 126.7 (CH), 124.7 (CH), 85.3(COH), 66.4 (C), 39.7 (CH_3 amide), 38.5 (CH_2), 36.4 (CH_2), 35.1 (CH_3 amide), 33.8 (CH_2), 32.3 (CH_2), 20.2 (CH_2), 18.7 (CH_2), 14.9 (CH_3) ppm. **HRMS** [APCI]: *m/z* calculated for $C_{19}H_{26}NO_3$ $[M + H]^+$: 316.1907, found 316.1908.

Enantioselectivity was determined by chiral HPLC analysis on a Chiralpack IF3 column at rt (Hexane:*i*PrOH = 95:5, 1 mL/min).

A: Racemic sample prepared using $[Ir(COD)_2]BAr^F{}_4$ / *rac*-DTBM-Segphos (5 mol%) in the presence of 1.25 equiv of Et_3SiH, at 130 ˚C for 3 h.

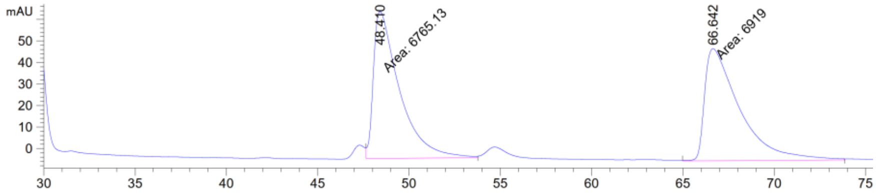

Signal 1: DAD1 A, Sig=220,4 Ref=off

Peak #	RetTime [min]	Type	Width [min]	Area [mAU*s]	Height [mAU]	Area %
1	48.410	FM	1.6536	6765.13281	68.18514	49.4378
2	66.642	MM	2.2140	6918.99707	52.08625	50.5622
Totals :				1.36841e4	120.27140	

B: Asymmetric sample prepared using $[Ir(COD)_2]BAr^F{}_4$ / *(R)*-DTBM-Segphos (5 mol%) in the presence of 1.25 equiv of Et_3SiH, at 130 ˚C for 3 h. (69% yield, 96:4 er).

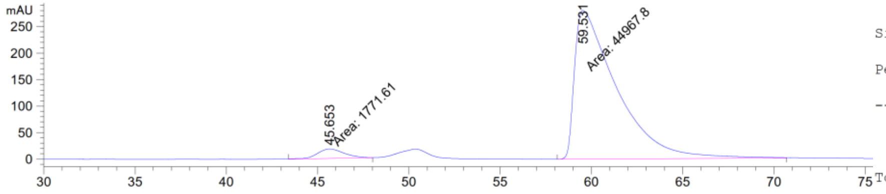

Signal 1: DAD1 A, Sig=220,4 Ref=off

Peak #	RetTime [min]	Type	Width [min]	Area [mAU*s]	Height [mAU]	Area %
1	45.653	MM	1.6824	1771.61353	17.54996	3.7904
2	59.531	MM	2.6749	4.49678e4	280.17972	96.2096
Totals :				4.67394e4	297.72968	

(R)-N,N-Dimethyl-1-oxo-9a-propyl-2,3,9,9a-tetrahydro-1H-fluorene-5-carboxamide (3c)

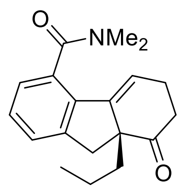

Prepared following General Procedure L from prochiral diketone **1c**. Obtained as yellow oil. **^{1}H NMR** (300 MHz, $CDCl_3$) δ 7.31 – 7.17 (m, 2H), 7.06 (s, 1H), 6.14 (br d, J = 51.1 Hz, 1H), 3.33 (br d, J = 16.9 Hz, 1H), 3.17 (d, J = 1.4 Hz, 3H), 2.96 – 2.50 (m, 8H), 2.45 – 2.27 (m, 1H), 1.79 – 1.54 (m, 3H), 1.38 – 1.07 (m, 2H), 0.83 (t, J = 7.3 Hz, 3H) ppm. **^{13}C NMR** (75 MHz, $CDCl_3$) δ 213.74 (CO), 170.77 (CO), 145.07 (C), 134.28 (C), 131.57 (C), 128.58 (CH), 125.96 (CH), 124.79 (CH), 120.33 (CH), 59.60 (C), 40.00 (CH_2), 38.36 (CH_3 amide), 37.60 (CH_2), 35.30 (CH_2), 34.82 (CH_3 amide), 24.29 (CH_2), 18.14 (CH_2), 14.46 (CH_3) ppm. **HRMS** [APCI]: m/z calculated for $C_{19}H_{24}NO_2$ $[M + H]^+$: 298.1802, found 298.1808.

Enantioselectivity was determined by chiral HPLC analysis on a Chiralpack IA3 column at rt (Hexane:*i*PrOH = 98:2, 1 mL/min).

A: Racemic sample prepared using $[Ir(COD)_2]BAr^F_4$ / *rac*-Binap (5 mol%) at 130 ˚C.

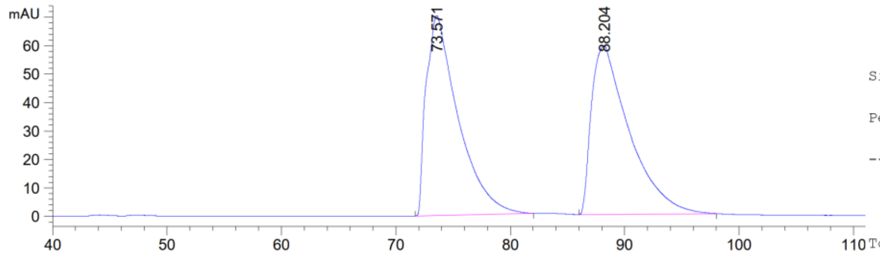

Signal 2: DAD1 B, Sig=254,4 Ref=off

Peak #	RetTime [min]	Type	Width [min]	Area [mAU*s]	Height [mAU]	Area %
1	73.571	BB	2.4031	1.36043e4	70.25624	49.8985
2	88.204	BB	2.6951	1.36597e4	59.26249	50.1015
Totals :				2.72640e4	129.51873	

B: Asymmetric sample prepared using $[Ir(COD)_2]BAr^F_4$ / *(R)*-DTBM-Segphos (5 mol%) at 130 ˚C (83% yield, 92:8 er).

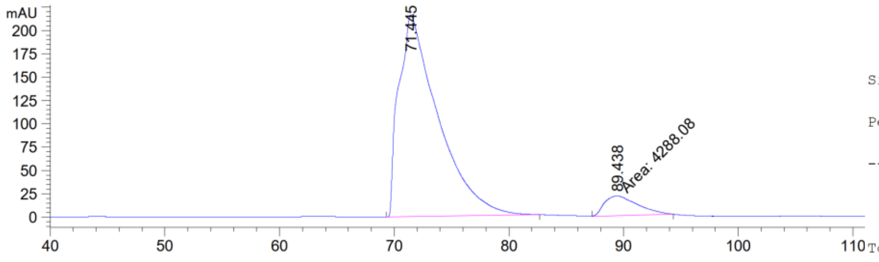

Signal 2: DAD1 B, Sig=254,4 Ref=off

Peak #	RetTime [min]	Type	Width [min]	Area [mAU*s]	Height [mAU]	Area %
1	71.445	BB	2.9823	5.14544e4	216.07074	92.3073
2	89.438	MM	3.3635	4288.08203	21.24780	7.6927
Totals :				5.57425e4	237.31854	

Methyl-(R)-3-(4-(dimethylcarbamoyl)-8-oxo-6,7,8,9-tetrahydro-8aH-fluoren-8a-yl)propanoate (3d)

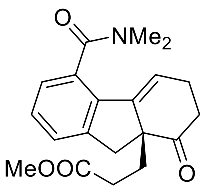

Prepared following General Procedure L from prochiral diketone **1d**. Obtained as yellow oil. **^{1}H NMR** (500 MHz, $CDCl_3$, 55 ˚C) δ 7.25 – 7.09 (m, 2H), 6.99 (d, J = 6.6 Hz, 1H), 6.18 (br s, 1H), 3.54 (s, 3H), 3.24 (dd, J = 16.5, 6.3 Hz, 1H), 3.08 (s, 3H), 2.76 (s + d, 5H), 2.64 – 2.56 (m, 1H), 2.54 – 2.43 (m, 1H), 2.31 – 2.10 (m, 3H), 2.01 – 1.85 (m, 2H).ppm. **^{13}C NMR** (126 MHz, $CDCl_3$, 55 ˚C) δ 213.0 (CO), 173.3 (CO), 170.6 (CO), 144.5 (C), 134.1 (C), 132.0 (C), 129.5 (CH), 128.8 (CH), 126.1 (CH), 125.8 (CH), 125.1 (CH), 58.6 (C), 51.8 (CH_3), 38.4 (CH_3 amide), 37.4 (CH_2), 35.5 (CH_2), 34.9 (CH_3 amide), 29.6 (CH_2), 24.1 (CH_2) ppm. [**NOTE**: Some carbon signals are duplicated due to strong rotamerism that persist even measuring NMR at higher temperatures. Only signals of the major rotamer were assigned. Purity was confirmed by GC-MS and HPLC]. **HRMS** [APCI]: m/z calculated for $C_{20}H_{24}NO_4$ $[M + H]^+$: 342.1700, found 342.1696.

Enantioselectivity was determined by chiral HPLC analysis on a Chiralpack IA3 column at rt (Hexane:*i*PrOH = 95:5, 1 mL/min).

A: Racemic sample prepared using $[Ir(COD)_2]BAr^F_4$ / *rac*-Binap (5 mol%) at 130 ˚C.

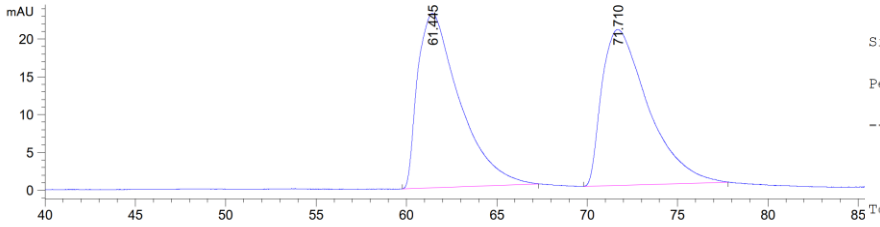

Signal 2: DAD1 B, Sig=254,4 Ref=off

Peak #	RetTime [min]	Type	Width [min]	Area [mAU*s]	Height [mAU]	Area %
1	61.445	BB	1.9013	3710.27759	23.08197	50.3530
2	71.710	BB	2.0800	3658.25049	20.61841	49.6470
Totals :				7368.52808	43.70038	

B: Asymmetric sample prepared using $[Ir(COD)_2]BAr^F_4$ / *(R)*-DTBM-Segphos (5 mol%) at 130 ˚C (62% yield, 87:13 er).

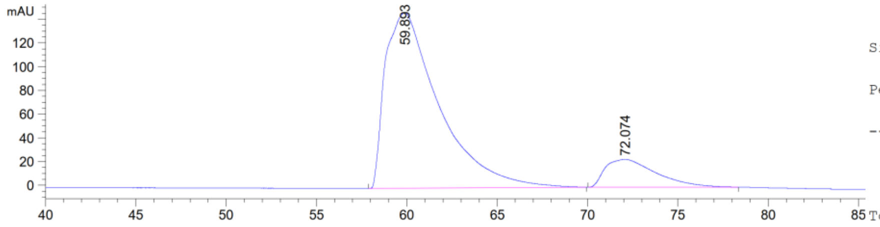

Signal 2: DAD1 B, Sig=254,4 Ref=off

Peak #	RetTime [min]	Type	Width [min]	Area [mAU*s]	Height [mAU]	Area %
1	59.893	BB	2.5692	3.06300e4	147.83817	86.9404
2	72.074	BB	2.3013	4601.02979	23.44714	13.0596
Totals :				3.52310e4	171.28531	

(R)-9a-*iso*Butyl-*N,N*-dimethyl-1-oxo-2,3,9,9a-tetrahydro-1*H*-fluorene-5-carboxamide (3e)

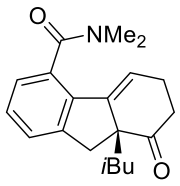

Prepared following General Procedure L from prochiral diketone **1e**. Obtained as yellow oil. **^{1}H NMR** (300 MHz, $CDCl_3$) δ 7.32 – 7.17 (m, 2H), 7.07 (br m, 1H), 6.13 (br d, *J* = 54.8 Hz, 1H), 3.46 – 3.31 (m, 1H), 3.17 (d, *J* = 1.1 Hz, 3H), 2.92 (d, *J* = 16.9 Hz, 1H), 2.85 (s, 3H), 2.82 – 2.67 (m, 2H), 2.65 – 2.48 (m, 1H), 2.45 – 2.31 (m, 1H), 1.66 (m, 4H), 0.86 (dd, *J* = 17.1, 6.3 Hz, 6H) ppm. **^{13}C NMR** (75 MHz, $CDCl_3$) δ 213.79 (CO), 170.78 (CO), 145.88 (C), 143.50 (C), 134.12 (C), 131.69 (C), 128.60 (CH), 126.12 (CH), 124.82 (CH), 120.58 (CH), 59.85 (C), 46.31 (CH_3), 38.42 (CH_2), 37.82 (CH_3), 35.54 (CH), 34.84 (CH_2), 25.29 (CH_2), 24.49 (CH_3), 23.96 (CH_2) ppm. **HRMS** [APCI]: *m/z* calculated for $C_{20}H_{26}NO_2$ $[M + H]^+$: 312.1958, found 312.1961.

Enantioselectivity was determined by chiral HPLC analysis on a Chiralpack IE3 column at rt (Hexane:*i*PrOH = 85:15, 1 mL/min).

A: Racemic sample prepared using $[Ir(COD)_2]BAr^F_4$ / *rac*-Binap (5 mol%) at 130 ˚C.

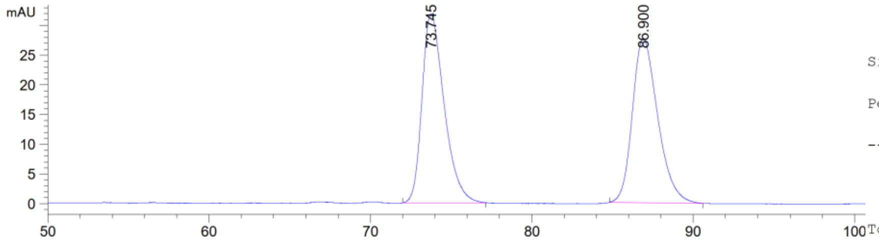

Signal 2: DAD1 B, Sig=254,4 Ref=off

Peak #	RetTime [min]	Type	Width [min]	Area [mAU*s]	Height [mAU]	Area %
1	73.745	BB	1.2353	3036.65112	31.82165	50.1676
2	86.900	BB	1.3160	3016.36035	27.66604	49.8324
Totals :				6053.01147	59.48769	

B: Asymmetric sample prepared using [Ir(COD)$_2$]BAr$^F_4$ / *(R)*-DTBM-Segphos (5 mol%) at 130 ˚C (87% yield, 86:14 er).

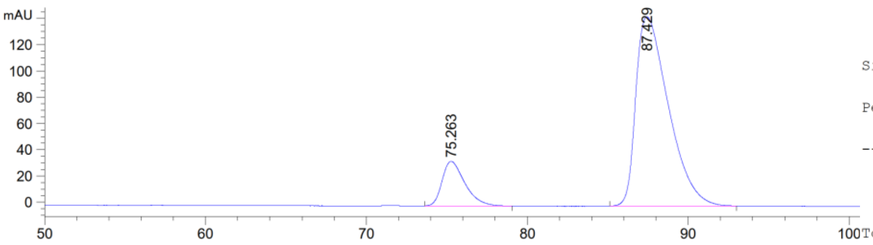

Signal 2: DAD1 B, Sig=254,4 Ref=off

Peak #	RetTime [min]	Type	Width [min]	Area [mAU*s]	Height [mAU]	Area %
1	75.263	BB	1.2579	3332.41870	33.92892	14.1122
2	87.429	BB	1.9546	2.02813e4	144.05305	85.8878
Totals :				2.36137e4	177.98198	

(4a*R*,9a*R*)-9a-benzyl-4a-hydroxy-*N,N*-dimethyl-1-oxo-2,3,4,4a,9,9a-hexahydro-1*H*-fluorene-5-carboxamide (2f)

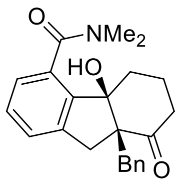

Prepared following General Procedure L from prochiral diketone **1f** in the presence of 1.25 equiv of Et_3SiH. Reaction time: 3 h. Obtained as pale-yellow oil. **^{1}H NMR** (500 MHz, $CDCl_3$) δ 7.26 – 7.17 (m, 5H), 7.14 (d, *J* = 6.6 Hz, 2H), 7.02 (d, *J* = 7.3 Hz, 1H), 4.00 (br s, 1H), 3.49 (d, *J* = 15.3 Hz, 1H), 3.34 – 3.24 (m, 2H), 3.16 (s, 3H), 3.08 (s, 3H), 2.90 (d, *J* = 15.2 Hz, 2H), 2.50 (ddd, *J* = 14.4, 12.9, 6.4 Hz, 1H), 2.40 – 2.33 (m, 1H), 2.29 – 2.20 (m, 1H), 1.95 – 1.86 (m, 1H), 1.26 – 1.13 (m, 1H) ppm. **^{13}C NMR** (125 MHz, $CDCl_3$) δ 212.0 (CO), 171.6 (CO), 145.4 (C), 143.1 (C), 137.9 (C), 131.4 (C), 130.1 (CH), 128.3 (CH), 128.2 (CH), 126.7 (CH), 126.6 (CH), 124.9 (CH), 85.3 (COH), 67.2 (C), 39.7 (CH_3 amide), 39.5 (CH_2), 38.0 (CH_2), 37.3 (CH_2), 35.2 (CH_3 amide), 32.4 (CH_2), 20.4 (CH_2) ppm. **HRMS** [APCI]: *m/z* calculated for $C_{23}H_{26}NO_3$ $[M + H]^+$: 364.1907, found 364.1907.

Enantioselectivity was determined by chiral HPLC analysis on a Chiralpack IE3 column at rt (Hexane:*i*PrOH = 90:10, 1 mL/min).

A: Racemic sample prepared using [Ir(COD)$_2$]BAr$^F_4$ / *rac*-DTBM-Segphos (5 mol%) in the presence of 1.25 equiv of Et_3SiH, at 130 ˚C for 3 h.

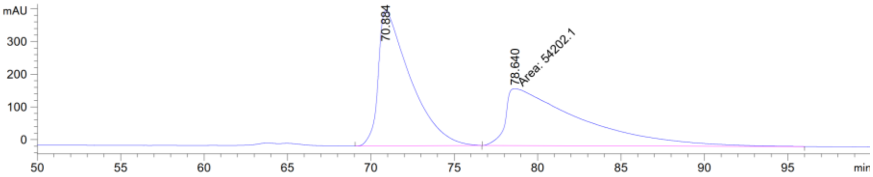

Signal 3: DAD1 C, Sig=210,4 Ref=off

Peak #	RetTime [min]	Type	Width [min]	Area [mAU*s]	Height [mAU]	Area %
1	70.884	BB	1.8464	5.52703e4	413.24179	50.4879
2	78.640	MM	5.2016	5.42021e4	173.67079	49.5121
Totals :				1.09472e5	586.91258	

B: Asymmetric sample prepared using [Ir(COD)$_2$]BAr$^F_4$ / *(R)*-DTBM-Segphos (5 mol%) in the presence of 1.25 equiv of Et_3SiH, at 130 ˚C for 3 h. (75% yield, 87:13 er).

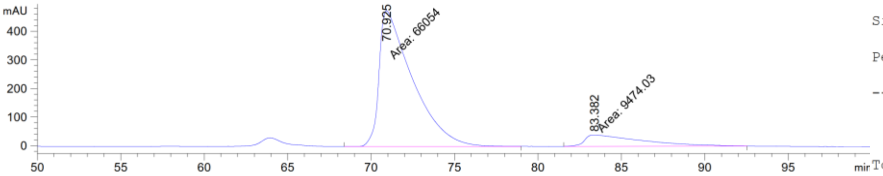

Signal 3: DAD1 C, Sig=210,4 Ref=off

Peak #	RetTime [min]	Type	Width [min]	Area [mAU*s]	Height [mAU]	Area %
1	70.925	MM	2.3184	6.60540e4	474.85971	87.4563
2	83.382	MM	3.9441	9474.02734	40.03449	12.5437
Totals :				7.55280e4	514.89420	

***(R)*-9a-Benzyl-*N,N*-dimethyl-1-oxo-2,3,9,9a-tetrahydro-1*H*-fluorene-5-carboxamide (3f)**

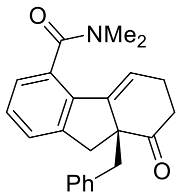

Prepared following General Procedure L from prochiral diketone **1f**. Obtained as white solid. **^{1}H NMR** (500 MHz, $CDCl_3$) δ 7.24 – 7.09 (m, 5H), 7.05 – 6.94 (m, 3H), 6.14 (d x2, *J* = 149.9, 6.6, 2.7 Hz, 1H), 3.17 – 2.75 (m, 10H), 2.54 – 2.38 (m, 1H), 2.32 – 2.13 (m, 2H), 1.88 – 1.63 (m, 2H), 1.25 – 1.15 (m, 1H) ppm. **^{13}C NMR** (126 MHz, $CDCl_3$) δ 215.44 (CO), 215.05 (CO), 170.67 (CO), 142.99 (C), 142.93 (C),

136.27 (C), 134.82 (C), 134.19 (C), 133.77 (C), 131.80 (C), 131.36 (C), 130.32 (CH), 130.02 (CH), 128.58 (CH), 128.35 (CH), 128.14 (CH), 127.02 (CH), 126.87 (CH), 126.12 (CH), 126.04 (CH), 124.78 (CH), 124.57 (CH), 122.33 (CH), 121.23 (CH), 60.46 (C), 43.90 (CH_2), 43.61 (CH_2), 38.98 (CH_2), 38.85 (CH_3 amide + CH_2), 38.60 (CH_3 amide), 36.83 (CH_2), 36.61 (CH_2), 34.86 (CH_3 amide), 34.68 (CH_3 amide), 31.72 (CH_2), 23.23 (CH_2), 22.80 (CH_2) ppm. Note: most of the signals are duplicated due to rotamerism. **HRMS** [APCI]: *m/z* calculated for $C_{23}H_{24}NO_2$ $[M + H]^+$: 346.1802, found 346.1800. **MP** = 107.2 – 108.4 °C

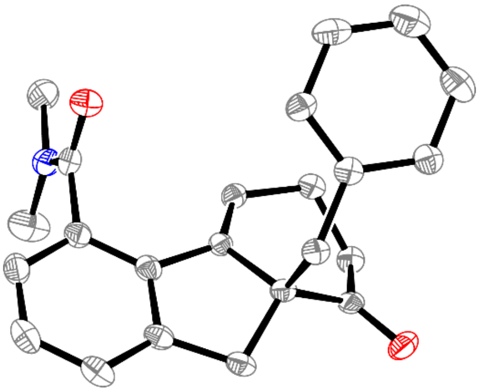

Figure S1. X-ray structure of **3f** (CCDC 2504165)

Enantioselectivity was determined by chiral HPLC analysis on a Chiralpack IE3 column at rt (Hexane:*i*PrOH = 85:15, 1 mL/min).

A: Racemic sample prepared using $[Ir(COD)_2]BAr^F_4$ / *rac*-Binap (5 mol%) at 130 ˚C.

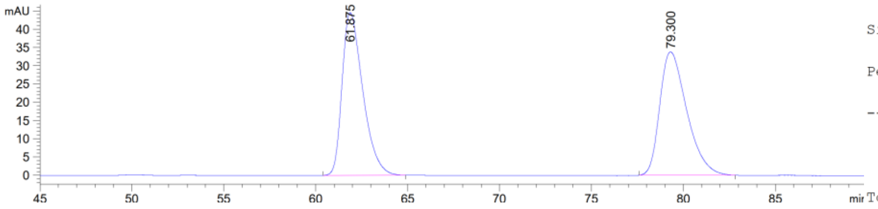

Signal 2: DAD1 B, Sig=254,4 Ref=off

Peak #	RetTime [min]	Type	Width [min]	Area [mAU*s]	Height [mAU]	Area %
1	61.875	BB	1.1522	3485.56543	44.57793	50.2446
2	79.300	BB	1.4140	3451.63477	33.78318	49.7554
Totals :				6937.20020	78.36112	

B: Asymmetric sample prepared using $[Ir(COD)_2]BAr^F_4$ / *(R)*-DTBM-Segphos (5 mol%) at 130 ˚C (79% yield, 87:13 er).

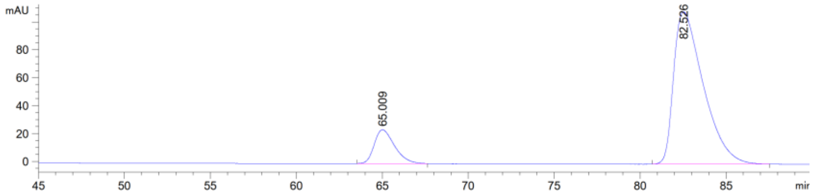

Signal 2: DAD1 B, Sig=254,4 Ref=off

Peak #	RetTime [min]	Type	Width [min]	Area [mAU*s]	Height [mAU]	Area %
1	65.009	BB	1.1586	1983.33972	24.18972	13.1489
2	82.526	BB	1.6980	1.31003e4	109.16790	86.8511
Totals :				1.50836e4	133.35762	

C: After recrystallization 97:3 er.

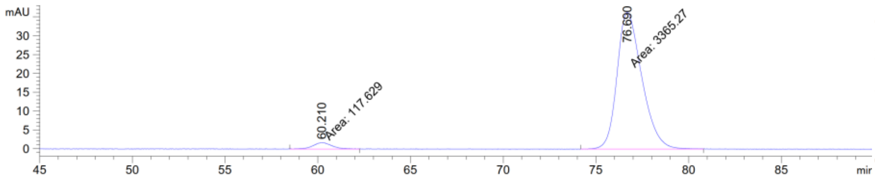

Signal 2: DAD1 B, Sig=254,4 Ref=off

Peak #	RetTime [min]	Type	Width [min]	Area [mAU*s]	Height [mAU]	Area %
1	60.210	MM	1.1625	117.62928	1.68640	3.3773
2	76.690	MM	1.5427	3365.26611	36.35717	96.6227
Totals :				3482.89539	38.04356	

(*R*)-*N,N*-dimethyl-1-oxo-9a-(3-phenylpropyl)-2,3,9,9a-tetrahydro-1*H*-fluorene-5-carboxamide (3g)

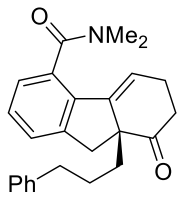

Prepared following General Procedure L from prochiral diketone **1g**. Reaction time: 24 h. Obtained as pale-yellow oil. **^{1}H NMR** (300 MHz, $CDCl_3$) 7.28 – 7.19 (m, 4H), 7.19 – 7.12 (m, 1H), 7.10 – 7.01 (m, 2H), 6.22 (br s, 1H), 3.33 (d, *J* = 16.9 Hz, 1H), 3.16 (s, 3H), 2.90 – 2.77 (m, 4H), 2.68 – 2.40 (m, 4H), 2.39 – 2.22 (m, 1H), 1.84 – 1.35 (m, 5H) ppm. **^{13}C NMR** (126 MHz, $CDCl_3$) δ 213.54 (CO), 170.74 (CO), 144.95 (C), 143.33 (C), 141.64 (C), 134.18 (C), 131.58 (C), 128.65 (CH), 128.46 (CH), 128.40 (CH), 126.03 (CH), 124.86 (CH), 120.55 (CH), 119.56 (CH), 59.37 (C), 38.35 (CH_3 amide), 37.62 (CH_2), 37.02 (CH_2), 35.97 (CH_2), 35.26 (CH_2), 34.82 (CH_3 amide), 26.36 (CH_2), 24.23 (CH_2) ppm. Note: The broad and low intensity of certain signals is presumably attributable to rotamerism. **HRMS** [APCI]: *m/z* calculated for $C_{25}H_{28}NO_2$ $[M + H]^+$: 374.2115, found 374.2123.

Enantioselectivity was determined by chiral HPLC analysis on a Chiralpack IF3 column at rt (Hexane:*i*PrOH = 85:15, 1 mL/min).

A: Racemic sample prepared using $[Ir(COD)_2]BAr^F_4$ / *rac*-Binap (5 mol%) at 130 ˚C.

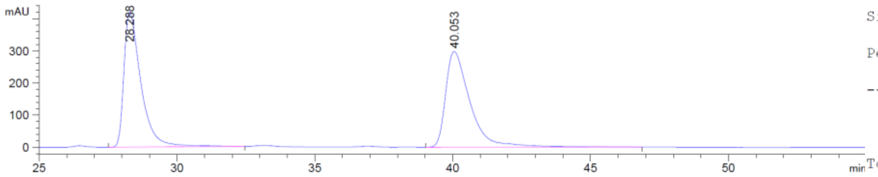

Signal 3: DAD1 C, Sig=210,4 Ref=off

Peak #	RetTime [min]	Type	Width [min]	Area [mAU*s]	Height [mAU]	Area %
1	28.288	BB	0.6455	1.82029e4	421.75827	49.7302
2	40.053	BB	0.9205	1.84003e4	297.95081	50.2698
Totals :				3.66032e4	719.70908	

B: Asymmetric sample prepared using $[Ir(COD)_2]BAr^F_4$ / *(R)*-DTBM-Segphos (5 mol%) at 130 ˚C (86% yield, 93:7 er).

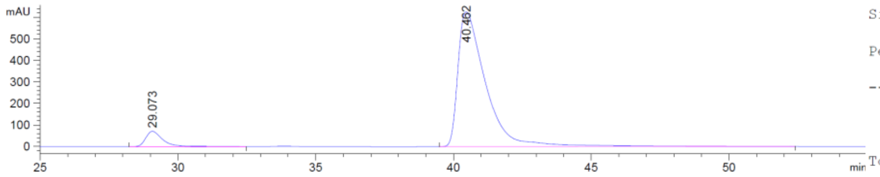

Signal 3: DAD1 C, Sig=210,4 Ref=off

Peak #	RetTime [min]	Type	Width [min]	Area [mAU*s]	Height [mAU]	Area %
1	29.073	BB	0.6434	3084.14160	71.19868	6.5526
2	40.462	BB	1.0348	4.39833e4	625.18561	93.4474
Totals :				4.70675e4	696.38428	

(*R*)-*N,N*-dimethyl-1-oxo-9a-phenyl-2,3,9,9a-tetrahydro-1*H*-fluorene-5-carboxamide (3h)

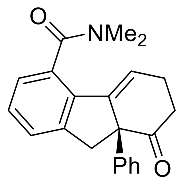

Prepared following General Procedure L from prochiral diketone **1h**. Obtained as a brown oil. **^{1}H NMR (500 MHz, $CDCl_3$):** δ 7.30 – 7.25 (m, 2H), 7.24 – 7.08 (m, 6H), 7.00 (t, *J* = 7.0 Hz, 1H), 6.38 (2 x t, *J* = 3.3 Hz, 1H), 3.93 – 3.78 (m, 1H), 3.18 (2 x s, *J* = 13.7 Hz, 3H), 3.05 – 2.94 (m, 1H), 2.90 (2 x s, *J* = 36.4 Hz, 3H), 2.45 – 2.26 (m, 3H), 2.24 – 2.15 (m, 1H) ppm. **^{13}C NMR (126 MHz, $CDCl_3$):** Rotamer A: δ 210.8 (CO), 170.8 (CO), 143.3 (C), 142.3 (C), 139.7 (C), 133.4 (C), 131.0 (C), 129.0 (CH), 128.7 (CH), 127.5 (CH), 126.6 (CH), 126.1 (CH), 124.5 (CH), 123.7 (CH), 64.6 (C), 42.3 (CH2), 38.9 (CH3 amide), 34.9 (CH3 amide), 34.8 (CH2), 24.2 (CH2) ppm. Rotamer B: δ 211.1 (CO), 170.8 (CO), 143.3 (C), 142.6 (C), 139.7 (C), 134.0 (C), 131.2 (C), 129.4 (CH), 129.1 (CH), 127.4 (CH), 126.7 (CH), 126.2 (CH), 124.4 (CH), 122.7 (CH), 64.5 (C), 42.2 (CH_2), 38.7 (CH_3 amide), 34.8 (CH_2), 34.7 (CH_3 amide), 24.5 (CH_2) ppm. Note: Many signals are duplicated due to rotamerism. **HRMS** (APCI-FIA-TOF): *m/z* calculated for $C_{22}H_{24}NO_3$ $[M + H]^+$: 350.1751, found 350.1748.

Enantioselectivity was determined by chiral HPLC analysis on a Chiralpack IE3 column at rt (Hexane:*i*PrOH = 85:15, 1 mL/min).

A: Racemic sample prepared using $[Ir(COD)_2]BAr^F{}_4$ / *rac*-Binap (10 mol%) at 130 ˚C.

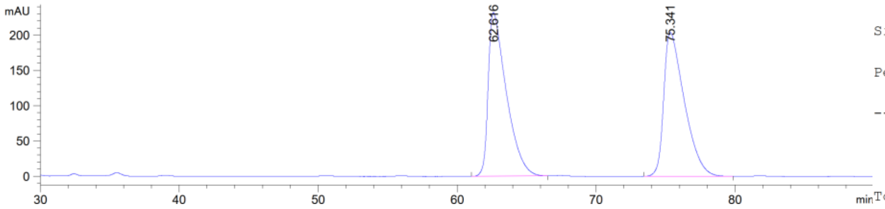

Signal 2: DAD1 B, Sig=254,4 Ref=off

Peak #	RetTime [min]	Type	Width [min]	Area [mAU*s]	Height [mAU]	Area %
1	62.616	BB	1.2801	2.10587e4	232.19760	50.0151
2	75.341	BB	1.4696	2.10460e4	204.33519	49.9849
Totals :				4.21046e4	436.53279	

B: Asymmetric sample prepared using $[Ir(COD)_2]BAr^F{}_4$ / *(R)*-Segphos (10 mol%) at 130 ˚C (97% yield, 89:11 er).

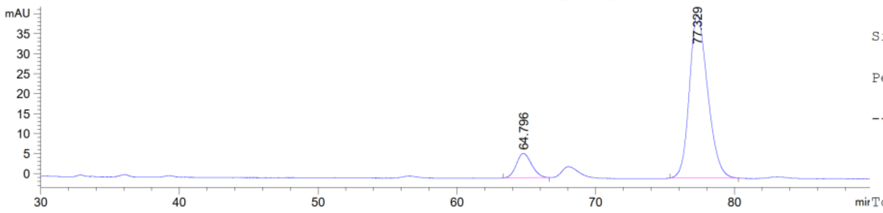

Signal 2: DAD1 B, Sig=254,4 Ref=off

Peak #	RetTime [min]	Type	Width [min]	Area [mAU*s]	Height [mAU]	Area %
1	64.796	BB	0.9249	466.96396	6.08800	11.0777
2	77.329	BB	1.2104	3748.40063	40.93027	88.9223
Totals :				4215.36459	47.01827	

***(R)-N,N*,3,3,9a-Pentamethyl-1-oxo-2,3,9,9a-tetrahydro-1*H*-fluorene-5-carboxamide (3i)**

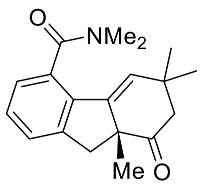

Prepared following General Procedure L from prochiral diketone **1i**. Obtained as white solid. **^{1}H NMR** (300 MHz, $CDCl_3$) δ 7.33 – 7.20 (m, 2H), 7.19 – 7.08 (m, 1H), 5.78 (s, 1H), 3.24 (d, *J* = 15.9 Hz, 1H), 3.15 (s, 3H), 2.94 – 2.77 (m, 4H), 2.68 (d, *J* = 15.8 Hz, 1H), 2.28 (d, *J* = 14.2 Hz, 1H), 1.39 (s, 3H), 1.27 (s, 3H), 1.08 (s, 3H) ppm. **^{13}C NMR** (75 MHz, $CDCl_3$) δ 212.65 (CO), 170.54 (CO), 142.43 (C), 134.57 (C), 131.79 (C), 131.10 (CH), 128.61 (CH), 126.60 (CH), 125.30 (CH), 54.65 (C), 49.85 (CH_2), 39.52 (CH_2), 39.24 (C), 38.20 (CH_3), 34.83 (CH_3), 30.92 (CH_3 amide), 30.70 (CH_3), 27.78 (CH_3 amide) ppm. **HRMS** [APCI]: *m/z* calculated for $C_{19}H_{24}NO_2$ $[M + H]^+$: 298.1802, found 298.1807. **MP** = 154.3 – 155.7 °C

Enantioselectivity was determined by chiral HPLC analysis on a Chiralpack IG3 column at rt (Hexane:*i*PrOH = 95:5, 1 mL/min).

A: Racemic sample prepared using $[Ir(COD)_2]BAr^F{}_4$ / *rac*-Binap (5 mol%) at 130 ˚C.

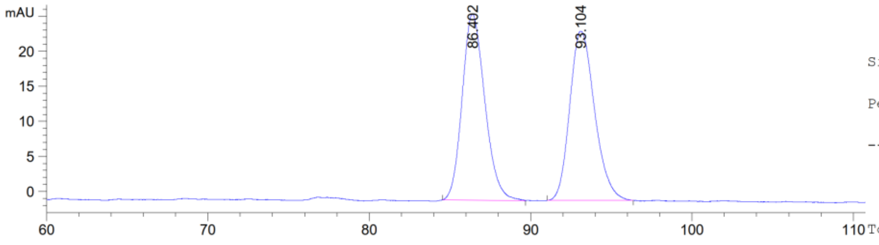

Signal 1: DAD1 A, Sig=220,4 Ref=off

Peak #	RetTime [min]	Type	Width [min]	Area [mAU*s]	Height [mAU]	Area %
1	86.402	BB	1.1796	2592.83154	26.54327	50.0611
2	93.104	BB	1.2658	2586.50684	24.14364	49.9389
Totals :				5179.33838	50.68691	

B: Asymmetric sample prepared using $[Ir(COD)_2]BAr^F{}_4$ / *(R)*-DTBM-Segphos (5 mol%) at 130 ˚C (69% yield, 94:6 er).

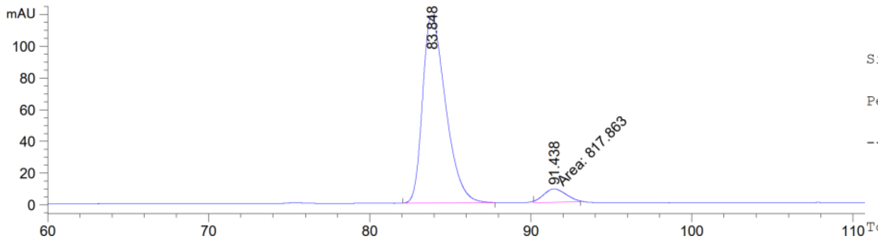

Signal 1: DAD1 A, Sig=220,4 Ref=off

Peak #	RetTime [min]	Type	Width [min]	Area [mAU*s]	Height [mAU]	Area %
1	83.848	BB	1.4196	1.17279e4	118.40192	93.4810
2	91.438	MM	1.6126	817.86310	8.45300	6.5190
Totals :				1.25458e4	126.85491	

(R)-N,N,3,3-Tetramethyl-1-oxo-9a-propyl-2,3,9,9a-tetrahydro-1H-fluorene-5-carboxamide (3j)

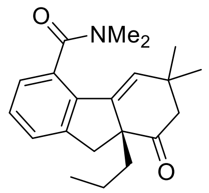

Prepared following General Procedure L from prochiral diketone **1j**. Obtained as white solid. **^{1}H NMR** (300 MHz, $CDCl_3$) δ 7.36 – 7.05 (m, 3H), 5.71 (br s, 1H), 3.20 (d, *J* = 16.5 Hz, 1H), 3.13 (s, 3H), 2.89 – 2.69 (m, 5H), 2.25 (dd, *J* = 13.5, 1.5 Hz, 1H), 1.73 – 1.60 (m, 2H), 1.43 – 1.31 (m, 1H), 1.28 (d, *J* = 1.8 Hz, 3H), 1.12 (d, *J* = 6.9 Hz, 1H), 1.04 (d, *J* = 1.8 Hz, 3H), 0.83 (dd, *J* = 8.1, 6.3 Hz, 3H) ppm. **^{13}C NMR** (75 MHz, $CDCl_3$) δ 211.41 (CO), 170.59 (CO), 142.62 (C), 134.83 (C), 131.65 (C), 130.97 (CH), 128.56 (CH), 126.50 (CH), 125.22 (CH), 59.37 (C), 49.62 (CH_2), 43.56 (CH_2), 40.18 (C), 38.32 (CH_3), 35.62 (CH_2), 34.85 (CH_3 amide), 31.11 (CH_3 + CH_3 amide), 18.33 (CH_2), 14.49 (CH_3) ppm. **HRMS** [APCI]: *m/z* calculated for $C_{21}H_{28}NO_2$ $[M + H]^+$: 326.2115, found 326.2113. **MP** = 122.4 – 124.1 °C

Enantioselectivity was determined by chiral HPLC analysis on a Chiralpack IG3 column at rt (Hexane:*i*PrOH = 95:5, 1 mL/min).

A: Racemic sample prepared using $[Ir(COD)_2]BAr^F{}_4$ / *rac*-Binap (10 mol%) at 130 ˚C.

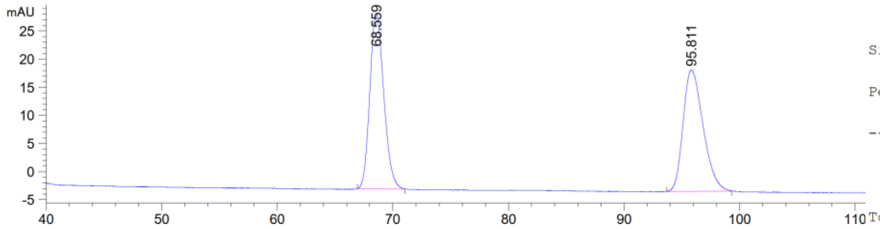

Signal 2: DAD1 B, Sig=254,4 Ref=off

Peak #	RetTime [min]	Type	Width [min]	Area [mAU*s]	Height [mAU]	Area %
1	68.559	BB	1.1949	2610.06348	31.17161	50.1357
2	95.811	BB	1.4158	2595.93213	21.54314	49.8643
Totals :				5205.99561	52.71475	

B: Asymmetric sample prepared using $[Ir(COD)_2]BAr^F{}_4$ / *(R)*-DTBM-Segphos (10 mol%) at 130 ˚C (82% yield, 96:4 er).

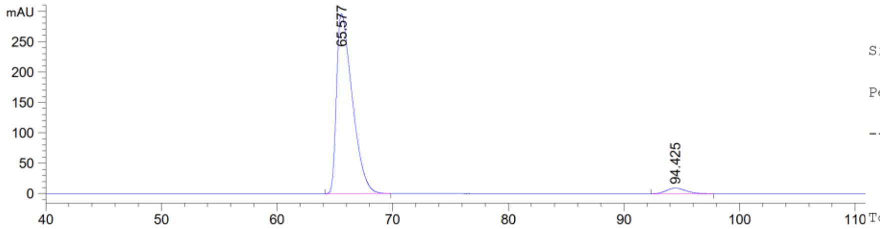

Signal 2: DAD1 B, Sig=254,4 Ref=off

Peak #	RetTime [min]	Type	Width [min]	Area [mAU*s]	Height [mAU]	Area %
1	65.577	BB	1.3565	2.79504e4	295.77328	96.1442
2	94.425	BB	1.4128	1120.93506	9.38970	3.8558
Totals :				2.90714e4	305.16298	

(4a'R,9a'R)-4a'-hydroxy-N,N,9a'-trimethyl-1'-oxo-1',2',4',4a',9',9a'-hexahydrospiro[cyclopentane-1,3'-fluorene]-5'-carboxamide (2k)

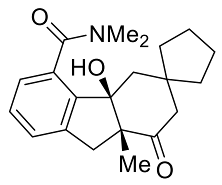

Prepared following General Procedure L from prochiral diketone **1k** in the presence of 1.25 equiv of Et_3SiH. Reaction time: 3 h. Obtained as pale-yellow oil. **^{1}H NMR** (500 MHz, $CDCl_3$) δ ^{1}H NMR (500 MHz, $CDCl_3$) δ 7.27 – 7.25 (m, 1H), 7.21 (t, *J* = 7.5 Hz, 1H), 7.09 (d, *J* = 6.3 Hz, 1H), 4.20 (br s, 1H), 3.47 (d, *J* = 15.8 Hz, 1H), 3.15 (s, 3H), 3.03 (s, 3H), 2.70 (d, *J* = 15.8 Hz, 1H), 2.58 (d, *J* = 13.4 Hz, 1H), 2.31 (d, *J* = 14.1 Hz, 1H), 2.26 (d, *J* = 13.8 Hz, 1H), 2.10 (d, *J* = 14.7 Hz, 1H), 1.71 – 1.56 (m, 6H), 1.58 – 1.49 (m, 2H), 1.26 (s, 3H) ppm. **^{13}C NMR** (126 MHz, $CDCl_3$) δ 214.0 (CO), 171.6 (CO), 146.0 (C), 142.2 (C), 131.2 (C), 127.4 (CH), 127.0 (CH), 125.8 (CH), 86.7 (COH), 59.9 (C), 48.0 (CH_2), 46.5 (CH_2), 44.3 (CH_2), 40.6 (CH_2), 40.0 (CH_3 amide), 39.9 (CH_2), 39.3 (CH_2), 35.5 (CH_3 amide), 24.0 (CH_2), 23.5 (CH_2), 18.4 (CH_3) ppm. **HRMS** [APCI]: *m/z* calculated for $C_{21}H_{28}NO_3$ $[M + H]^+$: 342.2064, found 342.2077.

Enantioselectivity was determined by chiral HPLC analysis on a Chiralpack IG3 column at rt (Hexane:*i*PrOH = 95:5, 1 mL/min).

A: Racemic sample prepared using [Ir(COD)$_2$]BAr$^F{}_4$ / *rac*-DTBM-Segphos (10 mol%) in the presence of 1.25 equiv of Et_3SiH, at 130 ˚C for 3 h.

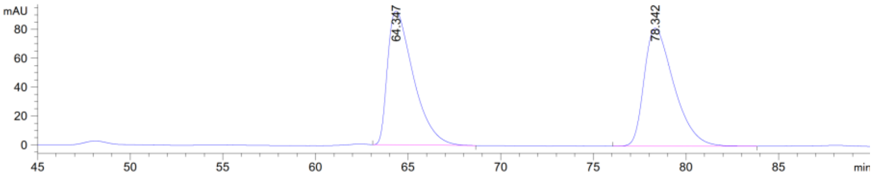

Signal 1: DAD1 A, Sig=220,4 Ref=off

Peak #	RetTime [min]	Type	Width [min]	Area [mAU*s]	Height [mAU]	Area %
1	64.347	BB	1.4272	8984.66211	92.66527	49.5553
2	78.342	BB	1.6565	9145.93066	81.67181	50.4447
Totals :				1.81306e4	174.33707	

B: Asymmetric sample prepared using [Ir(COD)$_2$]BAr$^F{}_4$ / *(R)*-DTBM-Segphos (10 mol%) in the presence of 1.25 equiv of Et_3SiH, at 130 ˚C for 3 h. (74% yield, 93:7 er).

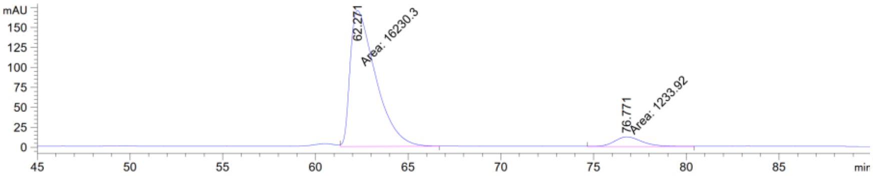

Signal 1: DAD1 A, Sig=220,4 Ref=off

Peak #	RetTime [min]	Type	Width [min]	Area [mAU*s]	Height [mAU]	Area %
1	62.271	FM	1.5919	1.62303e4	169.92757	92.9346
2	76.771	MM	1.7251	1233.91614	11.92098	7.0654
Totals :				1.74642e4	181.84855	

(*R*)-*N,N*,9a'-trimethyl-1'-oxo-1',2',9',9a'-tetrahydrospiro[cyclopentane-1,3'-fluorene]-5'-carboxamide (3k)

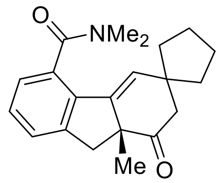

Prepared following General Procedure L from prochiral diketone **1k**. Obtained as white solid. **^{1}H NMR** (300 MHz, $CDCl_3$) δ 7.31 – 7.17 (m, 2H), 7.17 – 7.09 (m, 1H), 5.87 (br s, 1H), 3.21 (d, *J* = 15.2 Hz, 1H), 3.11 (s, 3H), 2.82 (s, 3H), 2.77 – 2.63 (m, 2H), 2.34 (d, *J* = 15.0 Hz, 1H), 1.72 (br s, 6H), 1.55 (br s, 2H), 1.36 (s, 3H) ppm. **^{13}C NMR** (75 MHz, $CDCl_3$) δ 213.30 (CO), 170.60 (CO), 142.99 (C), 142.28 (C), 134.52 (C), 131.72 (C), 130.36 (CH), 128.57 (CH), 126.55 (CH), 125.31 (CH), 54.78 (C), 48.99 (C), 48.05 (CH_2), 41.67 (CH_2), 40.84 (CH_2), 39.91 (CH_2), 38.18 (CH_3 amide), 34.81 (CH_3 amide), 27.40 (CH_3), 24.58 (CH_2), 23.92 (CH_2) ppm. **HRMS** [APCI]: *m/z* calculated for $C_{21}H_{26}NO_2$ [M + H]$^+$: 324.1958, found 324.1961. **MP** = 130.8 – 132.5 °C

Enantioselectivity was determined by chiral HPLC analysis on a Chiralpack IG3 column at rt (Hexane:*i*PrOH = 95:5, 1 mL/min).

A: Racemic sample prepared using [Ir(COD)$_2$]BAr$^F{}_4$ / *rac*-Binap (5 mol%) at 130 ˚C.

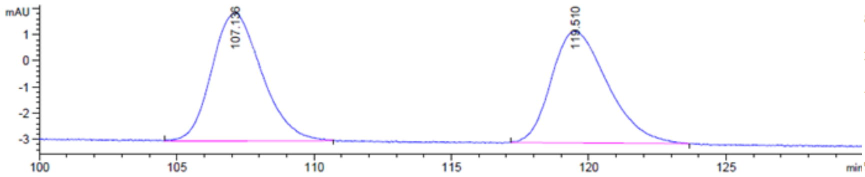

Signal 4: DAD1 D, Sig=300,4 Ref=off

Peak #	RetTime [min]	Type	Width [min]	Area [mAU*s]	Height [mAU]	Area %
1	107.136	MM	2.0991	619.61041	4.91974	50.5186
2	119.510	MM	2.3726	606.88977	4.26327	49.4814
Totals :				1226.50018	9.18300	

B: Asymmetric sample prepared using [Ir(COD)$_2$]BAr$^F{}_4$ / *(R)*-DTBM-Segphos (5 mol%) at 130 ˚C (73% yield, 94:6 er).

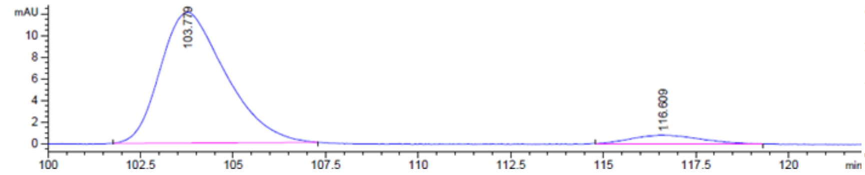

Signal 4: DAD1 D, Sig=300,4 Ref=off

Peak #	RetTime [min]	Type	Width [min]	Area [mAU*s]	Height [mAU]	Area %
1	103.779	BB	1.4468	1484.73486	12.08981	94.2294
2	116.609	MM	2.1558	107.82604	8.33625e-1	5.7706
Totals :				1592.56091	12.92343	

(R)-N,N,9a'-Trimethyl-1'-oxo-1',2',9',9a'-tetrahydrospiro[cyclohexane-1,3'-fluorene]-5'-carboxamide (3l)

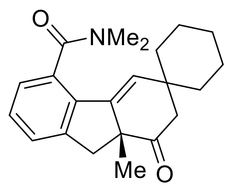

Prepared following General Procedure L from prochiral diketone **1l**. Reaction time: 24 h. Obtained as white solid. ^{1}**H NMR** (300 MHz, $CDCl_3$) δ 7.34 – 7.20 (m, 2H), 7.17 (br m, 1H), 6.01 (br s, 1H), 3.28 – 3.07 (m, 4H), 2.85 (s, 3H), 2.69 (d, J = 15.9 Hz, 2H), 2.45 (d, J = 14.8 Hz, 1H), 1.76 – 1.40 (m, 10H), 1.39 (s, 3H) ppm. ^{13}**C NMR** (75 MHz, $CDCl_3$) δ 213.29 (CO), 170.59 (CO), 143.04 (C), 142.33 (C), 134.78 (C), 131.74 (C), 130.12 (CH), 128.62 (CH), 126.56 (CH), 125.39 (CH), 55.10 (C), 47.86 (CH_2), 41.79 (C), 39.99 (CH_2), 39.76 (CH_2), 38.18 (CH_3 amide), 34.92 (CH_3 amide), 27.63 (CH_3), 25.67 (CH_2), 22.19 (CH_2), 21.94 (CH_2) ppm. **HRMS** [APCI]: m/z calculated for $C_{22}H_{28}NO_2$ $[M + H]^+$: 338.2115, found 338.2112. **MP** = 127.3 – 128.2 °C

Enantioselectivity was determined by chiral HPLC analysis on a Chiralpack IG3 column at rt (Hexane:*i*PrOH = 95:5, 1 mL/min).

A: Racemic sample prepared using $[Ir(COD)_2]BAr^F{}_4$ / *rac*-Binap (10 mol%) at 130 ˚C.

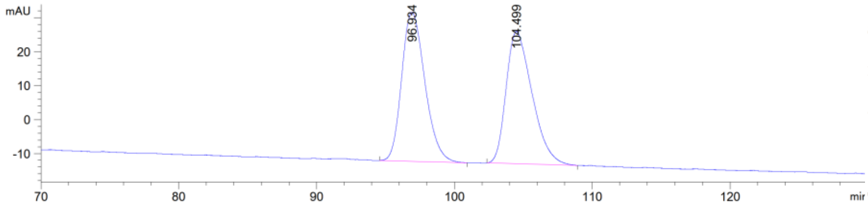

Signal 1: DAD1 A, Sig=220,4 Ref=off

Peak #	RetTime [min]	Type	Width [min]	Area [mAU*s]	Height [mAU]	Area %
1	96.934	BB	1.4283	5141.37842	43.90170	50.1871
2	104.499	BB	1.5709	5103.04639	38.99007	49.8129
Totals :				1.02444e4	82.89177	

B: Asymmetric sample prepared using $[Ir(COD)_2]BAr^F{}_4$ / *(R)*-DTBM-Segphos (10 mol%) at 130 ˚C (99% yield, 94:6 er).

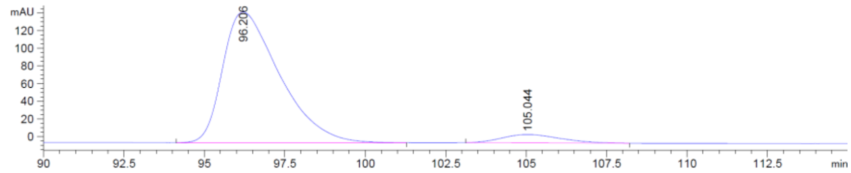

Signal 1: DAD1 A, Sig=220,4 Ref=off

Peak #	RetTime [min]	Type	Width [min]	Area [mAU*s]	Height [mAU]	Area %
1	96.206	BB	1.7405	1.82147e4	148.14220	93.9355
2	105.044	BB	1.4678	1175.95276	9.38545	6.0645
Totals :				1.93907e4	157.52765	

(4b*R*,9a*R*)-4b-Hydroxy-*N,N*,9a-trimethyl-9-oxo-4b,5,6,7,8,9,9a,10-octahydrobenzo[*a*]azulene-4-carboxamide (2m)

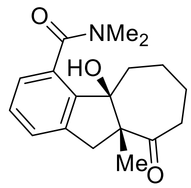

Prepared following General Procedure L from prochiral diketone **1m** in the presence of 1.25 equiv of Et_3SiH. Reaction time: 6 h. Obtained as pale-yellow solid. ^{1}**H NMR** (500 MHz, $CDCl_3$) δ 7.26 – 7.19 (m, 2H), 7.12 – 7.07 (m, 1H), 4.93 (br s, 1H), 3.35 (d, J = 16.6 Hz, 1H), 3.14 (s, 3H), 3.13 – 3.07 (m, 1H), 2.96 (s, 3H), 2.73 (d, J = 16.7 Hz, 1H), 2.32 – 2.26 (m, 1H), 2.24 – 2.14 (m, 1H), 1.96 – 1.86 (m, 2H), 1.64 – 1.57 (m, 1H), 1.48 – 1.28 (m, 2H), 1.26 (s, 3H) ppm. ^{13}**C NMR** (126 MHz, $CDCl_3$) δ 216.8 (CO), 172.2 (CO), 146.4 (C), 140.3 (C), 131.6 (C), 127.5 (CH), 126.9 (CH), 126.4 (CH), 81.8 (COH), 64.5 (C), 40.1 (CH_3 amide), 39.9 (CH_2), 39.7 (CH_2), 39.0 (CH_2), 35.6 (CH_3 amide), 27.8 (CH_2), 23.2 (CH_2), 21.6 (CH_3) ppm. **HRMS** [APCI]: m/z calculated for $C_{18}H_{24}NO_3$ $[M + H]^+$: 302.1751, found 302.1754. **MP** = 164.9 – 167.2 °C

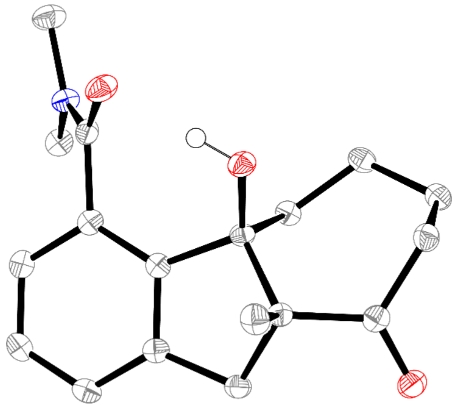

Figure S2. X-ray structure of **2m** (CCDC 2504171)

Enantioselectivity was determined by chiral HPLC analysis on a Chiralpack IF3 column at rt (Hexane:*i*PrOH = 95:5, 1 mL/min).

A: Racemic sample prepared using $[Ir(COD)_2]BAr^F{}_4$ / *rac*-DTBM-Segphos (5 mol%) in the presence of 1.25 equiv of Et_3SiH, at 130 ˚C for 3 h.

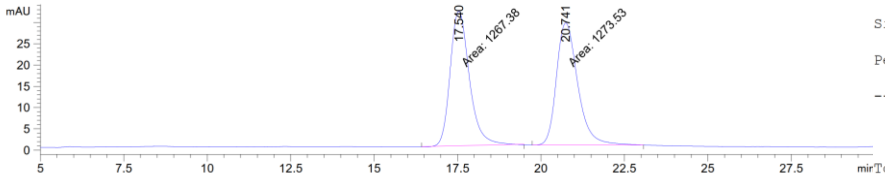

Signal 2: DAD1 B, Sig=254,4 Ref=off

Peak #	RetTime [min]	Type	Width [min]	Area [mAU*s]	Height [mAU]	Area %
1	17.540	MM	0.6679	1267.38013	31.62527	49.8790
2	20.741	MM	0.7391	1273.53064	28.71770	50.1210
Totals :				2540.91077	60.34297	

B: Asymmetric sample prepared using $[Ir(COD)_2]BAr^F{}_4$ / *(R)*-DTBM-Segphos (5 mol%) in the presence of 1.25 equiv of Et_3SiH, at 130 ˚C for 6 h. (58% yield, 94:6 er).

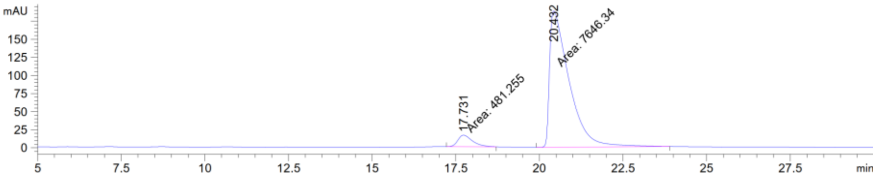

Signal 2: DAD1 B, Sig=254,4 Ref=off

Peak #	RetTime [min]	Type	Width [min]	Area [mAU*s]	Height [mAU]	Area %
1	17.731	MM	0.5036	481.25504	15.92815	5.9213
2	20.432	MM	0.6767	7646.33545	188.32312	94.0787
Totals :				8127.59048	204.25127	

C: After recrystallization from a sample with 94:6 er: 99:1 er

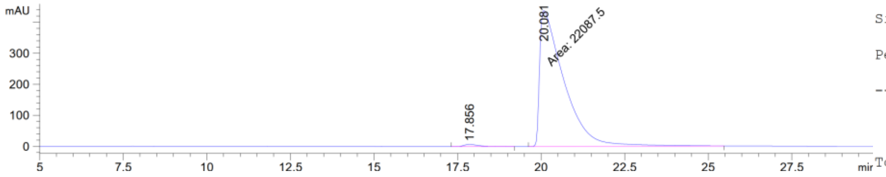

Signal 2: DAD1 B, Sig=254,4 Ref=off

Peak #	RetTime [min]	Type	Width [min]	Area [mAU*s]	Height [mAU]	Area %
1	17.856	BB	0.4153	191.29868	6.94710	0.8587
2	20.081	MM	0.8405	2.20875e4	437.97992	99.1413
Totals :				2.22788e4	444.92702	

(R)-N,N,9a-Trimethyl-9-oxo-6,7,8,9,9a,10-hexahydrobenzo[*a*]azulene-4-carboxamide (3m)

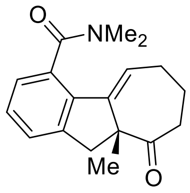

Prepared following General Procedure L from prochiral diketone **1m**. Reaction temperature: 140 ˚C. Obtained as colorless oil. **^{1}H NMR** (500 MHz, $CDCl_3$) δ 7.21 – 7.19 (m, 2H), 7.05 (t, J = 4.4 Hz, 1H), 6.07 (t, J = 7.3 Hz, 1H), 3.19 (d, J = 16.1 Hz, 1H), 3.06 (s, 3H), 2.94 (td, J = 11.0, 8.1 Hz, 1H), 2.69 (s, 3H), 2.57 (d, J = 16.1 Hz, 1H), 2.34 – 2.28 (m, 1H), 2.24 – 2.15 (m, 2H), 2.03 – 1.95 (m, 1H), 1.81 – 1.71 (m, 1H), 1.27 (s, 3H) ppm. **^{13}C NMR** (126 MHz, $CDCl_3$) δ 215.06 (CO), 171.01 (CO), 147.51 (C), 142.64 (C), 135.29 (C), 131.80 (C), 128.54 (CH), 126.17 (CH), 125.40 (CH), 120.84 (CH), 59.63 (C), 42.72 (CH_2), 38.01 (CH_3 amide), 37.80

(CH_2), 34.94 (CH_3 amide), 25.10 (CH_2), 24.56 (CH_2), 21.56 (CH_3) ppm. Note: two rotamers are observed by NMR, only the major one was assigned for simplicity. **HRMS** [APCI]: *m/z* calculated for $C_{18}H_{22}NO_2$ $[M + H]^+$: 284.1645, found 284.1649.

Enantioselectivity was determined by chiral HPLC analysis on a Chiralpack IF3 column at rt (Hexane:*i*PrOH = 85:15, 1 mL/min).

A: Racemic sample prepared using $[Ir(COD)_2]BAr^F{}_4$ / *rac*-Binap (5 mol%) at 140 ˚C.

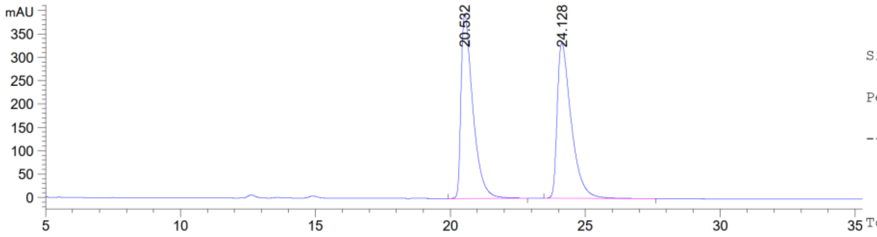

Signal 1: DAD1 A, Sig=220,4 Ref=off

Peak #	RetTime [min]	Type	Width [min]	Area [mAU*s]	Height [mAU]	Area %
1	20.532	BB	0.4516	1.19683e4	394.91968	49.9386
2	24.128	BB	0.5392	1.19977e4	332.31760	50.0614
Totals :				2.39660e4	727.23727	

B: Asymmetric sample prepared using $[Ir(COD)_2]BAr^F{}_4$ / *(R)*-DTBM-Segphos (5 mol%) at 140 ˚C (81% yield, 93:7 er).

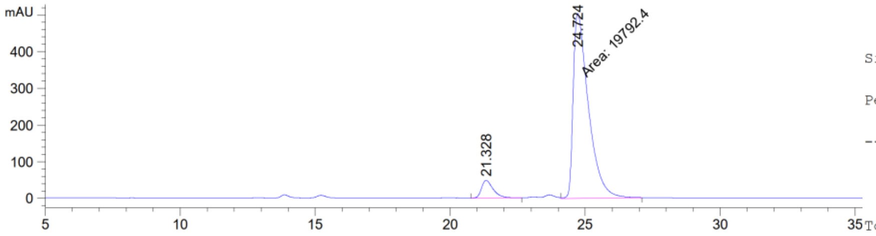

Signal 1: DAD1 A, Sig=220,4 Ref=off

Peak #	RetTime [min]	Type	Width [min]	Area [mAU*s]	Height [mAU]	Area %
1	21.328	BB	0.4583	1445.44751	47.86138	6.8060
2	24.724	MM	0.6535	1.97924e4	504.76437	93.1940
Totals :				2.12379e4	552.62576	

(*R*)-8-methoxy-*N,N*,9a-trimethyl-1-oxo-2,3,9,9a-tetrahydro-1*H*-fluorene-5-carboxamide (3n)

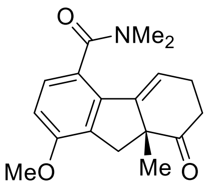

Prepared following General Procedure L from prochiral diketone **1n**. Obtained as yellow oil. **^{1}H NMR** (300 MHz, $CDCl_3$) δ 7.07 (d, *J* = 8.4 Hz, 1H), 6.73 (d, *J* = 8.2 Hz, 1H), 6.13 (br s, 1H), 3.82 (s, 3H), 3.19 (d, *J* = 17.6 Hz, 1H), 3.12 (s, 3H), 2.82 (s, 3H), 2.77 – 2.45 (m, 4H), 2.39 – 2.22 (m, 1H), 1.29 (s, 3H) ppm. **^{13}C NMR** (75 MHz, $CDCl_3$) δ 214.30 (CO), 170.91 (CO), 157.15 (C), 146.14 (C), 135.52 (C), 126.89 (CH), 124.40 (C), 119.59 (CH), 109.73 (CH), 55.50 (CH_3), 55.03 (C), 38.44 (CH_3 amide), 37.02 (CH_2), 35.37 (CH_2), 34.91 (CH_3 amide), 24.65 (CH_3), 23.97 (CH_2) ppm. **HRMS** [APCI]: *m/z* calculated for $C_{18}H_{22}NO_3$ $[M + H]^+$: 300.1594, found 300.1590.

Enantioselectivity was determined by chiral HPLC analysis on a Chiralpack IG3 column at rt (Hexane:*i*PrOH = 85:15, 1 mL/min).

A: Racemic sample prepared using $[Ir(COD)_2]BAr^F{}_4$ / *rac*-Binap (5 mol%) at 130 ˚C.

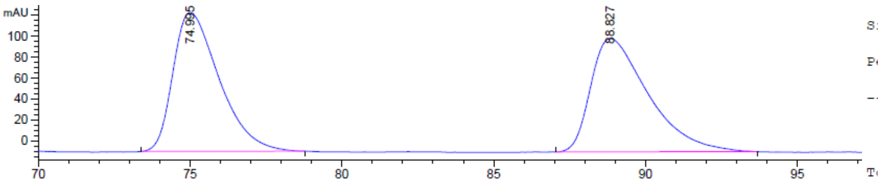

Signal 3: DAD1 C, Sig=210,4 Ref=off

Peak #	RetTime [min]	Type	Width [min]	Area [mAU*s]	Height [mAU]	Area %
1	74.995	BB	1.3352	1.39948e4	132.60989	49.5901
2	88.827	BB	1.5704	1.42261e4	108.16399	50.4099
Totals :				2.82209e4	240.77389	

B: Asymmetric sample prepared using $[Ir(COD)_2]BAr^F_4$ / *(R)*-DTBM-Segphos (10 mol%) at 130 ˚C (91% yield, 88:12 er).

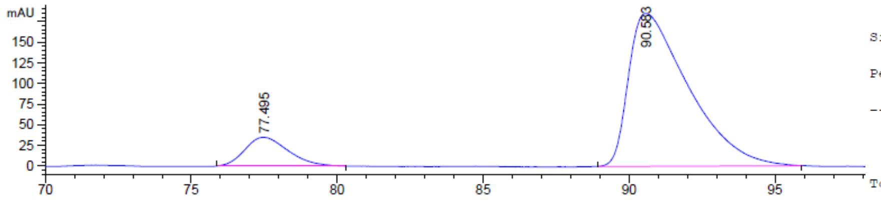

Signal 3: DAD1 C, Sig=210,4 Ref=off

Peak #	RetTime [min]	Type	Width [min]	Area [mAU*s]	Height [mAU]	Area %
1	77.495	BB	1.2098	3589.18799	34.84321	11.9701
2	90.583	BB	1.6855	2.63954e4	185.12257	88.0299
Totals :				2.99846e4	219.96579	

(*R*)-*N,N*,9a-trimethyl-1-oxo-7-phenyl-2,3,9,9a-tetrahydro-1*H*-fluorene-5-carboxamide (3o)

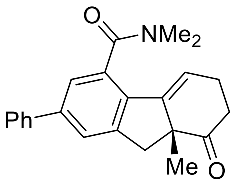

Prepared following General Procedure L from prochiral diketone **1o**. Obtained as white foam. **^{1}H NMR** (300 MHz, $CDCl_3$) δ 7.67 – 7.55 (m, 2H), 7.50 (s, 1H), 7.46 – 7.39 (m, 2H), 7.38 – 7.28 (m, 2H), 6.15 (br s, 1H), 3.62 – 3.31 (m, 1H), 3.18 (s, 3H), 2.87 (s, 3H), 2.86 – 2.51 (m, 4H), 2.44 – 2.28 (m, 1H), 1.35 (s, 3H) ppm. **^{13}C NMR** (75 MHz, $CDCl_3$) δ 214.39 (CO), 170.63 (CO), 145.38 (C), 143.72 (C), 141.92 (C), 140.29 (C), 132.69 (C), 132.23 (C), 128.98 (CH), 127.82 (CH), 127.16 (CH), 124.86 (CH), 124.06 (CH), 119.81 (CH), 55.35 (C), 40.33 (CH_2), 38.39 (CH_3 amide), 35.48 (CH_2), 34.89 (CH_3 amide), 24.71 (CH_3), 24.14 (CH_2) ppm. **HRMS** [APCI]: *m/z* calculated for $C_{23}H_{24}NO_2$ $[M + H]^+$: 346.1802, found 346.1804.

Enantioselectivity was determined by chiral HPLC analysis on a Chiralpack IF3 column at rt (Hexane:*i*PrOH = 85:15, 1 mL/min).

A: Racemic sample prepared using $[Ir(COD)_2]BAr^F_4$ / *rac*-Binap (5 mol%) at 130 ˚C.

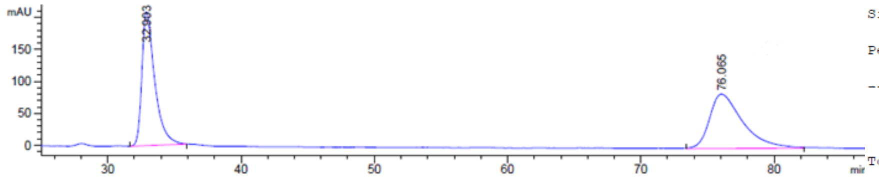

Signal 3: DAD1 C, Sig=210,4 Ref=off

Peak #	RetTime [min]	Type	Width [min]	Area [mAU*s]	Height [mAU]	Area %
1	32.903	BB	0.9639	1.40359e4	209.41690	48.5705
2	76.065	MM	2.9027	1.48621e4	85.33538	51.4295
Totals :				2.88981e4	294.75228	

B: Asymmetric sample prepared using $[Ir(COD)_2]BAr^F_4$ / *(R)*-DTBM-Segphos (5 mol%) at 130 ˚C (87% yield, 87:13 er).

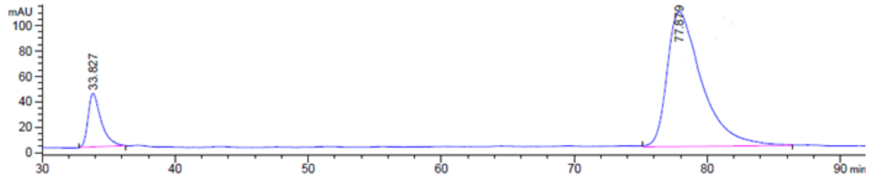

Signal 3: DAD1 C, Sig=210,4 Ref=off

Peak #	RetTime [min]	Type	Width [min]	Area [mAU*s]	Height [mAU]	Area %
1	33.827	BB	0.8760	2867.48511	42.13193	12.9860
2	77.879	MM	3.0243	1.92138e4	105.88760	87.0140
Totals :				2.20813e4	148.01953	

(4a*R*,9b*R*)-9b-hydroxy-*N,N*,4a-trimethyl-4-oxo-1,3,4,4a,5,9b-hexahydroindeno[1,2-*c*]pyran-9-carboxamide (2p)

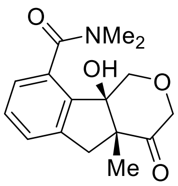

Prepared following General Procedure L from prochiral diketone **1p**. Obtained as white solid. **^{1}H NMR** (300 MHz, $CDCl_3$) δ 7.33 – 7.25 (m, 2H), 7.17 – 7.08 (m, 1H), 4.22 – 3.94 (m, 5H), 3.56 (d, *J* = 16.1 Hz, 1H), 3.17 (s, 3H), 3.05 (s, 3H), 2.88 (d, *J* = 16.2 Hz, 1H), 1.40 (s, 3H) ppm. **^{13}C NMR** (75 MHz, $CDCl_3$) δ 211.71 (CO), 171.59 (CO), 143.31 (C), 141.97 (C), 132.63 (C), 128.54 (CH), 126.52 (CH), 125.34 (CH), 82.28 (COH), 72.79 (CH_2), 71.99 (CH_2), 59.44 (C), 40.71 (CH_2), 39.85 (CH_3 amide), 35.33 (CH_3 amide), 17.75 (CH_3) ppm. **HRMS** [APCI]: *m/z* calculated for $C_{16}H_{20}NO_3$ $[M + H]^+$: 274.1438, found 274.1439. **MP** = 147.6 – 148.9 °C

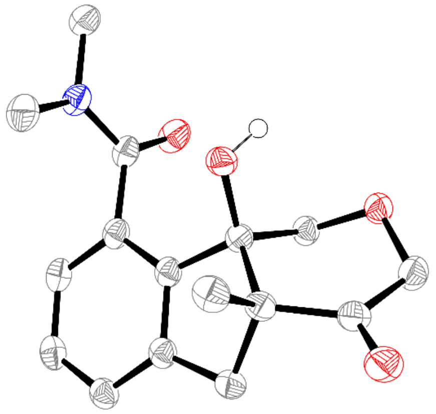

Figure S3. X-ray structure of **2p** (CCDC 2488404)

Enantioselectivity was determined by chiral HPLC analysis on a Chiralpack IF3 column at rt (Hexane:*i*PrOH = 95:5, 1 mL/min).

A: Racemic sample prepared using [Ir(COD)$_2$]BAr^F$_4$ / *rac*-Binap (5 mol%) at 130 ˚C.

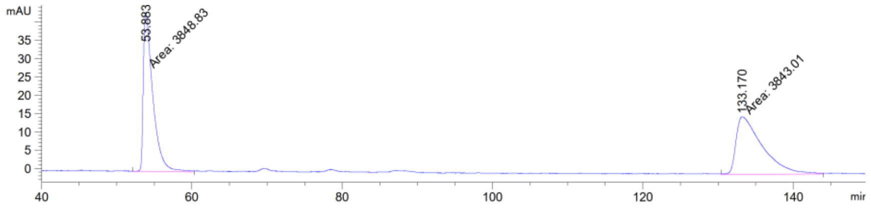

Signal 1: DAD1 A, Sig=220,4 Ref=off

Peak #	RetTime [min]	Type	Width [min]	Area [mAU*s]	Height [mAU]	Area %
1	53.883	MM	1.4776	3848.82739	43.41310	50.0378
2	133.170	MM	4.0703	3843.01343	15.73593	49.9622
Totals :				7691.84082	59.14903	

B: Asymmetric sample prepared using [Ir(COD)$_2$]BAr^F$_4$ / *(R)*-DTBM-Segphos (5 mol%) at 130 ˚C (42% yield, 90:10 er).

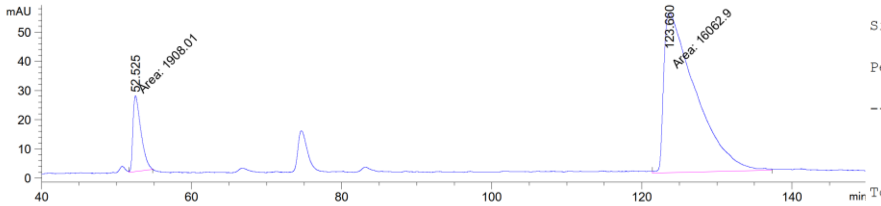

Signal 1: DAD1 A, Sig=220,4 Ref=off

Peak #	RetTime [min]	Type	Width [min]	Area [mAU*s]	Height [mAU]	Area %
1	52.525	MM	1.2315	1908.00769	25.82290	10.6172
2	123.660	MM	4.8944	1.60629e4	54.69849	89.3828
Totals :				1.79710e4	80.52139	

C: Asymmetric sample prepared using [Ir(COD)$_2$]BAr^F$_4$ / *(R)*-DTBM-Segphos (5 mol%) in the presence of 1.25 equiv of Et_3SiH at 130 ˚C, reaction time: 3 h (89% yield, 93:7 er).

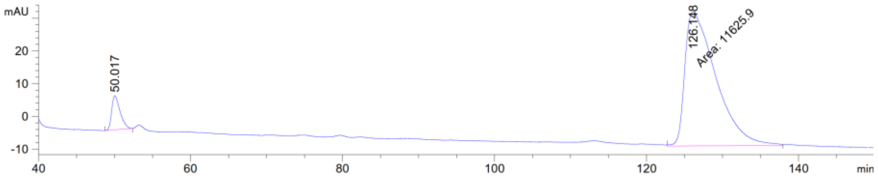

Signal 1: DAD1 A, Sig=220,4 Ref=off

Peak #	RetTime [min]	Type	Width [min]	Area [mAU*s]	Height [mAU]	Area %
1	50.017	BB	1.0431	801.88135	10.45132	6.4523
2	126.148	MM	4.7192	1.16259e4	41.05920	93.5477
Totals :				1.24278e4	51.51051	

(R)-N,N,4a-Trimethyl-4-oxo-3,4,4a,5-tetrahydroindeno[1,2-*c*]pyran-9-carboxamide (3p)

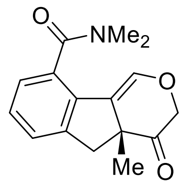

Prepared following General Procedure L from prochiral diketone **1p**. Obtained as colorless oil. **^{1}H NMR** (300 MHz, $CDCl_3$) δ 7.33 – 7.20 (m, 2H), 7.10 (d, *J* = 7.2 Hz, 1H), 6.99 (br s, 1H), 4.66 (d, *J* = 18.2 Hz, 1H), 4.17 (d, *J* = 18.1 Hz, 1H), 3.49 (d, *J* = 17.0 Hz, 1H), 3.17 (d, *J* = 1.6 Hz, 3H), 2.88 (s, 3H), 2.81 (d, *J* = 17.2 Hz, 1H), 1.40 (d, *J* = 1.7 Hz, 3H) ppm. **^{13}C NMR** (75 MHz, $CDCl_3$) δ 211.37 (CO), 170.71 (CO), 141.99 (C), 139.88 (CH), 131.70 (C), 131.08 (C), 128.08 (CH), 126.24 (CH), 125.07 (CH), 71.50 (CH_2), 52.59 (C), 39.35 (CH_2), 38.35 (CH_3 amide), 34.89 (CH_3 amide), 23.85 (CH_3) ppm. **HRMS** [APCI]: *m/z* calculated for $C_{16}H_{18}NO_3$ [M + H]$^+$: 272.1281, found 272.1283.

Enantioselectivity was determined by chiral HPLC analysis on a Chiralpack IF3 column at rt (Hexane:*i*PrOH = 95:5, 1 mL/min).

A: Racemic sample prepared using [Ir(COD)$_2$]BAr$^F{}_4$ / *rac*-Binap (5 mol%) at 130 ˚C.

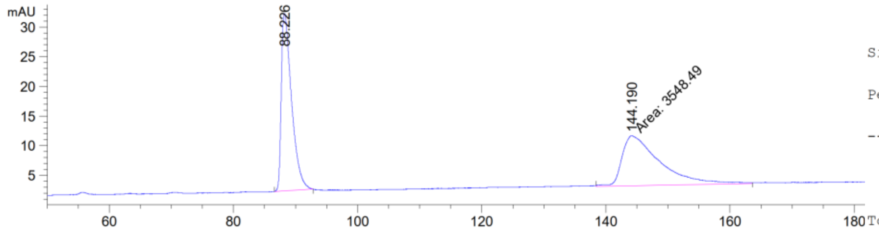

Signal 1: DAD1 A, Sig=220,4 Ref=off

Peak #	RetTime [min]	Type	Width [min]	Area [mAU*s]	Height [mAU]	Area %
1	88.232	BB	1.3894	6120.70459	52.99802	51.3319
2	144.080	MM	6.6160	5803.07031	14.61870	48.6681
Totals :				1.19238e4	67.61672	

B: Asymmetric sample prepared using [Ir(COD)$_2$]BAr$^F{}_4$ / *(R)*-DTBM-Segphos (5 mol%) at 130 ˚C (18% yield, 91:9 er).

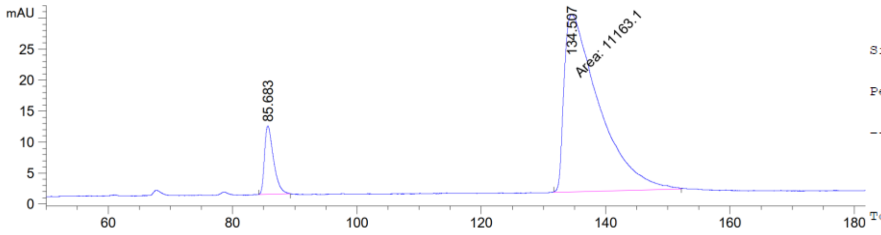

Signal 2: DAD1 B, Sig=254,4 Ref=off

Peak #	RetTime [min]	Type	Width [min]	Area [mAU*s]	Height [mAU]	Area %
1	85.683	BB	1.1838	1111.04907	11.06162	9.0519
2	134.507	MM	6.5069	1.11631e4	28.59300	90.9481
Totals :				1.22741e4	39.65462	

(4a*R*,9a*S*)-4a-hydroxy-*N,N*-dimethyl-1-oxo-9a-(trifluoromethyl)-2,3,4,4a,9,9a-hexahydro-1*H*-fluorene-5-carboxamide (2q)

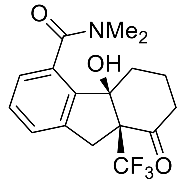

Prepared following General Procedure L from prochiral diketone **1q**. Obtained as colorless oil. **^{1}H NMR** (500 MHz, $CDCl_3$) δ 7.20 – 7.12 (m, 2H), 6.96 (d, *J* = 7.3 Hz, 1H), 4.04 (br, 1H), 3.66 (d, *J* = 15.9 Hz, 1H), 3.22 (d, *J* = 15.9 Hz, 1H), 3.01 (s, 3H), 2.91 (s, 3H), 2.59 – 2.50 (m, 1H), 2.37 – 2.27 (m, 2H), 2.20 – 2.15 (m, 1H), 2.00 – 1.90 (m, 1H), 1.28 – 1.14 (m, 1H) ppm. **^{13}C NMR** (126 MHz, $CDCl_3$) δ 203.5 (CO), 171.1 (CO), 142.6 (C), 141.2 (C), 131.6 (C), 128.9 (CH), 126.7 (CH), 125.8 (CH), 124.8 (CF_3, q, *J* = 284.1 Hz), 86.2 (C), 68.3 (C, q, *J* = 22.9 Hz), 39.7 (CH_3 amide), 39.7 (CH_2), 35.2 (CH_3 amide), 33.5 (CH_2), 33.3 (CH_2, q, *J* = 2.6 Hz), 19.6 (CH_2) ppm. **^{19}F NMR** (471 MHz, $CDCl_3$) δ -62.57 (s, 3F) ppm. **HRMS** [APCI]: *m/z* calculated for $C_{17}H_{19}F_3NO_3$ $[M + H]^+$: 342.1312, found 342.1317.

Enantioselectivity was determined by chiral HPLC analysis on a Chiralpack OZH column at rt (Hexane:*i*PrOH = 92:8, 1 mL/min).

A: Racemic sample prepared using [Ir(COD)$_2$]BAr$^F{}_4$ / *rac*-Binap (5 mol%) at 130 ˚C.

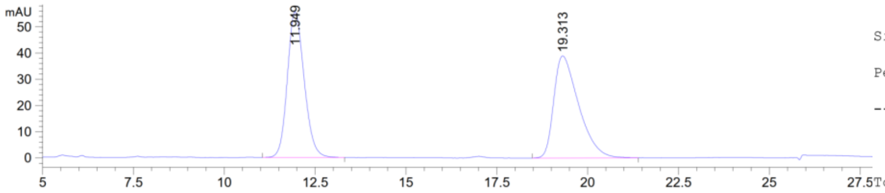

Signal 2: DAD1 B, Sig=254,4 Ref=off

Peak #	RetTime [min]	Type	Width [min]	Area [mAU*s]	Height [mAU]	Area %
1	11.949	BB	0.4976	1787.00867	55.51065	49.1202
2	19.313	BB	0.6784	1851.02539	38.95158	50.8798
Totals :				3638.03406	94.46222	

B: Asymmetric sample prepared using [Ir(COD)$_2$]BArF$_4$ / (*R*)-Segphos (5 mol%) at 130 ˚C (94% yield, 92:8 er).

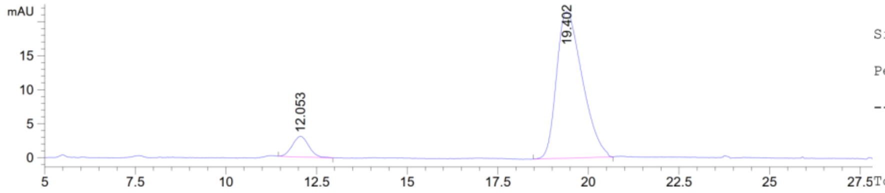

Signal 2: DAD1 B, Sig=254,4 Ref=off

Peak #	RetTime [min]	Type	Width [min]	Area [mAU*s]	Height [mAU]	Area %
1	12.053	BB	0.4192	95.01324	3.01855	8.2064
2	19.402	BB	0.7106	1062.77502	21.57701	91.7936
Totals :				1157.78826	24.59556	

(4a*R*,9a*S*)-9a-fluoro-4a-hydroxy-*N,N*-dimethyl-1-oxo-2,3,4,4a,9,9a-hexahydro-1*H*-fluorene-5-carboxamide (2r)

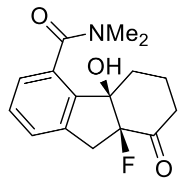

Prepared following General Procedure L from prochiral diketone **1r**. Reaction time: 24 h. Obtained as colorless solid. **^{1}H NMR** (500 MHz, $CDCl_3$) δ 7.34 – 7.26 (m, 2H), 7.14 (d, *J* = 7.3 Hz, 1H), 3.44 – 3.18 (m, 3H), 3.12 (s, 3H), 2.88 (s, 3H), 2.76 – 2.68 (m, 1H), 2.68 – 2.59 (m, 1H), 2.53 (td, *J* = 14.3, 5.7 Hz, 1H), 2.16 (qt, *J* = 12.5, 3.1 Hz, 1H), 1.94 – 1.86 (m, 1H), 1.75 (td, *J* = 14.0, 3.6 Hz, 1H) ppm. **^{13}C NMR** (126 MHz, $CDCl_3$) δ 205.11 (CO, d, *J* = 17.8 Hz), 170.3 (CO), 142.4 (C), 137.4 (C), 132.9 (C), 128.8 (CH), 126.5 (CH), 126.0 (CH), 104.4 (CF, d, *J* = 198.1 Hz), 88.8 (COH, d, *J* = 17.3 Hz), 39.7 (CH_2), 39.2 (CH_2, d, *J* = 25.2 Hz), 39.6 (CH_3 amide), 37.4 (CH_2, d, *J* = 2.5 Hz), 35.0 (CH_3 amide), 21.4 (CH_2) ppm. **^{19}F NMR** (471 MHz, $CDCl_3$) δ -176.4 (s, 1F) ppm. **HRMS** [APCI]: *m/z* calculated for $C_{16}H_{19}FNO_3$ $[M + H]^+$: 292.1343, found 292.1345. **MP** = 152.8 – 153.8 °C

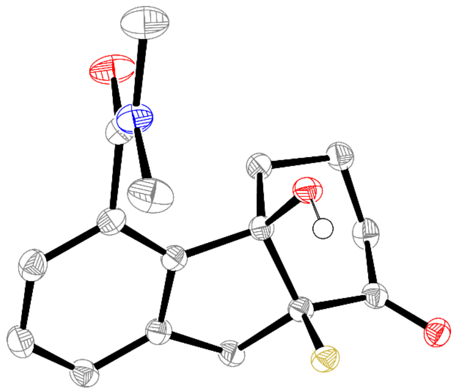

Figure S4. X-ray structure of **2r** (CCDC 2504167)

Enantioselectivity was determined by chiral HPLC analysis on a Chiralpack IE3 column at rt (Hexane:*i*PrOH = 85:15, 1 mL/min).

A: Racemic sample prepared using $[Ir(COD)_2]BAr^F_4$ / *rac*-Binap (5 mol%) at 130 ˚C.

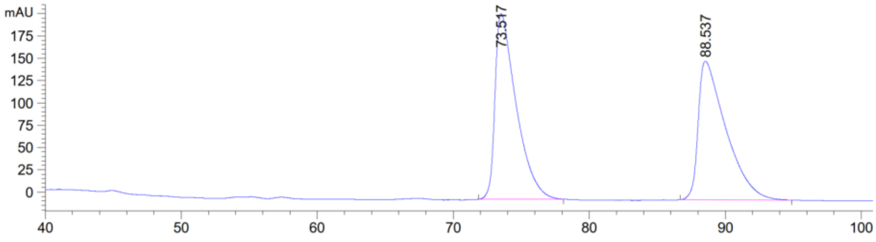

Signal 3: DAD1 C, Sig=210,4 Ref=off

Peak #	RetTime [min]	Type	Width [min]	Area [mAU*s]	Height [mAU]	Area %
1	73.517	BB	1.4877	2.25811e4	207.81931	50.6325
2	88.537	BB	1.8496	2.20169e4	155.33815	49.3675
Totals :				4.45979e4	363.15746	

B: Asymmetric sample prepared using $[Ir(COD)_2]BAr^F_4$ / *(R)*-Segphos (5 mol%) at 130 ˚C (81% yield, 90:10 er).

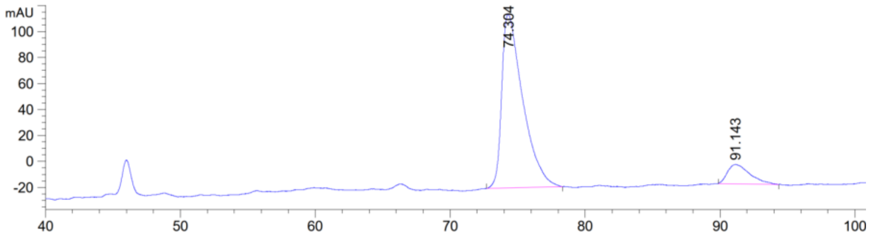

Signal 3: DAD1 C, Sig=210,4 Ref=off

Peak #	RetTime [min]	Type	Width [min]	Area [mAU*s]	Height [mAU]	Area %
1	74.304	BB	1.3411	1.44747e4	133.69859	89.5029
2	91.143	BB	1.3434	1697.62000	14.92108	10.4971
Totals :				1.61723e4	148.61968	

C: Asymmetric sample prepared using $[Ir(COD)_2]BAr^F_4$ / *(R)*-DTBM-Segphos (5 mol%) at 130 ˚C (83% yield, 79:21 er).

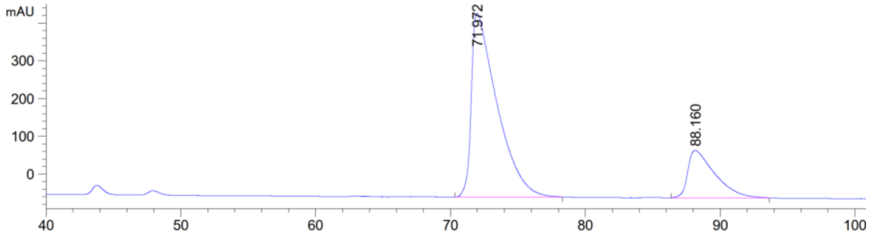

Signal 3: DAD1 C, Sig=210,4 Ref=off

Peak #	RetTime [min]	Type	Width [min]	Area [mAU*s]	Height [mAU]	Area %
1	71.972	BB	1.6795	6.44723e4	486.68130	78.7531
2	88.160	BB	1.6366	1.73941e4	125.22191	21.2469
Totals :				8.18664e4	611.90321	

(4b*R*,9a*S*)-4b-hydroxy-*N,N*-dimethyl-9-oxo-9a-(trifluoromethyl)-4b,5,6,7,8,9,9a,10-octahydrobenzo[*a*]azulene-4-carboxamide (2s)

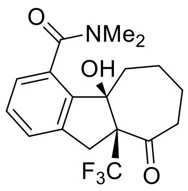

Prepared following General Procedure L from prochiral diketone **1s**. Reaction temperature: 140 ˚C. Obtained as colorless solid. **^{1}H NMR** (500 MHz, $CDCl_3$) δ 7.29 – 7.26 (m, 1H), 7.26 – 7.23 (m, 1H), 7.14 (d, *J* = 7.2 Hz, 1H), 3.52 (d, *J* = 16.8 Hz, 1H), 3.36 (d, *J* = 18.0 Hz, 1H), 3.29 (ddd, *J* = 13.4, 10.6, 3.0 Hz, 1H), 3.15 (s, 3H), 2.98 (s, 3H), 2.60 – 2.54 (m, 1H), 2.30 – 2.19 (m, 1H), 2.14 (dd, *J* = 15.3, 6.1 Hz, 1H), 2.05 – 1.97 (m, 1H), 1.69 – 1.59 (m, 1H), 1.56 – 1.43 (m, 2H) ppm. **^{13}C NMR** (126 MHz, $CDCl_3$) δ 207.6 (CO), 171.7 (CO), 145.9 (C), 138.5 (C), 130.3 (C), 128.0 (CH), 126.8 (CH), 126.0 (CH), 125.7 (CF_3, q, *J* = 285.6 Hz), 82.7 (COH), 72.4 (C, q, *J* = 20.9 Hz), 42.5 (CH_2, q, *J* = 2.9 Hz), 40.6 (CH_2), 40.0 (CH_3 amide), 35.7 (CH_3 amide), 34.4 (CH_2, q, *J* = 2.7 Hz), 28.4 (CH_2), 23.0 (CH_2) ppm. **^{19}F NMR** (471 MHz, $CDCl_3$) δ -64.7 (s, 3F) ppm. **HRMS** [APCI]: *m/z* calculated for $C_{18}H_{21}F_3NO_3$ $[M + H]^+$: 356.1468, found 356.1465. **MP** = 159.9 – 161.3 °C

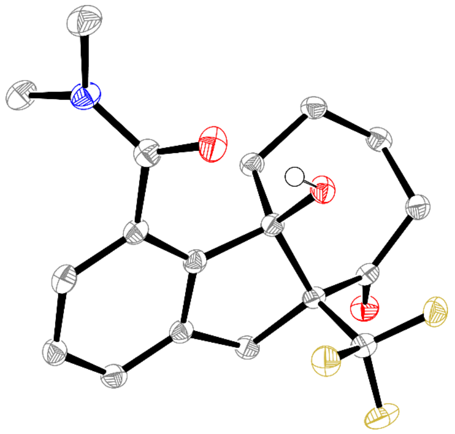

Figure S5. X-ray structure of **2s** (CCDC 2504169)

Enantioselectivity was determined by chiral HPLC analysis on a Chiralpack IG3 column at rt (Hexane:*i*PrOH = 95:5, 1 mL/min).

A: Racemic sample prepared using $[Ir(COD)_2]BAr^F_4$ / *rac*-Binap (5 mol%) at 140 ˚C.

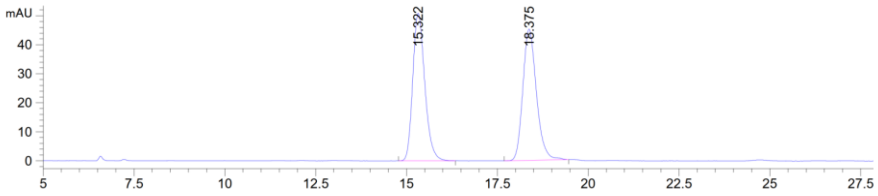

Signal 1: DAD1 A, Sig=220,4 Ref=off

Peak #	RetTime [min]	Type	Width [min]	Area [mAU*s]	Height [mAU]	Area %
1	15.323	BB	0.3566	7335.50098	318.64142	49.6654
2	18.375	BB	0.4031	7434.35400	284.29178	50.3346
Totals :				1.47699e4	602.93320	

B: <u>Asymmetric sample</u> prepared using [Ir(COD)$_2$]BAr$^F{}_4$ / *(R)*-DTBM-Segphos (5 mol%) at 140 ˚C, 5 days (81% yield, 88:12 er).

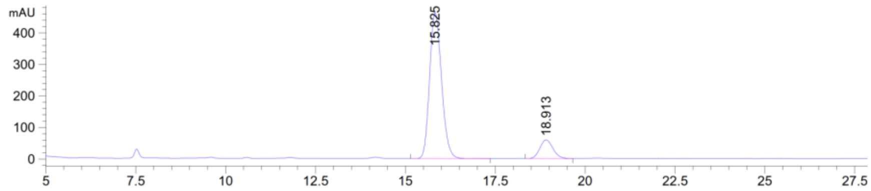

Signal 1: DAD1 A, Sig=220,4 Ref=off

Peak #	RetTime [min]	Type	Width [min]	Area [mAU*s]	Height [mAU]	Area %
1	15.825	BB	0.3605	1.07149e4	462.18643	87.7621
2	18.913	BB	0.3914	1494.12671	59.40521	12.2379
Totals :				1.22090e4	521.59164	

C: After <u>recrystallization</u> from a sample with 88:12 er: >99:1 er

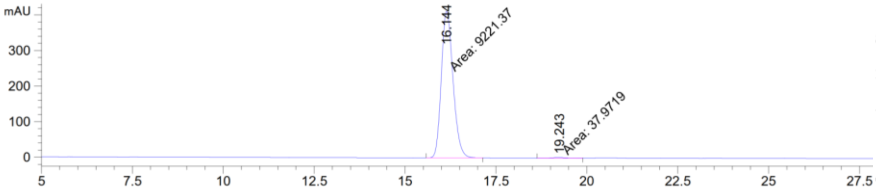

Signal 1: DAD1 A, Sig=220,4 Ref=off

Peak #	RetTime [min]	Type	Width [min]	Area [mAU*s]	Height [mAU]	Area %
1	16.144	MM	0.3730	9221.36914	412.06180	99.5899
2	19.243	MM	0.4470	37.97194	1.41565	0.4101
Totals :				9259.34108	413.47745	

(4b*R*,9a*S*)-9a-fluoro-4b-hydroxy-*N,N*-dimethyl-9-oxo-4b,5,6,7,8,9,9a,10-octahydrobenzo[*a*]azulene-4-carboxamide (2t)

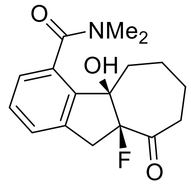

Prepared following <u>General Procedure L</u> from prochiral diketone **1t**. Reaction temperature: 110 ˚C. Obtained as colorless oil. **^{1}H NMR** (500 MHz, CDCl$_3$) δ 7.32 – 7.27 (m, 2H), 7.11 (d, *J* = 6.8 Hz, 1H), 3.70 (dd, *J* = 21.0, 17.6 Hz, 1H), 3.30 – 3.16 (m, 2H), 3.14 (s, 3H), 2.96 (s, 3H), 2.45 – 2.35 (m, 2H), 2.21 – 2.10 (m, 1H), 2.05 – 1.98 (m, 1H), 1.71 – 1.63 (m, 1H), 1.55 – 1.44 (m, 1H), 1.42 – 1.33 (m, 1H) ppm. **^{13}C NMR** (126 MHz, CDCl$_3$) δ 207.9 (CO, d, *J* = 27.7 Hz), 171.3 (CO), 142.6 (C), 139.3 (C), 132.7 (C), 128.9 (CH), 126.3 (CH), 126.2 (CH), 106.0 (CF, d, *J* = 197.9 Hz), 81.1 (COH, d, *J* = 17.2 Hz), 39.8 (CH$_3$ amide), 38.7 (CH$_2$), 38.2 (CH$_2$, d, *J* = 24.8 Hz) 37.2 (CH$_2$), 35.3 (CH$_3$ amide), 27.3 (CH$_2$), 21.8 (CH$_2$) ppm. **^{19}F NMR** (471 MHz, CDCl$_3$) δ -160.3 (s, 1F) ppm. **HRMS** [APCI]: *m/z* calculated for $C_{17}H_{21}FNO_3$ [M + H]$^+$: 306.1500, found 306.1502.

Enantioselectivity was determined by chiral HPLC analysis on a Chiralpack IE3 column at rt (Hexane:*i*PrOH = 90:10, 1 mL/min).

A: <u>Racemic sample</u> prepared using [Ir(COD)$_2$]BAr$^F{}_4$ / *rac*-Binap (5 mol%) at 110 ˚C.

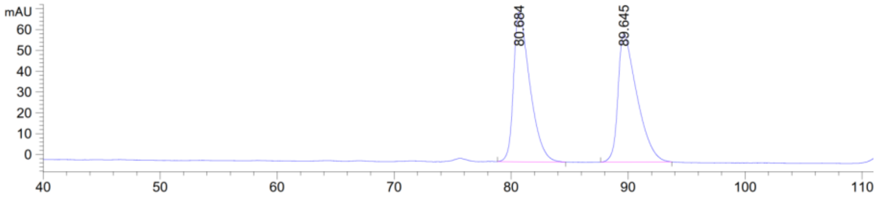

Signal 1: DAD1 A, Sig=220,4 Ref=off

Peak #	RetTime [min]	Type	Width [min]	Area [mAU*s]	Height [mAU]	Area %
1	80.684	BB	1.3161	6963.11035	71.88556	50.1878
2	89.645	BB	1.5037	6910.99951	61.18748	49.8122
Totals :				1.38741e4	133.07304	

B: <u>Asymmetric sample</u> prepared using [Ir(COD)$_2$]BAr$^F{}_4$ / *(R)*-Segphos (5 mol%) at 110 ˚C (53% yield, 87:13 er).

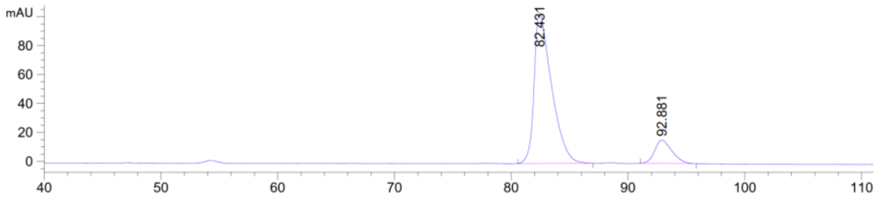

Signal 1: DAD1 A, Sig=220,4 Ref=off

Peak #	RetTime [min]	Type	Width [min]	Area [mAU*s]	Height [mAU]	Area %
1	82.431	BB	1.4337	1.08346e4	104.14994	86.6862
2	92.881	BB	1.2350	1664.04224	16.13816	13.3138
Totals :				1.24986e4	120.28810	

(3a*R*,8a*S*)-8a-fluoro-3a-hydroxy-*N,N*-dimethyl-1-oxo-1,2,3,3a,8,8a-hexahydrocyclopenta[*a*]indene-4-carboxamide (2u)

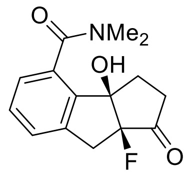

Prepared following General Procedure L from prochiral diketone **1u**. Obtained as colorless oil. ^{1}**H NMR** (500 MHz, $CDCl_3$) δ 7.33 (t, *J* = 7.5 Hz, 1H), 7.27 (dd, *J* = 7.6, 1.2 Hz, 1H), 7.21 (dd, *J* = 7.5, 1.1 Hz, 1H), 4.18 (br, 1H), 3.41 – 3.23 (m, 2H), 3.16 (s, 3H), 3.03 (s, 3H), 2.76 – 2.67 (m, 1H), 2.63 – 2.54 (m, 1H), 2.33 – 2.24 (m, 1H), 2.23 – 2.14 (m, 1H) ppm. ^{13}**C NMR** (126 MHz, $CDCl_3$) δ 211.13 (CO, d, *J* = 18.3 Hz), 170.7 (CO), 142.1 (C), 138.92 (CH, d, *J* = 3.6 Hz), 132.5 (C), 129.1 (CH), 127.0 (CH), 126.5 (CH), 102.4 (CF, d, *J* = 210.1 Hz), 85.7 (COH, d, *J* = 15.1 Hz), 39.8 (CH_3 amide), 37.2 (CH_2, d, *J* = 25.8 Hz), 35.9 (CH_2, d, *J* = 2.1 Hz) 35.4 (CH_3 amide), 32.8 (CH_2) ppm. ^{19}**F NMR** (471 MHz, $CDCl_3$) δ -178.8 (s, 1F) ppm. **HRMS** [APCI]: *m/z* calculated for $C_{15}H_{17}FNO_3$ $[M + H]^+$: 278.1187, found 278.1183.

Enantioselectivity was determined by chiral HPLC analysis on a Chiralpack IE3 column at rt (Hexane:*i*PrOH = 90:10, 1 mL/min).

A: Racemic sample prepared using $[Ir(COD)_2]BAr^F_4$ / *rac*-Binap (5 mol%) at 130 ˚C.

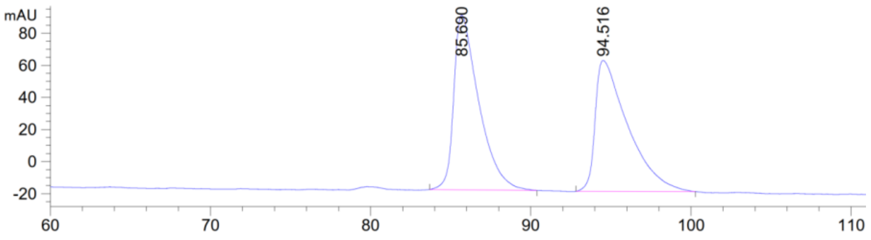

Signal 3: DAD1 C, Sig=210,4 Ref=off

Peak #	RetTime [min]	Type	Width [min]	Area [mAU*s]	Height [mAU]	Area %
1	85.690	BB	1.4294	1.18570e4	108.80546	50.5546
2	94.516	BB	1.8327	1.15969e4	81.56771	49.4454
Totals :				2.34539e4	190.37317	

B: Asymmetric sample prepared using $[Ir(COD)_2]BAr^F_4$ / *(R)*-Segphos (5 mol%) at 130 ˚C (97% yield, 76:24 er).

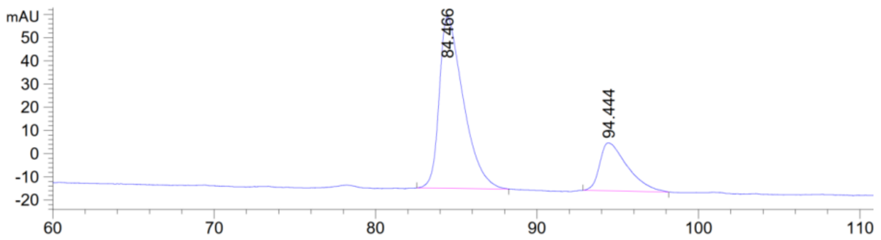

Signal 3: DAD1 C, Sig=210,4 Ref=off

Peak #	RetTime [min]	Type	Width [min]	Area [mAU*s]	Height [mAU]	Area %
1	84.466	BB	1.4049	7659.65479	73.92352	75.6781
2	94.444	BB	1.4011	2461.71411	20.67473	24.3219
Totals :				1.01214e4	94.59826	

C: Asymmetric sample prepared using $[Ir(COD)_2]BAr^F_4$ / *(R)*-DTBM-Segphos (5 mol%) at 130 ˚C (81% yield, 71:29 er).

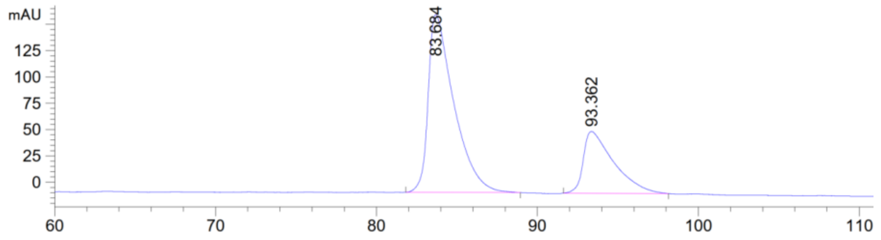

Signal 3: DAD1 C, Sig=210,4 Ref=off

Peak #	RetTime [min]	Type	Width [min]	Area [mAU*s]	Height [mAU]	Area %
1	83.684	BB	1.4974	1.88123e4	168.90424	70.8329
2	93.362	BB	1.5896	7746.41309	58.85320	29.1671
Totals :				2.65588e4	227.75743	

(3a*R*,8a*R*)-3a-hydroxy-*N,N*,8a-trimethyl-1-oxo-1,2,3,3a,8,8a-hexahydrocyclopenta[*a*]indene-4-carboxamide (2v)

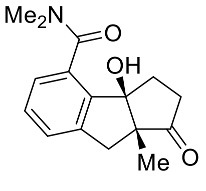

Prepared following General Procedure L from prochiral diketone **1v**. Obtained as colorless oil. ^{1}**H NMR** (300 MHz, $CDCl_3$) δ 7.29 – 7.24 (m, 2H), 7.16 (dd, *J* = 6.1, 1.7 Hz, 1H), 5.05 (s, 1H), 3.28 (d, *J* = 16.4 Hz, 1H), 3.21 (s, 3H), 3.11 (s, 3H), 2.95 (d, *J* = 16.4 Hz, 1H), 2.54 (ddd, *J* = 18.6, 8.7, 2.6 Hz, 1H), 2.42 – 2.27 (m, 2H), 1.85 (ddd, *J* = 18.6, 11.3, 9.4 Hz, 1H), 1.27 (s, 3H) ppm. ^{13}**C NMR** (75 MHz, $CDCl_3$) δ 221.86 (CO), 171.90 (CO), 144.42 (C), 144.25 (C), 131.53 (C), 128.39 (CH), 126.84 (CH), 125.88 (CH), 87.58

(COH), 59.12 (C), 41.57 (CH_2), 39.83 (CH_3 amide), 37.35 (CH_2), 35.50 (CH_3 amide), 32.38 (CH_2), 15.52 (CH_3) ppm. **HRMS** [APCI]: *m/z* calculated for $C_{16}H_{20}NO_3$ $[M + H]^+$: 274.1438, found 274.1439.

Enantioselectivity was determined by chiral HPLC analysis on a Chiralpack IG3 column at rt (Hexane:*i*PrOH = 90:10, 1 mL/min).

A: Racemic sample prepared using $[Ir(COD)_2]BAr^F_4$ / *rac*-Binap (5 mol%) at 130 ˚C.

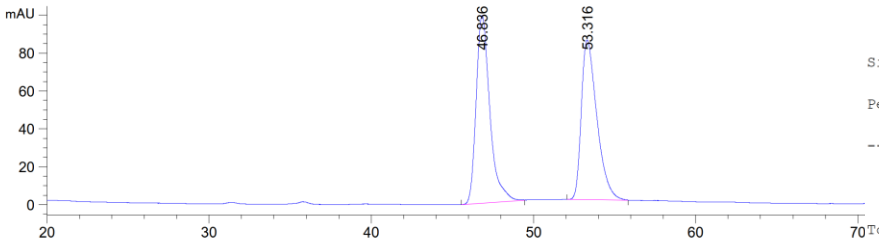

Signal 1: DAD1 A, Sig=220,4 Ref=off

Peak #	RetTime [min]	Type	Width [min]	Area [mAU*s]	Height [mAU]	Area %
1	46.836	BB	0.8911	5817.60791	99.07535	50.8700
2	53.316	BB	0.9791	5618.61816	84.35500	49.1300
Totals :				1.14362e4	183.43035	

B: Asymmetric sample prepared using $[Ir(COD)_2]BAr^F_4$ / *(R)*-DTBM-Segphos (5 mol%) at 130 ˚C (89% yield, 86:14 er).

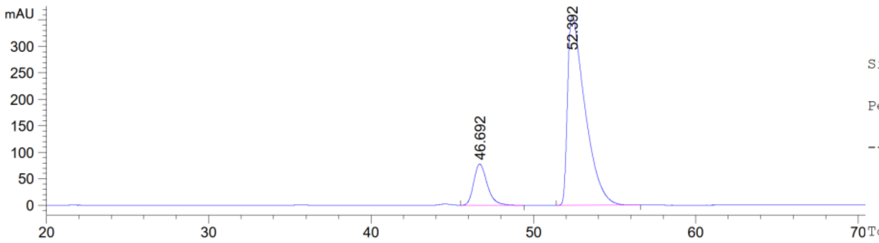

Signal 1: DAD1 A, Sig=220,4 Ref=off

Peak #	RetTime [min]	Type	Width [min]	Area [mAU*s]	Height [mAU]	Area %
1	46.692	BB	0.8802	4507.39014	77.76432	14.4007
2	52.392	BB	1.0829	2.67925e4	358.90402	85.5993
Totals :				3.12999e4	436.66834	

(3a*R*,8a*R*)-3a-hydroxy-*N,N*-dimethyl-1-oxo-8a-propyl-1,2,3,3a,8,8a-hexahydrocyclopenta[*a*]indene-4-carboxamide (2w)

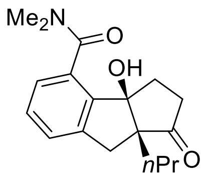

Prepared following General Procedure L from prochiral diketone **1w**. Reaction time: 24 h. Obtained as pale-yellow oil. **^{1}H NMR** (300 MHz, $CDCl_3$) δ 7.33 – 7.21 (m, 2H), 7.19 – 7.11 (m, 1H), 5.11 (s, 1H), 3.31 (d, J = 16.3 Hz, 1H), 3.21 (s, 3H), 3.11 (s, 3H), 2.97 (d, J = 16.3 Hz, 1H), 2.62 – 2.42 (m, 1H), 2.42 – 2.33 (m, 2H), 1.98 – 1.53 (m, 4H), 1.41 – 1.20 (m, 1H), 0.96 (t, J = 7.2 Hz, 3H) ppm. **^{13}C NMR** (75 MHz, $CDCl_3$) δ 221.56 (CO), 171.98 (CO), 144.78 (C), 144.41 (C), 131.36 (C), 128.34 (CH), 126.80 (CH), 125.80 (CH), 87.84 (C), 62.58 (C), 40.16 (CH_2), 39.77 (CH_3 amide), 37.50 (CH_2), 35.46 (CH_3 amide), 32.94 (CH_2), 32.67 (CH_2), 18.18 (CH_2), 14.96 (CH_3) ppm. **HRMS** [APCI]: *m/z* calculated for $C_{18}H_{24}NO_3$ $[M + H]^+$: 302.1751, found 302.1746.

Enantioselectivity was determined by chiral HPLC analysis on a Chiralpack IG3 column at rt (Hexane:*i*PrOH = 95:5, 1 mL/min).

A: Racemic sample prepared using $[Ir(COD)_2]BAr^F_4$ / *rac*-Binap (5 mol%) at 130 ˚C.

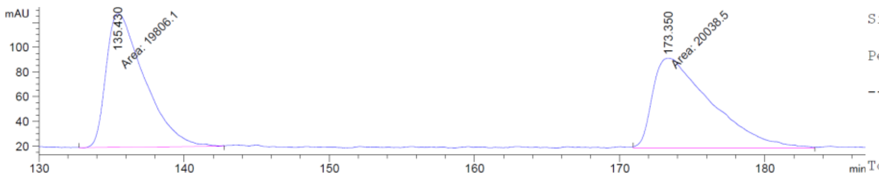

Signal 3: DAD1 C, Sig=210,4 Ref=off

Peak #	RetTime [min]	Type	Width [min]	Area [mAU*s]	Height [mAU]	Area %
1	135.430	MM	3.0562	1.98061e4	108.01137	49.7084
2	173.350	MM	4.6231	2.00385e4	72.24083	50.2916
Totals :				3.98446e4	180.25220	

B: Asymmetric sample prepared using $[Ir(COD)_2]BAr^F_4$ / *(R)*-DTBM-Segphos (5 mol%) at 130 ˚C (78% yield, 92:8 er).

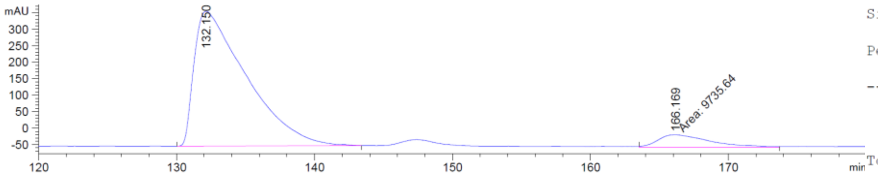

Signal 3: DAD1 C, Sig=210,4 Ref=off

Peak #	RetTime [min]	Type	Width [min]	Area [mAU*s]	Height [mAU]	Area %
1	132.150	BB	3.4921	1.07330e5	408.39612	91.6836
2	166.169	MM	4.3180	9735.64355	37.57733	8.3164
Totals :				1.17066e5	445.97345	

(3a*R*,8a*S*)-3a-hydroxy-*N,N*-dimethyl-1-oxo-8a-phenyl-1,2,3,3a,8,8a-hexahydrocyclopenta[*a*]indene-4-carboxamide (2x)

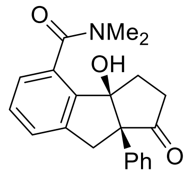

Prepared following General Procedure L from prochiral diketone **1x**. Obtained as a yellow oil. **^{1}H NMR** (500 MHz, $CDCl_3$) δ 7.35 – 7.30 (m, 2H), 7.29 – 7.17 (m, 5H), 7.11 – 7.07 (m, 1H), 4.85 (br, 1H), 3.62 (d, *J* = 16.3 Hz, 1H), 3.47 (d, *J* = 16.3 Hz, 1H), 3.09 (s, 3H), 3.00 (s, 3H), 2.61 (ddd, *J* = 19.4, 8.9, 3.7 Hz, 1H), 2.44 – 2.33 (m, 2H), 1.95 (ddd, *J* = 19.5, 10.7, 9.0 Hz, 1H) ppm. **^{13}C NMR** (126 MHz, $CDCl_3$) δ 219.9 (CO), 171.7 (CO), 143.8 (C), 143.8 (C), 136.6 (C), 131.8 (C), 128.7 (CH), 128.6 (CH), 128.4 (CH), 127.4 (CH), 126.8 (CH), 126.1 (CH), 88.9 (COH), 67.5 (C), 41.6 (CH_2), 39.8 (CH_3 amide), 38.1 (CH_2), 35.4 (CH_3 amide), 32.7 (CH_2) ppm. **HRMS** [APCI]: *m/z* calculated for $C_{21}H_{22}NO_3$ $[M + H]^+$: 336.1594, found 336.1591.

Enantioselectivity was determined by chiral HPLC analysis on a Chiralpack IE3 column at rt (Hexane:*i*PrOH = 70:30, 1 mL/min).

A: Racemic sample prepared using $[Ir(COD)_2]BAr^F_4$ / *rac*-Binap (5 mol%) at 130 ˚C.

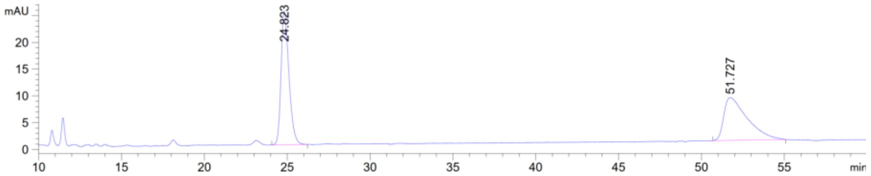

Signal 2: DAD1 B, Sig=254,4 Ref=off

Peak #	RetTime [min]	Type	Width [min]	Area [mAU*s]	Height [mAU]	Area %
1	24.823	BB	0.4984	825.77258	24.93073	51.2131
2	51.727	BB	1.1613	786.65131	7.98555	48.7869
Totals :				1612.42389	32.91628	

B: Asymmetric sample prepared using $[Ir(COD)_2]BAr^F_4$ / *(R)*-DTBM-Segphos (5 mol%) at 130 ˚C (98% yield, 86:14 er).

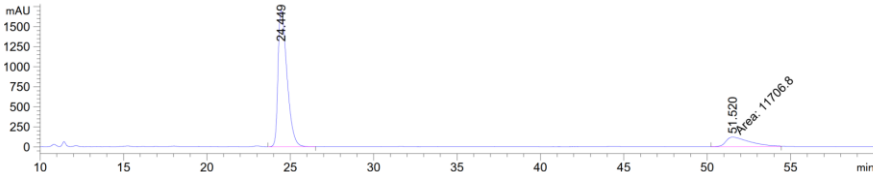

Signal 2: DAD1 B, Sig=254,4 Ref=off

Peak #	RetTime [min]	Type	Width [min]	Area [mAU*s]	Height [mAU]	Area %
1	24.450	BB	0.5209	4668.53809	133.85597	85.6710
2	51.506	BB	1.1558	780.83972	7.97823	14.3290
Totals :				5449.37781	141.83420	

(3a*R*,8a*R*)-3a-hydroxy-7-methoxy-*N,N*,8a-trimethyl-1-oxo-1,2,3,3a,8,8a-hexahydrocyclopenta[*a*]indene-4-carboxamide (2y)

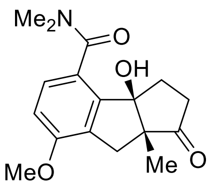

Prepared following General Procedure L from prochiral diketone **1y**. Obtained as colorless oil. **^{1}H NMR** (300 MHz, $CDCl_3$) δ 7.16 (d, *J* = 8.3 Hz, 1H), 6.70 (d, *J* = 8.3 Hz, 1H), 3.81 (s, 3H), 3.23 (d, *J* = 16.8 Hz, 1H), 3.15 – 3.08 (m, 6H), 2.77 (d, *J* = 16.9 Hz, 1H), 2.56 – 2.15 (m, 3H), 1.93 – 1.74 (m, 1H), 1.23 (s, 3H) ppm. **^{13}C NMR** (75 MHz, $CDCl_3$) δ 221.85 (CO), 172.06 (CO), 157.25 (C), 146.93 (C), 131.97 (C), 128.38 (CH), 123.31 (C), 108.96 (CH), 88.09 (C), 58.84 (C), 55.50 (CH_3), 40.04 (CH_3 amide), 38.68 (CH_2), 37.37 (CH_2), 35.61 (CH_3 amide), 32.53 (CH_2), 15.65 (CH_3) ppm. **HRMS** [APCI]: *m/z* calculated for $C_{17}H_{22}NO_4$ $[M + H]^+$: 304.1543, found 304.1536.

Enantioselectivity was determined by chiral HPLC analysis on a Chiralpack IA3 column at rt (Hexane:*i*PrOH = 90:10, 1 mL/min).

A: Racemic sample prepared using [Ir(COD)$_2$]BAr$^F{}_4$ / *rac*-Binap (5 mol%) at 130 ˚C.

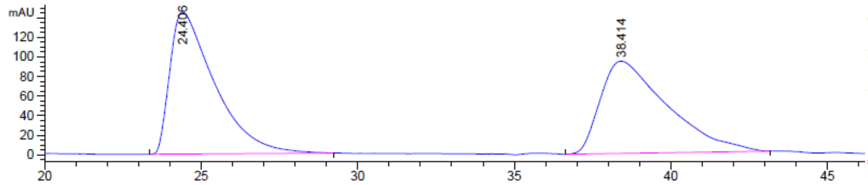

Signal 2: DAD1 B, Sig=254,4 Ref=off

Peak #	RetTime [min]	Type	Width [min]	Area [mAU*s]	Height [mAU]	Area %
1	24.406	BB	1.3579	1.43769e4	144.60342	50.8052
2	38.414	MM	1.7923	1.39212e4	94.08479	49.1948
Totals :				2.82982e4	238.68822	

B: Asymmetric sample prepared using [Ir(COD)$_2$]BAr$^F{}_4$ / *(R)*-DTBM-Segphos (5 mol%) at 130 ˚C (87% yield, 88:12 er).

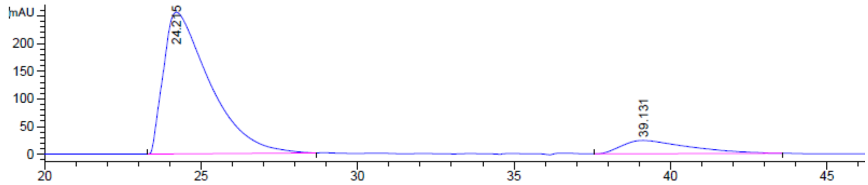

Signal 2: DAD1 B, Sig=254,4 Ref=off

Peak #	RetTime [min]	Type	Width [min]	Area [mAU*s]	Height [mAU]	Area %
1	24.215	BB	1.4418	2.62770e4	255.93033	88.0521
2	39.131	BB	1.7397	3565.56738	24.10483	11.9479
Totals :				2.98426e4	280.03516	

(3a*R*,8a*R*)-3a-hydroxy-*N,N*,8a-trimethyl-1-oxo-6-phenyl-1,2,3,3a,8,8a-hexahydrocyclopenta[*a*]indene-4-carboxamide (2z)

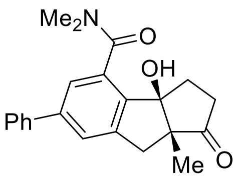

Prepared following General Procedure L from prochiral diketone **1z**. Obtained as colorless oil. **^{1}H NMR** (300 MHz, CDCl$_3$) δ 7.54 – 7.48 (m, 2H), 7.47 – 7.32 (m, 5H), 3.32 (d, *J* = 16.4 Hz, 1H), 3.21 (s, 3H), 3.13 (s, 3H), 2.98 (d, *J* = 16.3 Hz, 1H), 2.62 – 2.48 (m, 1H), 2.46 – 2.23 (m, 2H), 1.99 – 1.79 (m, 1H), 1.27 (s, 3H) ppm. **^{13}C NMR** (75 MHz, CDCl$_3$) δ 221.74 (CO), 171.79 (CO), 145.01 (C), 143.35 (C), 141.95 (C), 140.26 (C), 131.91 (C), 129.04 (CH), 127.98 (CH), 127.35 (CH), 125.48 (CH), 124.98 (CH), 87.38 (C), 59.38 (C), 41.58 (CH$_2$), 39.89 (CH$_3$ amide), 37.35 (CH$_2$), 35.52 (CH$_3$ amide), 32.39 (CH$_2$), 15.54 (CH$_3$) ppm. **HRMS** [APCI]: *m/z* calculated for $C_{22}H_{24}NO_3$ [M + H]$^+$: 350.1751, found 350.1748.

Enantioselectivity was determined by chiral HPLC analysis on a Chiralpack IG3 column at rt (Hexane:*i*PrOH = 90:10, 1 mL/min).

A: Racemic sample prepared using [Ir(COD)$_2$]BAr$^F{}_4$ / *rac*-Binap (5 mol%) at 130 ˚C.

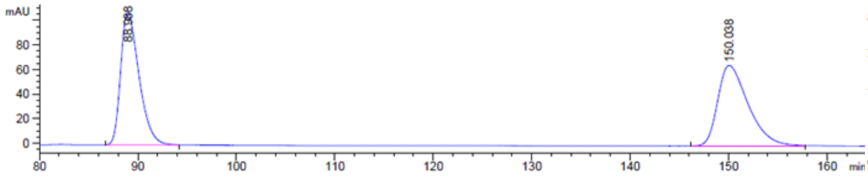

Signal 2: DAD1 B, Sig=254,4 Ref=off

Peak #	RetTime [min]	Type	Width [min]	Area [mAU*s]	Height [mAU]	Area %
1	88.988	BB	1.7861	1.39545e4	108.09895	49.5069
2	150.038	MM	3.6135	1.42325e4	65.64436	50.4931
Totals :				2.81870e4	173.74332	

B: Asymmetric sample prepared using [Ir(COD)$_2$]BAr$^F{}_4$ / *(S)*-C$_3$-Tunephos (5 mol%) at 130 ˚C (72% yield, 75:25 er).

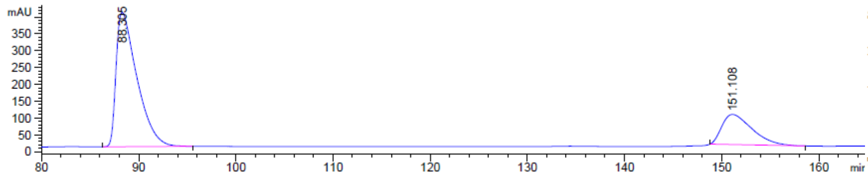

Signal 2: DAD1 B, Sig=254,4 Ref=off

Peak #	RetTime [min]	Type	Width [min]	Area [mAU*s]	Height [mAU]	Area %
1	88.305	BB	2.0387	5.92968e4	398.80484	75.4117
2	151.108	BB	2.5308	1.93340e4	89.48146	24.5883
Totals :				7.86308e4	488.28630	

(4b*R*,9a*R*)-4b-hydroxy-*N,N*,9a-trimethyl-9-oxo-4b,9,9a,10-tetrahydroindeno[1,2-*a*]indene-4-carboxamide (2α)

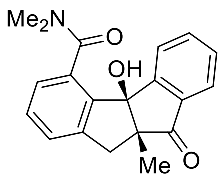

Prepared following General Procedure L from prochiral diketone **1α**. Obtained as pale-yellow solid. **^{1}H NMR** (500 MHz, $CDCl_3$) δ 7.84 (d, *J* = 7.8 Hz, 1H), 7.67 (d, *J* = 7.6 Hz, 1H), 7.62 (t, *J* = 7.5 Hz, 1H), 7.40 (t, *J* = 7.4 Hz, 1H), 7.25 – 7.19 (m, 2H), 7.01 (d, *J* = 7.1 Hz, 1H), 5.87 (s, 1H), 3.60 (d, *J* = 16.8 Hz, 1H), 3.19 (s, 3H), 3.04 (d, *J* = 16.8 Hz, 1H), 2.55 (s, 3H), 1.39 (s, 3H) ppm. **^{13}C NMR** (75 MHz, $CDCl_3$) δ 208.25 (CO), 172.75 (CO), 156.37 (C), 144.59 (C), 143.74 (C), 134.89 (C), 134.47 (CH), 132.91 (C), 129.02 (CH), 128.47 (CH), 127.40 (CH), 125.41 (CH), 124.76 (CH), 123.87 (CH), 87.82 (COH), 62.72 (C), 39.48 (CH_3 amide), 39.41 (CH_2), 35.35 (CH_3 amide), 18.37 (CH_3) ppm. **HRMS** [APCI]: *m/z* calculated for $C_{20}H_{20}NO_3$ $[M + H]^+$: 322.1438, found 322.1435. **MP** = 149.0 – 150.5 °C

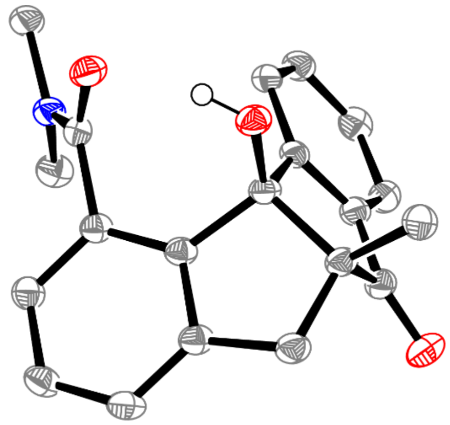

Figure S6. X-ray structure of **2α** (CCDC 2504166)

Enantioselectivity was determined by chiral HPLC analysis on a Chiralpack IG3 column at rt (Hexane:*i*PrOH = 90:10, 1 mL/min).

A: Racemic sample prepared using $[Ir(COD)_2]BAr^F_4$ / *rac*-Binap (10 mol%) at 130 ˚C.

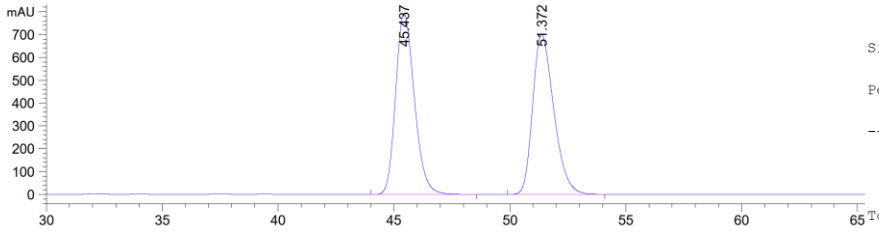

Signal 3: DAD1 C, Sig=210,4 Ref=off

Peak #	RetTime [min]	Type	Width [min]	Area [mAU*s]	Height [mAU]	Area %
1	45.437	BB	0.8754	4.47011e4	793.34485	50.1578
2	51.372	BB	0.9838	4.44198e4	700.51093	49.8422
Totals :				8.91209e4	1493.85577	

B: Asymmetric sample prepared using $[Ir(COD)_2]BAr^F_4$ / *(R)*-DTBM-Segphos (10 mol%) at 130 ˚C (83% yield, 84:16 er).

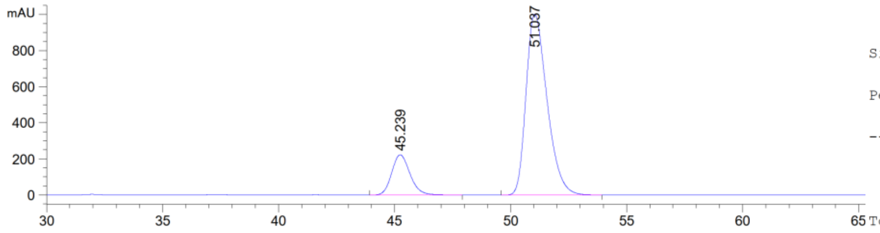

Signal 3: DAD1 C, Sig=210,4 Ref=off

Peak #	RetTime [min]	Type	Width [min]	Area [mAU*s]	Height [mAU]	Area %
1	45.239	BB	0.8474	1.22725e4	223.24045	16.3050
2	51.037	BB	0.9682	6.29959e4	1001.01520	83.6950
Totals :				7.52684e4	1224.25565	

(4a*R*,9b*R*)-*N*,*N*-dimethyl-12-oxo-3,4-dihydro-2*H*,5*H*-4a,9b-propanoindeno[1,2-*b*]pyran-9-carboxamide (2β')

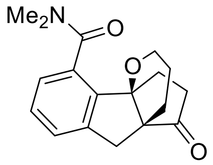

Prepared following General Procedure L from prochiral diketone **1β**. Reaction time: 72 h. Obtained as white solid. **^{1}H NMR** (500 MHz, DMSO, 100 ˚C) δ 6.92 – 6.73 (m, 2H), 6.61 (d, *J* = 7.3 Hz, 1H), 3.17 – 3.10 (m, 1H), 2.87 – 2.80 (m, 1H), 2.60 – 2.34 (m, 7H), 2.15 (d, *J* = 16.1 Hz, 1H), 2.09 – 1.99 (m, 2H), 1.92 – 1.84 (m, 1H), 1.68 – 1.62 (m, 1H), 1.60 – 1.51 (m, 1H), 0.91 – 0.74 (m, 3H) ppm. **^{13}C NMR** (126 MHz, DMSO, 100 ˚C) δ 218.0 (CO), 168.3 (CO), 140.4 (C), 139.6 (C), 133.1 (C), 127.7 (CH), 125.5 (CH), 124.1 (CH), 90.8 (C), 61.8 (CH_2), 58.1(C), 38.2 (CH_2), 38.1 (CH_2), 37.2 (CH_3 amide), 33.6 (CH_2), 31.7 (CH_2), 26.1 (CH_2), 21.6 (CH_2) ppm.. Note: The low intensity of certain signals is presumably attributable to rotamerism. **HRMS** [APCI]: *m/z* calculated for $C_{18}H_{22}NO_3$ $[M + H]^+$: 300.1594, found 300.1592. **MP** = 144.2 – 145.6 °C

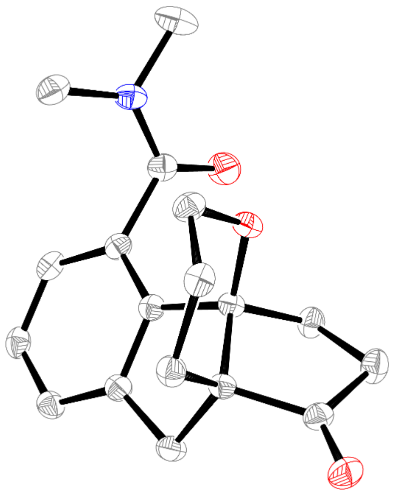

Figure S7. X-ray structure of **2β'** (CCDC 2488405)

Enantioselectivity was determined by chiral HPLC analysis on a Chiralpack IG3 column at rt (Hexane:*i*PrOH = 95:5, 1 mL/min).

A: Racemic sample prepared using $[Ir(COD)_2]BAr^F{}_4$ / *rac*-Binap (10 mol%) at 130 ˚C.

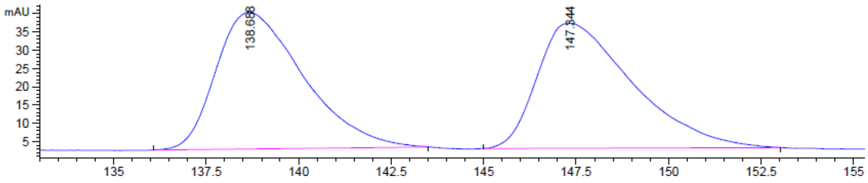

Signal 1: DAD1 A, Sig=220,4 Ref=off

Peak #	RetTime [min]	Type	Width [min]	Area [mAU*s]	Height [mAU]	Area %
1	138.688	BB	1.9331	6136.45898	37.46124	50.0427
2	147.344	BB	2.1025	6125.98047	34.18826	49.9573
Totals :				1.22624e4	71.64950	

B: Asymmetric sample prepared using $[Ir(COD)_2]BAr^F{}_4$ / *(R)*-DTBM-Segphos (10 mol%) at 130 ˚C (42% yield, 93:7 er).

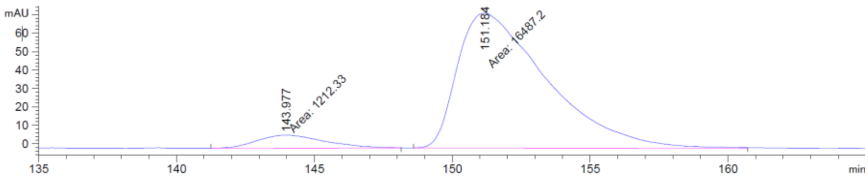

Signal 1: DAD1 A, Sig=220,4 Ref=off

Peak #	RetTime [min]	Type	Width [min]	Area [mAU*s]	Height [mAU]	Area %
1	143.977	MM	2.8617	1212.33228	7.06061	6.8495
2	151.184	MM	3.7514	1.64872e4	73.24952	93.1505
Totals :				1.76995e4	80.31013	

(4a*S*,11a*S*)-11a-methyl-3,4,11,11a-tetrahydro-2*H*,6*H*-phenanthro[4b,4-*bc*]furan-1,6(10*H*)-dione (2γ')

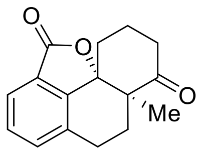

Prepared following General Procedure L from prochiral diketone **1γ**. Reaction time: 72 h. Obtained as colorless solid. ^{1}**H NMR** (500 MHz, $CDCl_3$) δ 7.68 (dd, *J* = 7.5, 0.9 Hz, 1H), 7.48 (t, *J* = 7.5 Hz, 1H), 7.41 (dd, *J* = 7.5, 1.1 Hz, 1H), 3.23 – 3.12 (m, 1H), 2.92 – 2.71 (m, 2H), 2.62 – 2.28 (m, 4H), 2.13 – 2.02 (m, 1H), 1.84 – 1.72 (m, 2H), 0.67 (s, 3H) ppm. ^{13}**C NMR** (126 MHz, $CDCl_3$) δ 211.17 (CO), 169.49 (CO), 150.04 (C), 132.60 (C), 132.55 (CH), 130.41 (CH), 124.90 (C), 123.76 (CH), 89.54 (C), 50.31 (CH), 36.58 (CH_2), 34.91 (CH_2), 31.36 (CH_2), 22.71 (CH_2), 21.35 (CH_2), 14.33 (CH_3) ppm. **HRMS** [APCI]: *m/z* calculated for $C_{16}H_{17}O_3$ $[M + H]^+$: 257.1172, found 257.1170. **MP** = 174.2 – 175.8 °C

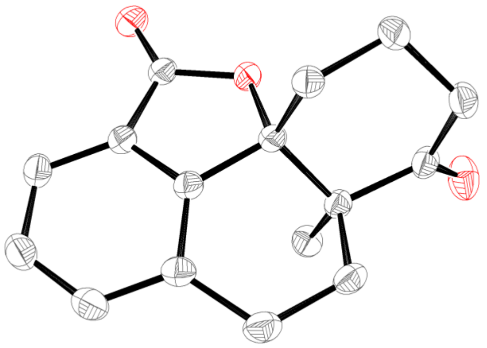

Figure S8. X-ray structure of racemic **2γ'** (CCDC 2503232)

Enantioselectivity was determined by chiral HPLC analysis on a Chiralpack IE3 column at rt (Hexane:*i*PrOH = 90:10, 1 mL/min).

A: Racemic sample prepared using $[Ir(COD)_2]BAr^F_4$ / *rac*-Binap (10 mol%) at 130 ˚C.

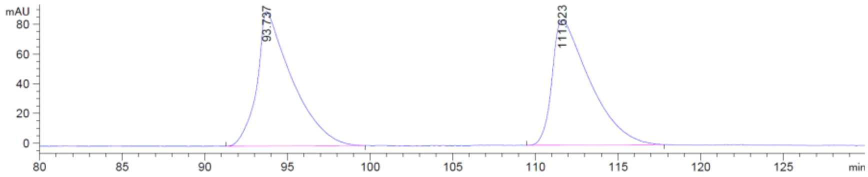

Signal 3: DAD1 C, Sig=210,4 Ref=off

Peak #	RetTime [min]	Type	Width [min]	Area [mAU*s]	Height [mAU]	Area %
1	93.737	BB	1.8897	1.33836e4	90.58768	50.0241
2	111.623	BB	2.1021	1.33708e4	84.22549	49.9759
Totals :				2.67544e4	174.81318	

B: Asymmetric sample prepared using $[Ir(COD)_2]BAr^F_4$ / *(S)*-C_3-Tunephos (10 mol%) at 130 ˚C (71% yield, 77:23 er).

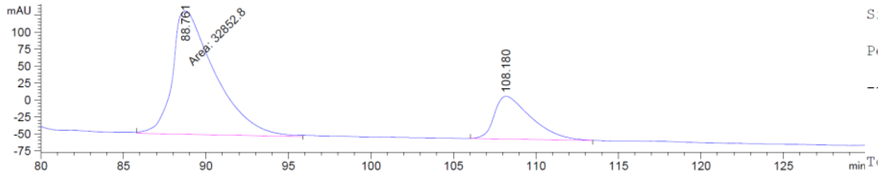

Signal 3: DAD1 C, Sig=210,4 Ref=off

Peak #	RetTime [min]	Type	Width [min]	Area [mAU*s]	Height [mAU]	Area %
1	88.761	MM	3.0039	3.28528e4	182.27698	77.1435
2	108.180	BB	1.8265	9733.80859	63.21209	22.8565
Totals :				4.25866e4	245.48907	

(3aS,10a*S*)-10a-methyl-2,3,10,10a-tetrahydro-5*H*-cyclopenta[1,2]naphtho[1,8-*bc*]furan-1,5(9*H*)-dione (2δ')

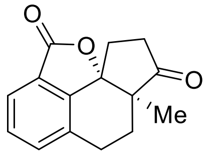

Prepared following General Procedure L from prochiral diketone **1δ**. Reaction time: 72 h. Obtained as colorless oil. ^{1}**H NMR** (500 MHz, $CDCl_3$) δ 7.72 (dd, *J* = 7.5, 0.7 Hz, 1H), 7.50 (t, *J* = 7.5 Hz, 1H), 7.45 (dd, *J* = 7.4, 0.9 Hz, 1H), 2.88 – 2.75 (m, 3H), 2.63 – 2.54 (m, 1H), 2.36 – 2.24 (m, 3H), 1.51 – 1.44 (m, 1H), 0.88 (s, 3H) ppm. ^{13}**C NMR** (126 MHz, $CDCl_3$) δ 218.87 (CO), 169.54 (CO), 149.28 (C), 134.24 (C), 131.98 (CH), 130.43 (CH), 125.01 (C), 123.63 (CH), 90.27 (C), 52.03 (C), 35.82 (CH_2), 34.30 (CH_2),

33.83 (CH_2), 24.83 (CH_2), 17.07 (CH_3) ppm. **HRMS** [APCI]: *m/z* calculated for $C_{15}H_{15}O_3$ $[M + H]^+$: 243.1016, found 243.1007.

Enantioselectivity was determined by chiral HPLC analysis on a Chiralpack IF3 column at rt (Hexane:*i*PrOH = 90:10, 1 mL/min).

A: Racemic sample prepared using $[Ir(COD)_2]BAr^F_4$ / *rac*-Binap (10 mol%) at 130 ˚C.

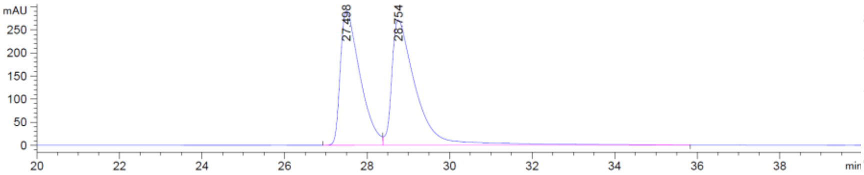

Signal 1: DAD1 A, Sig=220,4 Ref=off

Peak #	RetTime [min]	Type	Width [min]	Area [mAU*s]	Height [mAU]	Area %
1	27.498	BV	0.4947	9583.13281	290.61151	46.3353
2	28.754	VB	0.5879	1.10990e4	270.96899	53.6647
Totals :				2.06822e4	561.58051	

B: Asymmetric sample prepared using $[Ir(COD)_2]BAr^F_4$ / *(S)*-C_3-Tunephos (10 mol%) at 130 ˚C (55% yield, 84:16 er).

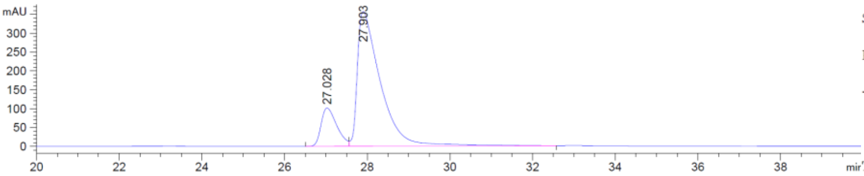

Signal 1: DAD1 A, Sig=220,4 Ref=off

Peak #	RetTime [min]	Type	Width [min]	Area [mAU*s]	Height [mAU]	Area %
1	27.028	BV	0.4043	2682.13525	101.49012	16.2437
2	27.903	VB	0.5640	1.38297e4	356.94693	83.7563
Totals :				1.65118e4	458.43705	

(3a*S*,12b*S*)-12b-methyl-2,3-dihydro-5*H*-cyclopenta[9,10]phenanthro[10,1-*bc*]furan-1,5(12bH)-dione (2ε')

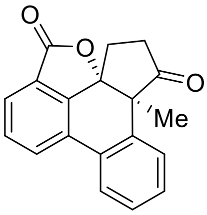

Prepared following General Procedure L from prochiral diketone **1ε**. Reaction time: 72 h. Obtained a brown solid. **¹H NMR** (500 MHz, $CDCl_3$) δ 7.94 (dd, *J* = 7.2, 2.0 Hz, 2H), 7.84 (d, *J* = 7.6 Hz, 1H), 7.68 (t, *J* = 7.6 Hz, 1H), 7.48 – 7.33 (m, 3H), 2.75 (ddd, *J* = 19.4, 11.5, 9.1 Hz, 1H), 2.53 (ddd, *J* = 19.4, 9.6, 1.5 Hz, 1H), 2.36 (ddd, *J* = 14.1, 11.5, 9.6 Hz, 1H), 2.18 (ddd, *J* = 14.1, 9.1, 1.4 Hz, 1H), 0.98 (s, 3H) ppm. **¹³C NMR** (126 MHz, $CDCl_3$) δ 215.4 (CO), 169.1 (CO), 146.8 (C), 136.1 (C), 131.4 (CH), 130.4 (C), 130.2 (CH), 129.9 (C), 129.0 (CH), 127.9 (CH), 127.9 (CH), 125.5 (C), 124.8 (CH), 124.6 (CH), 90.8 (CO), 58.7 (C), 34.8 (CH_2), 31.6 (CH_2), 15.8 (CH_3) ppm. **HRMS** [APCI]: *m/z* calculated for $C_{19}H_{15}O_3$ $[M + H]^+$: 291.1016, found 291.1024. **MP** = 190.2 – 191.9 °C

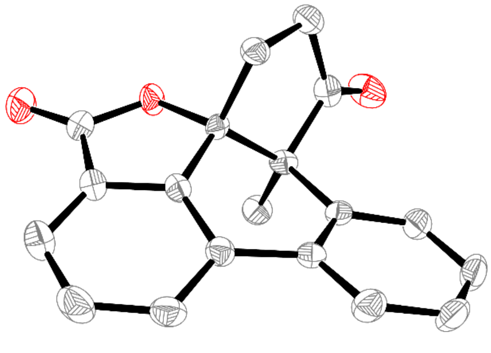

Figure S9. X-ray structure of **2ε'** (CCDC 2504174)

Enantioselectivity was determined by chiral HPLC analysis on a Chiralpack IE3 column at rt (Hexane:*i*PrOH = 90:10, 1 mL/min).

A: Racemic sample prepared using [Ir(COD)$_2$]BAr$^F_4$ / *rac*-Binap (10 mol%) at 130 ˚C.

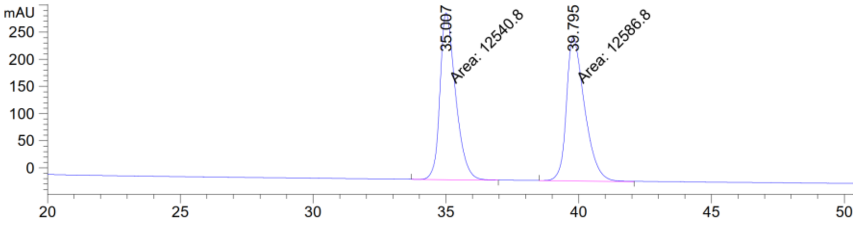

Signal 1: DAD1 A, Sig=220,4 Ref=off

Peak #	RetTime [min]	Type	Width [min]	Area [mAU*s]	Height [mAU]	Area %
1	35.007	MM	0.6802	1.25408e4	307.27603	49.9086
2	39.795	MM	0.7953	1.25868e4	263.76794	50.0914
Totals :				2.51276e4	571.04398	

B: Asymmetric sample prepared using [Ir(COD)$_2$]BAr$^F_4$ / *(R)*-Segphos (10 mol%) at 130 ˚C (56% yield, 99:1 er).

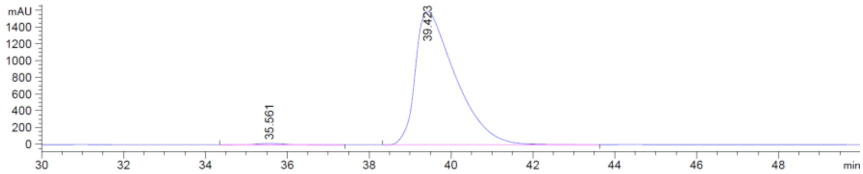

Signal 1: DAD1 A, Sig=220,4 Ref=off

Peak #	RetTime [min]	Type	Width [min]	Area [mAU*s]	Height [mAU]	Area %
1	35.561	BB	0.6244	670.82025	16.42747	0.6166
2	39.423	BB	0.9888	1.08123e5	1591.18787	99.3834
Totals :				1.08793e5	1607.61533	

(4a*R*,12a*R*)-*N,N*-dimethyl-1-oxo-1,2,3,4,4a,5,6,12-octahydrobenzo[*j*]fluorene-8-carboxamide (5a)

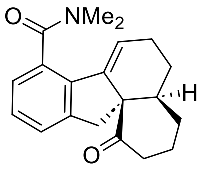

Prepared following General Procedure L from prochiral diketone **4a**. Reaction time: 48 h. Obtained as white solid. **^{1}H NMR** (500 MHz, CDCl$_3$) δ 7.14 (d, *J* = 4.6 Hz, 2H), 7.06 (q, *J* = 4.5 Hz, 1H), 6.05 (t, *J* = 3.7 Hz, 1H), 3.30 (d, *J* = 15.9 Hz, 1H), 3.13 (s, 3H), 3.06 (d, *J* = 15.9 Hz, 1H), 2.99 (s, 3H), 2.87 – 2.78 (m, 1H), 2.35 – 2.08 (m, 5H), 2.01 – 1.95 (m, 2H), 1.77 – 1.69 (m, 1H), 1.58 – 1.48 (m, 2H) ppm. **^{13}C NMR** (126 MHz, CDCl$_3$) δ 213.6 (CO), 171.1 (C), 140.7 (C), 140.5 (C), 137.3 (C), 130.5 (C), 127.7 (CH), 125.2 (CH), 125.0 (CH), 122.9 (CH), 62.2 (C), 42.6 (CH$_2$), 42.5 (CH), 38.7 (CH$_3$ amide), 37.5 (CH$_2$), 35.1 (CH$_3$ amide), 27.8 (CH$_2$), 25.9 (CH$_2$), 24.5 (CH$_2$), 22.6 (CH$_2$) ppm. **HRMS** [APCI]: *m/z* calculated for $C_{20}H_{24}NO_2$ [M + H]$^+$: 310.1802, found 310.1801. **MP** = 170.2 – 171.2 °C

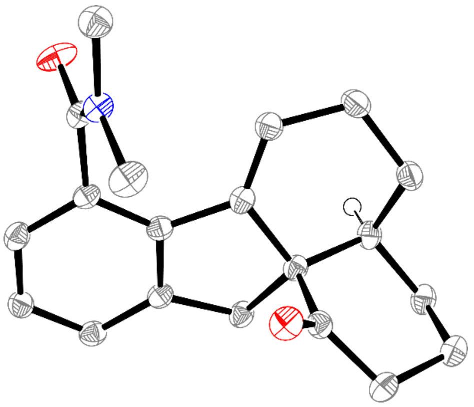

Figure S10. X-ray structure of **5a** (CCDC 2504168)

Enantioselectivity was determined by chiral HPLC analysis on a Chiralpack IF3 column at rt (Hexane:*i*PrOH = 85:15, 1 mL/min).

A: Racemic sample prepared using $[Ir(COD)_2]BAr^F_4$ / *rac*-Binap (10 mol%) at 130 ˚C.

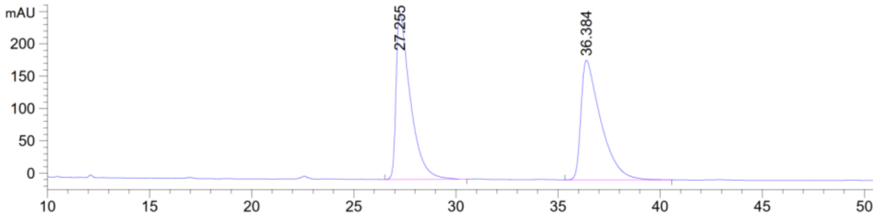

Signal 1: DAD1 A, Sig=220,4 Ref=off

Peak #	RetTime [min]	Type	Width [min]	Area [mAU*s]	Height [mAU]	Area %
1	27.255	BB	0.6897	1.24439e4	257.53754	49.8421
2	36.384	BB	0.9455	1.25227e4	185.04274	50.1579
Totals :				2.49666e4	442.58028	

B: Asymmetric sample prepared using $[Ir(COD)_2]BAr^F_4$ / *(R)*-DTBM-Segphos (10 mol%) at 130 ˚C (91% yield, 98:2 er).

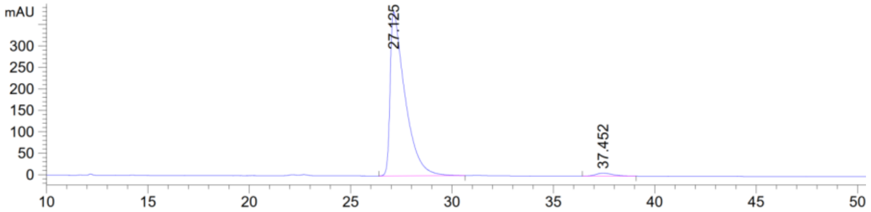

Signal 1: DAD1 A, Sig=220,4 Ref=off

Peak #	RetTime [min]	Type	Width [min]	Area [mAU*s]	Height [mAU]	Area %
1	27.125	BB	0.7276	1.94408e4	382.07645	97.9445
2	37.452	BB	0.7044	407.99713	6.95517	2.0555
Totals :				1.98488e4	389.03162	

C: After recrystallization from a sample with 98:2 er: >99.9: 0.1 er

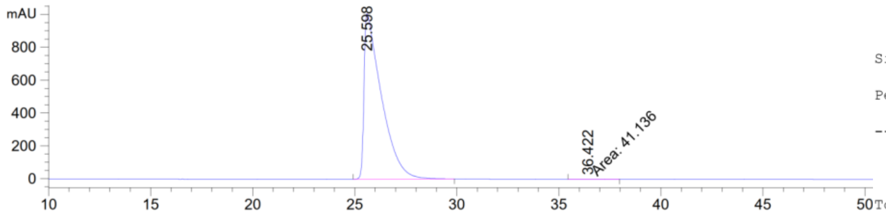

Signal 1: DAD1 A, Sig=220,4 Ref=off

Peak #	RetTime [min]	Type	Width [min]	Area [mAU*s]	Height [mAU]	Area %
1	25.598	BB	0.7851	5.94419e4	1006.38306	99.9308
2	36.422	MM	0.9307	41.13601	7.36662e-1	0.0692
Totals :				5.94830e4	1007.11972	

(3a*R*,10a*R*)-*N,N*-dimethyl-1-oxo-1,2,3,3a,4,10-hexahydropentaleno[1,6a-*a*]indene-6-carboxamide (5b)

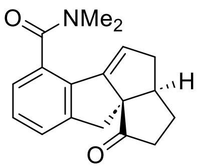

Prepared following General Procedure L from prochiral diketone **4b**. Reaction time: 36 h. Obtained as colorless oil. **^{1}H NMR** (500 MHz, $CDCl_3$) δ 7.29 – 7.23 (m, 2H), 7.16 – 7.13 (m, 1H), 5.69 (d, *J* = 3.5 Hz, 1H), 3.24 – 3.15 (m, 2H), 3.10 (s, 3H), 3.00 (d, *J* = 15.4 Hz, 1H), 2.82 (d, *J* = 15.5 Hz, 1H), 2.79 (s, 3H), 2.73 – 2.67 (m, 1H), 2.65 – 2.56 (m, 1H), 2.39 (dd, *J* = 18.0, 7.6 Hz, 1H), 2.11 – 2.02 (m, 1H), 1.96 (dd, *J* = 12.9, 8.6 Hz, 1H) ppm. **^{13}C NMR** (126 MHz, $CDCl_3$) δ 218.9 (CO), 170.5 (CO), 149.0 (C), 132.3 (C), 131.4 (C), 128.8 (CH), 126.1 (CH), 124.9 (CH), 122.6 (CH), 71.4 (C), 47.6 (CH), 45.2 (CH_2), 38.6 (CH_3 amide), 38.6 (CH_2), 36.8 (CH_2), 35.0 (CH_3 amide), 25.4 (CH_2) ppm. **HRMS** [APCI]: *m/z* calculated for $C_{18}H_{20}NO_2$ $[M + H]^+$: 282.1489, found 282.1485.

Enantioselectivity was determined by chiral HPLC analysis on a Chiralpack IF3 column at rt (Hexane:*i*PrOH = 85:15, 1 mL/min).

A: Racemic sample prepared using $[Ir(COD)_2]BAr^F_4$ / *rac*-Binap (10 mol%) at 130 ˚C.

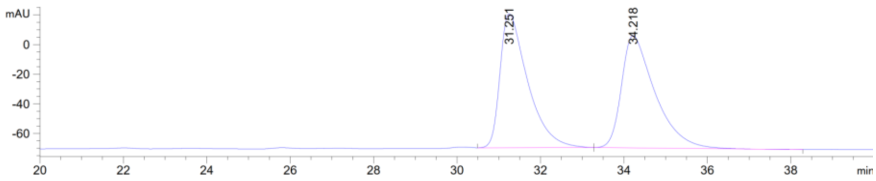

Signal 1: DAD1 A, Sig=220,4 Ref=off

Peak #	RetTime [min]	Type	Width [min]	Area [mAU*s]	Height [mAU]	Area %
1	31.251	BB	0.6798	4152.28271	90.40633	49.5818
2	34.218	BB	0.8206	4222.33105	75.71127	50.4182
Totals :				8374.61377	166.11760	

B: Asymmetric sample prepared using $[Ir(COD)_2]BAr^F{}_4$ / *(R)*-DTBM-Segphos (10 mol%) at 130 ˚C (83% yield, 97:3 er).

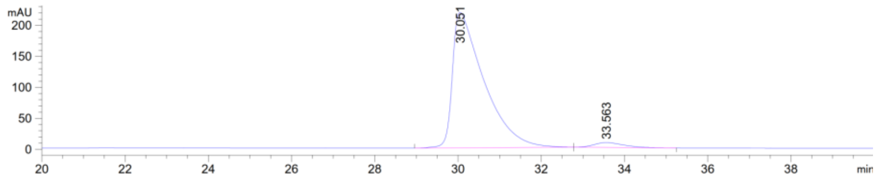

Signal 1: DAD1 A, Sig=220,4 Ref=off

Peak #	RetTime [min]	Type	Width [min]	Area [mAU*s]	Height [mAU]	Area %
1	30.051	BB	0.7664	2.41349e4	452.42578	96.7355
2	33.563	BB	0.7455	814.47418	16.22971	3.2645
Totals :				2.49493e4	468.65549	

(3a*S*,5a*R*,10a*R*)-5a-hydroxy-*N,N*-dimethyl-1-oxo-1,2,3,3a,4,5,5a,10-octahydropentaleno[1,6a-*a*]indene-6-6-carboxamide (6b)

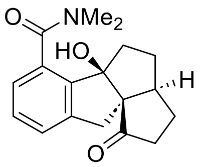

Prepared following General Procedure L from prochiral diketone **4b** in the presence of 1.25 equiv of Et_3SiH. Reaction time: 3 h. Obtained as colorless oil. **^{1}H NMR** (500 MHz, $CDCl_3$) δ 7.27 – 7.21 (m, 2H), 7.11 (d, *J* = 6.8 Hz, 1H), 3.65 (d, *J* = 17.0 Hz, 1H), 3.13 (s, 3H), 2.98 (s, 3H), 2.75 (d, *J* = 17.0 Hz, 1H), 2.67 – 2.56 (m, 2H), 2.36 – 2.24 (m, 2H), 2.18 – 2.06 (m, 2H), 1.84 – 1.71 (m, 3H) ppm. **^{13}C NMR** (126 MHz, $CDCl_3$) δ 219.8 (CO), 171.7 (CO), 144.1 (C), 143.9 (C), 131.7 (C), 128.2 (CH), 126.4 (CH), 126.0 (CH), 96.6 (COH), 68.0 (C), 50.5 (CH), 41.2 (CH_2), 40.6 (CH_2), 39.9 (CH_3 amide), 39.0 (CH_2), 35.4 (CH_3 amide), 32.4 (CH_2), 25.1 (CH_2) ppm. **HRMS** [APCI]: *m/z* calculated for $C_{18}H_{22}NO_3$ $[M + H]^+$: 300.1594, found 300.1594.

Enantioselectivity was determined by chiral HPLC analysis on a Chiralpack IF3 column at rt (Hexane:*i*PrOH = 85:15, 1 mL/min).

A: Racemic sample prepared using $[Ir(COD)_2]BAr^F{}_4$ / *rac*-Binap (10 mol%) at 130 ˚C.

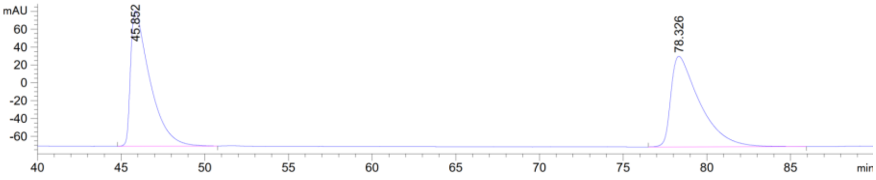

Signal 1: DAD1 A, Sig=220,4 Ref=off

Peak #	RetTime [min]	Type	Width [min]	Area [mAU*s]	Height [mAU]	Area %
1	45.852	BB	1.1304	1.21247e4	151.71335	49.9739
2	78.326	BB	1.6633	1.21374e4	101.34016	50.0261
Totals :				2.42621e4	253.05351	

B: Asymmetric sample prepared using $[Ir(COD)_2]BAr^F{}_4$ / *(R)*-DTBM-Segphos (10 mol%) at 130 ˚C, 4h (40% yield, 97:3 er).

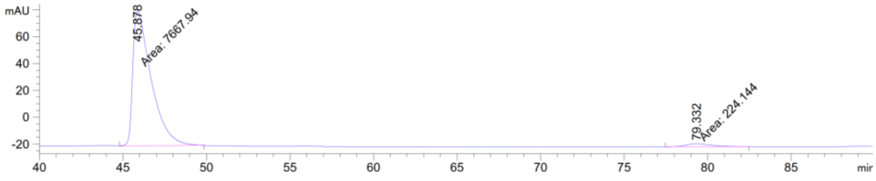

Signal 1: DAD1 A, Sig=220,4 Ref=off

Peak #	RetTime [min]	Type	Width [min]	Area [mAU*s]	Height [mAU]	Area %
1	45.878	MM	1.2716	7667.94287	100.50104	97.1599
2	79.332	MM	1.7467	224.14389	2.13872	2.8401
Totals :				7892.08676	102.63976	

C: Asymmetric sample prepared using $[Ir(COD)_2]BAr^F{}_4$ / *(R)*-DTBM-Segphos (10 mol%) in the presence of 1.25 equiv of Et_3SiH at 130 ˚C, reaction time: 3 h (87% yield, 89:11 er).

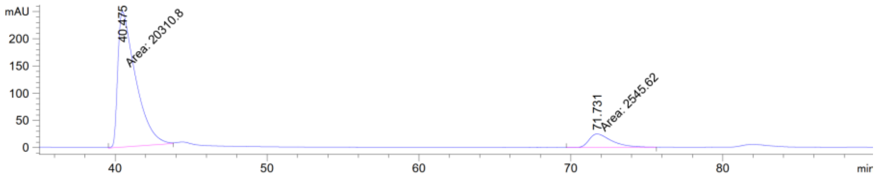

Signal 1: DAD1 A, Sig=220,4 Ref=off

Peak #	RetTime [min]	Type	Width [min]	Area [mAU*s]	Height [mAU]	Area %
1	40.475	MM	1.3604	2.03108e4	248.83266	88.8625
2	71.731	MM	1.7187	2545.62329	24.68538	11.1375
Totals :				2.28564e4	273.51803	

(*R*)-*N,N*,10a-trimethyl-1-oxo-2,3,10,10a-tetrahydroindeno[1,2-*b*]indole-5(1*H*)-carboxamide (8a)

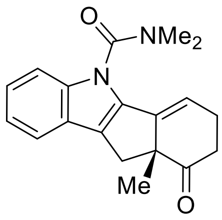

Prepared following General Procedure L from prochiral diketone **7a**. Obtained as yellow oil. **^{1}H NMR** (300 MHz, $CDCl_3$) δ 7.50 (d, *J* = 7.9 Hz, 1H), 7.44 (d, *J* = 8.3 Hz, 1H), 7.33 – 7.15 (m, 2H), 6.08 (d, *J* = 6.4 Hz, 1H), 3.38 (d, *J* = 16.0 Hz, 1H), 3.13 (s, 3H), 3.07 (s, 3H), 2.89 – 2.69 (m, 3H), 2.66 – 2.29 (m, 2H), 1.49 (s, 3H) ppm. **^{13}C NMR** (75 MHz, $CDCl_3$) δ 214.19 (CO), 154.05 (CO), 141.10 (C), 138.91 (C), 138.52 (C), 125.73 (C), 125.44 (C), 124.19 (CH), 121.96 (CH), 119.98 (CH), 115.26 (CH), 113.13 (CH), 60.09 (C), 38.30 (CH_3 urea), 37.89 (CH_3 urea), 35.94 (CH_2), 33.81 (CH_2), 25.01 (CH_3), 23.23 (CH_2) ppm. **HRMS** [APCI]: *m/z* calculated for $C_{19}H_{21}N_2O_2$ $[M + H]^+$: 309.1598, found 309.1589.

Enantioselectivity was determined by chiral HPLC analysis on a Chiralpack IA3 column at rt (Hexane:*i*PrOH = 98:2, 1 mL/min).

A: Racemic sample prepared using $[Ir(COD)_2]BAr^F{}_4$ / *rac*-Binap (10 mol%) at 130 ˚C.

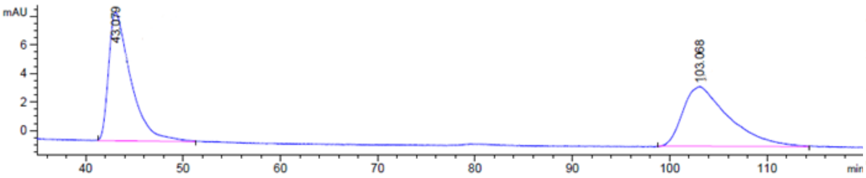

Signal 4: DAD1 D, Sig=300,4 Ref=off

Peak #	RetTime [min]	Type	Width [min]	Area [mAU*s]	Height [mAU]	Area %
1	43.079	MM	2.6383	1420.53577	8.97381	50.9874
2	103.068	MM	5.4466	1365.51575	4.17849	49.0126
Totals :				2786.05151	13.15230	

B: Asymmetric sample prepared using $[Ir(COD)_2]BAr^F{}_4$ / *(R)*-DTBM-Segphos (10 mol%) at 130 ˚C for 48 h (Table S6, entry 1, 52% yield, 58:42 er).

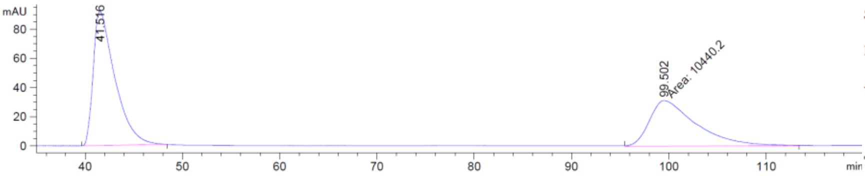

Signal 4: DAD1 D, Sig=300,4 Ref=off

Peak #	RetTime [min]	Type	Width [min]	Area [mAU*s]	Height [mAU]	Area %
1	41.516	BB	2.0967	1.42821e4	91.92036	57.7701
2	99.502	MM	5.5828	1.04402e4	31.16755	42.2299
Totals :				2.47223e4	123.08791	

C: Asymmetric sample prepared using $[Ir(COD)_2]BAr^F{}_4$ / *(R)*-Difluorphos (10 mol%) at 130 ˚C (Table S6, entry 2, 38% yield, 63:37 er).

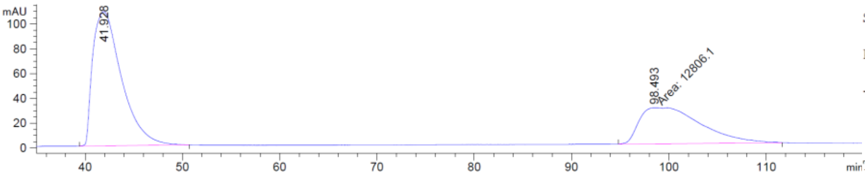

Signal 4: DAD1 D, Sig=300,4 Ref=off

Peak #	RetTime [min]	Type	Width [min]	Area [mAU*s]	Height [mAU]	Area %
1	41.928	BB	2.4311	2.22037e4	108.24731	63.4214
2	98.493	MM	7.3308	1.28061e4	29.11487	36.5786
Totals :				3.50097e4	137.36217	

D: Asymmetric sample prepared using $[Ir(COD)_2]BAr^F{}_4$ / (*R*)-H8-Binap (10 mol%) at 130 ˚C (Table S6, entry 3, 60% yield, 65:35 er).

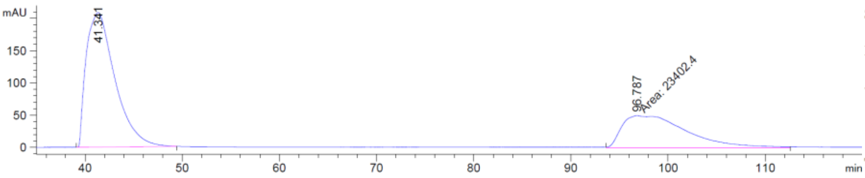

Signal 4: DAD1 D, Sig=300,4 Ref=off

Peak #	RetTime [min]	Type	Width [min]	Area [mAU*s]	Height [mAU]	Area %
1	41.341	BB	2.5193	4.27782e4	208.53081	64.6386
2	96.787	MM	7.7663	2.34024e4	50.22205	35.3614
Totals :				6.61807e4	258.75285	

E: <u>Asymmetric sample</u> prepared using [Ir(COD)$_2$]BAr$^F{}_4$ / *(R)*-Ph-Garphos (10 mol%) at 130 ˚C (Table S6, entry 4, 49% yield, 74:26 er).

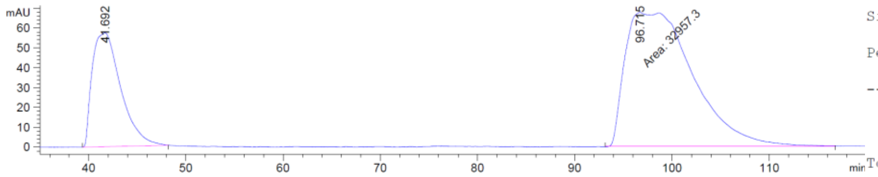

Signal 4: DAD1 D, Sig=300,4 Ref=off

Peak #	RetTime [min]	Type	Width [min]	Area [mAU*s]	Height [mAU]	Area %
1	41.692	BB	2.3943	1.16992e4	57.43719	26.1982
2	96.715	MM	8.1333	3.29573e4	67.53561	73.8018
Totals :				4.46565e4	124.97279	

F: <u>Asymmetric sample</u> prepared using [Ir(COD)$_2$]BAr$^F{}_4$ / *(R)*-BTFM-Garphos (10 mol%) at 130 ˚C (Table S6, entry 5, 39% yield, 60:40 er).

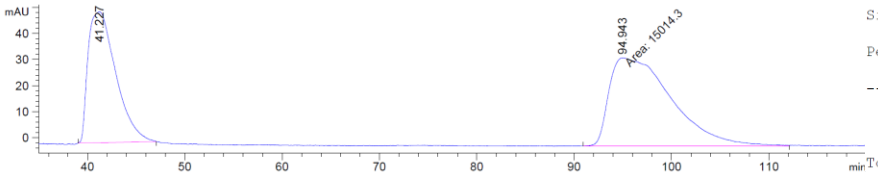

Signal 4: DAD1 D, Sig=300,4 Ref=off

Peak #	RetTime [min]	Type	Width [min]	Area [mAU*s]	Height [mAU]	Area %
1	41.227	BB	2.3099	9936.86914	50.27471	39.8253
2	94.943	MM	7.3952	1.50143e4	33.83788	60.1747
Totals :				2.49511e4	84.11259	

G: <u>Asymmetric sample</u> prepared using [Ir(COD)$_2$]BAr$^F{}_4$ / *(R)*-MeO-Biphep (10 mol%) at 130 ˚C (Table S6, entry 6, 68% yield, 74:26 er).

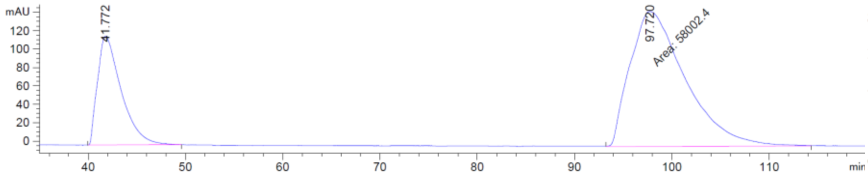

Signal 4: DAD1 D, Sig=300,4 Ref=off

Peak #	RetTime [min]	Type	Width [min]	Area [mAU*s]	Height [mAU]	Area %
1	41.772	BB	2.0471	2.00015e4	118.50726	25.6417
2	97.720	MM	6.5787	5.80024e4	146.94580	74.3583
Totals :				7.80040e4	265.45306	

H: <u>Asymmetric sample</u> prepared using [Ir(COD)$_2$]BAr$^F{}_4$ / *(S)*-C$_1$-Tunephos (10 mol%) at 130 ˚C (Table S6, entry 7, 92% yield, 72:28 er).

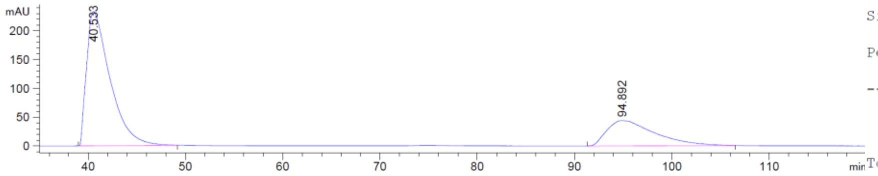

Signal 4: DAD1 D, Sig=300,4 Ref=off

Peak #	RetTime [min]	Type	Width [min]	Area [mAU*s]	Height [mAU]	Area %
1	40.533	BB	2.1708	3.89314e4	233.06702	72.1591
2	94.892	BB	3.9924	1.50208e4	44.02663	27.8409
Totals :				5.39522e4	277.09365	

I: <u>Asymmetric sample</u> prepared using [Ir(COD)$_2$]BAr$^F{}_4$ / *(S)*-C$_2$-Tunephos (10 mol%) at 130 ˚C (Table S6, entry 8, 92% yield, 73:27 er).

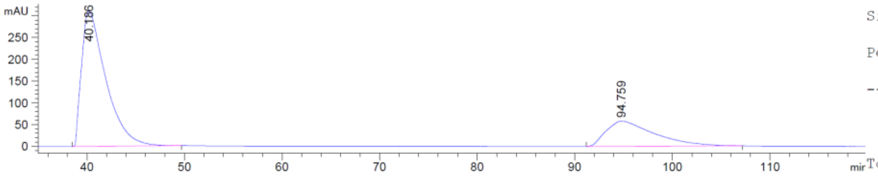

Signal 4: DAD1 D, Sig=300,4 Ref=off

Peak #	RetTime [min]	Type	Width [min]	Area [mAU*s]	Height [mAU]	Area %
1	40.186	BB	2.3711	5.35745e4	310.49051	73.0487
2	94.759	BB	4.0078	1.97663e4	57.74112	26.9513
Totals :				7.33408e4	368.23163	

J: <u>Asymmetric sample</u> prepared using [Ir(COD)$_2$]BAr$^F{}_4$ / *(S)*-C$_3$-Tunephos (10 mol%) at 130 ˚C (Table S6, entry 9, 84% yield, 80:20 er).

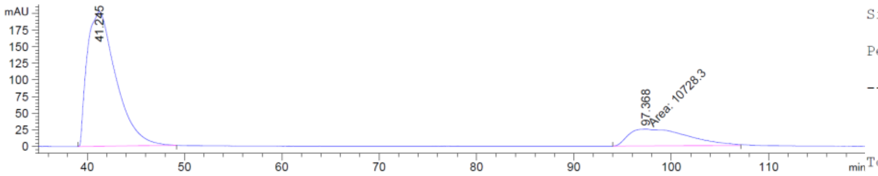

Signal 4: DAD1 D, Sig=300,4 Ref=off

Peak #	RetTime [min]	Type	Width [min]	Area [mAU*s]	Height [mAU]	Area %
1	41.245	BB	2.5065	4.15806e4	202.25739	79.4904
2	97.368	MM	7.1045	1.07283e4	25.16793	20.5096
Totals :				5.23090e4	227.42531	

K: Asymmetric sample prepared using [Ir(COD)$_2$]BAr^F$_4$ / *(S)*-C$_5$-Tunephos (10 mol%) at 130 ˚C (Table S6, entry 10, 78% yield, 75:25 er).

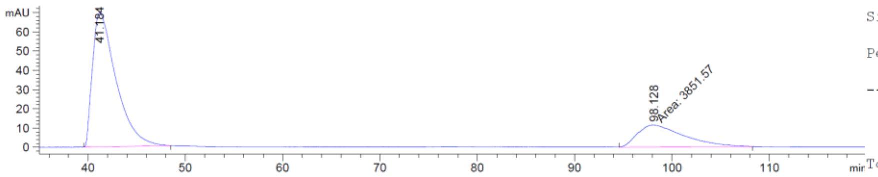

Signal 4: DAD1 D, Sig=300,4 Ref=off

Peak #	RetTime [min]	Type	Width [min]	Area [mAU*s]	Height [mAU]	Area %
1	41.184	BB	2.1141	1.17256e4	69.57707	75.2742
2	98.128	MM	5.5877	3851.56519	11.48816	24.7258
Totals :				1.55771e4	81.06523	

L: Asymmetric sample prepared using [Ir(COD)$_2$]BAr^F$_4$ / *(R,R)*-QuinoxP (10 mol%) at 130 ˚C (Table S6, entry 11, 59% yield, 68:32 er).

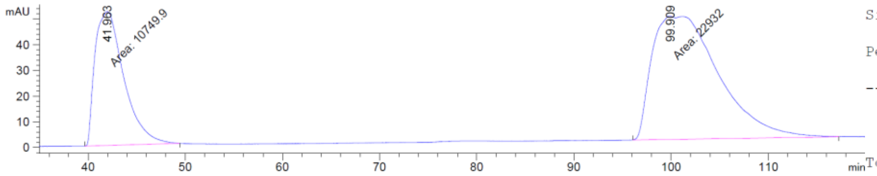

Signal 4: DAD1 D, Sig=300,4 Ref=off

Peak #	RetTime [min]	Type	Width [min]	Area [mAU*s]	Height [mAU]	Area %
1	41.963	MM	3.4407	1.07499e4	52.07255	31.9160
2	99.909	MM	7.9588	2.29320e4	48.02209	68.0840
Totals :				3.36819e4	100.09465	

M: Asymmetric sample prepared using [Ir(COD)$_2$]BAr^F$_4$ / *(R,R)*-BenzP (10 mol%) at 130 ˚C (Table S6, entry 12, 84% yield, 86:14 er).

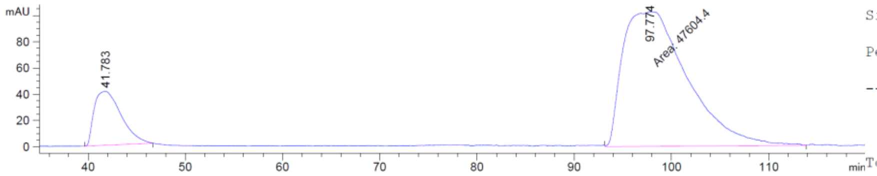

Signal 4: DAD1 D, Sig=300,4 Ref=off

Peak #	RetTime [min]	Type	Width [min]	Area [mAU*s]	Height [mAU]	Area %
1	41.783	BB	2.2608	7925.74658	41.15555	14.2729
2	97.774	MM	7.7247	4.76044e4	102.71022	85.7271
Totals :				5.55302e4	143.86577	

N: Asymmetric sample prepared using [Ir(COD)$_2$]BAr^F$_4$ / *(R,R)*-BenzP (10 mol%) at 115 ˚C (Table S6, entry 13, 31% yield, 93:7 er).

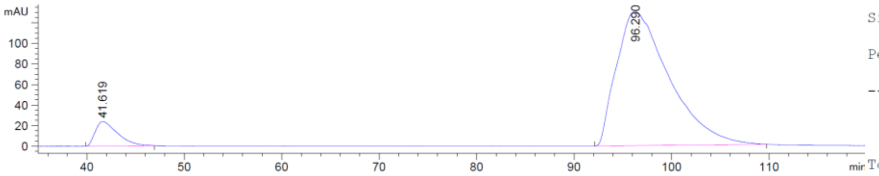

Signal 4: DAD1 D, Sig=300,4 Ref=off

Peak #	RetTime [min]	Type	Width [min]	Area [mAU*s]	Height [mAU]	Area %
1	41.619	BB	1.8427	3746.54126	23.82387	7.1315
2	96.290	BB	4.3745	4.87889e4	130.29588	92.8685
Totals :				5.25354e4	154.11976	

O: Asymmetric sample prepared using [Ir(COD)$_2$]BAr^F$_4$ / *(R,R)-i*Pr-Duphos (10 mol%) at 130 ˚C (Table S6, entry 16, 67% yield, 89:11 er).

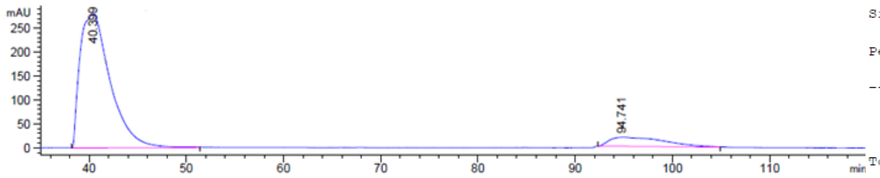

Signal 4: DAD1 D, Sig=300,4 Ref=off

Peak #	RetTime [min]	Type	Width [min]	Area [mAU*s]	Height [mAU]	Area %
1	40.399	MM	3.5873	6.05683e4	281.39853	89.0679
2	94.741	MM	6.5144	7434.12256	19.01973	10.9321
Totals :				6.80024e4	300.41826	

P: Asymmetric sample prepared using [Ir(COD)$_2$]BAr^F$_4$ / *(R,R)*-Et-Duphos (10 mol%) at 130 ˚C (Table S6, entry 17, 71% yield, 62:38 er).

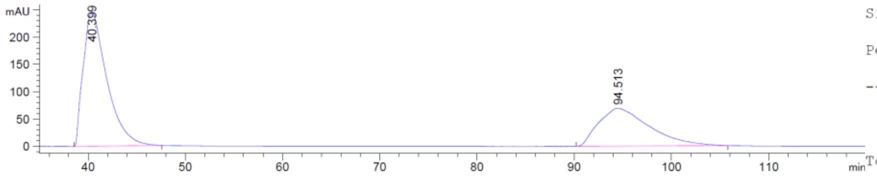

Signal 4: DAD1 D, Sig=300,4 Ref=off

Peak #	RetTime [min]	Type	Width [min]	Area [mAU*s]	Height [mAU]	Area %
1	40.399	BB	2.2353	4.13252e4	246.07521	62.2211
2	94.513	BB	4.2582	2.50915e4	68.97205	37.7789
Totals :				6.64167e4	315.04726	

Q: Asymmetric sample prepared using $[Ir(COD)_2]BAr^F{}_4$ / Josiphos SL-J002-1 (10 mol%) at 130 ˚C (Table S6, entry 19, 16% yield, 73:27 er).

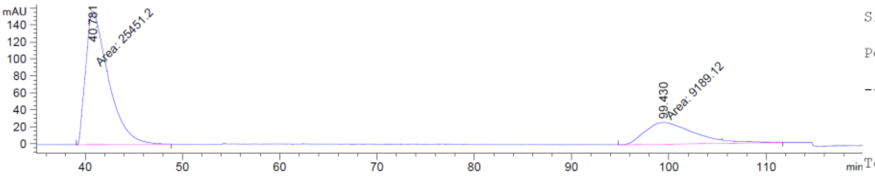

Signal 4: DAD1 D, Sig=300,4 Ref=off

Peak #	RetTime [min]	Type	Width [min]	Area [mAU*s]	Height [mAU]	Area %
1	40.781	MM	2.6977	2.54512e4	157.24203	73.4727
2	99.430	MM	5.9085	9189.12012	25.92055	26.5273
Totals :				3.46403e4	183.16259	

R: Asymmetric sample prepared using $[Ir(COD)_2]BAr^F{}_4$ / Josiphos SL-J502-2 (10 mol%) at 130 ˚C (Table S6, entry 20, 36% yield, 75:25 er).

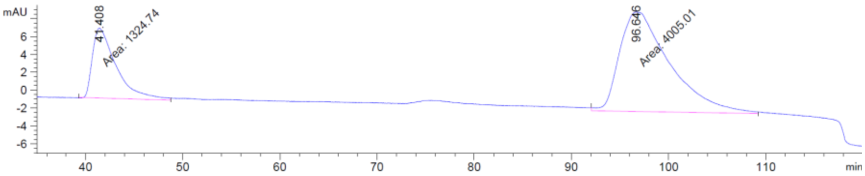

Signal 4: DAD1 D, Sig=300,4 Ref=off

Peak #	RetTime [min]	Type	Width [min]	Area [mAU*s]	Height [mAU]	Area %
1	41.408	MM	2.8247	1324.74487	7.81635	24.8556
2	96.646	MM	5.9993	4005.01489	11.12640	75.1444
Totals :				5329.75977	18.94275	

S: Asymmetric sample prepared using $[Ir(COD)_2]BAr^F{}_4$ / *(S,S)*-f-Binaphane (10 mol%) at 130 ˚C (Table S6, entry 21, 22% yield, 66:34 er).

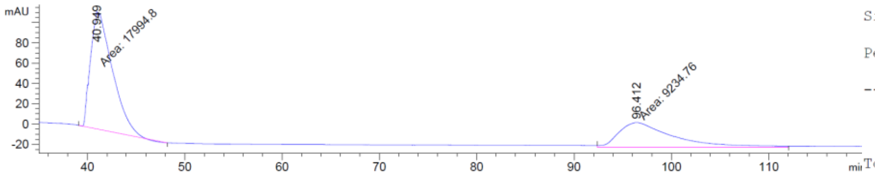

Signal 4: DAD1 D, Sig=300,4 Ref=off

Peak #	RetTime [min]	Type	Width [min]	Area [mAU*s]	Height [mAU]	Area %
1	40.949	MM	2.5933	1.79948e4	115.65122	66.0856
2	96.412	MM	6.3712	9234.76270	24.15744	33.9144
Totals :				2.72296e4	139.80867	

(*R*)-10a-methyl-5-(piperidine-1-carbonyl)-3,5,10,10a-tetrahydroindeno[1,2-*b*]indol-1(2*H*)-one (8aˡ)

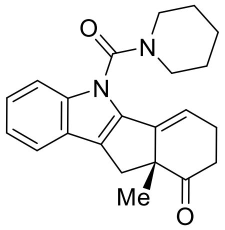

Prepared following General Procedure L from prochiral diketone **7aˡ**. Obtained as yellow oil. **^{1}H NMR** (300 MHz, $CDCl_3$) δ 7.50 – 7.41 (m, 1H), 7.30 – 7.18 (m, 1H), 7.20 – 7.13 (m, 1H), 6.08 (dd, *J* = 7.0, 3.0 Hz, 1H), 3.68 – 3.40 (m, 4H), 3.35 (d, *J* = 16.1 Hz, 1H), 2.97 – 2.69 (m, 2H), 2.70 – 2.65 (m, 1H), 2.64 – 2.50 (m, 1H), 2.41 – 2.28 (m, 1H), 1.75 – 1.58 (m, 6H), 1.47 (s, 3H) ppm. **^{13}C NMR** (75 MHz, $CDCl_3$) δ 214.31 (CO), 152.88 (CO), 141.17 (C), 138.75 (C), 125.60 (C), 125.44 (C), 124.07 (CH), 121.92 (CH), 119.97 (CH), 115.57 (CH), 112.87 (CH), 60.12 (C), 47.63 (CH_2), 35.95 (CH_2), 33.81 (CH_2), 26.24 (CH_2), 24.98 (CH_3), 24.53 (CH_2), 23.24 (CH_2) ppm. **HRMS** [APCI]: *m/z* calculated for $C_{22}H_{25}N_2O_2$ $[M + H]^+$: 349.1911, found 349.1909.

Enantioselectivity was determined by chiral HPLC analysis on a Chiralpack IA3 column at rt (Hexane:*i*PrOH = 95:5, 1 mL/min).

A: Racemic sample prepared using $[Ir(COD)_2]BAr^F{}_4$ / *rac*-Binap (10 mol%) at 130 ˚C.

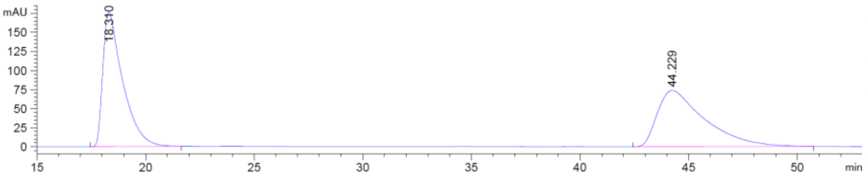

Signal 4: DAD1 D, Sig=300,4 Ref=off

Peak #	RetTime [min]	Type	Width [min]	Area [mAU*s]	Height [mAU]	Area %
1	18.310	BB	0.9039	1.11485e4	176.75247	50.0095
2	44.229	BB	1.8434	1.11442e4	73.81457	49.9905
Totals :				2.22927e4	250.56704	

B: Asymmetric sample prepared using $[Ir(COD)_2]BAr^F{}_4$ / *(R,R)*-BenzP (10 mol%) at 130 ˚C (Table S6, entry 26, 80% yield, 66:34 er).

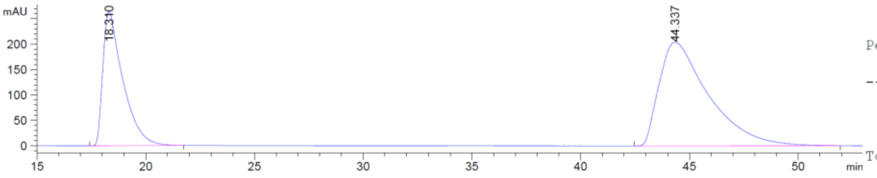

Peak #	RetTime [min]	Type	Width [min]	Area [mAU*s]	Height [mAU]	Area %
1	18.310	BB	0.9126	1.68454e4	264.64050	34.2555
2	44.337	BB	2.2636	3.23304e4	204.59514	65.7445
Totals :				4.91758e4	469.23564	

(*R*)-*N,N*,4a-trimethyl-4-oxo-3,4,4a,5-tetrahydro-10*H*-pyrano[4',3':4,5]cyclopenta[1,2-*b*]indole-10-carboxamide (8b)

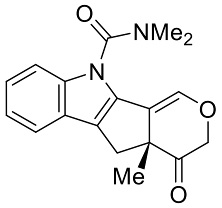

Prepared following General Procedure L from prochiral diketone **7b**. Obtained as colorless oil. **^{1}H NMR** (300 MHz, $CDCl_3$) δ 7.48 – 7.30 (m, 2H), 7.29 – 7.09 (m, 2H), 7.00 (s, 1H), 4.63 (d, *J* = 18.1 Hz, 1H), 4.16 (d, *J* = 18.4 Hz, 1H), 3.45 (d, *J* = 16.1 Hz, 1H), 3.08 (s, 3H), 3.05 (s, 3H), 2.68 (d, *J* = 16.1 Hz, 1H), 1.54 (s, 3H) ppm. **^{13}C NMR** (75 MHz, $CDCl_3$) δ 211.28 (CO), 154.38 (CO), 140.85 (C), 137.34 (CH), 136.18 (C), 125.65 (C), 123.79 (CH), 123.60 (C), 122.15 (CH), 121.40 (C), 119.56 (CH), 113.27 (CH), 72.07 (CH_2), 58.04 (C), 38.69 (CH_3 urea), 38.03 (CH_3 urea), 32.76 (CH_2), 24.34 (CH_3) ppm. **HRMS** [APCI]: *m/z* calculated for $C_{18}H_{19}N_2O_3$ $[M + H]^+$: 311.1390, found 311.1389.

Enantioselectivity was determined by chiral HPLC analysis on a Chiralpack IA3 column at rt (Hexane:*i*PrOH = 98:2, 1 mL/min).

A: Racemic sample prepared using $[Ir(COD)_2]BAr^F{}_4$ / *rac*-Binap (10 mol%) at 130 ˚C.

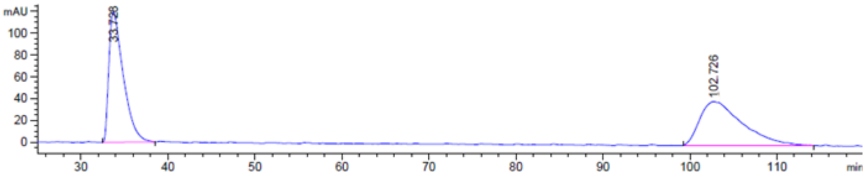

Signal 3: DAD1 C, Sig=210,4 Ref=off

Peak #	RetTime [min]	Type	Width [min]	Area [mAU*s]	Height [mAU]	Area %
1	33.728	BB	1.5506	1.37489e4	119.29317	49.5708
2	102.726	MM	5.7506	1.39870e4	40.53796	50.4292
Totals :				2.77358e4	159.83113	

B: Asymmetric sample prepared using $[Ir(COD)_2]BAr^F{}_4$ / *(R,R)-i*Pr-Duphos (10 mol%) at 130 ˚C (65% yield, 73:27 er).

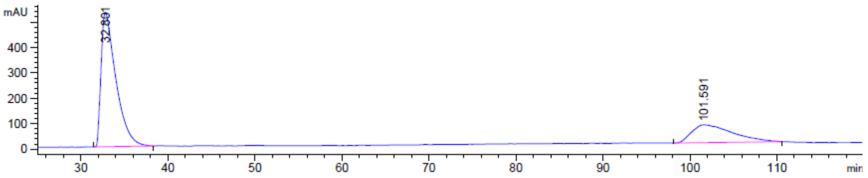

Signal 3: DAD1 C, Sig=210,4 Ref=off

Peak #	RetTime [min]	Type	Width [min]	Area [mAU*s]	Height [mAU]	Area %
1	32.801	BB	1.6080	6.24179e4	528.43774	73.0739
2	101.591	BB	3.8019	2.29996e4	70.68920	26.9261
Totals :				8.54176e4	599.12695	

(*R*)-10a-benzyl-*N,N*-dimethyl-1-oxo-2,3,10,10a-tetrahydroindeno[1,2-*b*]indole-5(1*H*)-carboxamide (8c)

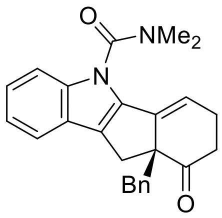

Prepared following General Procedure L from prochiral diketone **7c**. Obtained as yellow oil. **^{1}H NMR** (300 MHz, $CDCl_3$) δ 7.46 – 7.40 (m, 1H), 7.36 – 7.30 (m, 1H), 7.27 – 7.10 (m, 7H), 6.17 – 6.08 (m, 1H), 3.29 – 3.17 (m, 2H), 3.11 (m, 3H), 3.06 – 2.91 (m, 5H), 2.68 – 2.52 (m, 1H), 2.47 – 2.24 (m, 2H), 2.19 – 2.06 (m, 1H) ppm. **^{13}C NMR** (75 MHz, $CDCl_3$) δ 214.04 (CO), 154.01 (CO), 140.94 (C), 136.65 (C), 136.51 (C), 130.25 (CH), 128.10 (CH), 126.99 (CH), 125.58 (C), 125.26 (C), 124.03 (CH), 121.88 (CH), 119.99 (CH), 117.44 (CH), 113.00 (CH), 65.16 (C), 44.07 (CH_2), 38.36 (CH_3 urea), 37.93 (CH_3 urea),

36.92 (CH_2), 32.23 (CH_2), 22.86 (CH_2) ppm. **HRMS** [APCI]: *m/z* calculated for $C_{25}H_{25}N_2O_2$ [M + H]$^+$: 385.1911, found 385.1916. **MP** = 193.2 – 195.5 °C

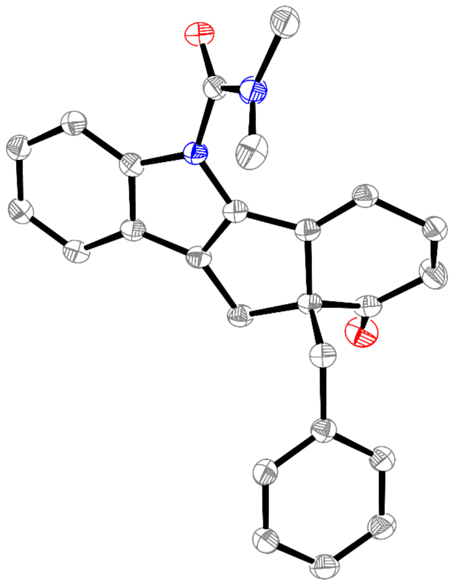

Figure S11. X-ray structure of **8c** (CCDC 2488406)

Enantioselectivity was determined by chiral HPLC analysis on a Chiralpack IA3 column at rt (Hexane:*i*PrOH = 95:5, 1 mL/min).

A: Racemic sample prepared using [Ir(COD)$_2$]BAr^F$_4$ / *rac*-Binap (10 mol%) at 130 ˚C.

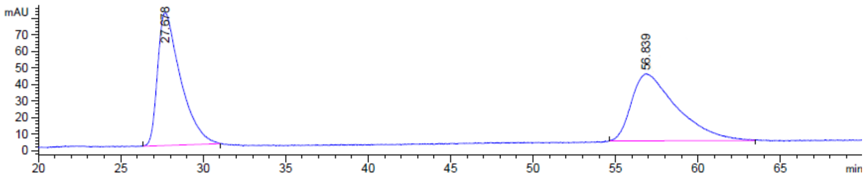

Signal 3: DAD1 C, Sig=210,4 Ref=off

Peak #	RetTime [min]	Type	Width [min]	Area [mAU*s]	Height [mAU]	Area %
1	27.678	MM	1.1451	7733.53857	80.17446	50.0985
2	56.839	MM	3.1727	7703.13184	40.46516	49.9015
Totals :				1.54367e4	120.63962	

B: Asymmetric sample prepared using [Ir(COD)$_2$]BAr^F$_4$ / *(S)*-C$_3$-Tunephos (10 mol%) at 130 ˚C (47% yield, 80:20 er).

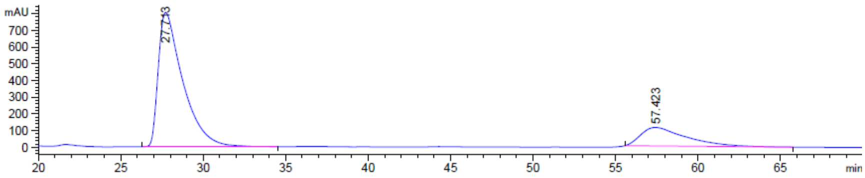

Signal 3: DAD1 C, Sig=210,4 Ref=off

Peak #	RetTime [min]	Type	Width [min]	Area [mAU*s]	Height [mAU]	Area %
1	27.713	BB	1.4580	8.31091e4	803.96545	79.7427
2	57.423	BB	2.2435	2.11125e4	110.87482	20.2573
Totals :				1.04222e5	914.84028	

(*R*)-*N,N*,3,3,10a-pentamethyl-1-oxo-2,3,10,10a-tetrahydroindeno[1,2-*b*]indole-5(1*H*)-carboxamide (8d)

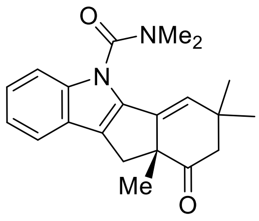

Prepared following General Procedure L from prochiral diketone **7d**. Obtained as colorless oil. **^{1}H NMR** (300 MHz, $CDCl_3$) δ 7.53 (d, *J* = 8.2 Hz, 1H), 7.49 – 7.43 (m, 1H), 7.30 – 7.22 (m, 1H), 7.21 – 7.14 (m, 1H), 5.64 (s, 1H), 3.24 (d, *J* = 15.5 Hz, 1H), 3.13 (s, 3H), 3.04 (s, 3H), 2.84 (d, *J* = 15.2 Hz, 1H), 2.65 (d, *J* = 15.4 Hz, 1H), 2.30 (d, *J* = 15.0 Hz, 1H), 1.62 (s, 3H), 1.32 (s, 3H), 1.10 (s, 3H) ppm. **^{13}C NMR** (75 MHz, $CDCl_3$) δ 212.27 (CO), 153.98 (CO), 141.08 (C), 138.82 (C), 136.54 (C), 126.25 (CH), 125.68 (C), 125.21 (C), 124.20 (CH), 122.07 (CH), 119.82 (CH), 113.56 (CH), 58.39 (C), 50.39

(CH$_2$), 38.69 (C), 38.58 (CH$_3$ urea), 37.53 (CH$_3$ urea), 33.08 (CH$_2$), 31.89 (CH$_3$), 30.91 (CH$_3$), 29.79 (CH$_3$) ppm. **HRMS** [APCI]: *m/z* calculated for $C_{21}H_{25}N_2O_2$ $[M + H]^+$: 337.1911, found 337.1912.

Enantioselectivity was determined by chiral HPLC analysis on a Chiralpack IA3 column at rt (Hexane:*i*PrOH = 95:5, 1 mL/min).

A: Racemic sample prepared using [Ir(COD)$_2$]BAr$^F{}_4$ / *rac*-Binap (10 mol%) at 130 ˚C.

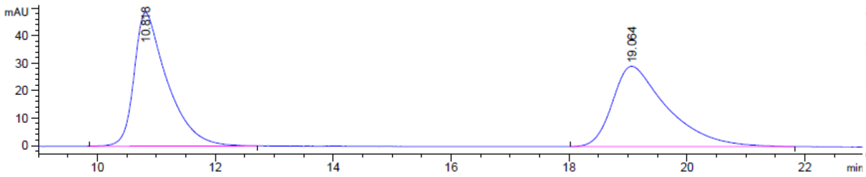

Signal 2: DAD1 B, Sig=254,4 Ref=off

Peak #	RetTime [min]	Type	Width [min]	Area [mAU*s]	Height [mAU]	Area %
1	10.818	BB	0.5608	1898.38708	48.69498	50.1385
2	19.064	BB	0.9131	1887.89783	29.24606	49.8615
Totals :				3786.28491	77.94104	

B: Asymmetric sample prepared using [Ir(COD)$_2$]BAr$^F{}_4$ / *(R,R)*-*i*Pr-Duphos (10 mol%) at 130 ˚C (51% yield, 84:16 er).

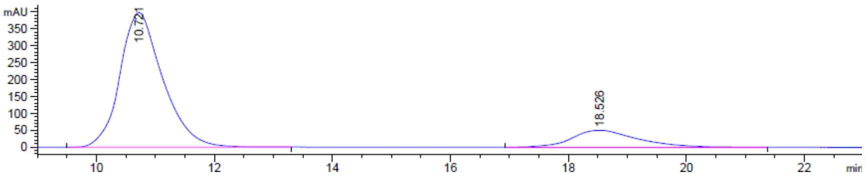

Signal 2: DAD1 B, Sig=254,4 Ref=off

Peak #	RetTime [min]	Type	Width [min]	Area [mAU*s]	Height [mAU]	Area %
1	10.721	BB	0.7517	2.00427e4	397.87732	83.8201
2	18.526	BB	1.1160	3868.86963	50.26426	16.1799
Totals :				2.39116e4	448.14158	

(*R*)-*N,N*,4a-trimethyl-5-oxo-4a,5,6,7-tetrahydroindeno[1,2-*b*]pyrrole-1(4*H*)-carboxamide (8e)

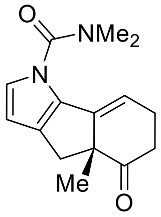

Prepared following General Procedure L from prochiral diketone **7e**. Obtained as pale-yellow oil. **^{1}H NMR** (300 MHz, C$_6$D$_6$) δ 6.75 (d, *J* = 3.0 Hz, 1H), 5.98 – 5.91 (m, 1H), 5.89 (d, *J* = 3.0 Hz, 1H), 3.41 (d, *J* = 16.0 Hz, 1H), 2.47 (d, *J* = 16.0 Hz, 1H), 2.40 (s, 6H), 2.35 – 2.12 (m, 2H), 2.08 – 1.86 (m, 2H), 1.21 (s, 3H) ppm. **^{13}C NMR** (75 MHz, C$_6$D$_6$) δ 212.36 (CO), 153.91 (CO), 138.89 (C), 134.32 (C), 132.32 (C), 126.22 (CH), 112.33 (CH), 107.15 (CH), 60.54 (C), 37.45 (CH$_3$ urea), 35.88 (CH$_2$), 35.09 (CH$_2$), 24.65 (CH$_3$), 22.92 (CH$_2$) ppm. **HRMS** [APCI]: *m/z* calculated for $C_{15}H_{19}N_2O_2$ $[M + H]^+$: 259.1441, found 259.1444.

Enantioselectivity was determined by chiral HPLC analysis on a Chiralpack IF3 column at rt (Hexane:*i*PrOH = 95:5, 1 mL/min).

A: Racemic sample prepared using [Ir(COD)$_2$]BAr$^F{}_4$ / *rac*-Binap (10 mol%) at 130 ˚C.

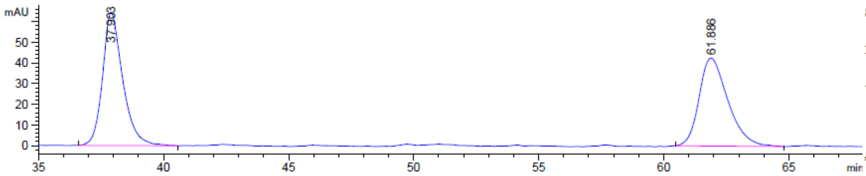

Signal 3: DAD1 C, Sig=210,4 Ref=off

Peak #	RetTime [min]	Type	Width [min]	Area [mAU*s]	Height [mAU]	Area %
1	37.903	BB	0.8183	3607.73047	64.32891	51.4964
2	61.886	BB	1.1158	3398.05933	42.45502	48.5036
Totals :				7005.78979	106.78393	

B: Asymmetric sample prepared using [Ir(COD)$_2$]BAr$^F{}_4$ / *(R,R)*-*i*Pr-Duphos (10 mol%) at 130 ˚C (56% yield, 73:27 er).

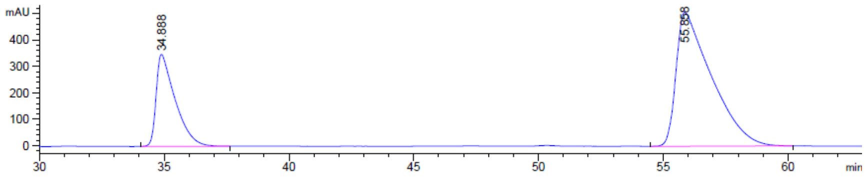

Signal 3: DAD1 C, Sig=210,4 Ref=off

Peak #	RetTime [min]	Type	Width [min]	Area [mAU*s]	Height [mAU]	Area %
1	34.888	BB	0.7551	1.84372e4	348.53949	26.9208
2	55.858	BB	1.3294	5.00496e4	506.27173	73.0792
Totals :				6.84868e4	854.81122	

2-((3*aR*,6*aR*,*Z*)-6*a*-hydroxy-3*a*-methyl-4-oxohexahydropentalen-1(2H)-ylidene)-*N*,*N*-dimethylacetamide (10a)

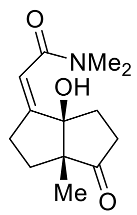

Prepared following General Procedure L from prochiral diketone **9a**. Obtained as white solid. **^{1}H NMR** (300 MHz, $CDCl_3$) δ 7.17 (br, 1H), 6.17 (t, *J* = 2.2 Hz, 1H), 3.12 (s, 3H), 3.02 (s, 3H), 2.83 – 2.67 (m, 1H), 2.64 – 2.44 (m, 2H), 2.43 – 2.24 (m, 2H), 2.17 – 2.06 (m, 1H), 1.89 – 1.77 (m, 1H), 1.67 – 1.55 (m, 1H), 1.12 (s, 3H) ppm. **^{13}C NMR** (75 MHz, $CDCl_3$) δ 220.9 (CO), 167.6 (CO), 167.3 (C), 112.9 (CH), 87.0 (COH), 60.9 (C), 38.5 (CH_3 amide), 36.2 (CH_3 amide + CH_2), 33.3 (CH_2), 33.0 (CH_2), 32.2 (CH_2), 14.5 (CH_3) ppm. **HRMS** [APCI]: *m/z* calculated for $C_{13}H_{20}NO_3$ $[M + H]^+$: 238.1438, found 238.1435.

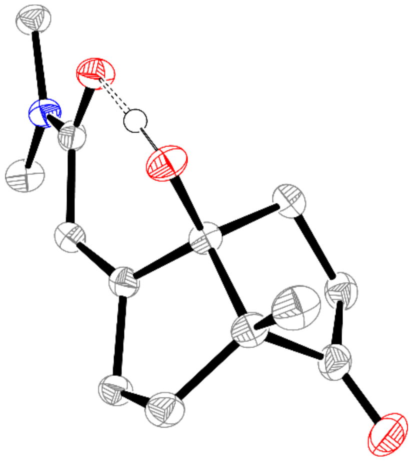

Figure S12. X-ray structure of **10a** (CCDC 2531879)

Enantioselectivity was determined by chiral HPLC analysis on a Chiralpack IF3 column at rt (Hexane:*i*PrOH = 85:15, 1 mL/min).

A: Racemic sample prepared using $[Ir(COD)_2]BAr^F{}_4$ / *rac*-Binap (10 mol%) at 130 ˚C.

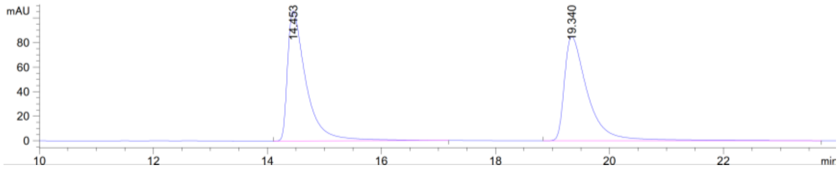

Signal 2: DAD1 B, Sig=254,4 Ref=off

Peak #	RetTime [min]	Type	Width [min]	Area [mAU*s]	Height [mAU]	Area %
1	14.453	BB	0.3346	2401.25195	105.89072	49.7886
2	19.340	BB	0.4210	2421.64673	84.83270	50.2114
Totals :				4822.89868	190.72342	

B: Asymmetric sample prepared using $[Ir(COD)_2]BAr^F{}_4$ / *(R)*-DTBM-Segphos (10 mol%) at 130 ˚C (Table S7, entry 1, 44% yield, 58:12 er).

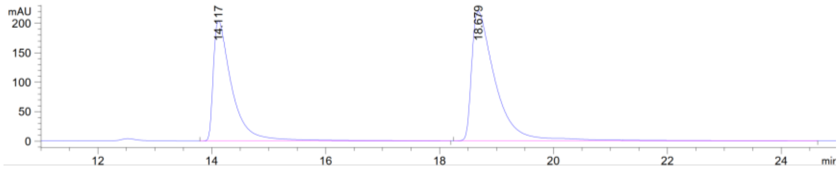

Signal 2: DAD1 B, Sig=254,4 Ref=off

Peak #	RetTime [min]	Type	Width [min]	Area [mAU*s]	Height [mAU]	Area %
1	14.117	BB	0.3318	4659.07568	204.48668	42.1051
2	18.679	BB	0.4267	6406.27100	219.39815	57.8949
Totals :				1.10653e4	423.88483	

C: Asymmetric sample prepared using $[Ir(COD)_2]BAr^F{}_4$ / *(R)*-Binap (10 mol%) at 130 ˚C (Table S7, entry 2, 85% yield, 77:23 er).

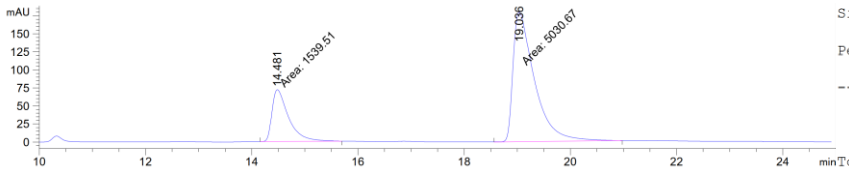

Signal 2: DAD1 B, Sig=254,4 Ref=off

Peak #	RetTime [min]	Type	Width [min]	Area [mAU*s]	Height [mAU]	Area %
1	14.481	MM	0.3579	1539.50610	71.69955	23.4317
2	19.036	MM	0.4674	5030.67188	179.39568	76.5683
Totals :				6570.17798	251.09523	

D: Asymmetric sample prepared using [Ir(COD)$_2$]BAr$^F{}_4$ / *(S)*-C3-Tunephos (10 mol%) at 130 ˚C (Table S7, entry 3, 83% yield, 72:28 er).

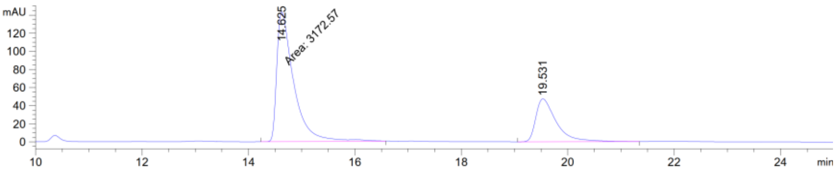

Signal 2: DAD1 B, Sig=254,4 Ref=off

Peak #	RetTime [min]	Type	Width [min]	Area [mAU*s]	Height [mAU]	Area %
1	14.625	MM	0.3694	3172.56567	143.14285	71.5226
2	19.531	BB	0.3970	1263.18848	47.39200	28.4774
Totals :				4435.75415	190.53485	

E: Asymmetric sample prepared using [Ir(COD)$_2$]BAr$^F{}_4$ / *(R,R)*-*i*Pr-Duphos (10 mol%) at 130 ˚C (Table S7, entry 5, 91% yield, 69:31 er).

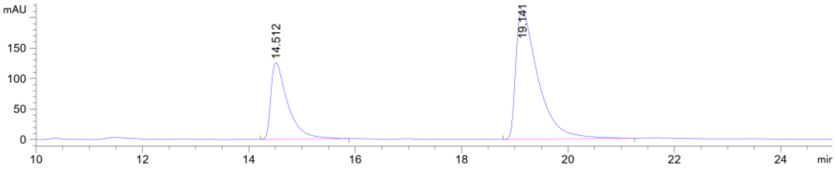

Signal 2: DAD1 B, Sig=254,4 Ref=off

Peak #	RetTime [min]	Type	Width [min]	Area [mAU*s]	Height [mAU]	Area %
1	14.512	BB	0.3251	2767.34741	125.60066	31.1487
2	19.141	BB	0.4198	6116.96338	212.53540	68.8513
Totals :				8884.31079	338.13606	

F: Asymmetric sample prepared using [Ir(COD)$_2$]BAr$^F{}_4$ / *(R,R)*-QuinoxP* (10 mol%) at 130 ˚C (Table S7, entry 6, 74% yield, 76:24 er).

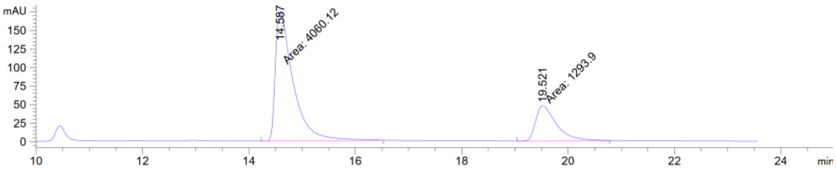

Signal 2: DAD1 B, Sig=254,4 Ref=off

Peak #	RetTime [min]	Type	Width [min]	Area [mAU*s]	Height [mAU]	Area %
1	14.587	MM	0.3865	4060.11816	175.06396	75.8331
2	19.521	MM	0.4545	1293.90063	47.45062	24.1669
Totals :				5354.01880	222.51458	

G: Asymmetric sample prepared using [Ir(COD)$_2$]BAr$^F{}_4$ / *(R,R)*-BenzP* (10 mol%) at 130 ˚C (Table S7, entry 7, 99% yield, 81:19 er).

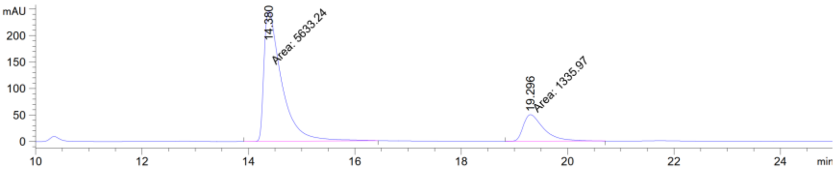

Signal 2: DAD1 B, Sig=254,4 Ref=off

Peak #	RetTime [min]	Type	Width [min]	Area [mAU*s]	Height [mAU]	Area %
1	14.380	MM	0.3826	5633.24463	245.42401	80.8304
2	19.296	MM	0.4445	1335.97302	50.09319	19.1696
Totals :				6969.21765	295.51720	

H: Asymmetric sample prepared using [Ir(COD)$_2$]BAr$^F{}_4$ / *(R,R)*-BenzP* (10 mol%) at 100 ˚C (Table S7, entry 8, 44% yield, 89:11 er).

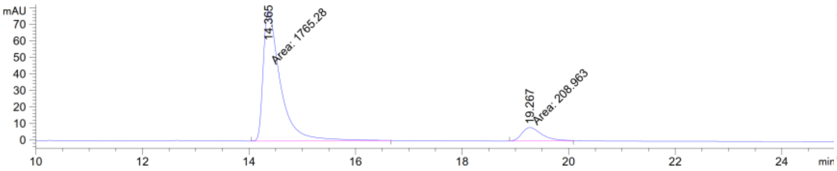

Signal 2: DAD1 B, Sig=254,4 Ref=off

Peak #	RetTime [min]	Type	Width [min]	Area [mAU*s]	Height [mAU]	Area %
1	14.365	MM	0.3738	1765.28455	78.70419	89.4155
2	19.267	MM	0.4295	208.96346	8.10797	10.5845
Totals :				1974.24800	86.81216	

I: Asymmetric sample prepared using [Ir(COD)$_2$]BAr$^F{}_4$ / *(R,R)*-BenzP* (10 mol%) at 100 ˚C for 3 days (Table S7, entry 9, 81% yield, 90:10 er).

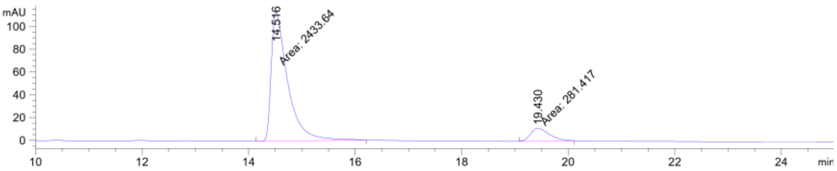

Signal 2: DAD1 B, Sig=254,4 Ref=off

Peak #	RetTime [min]	Type	Width [min]	Area [mAU*s]	Height [mAU]	Area %
1	14.516	MM	0.3595	2433.63843	112.81813	89.6349
2	19.430	MM	0.4096	281.41730	11.44993	10.3651
Totals :				2715.05573	124.26806	

5. Dehydration control experiments

Table S8. Control experiments with **2b** (obtained as single product in catalysis in the presence of Et_3SiH)

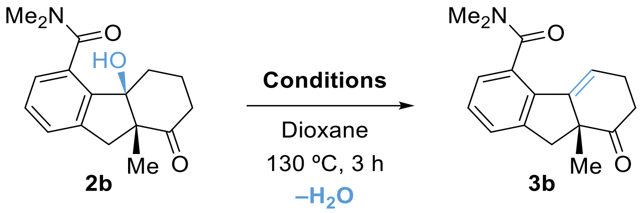

Conditions[a]	Conversion (%)[b]
No additive	0
$Ir(COD)_2BAr^F_4$ (10 mol%)	100
$Ir(COD)_2BAr^F_4$ (10 mol%) + *(R)*-DTBM-Segphos (10 mol%)	100
$Ir(COD)_2BAr^F_4$ (10 mol%) + *(R)*-DTBM-Segphos (10 mol%) + Et_3SiH	75
$[Ir(COD)Cl]_2$ (10 mol%)	21
$IrCl_3$	100 (85% **3b**)[c]
$ZnCl_2$	100
*p*TsOH (20 mol%)	100

[a] Reactions performed following General Procedure L, with component modifications as indicated in the table. [b] Conversion was determined by ^{1}H-NMR of the crude. [c] A 15% of an unknown product was observed.

Table S9. Control experiments with **2q** (product that do not dehydrate under standard conditions)

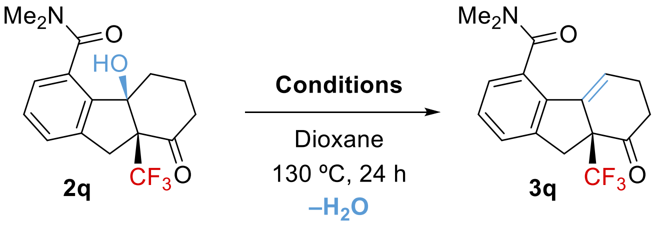

Conditions	Conversion (%)
$Ir(COD)_2BAr^F_4$ (10 mol%)	No reaction
p-TsOH (20 mol%)	81 % (66 % isolated yield)

Table S10. Control experiments with **2v** (product that do not dehydrate under standard conditions).

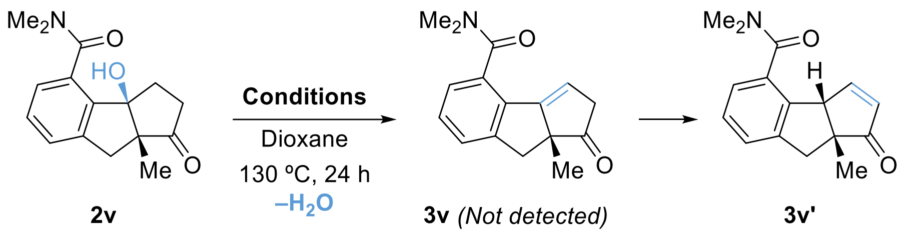

Conditions	Conversion (%)
Ir(COD)$_2$BAr^F$_4$ (10 mol%)	0
p-TsOH (20 mol%)	100 (80 % isolated yield)

(8a*R*)-*N,N*,8a-trimethyl-1-oxo-1,3a,8,8a-tetrahydrocyclopenta[*a*]indene-4-carboxamide (3v')

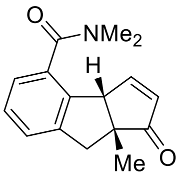

Prepared following the procedure: *p*-TSA (2.5 mg, 0.015 mmol, 20 mol%), alcohol **2v** (20 mg, 0.073 mmol) and dioxane (0.6 mL, 0.125 M) were sequentially added to a sealed tube and the reaction mixture was stirred at 130 ˚C, 24h. The reaction mixture was diluted with EtOAc, washed with sat. aq. $NaHCO_3$, dried over anhydrous Na_2SO_4, filtered, and concentrated in vacuo. The crude residue was purified by flash chromatography (SiO_2-4g, 13 mL/min, 0 → 10% → 20% CH_2Cl_2/Et_2O) to give 15 mg of **3v'** (80% yield) as colorless oil. **^{1}H NMR** (500 MHz, $CDCl_3$) δ 7.76 (dd, *J* = 5.7, 2.9 Hz, 1H), 7.23 (t, *J* = 7.4 Hz, 1H), 7.16 (d, *J* = 7.9 Hz, 1H), 7.10 (d, *J* = 7.4 Hz, 1H), 6.06 (dd, *J* = 5.4, 1.7 Hz, 1H), 4.23 (s, 1H), 3.24 (d, *J* = 17.5 Hz, 1H), 3.18 (s, 3H), 2.94 (s, 3H), 2.90 (d, *J* = 17.6 Hz, 1H), 1.38 (s, 3H) ppm. **^{13}C NMR** (126 MHz, $CDCl_3$) δ 213.79 (CO), 170.75 (CO), 164.25 (CH), 143.10 (C), 138.76 (C), 133.06 (C), 131.65 (CH), 127.99 (CH), 126.56 (CH), 124.91 (CH), 60.98 (CH), 53.30 (C), 41.73 (CH_2), 39.27 (CH_3 amide), 35.13 (CH_3 amide) 22.83 (CH_3) ppm. Note: The relative stereochemical configuration was determined by NOE experiments. **HRMS** [APCI]: *m/z* calculated for $C_{16}H_{18}NO_2$ $[M + H]^+$: 256.1332, found 256.1326.

(*S*)-*N,N*-dimethyl-1-oxo-9a-(trifluoromethyl)-2,3,9,9a-tetrahydro-1*H*-fluorene-5-carboxamide (3q)

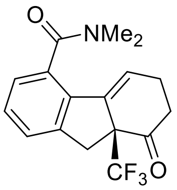

Prepared following the procedure: *p*-TSA (2.5 mg, 0.015 mmol, 20 mol%), alcohol **2q** (25 mg, 0.073 mmol) and dioxane (0.6 mL, 0.125 M) were sequentially added to a sealed tube and the reaction mixture was stirred at 130 ˚C, 24h. The reaction mixture was diluted with EtOAc, washed with sat. aq. $NaHCO_3$, dried over anhydrous Na_2SO_4, filtered, and concentrated in vacuo. The crude residue was purified by flash chromatography (SiO_2-4g, 13 mL/min, 0 → 10% → 20% CH_2Cl_2/Et_2O) to give 14 mg of **3q** (66% yield) as yellow oil. **^{1}H NMR** (500 MHz, $CDCl_3$) δ 7.31 – 7.27 (m, 2H), 7.13 (s, 1H), 6.54 (s, 1H), 3.45 (d, *J* = 17.7 Hz, 1H), 3.33 (d, *J* = 17.6 Hz, 1H), 3.16 (s, 3H), 2.96 – 2.85 (m, 1H), 2.84 – 2.76 (m, 1H), 2.80 (s, 3H), 2.71 – 2.60 (m, 1H), 2.52 – 2.42 (m, 1H) ppm. **^{13}C NMR** (126 MHz, $CDCl_3$) δ 204.78 (CO), 170.25 (CO), 141.51 (C), 137.02 (C), 133.91 (C), 131.43 (C), 129.31 (CH), 126.83 (CH), 126.19 (q, *J* = 284.7 Hz, CF_3), 125.47 (CH), 125.44 (CH), 62.35 (q, *J* = 25.2 Hz, C), 38.10 (CH_3 amide), 35.41(CH_2), 34.97 (CH_3 amide), 34.79 (CH_2), 24.28 (CH_2) ppm. **^{19}F NMR** (471 MHz, $CDCl_3$) δ -71.08 (s, 3F) ppm. **HRMS** [APCI]: *m/z* calculated for $C_{17}H_{17}F_3NO_2$ $[M + H]^+$: 324.1206, found 324.1204.

6. Transformations of the products obtained

Diasteroselective hydrogenation of 5a (synthesis of 5a')

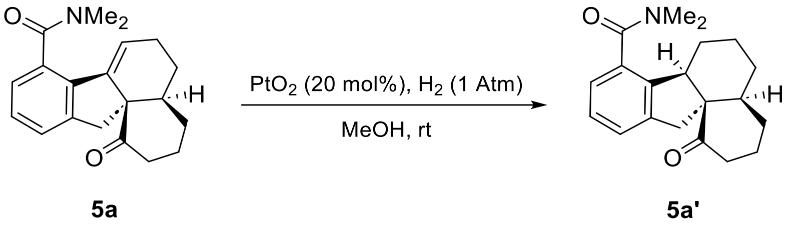

PtO_2 (as hydrate, 2.4 mg, 20 mol%) was added to a solution of **5a** (15 mg, 0.05 mmol) in MeOH (0.49 mL) under argon. The resulting black suspension was purged with hydrogen for 1 minute and then stirred at rt under hydrogen atmosphere (balloon) until complete consumption of starting material as indicated by TLC (2 h). The catalyst was removed by filtration through a Celite pad, and the solids were washed with MeOH. The filtrate was concentrated under reduced pressure and the crude residue was purified by flash chromatography (SiO_2-4g, 13 mL/min, 0 $\rightarrow$ 70% hexane/EtOAc), to afford 11.6 mg of **(4a*S*,7a*R*,12a*R*)-*N,N*-dimethyl-1-oxo-1,2,3,4,4a,5,6,7,7a,12-decahydrobenzo[*j*]fluorene-8-carboxamide 5a'** as a white solid (77% yield). Note: The relative and absolute configuration was determined by single crystal X-ray diffraction. **^{1}H NMR** (500 MHz, $CDCl_3$) δ 7.11 – 7.03 (m, 1H), 6.92 (d, *J* = 7.1 Hz, 1H), 3.25 (d, *J* = 15.1 Hz, 1H), 3.11 (s, 3H), 3.01 (s, 3H), 2.90 – 2.84 (m, 1H), 2.81 – 2.68 (m, 2H), 2.27 – 2.21 (m, 1H), 2.14 – 2.02 (m, 3H), 1.93 – 1.84 (m, 2H), 1.81 – 1.73 (m, 1H), 1.67 – 1.61 (m, 2H), 1.50 – 1.41 (m, 2H), 1.34 (td, *J* = 12.7, 3.0 Hz, 1H) ppm. **^{13}C NMR** (126 MHz, $CDCl_3$) δ 213.3 (CO), 171.6 (CO), 142.6 (C), 139.8 (C), 131.4 (C), 125.9 (CH), 124.6 (CH), 123.8 (CH), 65.6 (C), 53.2 (CH), 45.0 (CH), 42.9 (CH_2), 39.6 (CH_3 amide), 38.1 (CH_2), 34.7 (CH_3 amide), 28.0 (CH_2), 27.8 (CH_2), 26.7 (CH_2), 22.0 (CH_2), 21.6 (CH_2) ppm. **HRMS** [APCI]: *m/z* calculated for $C_{20}H_{26}NO_2$ $[M + H]^+$: 312.1958, found 312.1947. **MP** = 160.4 – 161.5 °C

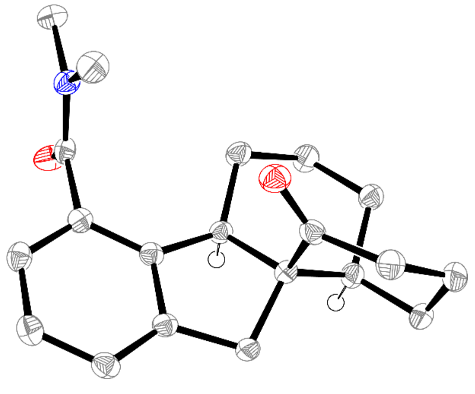

Figure S13. X-ray structure of **5a'** (CCDC 2504170)

Diastereoselective hydrogenation of 10a (synthesis of **10a'**)

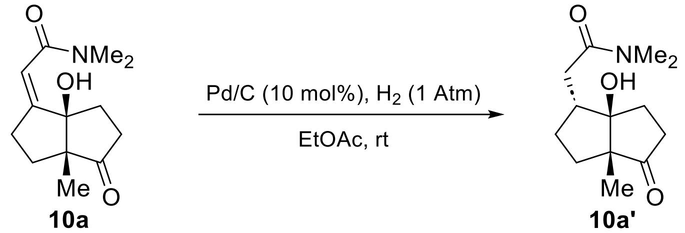

10a **10a'**

Pd/C (5% Pd content, 9 mg, 20 mol%) was added to a solution of **10a** (10 mg, 0.04 mmol) in EtOAc (1 mL) under argon. The resulting black suspension was purged with hydrogen for 1 minute and then stirred at rt under hydrogen atmosphere (balloon) until complete consumption of starting material as indicated by TLC (2 h). The catalyst was removed by filtration through a Celite pad, and the solids were washed with EtOAc. The filtrate was concentrated under reduced pressure and the crude residue was purified by flash chromatography (SiO_2-4g, 13 mL/min, 0 $\rightarrow$ 100% hexane/EtOAc), to afford 8 mg of **2-((1*R*,3a*R*,6a*R*)-6a-hydroxy-3a-methyl-4-oxooctahydropentalen-1-yl)-*N,N*-dimethylacetamide 10a'** as a colourless oil (79% yield). Note: The relative and absolute configuration was tentatively assigned by NOE spectroscopy. **^{1}H NMR** (500 MHz, $CDCl_3$) δ 5.81 (s, 1H), 3.06 (s, 3H), 2.98 (s, 3H), 2.70 – 2.53 (m, 3H), 2.42 (dd, *J* = 16.8, 11.0 Hz, 1H), 2.30 (ddd, *J* = 17.9, 8.2, 2.6 Hz, 1H), 1.94 – 1.84 (m, 2H), 1.82 – 1.74 (m, 2H), 1.59 – 1.52 (m, 1H), 1.28 – 1.20 (m, 1H), 1.12 (s, 3H) ppm. **^{13}C NMR** (126 MHz, $CDCl_3$) δ 222.9 (CO), 173.6 (CO), 87.1 (COH), 59.3 (C), 44.1 (CH), 37.9 (CH_3), 36.2 (CH_2), 35.9 (CH_3), 34.1 (CH_2), 34.0 (CH_2), 30.0 (CH_2), 29.3 (CH_2), 17.9 (CH_3) ppm. **HRMS** [APCI]: *m/z* calculated for $C_{13}H_{22}NO_3$ $[M + H]^+$: 240.1594, found 240.1596.

Directing group cleavage of compound 8a (synthesis of **8a'**)[12]

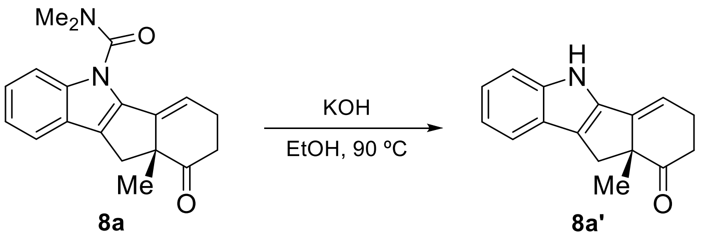

8a **8a'**

A mixture of **8a** (55 mg, 0.178 mmol, 1.0 equiv) and KOH (30 mg, 0.53 mmol, 3.0 equiv) in EtOH (1.8 mL) was stirred at 90 °C until complete consumption of starting material as indicated by TLC. The reaction was then quenched with sat. aq. NH_4Cl solution. The aqueous phase was extracted with CH_2Cl_2. The combined organic phases were dried over anhydrous Na_2SO_4, filtered and concentrated under reduced pressure. The crude residue was purified by flash chromatography (SiO_2-4g, 13 mL/min, 0 $\rightarrow$ 12% $\rightarrow$ 20% hexane/EtOAc), to afford 26 mg of **(*R*)-10a-methyl-3,5,10,10a-tetrahydroindeno[1,2-b]indol-1(2*H*)-one 8a'** as an orange solid (61% yield). **^{1}H NMR** (500 MHz, CD_2Cl_2) δ 8.11 (s, 1H), 7.48 (dd, *J* = 7.9, 1.1 Hz, 1H), 7.34 (d, *J* = 8.1 Hz, 1H), 7.16 (ddd, *J* = 8.3, 7.1, 1.2 Hz, 1H), 7.08 (ddd, *J* = 8.1, 7.1, 1.1 Hz, 1H), 5.97 (dd, *J* = 7.1, 2.9 Hz, 1H), 3.33 (dd, *J* = 15.7, 1.4 Hz, 1H), 2.86 – 2.75 (m, 1H), 2.69 (d, *J* = 15.6 Hz, 1H), 2.68 (m, 1H), 2.63 – 2.51 (m, 1H), 2.39 – 2.28 (m, 1H), 1.43 (s, 3H) ppm. **^{13}C NMR** (126 MHz, CD_2Cl_2) δ 214.35 (CO), 141.91 (C), 139.91 (C), 139.31 (C), 125.28 (C), 122.97 (CH), 122.95 (C), 120.42 (CH), 119.77 (CH), 113.30 (CH), 112.08 (CH), 60.50 (C), 36.39 (CH_2), 34.35 (CH_2), 24.97 (CH_3), 23.36 (CH_2) ppm. **HRMS** [APCI]: *m/z* calculated for $C_{18}H_{18}NO$ $[M + H]^+$: 264.1383, found 264.1377. **MP** = 171.2 – 172.2 °C

Diasteroselective Pd/C-catalyzed hydrogenation (synthesis of **11b**)

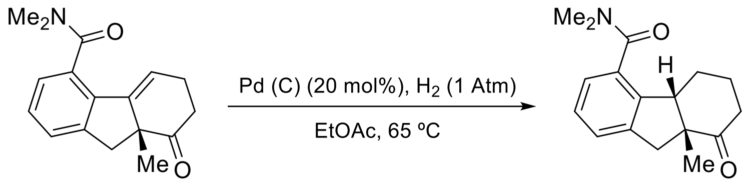

3b **11b**

Pd/C (10% w/w, 38 mg, 20 mol%) was added to a solution of **3b** (48 mg, 0.179 mmol, 1.0 equiv) in EtOAc (1.8 mL) under argon. The resulting black suspension was purged with hydrogen for 1 minute and then stirred at 65 °C under hydrogen atmosphere (balloon) until complete consumption of starting material as indicated by TLC. The catalyst was removed by filtration through a Celite pad, and the solids were washed with EtOAc. The filtrate was concentrated under reduced pressure and the crude residue was purified by flash chromatography (SiO_2-4g, 13 mL/min, 0 $\rightarrow$ 70% $\rightarrow$ 100% hexane/EtOAc), to afford 26 mg of **(4a*S*,9a*R*)-*N,N*,9a-trimethyl-1-oxo-2,3,4,4a,9,9a-hexahydro-1*H*-fluorene-5-carboxamide 11b** as a colorless oil (53% yield). Note: The relative stereochemical configuration was determined by NOE experiments. **^{1}H NMR** (500 MHz, $CDCl_3$): δ 7.24 (d, *J* = 7.5 Hz, 1H), 7.20 (t, *J* = 7.5 Hz, 1H), 7.07 (d, *J* = 7.4 Hz, 1H), 3.40 (d, *J* = 15.9 Hz, 1H), 3.23 (dd, *J* = 9.5, 5.5 Hz, 1H), 3.13 (s, 3H), 2.88 (s, 3H), 2.63 (d, *J* = 15.9 Hz, 1H), 2.55 – 2.45 (m, 1H), 2.40 (ddd, *J* = 15.8, 9.5, 5.9 Hz, 1H), 2.14 – 2.06 (m, 1H), 1.90 – 1.73 (m, 2H), 1.63 – 1.51 (m, 1H), 1.23 (s, 3H) ppm. **^{13}C NMR** (126 MHz, $CDCl_3$): δ 215.01 (CO), 170.93 (CO), 142.96 (C), 141.94 (C), 133.09 (C), 127.24 (CH), 126.03 (CH), 124.71 (CH), 55.27 (C), 53.98 (CH), 42.33 (CH_2), 38.97 (CH_3 amide), 38.46 (CH_2), 34.90 (CH_3 amide), 28.68 (CH_2), 23.55 (CH_3), 23.24 (CH_2) ppm. **HRMS** (APCI): *m/z* calculated for $C_{17}H_{22}NO_2$ $[M + H]^+$: 272.1645, found 272.1641.

Enantioselectivity was determined by chiral HPLC analysis on a Chiralpack IF3 column at rt (Hexane:*i*PrOH = 90:10, 1 mL/min).

A: Racemic sample

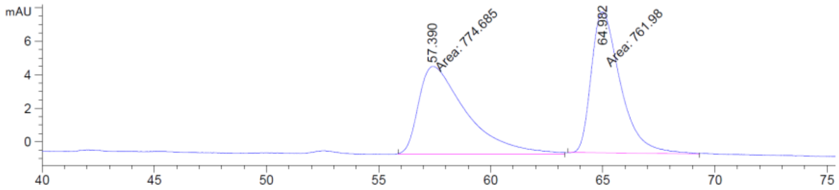

Signal 4: DAD1 D, Sig=300,4 Ref=off

Peak #	RetTime [min]	Type	Width [min]	Area [mAU*s]	Height [mAU]	Area %
1	57.390	MM	2.4694	774.68530	5.22863	50.4134
2	64.982	MM	1.5102	761.97955	8.40902	49.5866
Totals :				1536.66486	13.63765	

B: Asymmetric sample (90:10 er).

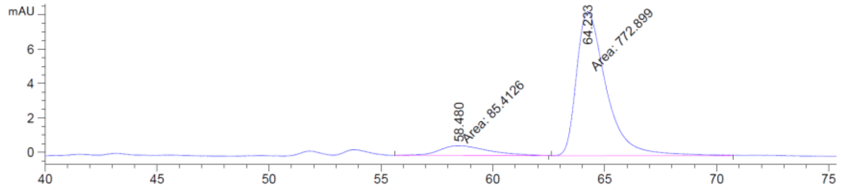

Signal 4: DAD1 D, Sig=300,4 Ref=off

Peak #	RetTime [min]	Type	Width [min]	Area [mAU*s]	Height [mAU]	Area %
1	58.480	MM	2.5008	85.41259	5.69231e-1	9.9512
2	64.233	MM	1.5340	772.89948	8.39765	90.0488
Totals :				858.31207	8.96688	

Olefin epoxidation (synthesis of **12b'**)

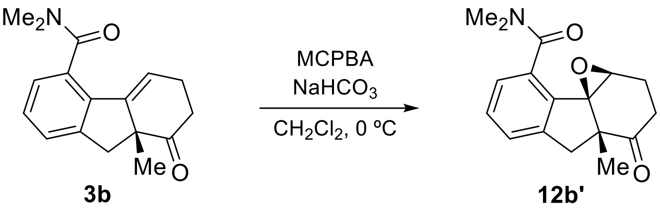

3b **12b'**

To a stirred solution of **3b** (44 mg, 0.164 mmol, 1.0 equiv) and $NaHCO_3$ (27.6 mg, 0.33 mmol, 2.0 equiv) in CH_2Cl_2 (2.0 mL), then added 3-chloroperoxybenzoic acid (42.6 mg, 0.247 mmol, 1.5 equiv) at 0 °C. The mixture was stirred at 0 °C until complete consumption of starting material as indicated by TLC and was quenched by adding water. The aqueous layer was extracted with DCM, the combined organic extracts and then dried over Na_2SO_4, filtered and concentrated under reduced pressure. The crude residue was purified by flash chromatography (SiO_2-4g, 13 mL/min, 0 → 70% → 80% hexane/EtOAc), to afford 19 mg of **(1a*S*,4a*R*,9b*R*)-*N,N*,4a-trimethyl-4-oxo-1a,2,3,4,4a,5-hexahydrofluoreno[4,4*a*-*b*]oxirene-9-carboxamide 12b'** as a colorless oil (42% yield). Note: Analysis of the crude reaction mixture by ^{1}H-NMR revealed the formation of a diastereomeric mixture (dr = 1:0.9). However, only one diastereomer could be isolated after chromatographic purification, as the other underwent complete decomposition. The relative stereochemical configuration of the isolated diastereomer was determined indirectly through NOE experiments on a derivative **10b** obtained from epoxide ring opening. **^{1}H NMR (300 MHz, $CDCl_3$):** *δ* 7.34 – 7.25 (m, 2H), 7.11 – 6.99 (m, 1H), 3.92 (s, 1H), 3.28 (d, *J* = 16.5 Hz, 1H), 3.09 (s, 3H), 2.91 (d, *J* = 16.4 Hz, 1H), 2.80 (s, 3H), 2.75 – 2.60 (m, 1H), 2.59 – 2.44 (m, 1H), 2.18 – 2.06 (m, 2H), 1.30 (s, 3H) ppm. **^{13}C NMR (75 MHz, $CDCl_3$):** *δ* 212.39 (CO), 169.96 (CO), 141.92 (C), 133.83 (C), 133.19 (C), 129.23 (CH), 126.03 (CH), 124.77 (CH), 71.41 (C), 61.47 (CH), 52.94 (C), 39.29 (CH_3 amide), 38.84 (CH_2), 34.67 (CH_3 amide), 31.73 (CH_2), 24.97 (CH_2), 21.73 (CH_3) ppm. **HRMS** (APCI): *m/z* calculated for $C_{17}H_{20}NO_3$ $[M + H]^+$: 286.1438, found 286.1436.

Epoxide ring opening (synthesis of **12b**)

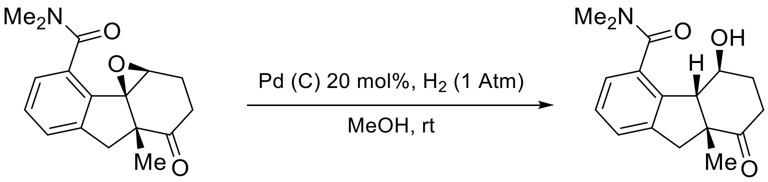

12b' **12b**

Pd/C (10% w/w, 18 mg, 20 mol%) was added to a solution of **12b'** (24 mg, 0.084 mmol, 1.0 equiv) in MeOH (0.85 mL) under argon. The resulting black suspension was purged with hydrogen for 1 minute and then stirred at rt under hydrogen atmosphere (balloon) until complete consumption of starting material as indicated by TLC. The catalyst was removed by filtration through a Celite pad and the solids were washed with MeOH. The filtrate was concentrated under reduced pressure and the crude residue was purified by preparative TLC (EtOAc), to afford 9 mg of **(4*S*,4a*S*,9a*R*)-4-hydroxy-*N,N*,9a-trimethyl-1-oxo-2,3,4,4a,9,9a-hexahydro-1*H*-fluorene-5-carboxamide 12b** as a colorless oil (39% yield). Note: The relative stereochemical configuration was determined by NOE experiments. **^{1}H NMR (500 MHz, $CDCl_3$):** *δ* 7.33 (d, *J* = 7.9 Hz, 1H), 7.27 (t, *J* = 7.5 Hz, 1H), 7.12 (d, *J* = 7.6 Hz, 1H), 3.79 – 3.72 (m, 1H), 3.32 (d, *J* = 16.0 Hz, 1H), 3.14 (s, 3H), 2.97 (d, *J* = 8.7 Hz, 1H), 2.87 (s, 3H), 2.67 – 2.50 (m, 3H), 2.23 – 2.14 (m, 1H), 1.91 – 1.79 (m, 2H), 1.13

(s, 3H) ppm. **^{13}C NMR (126 MHz, $CDCl_3$):** δ 213.08 (CO), 172.28 (CO), 142.74 (C), 142.34 (C), 133.06 (C), 127.55 (CH), 126.98 (CH), 125.00 (CH), 70.99 (CH), 62.09 (CH), 54.99 (C), 43.37 (CH_2 amide), 39.44 (CH_3), 35.92(CH_2), 35.54 (CH_3 amide), 31.95 (CH_2), 23.05 (CH_3) ppm. **HRMS** (APCI): *m/z* calculated for $C_{17}H_{20}NO_3$ [M + H]$^+$: 288.1594, found 288.1585.

Diasteroselective olefin dihydroxylation (synthesis of **13b**)[13]

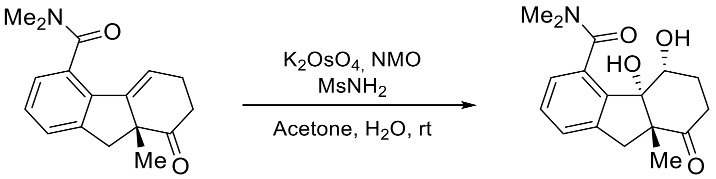

A solution of **3b** (36 mg, 0.134 mmol, 1.0 equiv) and NMO (*N*-methylmorpholine *N*-oxide, 31 mg, 0.267 mmol, 2.0 equiv) in acetone (1.7 mL) was added to a solution of K_2OsO_4 (14.8 mg, 0.04 mmol, 0.3 equiv) and methanesulfonamide (19 mg, 0.2 mmol, 1.5 equiv) in acetone (1 mL) and water (0.3 mL) at rt. The mixture was stirred until complete consumption of starting material as indicated by TLC, then sat. aq. Na_2SO_3 was added, and the resulting mixture was stirred for additional 30 min. The volatile materials were concentrated in vacuo and the aqueous layer was extracted with EtOAc. The combined organic layers were washed with brine, dried over anhydrous Na_2SO_4, filtered and concentrated in vacuo. The crude residue was purified by flash chromatography (SiO_2-4g, 13 mL/min, 0 $\rightarrow$ 100% hexane/EtOAc), to afford 20 mg of **(4*R*,4a*S*,9a*R*)-4,4a-dihydroxy-*N,N*,9a-trimethyl-1-oxo-2,3,4,4a,9,9a-hexahydro-1*H*-fluorene-5-carboxamide 13b** as a colorless solid (50% yield). **^{1}H NMR** (500 MHz, $CDCl_3$) δ 7.35 (d, *J* = 7.5 Hz, 1H), 7.29 – 7.23 (m, 1H), 7.00 (d, *J* = 7.6 Hz, 1H), 4.80 – 4.34 (br m, 1H), 3.91 (br s, 1H), 3.59 (dd, *J* = 15.2, 1.1 Hz, 1H), 3.09 (s, 3H), 2.97 – 2.80 (br m, 3H), 2.56 (br s, 1H), 2.50 (d, *J* = 15.3 Hz, 1H), 2.35 (br s, 1H), 2.05 (br s, 1H), 1.13 (s, 3H) ppm. **^{13}C NMR** (126 MHz, $CDCl_3$) δ 212.31 (CO), 173.63 (CO), 145.49 (C), 141.38 (C), 132.49 (C), 128.37 (CH), 127.47(CH), 124.77 (CH), 87.82 (C), 67.52 (CH), 60.94 (C), 39.65 (CH_3 amide), 38.49 (CH_2), 35.25 (CH_3 amide), 35.15 (CH_2), 29.95 (CH_2), 21.51 (CH_3) ppm. Note: most carbon signals are duplicated due to rotamerism. The relative stereochemical configuration was determined by single crystal X-ray diffraction. **HRMS** [APCI]: *m/z* calculated for $C_{17}H_{20}NO_3$ [M - H_2O]$^+$: 286.1438, found 286.1430. **MP** = 135.6 – 137.8 °C

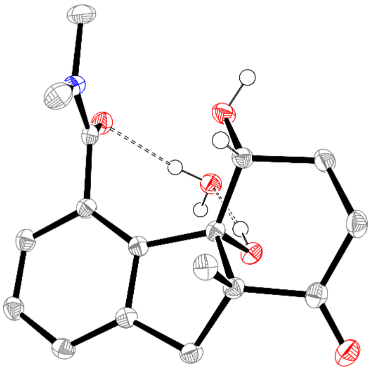

Figure S14. X-ray structure of **13b** (CCDC 2488407)

Diastereoselective ketone reduction (synthesis of **14b**)

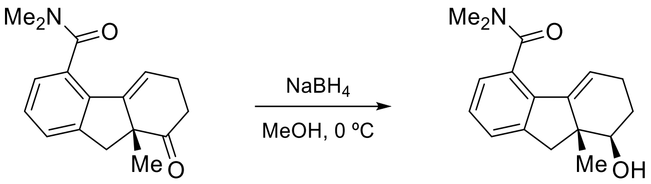

3b **14b**

$NaBH_4$ (8.2 mg, 0.218 mmol, 1.5 equiv) was added portionwise to a cooled solution of **3b** (39 mg, 0.146 mmol, 1.0 equiv) in MeOH (0.6 mL) at 0 °C. The resulting mixture was stirred at 0 °C until complete consumption of starting material as indicated by TLC. The reaction was quenched with water and extracted with EtOAc. The combined organic layer was washed with brine, dried over anhydrous Na_2SO4, filtered and concentrated under reduced pressure. The crude residue was purified by flash chromatography (SiO_2-4g, 13 mL/min, 0 $\rightarrow$ 70% hexane/EtOAc), to afford 26 mg of **(1*R*,9a*R*)-1-hydroxy-*N,N*,9a-trimethyl-2,3,9,9a-tetrahydro-1*H*-fluorene-5-carboxamide 14b** as a white solid (65% yield). <u>Note</u>: The relative stereochemical configuration was determined by single crystal X-ray diffraction. **^{1}H NMR** (300 MHz, $CDCl_3$): δ 7.25 – 7.15 (m, 2H), 7.08 – 7.01 (m, 1H), 5.79 (br s, 1H), 3.82 (s, 1H), 3.10 (s, 3H), 2.92 – 2.68 (m, 4H), 2.33 (br s, 2H), 1.90 – 1.67 (m, 3H), 1.08 (s, 3H) ppm. **^{13}C NMR** (75 MHz, $CDCl_3$): δ 171.09 (CO), 145.67 (C), 143.63 (C), 135.96 (C), 131.49 (C), 127.81 (CH), 126.20 (CH), 124.80 (CH), 119.69 (CH), 75.62 (CH), 47.81 (C), 45.07 (CH_2), 38.18 (CH_3 amide), 34.87 (CH_3 amide), 26.99 (CH_2), 25.80 (CH_2), 19.94 (CH_3) ppm. **HRMS** (APCI): *m/z* calculated for $C_{17}H_{22}NO_2$ $[M + H]^+$: 272.1645, found 272.1640. **MP** = 152.3 – 154.1 °C

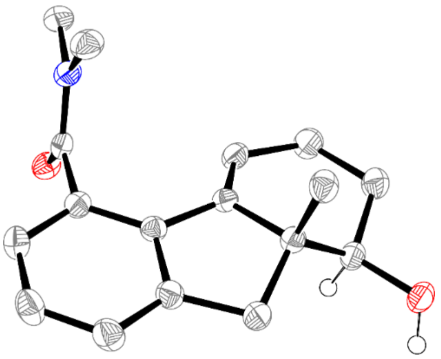

X-ray structure of **14b** (CCDC 2488402)

Enantioselectivity was determined by chiral HPLC analysis on a Chiralpack IF3 column at rt (Hexane:*i*PrOH = 85:15, 1 mL/min).

A: <u>Racemic sample</u>

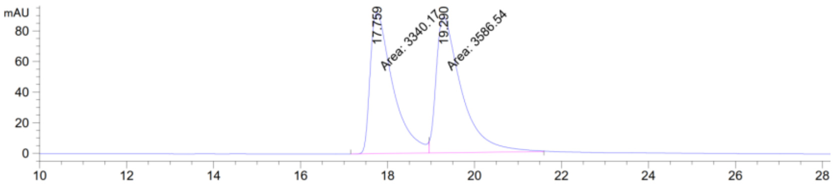

Signal 2: DAD1 B, Sig=254,4 Ref=off

Peak #	RetTime [min]	Type	Width [min]	Area [mAU*s]	Height [mAU]	Area %
1	17.759	MF	0.6047	3340.17090	92.06356	48.2216
2	19.290	FM	0.6748	3586.53564	88.58444	51.7784
Totals :				6926.70654	180.64800	

B: Asymmetric sample (90:10 er).

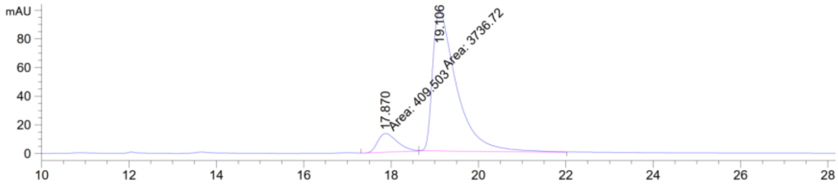

Signal 2: DAD1 B, Sig=254,4 Ref=off

Peak #	RetTime [min]	Type	Width [min]	Area [mAU*s]	Height [mAU]	Area %
1	17.870	MM	0.5209	409.50259	13.10126	9.8765
2	19.106	MM	0.6376	3736.71924	97.67668	90.1235
Totals :				4146.22183	110.77794	

Wittig reaction (synthesis of **15b**)

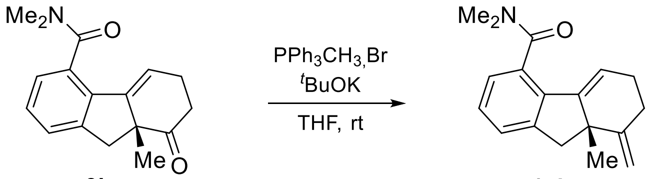

3b **15b**

PPh_3CH_3Br (132 mg, 0.371 mmol, 3.0 equiv) and ^tBuOK (42 mg, 0.371 mmol, 3.0 equiv) were added to a flame dried round bottom flask under argon atmosphere. Then THF (0.5 ml) was added and the reaction mixture was stirred for 1 h. Finally, a solution of **3b** (33 mg, 0.124 mmol, 1.0 equiv) in THF (0.25 mL) was added to the reaction mixture at rt. After completion of the reaction as indicated by TLC, the reaction mixture was quenched by adding saturated aq. NH_4Cl solution. The aqueous layer was extracted with EtOAc, the combined organic layers were dried over anhydrous Na_2SO_4, filtered and evaporated under reduced pressure. The crude residue was purified by flash chromatography (SiO_2-4g, 13 mL/min, 0 → 50% → 70% hexane/EtOAc), to afford 19 mg of **(*S*)-*N,N*,9a-trimethyl-1-methylene-2,3,9,9a-tetrahydro-1*H*-fluorene-5-carboxamide 15b** as a colorless oil (57% yield). **^{1}H NMR** (300 MHz, $CDCl_3$): δ 7.29 – 7.26 (m, 1H), 7.23 – 7.16 (m, 1H), 7.10 – 7.05 (m, 1H), 5.93 (s, 1H), 4.84 (s, 1H), 4.75 (s, 1H), 3.17 – 3.06 (m, 4H), 2.83 – 2.76 (m, 4H), 2.64 – 2.54 (m, 1H), 2.40 – 2.24 (m, 3H), 1.24 (s, 3H) ppm. **^{13}C NMR** (75 MHz, $CDCl_3$): δ 171.09 (CO), 152.44 (C), 145.60 (C), 143.22 (C), 135.99 (C), 131.51 (C), 127.76 (CH), 126.03 (CH), 124.75 (CH), 120.85 (CH), 106.43 (CH_2), 48.70 (C), 43.40, (CH_2) 38.21 (CH_3 amide), 34.92 (CH_3 amide), 29.41 (CH_3), 28.93 (CH_2), 27.33 (CH_2) ppm. **HRMS** (APCI-FIA-TOF): *m/z* calculated for $C_{18}H_{22}NO$ $[M + H]^+$: 268.1696, found 268.1701.

Enantioselectivity was determined by chiral HPLC analysis on a Chiralpack IG3 column at rt (Hexane:*i*PrOH = 95:5, 1 mL/min).

A: Racemic sample

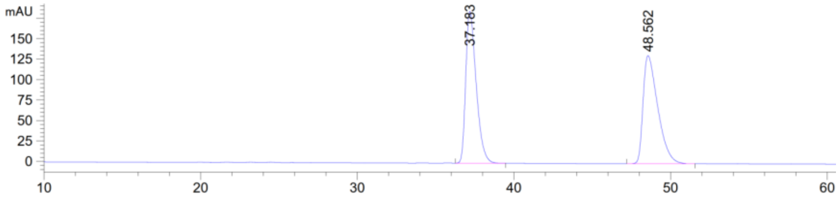

Signal 2: DAD1 B, Sig=254,4 Ref=off

Peak #	RetTime [min]	Type	Width [min]	Area [mAU*s]	Height [mAU]	Area %
1	37.183	BB	0.7094	8570.03906	185.35208	49.9980
2	48.562	BB	0.9657	8570.71191	131.96956	50.0020
Totals :				1.71408e4	317.32164	

B: Asymmetric sample (90:10 er).

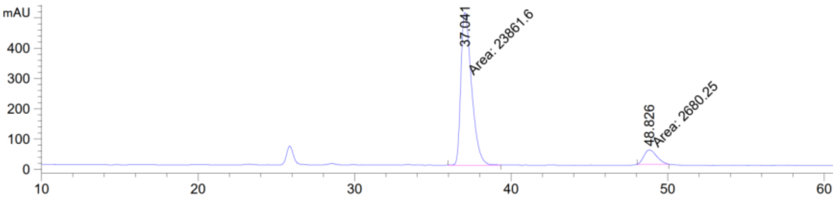

Signal 2: DAD1 B, Sig=254,4 Ref=off

Peak #	RetTime [min]	Type	Width [min]	Area [mAU*s]	Height [mAU]	Area %
1	37.041	MM	0.7883	1.23544e4	261.20688	89.7430
2	48.823	MM	0.9563	1412.01367	24.60960	10.2570
Totals :				1.37664e4	285.81648	

Ring iodination-oxidation (synthesis of **16b**)[14]

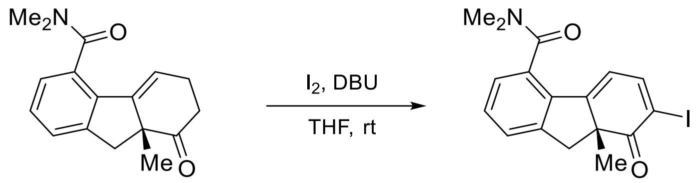

3b **16b**

A solution of **3b** (20 mg, 0.074 mmol, 1.0 equiv) in THF (0.75 mL) at 0 °C was treated with DBU (226 mg, 1.485 mmol, 20 equiv). Three portions of I_2 (94 mg, 0.37 mmol, 5.0 equiv) were added over 30 min and the reaction mixture was allowed to warm to rt until complete consumption of starting material as indicated by TLC. The mixture was poured into ether and water. The organic layer was washed with water, sat. aq. $Na_2S_2O_3$ solution and sat. aq. NaCl solution, dried over anhydrous Na_2SO_4, filtered and concentrated under reduced pressure. The crude residue was purified by flash chromatography (SiO_2-4g, 13 mL/min, 0 → 50% → 60% hexane/EtOAc), to afford 12 mg of **(*R*)-2-iodo-*N,N,*9a-trimethyl-1-oxo-9,9a-dihydro-1*H*-fluorene-5-carboxamide 16b** as a yellow oil (41% yield). **^{1}H NMR** (500 MHz, $CDCl_3$) δ 7.84 (d, *J* = 6.6 Hz, 1H), 7.45 (d, *J* = 7.7 Hz, 1H), 7.40 (t, *J* = 7.5 Hz, 1H), 7.20 (d, *J* = 7.3 Hz, 1H), 6.21 (br s, 1H), 3.19 (d, *J* = 16.3 Hz, 1H), 3.18 (s, 3H), 3.04 (d, *J* = 16.3 Hz, 1H), 2.82 (s, 3H), 1.31 (s, 3H) ppm. **^{13}C NMR** (126 MHz, $CDCl_3$) δ 200.22 (CO), 169.80 (CO), 161.74 (C), 151.59 (CH), 146.69 (C), 132.92, 132.49 (CH), 130.65 (C), 127.05 (CH), 125.47 (CH), 114.89 (CH), 98.46 (C), 58.35 (C), 40.33 (CH_2), 38.43 (CH_3 amide), 34.96 (CH_3 amide), 30.18 (CH_3) ppm. Note: The low intensity of certain signals is presumably attributable to rotamerism. **HRMS** [APCI]: *m/z* calculated for $C_{17}H_{17}INO_2$ $[M + H]^+$: 394.0299, found 394.0286.

Enantioselectivity was determined by chiral HPLC analysis on a Chiralpack IG3 column at rt (Hexane:*i*PrOH = 85:15, 1 mL/min).

A: Racemic sample

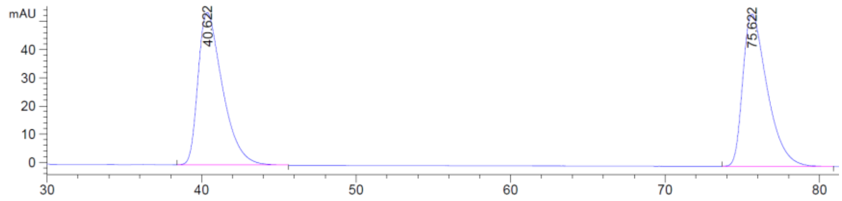

Signal 2: DAD1 B, Sig=254,4 Ref=off

Peak #	RetTime [min]	Type	Width [min]	Area [mAU*s]	Height [mAU]	Area %
1	40.139	BB	1.5097	6610.47070	115.37387	49.1430
2	75.622	BB	1.5662	6841.03809	88.47007	50.8570
Totals :				1.34515e4	203.84394	

B: Asymmetric sample (90:10 er).

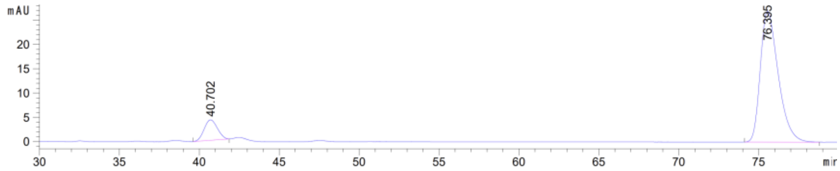

Signal 2: DAD1 B, Sig=254,4 Ref=off

Peak #	RetTime [min]	Type	Width [min]	Area [mAU*s]	Height [mAU]	Area %
1	40.702	BB	0.7128	241.96811	4.20848	10.3520
2	76.395	BB	1.1101	2095.43774	26.92164	89.6480
Totals :				2337.40585	31.13013	

Amide-directed alkenylation (synthesis of **17b**) [15]

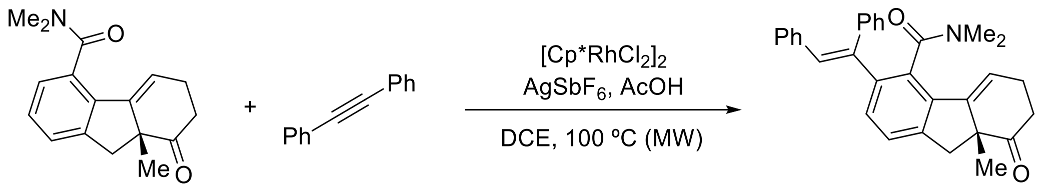

3b **17b**

An oven-dried microwave tube was charged with **5** (22 mg, 0.082 mmol, 1.0 equiv), diphenylacetylene (17.5 mg, 0.1 mmol, 1.2 equiv), $[Cp^*RhCl_2]_2$ (2.5 mg, 0.004 mmol, 5 mol%), $AgSbF_6$ (2.8 mg, 0.008 mmol, 0.1 equiv), AcOH (2.5 mg, 0.041 mmol, 0.5 equiv), and DCE (0.4 mL) sequentially. The reaction mixture was heated at 100 °C in microwave reactor until complete consumption of starting material as indicated by TLC. The mixture was cooled to room temperature, diluted with dichloromethane, filtered through a short pad of Celite and concentrated in vacuo. The crude residue was purified by flash chromatography system (SiO_2-4g, 13 mL/min, 0 → 30% → 40% hexane/EtOAc), to afford 26 mg of **(*R,E*)-6-(1,2-diphenylvinyl)-*N,N*,9a-trimethyl-1-oxo-2,3,9,9a-tetrahydro-1*H*-fluorene-5-carboxamide (17b)** as a brown oil (71% yield). **^{1}H NMR** (300 MHz, $CDCl_3$) δ 7.35 – 7.06 (m, 10H), 7.06 – 6.99 (m, 2H), 6.69 (s, 1H), 6.12 (dd, *J* = 6.4, 3.0 Hz, 1H), 3.30 (d, *J* = 16.8 Hz, 1H), 2.84 – 2.54 (m, 6H), 2.52 – 2.25 (m, 1H), 2.43 (s, 3H), 2.05 (br s, 1H), 1.30 (s, 3H) ppm. **^{13}C NMR** (75 MHz, $CDCl_3$) δ 214.57 (CO), 168.98 (CO), 145.52 (C), 141.93 (C), 141.34 (C), 140.83 (C), 139.87 (C), 137.30 (C), 134.06 (C), 131.08 (CH), 130.95 (CH), 130.43 (CH), 129.72 (CH), 129.60 (CH), 128.22 (CH), 128.14 (CH), 128.03 (CH), 127.44 (CH), 126.96 (CH), 126.20 (CH), 125.93 (CH), 119.75 (CH), 119.57 (CH), 55.20 (C), 40.10 (CH_2), 37.53 (CH_3 amide), 35.54 (CH_2), 35.51 (CH_2), 34.14 (CH_3 amide), 24.94 (CH_3), 24.24 (CH_2) ppm. Note: most carbon signals are duplicated due to rotamerism. **HRMS** [APCI]: *m/z* calculated for $C_{31}H_{30}NO_2$ $[M + H]^+$: 448.2271, found 448.2279.

Selective amide reduction (synthesis of **18b**)[16]

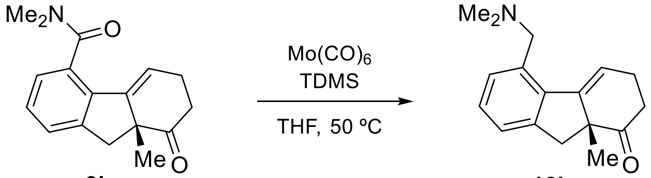

3b **18b**

$Mo(CO)_6$ (1.2 mg, 0.004 mmol, 5 mol%) and **3b** (24 mg, 0.089 mmol, 1.0 equiv) were added to an oven dried 10 mL sealed tube equipped with a magnetic stirring bar. To the sealed tube, dry THF (2 mL) was added and the atmosphere was exchanged for argon. The reaction mixture was heated at 80 °C for 10 minutes to activate the catalyst and was cooled at 50 °C. TMDS (tetramethyldisiloxane, 48 mg, 63 µL, 0.356 mmol, 4.0 equiv) was added and the reaction was

performed until complete consumption of starting material as indicated by TLC. The crude reaction was transferred to a round bottom flask and evaporated. The crude residue was purified by flash chromatography (SiO_2-4g, 13 mL/min, 0 → 70% → 100% hexane/EtOAc), to afford 14 mg of **(*R*)-5-((dimethylamino)methyl)-9a-methyl-2,3,9,9a-tetrahydro-1*H*-fluoren-1-one 18b** as a colorless oil (62% yield). **^{1}H NMR** (300 MHz, $CDCl_3$) δ 7.22 – 7.13 (m, 3H), 6.44 (dd, *J* = 6.5, 2.9 Hz, 1H), 3.80 (d, *J* = 12.7 Hz, 1H), 3.37 (d, *J* = 16.4 Hz, 1H), 3.25 (d, *J* = 12.7 Hz, 1H), 2.91 – 2.55 (m, 4H), 2.48 – 2.35 (m, 1H), 2.30 (s, 6H), 1.28 (s, 3H) ppm. **^{13}C NMR** (75 MHz, $CDCl_3$) δ 215.33 (CO), 146.84 (C), 143.23 (C), 137.25 (C), 129.39 (CH), 127.68 (CH), 124.89 (CH), 121.70 (CH), 62.41 (CH_2), 55.40 (C), 45.49 (CH_3), 40.72 (CH_2), 35.49 (CH_2), 24.58 (CH_3), 24.42 (CH_2) ppm. Note: The low intensity of certain signals is presumably attributable to rotamerism. **HRMS** [APCI]: *m/z* calculated for $C_{17}H_{22}NO$ $[M + H]^+$: 256.1696, found 256.1690.

Enantioselectivity was determined by chiral HPLC analysis on a Chiralpack IE3 column at rt (Hexane:*i*PrOH = 97:3, 1 mL/min).

A: Racemic sample

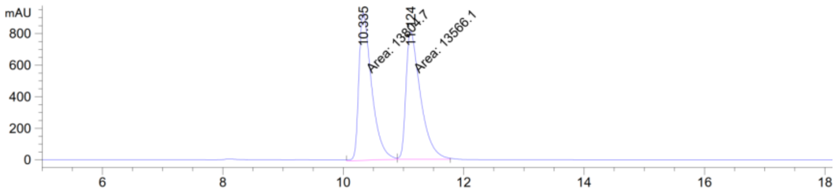

Signal 2: DAD1 B, Sig=254,4 Ref=off

Peak #	RetTime [min]	Type	Width [min]	Area [mAU*s]	Height [mAU]	Area %
1	10.335	MM	0.2465	1.38047e4	933.24780	50.4358
2	11.124	MM	0.2796	1.35661e4	808.64111	49.5642
Totals :				2.73708e4	1741.88892	

B: Asymmetric sample (90:10 er).

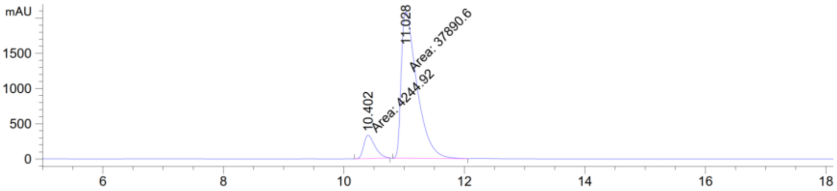

Signal 2: DAD1 B, Sig=254,4 Ref=off

Peak #	RetTime [min]	Type	Width [min]	Area [mAU*s]	Height [mAU]	Area %
1	10.402	MM	0.2141	4244.91748	330.46732	10.0744
2	11.028	MM	0.3031	3.78906e4	2083.59814	89.9256
Totals :				4.21355e4	2414.06546	

Selective directing group manipulation: Knoevenagel condensation (synthesis of **19b**)[17]

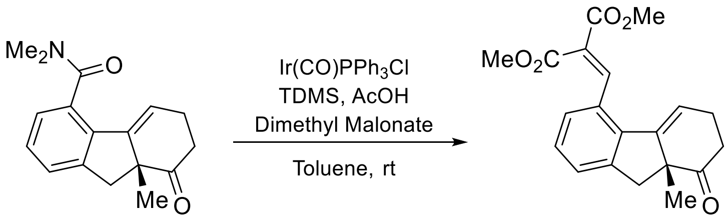

3b 19b

To a solution of **3b** (32 mg, 0.12 mmol, 1.0 equiv) in anhydrous toluene (0.6 mL) was added Vaska's complex (1.9 mg, 0.002 mmol, 2 mol%). The resulting suspension was stirred for 5 minutes at rt. To the resulting yellow solution, TMDS (tetramethyldisiloxane, 22 mg, 0.166 mmol, 1.4 equiv) was added in one portion and the reaction mixture stirred for 10 minutes until H_2 gas evolution had ceased and the solution turned colorless. Then dimethyl malonate (28 mg, 0.21 mmol, 24.5 µL, 1.8 equiv) was added in one portion and the mixture was stirred for 5 minutes. Finally, AcOH (8.6 mg, 8.2 µL, 0.14 mmol, 1.2 equiv) was added in one portion and the mixture was stirred at rt until complete consumption of starting material as indicated by TLC. The reaction was quenched with sat. aq. NH_4Cl solution and extracted with

EtOAc. The combined organic layers were washed with brine, dried over anhydrous Na_2SO_4, filtered, and concentrated under reduced pressure. The crude residue was purified by flash chromatography (SiO_2-4g, 13 mL/min, 0 $\rightarrow$ 20% hexane/EtOAc), to afford 14 mg of dimethyl **(*R*)-2-((9a-methyl-1-oxo-2,3,9,9a-tetrahydro-1*H*-fluoren-5-yl)methylene)malonate 19b** as a colorless oil (45% yield). **^{1}H NMR** (500 MHz, $CDCl_3$) δ 8.13 (s, 1H), 7.30 (t, *J* = 4.3 Hz, 1H), 7.20 (d, *J* = 4.7 Hz, 2H), 6.11 (dd, *J* = 6.3, 2.8 Hz, 1H), 3.89 (s, 3H), 3.73 (s, 3H), 3.38 (d, *J* = 16.6 Hz, 1H), 2.90 – 2.58 (m, 4H), 2.45 – 2.27 (m, 1H), 1.32 (s, 3H) ppm. **^{13}C NMR** (126 MHz, $CDCl_3$) δ 214.31 (CO), 166.65 (CO), 164.59 (CO), 146.46 (C), 143.02 (C), 142.51 (CH), 137.50 (C), 129.73 (C), 128.29 (C), 127.48 (CH), 127.39 (CH), 126.50 (CH), 123.71 (CH), 55.12 (C), 52.88 (CH_3), 52.67 (CH_3), 40.47 (CH_2), 35.18 (CH_2), 24.74 (CH_3), 24.71 (CH_2) ppm. Note: The low intensity of certain signals is presumably attributable to rotamerism. **HRMS** [APCI]: *m/z* calculated for $C_{20}H_{21}O_5$ $[M + H]^+$: 341.1384, not found.

Enantioselectivity was determined by chiral HPLC analysis on a Chiralpack IG3 column at rt (Hexane:*i*PrOH = 90:10, 1 mL/min).

A: Racemic sample

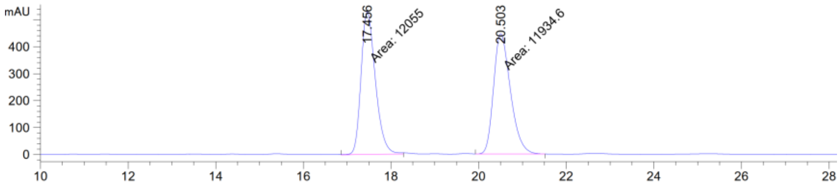

Signal 2: DAD1 B, Sig=254,4 Ref=off

Peak #	RetTime [min]	Type	Width [min]	Area [mAU*s]	Height [mAU]	Area %
1	17.456	MM	0.3789	1.20550e4	530.29974	50.2508
2	20.503	MM	0.4457	1.19346e4	446.27872	49.7492
Totals :				2.39896e4	976.57846	

B: Asymmetric sample (90:10 er).

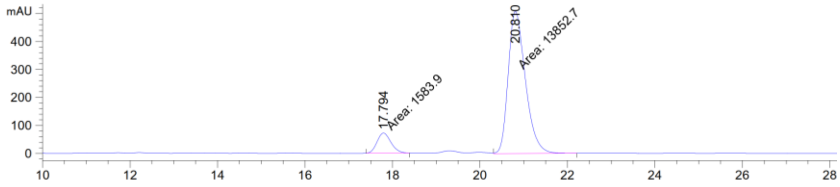

Signal 2: DAD1 B, Sig=254,4 Ref=off

Peak #	RetTime [min]	Type	Width [min]	Area [mAU*s]	Height [mAU]	Area %
1	17.794	MM	0.3679	1583.90234	71.74686	10.2607
2	20.810	MM	0.4538	1.38527e4	508.75165	89.7393
Totals :				1.54366e4	580.49851	

Amide hydrolysis (synthesis of **20b'**)

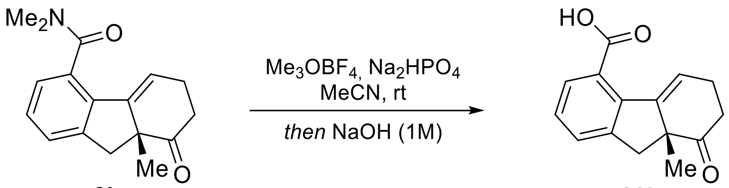

3b **20b**

To a stirring solution of **3b** (191.0 mg, 0.71 mmol) in CH_3CN (2.5 mL) at rt, was added Na_2HPO_4 (151.0 mg, 1.1 mmol), followed by Me_3OBF_4 (315.0 mg, 2.1 mmol). After consumption of the amide was complete as indicated by TLC analysis (30 min), a 1.0 M aqueous NaOH solution (10 mL) was added. The resulting mixture was stirred for 16 h at rt and then 10 mL of HCl 1.0 M were added and the mixture was extracted with EtOAc (3 x 50 mL). The combined organic phases were concentrated to afford 144 mg of **(*R*)-9a-methyl-1-oxo-2,3,9,9a-tetrahydro-1H-fluorene-5-carboxylic acid 20b'** as yellow solid (84% yield), that was used without further purification. **^{1}H NMR** (300 MHz, $CDCl_3$) δ 10.59 (br, 1H), 7.82 (d,

J = 7.7 Hz, 1H), 7.44 (d, J = 7.4 Hz, 1H), 7.26 (t, J = 7.5 Hz, 1H), 6.94 (dd, J = 6.2, 3.1 Hz, 1H), 3.32 (d, J = 16.6 Hz, 1H), 2.89 – 2.69 (m, 3H), 2.69 – 2.50 (m, 1H), 2.44 – 2.27 (m, 1H), 1.33 (s, 3H) ppm. **^{13}C NMR** (75 MHz, $CDCl_3$) δ ^{13}C NMR (75 MHz, cdcl$_3$) δ 215.1 (CO), 173.2 (COOH), 144.1 (C), 143.9 (C), 138.2 (C), 130.5 (CH), 130.1 (CH), 127.8 (CH), 126.2 (C), 124.6 (CH), 54.9 (C), 40.2 (CH_2), 35.0 (CH_2), 25.1 (CH_3), 24.8 (CH_2) ppm. **HRMS** [APCI]: *m/z* calculated for $C_{15}H_{15}O_3$ $[M + H]^+$: 243.1016, found 243.1008.

Directing group removal by decarboxylation of 20b' (synthesis of **20b**)[18]

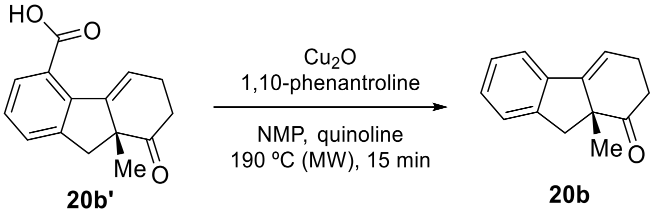

To solution of **20b'** (40.0 mg, 0.165 mmol) in a mixture of NMP (1-methyl-Pyrrolidinone, 0.55 mL) and quinoline (0.165 mL) in a microwave vial was added Cu_2O (2.4 mg, 0.017 mmol) and 1,10-phenantroline (6.0 mg, 0.033 mmol). The vial was heated at 190 °C for 15 mins in a microwave reactor. The reaction mixture was diluted with 15 mL of EtOAc and 30 mL of aqueous solution of HCL 1 M. After 3 extractions with EtOAc (25 mL each), the combined organic phases were dried over anhydrous Na_2SO_4, filtered, and concentrated under reduced pressure. The crude residue was purified by flash chromatography (SiO_2-4g, 13 mL/min, 0 → 10% hexane/EtOAc), to afford 23 mg of **(*R*)-9a-methyl-2,3,9,9a-tetrahydro-1H-fluoren-1-one 20b** as pale-yellow solid (69% yield). **^{1}H NMR** (500 MHz, $CDCl_3$) δ 7.45 (dd, J = 6.4, 2.4 Hz, 1H), 7.28 – 7.26 (m, 1H), 7.25 – 7.18 (m, 2H), 6.27 (dd, J = 6.7, 2.7 Hz, 1H), 3.39 (d, J = 16.5 Hz, 1H), 2.81 – 2.71 (m, 3H), 2.64 – 2.56 (m, 1H), 2.40 – 2.31 (m, 1H), 1.32 (s, 3H) ppm. **^{13}C NMR** (126 MHz, $CDCl_3$) δ 214.9 (CO), 147.3 (C), 142.2 (C), 138.1 (C), 128.6 (CH), 127.0 (CH), 126.0 (CH), 121.1 (CH), 116.3 (CH), 55.1 (C), 40.4 (CH_2), 35.7 (CH_2), 24.3 (CH_3), 23.6 (CH_2) ppm. **HRMS** [APCI]: *m/z* calculated for $C_{14}H_{15}O$ $[M + H]^+$: 199.1117, found 199.1113.

Decarboxylative Suzuki-Miyaura cross-coupling (synthesis of **21b**)[19]

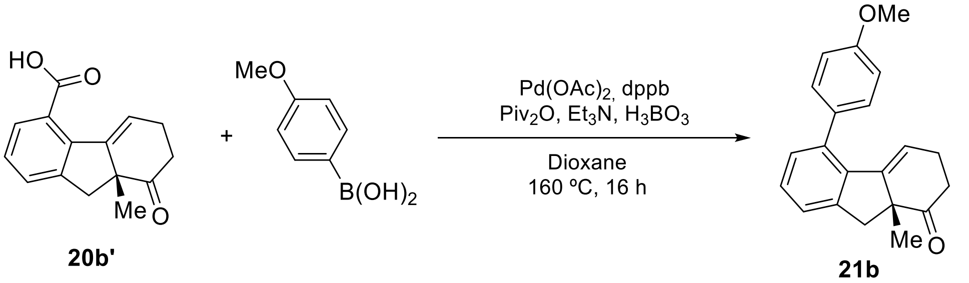

In a sealed tube, a mixture of compound **20b'** (40.0 mg, 0.17 mmol), *p*-MeO-phenylboronic acid (37.6, 0.25 mmol), $Pd(OAc)_2$ (2.8 mg, 0.012 mmol), dppb (10.6 mg, 0.024 mmol), triethylamine (34 μL, 0.25 mmol), boric acid (15.3 mg, 0.25 mmol) and Piv_2O (46.1, 0.25 mmol) in dioxane (0.83 mL) was heated at 160 °C for 16 h. Then, water (20 mL) was added and the resulting crude mixture was extracted with EtOAc (3 x 25 mL). The combined organic phases were dried over anhydrous Na_2SO_4, filtered, and concentrated under reduced pressure. The crude residue was purified by flash

chromatography (SiO_2-4g, 13 mL/min, 0 → 20% hexane/EtOAc), to afford 37 mg of **(*R*)-5-(4-methoxyphenyl)-9a-methyl-2,3,9,9a-tetrahydro-1H-fluoren-1-one 21b** as pale-yellow oil (71% yield). **^{1}H NMR** (500 MHz, $CDCl_3$) δ 7.33 (d, *J* = 8.7 Hz, 2H), 7.23 (d, *J* = 5.0 Hz, 2H), 7.05 (t, *J* = 4.3 Hz, 1H), 6.96 (d, *J* = 8.7 Hz, 2H), 5.53 (dd, *J* = 6.4, 3.1 Hz, 1H), 3.87 (s, 3H), 3.37 (d, *J* = 16.5 Hz, 1H), 2.81 (d, *J* = 16.5 Hz, 1H), 2.71 – 2.64 (m, 1H), 2.63 – 2.53 (m, 1H), 2.37 – 2.28 (m, 1H), 2.28 – 2.20 (m, 1H), 1.39 (s, 3H) ppm. **^{13}C NMR** (126 MHz, $CDCl_3$) δ 215.2 (CO), 159.1 (C), 146.3 (C), 142.8 (C), 138.6 (C), 135.3 (C), 133.4 (C), 130.1 (CH), 129.1 (CH), 128.1 (CH), 124.7 (CH), 119.9 (CH), 114.0 (CH), 55.4 (CH_3), 54.6 (C), 40.5 (CH_2), 35.4 (CH_2), 24.9 (CH_3), 24.2 (CH_2) ppm. **HRMS** [APCI]: *m/z* calculated for $C_{21}H_{21}O_2$ $[M + H]^+$: 305.1536, found 305.1536.

Enantioselectivity was determined by chiral HPLC analysis on a Chiralpack IG3 column at rt (Hexane:*i*PrOH = 95:15, 1 mL/min).

A: <u>Racemic sample</u>

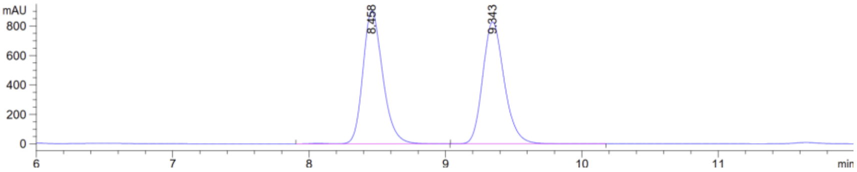

Signal 3: DAD1 C, Sig=210,4 Ref=off

Peak #	RetTime [min]	Type	Width [min]	Area [mAU*s]	Height [mAU]	Area %
1	8.458	VB R	0.1556	9126.51270	903.12872	50.2188
2	9.343	BB	0.1676	9046.98633	827.25220	49.7812
Totals :				1.81735e4	1730.38092	

B: <u>Asymmetric sample</u> (90:10 er).

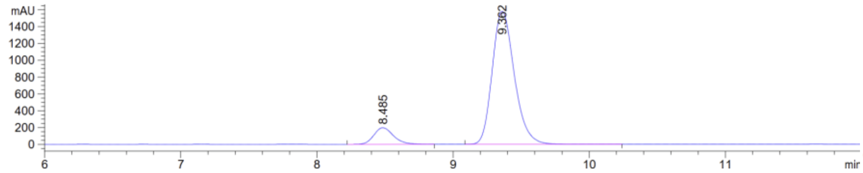

Signal 3: DAD1 C, Sig=210,4 Ref=off

Peak #	RetTime [min]	Type	Width [min]	Area [mAU*s]	Height [mAU]	Area %
1	8.485	BB	0.1523	1967.68396	197.44473	9.9383
2	9.362	BB	0.1736	1.78312e4	1580.50500	90.0617
Totals :				1.97989e4	1777.94974	

7. Mechanistic studies with Et_3SiH

Synthesis of Ir(COD)((*R*)-DTBM-Segphos)BAr^F_4 (Ir1)

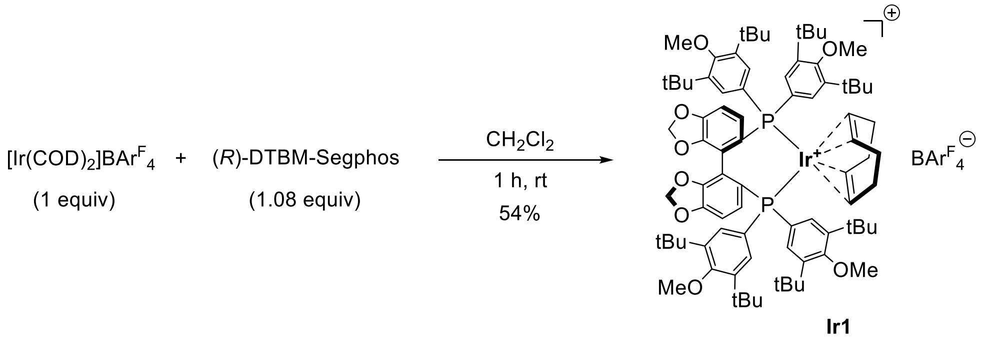

Freshly prepared $Ir(COD)_2BAr^F_4$ (100 mg, 0.08 mmol) and (*R*)-DTBM-Segphos (102 mg, 0.086 mmol) were dissolved in anhydrous CH_2Cl_2 (2 mL) and stirred under argon for 1 hour at rt. Then, volatiles were removed under reduced pressure, and pentane (4 mL) was added and the mixture was sonicated. After 30 minutes, a red oil deposited and the pentane was decanted. Another 4 mL of pentane were added, and the same procedure was repeated twice. Finally, volatiles were removed under vacuum affording Ir(COD)((*R*)-DTBM-Segphos)BAr^F_4 (**Ir1**) in 54% yield as a fluffy red solid. Crystals suitable for X-Ray diffraction were obtained from a solution of **Ir1** in CH_2Cl_2 layered with pentane. **^{1}H NMR** (300 MHz, CD_2Cl_2) δ 8.16 (br d, *J* = 11.5 Hz, 2H), 7.77 (s, 8H), 7.61 (s, 4H), 7.46 (t, *J* = 9.2 Hz, 2H), 7.12 (br s, 4H), 6.92 (br s, 2H), 6.67 (d, *J* = 8.1 Hz, 2H), 5.81 (s, 2H), 5.63 (s, 2H), 4.43 (br m, 2H), 3.77 (br s, 14H), 2.57 (br m, 4H), 1.89 – 1.29 (m, 76H) ppm. **^{13}C NMR** (126 MHz, CD_2Cl_2) δ 137.2 (CH), 135.2 (CH), 133.7 (CH), 132.3 (CH), 127.4 (CH), 117.9 (CH), 108.3 (CH), 101.6 (O-CH_2-O), 90.2 (CH), 88.5 (CH), 65.0 (O-CH_3), 64.9 (O-CH_3), 36.5 (CH_2), 32.5 (CH_3), 32.0 (CH_3), 31.8 (CH_3), 26.3 (CH_2) ppm. Note: Quaternary carbons were omitted for clarity. **^{31}P NMR** (202 MHz, CD_2Cl_2) δ 15.1 (s, 2P) ppm. **^{19}F NMR** (471 MHz, CD_2Cl_2) δ -62.9 (s, 24F) ppm. This complex showed the same catalytic competence than the catalyst formed *in situ* with Ir(COD)BAr^F_4 and (*R*)-DTBM-Segphos.

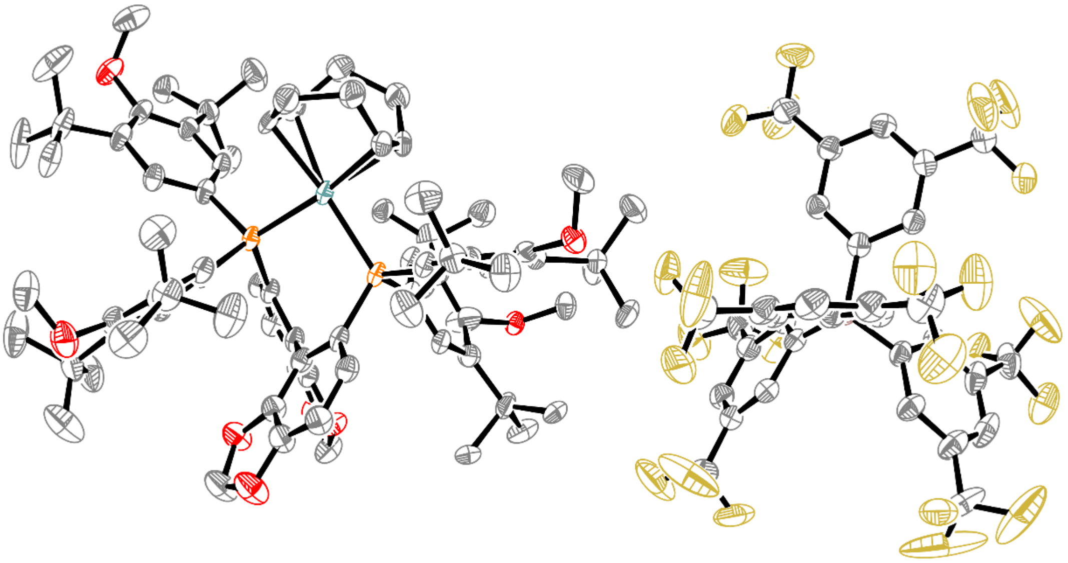

X-ray structure of **Ir1** (CCDC 2513813)

HRMS was measured in a *timsTOF pro 2* equipped with an ESI source, using dioxane as carrier. A capillary voltage of 3.5 kV was used in the positive ESI(+) scan mode. The cone voltage was adjusted to a low value (typically Uc= 5–15 V) to control the extent of fragmentation in the source region. Chemical identification of the Ir-containing species was facilitated by the characteristic isotopic pattern at natural abundance of Ir and it was carried out by comparison of the isotope experimental and theoretical patterns using *SmartFormula* software.

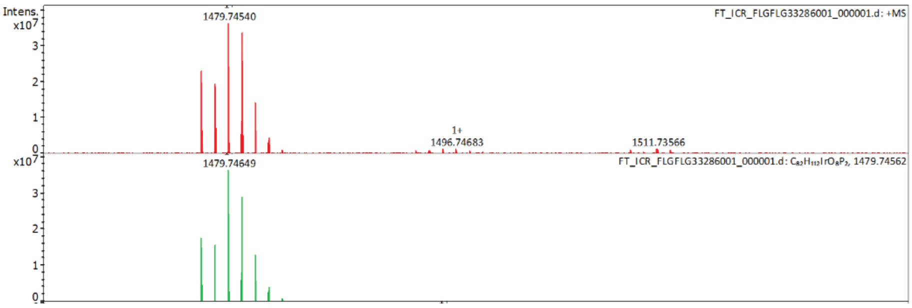

Meas. m/z	#	Ion Formula	Score	m/z	err [ppm]	Mean err [ppm]	mSigma	rdb (neutral)	e⁻ Conf	N-Rule
1479.745403	1	C82H112IrO8P2	100.00	1479.745618	0.7	0.3	93.6	32.5	odd	-

HRMS (ESI): *m/z* calculated for $C_{82}H_{112}IrO_8P_2$ $[M]^+$: 1479.74649, found 1489.74540.

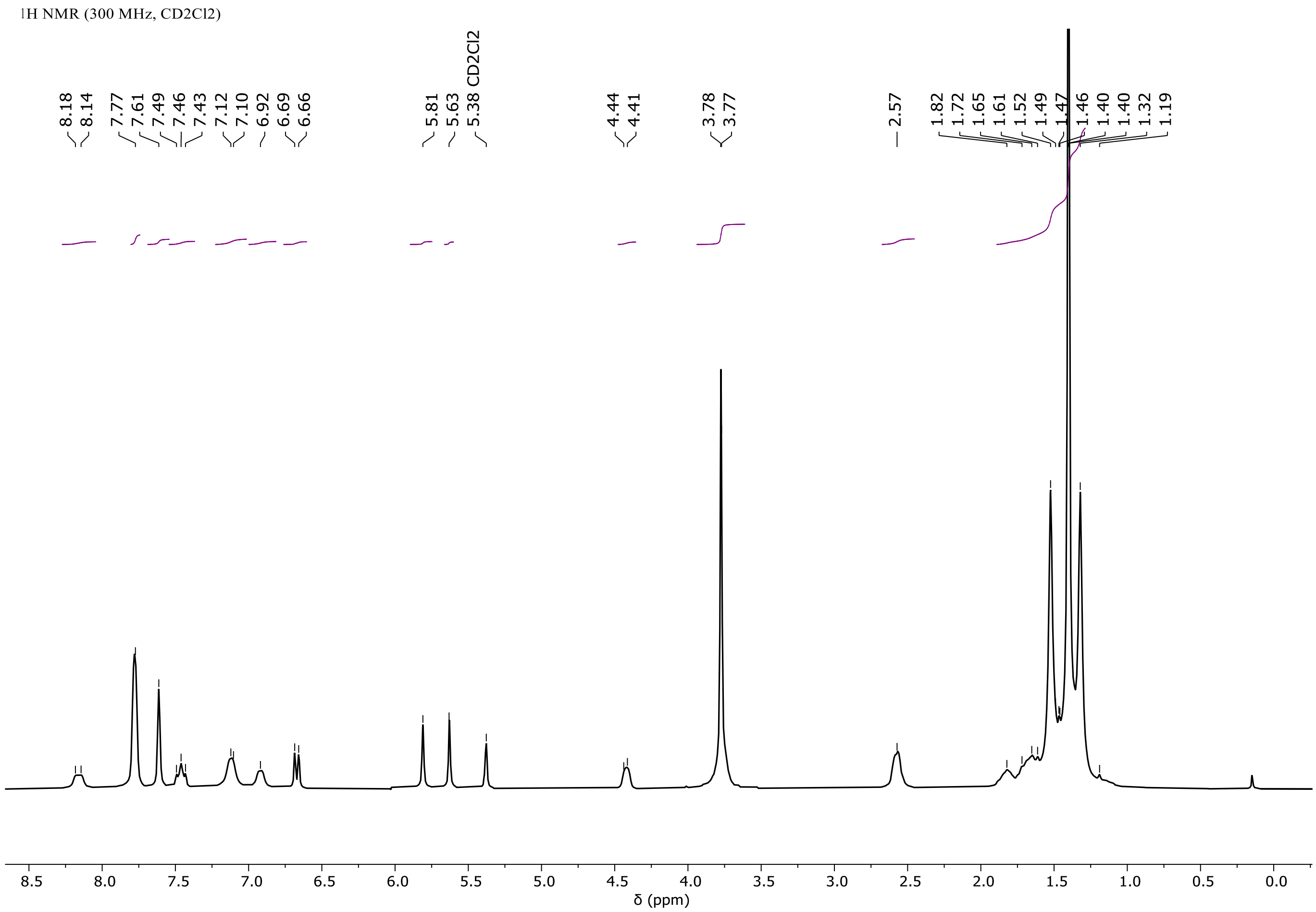

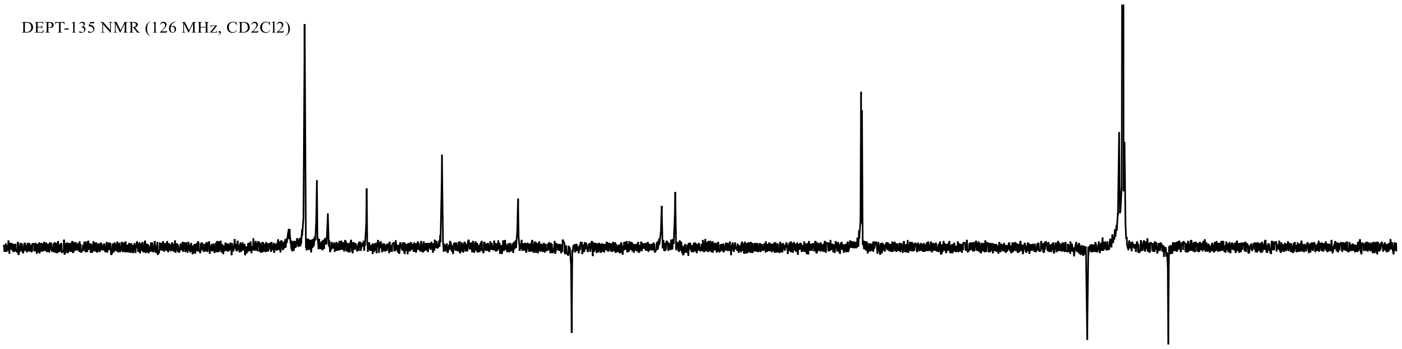

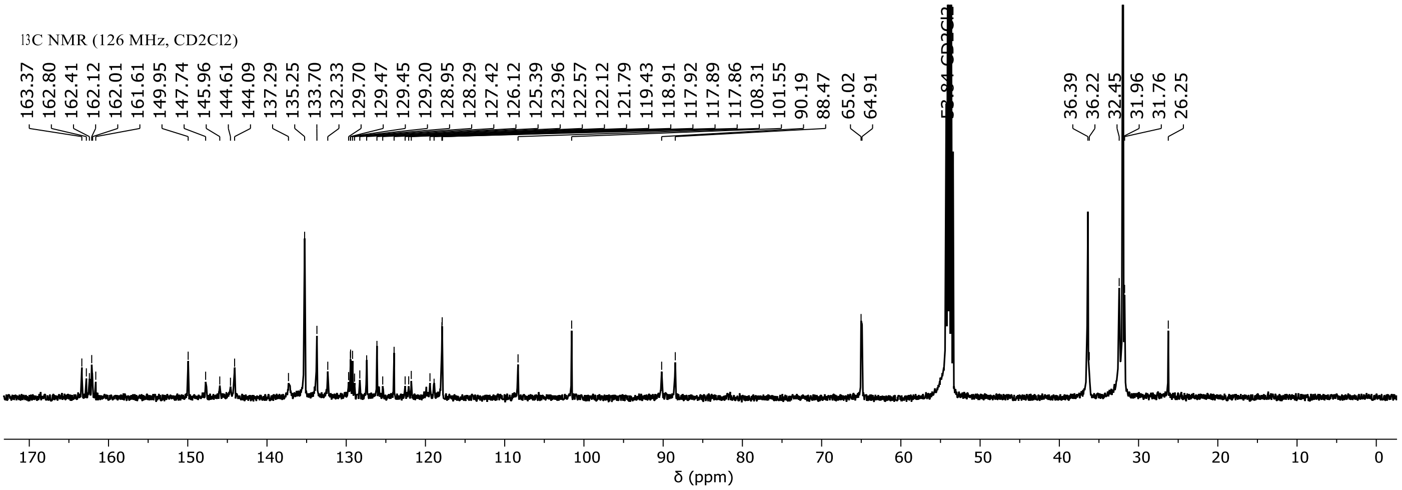

Reaction of Ir1 with Et_3SiH: Isolation of Ir2

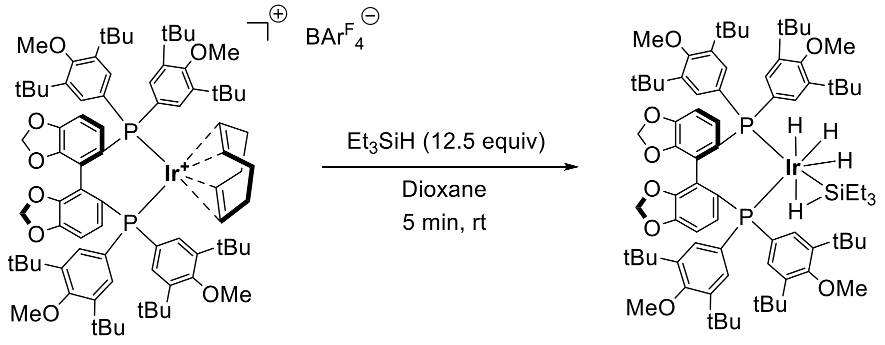

Ir1 **Ir2**

Ir1 (16.1 mg, 0.007 mmol) was dissolved in 1,4-dioxane (0.56 mL). Triethylsilane (13.9 μL, 0.088 mmol, 12.5 equiv) were added and the mixture was stirred until the solution becomes yellow (5 min approx.). [NOTE: 12.5 equivalents of Et_3SiH relative to **Ir1** correspond to 1.25 equivalents with respect to the substrate when using a 10 mol% catalyst loading]. ^{1}H-NMR was measured using a glass capillary with C_6D_6 as internal standard. **^{1}H NMR** (500 MHz, Dioxane and C_6D_6 as internal standard, partial data) δ -10.8 (t, *J* = 11.8 Hz, 4H) ppm. **^{31}P{1H} NMR** (202 MHz, Dioxane and C_6D_6 as internal standard) δ 8.88 (s, 2P) ppm. The obtained mixture was bubbled with argon to remove all volatiles (1,4-dioxane and Triethylsilane). The residue was extracted with pentane (1 x 1mL), and pentane was evaporated under reduced pressure, obtaining a mixture of **Ir2**, cyclooctane and hexaethyldisiloxane. **^{1}H NMR** (300 MHz, C_6D_6) δ 8.42 (s, 2H), 7.98 – 7.63 (m, 5H), 7.47 (q, *J* = 10.3 Hz, 2H), 6.38 (d, *J* = 8.1 Hz, 2H), 5.07 (d, *J* = 16.8 Hz, 4H), 3.45 (d, *J* = 11.3 Hz, 12H), 0.88 – 0.82 (m, 9H), -10.69 (t, *J* = 12.1 Hz, 4H) ppm [NOTE: One aromatic C-H and the CH_2-Si signal are overlapped]

[NOTE: The 4 Ir-hydrides appear equivalent by NMR, possibly due to rapid exchange; the same behavior was observed by Schley and coworkers[20,21]][NOTE: Compound **Ir2** was isolated together with cyclooctane and $Et_3SiOSiEt_3$]. **^{31}P{1H} NMR** (202 MHz, C_6D_6) δ 9.27 (s, 2P) ppm.

HRMS was measured in a *timsTOF pro 2* equipped with an ESI source using water as carrier. A capillary voltage of 3.5 kV was used in the positive ESI(+) scan mode. The cone voltage was adjusted to a low value (typically Uc= 5–15 V) to control the extent of fragmentation in the source region. Chemical identification of the Ir-containing species was facilitated by the characteristic isotopic pattern at natural abundance of Ir and it was carried out by comparison of the isotope experimental and theoretical patterns using *SmartFormula* software.

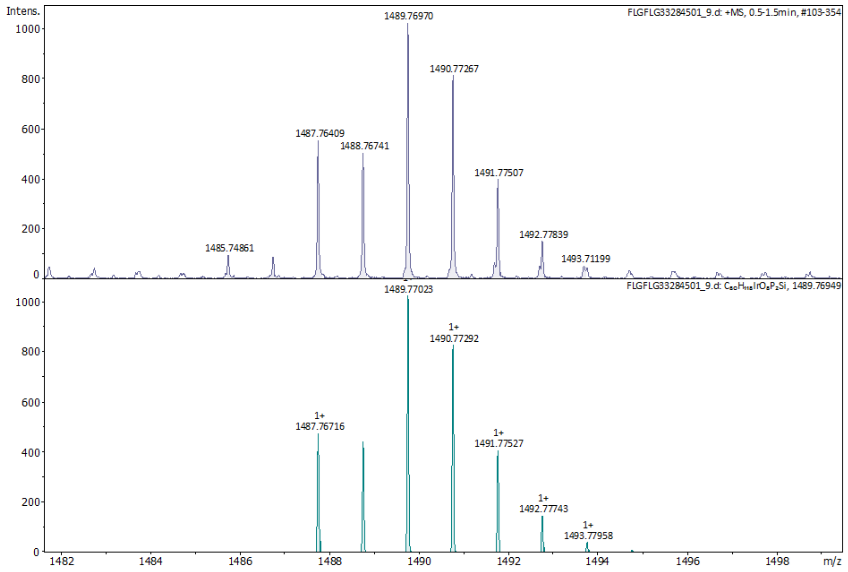

HRMS (ESI): *m/z* calculated for $C_{80}H_{11}IrO_8P_2Si$ [M - H]$^+$: 1489.76949, found 1489.76970.

Meas. m/z	#	Ion Formula	m/z	err [ppm]	mSigma	#mSigma	Score	rdb (neutral)	e⁻ Conf	N-Rule
1489.76970	1	C80H118IrO8P2Si	1489.76949	0.36	41.3	1	100.00	28.5	odd	-

^{1}H-NMR of Ir2 (Dioxane, C_6D_6 as internal standard):

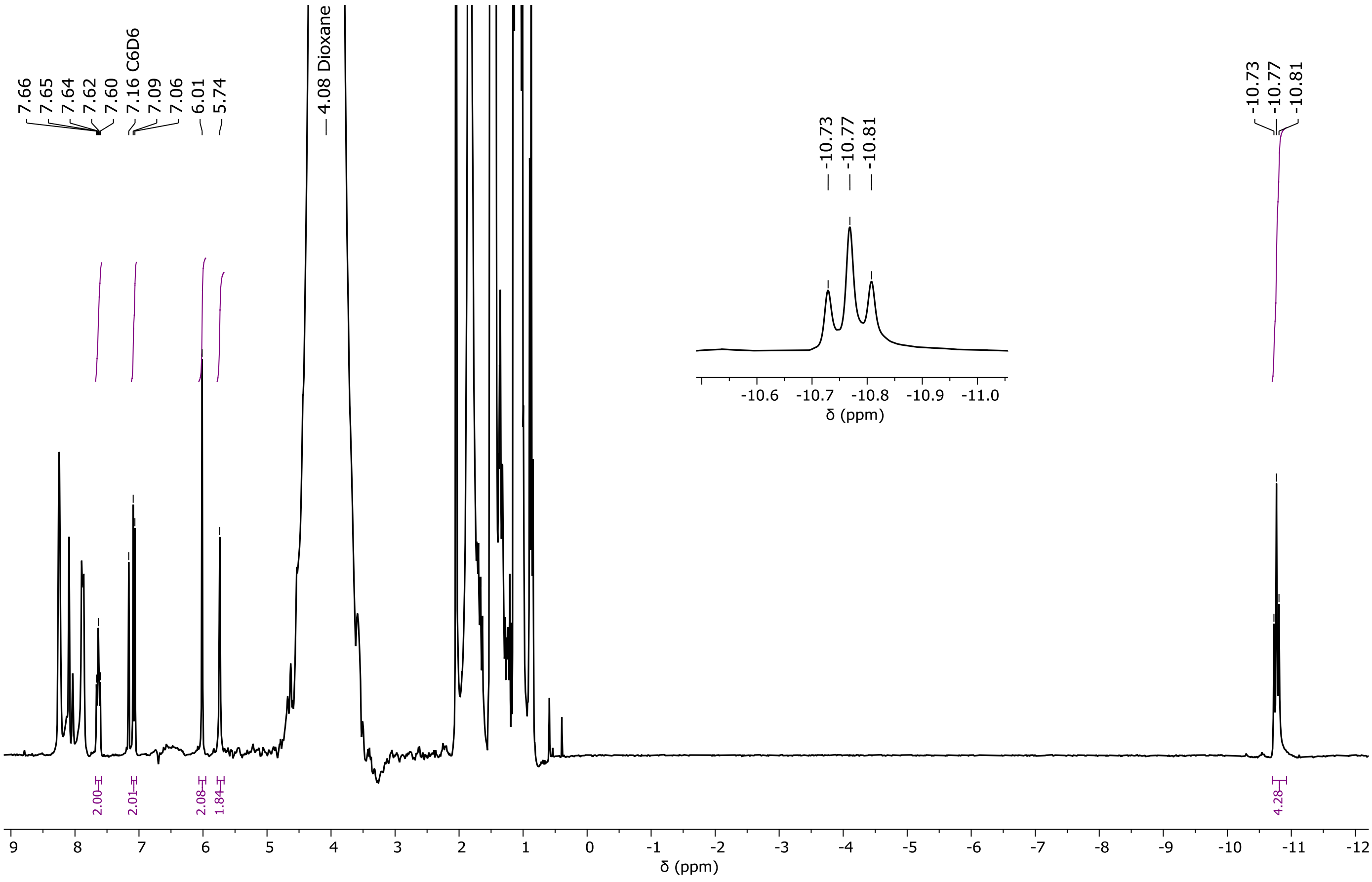

^{1}**H-NMR of Ir2 with solvent PRESAT** (Dioxane, C_6D_6 as internal standard):

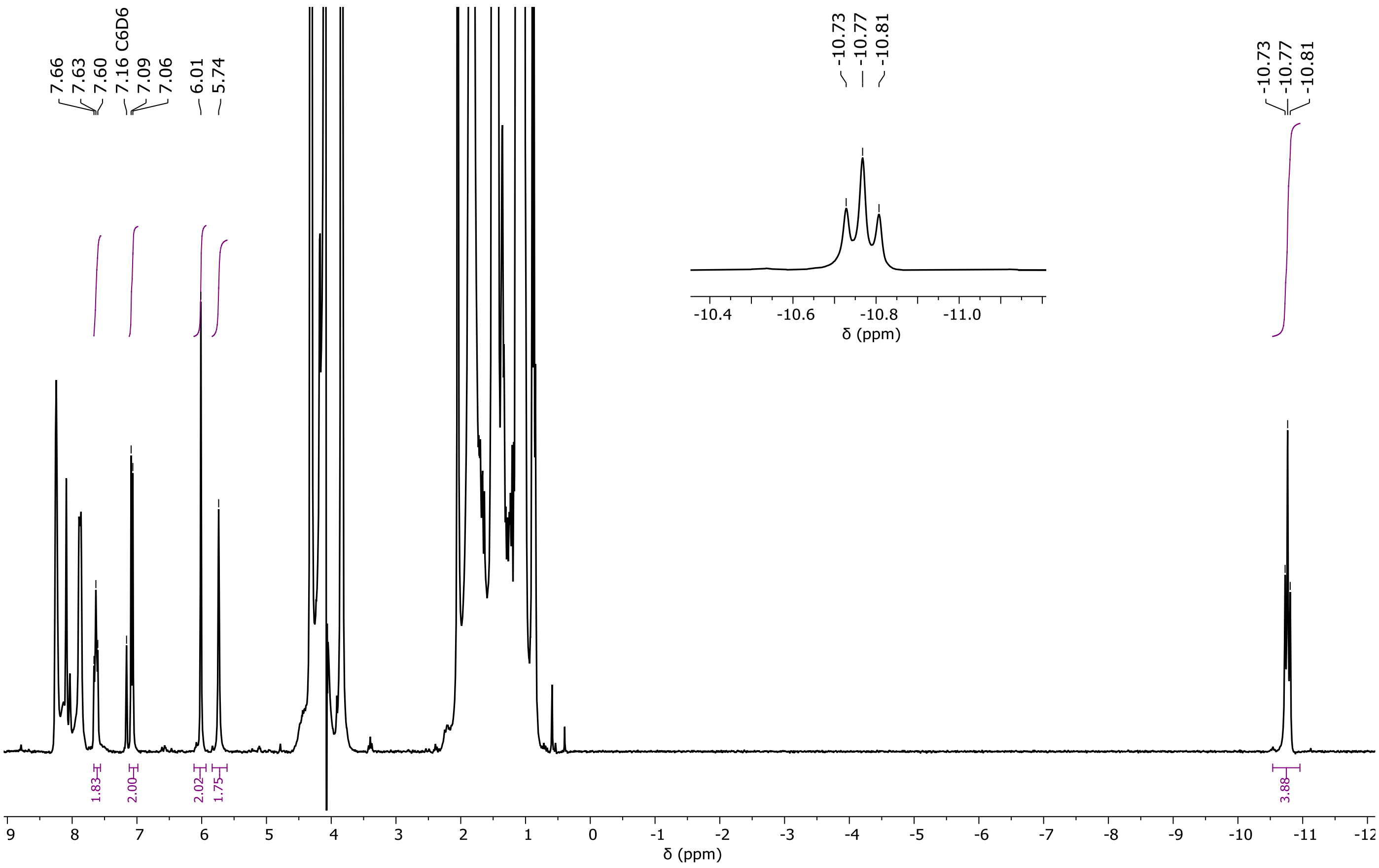

^{1}**H-NMR of Ir2** (C_6D_6):

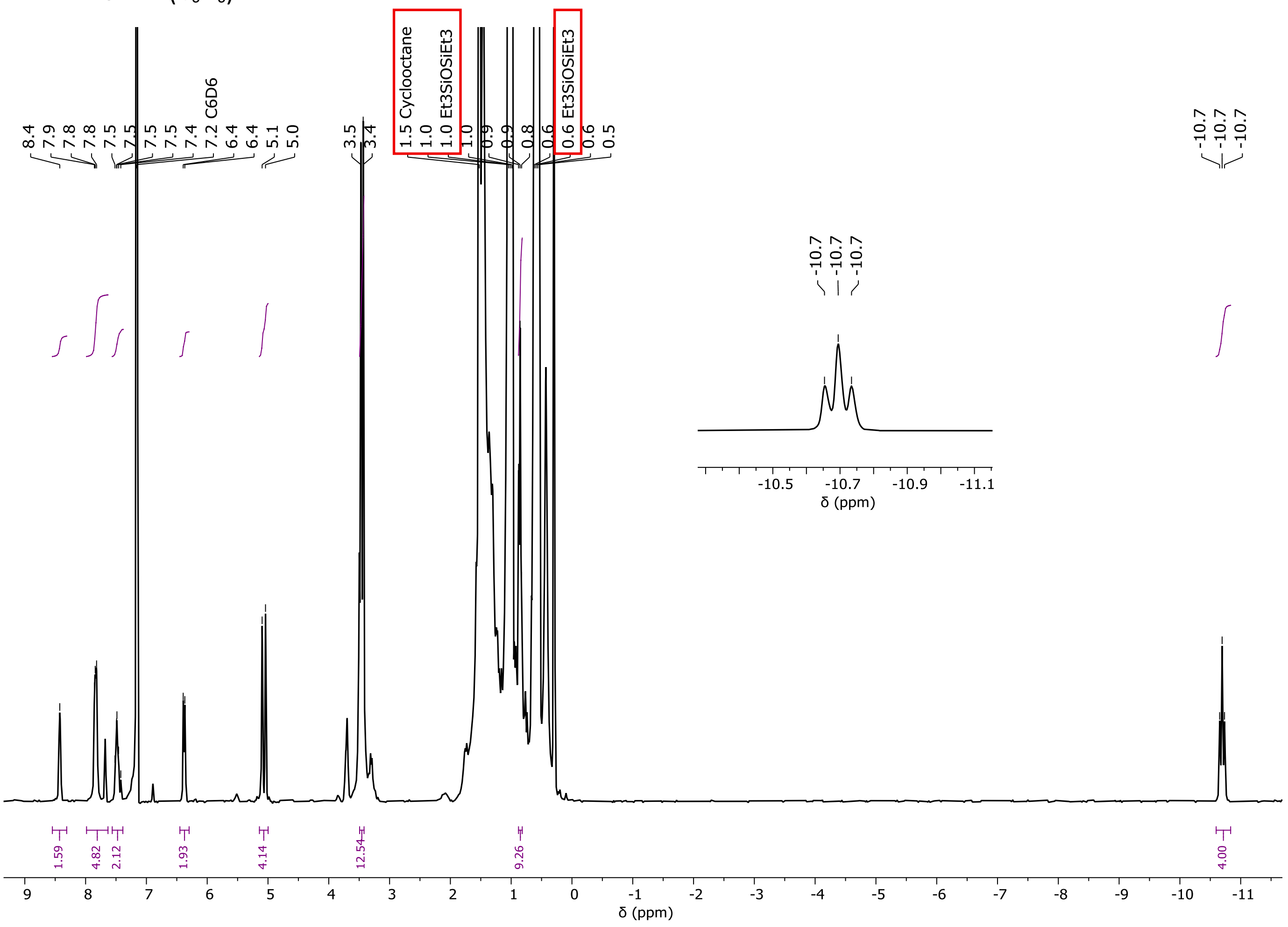

Reaction of Ir1 with Et_3SiD: Detection of Ir2-D

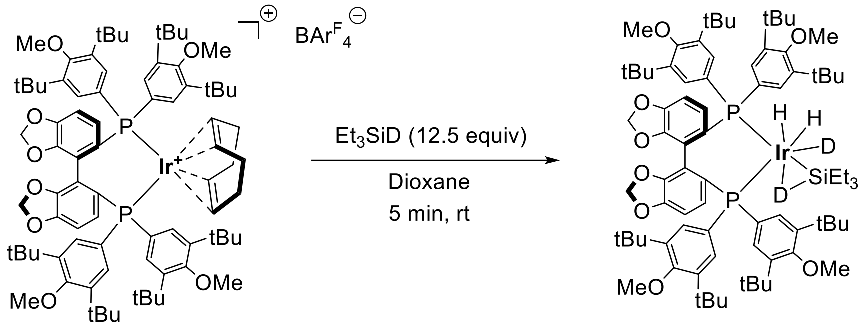

The same experiment was repeated employing Et_3SiD. The characteristic Ir-H signal becomes a multiplet and integrates by 2 protons. This result indicates that approx. 50% of the hydrogens attached to the iridium center comes from Et_3SiD, and the rest could come from the dehydrosilylation of water traces. By ^{2}H-NMR we detected the Iridium deuteride species. **^{1}H NMR** (500 MHz, Dioxane and C_6D_6 as internal standard, partial data) δ -10.8 (m, 2H) ppm. **^{2}H NMR** (500 MHz, Dioxane and C_6D_6 as internal standard) δ -10.7 (s, 2H) ppm. **^{31}P{1H} NMR** (202 MHz, Dioxane and C_6D_6 as internal standard) δ 8.88 (s, 2P) ppm.

^{1}H-NMR of Ir2 generated using Et_3SiD (Dioxane, C_6D_6 as internal standard):

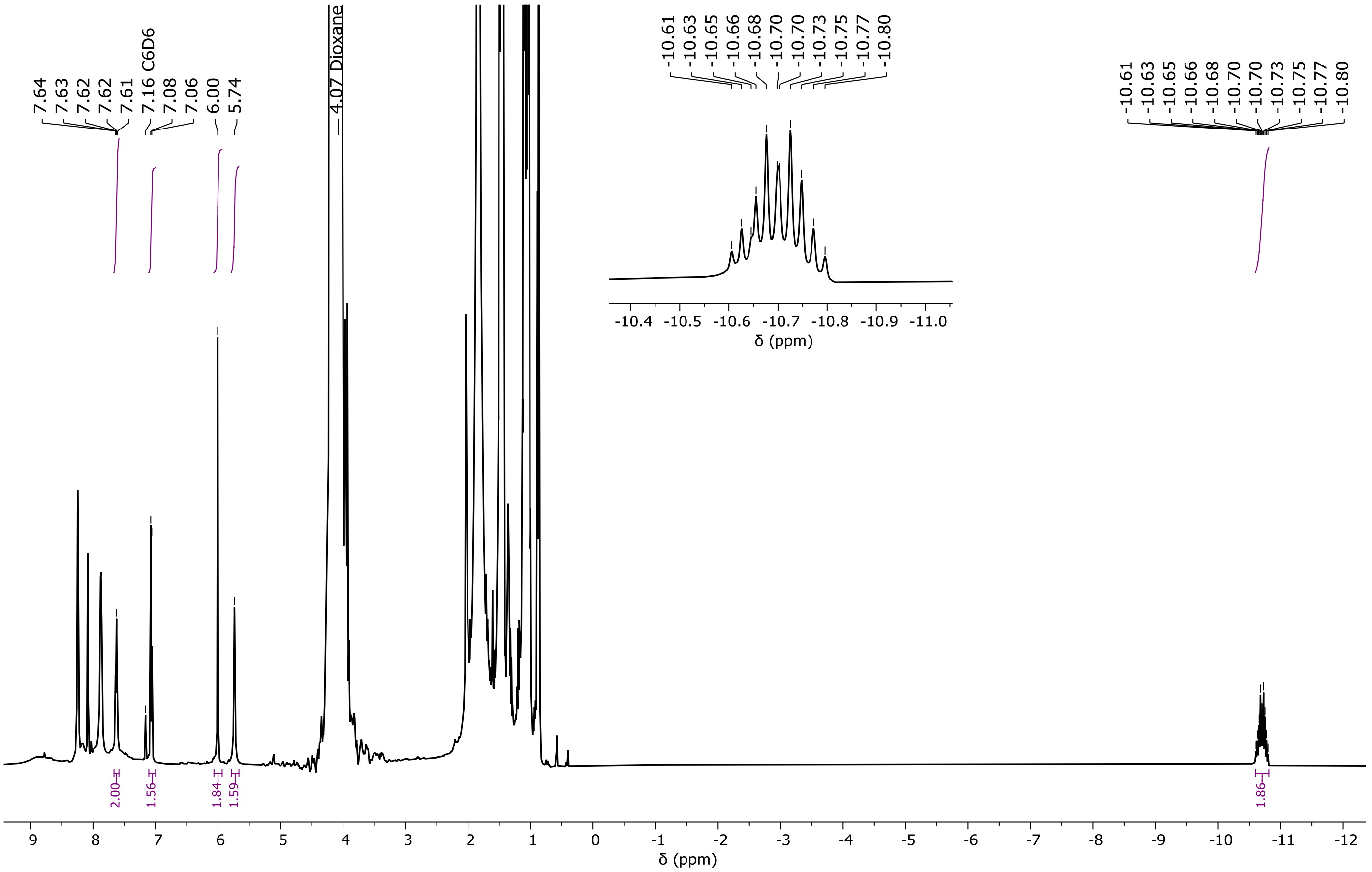

^{1}H-NMR of Ir2 generated using Et_3SiD, with solvent PRESAT (Dioxane, C_6D_6 as internal standard):

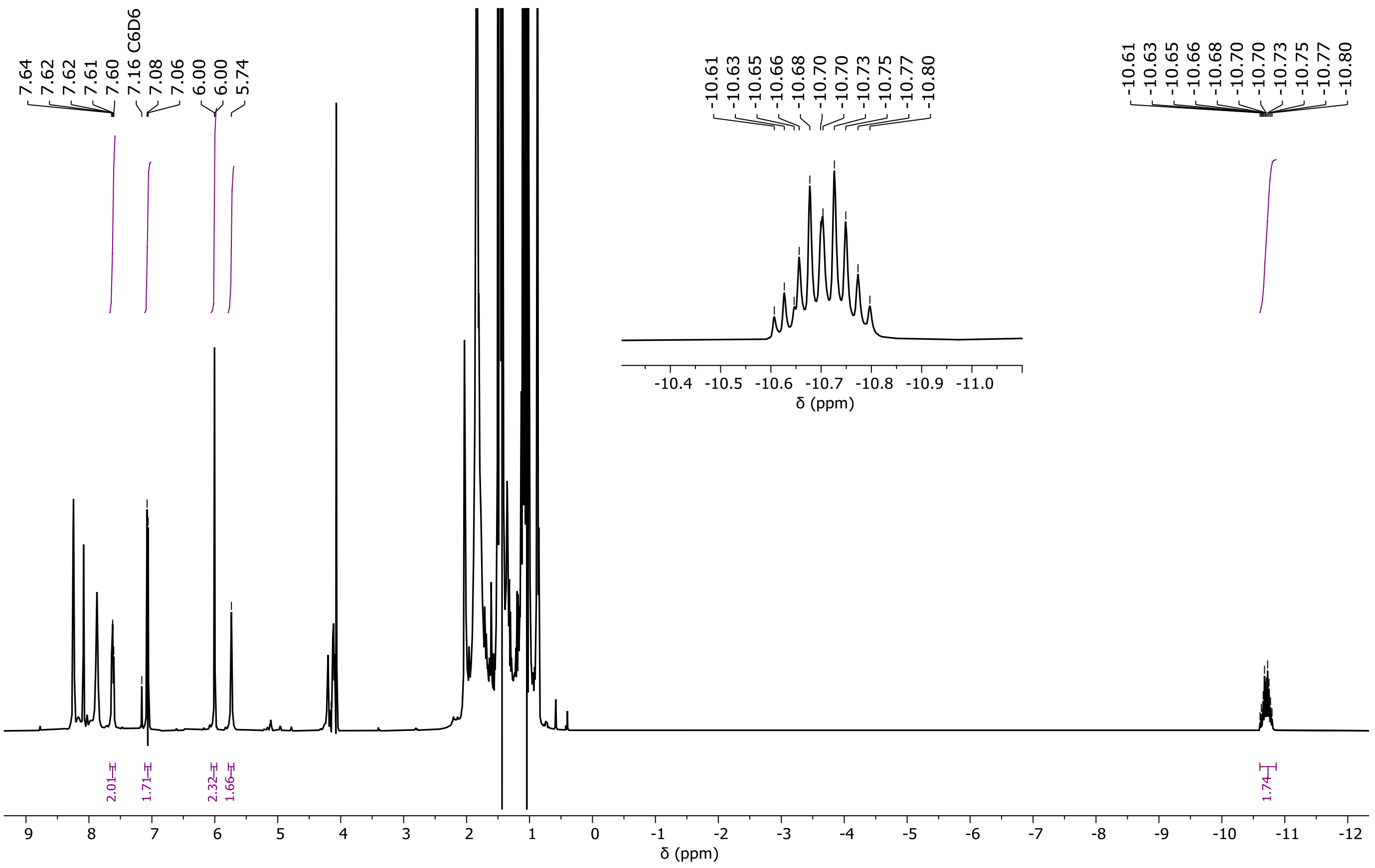

^{2}H-NMR of Ir2 generated using Et_3SiD (Dioxane, C_6D_6 as internal standard):

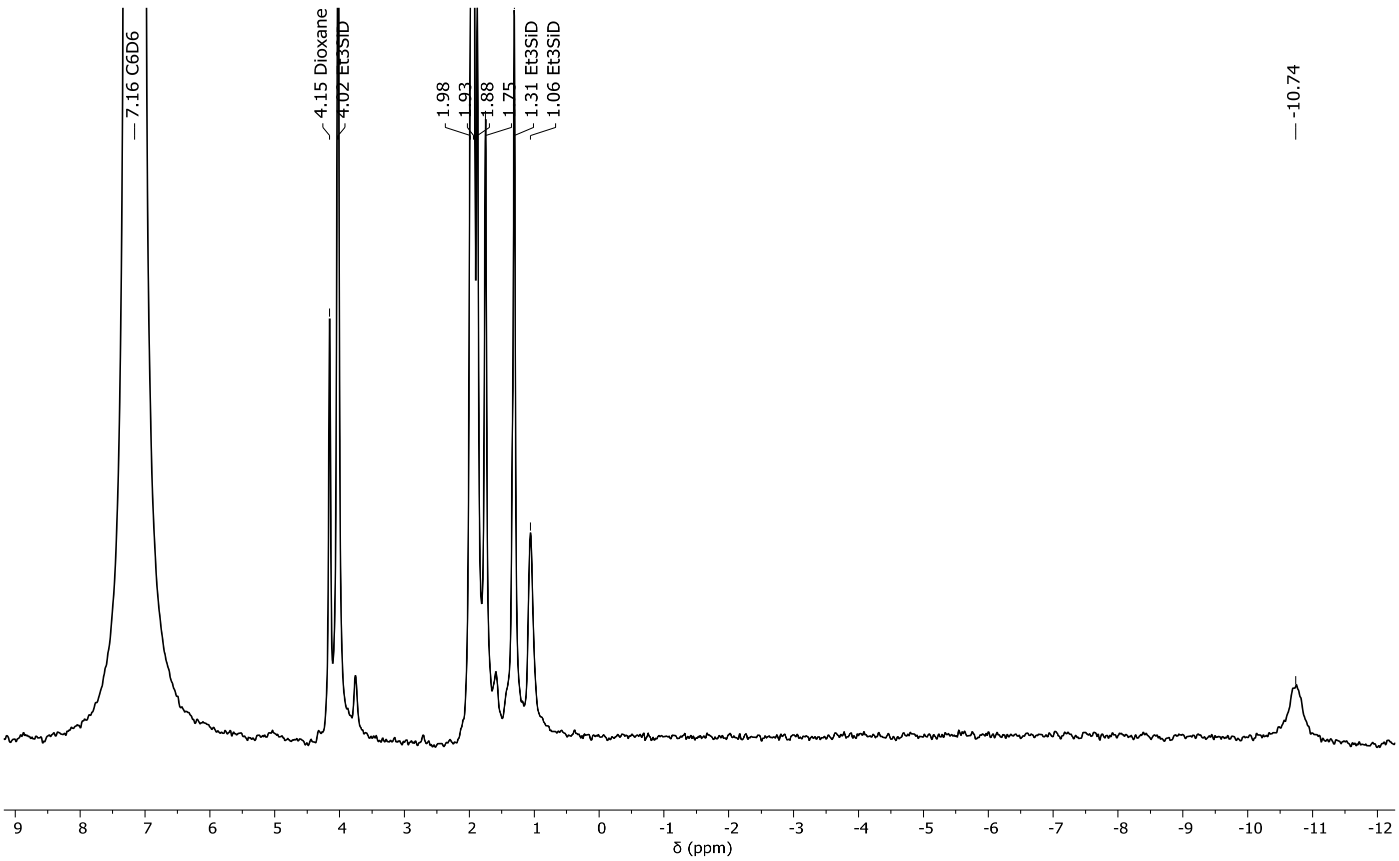

Reaction of Ir1 with $BnMe_2SiH$: Detection of Ir2'

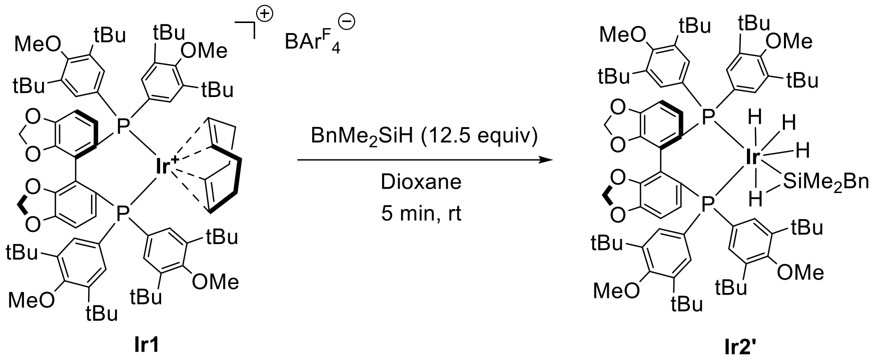

Ir1 (16.1 mg, 0.007 mmol) was dissolved in 1,4-dioxane (0.56 mL). Benzyldimethylsilane (13.6 µL, 0.088 mmol, 12.5 equiv) were added and the mixture was stirred until the solution becomes yellow (5 min approx.). ^{1}H-NMR was measured using a glass capillary with C_6D_6 as internal standard. **^{1}H NMR** (500 MHz, Dioxane and C_6D_6 as internal standard, partial data) δ -10.3 (t, *J* = 13.4 Hz, 4H) ppm. **^{31}P{1H} NMR** (202 MHz, Dioxane and C_6D_6 as internal standard) δ 9.01 (s, 2P) ppm.

Likewise, Iridium hydride **Ir2'** was formed, showing a triplet at -10.27 ppm by ^{1}H-NMR. The same reaction was tried with the bulkier ^{t}BuMe$_2$SiH and ^{i}PrSiH, but no Iridium hydride complexes were detected in these cases. The difference between the chemical shifts of **Ir2** and **Ir2'** further confirms that the silane fragment is near to the Ir-hydrides, analogously to the complexes described by Schley and coworkers, with a ^{1}H-NMR signal (at CD_2Cl_2) at -10.82 ppm (t, *J* = 11.6 Hz).[23,24]

^{1}H-NMR of Ir2' generated using $BnMe_2SiH$ (Dioxane, C_6D_6 as internal standard):

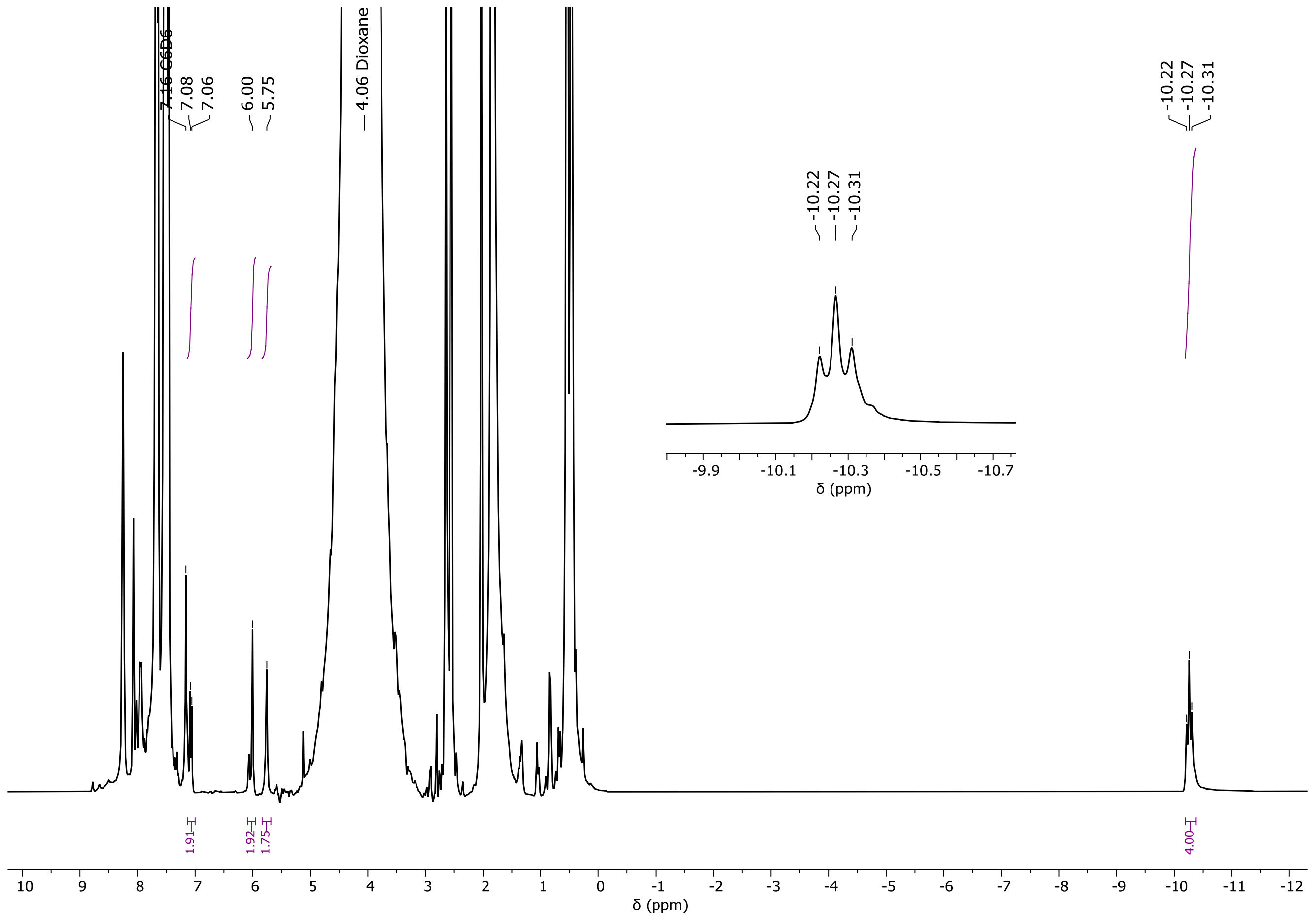

Catalytic competence of Ir2

Substrate **1b** (20 mg, 0.07 mmol) was added to 0.56 mL of the solution containing the in situ generated complex **Ir2** (**Ir1** + 12.5 equiv of Et_3SiH in dioxane), which corresponds to a 10 mol% cat. Full and clean conversion towards alcohol **2b** was observed after 3 hours, confirming the catalytic competence of **Ir2**.

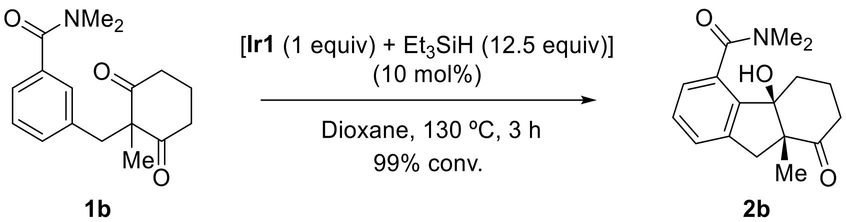

To a solution of substrate **1b** (20 mg, 0.07 mmol) and previously isolated **Ir2** (0.007 mmol, 10 mol% cat) in dioxane (0.56 mL), was added Et_3SiH (14 μL, 1.25 equiv). Formation of alcohol **2b** was observed after 3 hours in 94% conversion by NMR, confirming the catalytic competence of **Ir2**.

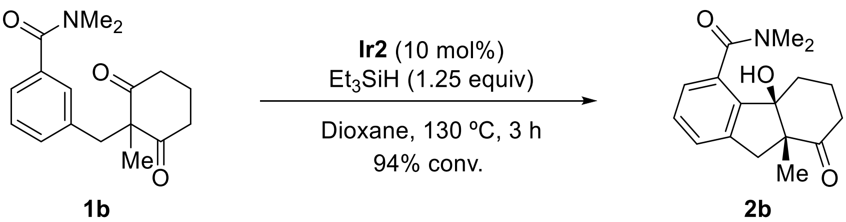

8. Kinetic studies

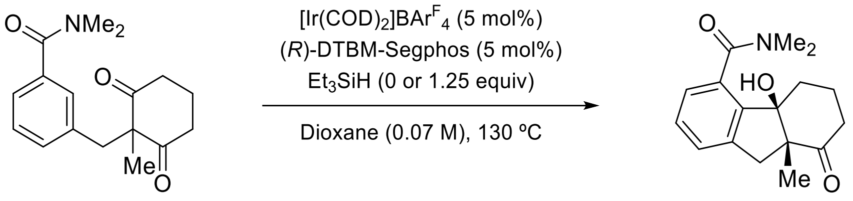

General procedure: In a Schlenk tube equipped with a stirring bar, **1b** (92.0 mg, 0.32 mmol), $Ir(COD)_2BAr^F_4$ (20.2 mg, 0.016 mmol, 5 mol%) and (*R*)-DTBM-Segphos (18.8 mg, 0.016 mmol, 5 mol%) were successively added under argon. Dioxane (4.6 mL, 0.07 M) was then added, followed by triethylsilane (63.7 μL, 0.4 mmol, 1.25 equiv) if corresponds. The reaction mixture was stirred at rt until all components, dissolved and divided in 0.35 mL aliquots using a syringe, that were placed in separated sealed tubes. All these tubes were heated in a pre-heated oil bath at 130 °C. At the times indicated, each reaction was removed from the oil bath, cooled with the help of an ice bath, filtered through Florisil®, and the volatiles were removed under reduced pressure. The crude residues were redissolved in CH_2Cl_2 and analyzed by GS/MS.

Kinetics without Et_3SiH

Carried out following the general procedure for kinetic studies, without adding triethylsilane. Aliquots were stopped every 30 minutes.

**Time (min)**	Average conversion (%)
**0**	0
**30**	0
**60**	0.03
**90**	0.73
**120**	1.56
**150**	2.53
**180**	3.98
**210**	5.46
**240**	6.80

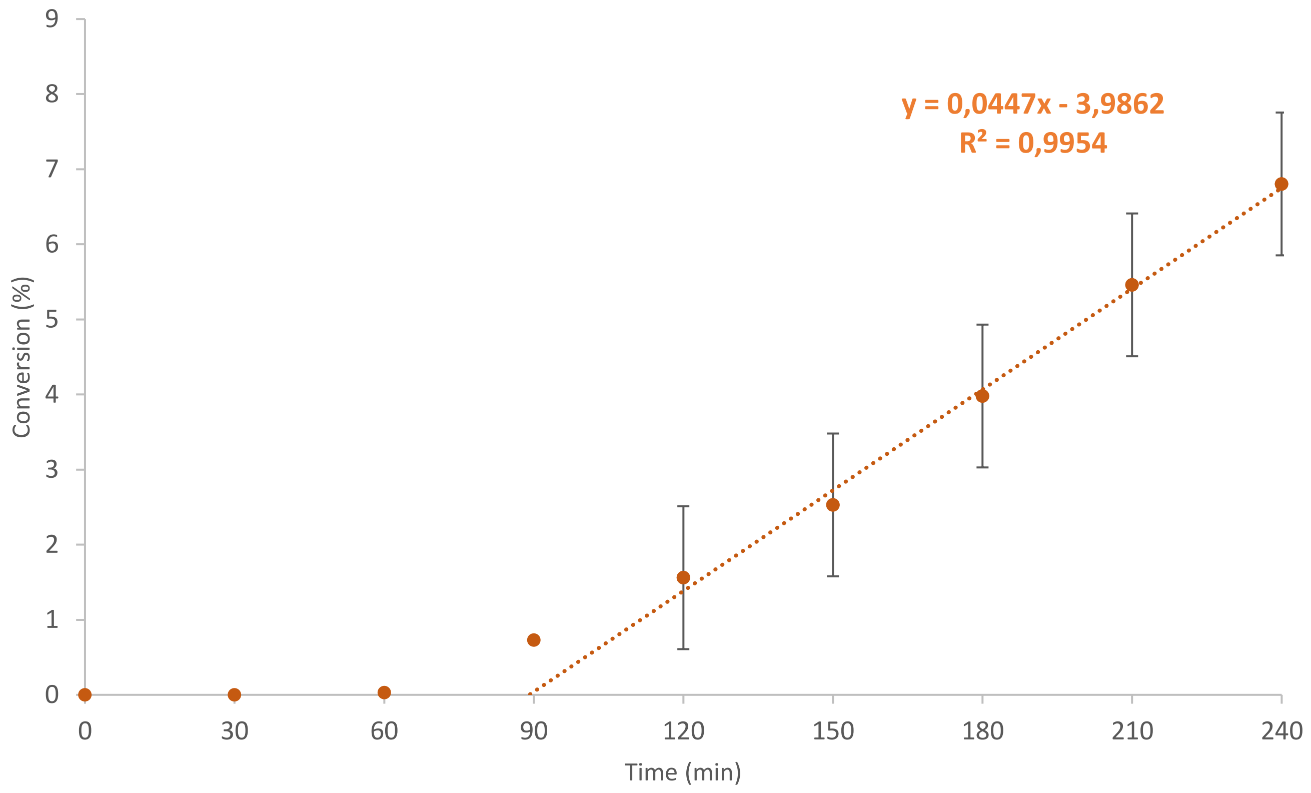

Kinetics using 1.25 equiv of Et_3SiH

Carried out following the general procedure for kinetic studies, adding 1.25 equiv of triethylsilane. Aliquots were stopped every 5 minutes. This experiment was repeated 3 times.

Time (min)	Average conversion (%)
**0**	0.00
**5**	0.00
**10**	0.55
**15**	3.59
**20**	6.13
**25**	9.75
**30**	12.16
**35**	16.52
**40**	18.28
**45**	23.01
**50**	26.10
**55**	30.62
**60**	34.46

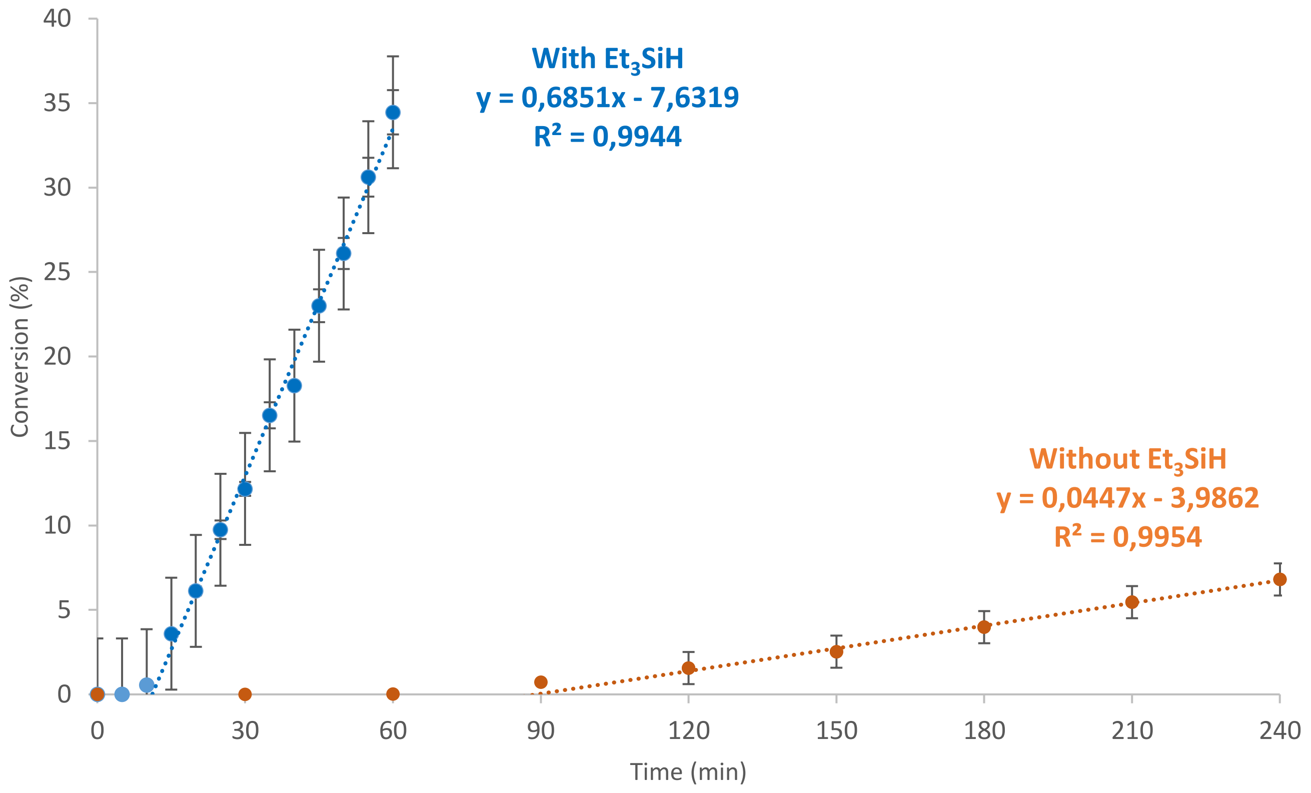

Calculated **induction time** was **11 ± 1 minutes.**

The reaction with Et_3SiH is 0.6851/0.0366 = **15 times faster than without Et_3SiH.**

Kinetics using 1.25 equiv of Et_3SiD

Carried out following the general procedure for kinetic studies, adding 1.25 equiv of deuterated Triethylsilane (Et_3SiD). Aliquots were stopped every 5 minutes. This experiment was repeated 3 times.

Time (min)	Average conversion (%)
0	0.00
5	0.00
10	0.43
15	2.19
20	3.12
25	5.01
30	8.75
35	12.44
40	13.54
45	17.39
50	19.60
55	23.90
60	28.05

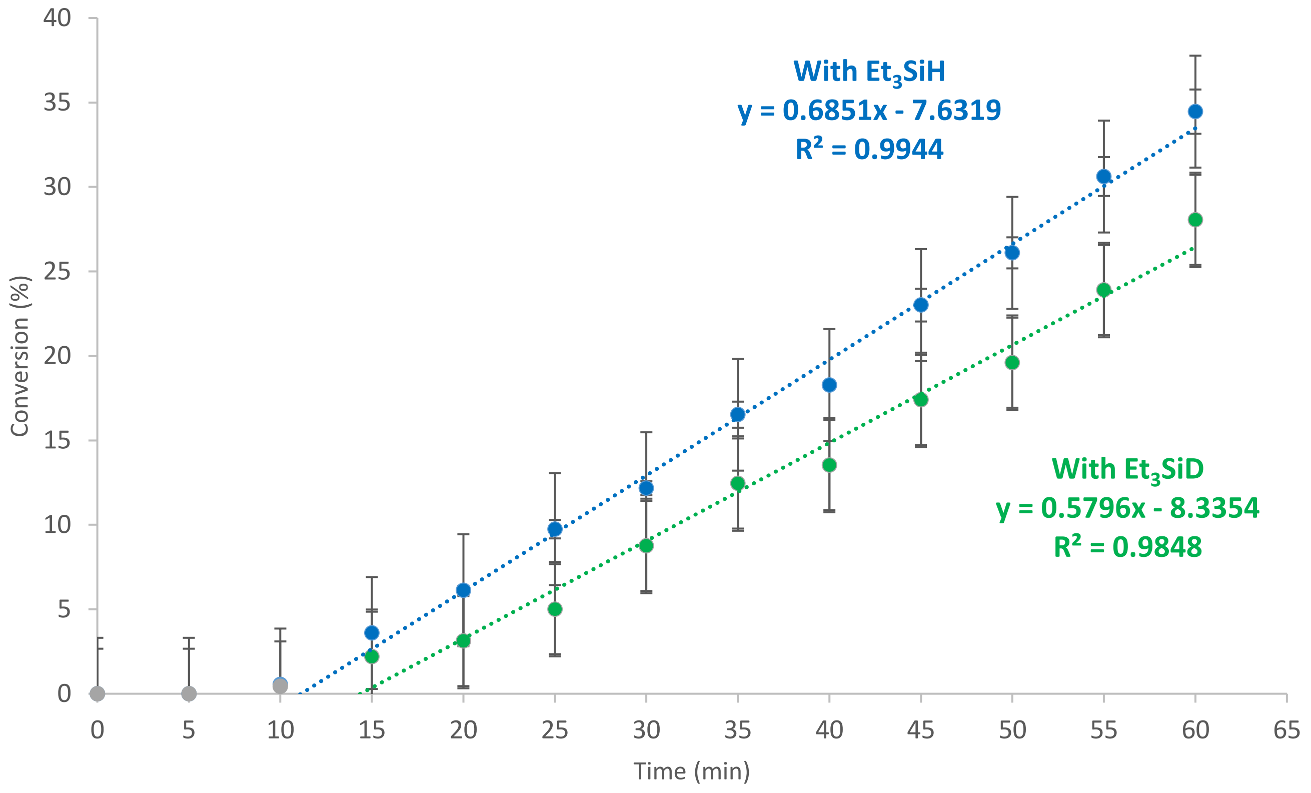

Calculated **induction time** was **14 ± 2 minutes.**

The calculated **Kinetic Isotope Effect** is 0.6851/0.5796 = **1.18 ± 0.06**

9. Single crystal X-ray diffractometry details

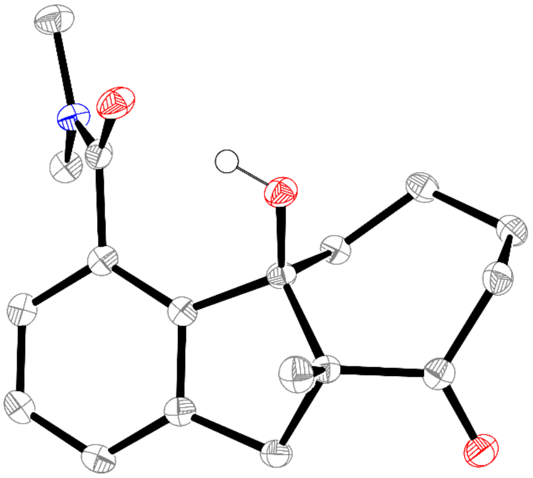

X-Ray structure of 2m (94:6 er) – CCDC: 2504171

Empirical formula	**$C_{36}H_{46}N_2O_6$**	μ/mm^{-1}	**0.705**
Formula weight	**602.75**	F(000)	**648.0**
Temperature/K	**100.15**	Crystal size/mm^3	**0.11 × 0.08 × 0.03**
Crystal system	**orthorhombic**	Radiation	**CuKα (λ = 1.54184)**
Space group	**$P2_12_12_1$**	2Θ range for data collection/°	**8.514 to 140.106**
a/Å	**7.04330(10)**	Index ranges	**-8 ≤ h ≤ 8, -16 ≤ k ≤ 13, -19 ≤ l ≤ 19**
b/Å	**13.55440(10)**	Reflections collected	**16883**
c/Å	**16.1649(2)**	Independent reflections	**2925 [R_{int} = 0.0230, R_{sigma} = 0.0163]**
α/°	**90**	Data/restraints/parameters	**2925/0/203**
β/°	**90**	Goodness-of-fit on F^2	**1.055**
γ/°	**90**	Final R indexes [I>=2σ (I)]	**R_1 = 0.0257, wR_2 = 0.0652**
Volume/Å^3	**1543.23(3)**	Final R indexes [all data]	**R_1 = 0.0270, wR_2 = 0.0659**
Z	**2**	Largest diff. peak/hole / e Å^{-3}	**0.14/-0.15**
ρ_{calc}g/cm^3	**1.297**	Flack parameter	**0.10(6)**

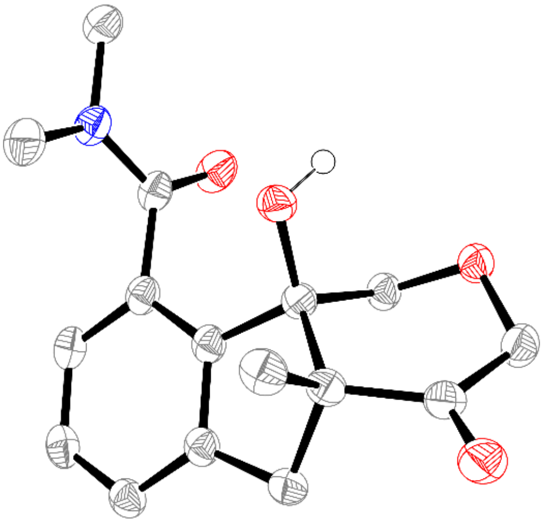

X-Ray structure of 2p (racemic) – CCDC: 2488404

Empirical formula	**$C_{16}H_{19}NO_4$**	μ/mm^{-1}	**0.777**
Formula weight	**289.32**	F(000)	**616.0**
Temperature/K	**100.00(10)**	Crystal size/mm^3	**0.14 × 0.12 × 0.1**
Crystal system	**monoclinic**	Radiation	**Cu Kα (λ = 1.54184)**
Space group	**$P2_1/c$**	2Θ range for data collection/°	**9.96 to 153.74**
a/Å	**8.14860(10)**	Index ranges	**-10 ≤ h ≤ 10, -21 ≤ k ≤ 19, -13 ≤ l ≤ 12**
b/Å	**17.3468(2)**	Reflections collected	**59927**
c/Å	**10.41100(10)**	Independent reflections	**3024 [R_{int} = 0.0458, R_{sigma} = 0.0125]**
α/°	**90**	Data/restraints/parameters	**3024/0/194**
β/°	**96.7900(10)**	Goodness-of-fit on F^2	**1.035**
γ/°	**90**	Final R indexes [I>=2σ (I)]	**R_1 = 0.0383, wR_2 = 0.1028**
Volume/Å^3	**1461.30(3)**	Final R indexes [all data]	**R_1 = 0.0407, wR_2 = 0.1054**
Z	**4**	Largest diff. peak/hole / e Å^{-3}	**0.41/-0.23**
ρ_{calc}g/cm^3	**1.315**		

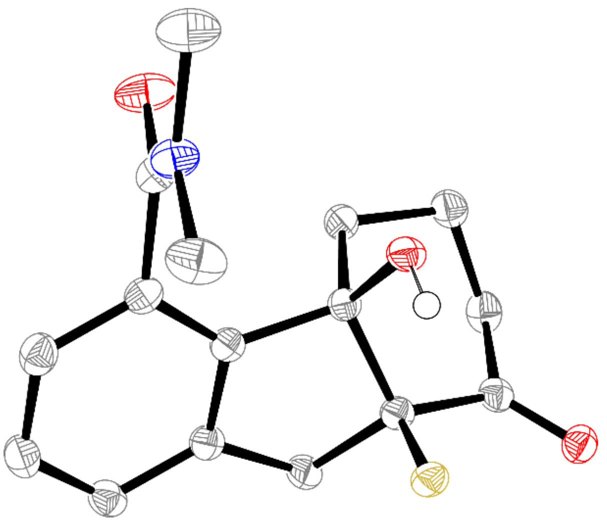

X-Ray structure of 2r (racemic) – CCDC: 2504167

Empirical formula	**$C_{16}H_{18}NO_3F$**	μ/mm^{-1}	**0.842**
Formula weight	**291.31**	F(000)	**616.0**
Temperature/K	**100.15**	Crystal size/mm^3	**0.2 × 0.12 × 0.12**
Crystal system	**monoclinic**	Radiation	**CuKα (λ = 1.54184)**
Space group	**$P2_1/n$**	2Θ range for data collection/°	**9.086 to 144.028**
a/Å	**11.03840(10)**	Index ranges	**-12 ≤ h ≤ 13, -11 ≤ k ≤ 11, -18 ≤ l ≤ 17**
b/Å	**9.19980(10)**	Reflections collected	**28489**
c/Å	**14.59740(10)**	Independent reflections	**2838 [R_{int} = 0.0294, R_{sigma} = 0.0143]**
α/°	**90**	Data/restraints/parameters	**2838/0/193**
β/°	**103.6690(10)**	Goodness-of-fit on F^2	**1.050**
γ/°	**90**	Final R indexes [I>=2σ (I)]	**R_1 = 0.0332, wR_2 = 0.0878**
Volume/Å^3	**1440.40(2)**	Final R indexes [all data]	**R_1 = 0.0354, wR_2 = 0.0898**
Z	**4**	Largest diff. peak/hole / e Å^{-3}	**0.27/-0.24**
ρ_{calc}g/cm^3	**1.343**		

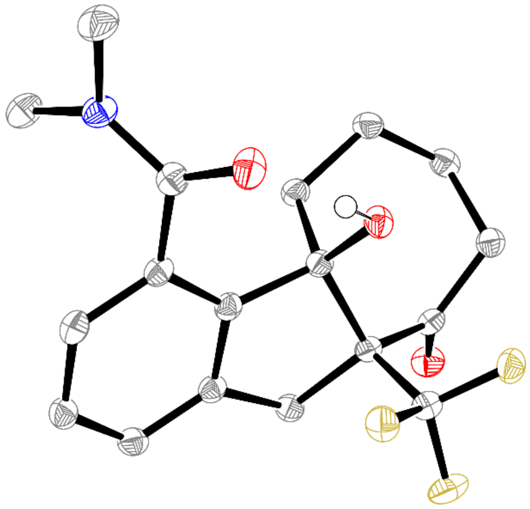

X-Ray structure of 2s (94:6 er) – CCDC: 2504169

Empirical formula	**$C_{36}H_{40}F_6N_2O_6$**	μ/mm^{-1}	**1.051**
Formula weight	**710.70**	F(000)	**744.0**
Temperature/K	**100.15**	Crystal size/mm^3	**0.13 × 0.07 × 0.05**
Crystal system	**orthorhombic**	Radiation	**CuKα (λ = 1.54184)**
Space group	**$P2_12_12_1$**	2Θ range for data collection/°	**8.416 to 144.22**
a/Å	**7.17399(4)**	Index ranges	**$-8 \leq h \leq 8$, $-17 \leq k \leq 17$, $-18 \leq l \leq 19$**
b/Å	**13.82644(8)**	Reflections collected	**60678**
c/Å	**16.15812(10)**	Independent reflections	**3162 [R_{int} = 0.0294, R_{sigma} = 0.0111]**
α/°	**90**	Data/restraints/parameters	**3162/0/229**
β/°	**90**	Goodness-of-fit on F^2	**1.049**
γ/°	**90**	Final R indexes [I>=2σ (I)]	**R_1 = 0.0227, wR_2 = 0.0586**
Volume/Å^3	**1602.736(16)**	Final R indexes [all data]	**R_1 = 0.0237, wR_2 = 0.0592**
Z	**2**	Largest diff. peak/hole / e Å^{-3}	**0.14/-0.18**
ρ_{calc}g/cm^3	**1.473**	Flack parameter	**0.03(2)**

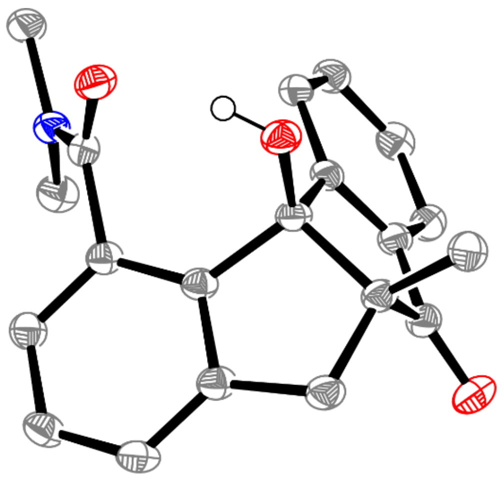

X-Ray structure of 2α (racemic) – CCDC: 2504166

Empirical formula	**$C_{20}H_{19}NO_3$**	μ/mm^{-1}	**0.717**
Formula weight	**321.36**	F(000)	**680.0**
Temperature/K	**100.15**	Crystal size/mm^3	**0.19 × 0.17 × 0.14**
Crystal system	**triclinic**	Radiation	**Cu Kα (λ = 1.54184)**
Space group	**P-1**	2Θ range for data collection/°	**5.22 to 140.144**
a/Å	**9.09430(10)**	Index ranges	**-11 ≤ h ≤ 11, -12 ≤ k ≤ 12, -21 ≤ l ≤ 21**
b/Å	**10.51560(10)**	Reflections collected	**65619**
c/Å	**17.4538(2)**	Independent reflections	**6074 [R_{int} = 0.0210, R_{sigma} = 0.0089]**
α/°	**88.2580(10)**	Data/restraints/parameters	**6074/0/441**
β/°	**75.9830(10)**	Goodness-of-fit on F^2	**1.036**
γ/°	**86.6090(10)**	Final R indexes [I>=2σ (I)]	**R_1 = 0.0348, wR_2 = 0.0894**
Volume/Å^3	**1616.38(3)**	Final R indexes [all data]	**R_1 = 0.0360, wR_2 = 0.0904**
Z	**4**	Largest diff. peak/hole / e Å^{-3}	**0.26/-0.23**
$ρ_{calc}$g/cm^3	**1.321**		

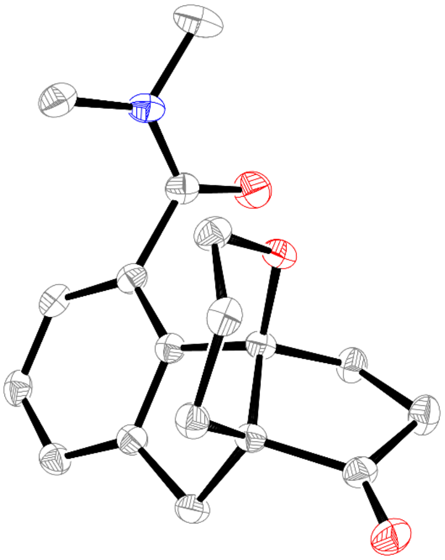

X-Ray structure of 2β' (racemic) – CCDC: 2488405

Empirical formula	**$C_{18}H_{21}NO_3$**	μ/mm^{-1}	**0.714**
Formula weight	**299.36**	F(000)	**1280.0**
Temperature/K	**100.15**	Crystal size/mm^3	**0.07 × 0.05 × 0.03**
Crystal system	**orthorhombic**	Radiation	**CuKα (λ = 1.54184)**
Space group	**Pbca**	2Θ range for data collection/°	**10.29 to 140.146**
a/Å	**10.53590(10)**	Index ranges	**-9 ≤ h ≤ 12, -20 ≤ k ≤ 20, -20 ≤ l ≤ 20**
b/Å	**16.7982(2)**	Reflections collected	**25958**
c/Å	**17.1942(2)**	Independent reflections	**2887 [R_{int} = 0.0216, R_{sigma} = 0.0146]**
α/°	**90**	Data/restraints/parameters	**2887/0/201**
β/°	**90**	Goodness-of-fit on F^2	**1.047**
γ/°	**90**	Final R indexes [I>=2σ (I)]	**R_1 = 0.0348, wR_2 = 0.0903**
Volume/Å^3	**3043.10(6)**	Final R indexes [all data]	**R_1 = 0.0390, wR_2 = 0.0934**
Z	**8**	Largest diff. peak/hole / e Å^{-3}	**0.25/-0.19**
ρ_{calc}g/cm^3	**1.307**		

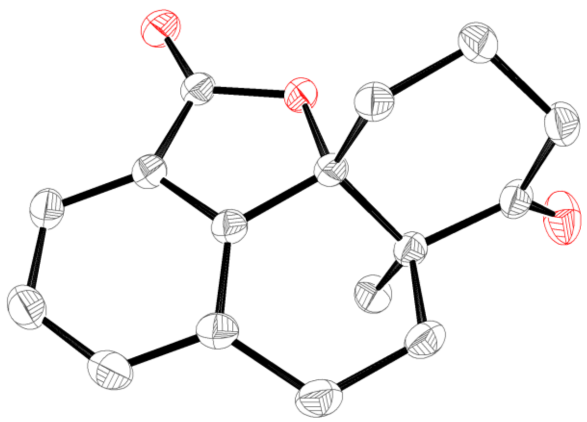

X-Ray structure of 2γ' (racemic) – CCDC: 2503232

Empirical formula	**$C_{32}H_{32}O_6$**	μ/mm^{-1}	**0.765**
Formula weight	**512.57**	F(000)	**544.0**
Temperature/K	**293.15**	Crystal size/mm^3	**0.36 × 0.22 × 0.06**
Crystal system	**monoclinic**	Radiation	**CuKα (λ = 1.54184)**
Space group	**$P2_1/c$**	2Θ range for data collection/°	**7.284 to 145.212**
a/Å	**13.1935(2)**	Index ranges	**-16 ≤ h ≤ 16, -9 ≤ k ≤ 9, -16 ≤ l ≤ 16**
b/Å	**7.53040(10)**	Reflections collected	**4787**
c/Å	**13.5085(2)**	Independent reflections	**4787 [R_{int} = ?, R_{sigma} = 0.0069]**
α/°	**90**	Data/restraints/parameters	**4787/0/174**
β/°	**113.095(2)**	Goodness-of-fit on F^2	**1.086**
γ/°	**90**	Final R indexes [I>=2σ (I)]	**R_1 = 0.0442, wR_2 = 0.1267**
Volume/Å^3	**1234.54(4)**	Final R indexes [all data]	**R_1 = 0.0467, wR_2 = 0.1293**
Z	**2**	Largest diff. peak/hole / e Å^{-3}	**0.19/-0.19**
ρ_{calc}g/cm^3	**1.379**		

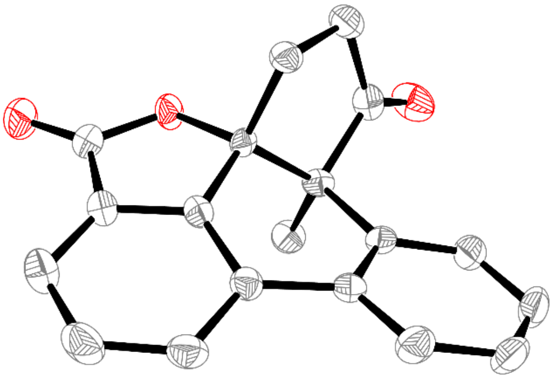

X-Ray structure of 2ε' (76:24 er) – CCDC: 2504174

Empirical formula	$C_{38}H_{28}O_6$	μ/mm^{-1}	**0.752**
Formula weight	**580.02**	F(000)	**607.0**
Temperature/K	**100.15**	Crystal size/mm^3	**0.08 × 0.04 × 0.02**
Crystal system	**monoclinic**	Radiation	**CuKα (λ = 1.54184)**
Space group	$P2_1$	2Θ range for data collection/°	**6.16 to 140.04**
a/Å	**13.5666(2)**	Index ranges	**-16 ≤ h ≤ 14, -8 ≤ k ≤ 8, -17 ≤ l ≤ 17**
b/Å	**7.17470(10)**	Reflections collected	**28898**
c/Å	**14.5526(2)**	Independent reflections	**5234 [R_{int} = 0.0289, R_{sigma} = 0.0210]**
α/°	**90**	Data/restraints/parameters	**5234/70/342**
β/°	**99.5920(10)**	Goodness-of-fit on F^2	**1.107**
γ/°	**90**	Final R indexes [I>=2σ (I)]	**R_1 = 0.0646, wR_2 = 0.1432**
Volume/Å^3	**1396.69(3)**	Final R indexes [all data]	**R_1 = 0.0671, wR_2 = 0.1445**
Z	**2**	Largest diff. peak/hole / e Å^{-3}	**0.30/-0.34**
ρ_{calc}g/cm^3	**1.379**	Flack parameter	**0.14(8)**

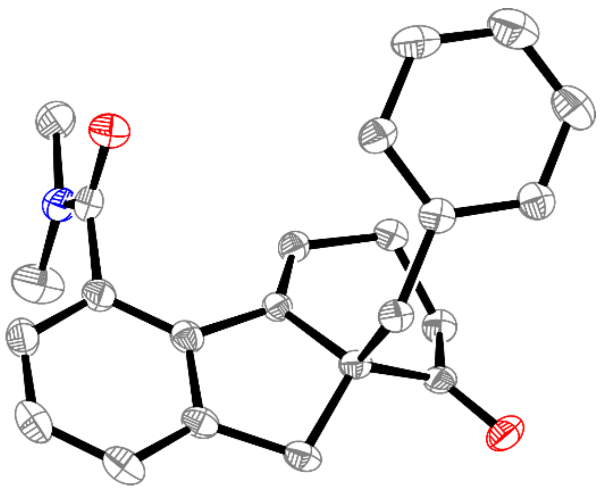

X-Ray structure of 3f (97:3 er) – CCDC: 2504165

Empirical formula	**$C_{23}H_{23}NO_2$**	μ/mm^{-1}	**0.617**
Formula weight	**345.42**	F(000)	**1104.0**
Temperature/K	**100.01(10)**	Crystal size/mm^3	**0.145 × 0.091 × 0.067**
Crystal system	**monoclinic**	Radiation	**Cu Kα (λ = 1.54184)**
Space group	**$P2_1$**	2Θ range for data collection/°	**5.478 to 151.822**
a/Å	**16.13522(8)**	Index ranges	**-15 ≤ h ≤ 18, -8 ≤ k ≤ 8, -31 ≤ l ≤ 31**
b/Å	**6.74884(3)**	Reflections collected	**119787**
c/Å	**25.57125(13)**	Independent reflections	**11184 [R_{int} = 0.0356, R_{sigma} = 0.0132]**
α/°	**90**	Data/restraints/parameters	**11184/12/732**
β/°	**90.4236(4)**	Goodness-of-fit on F^2	**1.117**
γ/°	**90**	Final R indexes [I>=2σ (I)]	**R_1 = 0.0462, wR_2 = 0.1371**
Volume/Å^3	**2784.48α**	Final R indexes [all data]	**R_1 = 0.0469, wR_2 = 0.1377**
Z	**6**	Largest diff. peak/hole / e Å^{-3}	**0.62/-0.26**
ρ_{calc}g/cm^3	**1.236**	**Flack parameter**	**0.01(4)**

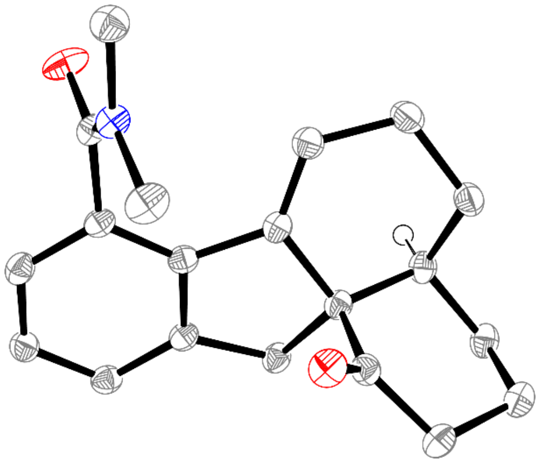

X-Ray structure of 5a (98:2 er) – CCDC: 2504168

Empirical formula	**$C_{40}H_{46}N_2O_4$**	μ/mm^{-1}	**0.643**
Formula weight	**618.79**	F(000)	**332.0**
Temperature/K	**100.15**	Crystal size/mm^3	**0.13 × 0.1 × 0.08**
Crystal system	**monoclinic**	Radiation	**CuKα (λ = 1.54184)**
Space group	**$P2_1$**	2Θ range for data collection/°	**9.976 to 144.196**
a/Å	**8.13940(10)**	Index ranges	**-10 ≤ h ≤ 9, -13 ≤ k ≤ 13, -11 ≤ l ≤ 11**
b/Å	**11.18140(10)**	Reflections collected	**13833**
c/Å	**9.14760(10)**	Independent reflections	**3140 [R_{int} = 0.0292, R_{sigma} = 0.0244]**
α/°	**90**	Data/restraints/parameters	**3140/1/210**
β/°	**104.2480(10)**	Goodness-of-fit on F^2	**1.019**
γ/°	**90**	Final R indexes [I>=2σ (I)]	**R_1 = 0.0298, wR_2 = 0.0753**
Volume/Å^3	**806.913(15)**	Final R indexes [all data]	**R_1 = 0.0307, wR_2 = 0.0763**
Z	**1**	Largest diff. peak/hole / e Å^{-3}	**0.16/-0.18**
ρ_{calc}g/cm^3	**1.273**	Flack parameter	**0.10(11)**

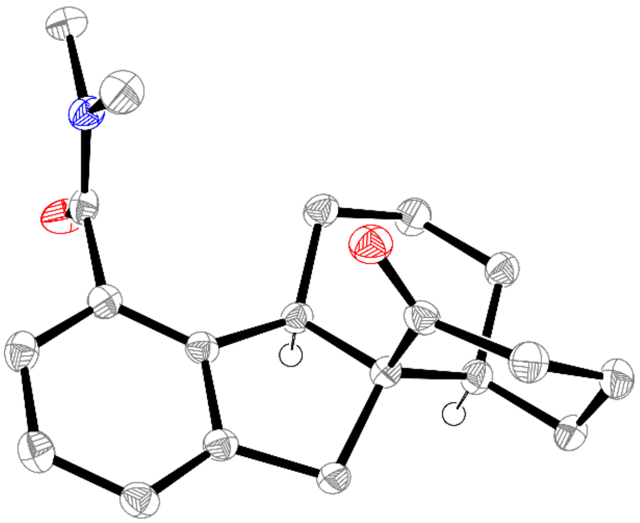

X-Ray structure of 5a' (98:2 er) – CCDC: 2504170

Empirical formula	**$C_{40}H_{50}N_2O_4$**	μ/mm^{-1}	**0.634**
Formula weight	**622.82**	F(000)	**336.0**
Temperature/K	**100.15**	Crystal size/mm^3	**0.19 × 0.1 × 0.08**
Crystal system	**monoclinic**	Radiation	**CuKα (λ = 1.54184)**
Space group	**$P2_1$**	2Θ range for data collection/°	**9.8 to 140.11**
a/Å	**8.13660(10)**	Index ranges	**-9 ≤ h ≤ 9, -13 ≤ k ≤ 10, -11 ≤ l ≤ 11**
b/Å	**11.15740(10)**	Reflections collected	**17840**
c/Å	**9.32340(10)**	Independent reflections	**2767 [R_{int} = 0.0304, R_{sigma} = 0.0246]**
α/°	**90**	Data/restraints/parameters	**2767/1/210**
β/°	**104.5370(10)**	Goodness-of-fit on F^2	**1.035**
γ/°	**90**	Final R indexes [I>=2σ (I)]	**R_1 = 0.0293, wR_2 = 0.0734**
Volume/Å^3	**819.312(16)**	Final R indexes [all data]	**R_1 = 0.0303, wR_2 = 0.0746**
Z	**1**	Largest diff. peak/hole / e Å^{-3}	**0.16/-0.17**
ρ_{calc}g/cm^3	**1.262**	**Flack parameter**	**-0.07(13)**

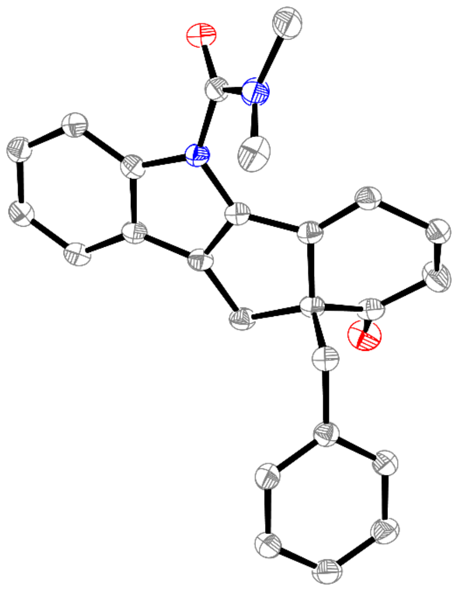

X-Ray structure of 8c (racemic) – CCDC: 2488406

Empirical formula	**$C_{50}H_{48}N_4O_4$**	μ/mm^{-1}	**0.667**
Formula weight	**768.92**	F(000)	**816.0**
Temperature/K	**100.15**	Crystal size/mm^3	**0.17 × 0.13 × 0.07**
Crystal system	**monoclinic**	Radiation	**CuKα (λ = 1.54184)**
Space group	**$P2_1/n$**	2Θ range for data collection/°	**9.824 to 144.226**
a/Å	**11.15910(10)**	Index ranges	**-13 ≤ h ≤ 13, -17 ≤ k ≤ 19, -13 ≤ l ≤ 13**
b/Å	**15.74500(10)**	Reflections collected	**44304**
c/Å	**11.28480(10)**	Independent reflections	**3787 [R_{int} = 0.0292, R_{sigma} = 0.0135]**
α/°	**90**	Data/restraints/parameters	**3787/0/264**
β/°	**103.4720(10)**	Goodness-of-fit on F^2	**1.080**
γ/°	**90**	Final R indexes [I>=2σ (I)]	**R_1 = 0.0346, wR_2 = 0.0887**
Volume/Å^3	**1928.18(3)**	Final R indexes [all data]	**R_1 = 0.0370, wR_2 = 0.0903**
Z	**2**	Largest diff. peak/hole / e Å^{-3}	**0.24/-0.24**
ρ_{calc}g/cm^3	**1.324**		

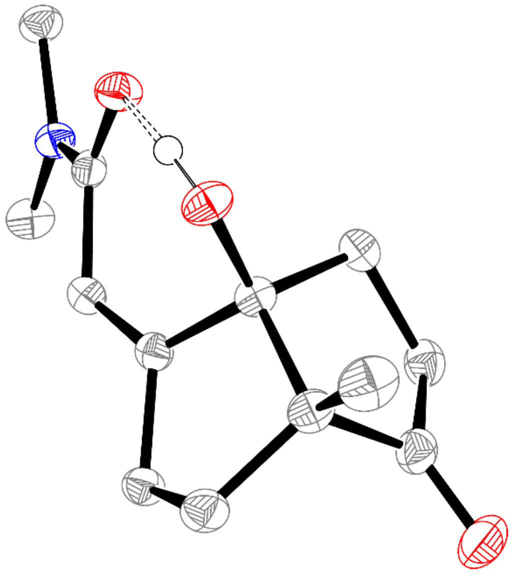

X-Ray structure of 10a (racemic) – CCDC: 2531879

Empirical formula	**$C_{26}H_{38}N_2O_6$**	μ/mm^{-1}	**0.742**
Formula weight	**474.58**	F(000)	**512.0**
Temperature/K	**100.15**	Crystal size/mm^3	**0.29 × 0.13 × 0.04**
Crystal system	**monoclinic**	Radiation	**CuKα (λ = 1.54184)**
Space group	**$P2_1/n$**	2Θ range for data collection/°	**8.448 to 140.11**
a/Å	**12.13710(10)**	Index ranges	**-14 ≤ h ≤ 14, -7 ≤ k ≤ 7, -19 ≤ l ≤ 20**
b/Å	**6.01400(10)**	Reflections collected	**22566**
c/Å	**16.8958(2)**	Independent reflections	**2313 [R_{int} = 0.0289, R_{sigma} = 0.0137]**
α/°	**90**	Data/restraints/parameters	**2313/0/158**
β/°	**97.8670(10)**	Goodness-of-fit on F^2	**1.066**
γ/°	**90**	Final R indexes [I>=2σ (I)]	**R_1 = 0.0367, wR_2 = 0.0975**
Volume/Å^3	**1221.66(3)**	Final R indexes [all data]	**R_1 = 0.0397, wR_2 = 0.1001**
Z	**2**	Largest diff. peak/hole / e Å^{-3}	**0.31/-0.22**
ρ_{calc}g/cm^3	**1.290**		

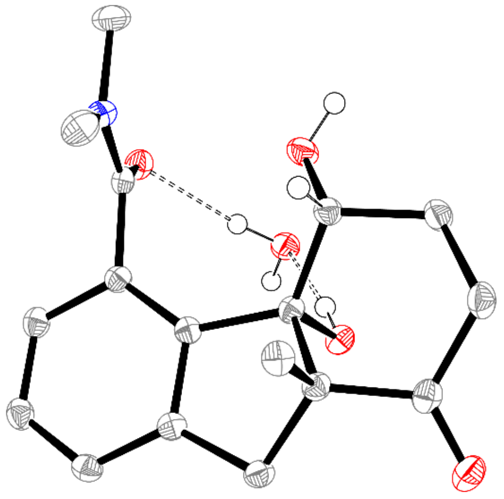

X-Ray structure of 13b (racemic) – CCDC: 2488407

Empirical formula	$\mathbf{C_{34}H_{46}N_2O_{10}}$	μ/mm^{-1}	**0.819**
Formula weight	**642.73**	F(000)	**688.0**
Temperature/K	**100.00(10)**	Crystal size/mm^3	**0.2 × 0.04 × 0.03**
Crystal system	**monoclinic**	Radiation	**Cu Kα (λ = 1.54184)**
Space group	$\mathbf{P2_1/c}$	2Θ range for data collection/°	**6.634 to 151.462**
a/Å	**13.50804(8)**	Index ranges	**-16 ≤ h ≤ 16, -17 ≤ k ≤ 16, -10 ≤ l ≤ 10**
b/Å	**13.86753(8)**	Reflections collected	**86541**
c/Å	**8.55118(4)**	Independent reflections	**3260 [R_{int} = 0.0434, R_{sigma} = 0.0134]**
α/°	**90**	Data/restraints/parameters	**3260/0/216**
β/°	**99.4857(5)**	Goodness-of-fit on F^2	**1.056**
γ/°	**90**	Final R indexes [I>=2σ (I)]	**R_1 = 0.0381, wR_2 = 0.1011**
Volume/Å^3	**1579.931(15)**	Final R indexes [all data]	**R_1 = 0.0407, wR_2 = 0.1031**
Z	**2**	Largest diff. peak/hole / e Å^{-3}	**0.35/-0.34**
$\rho_{calc}g/cm^3$	**1.351**		

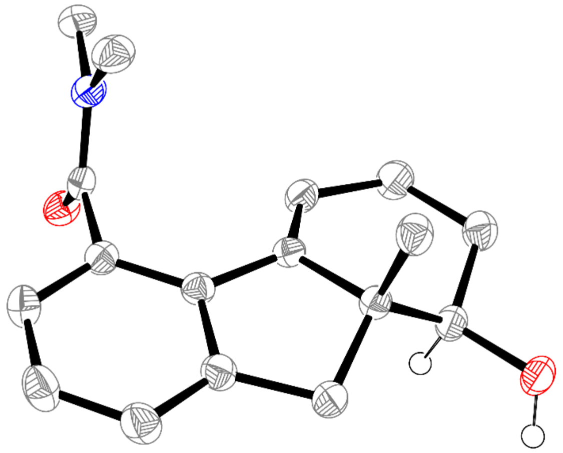

X-Ray structure of 14b (racemic) – CCDC: 2488402

Empirical formula	**$C_{34}H_{42}N_2O_4$**	μ/mm^{-1}	**0.639**
Formula weight	**542.69**	F(000)	**584.0**
Temperature/K	**100.15**	Crystal size/mm^3	**0.08 × 0.07 × 0.06**
Crystal system	**triclinic**	Radiation	**CuKα (λ = 1.54184)**
Space group	**P-1**	2Θ range for data collection/°	**5.338 to 140.138**
a/Å	**9.78100(10)**	Index ranges	**-11 ≤ h ≤ 11, -12 ≤ k ≤ 12, -20 ≤ l ≤ 20**
b/Å	**10.2165(2)**	Reflections collected	**74859**
c/Å	**16.5845(2)**	Independent reflections	**5433 [R_{int} = 0.0279, R_{sigma} = 0.0138]**
α/°	**91.1320(10)**	Data/restraints/parameters	**5433/0/369**
β/°	**92.1460(10)**	Goodness-of-fit on F^2	**1.056**
γ/°	**118.5620(10)**	Final R indexes [I>=2σ (I)]	**R_1 = 0.0353, wR_2 = 0.0876**
Volume/Å^3	**1453.20(4)**	Final R indexes [all data]	**R_1 = 0.0407, wR_2 = 0.0910**
Z	**2**	Largest diff. peak/hole / e Å^{-3}	**0.24/-0.18**
ρ_{calc}g/cm^3	**1.240**		

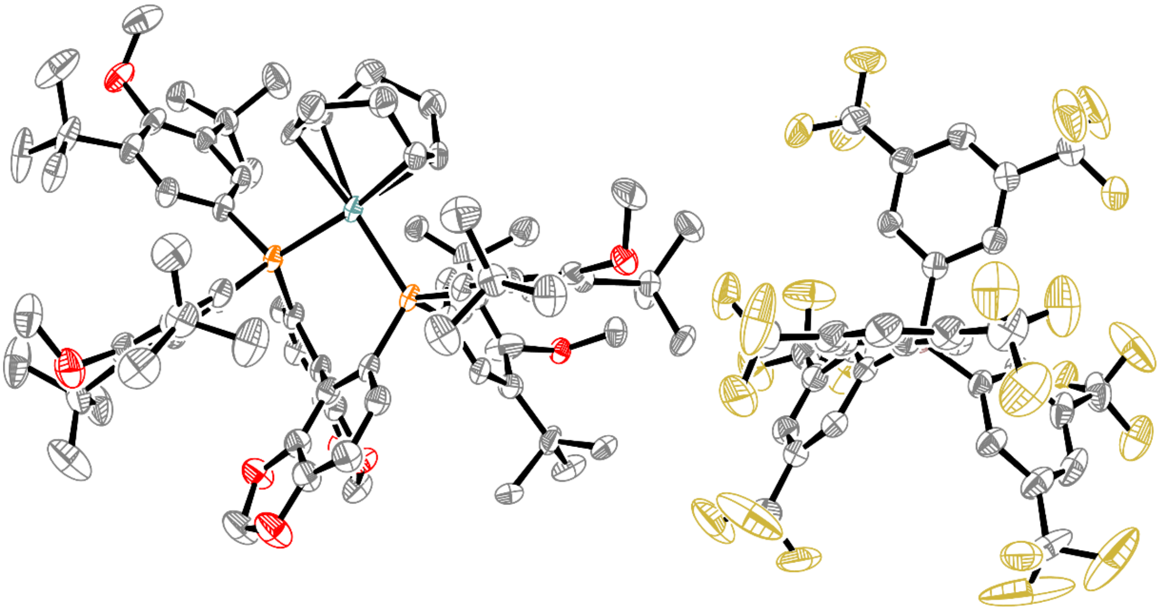

X-Ray structure of Ir1 – CCDC: 2513813

Empirical formula	**$C_{228}H_{248}B_2F_{47.97}Ir_2O_{16}P_4$**	μ/mm^{-1}	**3.468**
Formula weight	**4685.58**	F(000)	**4799.0**
Temperature/K	**100.15**	Crystal size/mm^3	**0.16 × 0.11 × 0.06**
Crystal system	**orthorhombic**	Radiation	**CuKα (λ = 1.54184)**
Space group	**$P2_12_12_1$**	2Θ range for data collection/°	**4.53 to 144.256**
a/Å	**13.82684(4)**	Index ranges	**-17 ≤ h ≤ 16, -30 ≤ k ≤ 30, -39 ≤ l ≤ 40**
b/Å	**24.42536(7)**	Reflections collected	**434640**
c/Å	**32.41474(8)**	Independent reflections	**21556 [R_{int} = 0.0521, R_{sigma} = 0.0187]**
α/°	**90**	Data/restraints/parameters	**21556/88/1535**
β/°	**90**	Goodness-of-fit on F^2	**1.034**
γ/°	**90**	Final R indexes [I>=2σ (I)]	**R_1 = 0.0268, wR_2 = 0.0679**
Volume/Å^3	**10947.28(5)**	Final R indexes [all data]	**R_1 = 0.0276, wR_2 = 0.0684**
Z	**2**	Largest diff. peak/hole / e Å^{-3}	**0.93/-0.69**
ρ_{calc}g/cm^3	**1.421**	**Flack parameter**	**-0.0298(11)**

10. Computational studies

All DFT calculations were carried out with the Gaussian 16 software package.[22] Geometry optimizations for all complexes were performed using the hybrid B3LYP functional, [23] together with the standard 6-31G(d) basis set for C, H, O, P, and N atoms, and the LANL2DZ basis set for Ir.[24] Harmonic frequency analyses at the same level of theory were used to verify the nature of the stationary points (minima, no imaginary frequencies; or transition states, one imaginary frequency) and to compute zero-point energy (ZPE). Gibbs free energies (G) were determined using the harmonic oscillator/rigid rotor approximations. The connectivity between each transition state and its corresponding minima was confirmed by Intrinsic Reaction Coordinate (IRC) calculations. To refine the electronic energies, single-point calculations were subsequently performed employing the M06 hybrid functional developed by Zhao and Truhlar,[25] combined with the 6-311++G(d,p) basis set for C, H, O, P, and N, and the Stuttgart–Dresden (SDD) ECP for Ir.[26] Solvation effects were included using the self-consistent reaction field (SCRF) approach with the SMD solvation model,[27] specifying dioxane as the solvent (ε = 2.2099), to obtain solvation-corrected relative free energies. The topological analyses of the electron density (AIM) were conducted using the Multiwfn software package,[28] based on the wavefunctions obtained from the Gaussian computations. Conformational sampling was performed using the CREST 3.0.2 program.[29]

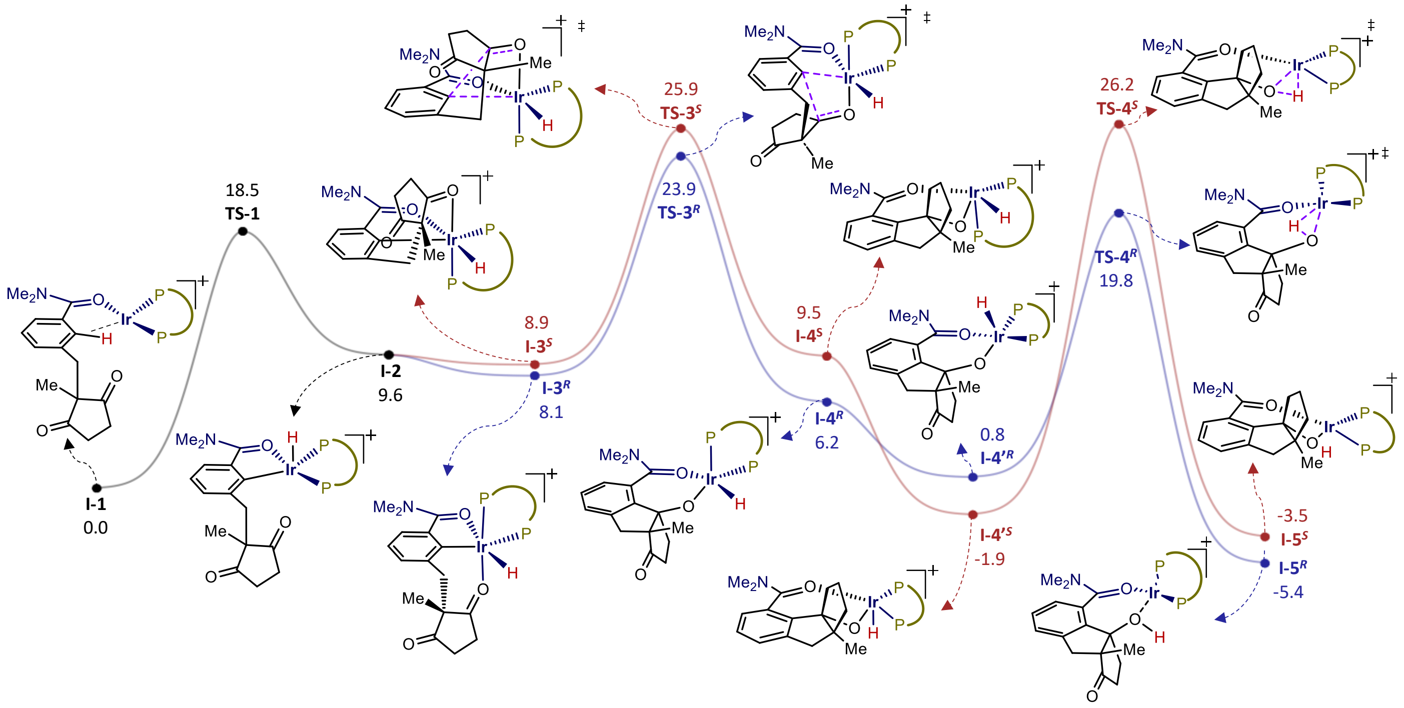

Figure S15: DFT profile (carbometallation/O-H reductive elimination pathway)

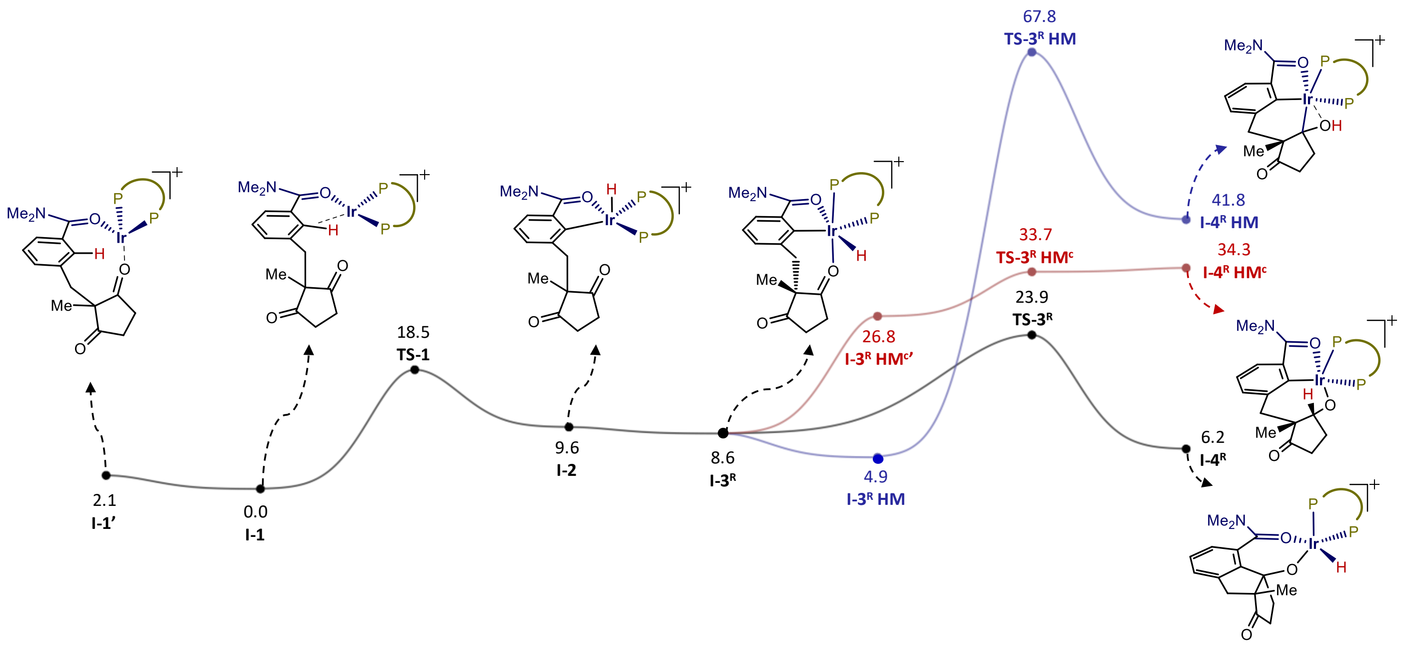

Figure S16: DFT profile comparing carbometallation (via TS-3^{R}) with *endo* and *exo* hydrometallation pathways (via TS-3^{R}HM and TS-3^{R}HM^{c})

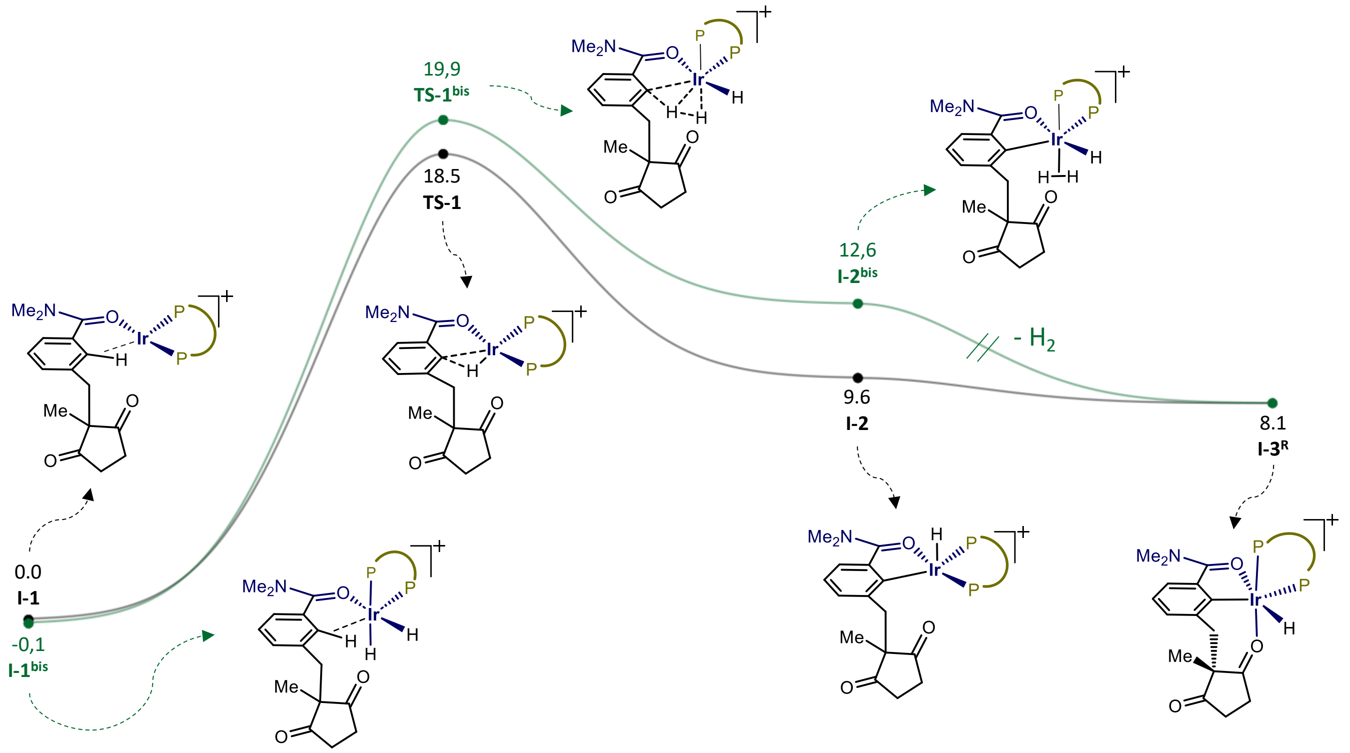

Figure S17: DFT profile comparing oxidative addition from Ir(I)-Segphos and Ir(III)-Segphos bishydride

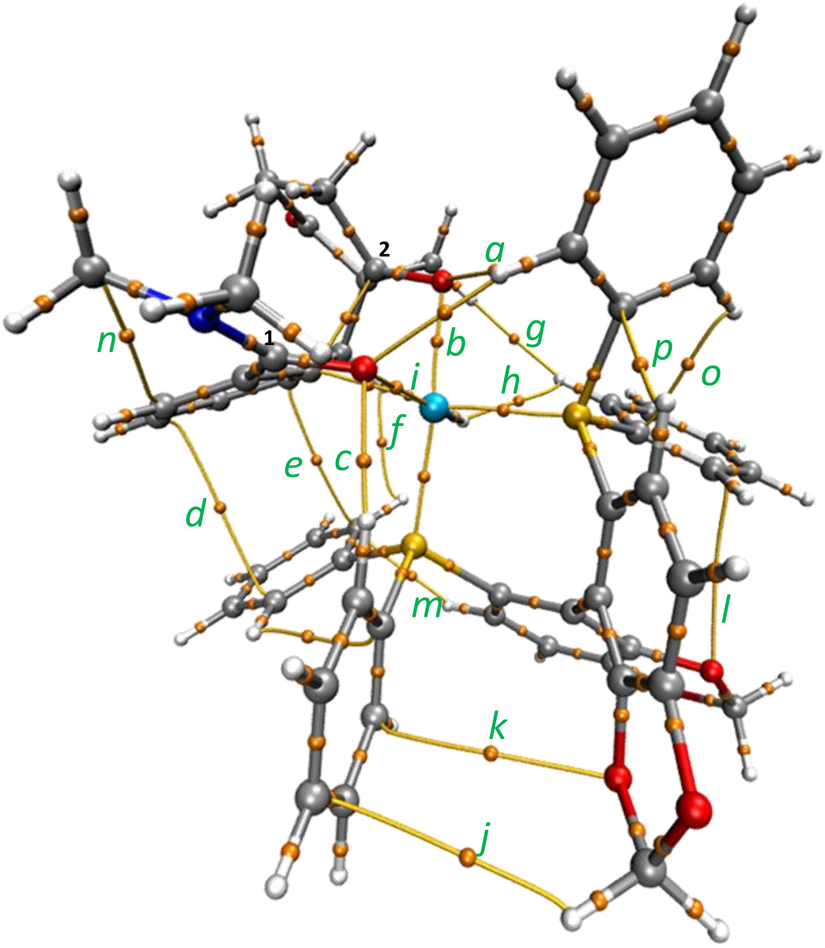

	TS-3^R		
	Interaction	d (Å)	ρ x10^{-2}
1	*a)* $C_{(2)}$=O···C_{Ar}	3.08	0.77
2	*b)* $C_{(1)}$=O···H–C_{Ar}	2.27	1.29
3	*c)* $C_{(1)}$=O···H–C_{Ar}	2.17	1.66
4	*d)* C_{Ar}···C_{Ar}	3.80	0.32
5	*e)* C_{Ar}···C_{Ar}	3.29	0.66
6	*f)* C–H···H–C_{Ar}	2.31	0.68
7	*g)* C–H···H–C_{Ar}	2.48	0.35
8	*h)* Ir–H··· H–C_{Ar}	2.17	1.03
9	*i)* Ir–H··· H–C	2.34	0.85
10	*j)* C–H·· C_{Ar}	3.35	0.27
11	*k)* O··· C_{Ar}	3.62	0.33
12	*l)* O··· C_{Ar}	3.35	0.52
13	*m)* C–H···C_{Ar}	2.37	1.43
14	*n)* C–H··· C_{Ar}	2.51	1.25
15	*o)* C–H···C_{Ar}	2.56	1.18
16	*p)* C–H···C_{Ar}	2.53	1.23

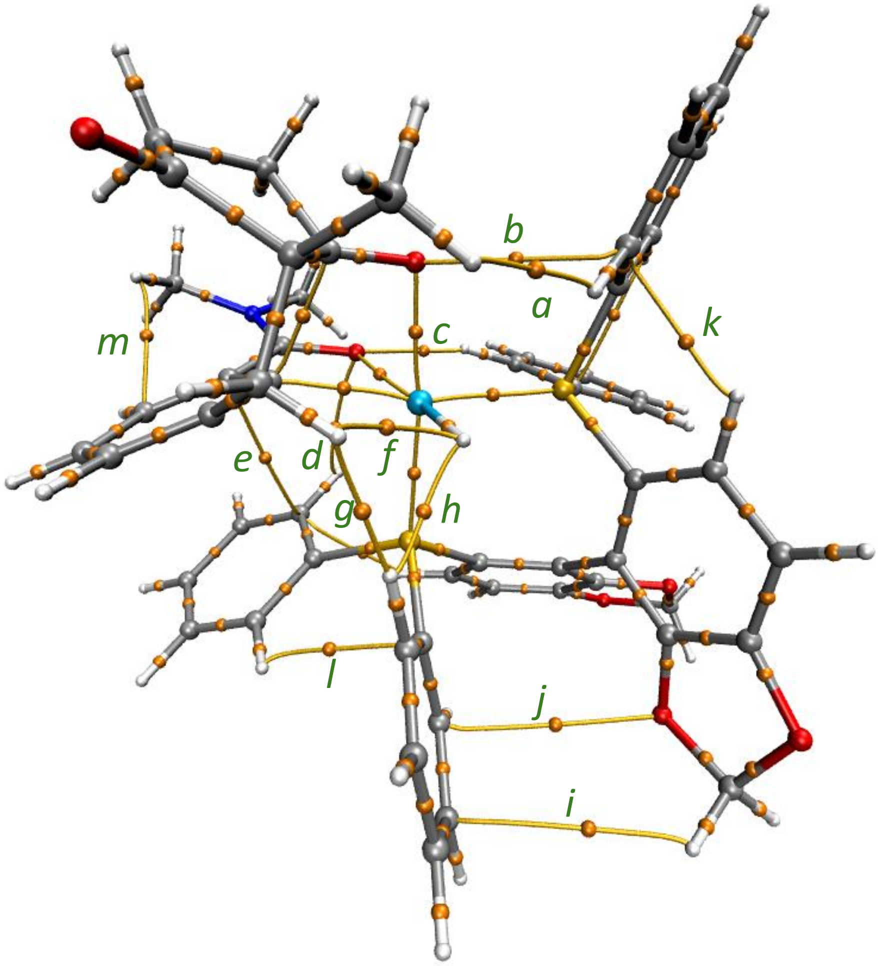

TS-3^{S}

	Interaction	d (Å)	ρ x10^{-2}
1	*a)* C–H···H–C_{Ar}	2.19	0.71
2	*b)* $C_{(2)}$=O···C_{Ar}	2.93	1.00
3	*c)* $C_{(1)}$=O···H–C_{Ar}	2.26	1.27
4	*d)* $C_{(1)}$=O···H–C_{Ar}	0.93	0.93
5	*e)* C_{Ar}···C_{Ar}	0.72	0.71
6	*f)* Ir–H··· H–C	2.33	0.87
7	*g)* C–H···H–C_{Ar}	2.04	0.92
8	*h)* Ir–H··· H–C_{Ar}	2.14	1.12
9	*i)* C–H·· C_{Ar}	3.36	0.23
10	*j)* O·· C_{Ar}	3.46	0.20
11	*k)* C–H··· C_{Ar}	2.46	1.24
12	*l)* C–H··· C_{Ar}	2.54	1.19
13	*m)* C–H··· C_{Ar}	2.52	1.23

Figure S18: Key interatomic contacts at TS-3^{R} and TS-3^{S}, which correspond to non-covalent interactions (NCI's) for which bond paths and bond critical points were found by AIM formalism. The type of each interatomic interaction and the distance (Å) are tabulated.

Conformational sampling of TS-3^R and TS-3^S

Conformational sampling was performed using the CREST 3.0.2 program. 9 different conformers for **TS-3^R** and 6 for **TS-3^S**.

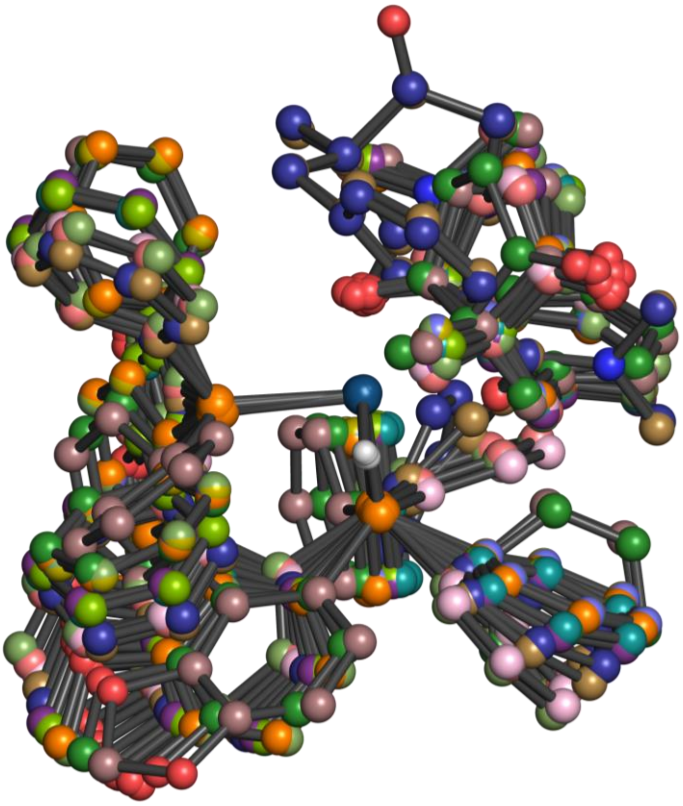

Figure S19: Overlapped structures of the analyzed conformations of TS-3^R

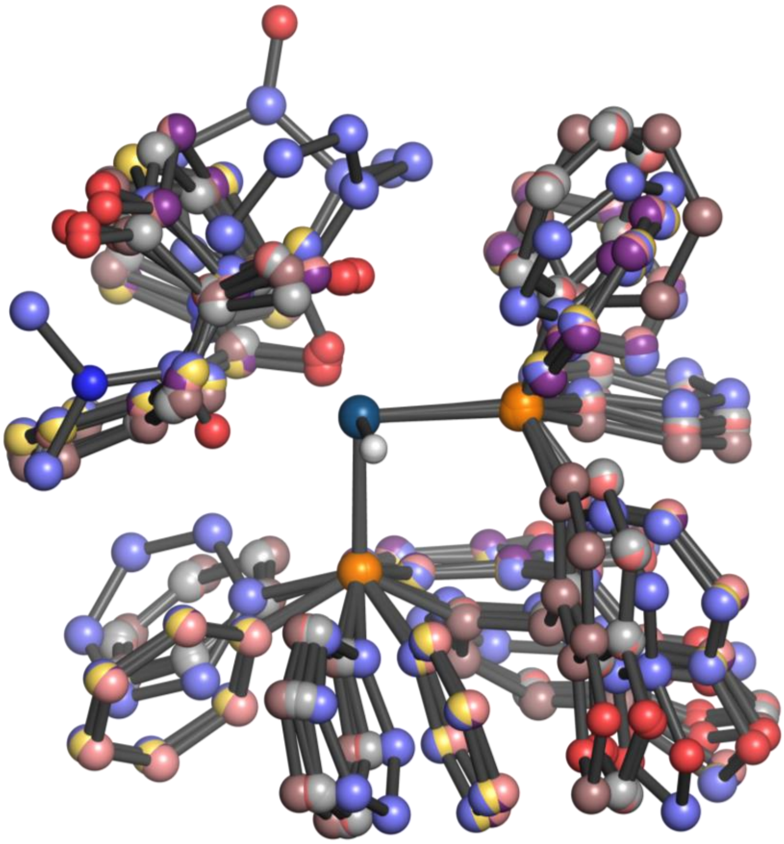

Figure S20: Overlapped structures of the analyzed conformations of TS-3^S

TS-3^{R}-1

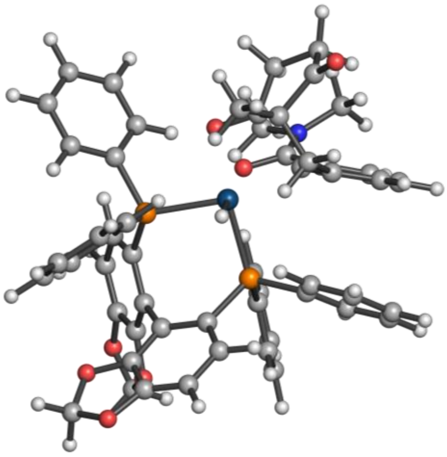

$\Delta\Delta G$= 23.9 kcal/mol

TS-3^{R}-2

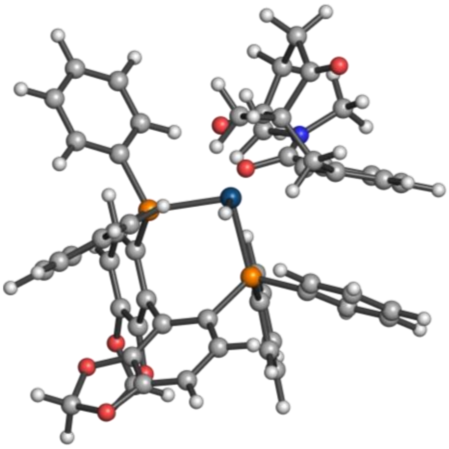

$\Delta\Delta G$= 25.8 kcal/mol

TS-3^{R}-3

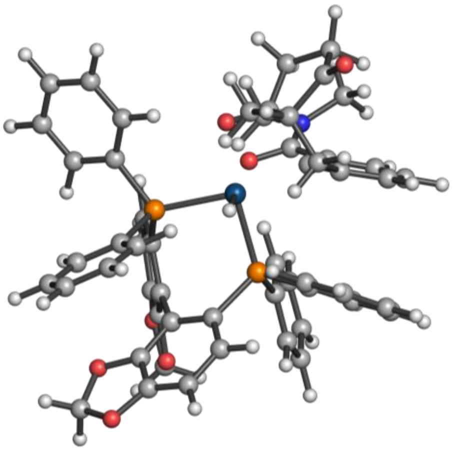

$\Delta\Delta G$= 26.3 kcal/mol

TS-3^{R}-4

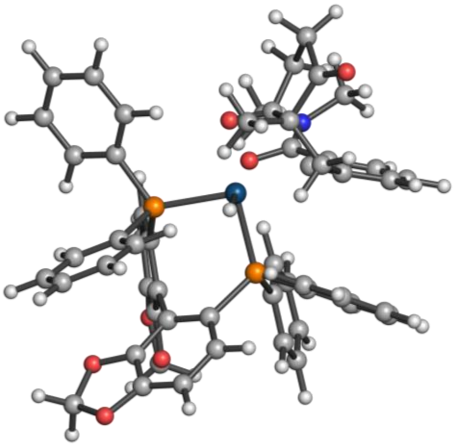

$\Delta\Delta G$= 28.4 kcal/mol

TS-3^{R}-5

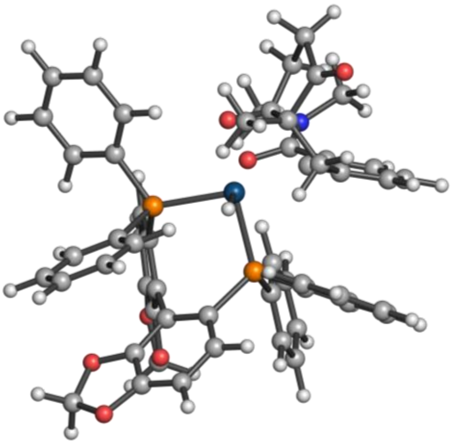

$\Delta\Delta G$= 31.8 kcal/mol

TS-3^{R}-6

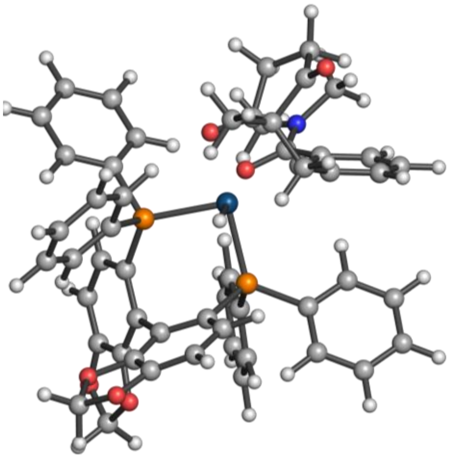

$\Delta\Delta G$= 29.1 kcal/mol

TS-3^{R}-7

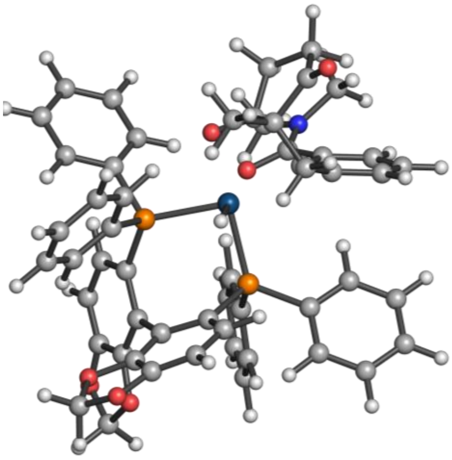

$\Delta\Delta G$= 33.2 kcal/mol

TS-3^{R}-8

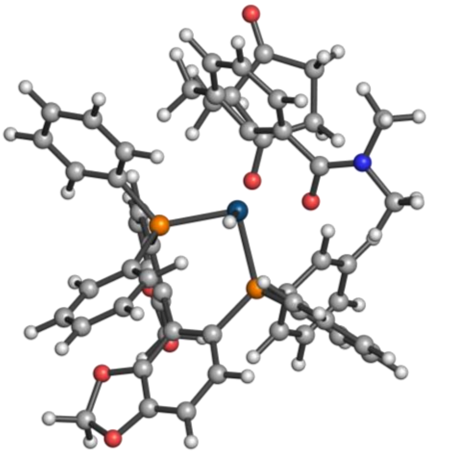

$\Delta\Delta G$= 34.2 kcal/mol

TS-3^{R}-9

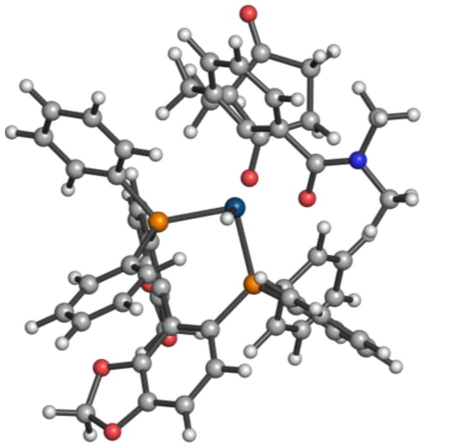

$\Delta\Delta G$= 31.2 kcal/mol

TS-3^{S}-1

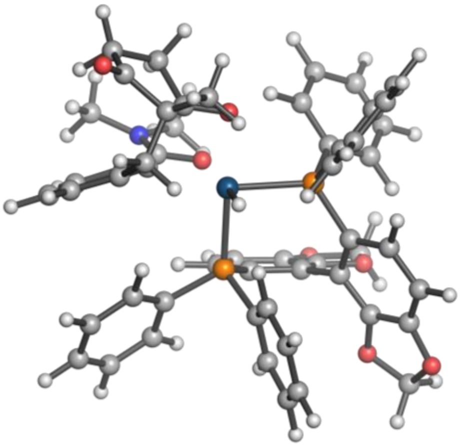

$\Delta\Delta G$= 25.9 kcal/mol

TS-3^{S}-2

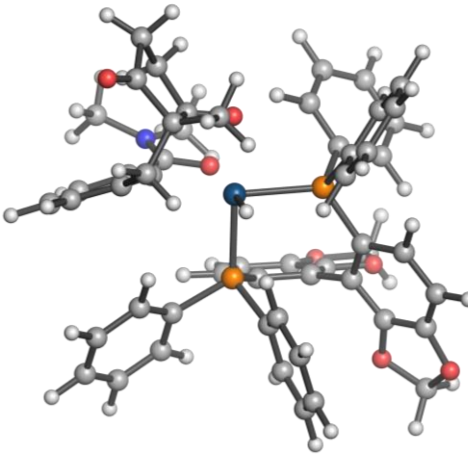

$\Delta\Delta G$= 28.0 kcal/mol

TS-3^{S}-3

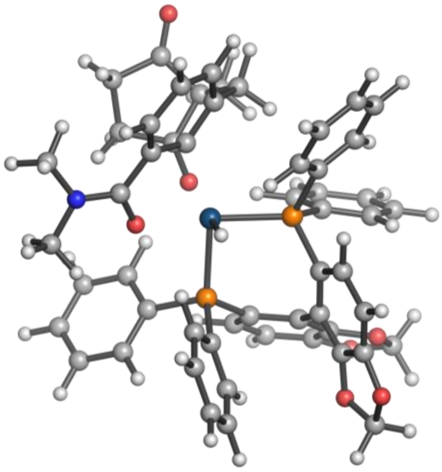

$\Delta\Delta G$= 27.3 kcal/mol

TS-3^{S}-4

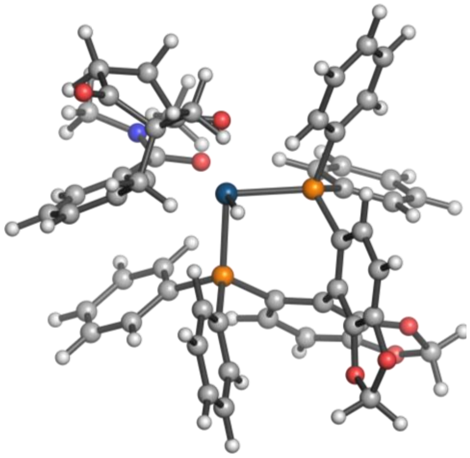

6

$\Delta\Delta G$= 25.6 kcal/mol

TS-3^{S}-5

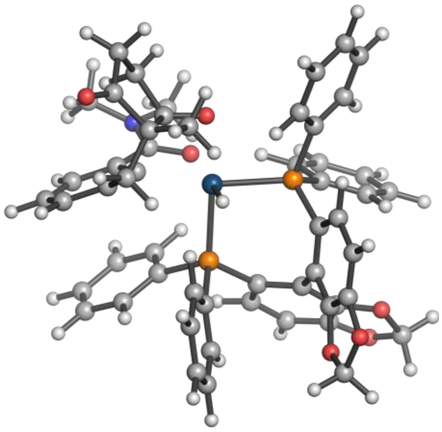

$\Delta\Delta G$= 28.6 kcal/mol

TS-3^{S}-6

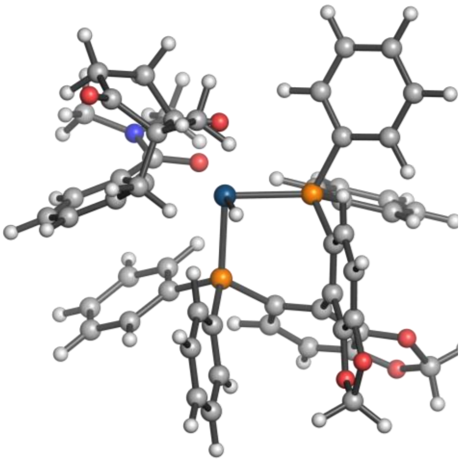

$\Delta\Delta G$= 27.0 kcal/mol

I-1’

Zero-point correction= 0.890315 (Hartree/Particle)
Thermal correction to Energy= 0.949350
Thermal correction to Enthalpy= 0.950294
Thermal correction to Gibbs Free Energy= 0.792441
Sum of electronic and zero-point Energies= -3453.697964
Sum of electronic and thermal Energies= -3453.638929
Sum of electronic and thermal Enthalpies= -3453.637985
Sum of electronic and thermal Free Energies= -3453.795839
HF(M06/6-311++G(d,p) and SDD, SMD[1,4-Dioxane]) = -3453.15801064

C	2.875346	2.896156	0.230775
O	1.887579	2.260392	-0.221381
N	2.814526	4.239080	0.364828
C	1.555281	4.935328	0.096087
H	1.556348	5.875530	0.654205
H	1.441712	5.151206	-0.972246
H	0.716344	4.322938	0.420155
C	3.984968	5.108405	0.500423
H	4.001163	5.820041	-0.333348
H	3.937732	5.675162	1.437176
H	4.904698	4.527627	0.478893
C	4.114887	2.168348	0.633454
C	4.561680	1.167790	-0.227083
C	3.929968	-2.898606	-2.698404
C	4.739504	2.335860	1.878357
C	5.792061	1.494101	2.238824
C	6.189570	0.456956	1.390789
C	5.559758	0.269563	0.153321
H	4.392503	3.099395	2.568381
H	6.283854	1.625648	3.198065
H	6.980498	-0.220607	1.701646
C	4.943126	-3.120014	-1.570033
O	5.812130	-3.958873	-1.557694
C	4.718227	-2.086214	-0.449143
C	2.832639	-1.997020	-2.106917
C	3.321174	-1.558167	-0.738563
C	5.797149	-0.943765	-0.706510
H	5.780112	-0.662770	-1.766820
H	6.770149	-1.406657	-0.512720
C	4.878375	-2.693935	0.948093
H	4.128504	-3.469007	1.128197
H	5.867891	-3.154560	1.024323
H	4.768224	-1.930418	1.721094
O	2.685672	-0.856259	0.050353
H	2.601075	-1.110790	-2.709893
H	1.880028	-2.521892	-1.972652
H	3.563624	-3.864461	-3.057310
H	4.448724	-2.427147	-3.542221
H	4.068130	1.056403	-1.187203
Ir	0.761871	0.279608	-0.105597
P	-1.075989	1.513176	-0.546177
P	-0.421072	-1.541015	0.461447
C	-2.510820	0.612156	-1.282169
C	-3.260045	-0.311600	-0.491969
C	-2.828325	0.798366	-2.633590
C	-4.284000	-0.977440	-1.148089
C	-3.872066	0.105012	-3.270717
H	-2.258918	1.505158	-3.223361
C	-4.582971	-0.785241	-2.496077
H	-4.106039	0.264951	-4.317180
C	-1.891000	-1.182126	1.532763
C	-3.052818	-0.579210	0.958147
C	-1.853226	-1.455020	2.905520
C	-4.083547	-0.289573	1.838197
C	-2.917928	-1.149981	3.771826
H	-0.981315	-1.931538	3.333393
C	-4.024294	-0.557400	3.204508
H	-2.869775	-1.373417	4.831605
C	0.620334	-2.663707	1.505396
C	0.715722	-4.047824	1.301619
C	1.345835	-2.086615	2.564867
C	1.492132	-4.839449	2.154096
H	0.182376	-4.520162	0.484485
C	2.102582	-2.880258	3.425758
H	1.310464	-1.010545	2.711765
C	2.174912	-4.261730	3.224812
H	1.551732	-5.910732	1.983063
H	2.640619	-2.419587	4.249695
H	2.764672	-4.881231	3.894605
C	-1.130988	-2.638188	-0.845710
C	-1.996372	-3.695818	-0.517645
C	-0.819367	-2.407096	-2.192620
C	-2.508598	-4.523816	-1.517208
H	-2.277703	-3.867924	0.517415
C	-1.339513	-3.231490	-3.193220
H	-0.197176	-1.553351	-2.445348
C	-2.177680	-4.295751	-2.856405
H	-3.167421	-5.345837	-1.250536
H	-1.098589	-3.036450	-4.234606
H	-2.579460	-4.940032	-3.633472
C	-0.701756	2.797613	-1.828088
C	-1.330369	4.050707	-1.855304
C	0.221290	2.482085	-2.838670
C	-1.042445	4.966556	-2.870739
H	-2.042480	4.322962	-1.083785
C	0.504656	3.394771	-3.854958
H	0.716191	1.514854	-2.817662
C	-0.126068	4.641663	-3.872561
H	-1.538820	5.933089	-2.878134
H	1.217375	3.133888	-4.632765

H	0.092937	5.353289	-4.663786
C	-1.814426	2.456124	0.859015
C	-1.035411	2.679046	2.004630
C	-3.121777	2.967560	0.811935
C	-1.546165	3.415729	3.075250
H	-0.037790	2.251054	2.053403
C	-3.628721	3.706011	1.882519
H	-3.751041	2.775833	-0.052260
C	-2.841337	3.933872	3.014086
H	-0.936613	3.576892	3.960284
H	-4.640077	4.100456	1.833287
H	-3.239458	4.505537	3.847828
O	-5.137015	-1.909132	-0.619193
O	-5.631985	-1.579606	-2.858324
O	-5.280577	0.306370	1.540500
O	-5.177837	-0.149257	3.809891
C	-5.962207	-2.359516	-1.701126
H	-5.753449	-3.416602	-1.903108
H	-7.015305	-2.205538	-1.446781
C	-6.025499	0.345459	2.764655
H	-6.308878	1.379240	2.985088
H	-6.909578	-0.296802	2.676797

I-1

Zero-point correction= 0.889006 (Hartree/Particle)
Thermal correction to Energy= 0.949008
Thermal correction to Enthalpy= 0.949952
Thermal correction to Gibbs Free Energy= 0.785410
Sum of electronic and zero-point Energies= -3453.697075
Sum of electronic and thermal Energies= -3453.637073
Sum of electronic and thermal Enthalpies= -3453.636129
Sum of electronic and thermal Free Energies= -3453.800670
HF(M06/6-311++G(d,p) and SDD, SMD[1,4Dioxane])= -3453.15429090

C	-2.501809	2.928627	-0.626348
O	-1.329565	2.720443	-0.205571
N	-2.983056	4.186341	-0.626834
C	-2.092573	5.282919	-0.232164
H	-2.415765	6.192950	-0.744623
H	-2.129572	5.445793	0.851307
H	-1.067819	5.043344	-0.511557
C	-4.398455	4.540000	-0.741281
H	-4.697893	5.106263	0.148156
H	-4.569338	5.166550	-1.623879
H	-5.016742	3.647232	-0.810458
C	-3.306694	1.780828	-1.138020
C	-3.278534	0.575176	-0.409766
C	-7.224929	-1.041192	1.728559
C	-4.008146	1.845266	-2.350750
C	-4.692578	0.721036	-2.808167
C	-4.684598	-0.464162	-2.068791
C	-3.974584	-0.558565	-0.864669
H	-4.001554	2.757600	-2.939470
H	-5.233501	0.765388	-3.748970
H	-5.238486	-1.326797	-2.424924
C	-6.443396	-1.778924	0.634000
O	-6.906003	-2.160343	-0.418724
C	-4.985092	-1.968294	1.067848
C	-6.180410	-0.537286	2.744166
C	-4.809800	-0.944590	2.191902
C	-3.957262	-1.855292	-0.079530
H	-4.140320	-2.686782	-0.766902
H	-2.965418	-2.007672	0.355965
C	-4.876722	-3.381385	1.714793
H	-5.571030	-3.507974	2.552798
H	-5.108445	-4.143520	0.964082
H	-3.858669	-3.538052	2.085568
O	-3.744340	-0.519944	2.592403
H	-6.195427	0.546968	2.893547
H	-6.304603	-0.986380	3.737144
H	-7.955806	-1.728928	2.169323
H	-7.802687	-0.236238	1.262548
H	-2.780365	0.538457	0.559357
Ir	-0.394821	0.785962	-0.354625
P	1.502046	1.522349	0.528434
P	0.390770	-1.281175	-0.762683
C	2.424339	0.312580	1.565931
C	3.076497	-0.808423	0.961467
C	2.433217	0.465531	2.958598
C	3.680762	-1.694747	1.839179
C	3.059905	-0.451528	3.819058
H	1.947753	1.324300	3.403931
C	3.673331	-1.532284	3.224303
H	3.060374	-0.314313	4.894385
C	2.124361	-1.393539	-1.382201
C	3.202353	-1.072896	-0.501067
C	2.377734	-1.709536	-2.721449
C	4.467486	-1.089515	-1.066990
C	3.675322	-1.720749	-3.263812
H	1.552402	-1.959774	-3.376419
C	4.703631	-1.396622	-2.406503
H	3.856238	-1.971908	-4.302821
C	-0.638425	-1.995732	-2.122969
C	-1.114783	-3.315422	-2.120423
C	-0.964037	-1.158253	-3.206198
C	-1.890945	-3.788348	-3.181324
H	-0.881176	-3.978223	-1.294243
C	-1.732692	-1.636229	-4.268018
H	-0.602446	-0.132504	-3.214283
C	-2.198371	-2.953188	-4.257218
H	-2.249517	-4.813948	-3.168422
H	-1.966665	-0.980986	-5.102431
H	-2.796147	-3.327340	-5.083670
C	0.316407	-2.529954	0.592982
C	1.017019	-3.745078	0.515576

C	-0.474680	-2.266521	1.721353
C	0.911979	-4.685355	1.541852
H	1.652687	-3.952919	-0.340585
C	-0.574366	-3.207961	2.748773
H	-0.998464	-1.317647	1.798731
C	0.115508	-4.418799	2.659283
H	1.453028	-5.625033	1.469294
H	-1.182858	-2.986969	3.621448
H	0.039391	-5.150199	3.459238
C	1.130509	2.940412	1.653526
C	1.869066	4.132224	1.633146
C	0.055241	2.820566	2.551992
C	1.546673	5.177416	2.503077
H	2.696343	4.252287	0.942497
C	-0.255832	3.860414	3.427069
H	-0.538266	1.910557	2.557740
C	0.489151	5.043285	3.403245
H	2.128358	6.094661	2.477063
H	-1.081028	3.748098	4.125098
H	0.246258	5.854660	4.083888
C	2.750439	2.174977	-0.656715
C	2.348720	2.540621	-1.949467
C	4.089825	2.357210	-0.274549
C	3.269635	3.086228	-2.845340
H	1.316139	2.376775	-2.245698
C	5.006571	2.906990	-1.171882
H	4.419198	2.063804	0.717905
C	4.598133	3.272151	-2.457487
H	2.951006	3.361548	-3.846882
H	6.039390	3.050394	-0.865961
H	5.314435	3.697152	-3.155132
O	4.353323	-2.843401	1.518040
O	4.341535	-2.562386	3.817775
O	5.655637	-0.814297	-0.444077
O	6.041916	-1.330658	-2.669761
C	4.768231	-3.429882	2.758183
H	4.291596	-4.409303	2.875980
H	5.859560	-3.516397	2.775899
C	6.671904	-0.917122	-1.450586
H	7.140363	0.062957	-1.596442
H	7.410381	-1.666857	-1.149178

TS-1

Zero-point correction=
0.885133 (Hartree/Particle)
Thermal correction to Energy=
0.944007
Thermal correction to Enthalpy=
0.944951
Thermal correction to Gibbs Free Energy=
0.786599
Sum of electronic and zero-point Energies=
-3453.671293
Sum of electronic and thermal Energies=
-3453.612419
Sum of electronic and thermal Enthalpies=
-3453.611475
Sum of electronic and thermal Free Energies=
-3453.769827
HF(M06/6-311++G(d,p) and SDD, SMD[1,4Dioxane])=
-3453.12596877

C	-2.135697	3.180641	-0.682607
O	-1.173227	2.833310	0.075806
N	-2.194238	4.477494	-1.051836
C	-1.269325	5.418657	-0.405654
H	-1.730749	6.409519	-0.411265
H	-1.079621	5.108117	0.620406
H	-0.313407	5.463263	-0.941517
C	-2.885884	5.004651	-2.230635
H	-2.219529	5.721380	-2.720180
H	-3.106083	4.210014	-2.942308
H	-3.811649	5.527535	-1.962498
C	-3.131777	2.139637	-1.033285
C	-2.776835	0.792725	-0.688096
C	-6.685465	-1.175786	1.953209
C	-4.394166	2.439356	-1.563067
C	-5.304207	1.418796	-1.818617
C	-4.986012	0.112884	-1.463849
C	-3.769848	-0.206413	-0.834428
H	-4.692374	3.459775	-1.761036
H	-6.269977	1.646727	-2.259538
H	-5.707358	-0.678031	-1.639776
C	-5.998276	-1.883144	0.779720
O	-6.573785	-2.372767	-0.167643
C	-4.475408	-1.913745	0.987716
C	-5.565949	-0.723730	2.907308
C	-4.251472	-0.955389	2.158129
C	-3.623883	-1.610853	-0.274563
H	-3.909736	-2.344397	-1.036313
H	-2.585205	-1.805492	-0.012040
C	-4.151863	-3.354686	1.486264
H	-4.730141	-3.621081	2.378283
H	-4.403005	-4.069196	0.696197
H	-3.088436	-3.439243	1.726932
O	-3.185419	-0.456355	2.464502
H	-5.630820	0.323391	3.216875
H	-5.542704	-1.320881	3.828156
H	-7.419737	-1.846486	2.411101
H	-7.252160	-0.330812	1.544034
H	-2.079853	0.409063	0.553560
Ir	-0.657217	0.776373	-0.168969
P	1.416631	1.350802	0.812628
P	0.077201	-1.285606	-0.833098
C	2.423876	-0.003450	1.537624
C	2.993790	-0.989435	0.676681
C	2.598523	-0.084992	2.924028
C	3.717007	-1.989314	1.305563
C	3.331249	-1.119761	3.531496
H	2.163139	0.674003	3.562050
C	3.879899	-2.062052	2.689177

H	3.459438	-1.171350	4.606733
C	1.755907	-1.152764	-1.613777
C	2.929008	-0.970817	-0.813148
C	1.860769	-1.155596	-3.011001
C	4.116034	-0.807943	-1.510408
C	3.081273	-0.982873	-3.686784
H	0.974019	-1.307905	-3.612221
C	4.198970	-0.805787	-2.902240
H	3.138203	-0.994107	-4.769328
C	-0.948339	-2.029831	-2.185528
C	-1.190292	-3.407434	-2.293879
C	-1.490038	-1.172227	-3.158492
C	-1.951624	-3.913171	-3.350097
H	-0.799913	-4.092953	-1.550475
C	-2.240063	-1.680090	-4.219529
H	-1.325714	-0.101604	-3.079625
C	-2.475304	-3.053386	-4.316617
H	-2.134378	-4.982130	-3.414615
H	-2.646401	-1.002341	-4.965103
H	-3.065220	-3.449724	-5.138123
C	0.263882	-2.620641	0.421304
C	1.023110	-3.772690	0.153100
C	-0.360974	-2.486422	1.669819
C	1.134074	-4.781767	1.109847
H	1.543619	-3.874861	-0.794718
C	-0.237962	-3.495806	2.628769
H	-0.937141	-1.592892	1.891538
C	0.503207	-4.645289	2.349552
H	1.717061	-5.671629	0.888573
H	-0.716958	-3.377434	3.596837
H	0.596553	-5.428678	3.096504
C	1.044142	2.468328	2.232928
C	1.835944	3.585760	2.534966
C	-0.068590	2.179170	3.042226
C	1.524564	4.395478	3.629904
H	2.694131	3.832092	1.919007
C	-0.369836	2.985660	4.139675
H	-0.703713	1.330171	2.806108
C	0.424672	4.096374	4.435162
H	2.145161	5.259209	3.851874
H	-1.230070	2.748294	4.759312
H	0.186292	4.726173	5.287776
C	2.593625	2.294322	-0.245054
C	2.108647	2.946678	-1.388882
C	3.957242	2.396414	0.072990
C	2.968073	3.693467	-2.196087
H	1.056527	2.863994	-1.646706
C	4.813829	3.148824	-0.732825
H	4.352329	1.881528	0.943593
C	4.321434	3.797965	-1.867745
H	2.583627	4.188076	-3.083889
H	5.865815	3.228308	-0.472345
H	4.990506	4.379846	-2.495425
O	4.342365	-3.055218	0.715167
O	4.616459	-3.165041	3.011837
O	5.364490	-0.619746	-0.978983
O	5.493194	-0.622071	-3.292221
C	5.018099	-3.754682	1.768341
H	4.723292	-4.807639	1.753414
H	6.102424	-3.645058	1.645125
C	6.253479	-0.442467	-2.089912
H	6.665585	0.572908	-2.068248
H	7.048151	-1.193611	-2.046059

I-2

Zero-point correction=
0.887473 (Hartree/Particle)
Thermal correction to Energy=
0.946376
Thermal correction to Enthalpy=
0.947320
Thermal correction to Gibbs Free Energy=
0.789605
Sum of electronic and zero-point Energies=
-3453.694927
Sum of electronic and thermal Energies=
-3453.636025
Sum of electronic and thermal Enthalpies=
-3453.635081
Sum of electronic and thermal Free Energies=
-3453.792795
HF(M06/6-311++G(d,p) and SDD, SMD[1,4Dioxane])=
-3453.14323206

C	-2.208254	3.050988	-0.913676
O	-1.233668	2.785945	-0.114888
N	-2.261218	4.294656	-1.423759
C	-1.375681	5.332198	-0.881863
H	-1.915883	6.283411	-0.887376
H	-1.087049	5.078254	0.136052
H	-0.471732	5.435352	-1.494026
C	-2.990862	4.684854	-2.632188
H	-2.327988	5.307176	-3.242109
H	-3.271964	3.808540	-3.213832
H	-3.885844	5.270588	-2.392341
C	-3.191603	1.979032	-1.151871
C	-2.760602	0.677695	-0.731212
C	-6.182833	-0.779136	2.205409
C	-4.502064	2.239342	-1.588119
C	-5.409047	1.194082	-1.686201
C	-5.021658	-0.078286	-1.271317
C	-3.747352	-0.339856	-0.735354
H	-4.831646	3.244733	-1.819323
H	-6.415694	1.369150	-2.053596
H	-5.739009	-0.890542	-1.336765
C	-5.747048	-1.826536	1.177960
O	-6.496925	-2.434881	0.445806
C	-4.220561	-2.024393	1.209601
C	-4.948624	-0.489938	3.072972
C	-3.754852	-1.105604	2.341332
C	-3.529255	-1.731322	-0.161384

H	-3.911198	-2.482868	-0.862008
H	-2.466572	-1.925624	-0.023703
C	-3.946666	-3.497918	1.608760
H	-4.373242	-3.740509	2.588678
H	-4.408761	-4.157663	0.868375
H	-2.870666	-3.687989	1.645496
O	-2.593947	-0.918057	2.658121
H	-4.759505	0.572782	3.253553
H	-5.016959	-0.966775	4.059756
H	-7.056935	-1.134121	2.758941
H	-6.502254	0.108625	1.644310
H	-1.194342	-0.276125	1.037174
Ir	-0.776660	0.706838	-0.086854
P	1.283973	1.262138	1.065938
P	0.083129	-1.193134	-1.017961
C	2.335804	-0.147786	1.592656
C	2.949222	-0.954151	0.585511
C	2.503173	-0.450263	2.947450
C	3.713423	-2.012582	1.048865
C	3.272780	-1.544060	3.383787
H	2.032613	0.177165	3.695182
C	3.866662	-2.308989	2.403772
H	3.396414	-1.769914	4.436886
C	1.743185	-0.796708	-1.731136
C	2.894340	-0.686044	-0.882850
C	1.860300	-0.558804	-3.107176
C	4.075126	-0.342075	-1.521140
C	3.074989	-0.204866	-3.719105
H	0.991986	-0.668698	-3.743661
C	4.171162	-0.098611	-2.892032
H	3.145472	-0.033144	-4.787145
C	-0.928361	-1.765323	-2.459627
C	-1.219774	-3.117789	-2.691915
C	-1.437932	-0.805699	-3.352926
C	-1.987950	-3.499524	-3.794860
H	-0.861051	-3.881579	-2.012430
C	-2.191939	-1.189680	-4.460519
H	-1.250839	0.249018	-3.177358
C	-2.470607	-2.540111	-4.684252
H	-2.207387	-4.551370	-3.954287
H	-2.570280	-0.433628	-5.142617
H	-3.064722	-2.840071	-5.542637
C	0.406229	-2.716645	-0.031150
C	1.201494	-3.733046	-0.591787
C	-0.113739	-2.892792	1.258483
C	1.450261	-4.908549	0.115731
H	1.637131	-3.603365	-1.577982
C	0.150005	-4.067195	1.968856
H	-0.720057	-2.121927	1.721499
C	0.925177	-5.078126	1.399641
H	2.057257	-5.688563	-0.335509
H	-0.250597	-4.185662	2.971628
H	1.123503	-5.991206	1.953841
C	0.818046	2.141449	2.618523
C	1.519650	3.265057	3.082596
C	-0.287898	1.676469	3.353500
C	1.129015	3.905793	4.260079
H	2.369767	3.647033	2.528125
C	-0.667410	2.316176	4.534156
H	-0.858401	0.818481	3.012297
C	0.037520	3.432354	4.989567
H	1.681058	4.775416	4.605581
H	-1.519319	1.940726	5.094082
H	-0.263217	3.931411	5.906534
C	2.473129	2.393689	0.221889
C	2.033572	3.197137	-0.840683
C	3.813921	2.480052	0.631246
C	2.915135	4.069670	-1.481019
H	0.997626	3.144238	-1.158081
C	4.691318	3.360346	-0.004473
H	4.175809	1.855040	1.441901
C	4.244726	4.154987	-1.063281
H	2.565347	4.681991	-2.307939
H	5.723230	3.425781	0.329883
H	4.929337	4.837168	-1.559508
O	4.399870	-2.928802	0.298752
O	4.653539	-3.415107	2.549817
O	5.305567	-0.181513	-0.943788
O	5.454592	0.219607	-3.219807
C	5.054027	-3.802423	1.229307
H	4.739800	-4.833804	1.041817
H	6.140238	-3.695268	1.130024
C	6.189280	0.247505	-1.987592
H	6.521038	1.272393	-1.786071
H	7.037495	-0.440014	-2.054623

H_2

Zero-point correction= 0.010147 (Hartree/Particle)
Thermal correction to Energy= 0.012508
Thermal correction to Enthalpy= 0.013452
Thermal correction to Gibbs Free Energy= -0.001340
Sum of electronic and zero-point Energies= -1.165335
Sum of electronic and thermal Energies= -1.162975
Sum of electronic and thermal Enthalpies= -1.162031
Sum of electronic and thermal Free Energies= -1.176822
HF(M06/6-311++G(d,p) and SDD, SMD[1,4-Dioxane]) = -1.16972262904

H	0.000000	0.000000	0.371359
H	0.000000	0.000000	-0.371359

I-1bis

Zero-point correction= 0.906982 (Hartree/Particle)
Thermal correction to Energy= 0.967322
Thermal correction to Enthalpy= 0.968266
Thermal correction to Gibbs Free Energy= 0.804105
Sum of electronic and zero-point Energies= -3454.876119
Sum of electronic and thermal Energies= -3454.815779

Sum of electronic and thermal Enthalpies= -3454.814835
Sum of electronic and thermal Free Energies= -3454.978995
HF(M06/6-311++G(d,p) and SDD, SMD[1,4-Dioxane]) = -3454.34429360

C	-2.025199	0.131782	2.644891
O	-0.976584	0.678344	2.209979
N	-2.337046	0.202910	3.954672
C	-1.424725	0.872528	4.883203
H	-1.383600	0.300994	5.815742
H	-0.432192	0.934144	4.441050
H	-1.780413	1.885468	5.108145
C	-3.634474	-0.166517	4.521239
H	-4.065893	0.703763	5.029132
H	-4.318269	-0.491012	3.739818
H	-3.515997	-0.972395	5.254459
C	-2.893544	-0.627151	1.693186
C	-3.328743	0.028867	0.527028
C	-7.711268	1.419816	0.013806
C	-3.197083	-1.981101	1.890132
C	-3.930355	-2.664471	0.921105
C	-4.366497	-2.010926	-0.234042
C	-4.076218	-0.656433	-0.445955
H	-2.842633	-2.499506	2.776314
H	-4.152820	-3.718310	1.058433
H	-4.938586	-2.554249	-0.978730
C	-6.258539	1.582417	-0.451162
O	-5.450695	2.341904	0.040148
C	-5.984385	0.641681	-1.628480
C	-8.228695	0.118169	-0.629893
C	-7.084029	-0.419419	-1.496946
C	-4.550971	0.068740	-1.689345
H	-3.868044	0.897119	-1.903382
H	-4.517224	-0.617831	-2.542511
C	-6.277317	1.439035	-2.934042
H	-7.292602	1.850172	-2.949723
H	-5.571599	2.271234	-3.022377
H	-6.166405	0.777487	-3.799223
O	-7.050677	-1.519111	-2.005853
H	-8.502840	-0.650469	0.100135
H	-9.111788	0.272021	-1.260886
H	-8.280315	2.302629	-0.301994
H	-7.736250	1.412222	1.107886
H	-3.175075	1.097532	0.415422
Ir	-0.499443	0.604260	-0.041381
P	1.332468	1.895240	-0.111623
P	0.578596	-1.593492	-0.221340
C	2.697653	1.102707	0.828400
C	3.355742	-0.079631	0.355958
C	3.014586	1.630566	2.089120
C	4.303721	-0.615318	1.213252
C	3.975006	1.053317	2.935280
H	2.508894	2.524544	2.431956
C	4.603482	-0.080327	2.467120
H	4.213089	1.480801	3.902661
C	2.030654	-1.510238	-1.364861
C	3.170813	-0.744994	-0.969199
C	2.022703	-2.172017	-2.597259
C	4.228018	-0.731704	-1.865142
C	3.106310	-2.121484	-3.493104
H	1.159290	-2.759848	-2.882163
C	4.201591	-1.388274	-3.094387
H	3.081919	-2.640381	-4.444728
C	-0.571089	-2.793037	-1.036467
C	-0.787137	-4.093572	-0.556697
C	-1.270066	-2.374999	-2.184587
C	-1.662006	-4.959427	-1.218512
H	-0.271202	-4.441030	0.331234
C	-2.130944	-3.246660	-2.851609
H	-1.128239	-1.368030	-2.565559
C	-2.329510	-4.543274	-2.370387
H	-1.811848	-5.964776	-0.834333
H	-2.646298	-2.912663	-3.748082
H	-2.999711	-5.222121	-2.890173
C	1.272782	-2.517917	1.219135
C	2.100016	-3.639859	1.038898
C	0.988221	-2.084411	2.521609
C	2.605037	-4.329493	2.141674
H	2.352180	-3.972700	0.036224
C	1.501348	-2.771430	3.624558
H	0.392523	-1.189750	2.664539
C	2.303747	-3.898235	3.437371
H	3.233441	-5.202879	1.989390
H	1.280068	-2.423060	4.629843
H	2.697169	-4.436353	4.295469
C	1.094169	3.545412	0.685042
C	1.888133	4.640096	0.306029
C	0.133692	3.714190	1.694781
C	1.723167	5.878697	0.927481
H	2.629026	4.536548	-0.478990
C	-0.023766	4.954604	2.314440
H	-0.486642	2.876493	1.991001
C	0.767454	6.039448	1.931713
H	2.338805	6.718817	0.619074
H	-0.773725	5.075398	3.091380
H	0.636825	7.006120	2.409870
C	1.974852	2.326723	-1.776775
C	1.074370	2.617252	-2.813465
C	3.351226	2.494415	-1.994850
C	1.545459	3.057988	-4.049937
H	0.008249	2.500143	-2.650161
C	3.816595	2.934566	-3.235633
H	4.063631	2.276580	-1.206155
C	2.916605	3.215472	-4.264675
H	0.839933	3.279025	-4.845743
H	4.884132	3.059856	-3.393997
H	3.281129	3.557216	-5.229236
O	5.084103	-1.718248	0.992300

O	5.569050	-0.826447	3.072330
O	5.422895	-0.072282	-1.725851
O	5.372673	-1.167491	-3.764049
C	5.805978	-1.947985	2.208028
H	5.431817	-2.860517	2.687311
H	6.875090	-2.018837	1.991150
C	6.223596	-0.468531	-2.848675
H	6.631207	0.420481	-3.336524
H	7.024748	-1.136776	-2.508167
H	-0.432421	0.597316	-1.613437
H	-1.277221	2.013494	-0.215708

TS-1bis

Zero-point correction= 0.901392 (Hartree/Particle)
Thermal correction to Energy= 0.960791
Thermal correction to Enthalpy= 0.961736
Thermal correction to Gibbs Free Energy= 0.802952
Sum of electronic and zero-point Energies= -3454.842504
Sum of electronic and thermal Energies= -3454.783105
Sum of electronic and thermal Enthalpies= -3454.782160
Sum of electronic and thermal Free Energies= -3454.940944
HF(M06/6-311++G(d,p) and SDD, SMD[1,4-Dioxane]) = -3454.31124744

C	-1.968485	-0.335707	2.892454
O	-0.897284	0.204243	2.478886
N	-2.231654	-0.303098	4.219843
C	-1.205006	0.211284	5.130073
H	-1.292226	-0.314241	6.085352
H	-0.214791	0.045408	4.710410
H	-1.337774	1.286534	5.305265
C	-3.557251	-0.447134	4.827943
H	-3.696559	0.371256	5.542142
H	-4.340336	-0.379721	4.075109
H	-3.651744	-1.395283	5.370136
C	-2.876410	-0.958039	1.900025
C	-2.727729	-0.507397	0.562997
C	-6.694332	2.054312	-0.583182
C	-3.806453	-1.962256	2.212974
C	-4.661883	-2.440000	1.225633
C	-4.639050	-1.864831	-0.044267
C	-3.699484	-0.877791	-0.387149
H	-3.843175	-2.395987	3.205769
H	-5.362570	-3.238348	1.450963
H	-5.348499	-2.199175	-0.795260
C	-5.198577	1.757777	-0.734850
O	-4.328377	2.229034	-0.030423
C	-4.974740	0.789514	-1.896768
C	-7.433857	0.979637	-1.403437
C	-6.349380	0.122210	-2.064964
C	-3.799191	-0.207390	-1.745254
H	-2.869386	0.323654	-1.955147
H	-3.939842	-0.960685	-2.528072
C	-4.753287	1.651270	-3.177275
H	-5.587140	2.335117	-3.370269
H	-3.841547	2.246999	-3.066602
H	-4.650004	0.990816	-4.044235
O	-6.542832	-0.924778	-2.643366
H	-8.067854	0.327708	-0.793552
H	-8.082337	1.399794	-2.180815
H	-6.887769	3.070934	-0.946745
H	-6.950400	2.052783	0.480754
H	-2.371407	1.064689	0.406035
Ir	-0.786977	0.470995	0.239239
P	1.175668	1.824456	0.173664
P	0.421907	-1.556565	-0.324820
C	2.671788	0.998542	0.855624
C	3.284303	-0.088505	0.157871
C	3.145136	1.385422	2.115865
C	4.353383	-0.688946	0.805574
C	4.224395	0.745522	2.749229
H	2.672984	2.213658	2.629643
C	4.808351	-0.297792	2.064752
H	4.583326	1.062577	3.721872
C	1.753146	-1.285601	-1.586176
C	2.936619	-0.573128	-1.211548
C	1.624379	-1.784889	-2.887518
C	3.888592	-0.421291	-2.207816
C	2.607403	-1.604682	-3.876715
H	0.748929	-2.358791	-3.157772
C	3.735096	-0.908700	-3.504227
H	2.485131	-2.003582	-4.877298
C	-0.712826	-2.810978	-1.070049
C	-0.918452	-4.066402	-0.478724
C	-1.435249	-2.491463	-2.233037
C	-1.800987	-4.985930	-1.051505
H	-0.391469	-4.337557	0.428405
C	-2.296531	-3.419886	-2.816698
H	-1.323095	-1.512556	-2.688182
C	-2.482692	-4.672106	-2.226437
H	-1.945659	-5.952820	-0.577767
H	-2.829365	-3.161753	-3.727780
H	-3.158028	-5.393312	-2.677660
C	1.329360	-2.509576	0.970919
C	2.138740	-3.594947	0.589951
C	1.230259	-2.175418	2.327351
C	2.815516	-4.342301	1.552884
H	2.240147	-3.856830	-0.459040
C	1.917518	-2.921198	3.288811
H	0.636280	-1.319764	2.623058
C	2.704865	-4.007631	2.906257
H	3.427845	-5.186000	1.246424
H	1.839167	-2.649849	4.338065
H	3.231480	-4.591137	3.656476
C	0.977966	3.344772	1.209316
C	1.657102	4.532959	0.896327

C	0.150321	3.312632	2.343473
C	1.507088	5.664963	1.698931
H	2.295667	4.585648	0.021544
C	0.008493	4.445932	3.145811
H	-0.375459	2.399378	2.599187
C	0.683047	5.625221	2.824596
H	2.032097	6.579603	1.438456
H	-0.636588	4.408970	4.019613
H	0.564812	6.508805	3.445438
C	1.661961	2.507585	-1.464883
C	0.665510	2.849072	-2.391718
C	3.002513	2.793618	-1.767484
C	1.003864	3.459252	-3.600014
H	-0.374921	2.639903	-2.165186
C	3.337095	3.401273	-2.979042
H	3.788672	2.535385	-1.065478
C	2.339971	3.734709	-3.897522
H	0.222909	3.719193	-4.309061
H	4.378499	3.617474	-3.201248
H	2.602334	4.207965	-4.839473
O	5.121123	-1.724810	0.345357
O	5.867932	-1.072285	2.437463
O	5.090897	0.229848	-2.106223
O	4.828124	-0.586671	-4.255970
C	6.017791	-2.062801	1.411187
H	5.749900	-3.047159	1.813319
H	7.046611	-2.049763	1.040865
C	5.765024	0.014850	-3.353570
H	6.095686	0.974660	-3.758911
H	6.612709	-0.664300	-3.199441
H	-0.877415	0.657157	-1.322369
H	-1.597821	1.936966	0.404280

I-2bis

Zero-point correction= 0.904492 (Hartree/Particle)
Thermal correction to Energy= 0.964071
Thermal correction to Enthalpy= 0.965015
Thermal correction to Gibbs Free Energy= 0.805330
Sum of electronic and zero-point Energies= -3454.854440
Sum of electronic and thermal Energies= -3454.794861
Sum of electronic and thermal Enthalpies= -3454.793917
Sum of electronic and thermal Free Energies= -3454.953602
HF(M06/6-311++G(d,p) and SDD, SMD[1,4-Dioxane]) = -3454.32528397

C	-1.994067	-0.805298	2.793094
O	-0.918647	-0.197845	2.480381
N	-2.255253	-0.974175	4.112447
C	-1.224465	-0.611884	5.088491
H	-1.334939	-1.256986	5.964704
H	-0.233671	-0.749904	4.660317
H	-1.328766	0.433956	5.405528
C	-3.582808	-1.183363	4.697904
H	-3.690395	-0.502894	5.549046
H	-4.366041	-0.954706	3.977558
H	-3.709776	-2.209644	5.061930
C	-2.888871	-1.252716	1.708578
C	-2.659224	-0.635762	0.444245
C	-6.401296	2.187310	-0.214212
C	-3.901683	-2.214795	1.883907
C	-4.780510	-2.483503	0.844802
C	-4.692242	-1.733821	-0.328319
C	-3.667284	-0.794963	-0.535458
H	-3.982568	-2.776292	2.807242
H	-5.543988	-3.248044	0.952484
H	-5.418893	-1.899760	-1.118310
C	-4.930355	1.990200	-0.592013
O	-4.000337	2.471297	0.026818
C	-4.805201	1.132673	-1.851067
C	-7.230974	1.434515	-1.268616
C	-6.229180	0.592096	-2.063557
C	-3.713151	0.031376	-1.811585
H	-2.747073	0.512032	-1.968650
H	-3.915979	-0.613009	-2.674831
C	-4.519619	2.096590	-3.040622
H	-5.294794	2.863047	-3.152285
H	-3.562256	2.602174	-2.879496
H	-4.474904	1.521656	-3.971051
O	-6.511856	-0.347662	-2.774067
H	-8.008591	0.786946	-0.854200
H	-7.731879	2.119270	-1.964908
H	-6.625535	3.256877	-0.145765
H	-6.538852	1.777346	0.793935
H	-2.074988	1.750431	0.410715
Ir	-0.818444	0.380578	0.319152
P	1.198219	1.758427	0.451450
P	0.338429	-1.495750	-0.494711
C	2.708788	0.849813	0.973289
C	3.270447	-0.134050	0.103610
C	3.251281	1.064131	2.245596
C	4.365373	-0.821654	0.604185
C	4.355451	0.339029	2.727443
H	2.817301	1.819550	2.889238
C	4.889862	-0.605294	1.878423
H	4.770312	0.520809	3.712474
C	1.639848	-1.042295	-1.735399
C	2.849157	-0.406105	-1.303451
C	1.457792	-1.335502	-3.092477
C	3.758691	-0.102044	-2.304832
C	2.402138	-1.010674	-4.081587
H	0.570387	-1.860836	-3.414040
C	3.548783	-0.380787	-3.653905
H	2.236900	-1.254100	-5.124909
C	-0.781482	-2.695773	-1.343273
C	-0.954691	-4.000933	-0.857947
C	-1.521360	-2.296347	-2.469541

C	-1.818406	-4.889743	-1.502808
H	-0.418923	-4.335306	0.022151
C	-2.365152	-3.191776	-3.124612
H	-1.445234	-1.278772	-2.836529
C	-2.515332	-4.494197	-2.643382
H	-1.938004	-5.895669	-1.110638
H	-2.914568	-2.867791	-4.004093
H	-3.177108	-5.190558	-3.150193
C	1.298259	-2.557395	0.671743
C	2.095079	-3.583225	0.130145
C	1.261847	-2.383312	2.060324
C	2.819800	-4.429901	0.967077
H	2.149407	-3.722777	-0.945242
C	1.998869	-3.228071	2.894744
H	0.676425	-1.576994	2.481633
C	2.772200	-4.255100	2.353593
H	3.420354	-5.226010	0.535724
H	1.968702	-3.079993	3.970720
H	3.336415	-4.915791	3.006019
C	0.990817	3.089219	1.720953
C	1.519079	4.377334	1.542801
C	0.281384	2.802168	2.900823
C	1.340943	5.356038	2.522575
H	2.063851	4.625895	0.639032
C	0.112973	3.782797	3.879665
H	-0.133209	1.810537	3.053870
C	0.639219	5.062508	3.692385
H	1.750017	6.350209	2.366560
H	-0.435707	3.547928	4.787915
H	0.500472	5.826810	4.451748
C	1.686131	2.695374	-1.057491
C	0.696408	3.118667	-1.957232
C	3.019369	3.074665	-1.282009
C	1.032905	3.902888	-3.061548
H	-0.337873	2.833218	-1.793930
C	3.352607	3.857460	-2.388685
H	3.799694	2.753604	-0.599391
C	2.361222	4.272699	-3.280490
H	0.257062	4.223995	-3.750836
H	4.387505	4.147349	-2.549377
H	2.622437	4.882460	-4.140764
O	5.095537	-1.788741	-0.032091
O	5.959212	-1.427450	2.086290
O	4.972916	0.513398	-2.151165
O	4.614217	0.043986	-4.390777
C	6.059991	-2.255821	0.920308
H	5.831488	-3.293583	1.189888
H	7.064398	-2.166997	0.496236
C	5.570092	0.559187	-3.454091
H	5.807045	1.596179	-3.710058
H	6.468915	-0.067867	-3.467548
H	-0.905927	0.761208	-1.213395
H	-1.516442	1.943665	0.974800

I-3^{R}

Zero-point correction= 0.889112 (Hartree/Particle)
Thermal correction to Energy= 0.947265
Thermal correction to Enthalpy= 0.948210
Thermal correction to Gibbs Free Energy= 0.792941
Sum of electronic and zero-point Energies= -3453.700980
Sum of electronic and thermal Energies= -3453.642827
Sum of electronic and thermal Enthalpies= -3453.641883
Sum of electronic and thermal Free Energies= -3453.797151
HF(M06/6-311++G(d,p) and SDD, SMD[1,4Dioxane])= -3453.14897281

C	-2.321826	-1.525325	2.177228
O	-1.357053	-0.697295	2.094414
N	-2.622516	-2.001888	3.411937
C	-1.758810	-1.642422	4.541289
H	-1.708933	-2.492287	5.228063
H	-0.758873	-1.399618	4.191063
H	-2.161438	-0.775932	5.082006
C	-3.877638	-2.648714	3.802966
H	-4.077810	-2.390949	4.846131
H	-4.710501	-2.285582	3.201040
H	-3.821147	-3.741012	3.727931
C	-3.071984	-1.834700	0.937870
C	-2.730652	-0.987512	-0.153314
C	-4.566798	2.924984	0.491694
C	-4.113656	-2.777543	0.805694
C	-4.926346	-2.760185	-0.320836
C	-4.752910	-1.754716	-1.275133
C	-3.671016	-0.869591	-1.197693
H	-4.288633	-3.534829	1.558099
H	-5.721213	-3.491543	-0.430861
H	-5.460525	-1.662458	-2.095583
C	-3.530280	2.014198	-0.152813
O	-2.519586	1.689778	0.472128
C	-3.966904	1.648704	-1.567570
C	-5.883397	2.663051	-0.261887
C	-5.505320	1.820910	-1.482780
C	-3.571199	0.280509	-2.177037
H	-2.558895	0.358673	-2.575849
H	-4.254900	0.128868	-3.019634
C	-3.474256	2.802923	-2.501035
H	-3.817841	3.790413	-2.175467
H	-2.381350	2.812281	-2.538288
H	-3.865472	2.622044	-3.506798
O	-6.281733	1.362468	-2.285947
H	-6.601941	2.092271	0.336870
H	-6.398091	3.574430	-0.582576
H	-4.226651	3.962944	0.379816

H	-4.607666	2.724711	1.565564
H	-0.919861	0.655870	-1.450053
Ir	-1.003911	0.135756	0.039756
P	0.419034	-1.514194	-0.717077
P	0.799948	1.684568	0.609737
C	1.842307	-0.825012	-1.681925
C	2.898081	-0.138876	-0.997993
C	1.893910	-0.973418	-3.072967
C	3.908959	0.360971	-1.804311
C	2.936771	-0.453065	-3.859528
H	1.118515	-1.529002	-3.581859
C	3.932009	0.223915	-3.191417
H	2.957795	-0.586834	-4.935128
C	2.276120	0.850755	1.327806
C	3.062795	0.011607	0.480100
C	2.583593	0.975207	2.686455
C	4.123581	-0.635170	1.094306
C	3.661575	0.293703	3.280907
H	1.981460	1.623965	3.311353
C	4.413201	-0.511450	2.453383
H	3.894642	0.404622	4.334004
C	0.222349	2.856368	1.919056
C	0.519705	4.227705	1.895854
C	-0.564116	2.350766	2.970159
C	0.053519	5.071332	2.906831
H	1.114037	4.646178	1.091731
C	-1.016593	3.195864	3.983890
H	-0.825652	1.297790	2.990052
C	-0.710592	4.558442	3.955292
H	0.293091	6.130522	2.872967
H	-1.615339	2.789814	4.795114
H	-1.067584	5.215767	4.743285
C	1.520479	2.792359	-0.679371
C	2.766231	3.411529	-0.480039
C	0.809718	3.065844	-1.855870
C	3.278861	4.292260	-1.433502
H	3.339151	3.201502	0.418028
C	1.325899	3.942546	-2.812031
H	-0.142540	2.575625	-2.028043
C	2.560067	4.559701	-2.601714
H	4.238992	4.771352	-1.261406
H	0.766144	4.140426	-3.721991
H	2.960880	5.244174	-3.344086
C	-0.467659	-2.667227	-1.860647
C	-0.582905	-4.037693	-1.584486
C	-1.108508	-2.159538	-3.004631
C	-1.295307	-4.878996	-2.443290
H	-0.120391	-4.460044	-0.700533
C	-1.802819	-3.002805	-3.869815
H	-1.064042	-1.097859	-3.222447
C	-1.897909	-4.368484	-3.591680
H	-1.372314	-5.937264	-2.210327
H	-2.276756	-2.591504	-4.756708
H	-2.443386	-5.025977	-4.262521
C	1.298335	-2.650538	0.445754
C	1.073369	-2.636241	1.827047
C	2.240108	-3.551795	-0.084317
C	1.762572	-3.518237	2.663441
H	0.379574	-1.917637	2.241565
C	2.918183	-4.437380	0.751859
H	2.444899	-3.563180	-1.150618
C	2.678713	-4.424687	2.129449
H	1.584893	-3.492902	3.735126
H	3.633618	-5.136100	0.327050
H	3.206420	-5.115966	2.780831
O	5.021988	1.046153	-1.395864
O	5.050594	0.815143	-3.700505
O	5.036054	-1.468770	0.506467
O	5.510061	-1.263364	2.764181
C	5.730940	1.410740	-2.587082
H	5.722041	2.501267	-2.696967
H	6.753382	1.024624	-2.536478
C	5.870658	-1.959204	1.563419
H	5.693750	-3.032985	1.697394
H	6.918608	-1.755377	1.325254

TS-3^R

Zero-point correction= 0.887541 (Hartree/Particle)
Thermal correction to Energy= 0.945029
Thermal correction to Enthalpy= 0.945973
Thermal correction to Gibbs Free Energy= 0.794049
Sum of electronic and zero-point Energies= -3453.669821
Sum of electronic and thermal Energies= -3453.612333
Sum of electronic and thermal Enthalpies= -3453.611389
Sum of electronic and thermal Free Energies= -3453.763313
HF(M06/6-311++G(d,p) and SDD, SMD[1,4Dioxane])= -3453.12479977

C	-2.536654	-0.712669	2.260547
O	-1.525137	0.024373	2.084143
N	-3.015835	-0.890855	3.512124
C	-2.277549	-0.328577	4.646410
H	-2.363649	-1.013591	5.495049
H	-1.230106	-0.200180	4.381584
H	-2.692012	0.645100	4.936008
C	-4.341740	-1.412948	3.851452
H	-4.795695	-0.748007	4.593636
H	-4.986899	-1.440351	2.975506
H	-4.275672	-2.417365	4.285862
C	-3.164870	-1.329907	1.057783
C	-2.969440	-0.601933	-0.141041
C	-4.564850	1.699627	0.709682
C	-3.849232	-2.552413	1.032869
C	-4.408035	-3.004404	-0.167083
C	-4.334355	-2.227133	-1.327160

C	-3.623096	-1.025805	-1.309345
H	-3.931467	-3.170186	1.920986
H	-4.936086	-3.953322	-0.185830
H	-4.836849	-2.555219	-2.232890
C	-3.406577	1.428033	-0.265437
O	-2.285628	2.004243	0.014349
C	-3.992859	1.341343	-1.718483
C	-5.850865	1.260935	0.009382
C	-5.526082	1.329456	-1.481173
C	-3.631393	0.007454	-2.414501
H	-2.646036	0.086874	-2.884527
H	-4.362792	-0.214838	-3.198263
C	-3.600405	2.568749	-2.555451
H	-3.866819	3.505480	-2.055161
H	-2.521395	2.577068	-2.733242
H	-4.126524	2.534164	-3.514342
O	-6.338377	1.348464	-2.377441
H	-6.106692	0.217557	0.239646
H	-6.729821	1.868168	0.242377
H	-4.553198	2.788068	0.860954
H	-4.394088	1.251222	1.690162
H	-0.733071	0.617495	-1.655045
Ir	-0.933310	0.376804	-0.114636
P	0.352976	-1.526969	-0.501339
P	0.903674	1.807168	0.223917
C	1.928139	-1.187930	-1.422204
C	2.989118	-0.489956	-0.762417
C	2.104553	-1.635385	-2.737324
C	4.139590	-0.294045	-1.510818
C	3.283847	-1.413622	-3.470270
H	1.320621	-2.199699	-3.222378
C	4.289628	-0.729526	-2.825895
H	3.395209	-1.771605	-4.487458
C	2.233962	1.021634	1.220883
C	3.010614	-0.040082	0.660783
C	2.417470	1.404380	2.555429
C	3.924478	-0.631551	1.519156
C	3.348835	0.776761	3.400933
H	1.830498	2.219537	2.960012
C	4.086042	-0.249337	2.851055
H	3.485878	1.090575	4.429637
C	0.385210	3.294202	1.185878
C	0.879669	4.572184	0.881725
C	-0.514009	3.148214	2.256685
C	0.492187	5.678688	1.639855
H	1.566524	4.712782	0.055205
C	-0.888900	4.256248	3.016319
H	-0.923698	2.172141	2.489108
C	-0.389311	5.523931	2.709790
H	0.882177	6.661395	1.390508
H	-1.581126	4.130700	3.844687
H	-0.688513	6.386097	3.299239
C	1.733476	2.522640	-1.253870
C	3.084396	2.902749	-1.209676
C	0.985175	2.794672	-2.408609
C	3.674559	3.532582	-2.306630
H	3.679167	2.703591	-0.324037
C	1.578428	3.425254	-3.502967
H	-0.063479	2.519079	-2.447530
C	2.924451	3.793308	-3.455302
H	4.720404	3.823500	-2.259811
H	0.988010	3.630583	-4.391416
H	3.385619	4.283871	-4.307788
C	-0.562438	-2.731381	-1.569252
C	-0.854050	-4.037015	-1.148694
C	-1.011801	-2.321434	-2.838102
C	-1.548431	-4.916105	-1.984961
H	-0.537110	-4.380579	-0.170956
C	-1.686927	-3.205124	-3.679020
H	-0.820937	-1.307987	-3.176926
C	-1.955780	-4.509014	-3.254599
H	-1.759077	-5.925150	-1.641572
H	-2.004529	-2.874110	-4.663971
H	-2.480635	-5.199268	-3.908971
C	0.987558	-2.563074	0.893767
C	0.673480	-2.275862	2.227786
C	1.839035	-3.646996	0.611943
C	1.180532	-3.069674	3.260231
H	0.060541	-1.413455	2.455115
C	2.336148	-4.442689	1.643163
H	2.116677	-3.869210	-0.414113
C	2.004271	-4.158072	2.971426
H	0.935809	-2.832060	4.291968
H	2.984027	-5.282940	1.409294
H	2.390555	-4.779411	3.774805
O	5.276415	0.362070	-1.116445
O	5.518314	-0.371358	-3.298819
O	4.795614	-1.646242	1.227246
O	5.053754	-1.010675	3.438562
C	6.214193	0.215633	-2.191396
H	6.593362	1.199493	-2.480338
H	7.029640	-0.447908	-1.878631
C	5.444672	-1.983511	2.459660
H	5.116076	-2.978451	2.783351
H	6.529047	-1.946626	2.323694

I-4^{R}

Zero-point correction= 0.889204 (Hartree/Particle)

Thermal correction to Energy= 0.947368

Thermal correction to Enthalpy= 0.948312

Thermal correction to Gibbs Free Energy= 0.791425

Sum of electronic and zero-point Energies= -3453.700272

Sum of electronic and thermal Energies= -3453.642107

Sum of electronic and thermal Enthalpies= -3453.641163

Sum of electronic and thermal Free Energies=
-3453.798051
HF(M06/6-311++G(d,p) and SDD, SMD[1,4Dioxane])=
-3453.15043046

C	-2.954342	-1.431046	1.511090
O	-1.795974	-0.934860	1.454000
N	-3.406429	-1.923762	2.686802
C	-2.510704	-1.975571	3.841784
H	-2.860248	-2.760255	4.517546
H	-1.498055	-2.208819	3.517006
H	-2.503994	-1.021594	4.384059
C	-4.812390	-2.214192	2.982813
H	-5.120865	-1.628946	3.856810
H	-5.446891	-1.948322	2.140631
H	-4.946816	-3.277195	3.211927
C	-3.820128	-1.541914	0.302215
C	-4.094620	-0.433761	-0.515548
C	-3.780901	1.712705	0.955529
C	-4.323813	-2.804235	-0.061958
C	-5.091503	-2.958878	-1.215092
C	-5.384630	-1.852254	-2.016491
C	-4.886183	-0.599215	-1.662826
H	-4.101810	-3.668566	0.557688
H	-5.467990	-3.941853	-1.482492
H	-5.998366	-1.968470	-2.906098
C	-3.578249	1.008189	-0.411481
O	-2.260409	1.079591	-0.902204
C	-4.568733	1.756732	-1.381400
C	-5.267799	2.089066	1.016362
C	-5.718107	2.184089	-0.442935
C	-5.068671	0.708904	-2.394979
H	-4.463578	0.727751	-3.311438
H	-6.107934	0.901942	-2.683818
C	-3.991386	3.016248	-2.041029
H	-3.627905	3.738111	-1.300679
H	-3.154622	2.747696	-2.691696
H	-4.767527	3.507328	-2.637407
O	-6.815028	2.534605	-0.817738
H	-5.878088	1.312673	1.497355
H	-5.477295	3.023180	1.547617
H	-3.154223	2.610907	0.947399
H	-3.452049	1.110216	1.804586
H	-0.047623	0.521622	-1.669535
Ir	-0.650522	0.011832	-0.315389
P	0.949509	-1.655306	-0.484699
P	0.768204	1.540656	0.551136
C	2.329932	-1.061989	-1.539979
C	3.216371	-0.074227	-1.007580
C	2.414509	-1.446256	-2.880797
C	4.117115	0.468395	-1.909656
C	3.350081	-0.882595	-3.767935
H	1.737533	-2.200956	-3.263462
C	4.180753	0.087356	-3.251603
H	3.412547	-1.194540	-4.804250
C	2.378357	0.990097	1.241982
C	3.334586	0.290646	0.436462
C	2.663231	1.275709	2.585533
C	4.510022	-0.061647	1.081351
C	3.867046	0.902578	3.204888
H	1.939062	1.816260	3.180421
C	4.776582	0.227823	2.420693
H	4.068504	1.135565	4.244156
C	-0.077266	2.424736	1.924883
C	-0.115996	3.827142	1.969305
C	-0.677873	1.687956	2.959726
C	-0.741770	4.478872	3.034408
H	0.340774	4.413169	1.179976
C	-1.291308	2.344935	4.025312
H	-0.680977	0.604746	2.917807
C	-1.326918	3.741546	4.064344
H	-0.767055	5.564452	3.057296
H	-1.747211	1.767762	4.825403
H	-1.809585	4.251501	4.893134
C	1.164816	2.850689	-0.671869
C	2.415685	3.486476	-0.678123
C	0.160910	3.276781	-1.558992
C	2.661182	4.533338	-1.569662
H	3.198718	3.168373	0.001608
C	0.414983	4.326092	-2.441963
H	-0.810535	2.788762	-1.550395
C	1.664020	4.953196	-2.451634
H	3.631795	5.021550	-1.569194
H	-0.363704	4.652243	-3.125487
H	1.858069	5.767794	-3.143762
C	0.105102	-3.008777	-1.408093
C	0.260791	-4.363966	-1.077566
C	-0.773788	-2.658699	-2.451463
C	-0.437584	-5.345930	-1.782658
H	0.923689	-4.657979	-0.271490
C	-1.467478	-3.644112	-3.154022
H	-0.909289	-1.614315	-2.720717
C	-1.299945	-4.989968	-2.821140
H	-0.304450	-6.391302	-1.518764
H	-2.138757	-3.358158	-3.958435
H	-1.839272	-5.757829	-3.368487
C	1.758789	-2.482542	0.940963
C	1.200988	-2.366946	2.220721
C	2.921498	-3.250643	0.761989
C	1.791285	-3.015811	3.307084
H	0.310617	-1.762180	2.354294
C	3.504113	-3.903621	1.848656
H	3.374800	-3.332123	-0.221867
C	2.940012	-3.787329	3.122168
H	1.358218	-2.916384	4.298774
H	4.399177	-4.501600	1.701327
H	3.397970	-4.293139	3.967453
O	5.077503	1.410635	-1.656461
O	5.175264	0.778019	-3.881340
O	5.561370	-0.757945	0.550185
O	5.998024	-0.261635	2.768418

C	5.675452	1.719608	-2.924864
H	5.387577	2.734643	-3.224859
H	6.761437	1.620222	-2.849207
C	6.567811	-0.808196	1.569470
H	6.856653	-1.847835	1.745994
H	7.429440	-0.201842	1.266832

I-4'^{R}

Zero-point correction= 0.888523 (Hartree/Particle)
Thermal correction to Energy= 0.946487
Thermal correction to Enthalpy= 0.947431
Thermal correction to Gibbs Free Energy= 0.792663
Sum of electronic and zero-point Energies= -3453.709322
Sum of electronic and thermal Energies= -3453.651358
Sum of electronic and thermal Enthalpies= -3453.650414
Sum of electronic and thermal Free Energies= -3453.805182
HF(M06/6-311++G(d,p) and SDD, SMD[1,4Dioxane])= -3453.16021608

C	-3.148925	2.143830	-0.340359
O	-2.057099	1.731168	-0.808748
N	-3.694408	3.269546	-0.847536
C	-2.942453	4.050508	-1.833646
H	-3.184272	5.108015	-1.696198
H	-1.875294	3.896896	-1.687254
H	-3.213902	3.754308	-2.854466
C	-5.076132	3.701959	-0.635921
H	-5.569321	3.814782	-1.608432
H	-5.625978	2.972151	-0.045181
H	-5.101273	4.669938	-0.122818
C	-3.829970	1.433686	0.785447
C	-4.102019	0.057023	0.733241
H	-2.246366	-1.945070	-1.039981
C	-4.369768	-0.535709	-1.800936
Ir	-0.650092	0.155898	-0.318781
C	-4.139540	2.136791	1.960329
C	-4.700391	1.482993	3.059345
C	-4.977556	0.116662	3.001397
C	-4.679923	-0.588672	1.834944
H	-3.922396	3.198954	2.016813
H	-4.926841	2.045956	3.960072
H	-5.425626	-0.389419	3.852009
C	-3.853921	-0.929798	-0.398269
O	-2.431693	-1.229240	-0.402694
C	-4.720627	-2.161065	0.032440
C	-5.880992	-0.806501	-1.758274
C	-6.064494	-1.884774	-0.688004
C	-4.899425	-2.056540	1.560771
H	-4.154095	-2.666922	2.087929
H	-5.889330	-2.413664	1.864891
C	-4.203473	-3.531257	-0.430639
H	-4.062308	-3.583389	-1.517060
H	-3.251853	-3.775737	0.052324
H	-4.933684	-4.300307	-0.160303
O	-7.100968	-2.448642	-0.425223
H	-6.451176	0.075661	-1.438832
H	-6.308569	-1.127727	-2.712824
H	-3.890252	-1.193184	-2.537328
H	-4.109328	0.486986	-2.079775
P	0.711597	-1.628055	-0.165607
P	1.015324	1.639642	-0.081155
C	2.379981	-1.491081	-0.948490
C	3.353511	-0.616511	-0.376118
C	2.675203	-2.194486	-2.122938
C	4.554464	-0.518386	-1.061498
C	3.905401	-2.074282	-2.792932
H	1.942096	-2.871047	-2.542088
C	4.828349	-1.216979	-2.236029
H	4.115708	-2.629374	-3.700103
C	2.312509	1.204911	1.164347
C	3.214255	0.125407	0.909373
C	2.358984	1.877428	2.391945
C	4.078652	-0.197898	1.944174
C	3.253923	1.529359	3.418684
H	1.687891	2.706594	2.572945
C	4.099461	0.472081	3.166191
H	3.276704	2.067260	4.359700
C	0.355143	3.263887	0.521511
C	0.725031	4.491934	-0.045080
C	-0.552328	3.261647	1.595906
C	0.207494	5.690308	0.456541
H	1.420021	4.521921	-0.876867
C	-1.054482	4.459376	2.105269
H	-0.857421	2.314846	2.033777
C	-0.677184	5.678733	1.534723
H	0.506440	6.632824	0.006180
H	-1.734901	4.441613	2.952804
H	-1.067187	6.611845	1.931838
C	1.960292	2.087014	-1.599117
C	3.219532	2.705268	-1.534117
C	1.379139	1.845036	-2.852474
C	3.879607	3.080287	-2.705251
H	3.689123	2.885165	-0.571323
C	2.042699	2.220266	-4.022190
H	0.412728	1.349444	-2.896287
C	3.292226	2.839344	-3.950229
H	4.852422	3.560809	-2.645172
H	1.586620	2.025286	-4.988978
H	3.809167	3.130094	-4.860570
C	-0.066300	-3.050662	-1.078874
C	-0.161084	-4.347025	-0.551759
C	-0.596570	-2.804995	-2.362809
C	-0.757220	-5.373135	-1.291280
H	0.234304	-4.562610	0.434803
C	-1.187911	-3.833843	-3.101184

H -0.522426 -1.804837 -2.784938
C -1.268671 -5.122249 -2.565516
H -0.818084 -6.371873 -0.868059
H -1.574703 -3.630321 -4.096159
H -1.725047 -5.924189 -3.138621
C 1.056084 -2.310783 1.511539
C 0.230991 -1.924413 2.578023
C 2.101001 -3.219729 1.745315
C 0.440322 -2.445931 3.856215
H -0.559013 -1.200588 2.394659
C 2.305343 -3.741873 3.023518
H 2.760158 -3.512343 0.932933
C 1.475101 -3.356440 4.079967
H -0.200016 -2.136930 4.677836
H 3.112180 -4.449627 3.194202
H 1.637775 -3.762106 5.074709
O 5.629102 0.266125 -0.735717
O 6.079668 -0.904534 -2.683094
O 5.029004 -1.185129 1.948911
O 5.057933 -0.065839 3.976320
C 6.646479 -0.025082 -1.702582
H 6.964537 0.902882 -2.186997
H 7.489752 -0.523077 -1.209437
C 5.593523 -1.190136 3.266623
H 5.308659 -2.115736 3.781612
H 6.680885 -1.093225 3.198709

TS-4^{R}

Zero-point correction= 0.886018 (Hartree/Particle)
Thermal correction to Energy= 0.943985
Thermal correction to Enthalpy= 0.944929
Thermal correction to Gibbs Free Energy= 0.789697
Sum of electronic and zero-point Energies= -3453.676264
Sum of electronic and thermal Energies= -3453.618297
Sum of electronic and thermal Enthalpies= -3453.617353
Sum of electronic and thermal Free Energies= -3453.772585
HF(M06/6-311++G(d,p) and SDD, SMD[1,4Dioxane])= -3453.12707855

C -3.149301 2.077271 -0.587024
O -2.015693 1.641229 -0.936571
N -3.661354 3.129164 -1.256915
C -2.844756 3.799274 -2.272456
H -3.160917 4.843668 -2.336478
H -1.793444 3.752127 -1.993148
H -2.975986 3.328360 -3.254557
C -5.066156 3.542819 -1.216884
H -5.467726 3.535684 -2.236582
H -5.652447 2.862518 -0.603154
H -5.156719 4.558270 -0.815701
C -3.894896 1.491400 0.561997
C -4.105336 0.106805 0.677779
H -1.608799 -0.637025 0.868415
C -4.093351 -0.851315 -1.755176
Ir -0.682870 0.066374 -0.305733
C -4.339414 2.337857 1.593280
C -4.977425 1.817207 2.718915
C -5.197066 0.442388 2.828999
C -4.764808 -0.403163 1.807486
H -4.162971 3.406491 1.519698
H -5.310645 2.487208 3.506195
H -5.709503 0.037470 3.697628
C -3.693523 -1.026531 -0.267885
O -2.316953 -1.358372 -0.133415
C -4.598652 -2.205761 0.245785
C -5.596846 -1.155304 -1.819589
C -5.874526 -2.056155 -0.613310
C -4.916120 -1.903093 1.722854
H -4.204145 -2.406352 2.391046
H -5.919510 -2.251833 1.990921
C -4.022983 -3.610140 0.016658
H -3.808590 -3.801363 -1.039968
H -3.088115 -3.731301 0.569554
H -4.745944 -4.358798 0.357187
O -6.937807 -2.573566 -0.354493
H -6.213524 -0.253859 -1.704491
H -5.920965 -1.638296 -2.746933
H -3.526722 -1.596380 -2.323693
H -3.826475 0.128070 -2.160068
P 0.740543 -1.712522 -0.165629
P 0.929003 1.650509 0.110029
C 2.312547 -1.396974 -1.081414
C 3.284500 -0.493838 -0.546722
C 2.524410 -1.996640 -2.329353
C 4.399862 -0.271950 -1.338744
C 3.667045 -1.746630 -3.109080
H 1.793595 -2.694437 -2.716926
C 4.590566 -0.869892 -2.584148
H 3.814477 -2.224206 -4.071045
C 2.309672 1.151954 1.218276
C 3.237708 0.149501 0.797921
C 2.418297 1.723543 2.492314
C 4.211190 -0.193159 1.722986
C 3.416343 1.349174 3.408287
H 1.716849 2.489695 2.796240
C 4.300257 0.377907 2.992291
H 3.487006 1.803371 4.390031
C 0.179227 3.116952 0.943849
C 0.512794 4.432648 0.589077
C -0.733097 2.904468 1.991252
C -0.048321 5.512935 1.274647
H 1.212621 4.620466 -0.218004
C -1.280204 3.985570 2.683283
H -1.010097 1.890325 2.264204
C -0.939870 5.292857 2.325536

H 0.219589 6.526439 0.989537
H -1.973295 3.804682 3.500009
H -1.365903 6.134497 2.864477
C 1.733831 2.338079 -1.395997
C 2.991224 2.960561 -1.348723
C 1.050003 2.266173 -2.620110
C 3.548578 3.506914 -2.506064
H 3.539880 3.009852 -0.413066
C 1.612405 2.810423 -3.775958
H 0.077108 1.784390 -2.666378
C 2.861375 3.432612 -3.720120
H 4.520288 3.990718 -2.458272
H 1.078020 2.744701 -4.719573
H 3.299246 3.855415 -4.619896
C -0.002703 -3.180057 -1.004484
C 0.054544 -4.470137 -0.460591
C -0.644273 -2.993809 -2.241891
C -0.505102 -5.552477 -1.145856
H 0.529713 -4.640778 0.498614
C -1.187576 -4.076743 -2.930715
H -0.719692 -1.996470 -2.665360
C -1.120300 -5.361134 -2.382262
H -0.455421 -6.545672 -0.708762
H -1.667670 -3.918420 -3.892564
H -1.548213 -6.205022 -2.915885
C 1.273258 -2.329252 1.482583
C 0.551779 -1.989419 2.635190
C 2.382200 -3.185275 1.596451
C 0.925255 -2.501563 3.879203
H -0.293136 -1.312450 2.558500
C 2.749910 -3.699359 2.840020
H 2.961724 -3.445602 0.715563
C 2.021701 -3.358933 3.983394
H 0.360478 -2.228576 4.766071
H 3.603284 -4.367818 2.915008
H 2.309705 -3.759353 4.951315
O 5.455996 0.555881 -1.063696
O 5.767429 -0.446111 -3.128277
O 5.203877 -1.122859 1.569652
O 5.347675 -0.165647 3.674848
C 6.388062 0.387504 -2.140712
H 6.616677 1.362755 -2.579809
H 7.295488 -0.101893 -1.767475
C 5.942025 -1.133775 2.799449
H 5.874693 -2.126880 3.256079
H 6.983403 -0.857533 2.603989

I-5*^R^*

Zero-point correction= 0.891829 (Hartree/Particle)
Thermal correction to Energy= 0.950001
Thermal correction to Enthalpy= 0.950945
Thermal correction to Gibbs Free Energy= 0.795320
Sum of electronic and zero-point Energies= -3453.707993
Sum of electronic and thermal Energies= -3453.649820
Sum of electronic and thermal Enthalpies= -3453.648876
Sum of electronic and thermal Free Energies= -3453.804501
HF(M06/6-311++G(d,p) and SDD, SMD[1,4Dioxane])= -3453.17283660

C -3.148925 2.143830 -0.340359
O -2.057099 1.731168 -0.808748
N -3.694408 3.269546 -0.847536
C -2.942453 4.050508 -1.833646
H -3.184272 5.108015 -1.696198
H -1.875294 3.896896 -1.687254
H -3.213902 3.754308 -2.854466
C -5.076132 3.701959 -0.635921
H -5.569321 3.814782 -1.608432
H -5.625978 2.972151 -0.045181
H -5.101273 4.669938 -0.122818
C -3.829970 1.433686 0.785447
C -4.102019 0.057023 0.733241
H -2.246366 -1.945070 -1.039981
C -4.369768 -0.535709 -1.800936
Ir -0.650092 0.155898 -0.318781
C -4.139540 2.136791 1.960329
C -4.700391 1.482993 3.059345
C -4.977556 0.116662 3.001397
C -4.679923 -0.588672 1.834944
H -3.922396 3.198954 2.016813
H -4.926841 2.045956 3.960072
H -5.425626 -0.389419 3.852009
C -3.853921 -0.929798 -0.398269
O -2.431693 -1.229240 -0.402694
C -4.720627 -2.161065 0.032440
C -5.880992 -0.806501 -1.758274
C -6.064494 -1.884774 -0.688004
C -4.899425 -2.056540 1.560771
H -4.154095 -2.666922 2.087929
H -5.889330 -2.413664 1.864891
C -4.203473 -3.531257 -0.430639
H -4.062308 -3.583389 -1.517060
H -3.251853 -3.775737 0.052324
H -4.933684 -4.300307 -0.160303
O -7.100968 -2.448642 -0.425223
H -6.451176 0.075661 -1.438832
H -6.308569 -1.127727 -2.712824
H -3.890252 -1.193184 -2.537328
H -4.109328 0.486986 -2.079775
P 0.711597 -1.628055 -0.165607
P 1.015324 1.639642 -0.081155
C 2.379981 -1.491081 -0.948490
C 3.353511 -0.616511 -0.376118
C 2.675203 -2.194486 -2.122938

C	4.554464	-0.518386	-1.061498
C	3.905401	-2.074282	-2.792932
H	1.942096	-2.871047	-2.542088
C	4.828349	-1.216979	-2.236029
H	4.115708	-2.629374	-3.700103
C	2.312509	1.204911	1.164347
C	3.214255	0.125407	0.909373
C	2.358984	1.877428	2.391945
C	4.078652	-0.197898	1.944174
C	3.253923	1.529359	3.418684
H	1.687891	2.706594	2.572945
C	4.099461	0.472081	3.166191
H	3.276704	2.067260	4.359700
C	0.355143	3.263887	0.521511
C	0.725031	4.491934	-0.045080
C	-0.552328	3.261647	1.595906
C	0.207494	5.690308	0.456541
H	1.420021	4.521921	-0.876867
C	-1.054482	4.459376	2.105269
H	-0.857421	2.314846	2.033777
C	-0.677184	5.678733	1.534723
H	0.506440	6.632824	0.006180
H	-1.734901	4.441613	2.952804
H	-1.067187	6.611845	1.931838
C	1.960292	2.087014	-1.599117
C	3.219532	2.705268	-1.534117
C	1.379139	1.845036	-2.852474
C	3.879607	3.080287	-2.705251
H	3.689123	2.885165	-0.571323
C	2.042699	2.220266	-4.022190
H	0.412728	1.349444	-2.896287
C	3.292226	2.839344	-3.950229
H	4.852422	3.560809	-2.645172
H	1.586620	2.025286	-4.988978
H	3.809167	3.130094	-4.860570
C	-0.066300	-3.050662	-1.078874
C	-0.161084	-4.347025	-0.551759
C	-0.596570	-2.804995	-2.362809
C	-0.757220	-5.373135	-1.291280
H	0.234304	-4.562610	0.434803
C	-1.187911	-3.833843	-3.101184
H	-0.522426	-1.804837	-2.784938
C	-1.268671	-5.122249	-2.565516
H	-0.818084	-6.371873	-0.868059
H	-1.574703	-3.630321	-4.096159
H	-1.725047	-5.924189	-3.138621
C	1.056084	-2.310783	1.511539
C	0.230991	-1.924413	2.578023
C	2.101001	-3.219729	1.745315
C	0.440322	-2.445931	3.856215
H	-0.559013	-1.200588	2.394659
C	2.305343	-3.741873	3.023518
H	2.760158	-3.512343	0.932933
C	1.475101	-3.356440	4.079967
H	-0.200016	-2.136930	4.677836
H	3.112180	-4.449627	3.194202
H	1.637775	-3.762106	5.074709
O	5.629102	0.266125	-0.735717
O	6.079668	-0.904534	-2.683094
O	5.029004	-1.185129	1.948911
O	5.057933	-0.065839	3.976320
C	6.646479	-0.025082	-1.702582
H	6.964537	0.902882	-2.186997
H	7.489752	-0.523077	-1.209437
C	5.593523	-1.190136	3.266623
H	5.308659	-2.115736	3.781612
H	6.680885	-1.093225	3.198709

I-3^{S}

Zero-point correction= 0.888955 (Hartree/Particle)
Thermal correction to Energy= 0.947104
Thermal correction to Enthalpy= 0.948049
Thermal correction to Gibbs Free Energy= 0.792765
Sum of electronic and zero-point Energies= -3453.700102
Sum of electronic and thermal Energies= -3453.641953
Sum of electronic and thermal Enthalpies= -3453.641009
Sum of electronic and thermal Free Energies= -3453.796292
HF(M06/6-311++G(d,p) and SDD, SMD[1,4Dioxane])= -3453.14741690

C	1.771398	-1.727829	2.262875
O	0.893367	-0.853307	1.987069
N	1.753442	-2.245842	3.517656
C	0.657346	-1.851874	4.410510
H	0.460161	-2.676043	5.101718
H	0.922560	-0.958080	4.989845
H	-0.236742	-1.638019	3.829862
C	2.862742	-2.936406	4.179194
H	2.821244	-2.696532	5.244736
H	2.795680	-4.025914	4.074086
H	3.824461	-2.594063	3.796549
C	2.771573	-2.055764	1.215461
C	2.703489	-1.201450	0.079180
H	1.277748	0.549384	-1.526152
C	4.047190	2.469050	-2.187620
H	4.378655	3.451991	-1.837059
H	4.598447	2.229102	-3.102004
C	4.311147	1.330621	-1.147911
Ir	1.043656	0.024299	-0.057621
C	3.771781	-3.045323	1.298638
C	4.796279	-3.082155	0.359502
C	4.864008	-2.089956	-0.620859
C	3.832329	-1.153242	-0.765406
H	3.756212	-3.799345	2.074006
H	5.562124	-3.849518	0.419061

H	5.720244	-2.048466	-1.289831
C	3.965309	-0.040007	-1.780710
H	4.782950	-0.251348	-2.478540
H	3.045137	0.057994	-2.360632
C	3.661660	1.764212	0.161756
C	5.819517	1.441792	-0.803684
O	6.698967	0.907016	-1.434750
O	2.554818	1.489905	0.631014
C	4.611199	2.668422	0.936069
H	4.330133	3.710016	0.732032
H	4.470279	2.509840	2.008574
C	6.023566	2.336892	0.420920
H	6.609922	3.218779	0.143461
H	6.619072	1.783257	1.155068
P	-0.673735	1.766640	0.187794
P	-0.485923	-1.502451	-0.836810
C	-2.322839	1.612986	-0.632410
C	-3.134222	0.462962	-0.383894
C	-2.788326	2.624791	-1.482261
C	-4.358330	0.436505	-1.032677
C	-4.033946	2.563634	-2.130749
H	-2.179395	3.503640	-1.648841
C	-4.800329	1.445710	-1.886110
H	-4.372797	3.357444	-2.786726
C	-1.811514	-1.640591	0.433910
C	-2.828109	-0.644112	0.569494
C	-1.684983	-2.646397	1.402622
C	-3.651893	-0.773610	1.679042
C	-2.543679	-2.754719	2.508870
H	-0.893851	-3.380001	1.305527
C	-3.521347	-1.789678	2.623761
H	-2.441446	-3.553704	3.234645
C	0.045195	-3.240863	-1.200622
C	-0.857593	-4.317400	-1.130710
C	1.318310	-3.467433	-1.741897
C	-0.477591	-5.590456	-1.554875
H	-1.860368	-4.171006	-0.745157
C	1.694201	-4.741876	-2.170116
H	2.021781	-2.650096	-1.830444
C	0.801052	-5.808393	-2.071794
H	-1.186267	-6.410958	-1.485305
H	2.688264	-4.895657	-2.580041
H	1.094321	-6.800734	-2.402380
C	-1.301586	-1.124707	-2.452671
C	-2.528770	-1.731966	-2.766305
C	-0.644875	-0.377411	-3.438418
C	-3.089712	-1.579057	-4.032988
H	-3.058478	-2.315469	-2.019688
C	-1.207371	-0.228606	-4.708427
H	0.310671	0.083895	-3.215172
C	-2.430432	-0.827297	-5.008815
H	-4.041099	-2.052338	-4.259527
H	-0.684907	0.353264	-5.462703
H	-2.866916	-0.713155	-5.996923
C	-0.010197	3.369602	-0.455610
C	0.125253	4.504105	0.356651
C	0.384219	3.452497	-1.803419
C	0.643858	5.693275	-0.165449
H	-0.177341	4.468209	1.397302
C	0.881818	4.645637	-2.326943
H	0.280958	2.585636	-2.449693
C	1.018665	5.769552	-1.506448
H	0.740972	6.562811	0.478715
H	1.162037	4.698996	-3.375589
H	1.408836	6.698054	-1.913684
C	-1.094376	2.147986	1.944620
C	-0.105241	2.000146	2.930735
C	-2.357930	2.635693	2.313962
C	-0.375957	2.338782	4.257438
H	0.873103	1.619548	2.660891
C	-2.626386	2.966145	3.643832
H	-3.134368	2.758183	1.566379
C	-1.636728	2.819453	4.617928
H	0.399298	2.226552	5.010799
H	-3.607011	3.348132	3.914727
H	-1.845525	3.081938	5.651395
H	2.979296	2.527470	-2.409561
O	-5.293141	-0.566076	-0.990800
O	-6.024415	1.120278	-2.395726
O	-4.707192	0.031453	2.020925
O	-4.480154	-1.659760	3.585296
C	-6.429934	-0.072516	-1.711849
H	-6.750548	-0.819789	-2.441772
H	-7.236544	0.166308	-1.007060
C	-5.161160	-0.429531	3.299859
H	-4.907835	0.313115	4.066578
H	-6.238335	-0.611302	3.261714

TS-3^{S}

Zero-point correction= 0.887686 (Hartree/Particle)
Thermal correction to Energy= 0.945119
Thermal correction to Enthalpy= 0.946064
Thermal correction to Gibbs Free Energy= 0.794197
Sum of electronic and zero-point Energies= -3453.665484
Sum of electronic and thermal Energies= -3453.608051
Sum of electronic and thermal Enthalpies= -3453.607107
Sum of electronic and thermal Free Energies - -3453.756218
HF(M06/6-311++G(d,p) and SDD, SMD[1,4Dioxane])= -3453.12222314

C	2.82654	-0.65431	2.09529
O	1.74778	-0.00139	2.05769
N	3.47709	-0.78535	3.27327
C	2.84039	-0.25959	4.48445
H	1.75975	-0.37826	4.41700

H	3.21655	-0.81846	5.34521
H	3.06873	0.80454	4.62080
C	4.87878	-1.17888	3.43311
H	5.39405	-1.18414	2.47460
H	5.37339	-0.45135	4.08524
H	4.96417	-2.16933	3.89534
C	3.33719	-1.23860	0.81934
C	2.93070	-0.54272	-0.34238
H	0.51491	0.64289	-1.55122
C	3.31865	2.65025	-2.79592
H	3.58366	3.57893	-2.28015
H	3.81804	2.64543	-3.76985
C	3.75630	1.40728	-2.00541
Ir	0.89780	0.38845	-0.04875
C	4.08924	-2.41559	0.70827
C	4.47231	-2.87172	-0.55600
C	4.17612	-2.12848	-1.70464
C	3.43287	-0.95312	-1.59123
H	4.33988	-3.00647	1.58190
H	5.04151	-3.79282	-0.64121
H	4.55297	-2.45054	-2.67207
C	3.29877	0.09177	-2.67714
H	3.92768	-0.12004	-3.54781
H	2.26512	0.19253	-3.02293
C	3.32835	1.48456	-0.49335
C	5.30761	1.36624	-1.92871
O	6.01877	1.35152	-2.90717
O	2.23523	2.03216	-0.07774
C	4.58483	1.78201	0.34112
H	4.58570	2.87453	0.46255
H	4.51978	1.36314	1.34685
C	5.78901	1.32538	-0.48110
H	6.68858	1.93434	-0.35576
H	6.06712	0.28600	-0.25849
P	-0.94851	1.80881	0.33707
P	-0.37705	-1.57116	-0.15184
C	-2.27462	1.50888	-0.90410
C	-3.00362	0.28086	-0.85922
C	-2.46926	2.40452	-1.96160
C	-3.85634	0.04786	-1.92702
C	-3.34820	2.14044	-3.02694
H	-1.93271	3.34437	-1.97104
C	-4.01720	0.93690	-2.98987
H	-3.49420	2.85084	-3.83277
C	-2.01578	-1.56302	0.72239
C	-3.04604	-0.66976	0.29118
C	-2.27411	-2.45901	1.76924
C	-4.24440	-0.74867	0.98446
C	-3.50152	-2.51216	2.45206
H	-1.51761	-3.17060	2.06591
C	-4.47441	-1.63118	2.03769
H	-3.67199	-3.21759	3.25738
C	0.49089	-3.04571	0.55985
C	0.85364	-4.13948	-0.23896
C	0.81432	-3.07764	1.92927

C	1.50492	-5.24335	0.31898
H	0.62328	-4.14233	-1.29803
C	1.44713	-4.18810	2.48746
H	0.55328	-2.23733	2.56364
C	1.79468	-5.27653	1.68261
H	1.77145	-6.08348	-0.31618
H	1.66361	-4.20558	3.55236
H	2.28347	-6.14400	2.11727
C	-0.86154	-2.15479	-1.83920
C	-1.90093	-3.09146	-1.97677
C	-0.17675	-1.73440	-2.98662
C	-2.24339	-3.58935	-3.23401
H	-2.44648	-3.43180	-1.10273
C	-0.52231	-2.23049	-4.24539
H	0.62417	-1.01259	-2.89507
C	-1.55655	-3.15880	-4.37228
H	-3.04656	-4.31572	-3.32229
H	0.01655	-1.88942	-5.12488
H	-1.82416	-3.54718	-5.35100
C	-0.45303	3.57275	0.14268
C	0.24430	3.97586	-1.00932
C	-0.73560	4.52999	1.12953
C	0.61800	5.30706	-1.18510
H	0.49400	3.24475	-1.76830
C	-0.34611	5.85990	0.95715
H	-1.26174	4.24739	2.03357
C	0.32516	6.25327	-0.20042
H	1.14553	5.60315	-2.08760
H	-0.57484	6.58807	1.73012
H	0.62127	7.28984	-0.33456
C	-1.77321	1.77680	1.97814
C	-3.09427	2.23010	2.12655
C	-1.04574	1.38572	3.11116
C	-3.67771	2.28709	3.39224
H	-3.66791	2.53318	1.25584
C	-1.63630	1.44564	4.37572
H	-0.02347	1.03925	3.00155
C	-2.95012	1.89449	4.51890
H	-4.69871	2.64311	3.49909
H	-1.06832	1.14294	5.25138
H	-3.40610	1.94084	5.50389
H	2.23680	2.64242	-2.95146
O	-4.68273	-1.02989	-2.10621
O	-4.93263	0.44222	-3.87364
O	-5.36145	0.01991	0.78463
O	-5.73511	-1.45651	2.52724
C	-5.24688	-0.87902	-3.41577
H	-4.79745	-1.61715	-4.09225
H	-6.33196	-0.99637	-3.36108
C	-6.36825	-0.50690	1.65810
H	-6.79422	0.30410	2.25435
H	-7.14081	-1.01272	1.06598

I-4^{S}

Zero-point correction= 0.889447 (Hartree/Particle)

Thermal correction to Energy= 0.947193
Thermal correction to Enthalpy= 0.948137
Thermal correction to Gibbs Free Energy= 0.794333
Sum of electronic and zero-point Energies= -3453.698504
Sum of electronic and thermal Energies= -3453.640757
Sum of electronic and thermal Enthalpies= -3453.639813
Sum of electronic and thermal Free Energies= -3453.793617
HF(M06/6-311++G(d,p) and SDD, SMD[1,4Dioxane])= -3453.14807440

C	-2.498641	0.931664	1.966417
O	-1.472535	0.433725	1.434250
N	-2.532931	1.072106	3.313764
C	-1.330825	0.800553	4.103588
H	-1.282603	1.524008	4.923872
H	-1.357836	-0.210085	4.526942
H	-0.447619	0.892427	3.474715
C	-3.746384	1.286385	4.104409
H	-3.818064	0.497038	4.861793
H	-3.711921	2.253586	4.619100
H	-4.632756	1.249230	3.474800
C	-3.646430	1.437033	1.154998
C	-4.191681	0.702515	0.087001
H	-0.383913	-0.421985	-2.149064
C	-4.676999	-1.938317	-2.560979
H	-4.234801	-2.885012	-2.229451
H	-5.588474	-2.169991	-3.121609
C	-5.019814	-1.021838	-1.378361
Ir	-0.765419	-0.088814	-0.662183
C	-4.134954	2.733261	1.402672
C	-5.144802	3.283591	0.614120
C	-5.688175	2.549794	-0.442735
C	-5.206574	1.266527	-0.701449
H	-3.704539	3.322265	2.206829
H	-5.506020	4.285744	0.826104
H	-6.479589	2.973771	-1.055271
C	-5.637762	0.325308	-1.800804
H	-6.725709	0.247016	-1.903707
H	-5.239443	0.655757	-2.769624
C	-3.801925	-0.682200	-0.440700
C	-5.988227	-1.777532	-0.443024
O	-7.156201	-1.982092	-0.686651
O	-2.603895	-0.603802	-1.185784
C	-3.791872	-1.824480	0.610610
H	-3.228516	-2.657985	0.176946
H	-3.287761	-1.536703	1.536363
C	-5.260101	-2.214494	0.829592
H	-5.419761	-3.280513	1.020451
H	-5.715788	-1.673844	1.669730
P	0.780756	-1.681306	-0.147068
P	0.866518	1.551234	-0.602001
C	2.499766	-1.426576	-0.745792
C	3.356768	-0.432214	-0.170214
C	2.951210	-2.210675	-1.815871
C	4.630601	-0.355801	-0.711020
C	4.242496	-2.089241	-2.355054
H	2.290646	-2.951521	-2.248214
C	5.066489	-1.152540	-1.770592
H	4.571925	-2.701366	-3.186679
C	1.961682	1.485762	0.870406
C	2.997369	0.501331	0.935656
C	1.710208	2.322292	1.964500
C	3.736497	0.486370	2.108714
C	2.467629	2.267358	3.148161
H	0.914850	3.055189	1.900821
C	3.484350	1.337489	3.185289
H	2.268095	2.927288	3.984897
C	0.052163	3.208561	-0.577016
C	0.798274	4.383331	-0.371872
C	-1.305100	3.317546	-0.906909
C	0.190407	5.632845	-0.472665
H	1.856420	4.326419	-0.137824
C	-1.910121	4.572336	-1.018183
H	-1.897963	2.426771	-1.093990
C	-1.165957	5.730143	-0.797115
H	0.778245	6.531347	-0.307947
H	-2.962502	4.636188	-1.276657
H	-1.635226	6.705935	-0.884738
C	1.974544	1.745910	-2.065399
C	3.215279	2.392530	-1.943302
C	1.519660	1.380682	-3.340500
C	3.985515	2.660060	-3.074898
H	3.592849	2.675708	-0.965702
C	2.294233	1.649487	-4.470504
H	0.558143	0.891536	-3.454579
C	3.527441	2.289279	-4.341239
H	4.942597	3.163064	-2.966311
H	1.929217	1.359762	-5.451685
H	4.127644	2.500253	-5.221672
C	0.233924	-3.279463	-0.901012
C	0.837579	-4.475314	-0.468493
C	-0.766384	-3.336753	-1.882878
C	0.459371	-5.697142	-1.021065
H	1.603381	-4.455509	0.300324
C	-1.144497	-4.566793	-2.428892
H	-1.270827	-2.432728	-2.202359
C	-0.532762	-5.745786	-2.004271
H	0.936200	-6.611370	-0.679491
H	-1.921504	-4.597102	-3.187412
H	-0.829339	-6.699487	-2.431447
C	0.922120	-2.151796	1.629513
C	-0.258600	-2.353419	2.361143
C	2.153104	-2.470764	2.222822
C	-0.208481	-2.856929	3.660325
H	-1.216345	-2.128143	1.908587
C	2.199133	-2.966576	3.527923

H 3.079156 -2.347462 1.673823
C 1.021331 -3.160600 4.249830
H -1.131528 -3.024492 4.208907
H 3.159359 -3.213212 3.972198
H 1.059164 -3.557036 5.260494
H -3.960348 -1.445208 -3.223171
O 5.623012 0.520874 -0.363320
O 6.343798 -0.820054 -2.106586
O 4.763377 -0.361488 2.431115
O 4.349104 1.065023 4.204683
C 6.765790 0.165957 -1.155311
H 7.130330 1.050029 -1.684928
H 7.542671 -0.259299 -0.508880
C 5.274970 0.098987 3.690382
H 5.346178 -0.742200 4.383931
H 6.251990 0.573609 3.537239

I-4'^{S}

Zero-point correction= 0.888961 (Hartree/Particle)
Thermal correction to Energy= 0.946849
Thermal correction to Enthalpy= 0.947793
Thermal correction to Gibbs Free Energy= 0.793303
Sum of electronic and zero-point Energies= -3453.716030
Sum of electronic and thermal Energies= -3453.658142
Sum of electronic and thermal Enthalpies= -3453.657198
Sum of electronic and thermal Free Energies= -3453.811687
HF(M06/6-311++G(d,p) and SDD, SMD[1,4Dioxane])= -3453.16513610

C 3.086685 2.238963 0.555165
O 1.955593 2.022146 0.031740
N 3.571123 3.497523 0.525593
C 2.732210 4.582226 0.008351
H 3.010838 5.508043 0.519255
H 2.878560 4.711385 -1.070263
H 1.682640 4.360384 0.190274
C 4.972918 3.857949 0.753296
H 5.347243 4.394913 -0.125724
H 5.065347 4.515088 1.625150
H 5.581436 2.969922 0.908685
C 3.862676 1.158933 1.221998
C 4.060364 -0.084028 0.599131
H 0.244032 0.578474 1.538850
C 3.736049 -2.971197 -1.800797
H 3.436699 -2.609379 -2.791466
H 4.434617 -3.801811 -1.946019
C 4.410626 -1.873154 -0.967349
Ir 0.684768 0.249133 0.063807
C 4.351360 1.355238 2.526288
C 5.031993 0.335893 3.191724
C 5.247966 -0.892528 2.561790
C 4.759634 -1.096980 1.271065
H 4.186633 2.306512 3.024309
H 5.399453 0.504296 4.199771
H 5.792554 -1.681134 3.074652
C 4.850615 -2.348676 0.432160
H 5.857574 -2.779879 0.403623
H 4.171285 -3.118698 0.819903
C 3.537125 -0.580650 -0.751148
C 5.630219 -1.345016 -1.754025
O 6.670233 -1.949578 -1.892378
O 2.161769 -0.896226 -0.646479
C 3.858703 0.305635 -1.984638
H 3.216425 -0.047760 -2.799816
H 3.624002 1.361476 -1.831524
C 5.334227 0.047149 -2.316260
H 5.575557 0.094384 -3.382975
H 6.006103 0.754496 -1.811583
P -0.521851 -1.693193 0.295714
P -1.125986 1.652310 -0.312290
C -2.037332 -1.448513 1.307750
C -3.130005 -0.676648 0.802351
C -2.069029 -1.941072 2.618185
C -4.174657 -0.462797 1.687748
C -3.145514 -1.704777 3.490737
H -1.238769 -2.530212 2.986606
C -4.186079 -0.950534 2.994613
H -3.156105 -2.098622 4.500619
C -2.502439 0.837452 -1.224507
C -3.277297 -0.188324 -0.599098
C -2.783180 1.225821 -2.541841
C -4.291547 -0.730575 -1.372139
C -3.813069 0.646182 -3.301689
H -2.199071 2.010958 -3.003397
C -4.555198 -0.336285 -2.683825
H -4.014172 0.959871 -4.319624
C -0.567697 2.997924 -1.447537
C -0.980612 4.328454 -1.286418
C 0.282722 2.673925 -2.518793
C -0.554312 5.313575 -2.181788
H -1.637721 4.602001 -0.468044
C 0.699374 3.656990 -3.415473
H 0.615120 1.648611 -2.654424
C 0.282869 4.981004 -3.247480
H -0.884633 6.339639 -2.046611
H 1.348041 3.389746 -4.245065
H 0.605944 5.746875 -3.946970
C -1.930294 2.546941 1.081782
C -3.273975 2.948192 1.012709
C -1.168946 2.899809 2.206824
C -3.842804 3.684801 2.053718
H -3.879277 2.681654 0.152373
C -1.740392 3.636542 3.244764
H -0.128281 2.596052 2.270456
C -3.078693 4.029246 3.170722

H	-4.882927	3.992121	1.987947
H	-1.141897	3.900266	4.112134
H	-3.523604	4.601296	3.979973
C	0.479606	-2.930105	1.221347
C	0.391859	-4.301788	0.940750
C	1.326257	-2.496302	2.255032
C	1.132252	-5.222170	1.685925
H	-0.248265	-4.657434	0.140708
C	2.055830	-3.419740	3.003679
H	1.418921	-1.436090	2.468022
C	1.961459	-4.784906	2.719732
H	1.058847	-6.281128	1.455641
H	2.704052	-3.070394	3.802266
H	2.533687	-5.503271	3.299936
C	-1.031255	-2.551263	-1.241970
C	-0.232321	-2.422571	-2.388615
C	-2.160877	-3.384407	-1.273406
C	-0.565518	-3.116360	-3.552888
H	0.654373	-1.797407	-2.354129
C	-2.485677	-4.077488	-2.440577
H	-2.791336	-3.486085	-0.395271
C	-1.690729	-3.942991	-3.581297
H	0.056787	-3.014078	-4.437452
H	-3.358197	-4.724830	-2.456794
H	-1.946449	-4.482986	-4.488570
H	2.841673	-3.336348	-1.289676
O	-5.331517	0.229177	1.448066
O	-5.341880	-0.583060	3.617769
O	-5.153422	-1.733682	-1.016329
O	-5.590081	-1.064786	-3.189242
C	-6.064516	0.227505	2.681957
H	-6.134720	1.252007	3.065131
H	-7.056486	-0.203291	2.516688
C	-6.066707	-1.883281	-2.112240
H	-6.085342	-2.928840	-2.430988
H	-7.063734	-1.542703	-1.808383

TS-4^{s}

Zero-point correction= 0.886658 (Hartree/Particle)
Thermal correction to Energy= 0.944366
Thermal correction to Enthalpy= 0.945311
Thermal correction to Gibbs Free Energy= 0.791229
Sum of electronic and zero-point Energies= -3453.666195
Sum of electronic and thermal Energies= -3453.608487
Sum of electronic and thermal Enthalpies= -3453.607543
Sum of electronic and thermal Free Energies= -3453.761624
HF(M06/6-311++G(d,p) and SDD, SMD[1,4Dioxane])= -3453.11840658

C	3.117592	2.235064	0.048638
O	2.053964	1.875303	-0.532938
N	3.635299	3.450101	-0.227926
C	2.894982	4.376604	-1.086376
H	3.182294	5.396941	-0.819156
H	3.131941	4.206142	-2.143210
H	1.826623	4.246792	-0.938197
C	5.011780	3.859002	0.068380
H	5.492594	4.175692	-0.864132
H	5.021047	4.703703	0.766062
H	5.581421	3.036948	0.495243
C	3.815081	1.370498	1.041824
C	4.133504	0.038830	0.739119
H	1.737968	-0.589960	0.725862
C	4.213680	-3.305857	-1.010502
H	4.018165	-3.172715	-2.080678
H	4.973766	-4.087049	-0.905008
C	4.718357	-2.011643	-0.355143
Ir	0.715782	0.143706	-0.363580
C	4.140779	1.874748	2.313083
C	4.778241	1.067264	3.255517
C	5.120313	-0.250857	2.940662
C	4.795717	-0.760303	1.683475
H	3.887537	2.900833	2.564439
H	5.017589	1.472011	4.234508
H	5.634580	-0.870676	3.670391
C	5.042293	-2.150273	1.145135
H	6.072274	-2.493765	1.293257
H	4.377425	-2.879007	1.626001
C	3.755194	-0.778788	-0.492992
C	5.967970	-1.542693	-1.133503
O	7.071735	-2.024009	-1.010777
O	2.392075	-1.192892	-0.374446
C	4.087848	-0.188544	-1.885325
H	3.534275	-0.783748	-2.620126
H	3.765402	0.846085	-2.010544
C	5.601298	-0.384489	-2.063759
H	5.908001	-0.601776	-3.092038
H	6.180286	0.492131	-1.744527
P	-0.614031	-1.708308	-0.129843
P	-1.056073	1.640052	-0.169695
C	-1.933242	-1.549691	1.163691
C	-3.042495	-0.668018	0.970585
C	-1.808349	-2.244554	2.374392
C	-3.914070	-0.542279	2.041612
C	-2.713923	-2.100473	3.439320
H	-0.992788	-2.940078	2.511300
C	-3.758433	-1.224578	3.246153
H	-2.595699	-2.655965	4.362699
C	-2.672743	1.107476	-0.891190
C	-3.399239	0.035366	-0.292114
C	-3.171478	1.729464	-2.044673
C	-4.581021	-0.322829	-0.921821
C	-4.372122	1.339579	-2.661757
H	-2.629539	2.553221	-2.487970
C	-5.057785	0.299646	-2.073997

H	-4.739306	1.835798	-3.552873
C	-0.692822	3.170337	-1.138139
C	-0.985779	4.456691	-0.665272
C	-0.169476	3.024942	-2.435247
C	-0.775155	5.573815	-1.479659
H	-1.383918	4.593899	0.334366
C	0.025667	4.138541	-3.251213
H	0.076836	2.035246	-2.811385
C	-0.278727	5.417525	-2.774481
H	-1.008977	6.564838	-1.100965
H	0.416951	4.009120	-4.256485
H	-0.128436	6.285859	-3.409717
C	-1.443621	2.210732	1.532060
C	-2.670606	2.822983	1.836238
C	-0.479036	2.061815	2.539448
C	-2.919264	3.289622	3.127723
H	-3.433806	2.925714	1.070599
C	-0.733974	2.527385	3.830482
H	0.459528	1.566670	2.307212
C	-1.951839	3.143403	4.125572
H	-3.868596	3.767395	3.353862
H	0.015854	2.403169	4.606731
H	-2.149321	3.504799	5.130887
C	0.324262	-3.217358	0.388233
C	0.218088	-4.422686	-0.319218
C	1.139391	-3.180341	1.534127
C	0.891653	-5.567073	0.118707
H	-0.391362	-4.481105	-1.213300
C	1.793684	-4.327261	1.980900
H	1.251996	-2.256182	2.093399
C	1.671755	-5.526628	1.272883
H	0.796503	-6.490968	-0.444502
H	2.400185	-4.284382	2.881493
H	2.184967	-6.419298	1.618694
C	-1.476402	-2.227825	-1.666480
C	-0.937292	-1.852488	-2.905876
C	-2.618320	-3.044123	-1.632255
C	-1.530325	-2.284745	-4.093570
H	-0.049854	-1.225296	-2.934755
C	-3.207033	-3.475945	-2.821087
H	-3.050631	-3.337184	-0.680142
C	-2.665042	-3.096976	-4.052323
H	-1.105465	-1.989094	-5.048627
H	-4.087499	-4.111629	-2.785891
H	-3.125954	-3.434865	-4.976168
H	3.287410	-3.637287	-0.537538
O	-5.037924	0.239037	2.101378
O	-4.769371	-0.899636	4.101753
O	-5.436804	-1.330035	-0.560936
O	-6.225602	-0.283078	-2.469263
C	-5.587742	0.055600	3.413491
H	-5.567530	1.008680	3.953585
H	-6.608615	-0.331153	3.331882
C	-6.553190	-1.244492	-1.456737
H	-6.722487	-2.219283	-1.921910
H	-7.440516	-0.907740	-0.907061

I-5^S

Zero-point correction= 0.892107 (Hartree/Particle)
Thermal correction to Energy= 0.950213
Thermal correction to Enthalpy= 0.951157
Thermal correction to Gibbs Free Energy= 0.795830
Sum of electronic and zero-point Energies= -3453.706240
Sum of electronic and thermal Energies= -3453.648134
Sum of electronic and thermal Enthalpies= -3453.647189
Sum of electronic and thermal Free Energies= -3453.802516
HF(M06/6-311++G(d,p) and SDD, SMD[1,4Dioxane])= -3453.17031012

C	3.094920	2.211628	0.284655
O	2.034560	1.897733	-0.316945
N	3.560931	3.476069	0.175636
C	2.752927	4.485764	-0.513593
H	2.964120	5.460173	-0.064206
H	3.001682	4.525196	-1.580386
H	1.695946	4.252865	-0.409574
C	4.929277	3.898234	0.484771
H	5.364774	4.364414	-0.406595
H	4.932468	4.636135	1.294874
H	5.546452	3.050265	0.773160
C	3.851054	1.242104	1.136932
C	4.215442	-0.032533	0.670656
H	2.357154	-1.823592	0.095580
C	4.308185	-3.184036	-1.409085
H	3.983561	-2.954830	-2.429621
H	5.112798	-3.923725	-1.468120
C	4.826393	-1.937456	-0.672294
Ir	0.652668	0.208150	-0.249636
C	4.198960	1.598979	2.449767
C	4.905102	0.717483	3.270876
C	5.300214	-0.531816	2.789796
C	4.951344	-0.901486	1.489997
H	3.910061	2.573696	2.831802
H	5.157785	1.015193	4.284236
H	5.872131	-1.206829	3.420800
C	5.249019	-2.206720	0.788532
H	6.305388	-2.493464	0.838578
H	4.669585	-3.029593	1.228154
C	3.849492	-0.717219	-0.639075
C	6.020975	-1.370921	-1.483505
O	7.121698	-1.869784	-1.500611
O	2.448519	-1.125880	-0.581925
C	4.089281	0.034258	-1.961559
H	3.508917	-0.480025	-2.735147
H	3.742583	1.067487	-1.940633

C	5.595420	-0.107156	-2.230526
H	5.854796	-0.176396	-3.291384
H	6.174772	0.730659	-1.820837
P	-0.621641	-1.619598	0.061843
P	-1.107196	1.605594	-0.276495
C	-2.024021	-1.440745	1.252717
C	-3.155012	-0.647265	0.888634
C	-1.954350	-2.016381	2.527208
C	-4.121307	-0.485229	1.869120
C	-2.955540	-1.837635	3.498056
H	-1.106389	-2.633990	2.792191
C	-4.028677	-1.052465	3.138966
H	-2.885929	-2.296131	4.477987
C	-2.641195	0.962703	-1.087310
C	-3.406568	-0.067863	-0.460474
C	-3.025283	1.451245	-2.342767
C	-4.505504	-0.524501	-1.171756
C	-4.145712	0.965688	-3.039168
H	-2.450370	2.241210	-2.807531
C	-4.868463	-0.032365	-2.423970
H	-4.425801	1.358904	-4.009869
C	-0.706749	3.106460	-1.285952
C	-1.103412	4.401557	-0.922810
C	-0.002628	2.925737	-2.489694
C	-0.812183	5.490066	-1.750747
H	-1.641340	4.569014	0.003983
C	0.275487	4.010884	-3.320489
H	0.323026	1.927417	-2.769158
C	-0.128832	5.297622	-2.952201
H	-1.127853	6.487155	-1.456261
H	0.808259	3.852928	-4.254436
H	0.086656	6.143630	-3.599002
C	-1.698124	2.278227	1.336031
C	-2.962989	2.873003	1.474636
C	-0.845043	2.218599	2.447683
C	-3.357749	3.410138	2.701021
H	-3.643831	2.907604	0.629051
C	-1.243735	2.753724	3.674005
H	0.120981	1.731612	2.342749
C	-2.498647	3.352580	3.801800
H	-4.336025	3.873750	2.796250
H	-0.578089	2.697291	4.531046
H	-2.808990	3.768766	4.756260
C	0.428096	-2.950771	0.832186
C	0.452643	-4.275246	0.368281
C	1.251973	-2.604160	1.923366
C	1.264528	-5.230216	0.987013
H	-0.164789	-4.567317	-0.473776
C	2.055422	-3.563814	2.545792
H	1.245092	-1.580201	2.290396
C	2.062498	-4.880756	2.078222
H	1.267958	-6.251088	0.615601
H	2.668453	-3.281169	3.397220
H	2.684285	-5.628855	2.561640
C	-1.362048	-2.450346	-1.406288
C	-0.846808	-2.155859	-2.676959
C	-2.397602	-3.391483	-1.284972
C	-1.352315	-2.798436	-3.808822
H	-0.060565	-1.410716	-2.765973
C	-2.899152	-4.033212	-2.418122
H	-2.818272	-3.616106	-0.308900
C	-2.376656	-3.738519	-3.680634
H	-0.950246	-2.561398	-4.789934
H	-3.697983	-4.762698	-2.315357
H	-2.770604	-4.237671	-4.561577
H	3.463382	-3.640091	-0.882232
O	-5.286344	0.228174	1.771154
O	-5.126200	-0.720109	3.879178
O	-5.374538	-1.516997	-0.801533
O	-5.976808	-0.686828	-2.878651
C	-5.916284	0.148799	3.056651
H	-5.949791	1.147028	3.508458
H	-6.921552	-0.269302	2.946092
C	-6.369901	-1.583236	-1.831056
H	-6.419241	-2.603015	-2.224361
H	-7.338835	-1.268512	-1.425534

I-3^{R}-*HM*

Zero-point correction= 0.889394 (Hartree/Particle)
Thermal correction to Energy= 0.947371
Thermal correction to Enthalpy= 0.948315
Thermal correction to Gibbs Free Energy= 0.794827
Sum of electronic and zero-point Energies= -3453.706192
Sum of electronic and thermal Energies= -3453.648215
Sum of electronic and thermal Enthalpies= -3453.647270
Sum of electronic and thermal Free Energies= -3453.800758
HF(M06/6-311++G(d,p) and SDD, SMD[1,4-Dioxane]) = -3453.15584689

C	-2.188440	-1.991439	1.895388
O	-1.424405	-0.990502	2.075754
N	-2.176560	-2.998812	2.799798
C	-1.508665	-2.818688	4.087954
H	-2.130452	-3.263748	4.872144
H	-0.530092	-3.313399	4.095762
H	-1.375362	-1.757176	4.287728
C	-2.601731	-4.370791	2.522482
H	-1.804745	-5.049161	2.847376
H	-3.516099	-4.630845	3.069478
H	-2.760242	-4.515966	1.455183
C	-3.113189	-1.946645	0.738329
C	-2.786602	-0.965211	-0.233807
C	-4.489890	2.956768	0.927778
C	-4.354506	-2.610386	0.725818
C	-5.284733	-2.322684	-0.264943
C	-5.005780	-1.316401	-1.190284

C	-3.791048	-0.616068	-1.161046
H	-4.623846	-3.310549	1.507883
H	-6.239558	-2.839278	-0.287107
H	-5.759800	-1.038629	-1.922795
C	-3.508856	2.094264	0.147253
O	-2.505302	1.638419	0.695939
C	-3.990884	1.921758	-1.286595
C	-5.837256	2.817000	0.194429
C	-5.520618	2.127711	-1.135096
C	-3.645642	0.612211	-2.035534
H	-2.631511	0.691702	-2.431901
H	-4.336826	0.567092	-2.884657
C	-3.497073	3.158292	-2.103872
H	-3.802087	4.111098	-1.658415
H	-2.406892	3.148863	-2.181952
H	-3.928027	3.099853	-3.107963
O	-6.331752	1.801624	-1.968102
H	-6.545296	2.185557	0.743014
H	-6.343950	3.769873	0.011557
H	-4.121664	3.991253	0.916220
H	-4.502901	2.639456	1.973554
H	-0.925538	0.735825	-1.347468
Ir	-1.040439	0.100404	0.088572
P	0.397230	-1.478976	-0.771497
P	0.763457	1.610891	0.779693
C	1.719663	-0.679637	-1.789602
C	2.792274	0.020552	-1.146421
C	1.650309	-0.725022	-3.186603
C	3.696853	0.637265	-1.997386
C	2.588146	-0.088176	-4.018278
H	0.857368	-1.284192	-3.665140
C	3.600705	0.599971	-3.388131
H	2.519728	-0.143234	-5.098810
C	2.322496	0.782369	1.301563
C	3.081248	0.073021	0.320009
C	2.739894	0.813961	2.636018
C	4.231207	-0.545166	0.785298
C	3.907014	0.163624	3.076757
H	2.155618	1.364970	3.363338
C	4.632717	-0.512759	2.120888
H	4.223229	0.200380	4.113197
C	0.225036	2.590641	2.252218
C	0.491208	3.961095	2.395289
C	-0.498100	1.930514	3.262113
C	0.056216	4.652629	3.528291
H	1.035970	4.497042	1.626413
C	-0.919522	2.623434	4.397394
H	-0.738785	0.877956	3.155655
C	-0.644904	3.986203	4.533694
H	0.270147	5.713632	3.622929
H	-1.470633	2.098743	5.173327
H	-0.978311	4.525307	5.416096
C	1.373152	2.888624	-0.408858
C	2.599993	3.539701	-0.192798
C	0.606844	3.248968	-1.525186
C	3.035875	4.537522	-1.065812
H	3.218879	3.263789	0.655445
C	1.046103	4.241868	-2.402843
H	-0.327258	2.732854	-1.715778
C	2.259936	4.891463	-2.172840
H	3.981854	5.038717	-0.879211
H	0.442896	4.504625	-3.267530
H	2.600889	5.667215	-2.852745
C	-0.482528	-2.659724	-1.891915
C	-0.432521	-4.049365	-1.705772
C	-1.284895	-2.152276	-2.929361
C	-1.143390	-4.906465	-2.550467
H	0.158650	-4.475441	-0.904211
C	-1.978610	-3.009084	-3.781516
H	-1.372275	-1.080248	-3.068291
C	-1.909646	-4.391773	-3.594784
H	-1.090500	-5.979542	-2.389161
H	-2.582524	-2.595925	-4.584411
H	-2.455651	-5.059980	-4.254464
C	1.396441	-2.572779	0.332832
C	1.257508	-2.561397	1.726204
C	2.359480	-3.415929	-0.251911
C	2.056904	-3.389399	2.519094
H	0.545067	-1.887998	2.185802
C	3.142102	-4.253166	0.542123
H	2.503134	-3.416371	-1.328291
C	2.991524	-4.242916	1.931773
H	1.957495	-3.356816	3.600846
H	3.872368	-4.908202	0.075035
H	3.605927	-4.889914	2.551765
O	4.807443	1.352965	-1.637445
O	4.637155	1.289707	-3.945487
O	5.130245	-1.274017	0.054801
O	5.792359	-1.216876	2.274552
C	5.357768	1.876267	-2.852965
H	5.226445	2.964882	-2.872966
H	6.413532	1.600706	-2.924103
C	6.128796	-1.727603	0.977608
H	6.126830	-2.822651	1.006176
H	7.108025	-1.341295	0.675963

TS-3^{R}-HM

Zero-point correction= 0.885956 (Hartree/Particle)
Thermal correction to Energy= 0.943803
Thermal correction to Enthalpy= 0.944747
Thermal correction to Gibbs Free Energy= 0.790299
Sum of electronic and zero-point Energies= -3453.594230
Sum of electronic and thermal Energies= -3453.536384
Sum of electronic and thermal Enthalpies= -3453.535440
Sum of electronic and thermal Free Energies= -3453.689887

HF(M06/6-311++G(d,p) and SDD, SMD[1,4-Dioxane]) = -3453.05113094

C	-1.935437	-1.315469	2.267969
O	-1.153035	-0.368865	1.876216
N	-1.704691	-1.819409	3.498362
C	-0.817980	-1.102019	4.419773
H	-1.182587	-1.259904	5.438831
H	0.214806	-1.464981	4.350950
H	-0.825410	-0.037192	4.194806
C	-2.111662	-3.153125	3.945246
H	-1.285129	-3.584471	4.518156
H	-2.995505	-3.115562	4.592784
H	-2.304492	-3.803572	3.093273
C	-3.008102	-1.681229	1.355020
C	-2.962414	-0.970478	0.116202
C	-2.824551	3.113388	-0.365267
C	-4.116339	-2.481386	1.705846
C	-5.180242	-2.611557	0.830198
C	-5.172466	-1.879370	-0.357606
C	-4.101414	-1.049643	-0.703762
H	-4.179021	-2.962093	2.672331
H	-6.035053	-3.227100	1.091903
H	-6.040229	-1.915700	-1.010989
C	-2.675002	1.729116	-0.983347
O	-1.636526	1.435734	-1.839977
C	-4.054239	1.344590	-1.526801
C	-4.209690	3.011570	0.314913
C	-4.973305	1.907539	-0.435657
C	-4.248830	-0.135956	-1.887610
H	-3.532622	-0.392264	-2.681204
H	-5.245772	-0.254195	-2.322981
C	-4.341031	2.178822	-2.815372
H	-4.288718	3.258341	-2.648474
H	-3.618642	1.918652	-3.596056
H	-5.348151	1.947610	-3.175487
O	-6.109870	1.555571	-0.215807
H	-4.115115	2.698683	1.362046
H	-4.776319	3.947717	0.311061
H	-2.834954	3.877345	-1.154514
H	-2.030214	3.375962	0.329620
H	-1.587475	0.085499	-1.764964
Ir	-1.297645	0.254981	-0.112454
P	0.385649	-1.584276	-0.827845
P	0.653900	1.816023	0.470297
C	1.895928	-0.920790	-1.665148
C	2.852291	-0.215707	-0.868525
C	2.138874	-1.132963	-3.027535
C	3.984354	0.223947	-1.535960
C	3.293085	-0.659381	-3.676425
H	1.431554	-1.700486	-3.617487
C	4.199579	0.026440	-2.899240
H	3.464616	-0.836957	-4.732075
C	1.962876	0.856011	1.347636
C	2.802796	-0.042558	0.616301
C	2.091604	0.965668	2.737076
C	3.716592	-0.759463	1.373528
C	3.024843	0.214872	3.475537
H	1.462140	1.663615	3.275223
C	3.823932	-0.649951	2.759933
H	3.120401	0.323340	4.550286
C	0.111844	3.088182	1.709765
C	0.662812	4.378456	1.752731
C	-0.899677	2.758624	2.631693
C	0.224133	5.307741	2.699096
H	1.429618	4.671183	1.045275
C	-1.329929	3.687260	3.580170
H	-1.355614	1.775630	2.597437
C	-0.768643	4.965751	3.617145
H	0.660661	6.302381	2.712635
H	-2.111016	3.414289	4.284679
H	-1.108393	5.691097	4.350829
C	1.590800	2.801539	-0.776170
C	2.878892	3.280227	-0.476964
C	1.006867	3.139190	-2.006895
C	3.559062	4.091050	-1.385879
H	3.355372	3.015042	0.461493
C	1.697138	3.943236	-2.916829
H	0.021322	2.765990	-2.254893
C	2.969922	4.424458	-2.608376
H	4.548241	4.464985	-1.135366
H	1.235901	4.193938	-3.867960
H	3.501022	5.055693	-3.315297
C	-0.556311	-2.494201	-2.138563
C	-0.984885	-3.815580	-1.948972
C	-0.977877	-1.816745	-3.299465
C	-1.793288	-4.447342	-2.898987
H	-0.693583	-4.362964	-1.059795
C	-1.772413	-2.453450	-4.252769
H	-0.668397	-0.790025	-3.480804
C	-2.183941	-3.774050	-4.055081
H	-2.113148	-5.471619	-2.729808
H	-2.068276	-1.916736	-5.149859
H	-2.804960	-4.269782	-4.795344
C	1.125663	-2.942888	0.194016
C	1.024386	-2.929543	1.590225
C	1.862107	-3.968611	-0.425115
C	1.621198	-3.936775	2.354319
H	0.511250	-2.111252	2.077281
C	2.451616	-4.975632	0.337505
H	1.976939	-3.980901	-1.504809
C	2.327005	-4.965888	1.730298
H	1.542167	-3.912416	3.438092
H	3.011227	-5.765812	-0.155061
H	2.783035	-5.753816	2.323432
O	5.049361	0.895418	-0.998214
O	5.395022	0.571248	-3.266350
O	4.632176	-1.665934	0.914871
O	4.800035	-1.485186	3.218634
C	5.945478	1.168122	-2.084883

H	6.024664	2.251297	-2.228075
H	6.922399	0.722818	-1.872114
C	5.304868	-2.183137	2.069815
H	5.086867	-3.252159	2.168344
H	6.380345	-2.002363	1.978808

I-3^{R}-*HM*

Zero-point correction= 0.891677 (Hartree/Particle)
Thermal correction to Energy= 0.949398
Thermal correction to Enthalpy= 0.950343
Thermal correction to Gibbs Free Energy= 0.797394
Sum of electronic and zero-point Energies= -3453.636480
Sum of electronic and thermal Energies= -3453.578758
Sum of electronic and thermal Enthalpies= -3453.577814
Sum of electronic and thermal Free Energies= -3453.730763
HF(M06/6-311++G(d,p) and SDD, SMD[1,4-Dioxane]) = -3453.09963426

C	-1.662541	-1.328564	2.385598
O	-0.943163	-0.355452	1.943144
N	-1.335948	-1.817992	3.600739
C	-0.391191	-1.081899	4.444537
H	-0.658640	-1.255020	5.490867
H	0.640945	-1.413906	4.279319
H	-0.448317	-0.016722	4.228237
C	-1.678454	-3.159018	4.076601
H	-0.810242	-3.561986	4.606928
H	-2.526343	-3.143250	4.771650
H	-1.896651	-3.822044	3.240351
C	-2.787427	-1.727411	1.551787
C	-2.841173	-1.015476	0.313595
C	-2.757245	3.086988	-0.328630
C	-3.849151	-2.550080	1.983803
C	-4.975064	-2.698249	1.192005
C	-5.070310	-1.963141	0.009473
C	-4.040688	-1.117914	-0.414032
H	-3.828208	-3.036359	2.949284
H	-5.796117	-3.329999	1.516051
H	-5.985283	-2.011981	-0.575827
C	-2.637499	1.641478	-0.792544
O	-1.626307	1.322570	-1.801913
C	-4.038945	1.265045	-1.299115
C	-4.104613	3.045400	0.433278
C	-4.903721	1.893127	-0.193867
C	-4.286872	-0.222227	-1.594280
H	-3.664701	-0.523778	-2.451681
H	-5.323275	-0.329024	-1.930042
C	-4.402679	2.051538	-2.601434
H	-4.319103	3.135450	-2.493760
H	-3.764241	1.747716	-3.440106
H	-5.437992	1.823954	-2.872411
O	-6.024948	1.551449	0.102637
H	-3.953800	2.813084	1.494378
H	-4.669083	3.981861	0.388361
H	-2.814896	3.774669	-1.182252
H	-1.935086	3.415880	0.301941
H	-1.991959	0.716257	-2.480954
Ir	-1.226580	0.251472	-0.009853
P	0.268649	-1.516057	-0.938844
P	0.690067	1.826370	0.431627
C	1.912170	-1.022707	-1.634841
C	2.861330	-0.353555	-0.802288
C	2.255642	-1.359963	-2.952344
C	4.068112	-0.031157	-1.407524
C	3.490124	-1.024585	-3.531429
H	1.553576	-1.912193	-3.563349
C	4.376448	-0.338227	-2.730593
H	3.734011	-1.297952	-4.551775
C	1.985154	0.876185	1.347630
C	2.764807	-0.115587	0.672724
C	2.143335	1.072079	2.724318
C	3.630035	-0.847026	1.474816
C	3.037763	0.318249	3.506564
H	1.563778	1.840177	3.220517
C	3.763580	-0.650299	2.849049
H	3.157460	0.499906	4.568994
C	0.197180	3.155443	1.629957
C	0.761696	4.439732	1.608505
C	-0.788466	2.867652	2.593092
C	0.357061	5.408659	2.530498
H	1.514072	4.694898	0.870945
C	-1.182863	3.834873	3.517983
H	-1.248852	1.885388	2.609059
C	-0.611628	5.109707	3.488473
H	0.802056	6.399060	2.495160
H	-1.942682	3.595171	4.257068
H	-0.924151	5.865044	4.203841
C	1.645748	2.748392	-0.851831
C	2.973421	3.145942	-0.616281
C	1.030466	3.119128	-2.057630
C	3.664388	3.900996	-1.564628
H	3.471599	2.860951	0.304888
C	1.727155	3.871930	-3.005644
H	0.009892	2.815806	-2.257813
C	3.043382	4.265771	-2.761805
H	4.686863	4.209803	-1.363828
H	1.238727	4.151519	-3.935062
H	3.582565	4.854234	-3.498805
C	-0.759987	-1.929267	-2.423466
C	-1.654911	-3.010625	-2.401101
C	-0.792474	-1.060989	-3.535567
C	-2.529210	-3.237820	-3.467156
H	-1.675826	-3.682969	-1.550345
C	-1.668796	-1.291163	-4.600015
H	-0.103648	-0.222914	-3.590023

C	-2.539030	-2.383962	-4.570819
H	-3.204438	-4.087942	-3.430342
H	-1.658229	-0.623473	-5.457451
H	-3.214544	-2.568281	-5.400932
C	0.674486	-3.141762	-0.155294
C	0.886527	-3.179519	1.230754
C	0.895623	-4.307452	-0.908174
C	1.293959	-4.362204	1.853641
H	0.757463	-2.276155	1.815522
C	1.291301	-5.489021	-0.282242
H	0.764327	-4.296186	-1.985543
C	1.488700	-5.520474	1.100667
H	1.463558	-4.376691	2.927215
H	1.454210	-6.383594	-0.876658
H	1.800388	-6.441565	1.585016
O	5.136674	0.601022	-0.826535
O	5.633598	0.095864	-3.032036
O	4.478003	-1.844482	1.073599
O	4.686069	-1.519053	3.357608
C	6.124121	0.752323	-1.855666
H	6.270455	1.817589	-2.067613
H	7.058636	0.279959	-1.537820
C	5.213936	-2.243247	2.236229
H	5.080765	-3.316727	2.400720
H	6.272793	-1.991407	2.105754

I-3^{R}HM^{c}

HF(M06/6-311++G(d,p) and SDD, SMD[1,4Dioxane])= -3453,12027728

C	-1.713000	-1.269000	2.577000
O	-0.836000	-0.450000	2.098000
N	-1.410000	-1.828000	3.765000
C	-0.281000	-1.305000	4.542000
H	-0.506000	-1.439000	5.604000
H	0.649000	-1.835000	4.304000
H	-0.138000	-0.248000	4.331000
C	-1.973000	-3.084000	4.266000
H	-1.164000	-3.652000	4.734000
H	-2.751000	-2.911000	5.018000
H	-2.375000	-3.681000	3.449000
C	-2.926000	-1.475000	1.795000
C	-2.894000	-0.828000	0.523000
C	-2.645000	3.188000	-0.832000
C	-4.086000	-2.125000	2.267000
C	-5.208000	-2.199000	1.458000
C	-5.175000	-1.609000	0.193000
C	-4.049000	-0.924000	-0.277000
H	-4.138000	-2.533000	3.267000
H	-6.109000	-2.688000	1.813000
H	-6.062000	-1.654000	-0.434000
C	-2.538000	1.751000	-1.335000
O	-1.477000	1.202000	-1.758000
C	-3.928000	1.245000	-1.658000
C	-4.033000	3.263000	-0.170000
C	-4.820000	2.057000	-0.705000
C	-4.132000	-0.277000	-1.639000
H	-3.393000	-0.715000	-2.315000
H	-5.118000	-0.477000	-2.069000
C	-4.240000	1.759000	-3.105000
H	-4.108000	2.840000	-3.214000
H	-3.588000	1.253000	-3.823000
H	-5.282000	1.521000	-3.336000
O	-5.969000	1.790000	-0.442000
H	-3.963000	3.162000	0.919000
H	-4.574000	4.192000	-0.372000
H	-2.594000	3.835000	-1.720000
H	-1.815000	3.467000	-0.183000
H	-2.047000	1.444000	1.052000
Ir	-1.209000	0.342000	0.243000
P	0.124000	-1.459000	-1.021000
P	0.789000	1.808000	0.561000
C	1.736000	-0.919000	-1.763000
C	2.746000	-0.358000	-0.919000
C	2.002000	-1.122000	-3.123000
C	3.930000	-0.008000	-1.553000
C	3.215000	-0.757000	-3.732000
H	1.258000	-1.597000	-3.749000
C	4.162000	-0.182000	-2.915000
H	3.395000	-0.927000	-4.787000
C	2.077000	0.759000	1.360000
C	2.740000	-0.237000	0.576000
C	2.321000	0.857000	2.734000
C	3.592000	-1.078000	1.278000
C	3.199000	-0.010000	3.412000
H	1.826000	1.632000	3.307000
C	3.810000	-0.984000	2.653000
H	3.394000	0.093000	4.474000
C	0.397000	3.146000	1.785000
C	0.868000	4.461000	1.645000
C	-0.439000	2.847000	2.878000
C	0.521000	5.445000	2.575000
H	1.504000	4.729000	0.810000
C	-0.772000	3.828000	3.812000
H	-0.832000	1.843000	2.994000
C	-0.295000	5.133000	3.662000
H	0.892000	6.458000	2.444000
H	-1.413000	3.576000	4.652000
H	-0.561000	5.899000	4.384000
C	1.728000	2.700000	-0.755000
C	3.032000	3.156000	-0.494000
C	1.145000	2.978000	-2.000000
C	3.730000	3.887000	-1.455000
H	3.505000	2.936000	0.458000
C	1.852000	3.703000	-2.962000
H	0.152000	2.607000	-2.219000
C	3.141000	4.163000	-2.692000
H	4.732000	4.245000	-1.235000
H	1.393000	3.906000	-3.926000
H	3.686000	4.731000	-3.441000
C	-0.934000	-2.024000	-2.428000

C -1.701000 -3.194000 -2.302000
C -1.104000 -1.235000 -3.582000
C -2.581000 -3.585000 -3.314000
H -1.613000 -3.812000 -1.416000
C -1.971000 -1.638000 -4.599000
H -0.563000 -0.303000 -3.689000
C -2.710000 -2.816000 -4.471000
H -3.159000 -4.498000 -3.197000
H -2.069000 -1.027000 -5.492000
H -3.384000 -3.129000 -5.264000
C 0.651000 -3.040000 -0.209000
C 0.773000 -3.122000 1.184000
C 1.045000 -4.143000 -0.989000
C 1.249000 -4.290000 1.788000
H 0.526000 -2.262000 1.791000
C 1.514000 -5.308000 -0.385000
H 0.986000 -4.094000 -2.071000
C 1.612000 -5.387000 1.008000
H 1.339000 -4.339000 2.870000
H 1.808000 -6.152000 -1.002000
H 1.975000 -6.298000 1.477000
O 5.043000 0.536000 -0.967000
O 5.415000 0.251000 -3.236000
O 4.358000 -2.088000 0.764000
O 4.702000 -1.938000 3.049000
C 5.989000 0.755000 -2.023000
H 6.176000 1.829000 -2.128000
H 6.913000 0.211000 -1.803000
C 5.021000 -2.701000 1.876000
H 4.656000 -3.727000 1.997000
H 6.103000 -2.682000 1.713000

TS-3^{R}HM^{c}

HF(M06/6-311++G(d,p) and SDD, SMD[1,4Dioxane])= -3453,10898768

C -1.627000 -1.658000 2.324000
O -0.844000 -0.695000 1.984000
N -1.320000 -2.317000 3.460000
C -0.313000 -1.763000 4.369000
H -0.596000 -2.020000 5.394000
H 0.683000 -2.173000 4.163000
H -0.270000 -0.681000 4.264000
C -1.740000 -3.685000 3.768000
H -0.871000 -4.228000 4.154000
H -2.526000 -3.708000 4.532000
H -2.083000 -4.195000 2.869000
C -2.800000 -1.884000 1.483000
C -2.814000 -1.088000 0.300000
C -2.767000 3.282000 -0.439000
C -3.929000 -2.624000 1.889000
C -5.078000 -2.606000 1.116000
C -5.116000 -1.807000 -0.027000
C -4.014000 -1.042000 -0.431000
H -3.940000 -3.168000 2.824000
H -5.955000 -3.168000 1.422000
H -6.035000 -1.747000 -0.603000
C -2.618000 1.835000 -0.939000
O -1.587000 1.370000 -1.600000
C -4.014000 1.371000 -1.364000
C -4.160000 3.278000 0.233000
C -4.904000 2.042000 -0.316000
C -4.178000 -0.136000 -1.623000
H -3.450000 -0.400000 -2.398000
H -5.173000 -0.288000 -2.053000
C -4.317000 2.091000 -2.721000
H -4.297000 3.182000 -2.648000
H -3.586000 1.779000 -3.473000
H -5.318000 1.799000 -3.053000
O -6.009000 1.679000 0.018000
H -4.085000 3.178000 1.322000
H -4.742000 4.184000 0.040000
H -2.736000 3.944000 -1.312000
H -1.958000 3.583000 0.227000
H -2.421000 1.322000 0.489000
Ir -1.170000 0.177000 0.112000
P 0.211000 -1.392000 -1.065000
P 0.681000 1.758000 0.641000
C 1.785000 -0.714000 -1.764000
C 2.784000 -0.170000 -0.892000
C 2.040000 -0.813000 -3.139000
C 3.942000 0.272000 -1.514000
C 3.228000 -0.357000 -3.733000
H 1.306000 -1.270000 -3.787000
C 4.162000 0.199000 -2.888000
H 3.400000 -0.446000 -4.799000
C 2.013000 0.760000 1.427000
C 2.776000 -0.127000 0.604000
C 2.212000 0.794000 2.810000
C 3.692000 -0.923000 1.276000
C 3.148000 -0.032000 3.459000
H 1.635000 1.486000 3.412000
C 3.870000 -0.891000 2.659000
H 3.303000 0.014000 4.532000
C 0.114000 2.947000 1.944000
C 0.542000 4.283000 1.987000
C -0.813000 2.505000 2.907000
C 0.061000 5.151000 2.971000
H 1.245000 4.657000 1.252000
C -1.283000 3.372000 3.894000
H -1.164000 1.479000 2.883000
C -0.848000 4.700000 3.928000
H 0.401000 6.183000 2.986000
H -1.993000 3.012000 4.633000
H -1.220000 5.377000 4.691000
C 1.589000 2.821000 -0.562000
C 2.858000 3.333000 -0.238000
C 1.009000 3.160000 -1.794000
C 3.524000 4.179000 -1.124000
H 3.329000 3.065000 0.703000
C 1.685000 4.002000 -2.681000

H	0.040000	2.751000	-2.057000
C	2.939000	4.516000	-2.348000
H	4.499000	4.579000	-0.856000
H	1.229000	4.255000	-3.634000
H	3.459000	5.175000	-3.037000
C	-0.831000	-1.902000	-2.501000
C	-1.517000	-3.127000	-2.491000
C	-1.069000	-1.006000	-3.560000
C	-2.379000	-3.468000	-3.535000
H	-1.383000	-3.824000	-1.671000
C	-1.919000	-1.358000	-4.609000
H	-0.600000	-0.029000	-3.563000
C	-2.573000	-2.592000	-4.603000
H	-2.895000	-4.424000	-3.511000
H	-2.074000	-0.662000	-5.428000
H	-3.233000	-2.865000	-5.421000
C	0.824000	-2.973000	-0.326000
C	1.056000	-3.079000	1.052000
C	1.209000	-4.038000	-1.161000
C	1.627000	-4.237000	1.589000
H	0.821000	-2.243000	1.698000
C	1.770000	-5.195000	-0.622000
H	1.075000	-3.967000	-2.235000
C	1.975000	-5.301000	0.757000
H	1.807000	-4.302000	2.659000
H	2.054000	-6.010000	-1.280000
H	2.412000	-6.203000	1.175000
O	5.038000	0.825000	-0.907000
O	5.390000	0.707000	-3.193000
O	4.538000	-1.847000	0.728000
O	4.822000	-1.799000	3.026000
C	5.942000	1.187000	-1.960000
H	6.034000	2.278000	-2.003000
H	6.913000	0.713000	-1.787000
C	5.329000	-2.361000	1.806000
H	5.235000	-3.450000	1.840000
H	6.374000	-2.057000	1.672000

I-4^{R}HM^{c}

HF(M06/6-311++G(d,p) and SDD, SMD[1,4Dioxane])= -3453,11254597

C	-1.403000	-2.424000	1.969000
O	-0.538000	-1.490000	1.802000
N	-1.064000	-3.445000	2.783000
C	0.128000	-3.348000	3.630000
H	-0.125000	-3.705000	4.634000
H	0.936000	-3.974000	3.233000
H	0.470000	-2.317000	3.680000
C	-1.689000	-4.770000	2.778000
H	-0.890000	-5.519000	2.794000
H	-2.320000	-4.923000	3.661000
H	-2.279000	-4.916000	1.876000
C	-2.695000	-2.281000	1.285000
C	-2.721000	-1.304000	0.242000
C	-3.336000	3.227000	0.059000
C	-3.863000	-2.924000	1.740000
C	-5.076000	-2.662000	1.121000
C	-5.112000	-1.771000	0.050000
C	-3.967000	-1.098000	-0.405000
H	-3.843000	-3.578000	2.603000
H	-5.987000	-3.136000	1.474000
H	-6.062000	-1.558000	-0.433000
C	-2.999000	1.772000	-0.338000
O	-1.728000	1.496000	-0.850000
C	-4.150000	1.350000	-1.272000
C	-4.870000	3.155000	0.272000
C	-5.346000	1.903000	-0.497000
C	-4.154000	-0.160000	-1.578000
H	-3.357000	-0.338000	-2.302000
H	-5.101000	-0.398000	-2.074000
C	-4.102000	2.119000	-2.618000
H	-4.293000	3.190000	-2.502000
H	-3.118000	1.998000	-3.078000
H	-4.862000	1.721000	-3.298000
O	-6.467000	1.441000	-0.468000
H	-5.140000	3.026000	1.327000
H	-5.408000	4.043000	-0.078000
H	-3.071000	3.915000	-0.750000
H	-2.793000	3.544000	0.955000
H	-3.147000	1.209000	0.615000
Ir	-1.024000	-0.064000	0.290000
P	0.259000	-0.946000	-1.371000
P	0.669000	1.471000	1.140000
C	1.754000	0.034000	-1.845000
C	2.797000	0.303000	-0.896000
C	1.922000	0.416000	-3.185000
C	3.894000	0.988000	-1.396000
C	3.055000	1.099000	-3.656000
H	1.166000	0.164000	-3.914000
C	4.026000	1.389000	-2.725000
H	3.156000	1.380000	-4.698000
C	2.125000	0.465000	1.611000
C	2.887000	-0.102000	0.543000
C	2.408000	0.146000	2.941000
C	3.895000	-0.973000	0.926000
C	3.438000	-0.747000	3.295000
H	1.825000	0.602000	3.734000
C	4.159000	-1.299000	2.258000
H	3.662000	-0.979000	4.331000
C	-0.066000	2.164000	2.683000
C	-0.047000	3.540000	2.960000
C	-0.751000	1.305000	3.566000
C	-0.685000	4.042000	4.096000
H	0.464000	4.224000	2.292000
C	-1.381000	1.811000	4.703000
H	-0.779000	0.238000	3.367000
C	-1.352000	3.182000	4.970000
H	-0.659000	5.109000	4.297000
H	-1.898000	1.135000	5.378000
H	-1.847000	3.576000	5.853000

C	1.362000	2.930000	0.266000
C	2.569000	3.505000	0.699000
C	0.667000	3.501000	-0.811000
C	3.072000	4.640000	0.062000
H	3.118000	3.067000	1.527000
C	1.181000	4.634000	-1.447000
H	-0.265000	3.048000	-1.137000
C	2.378000	5.205000	-1.013000
H	4.000000	5.086000	0.408000
H	0.642000	5.072000	-2.282000
H	2.771000	6.089000	-1.508000
C	-0.799000	-1.106000	-2.865000
C	-1.356000	-2.349000	-3.211000
C	-1.146000	0.027000	-3.622000
C	-2.187000	-2.466000	-4.326000
H	-1.141000	-3.231000	-2.618000
C	-1.956000	-0.101000	-4.750000
H	-0.802000	1.008000	-3.319000
C	-2.472000	-1.347000	-5.110000
H	-2.604000	-3.435000	-4.582000
H	-2.198000	0.780000	-5.338000
H	-3.106000	-1.443000	-5.987000
C	1.038000	-2.612000	-1.153000
C	1.347000	-3.148000	0.104000
C	1.488000	-3.284000	-2.306000
C	2.054000	-4.350000	0.204000
H	1.057000	-2.614000	0.997000
C	2.186000	-4.485000	-2.200000
H	1.301000	-2.871000	-3.292000
C	2.464000	-5.027000	-0.943000
H	2.289000	-4.752000	1.186000
H	2.516000	-4.993000	-3.101000
H	3.008000	-5.965000	-0.862000
O	5.005000	1.375000	-0.695000
O	5.209000	2.036000	-2.904000
O	4.764000	-1.643000	0.111000
O	5.191000	-2.191000	2.320000
C	5.825000	2.114000	-1.610000
H	5.873000	3.162000	-1.293000
H	6.821000	1.665000	-1.654000
C	5.664000	-2.349000	0.975000
H	5.664000	-3.410000	0.710000
H	6.669000	-1.918000	0.889000

11. NMR spectra

1H NMR (300 MHz, CDCl3)

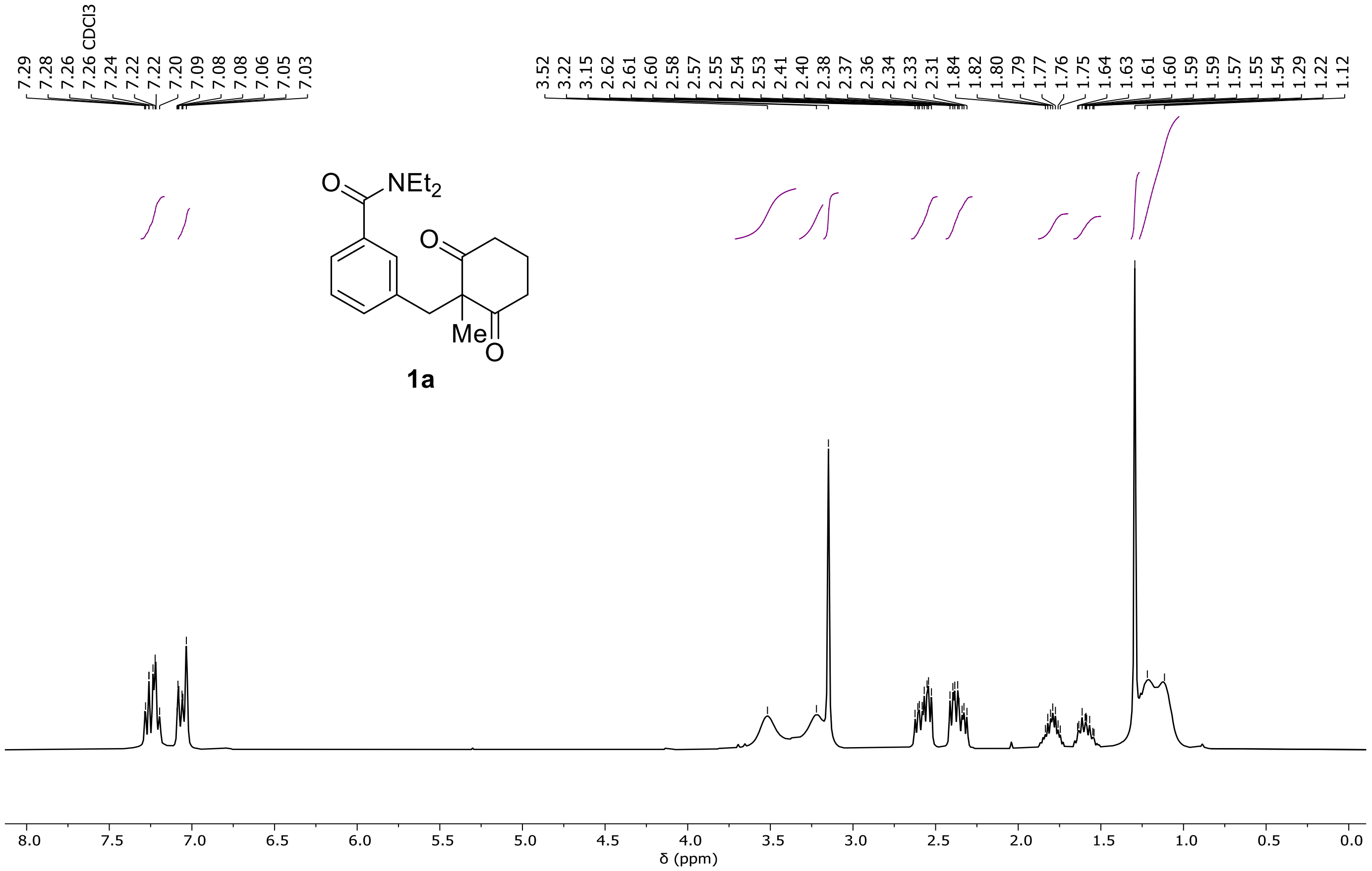

DEPT-135 NMR (75 MHz, CDCl3)

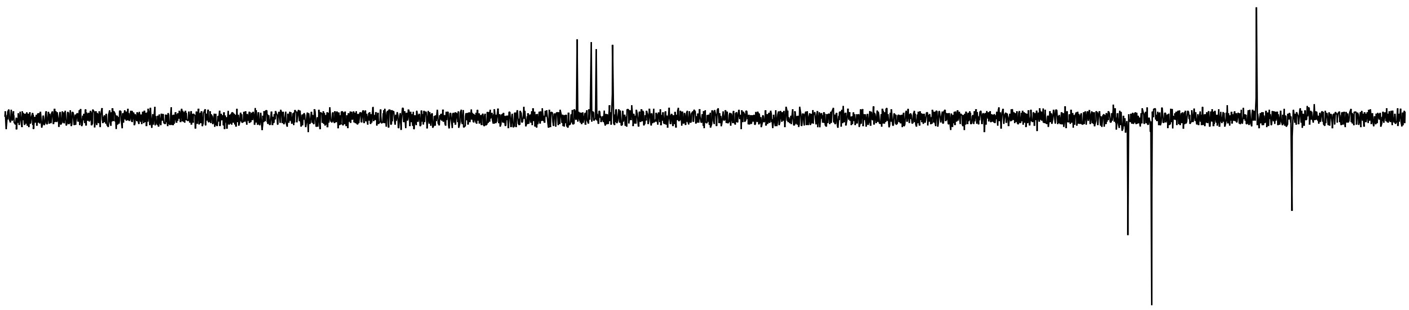

13C NMR (75 MHz, CDCl3)

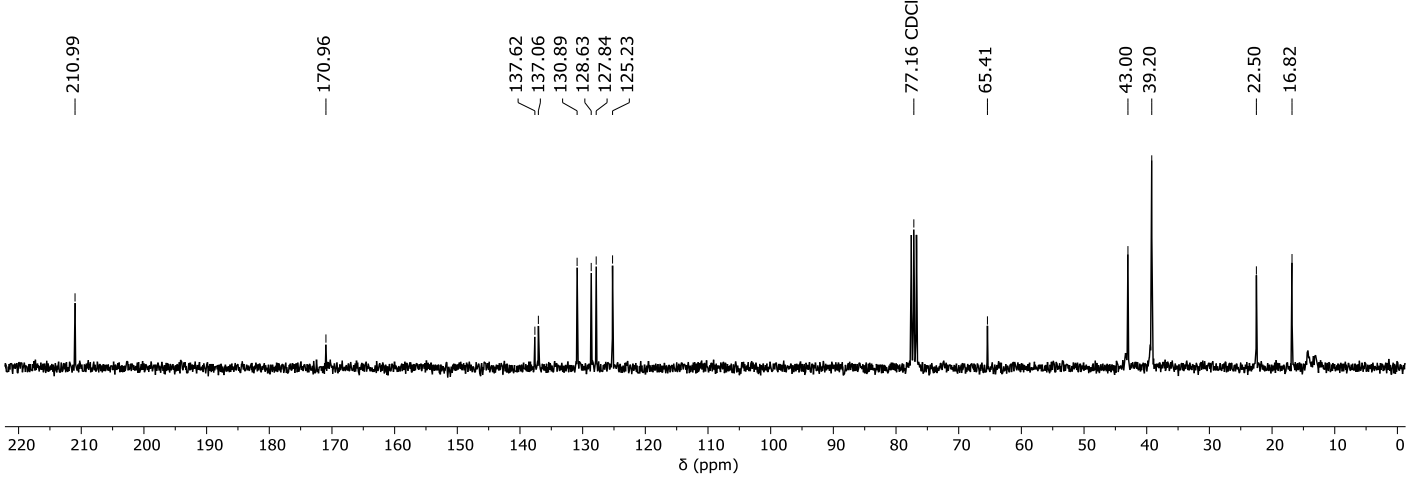

1H NMR (300 MHz, CDCl3)

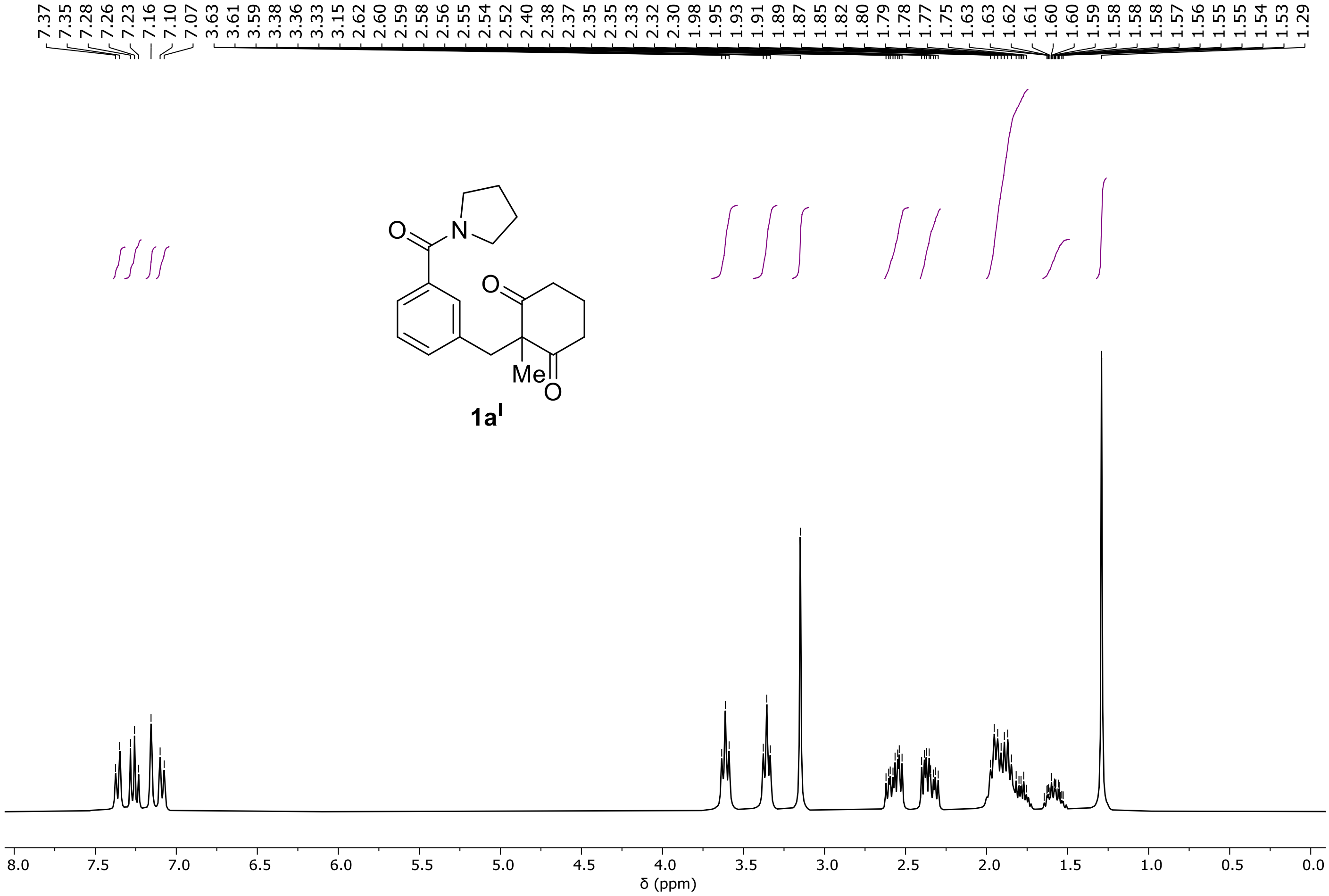

DEPT-135 NMR (75 MHz, CDCl3)

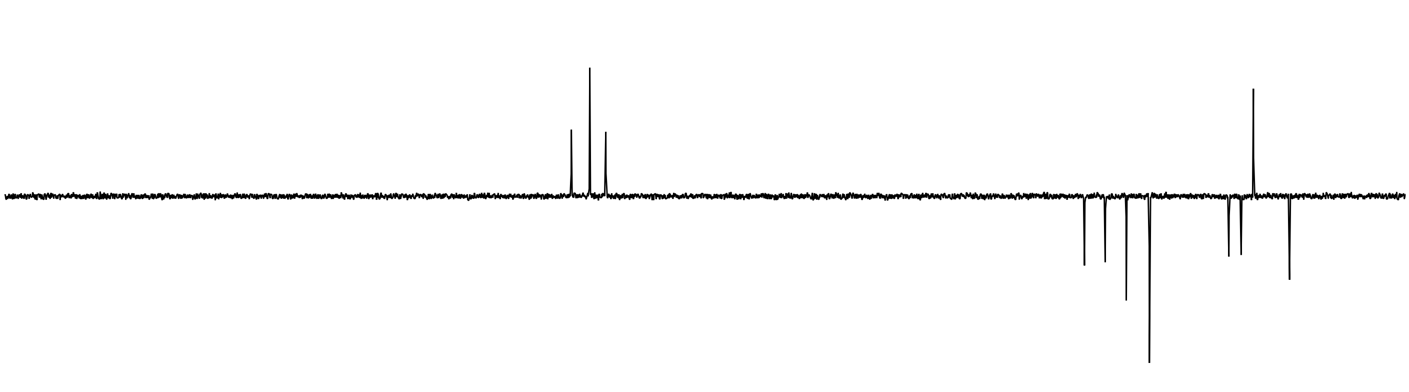

13C NMR (75 MHz, CDCl3)

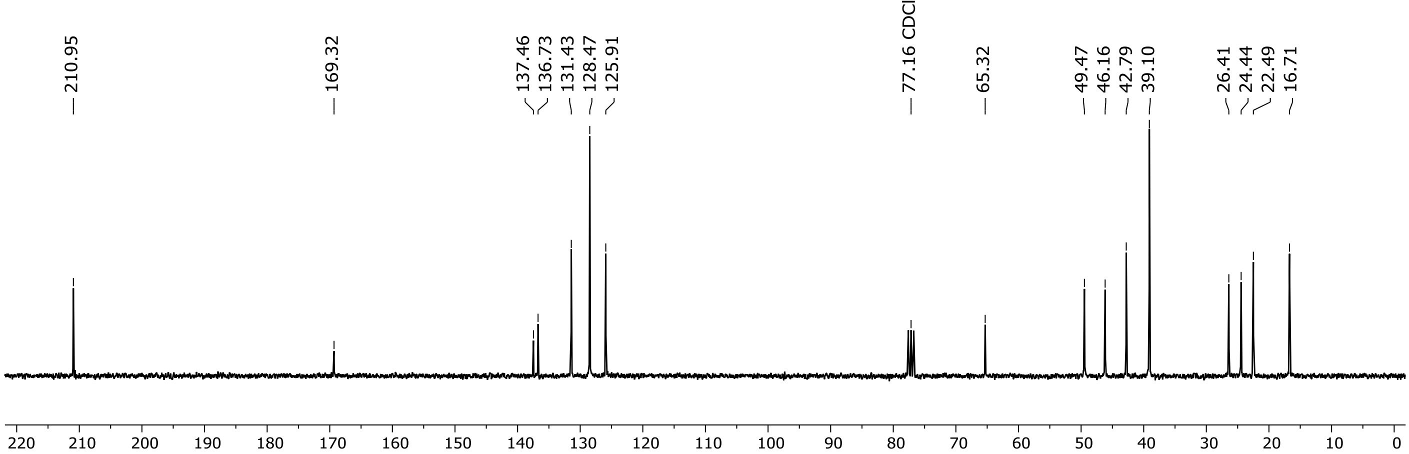

1H NMR (300 MHz, CDCl3)

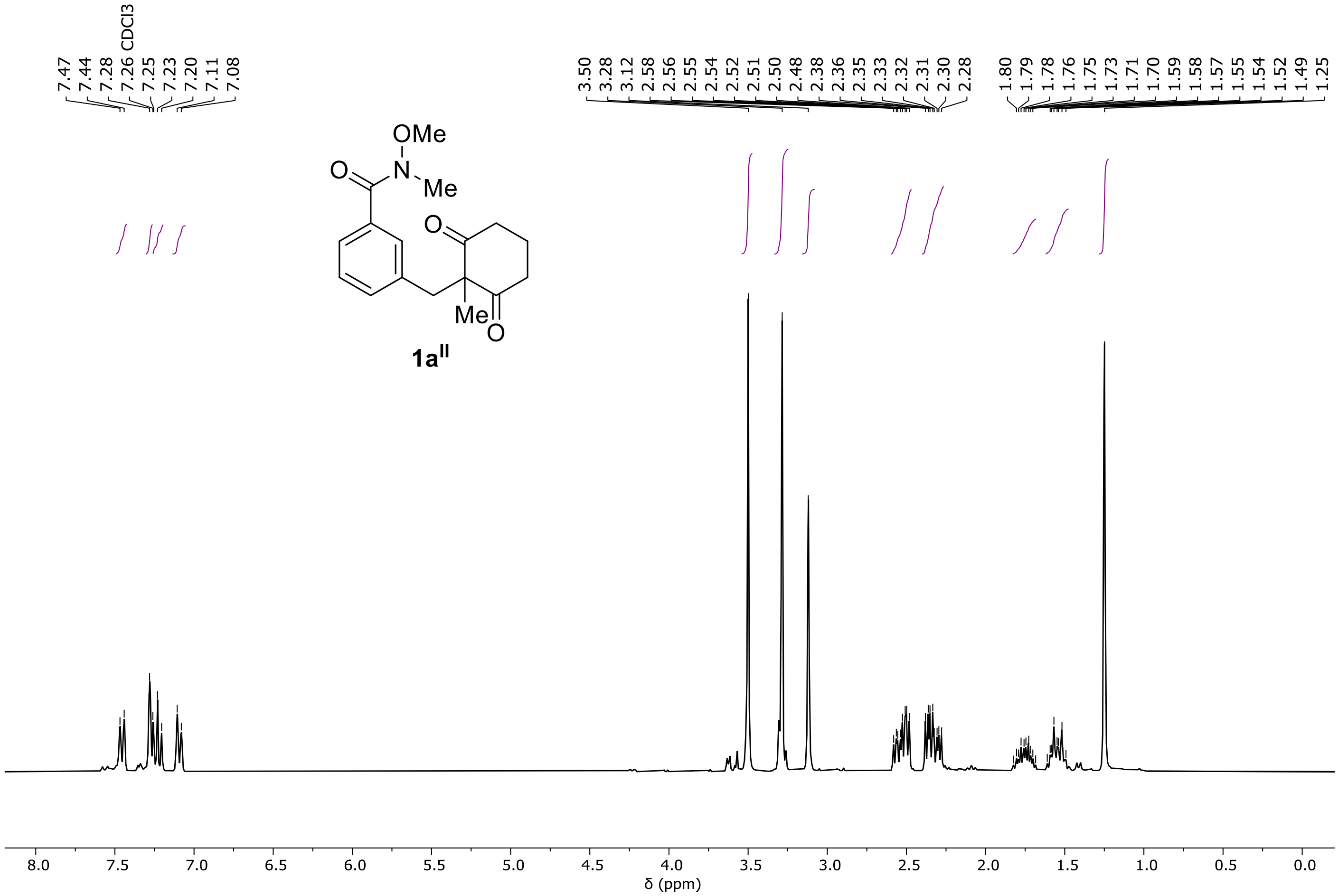

DEPT-135 NMR (75 MHz, CDCl3)

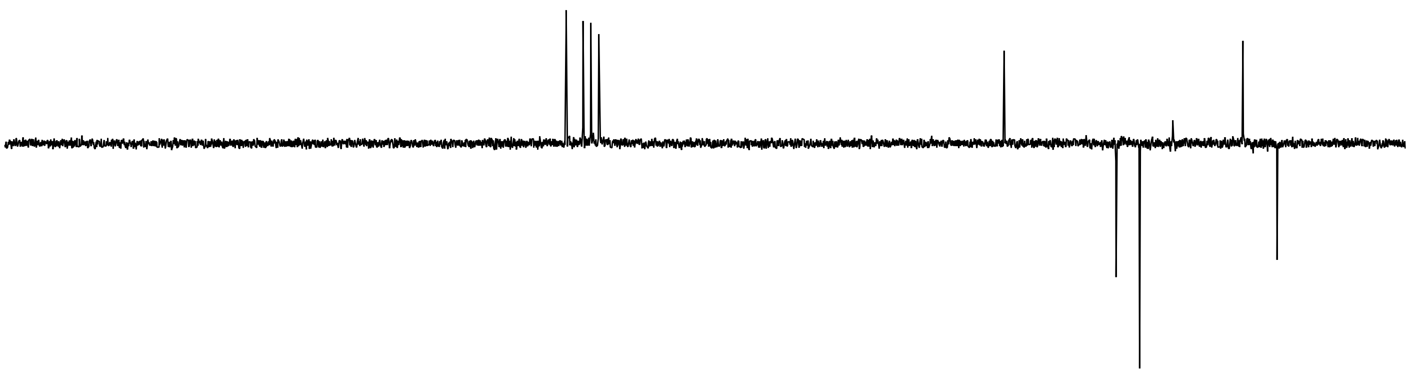

13C NMR (75 MHz, CDCl3)

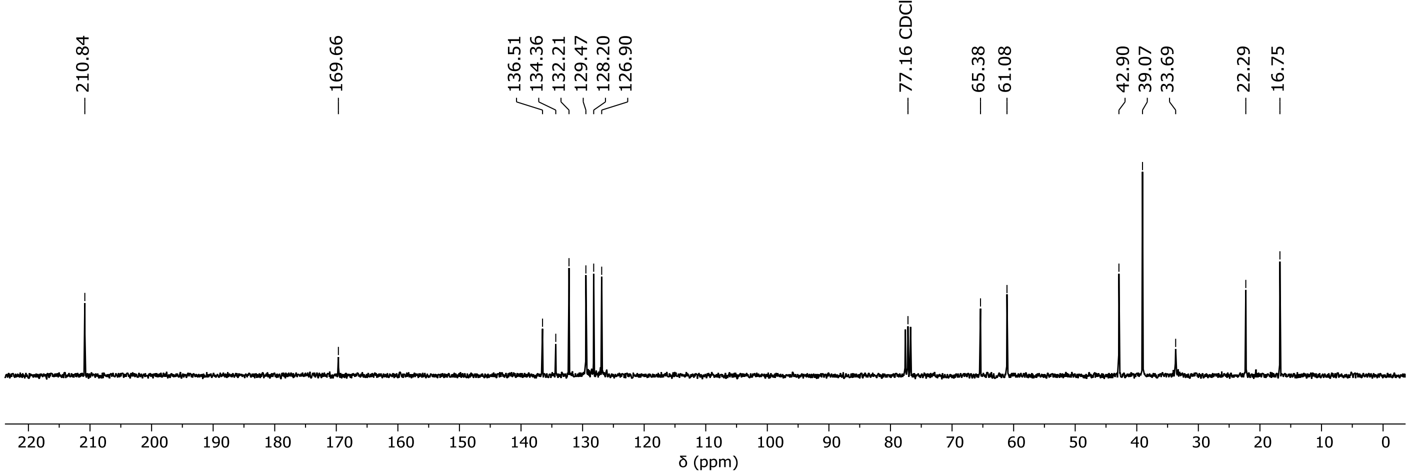

1H NMR (300 MHz, CDCl3)

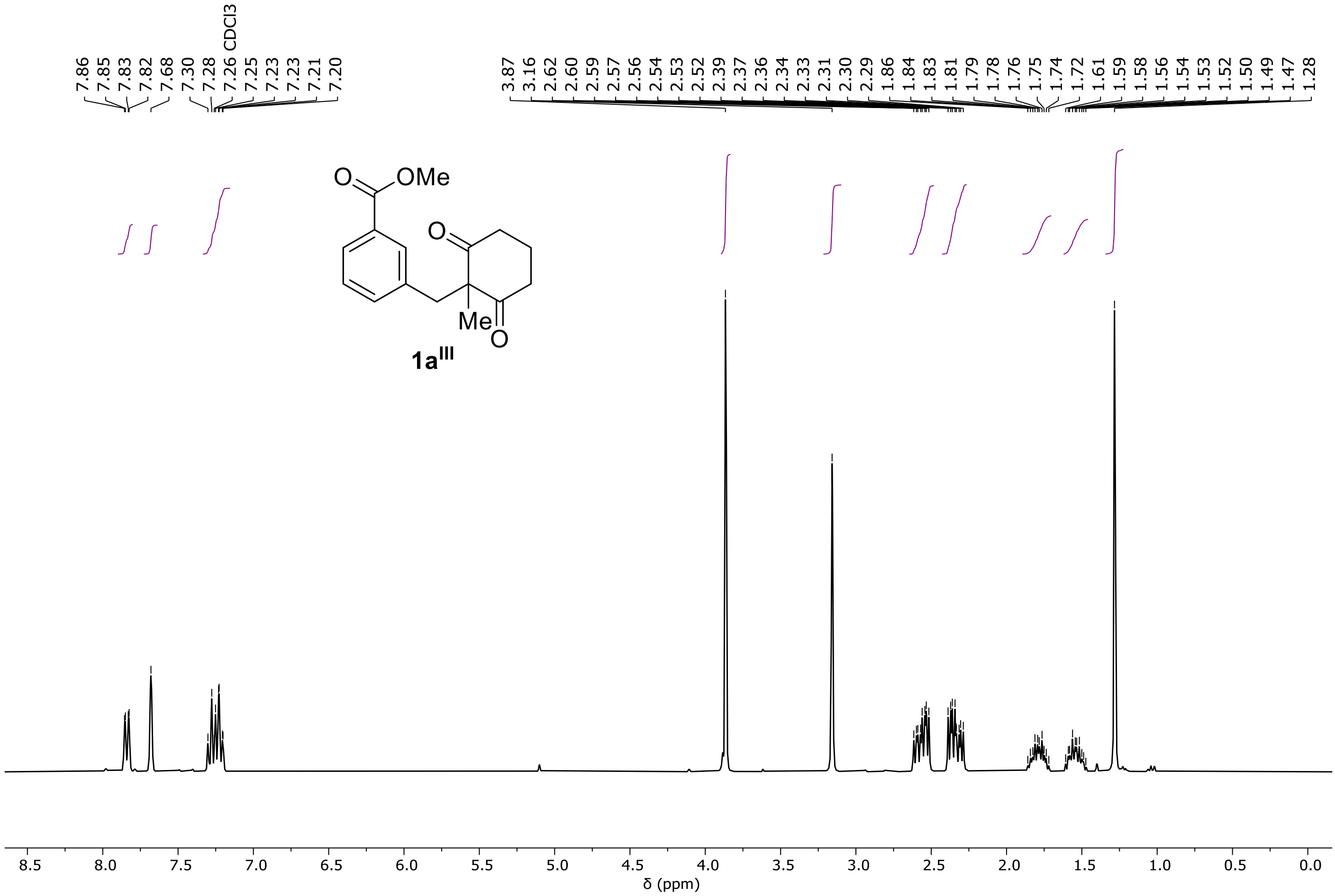

DEPT-135 NMR (75 MHz, CDCl3)

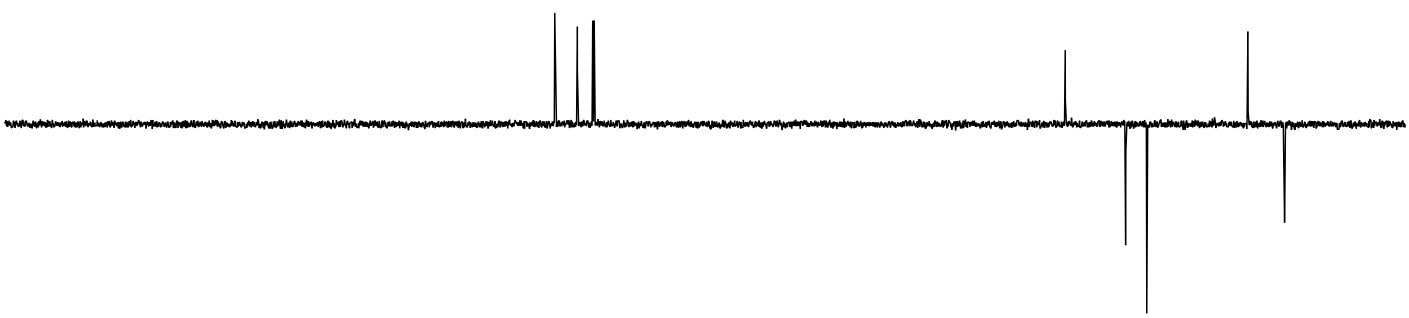

13C NMR (75 MHz, CDCl3)

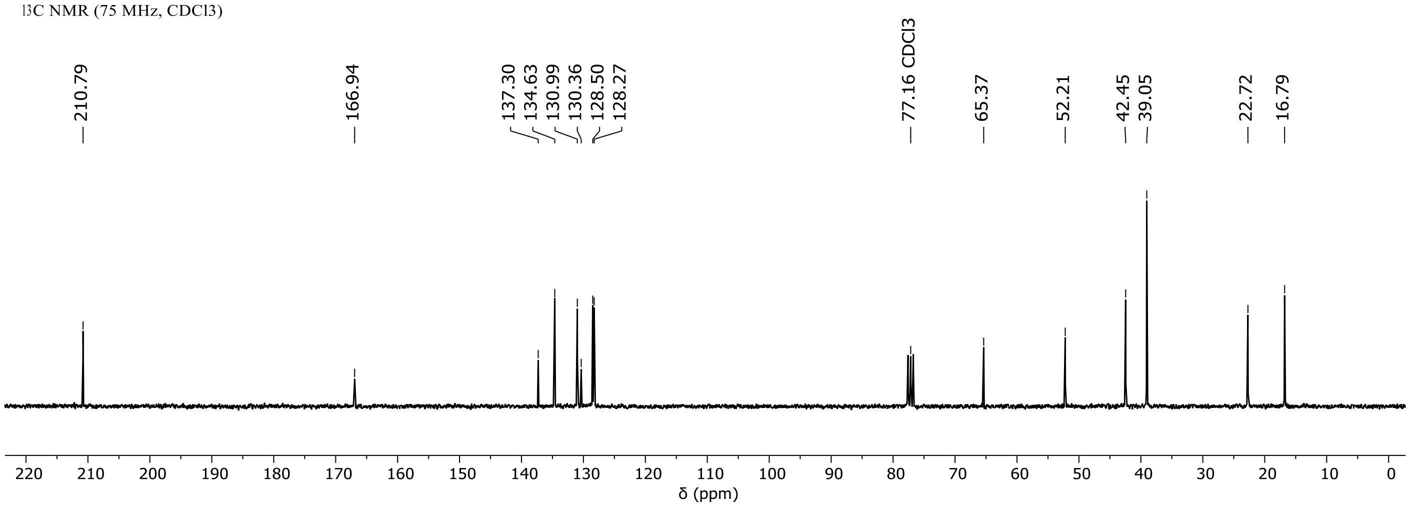

1H NMR (300 MHz, CDCl3)

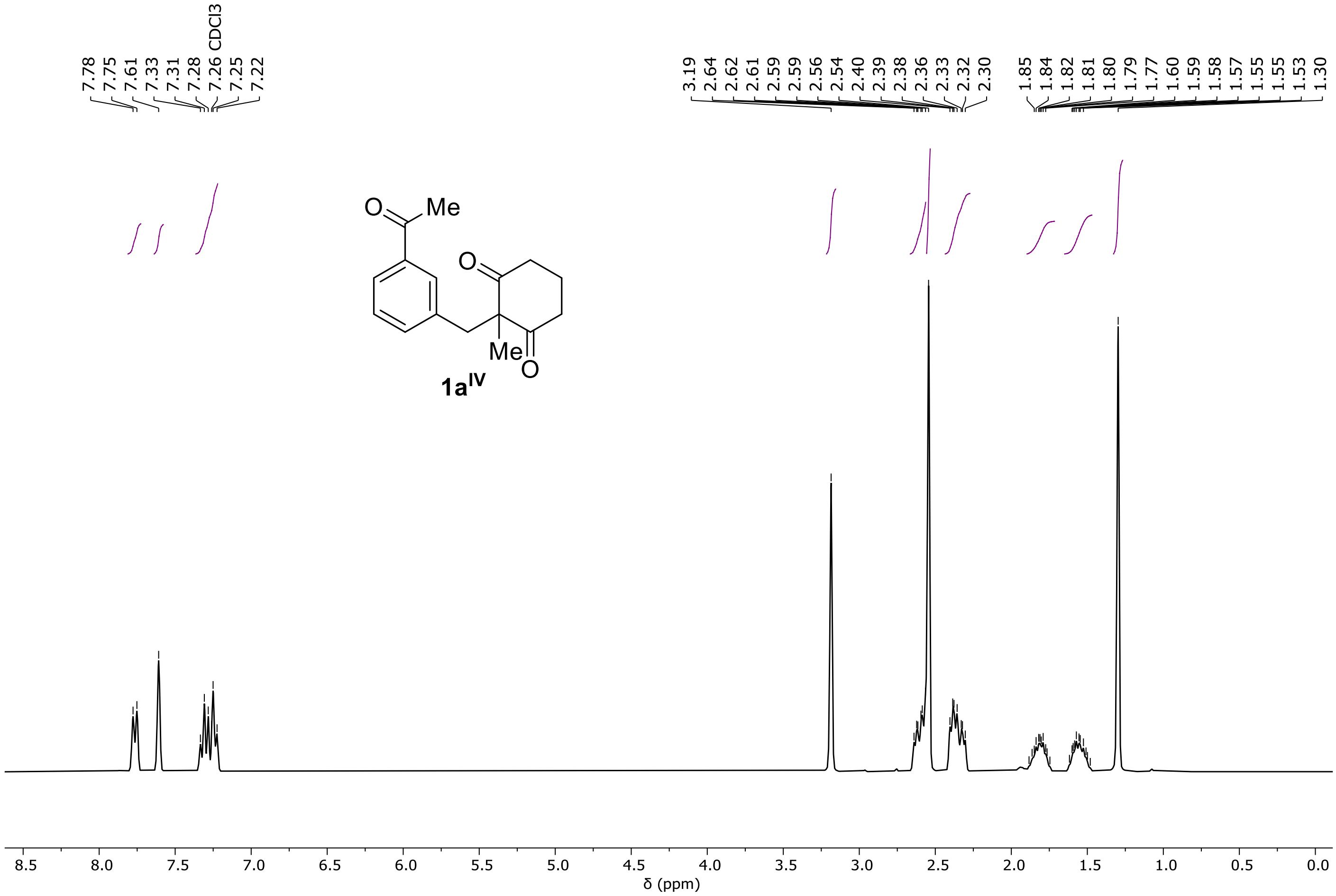

DEPT-135 NMR (75 MHz, CDCl3)

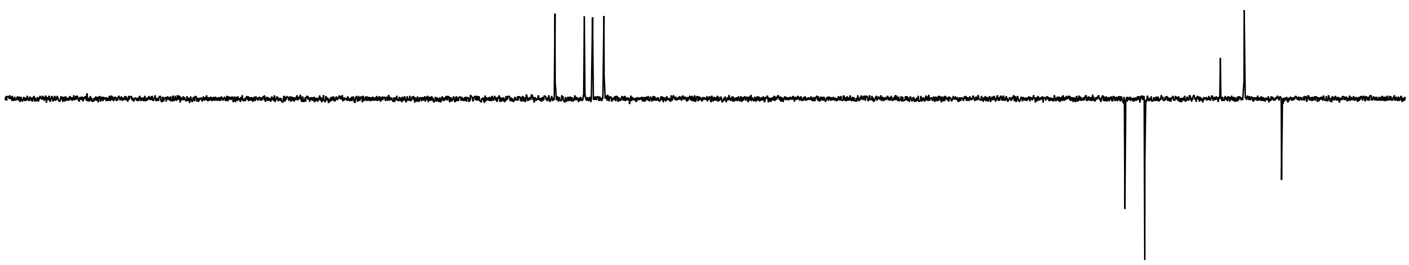

13C NMR (75 MHz, CDCl3)

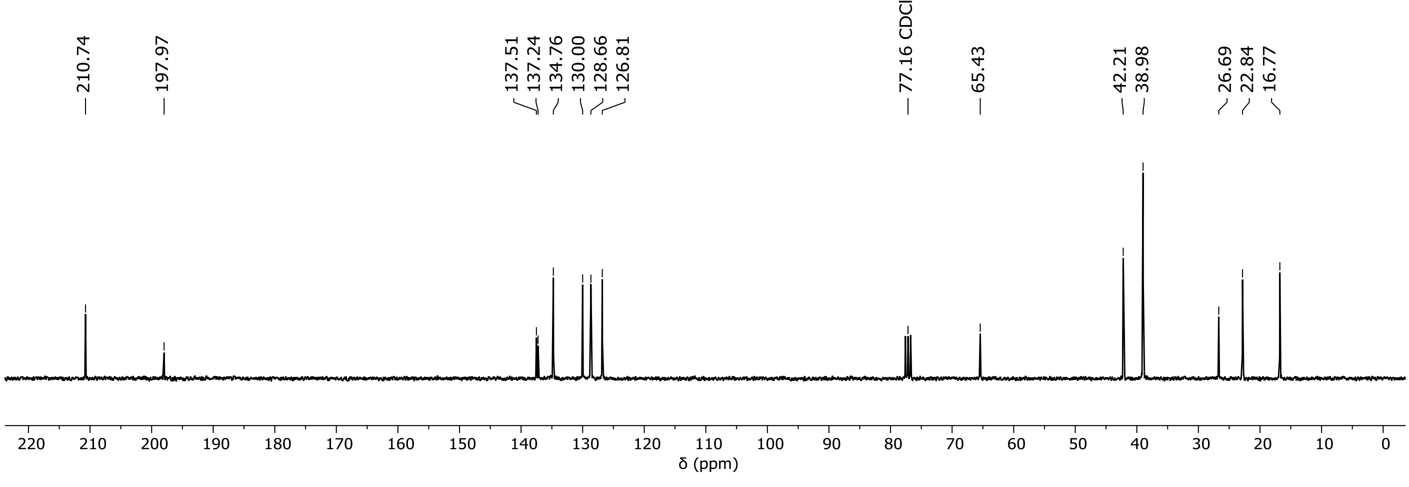

1H NMR (300 MHz, CDCl3)

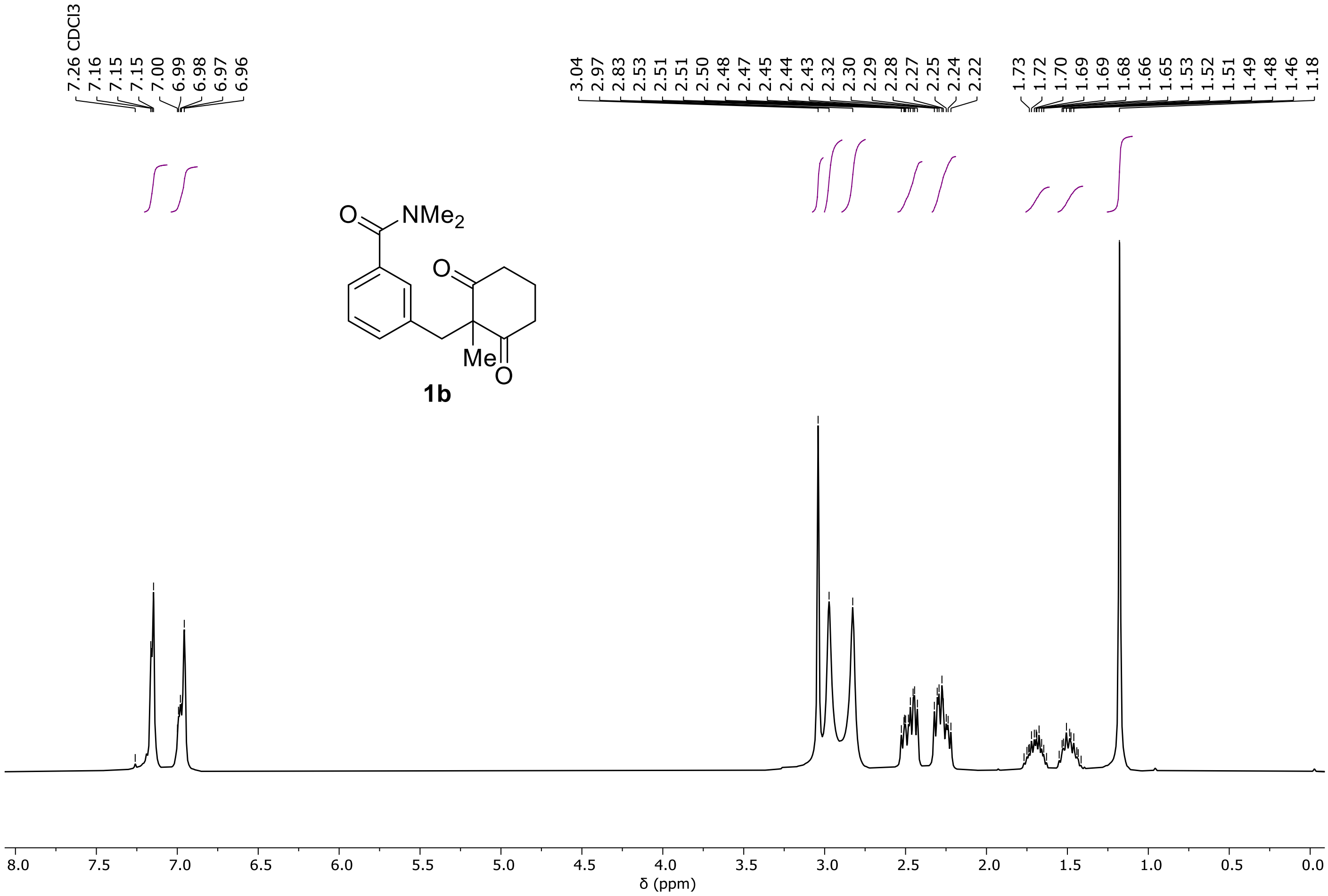

DEPT-135 NMR (75 MHz, CDCl3)

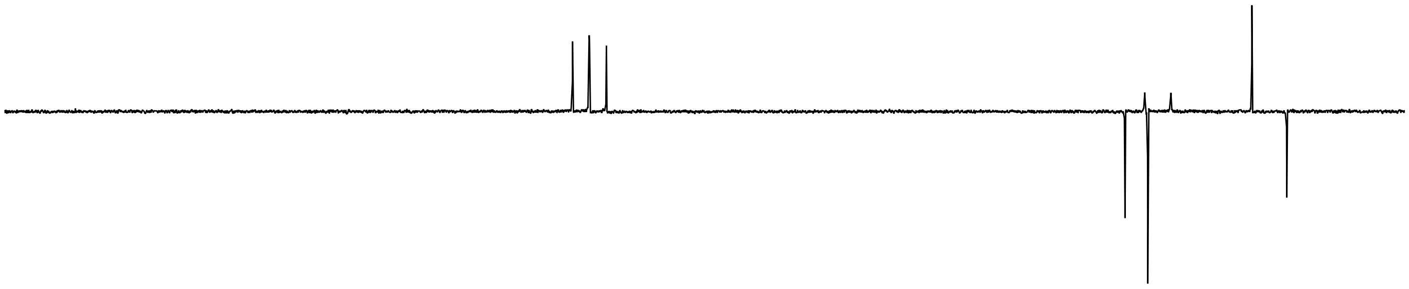

13C NMR (75 MHz, CDCl3)

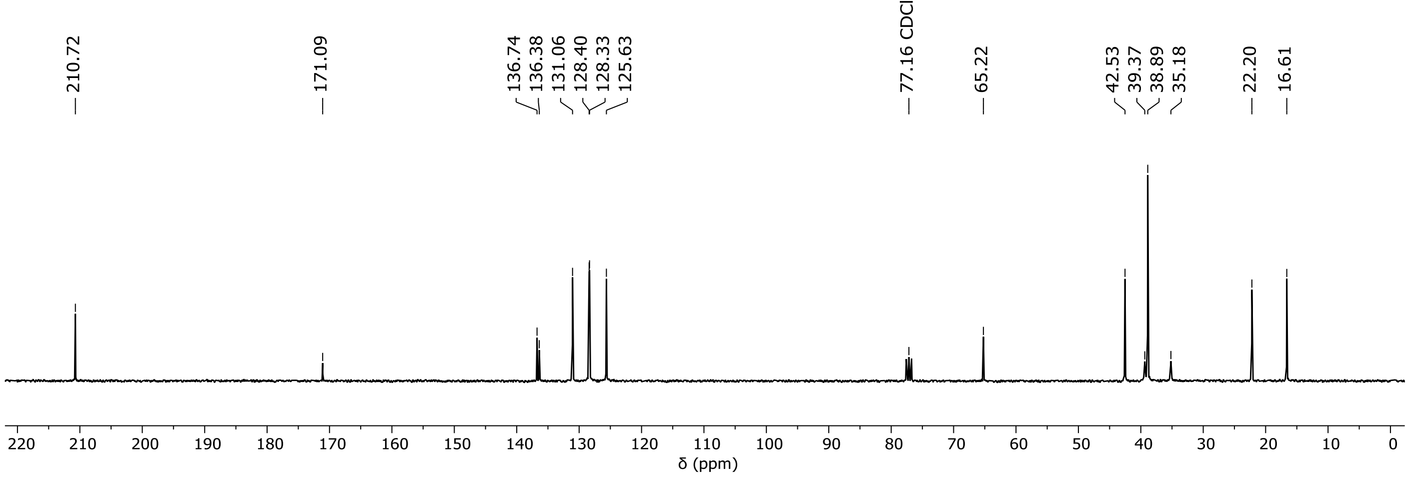

1H NMR (300 MHz, CDCl3)

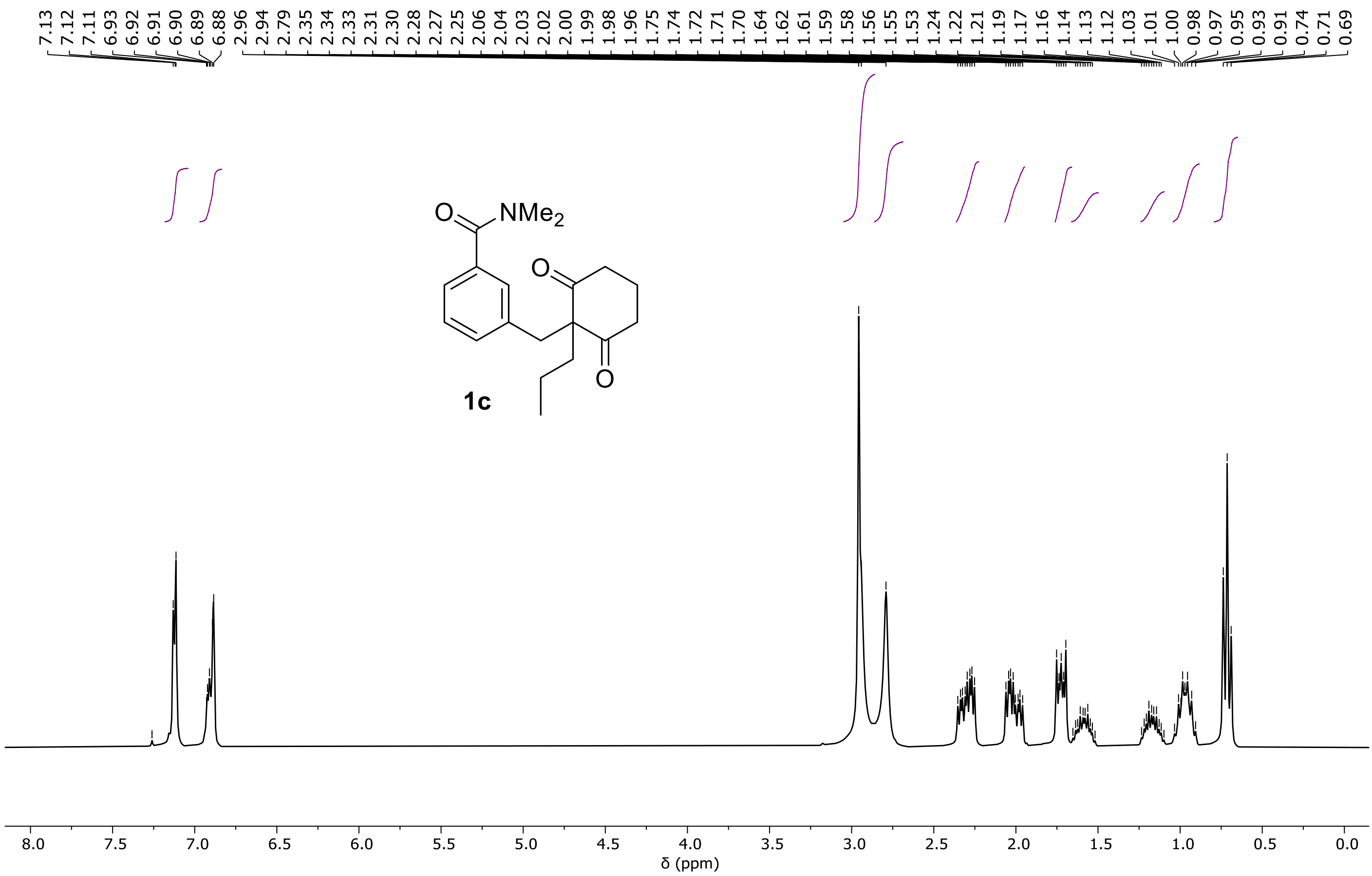

DEPT-135 NMR (75 MHz, CDCl3)

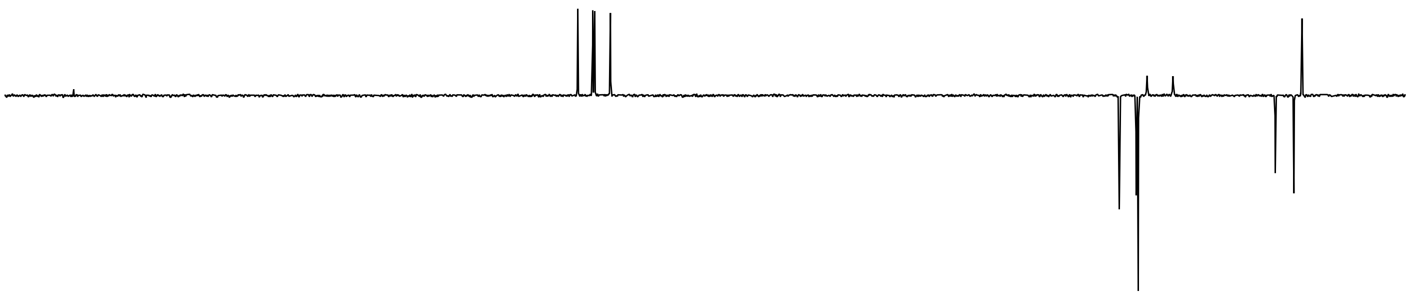

13C NMR (75 MHz, CDCl3)

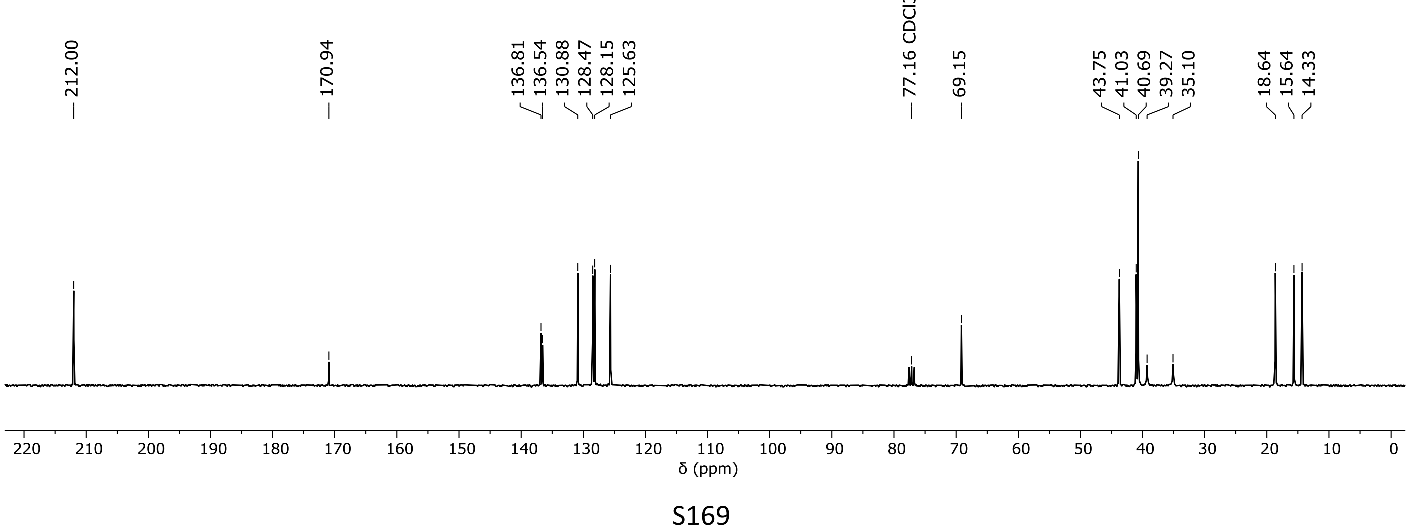

1H NMR (300 MHz, CDCl3)

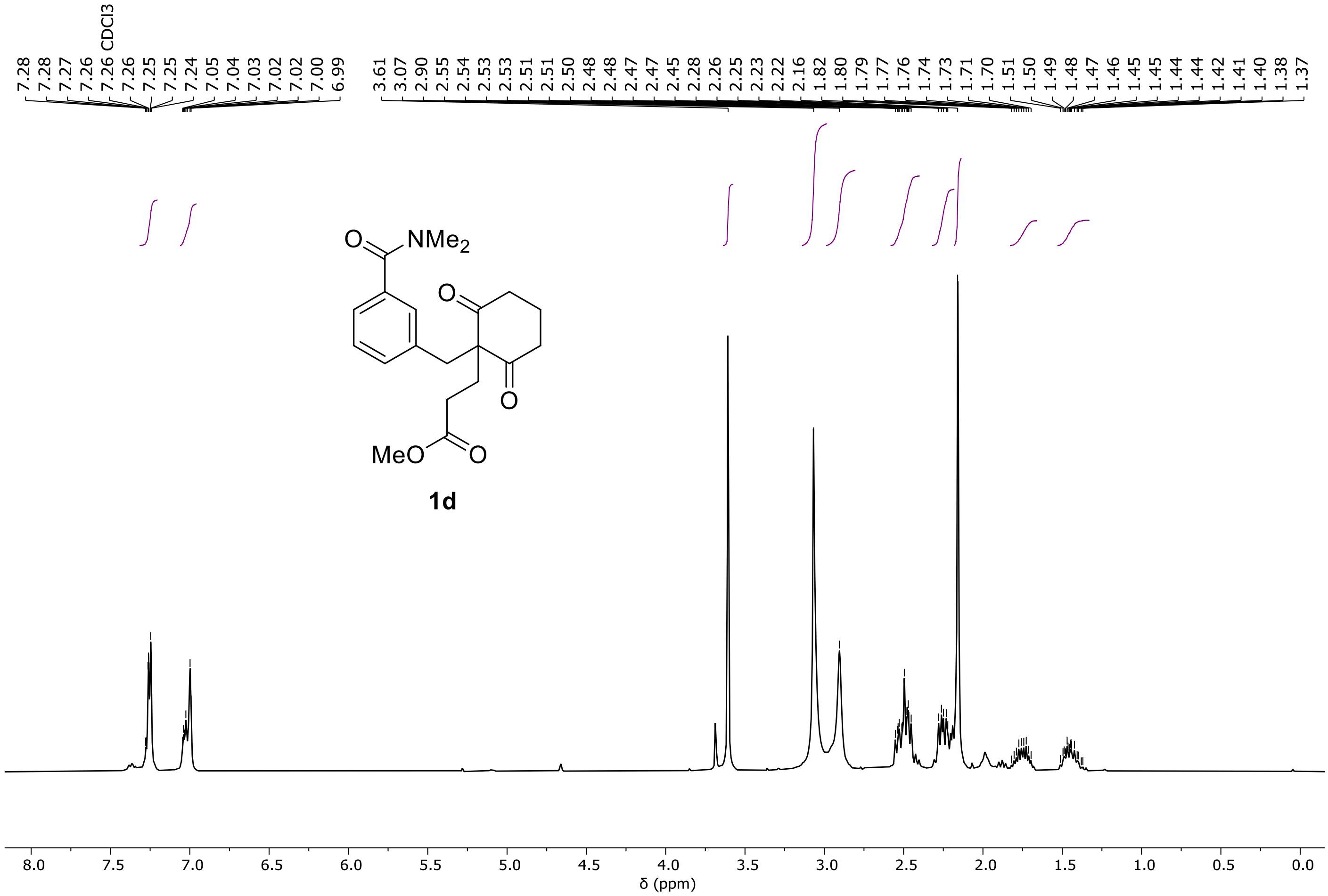

DEPT-135 NMR (75 MHz, CDCl3)

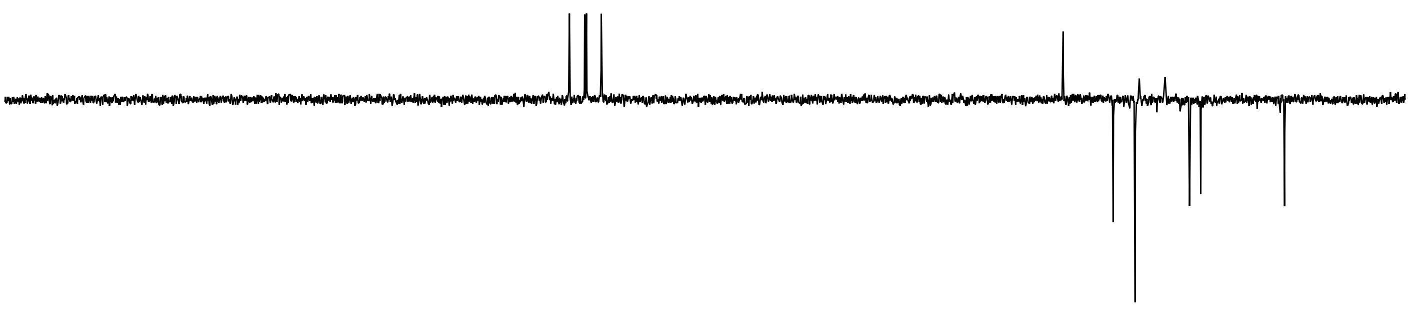

13C NMR (75 MHz, CDCl3)

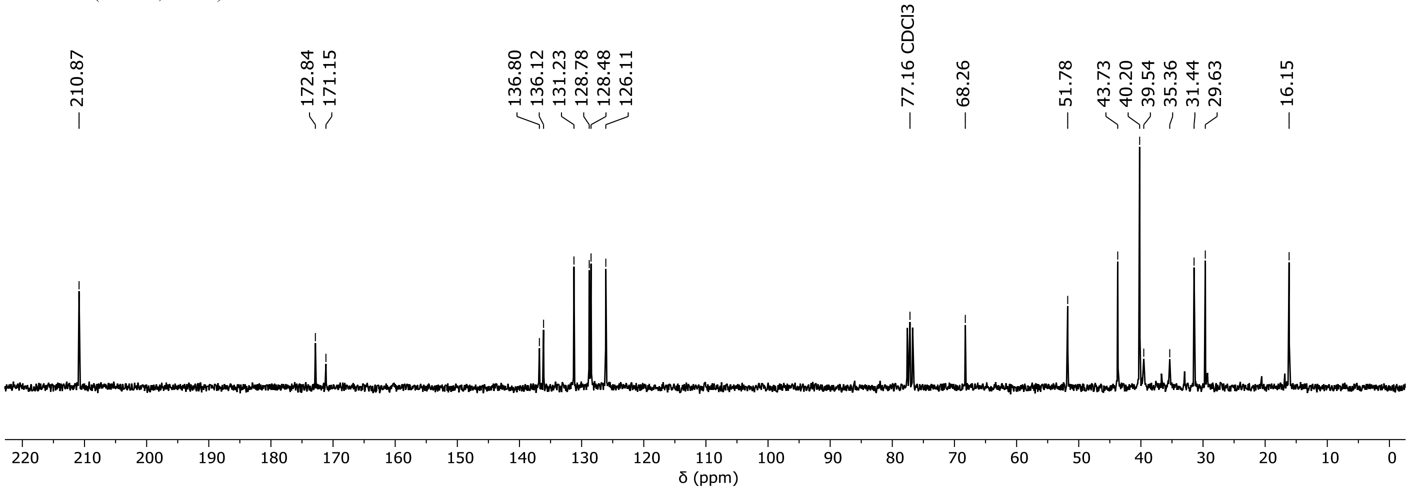

1H NMR (300 MHz, CDCl3)

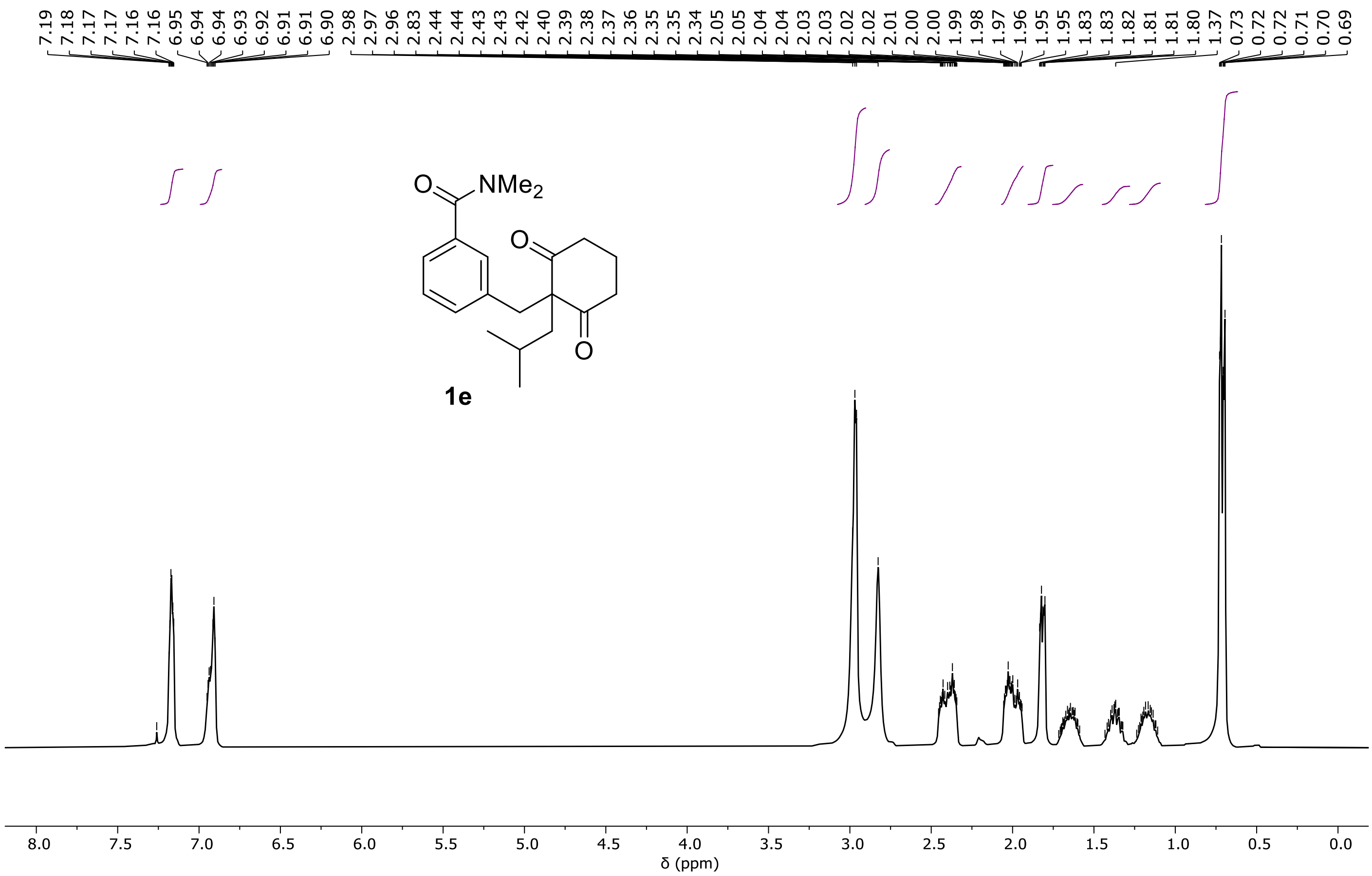

DEPT-135 NMR (75 MHz, CDCl3)

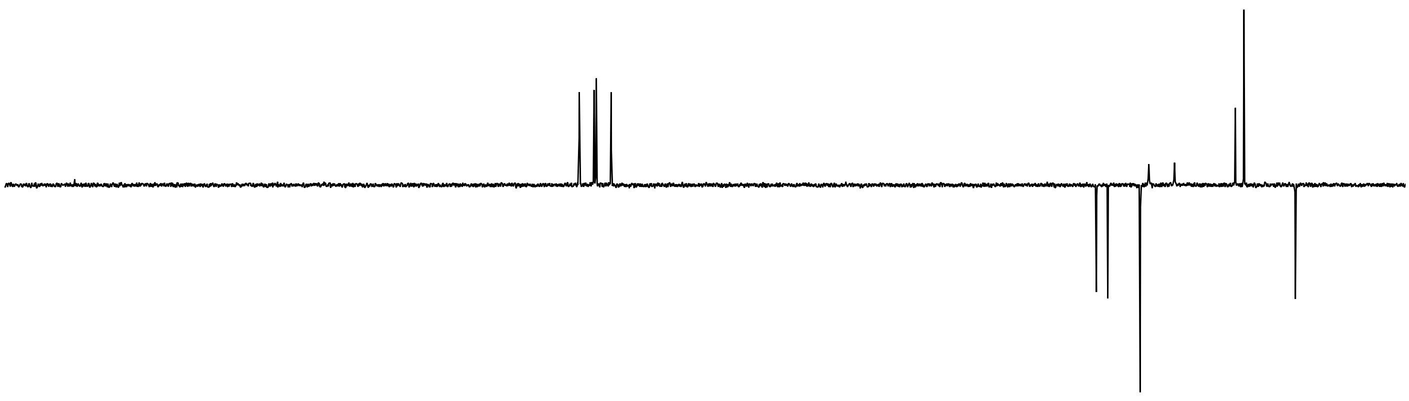

13C NMR (75 MHz, CDCl3)

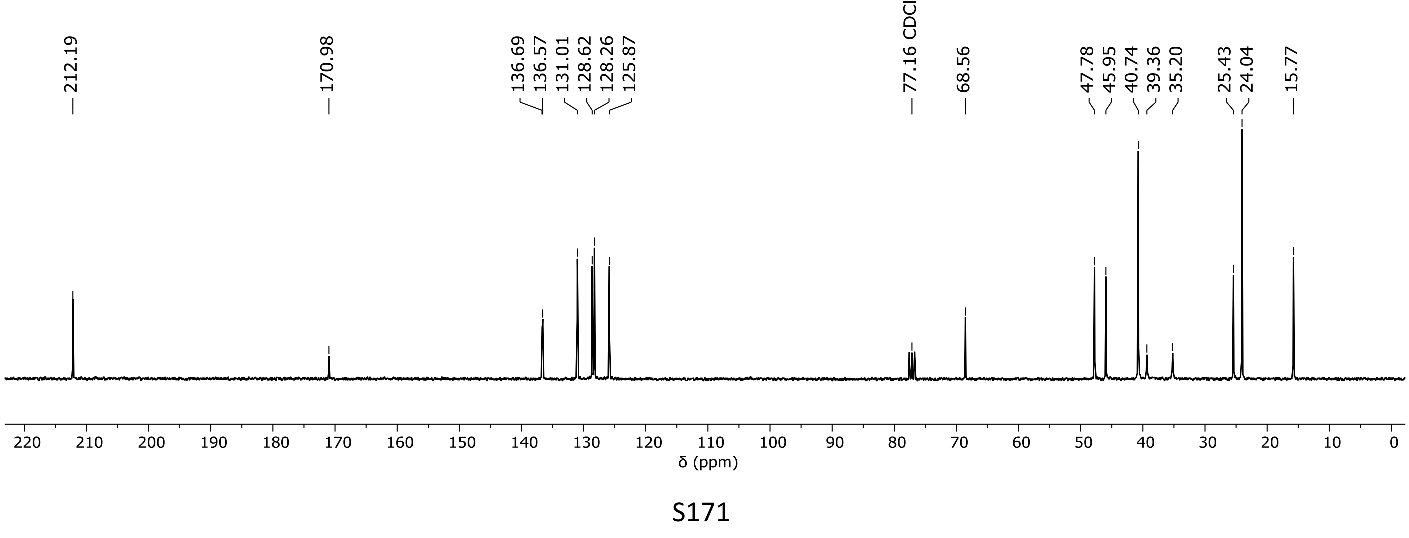

1H NMR (300 MHz, CDCl3)

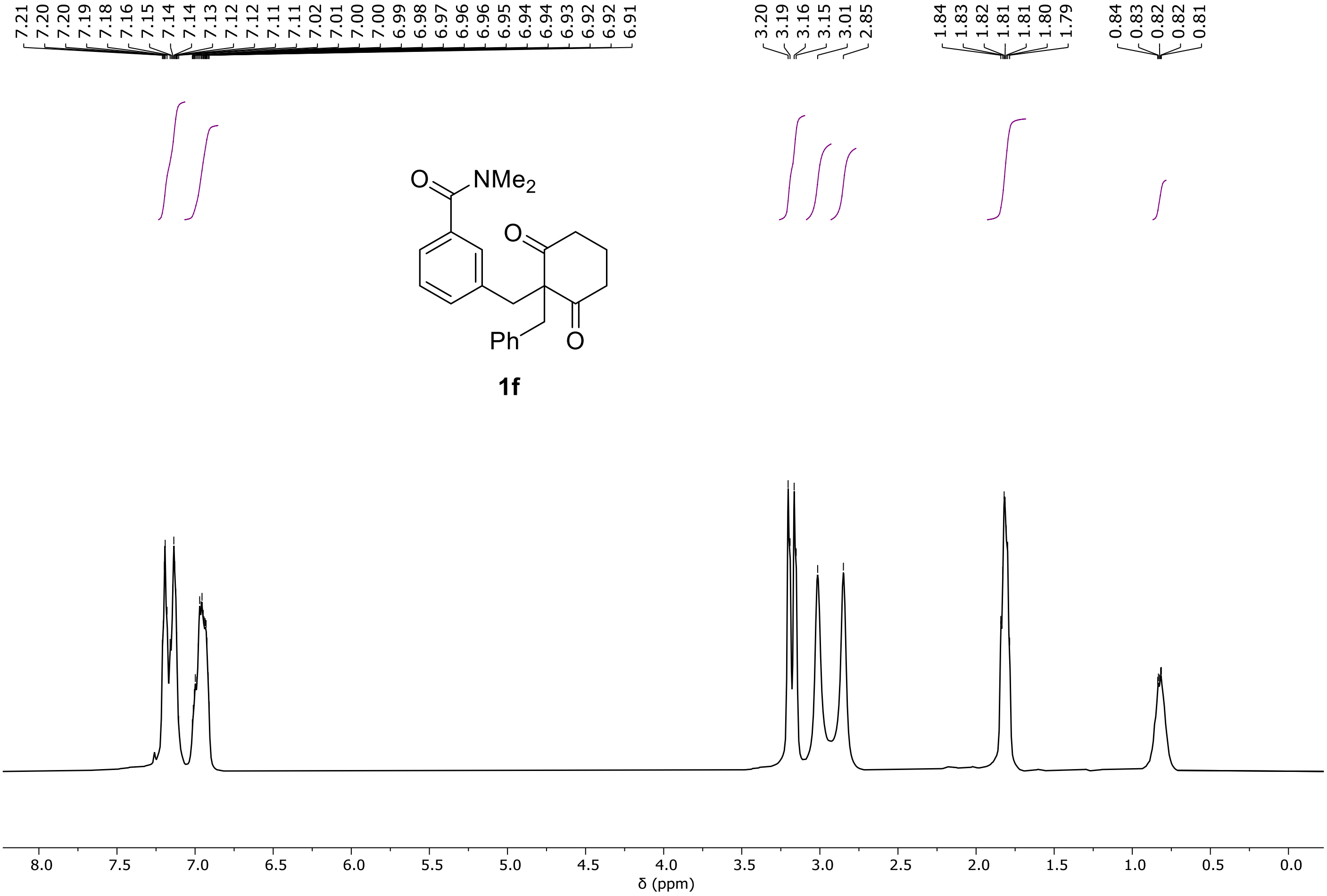

DEPT-135 NMR (75 MHz, CDCl3)

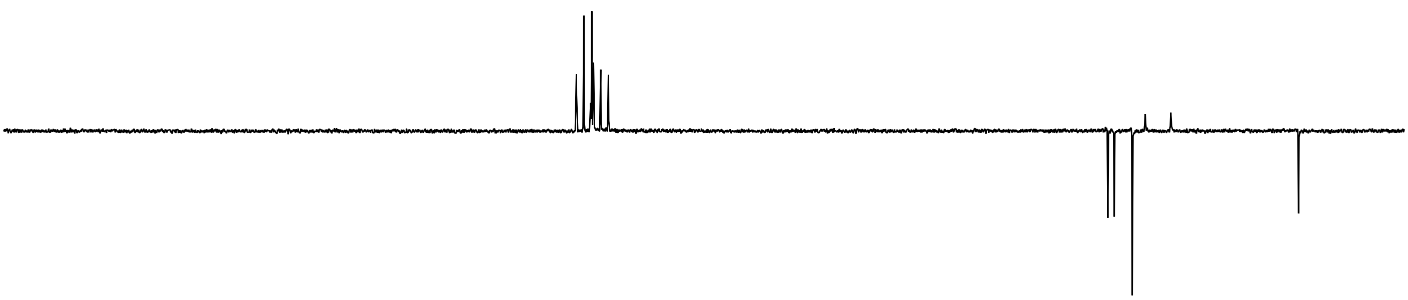

13C NMR (75 MHz, CDCl3)

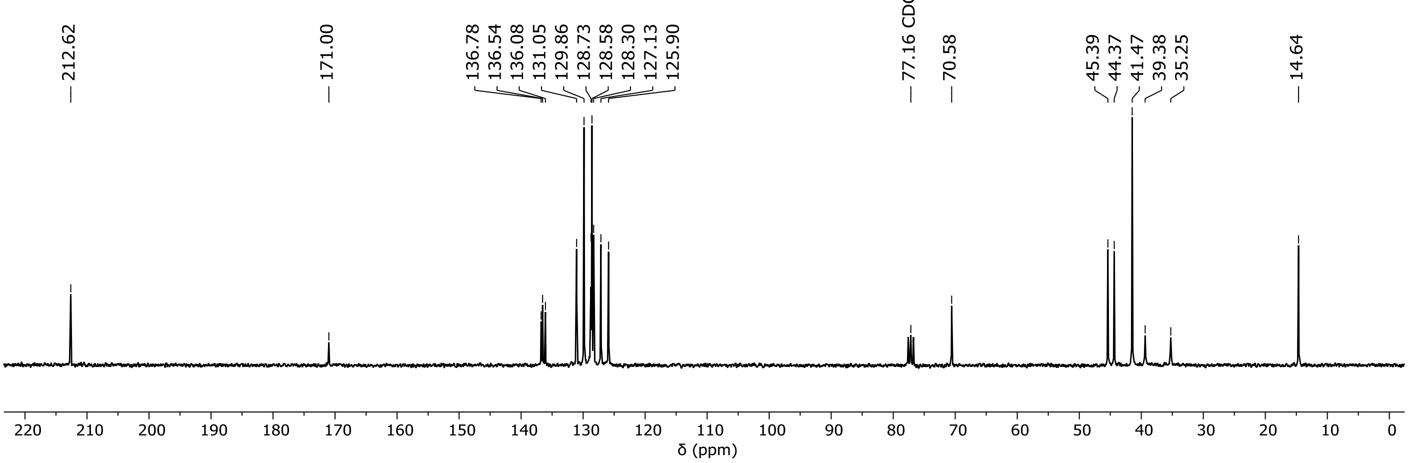

1H NMR (300 MHz, CDCl3)

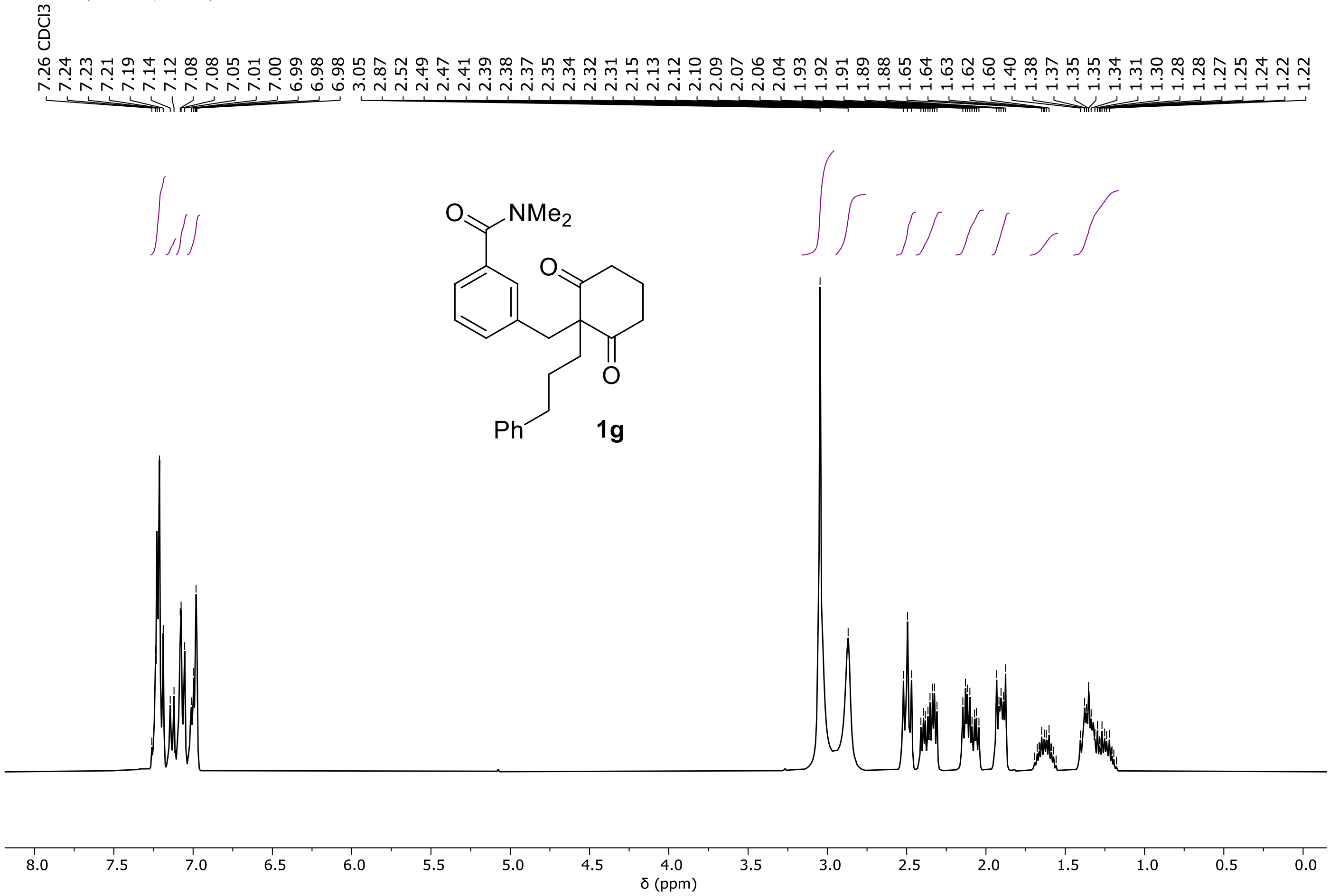

DEPT-135 NMR (75 MHz, CDCl3)

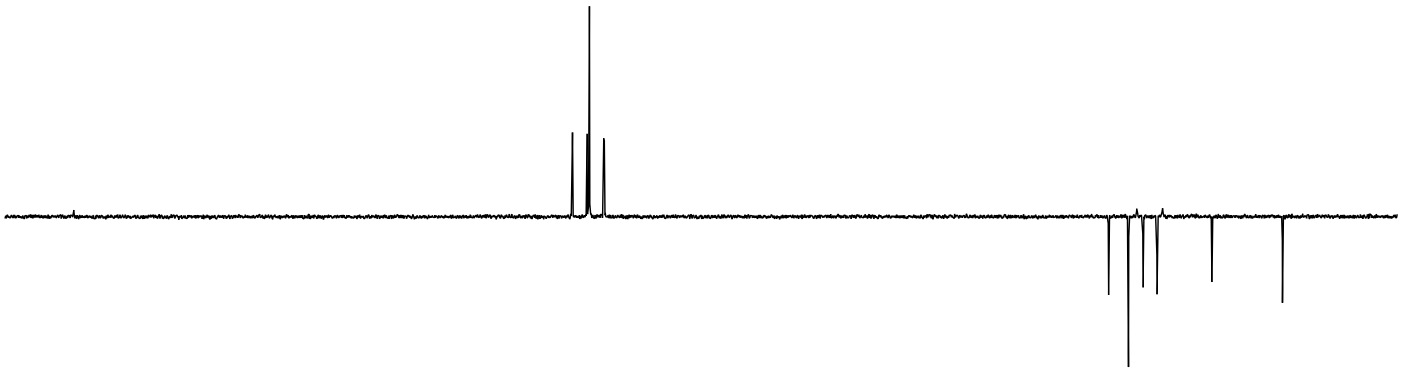

13C NMR (75 MHz, CDCl3)

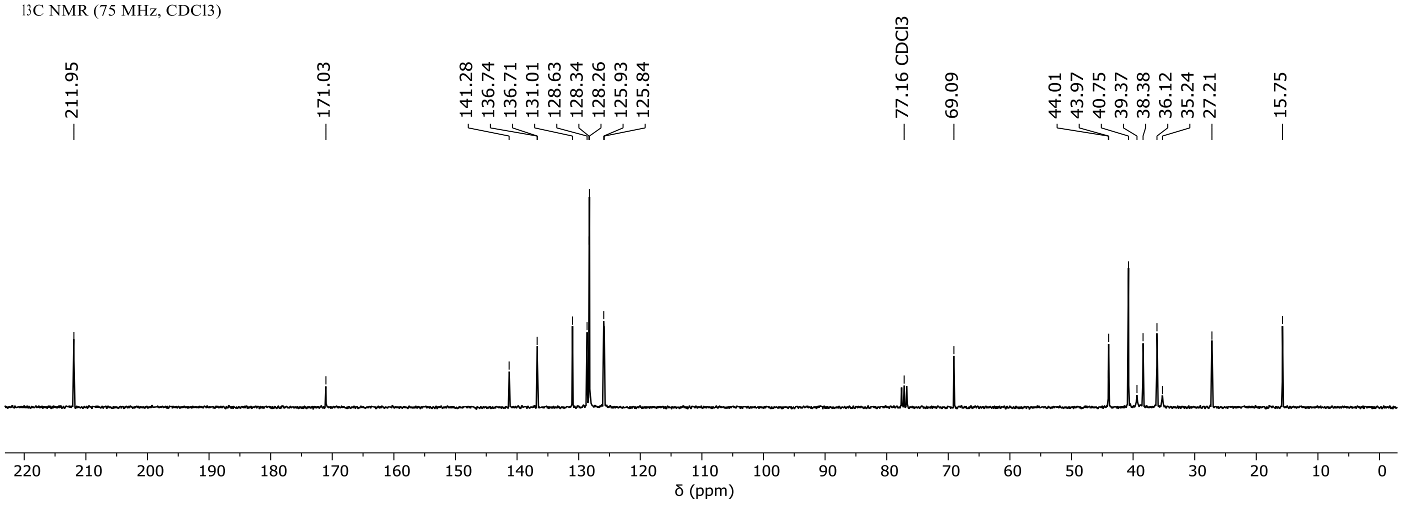

1H NMR (300 MHz, CDCl3)

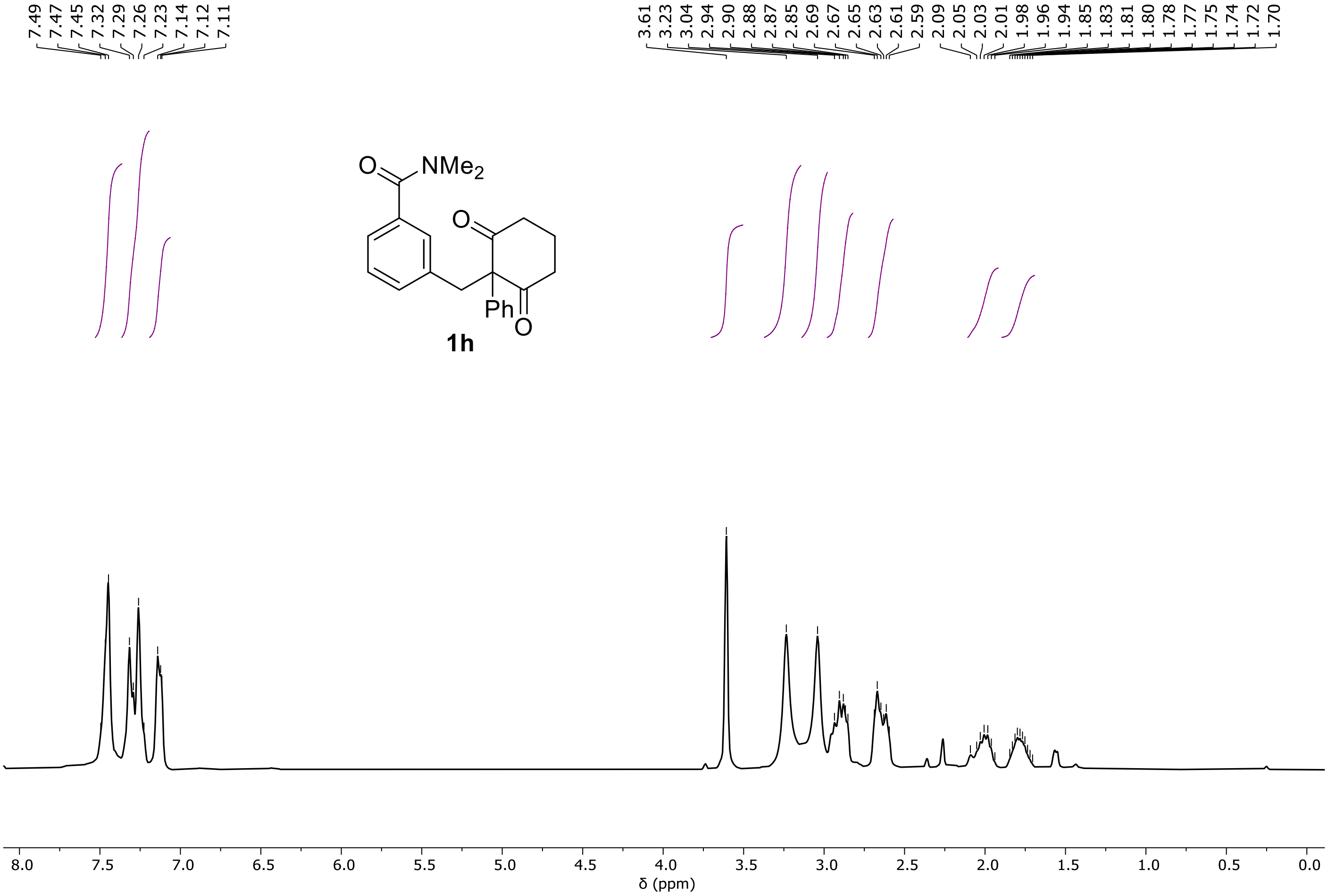

DEPT-135 NMR (75 MHz, CDCl3)

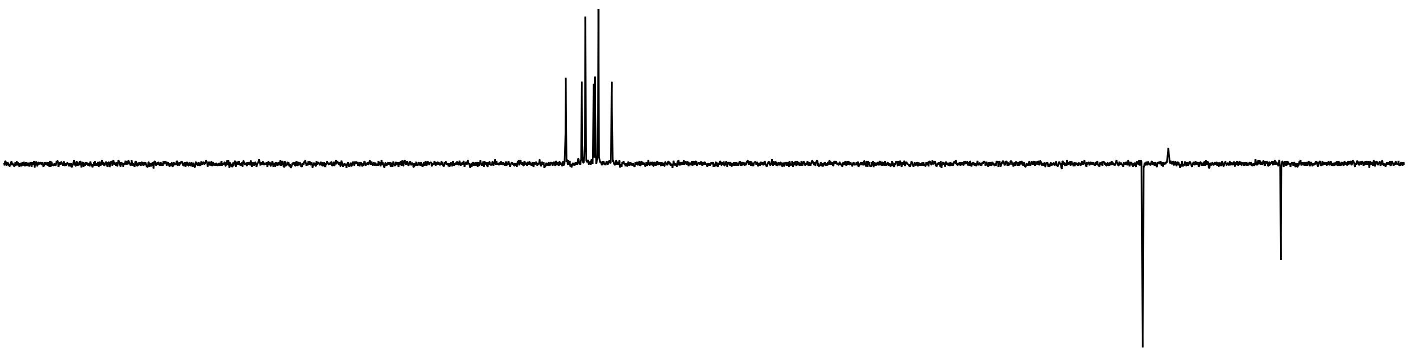

13C NMR (75 MHz, CDCl3)

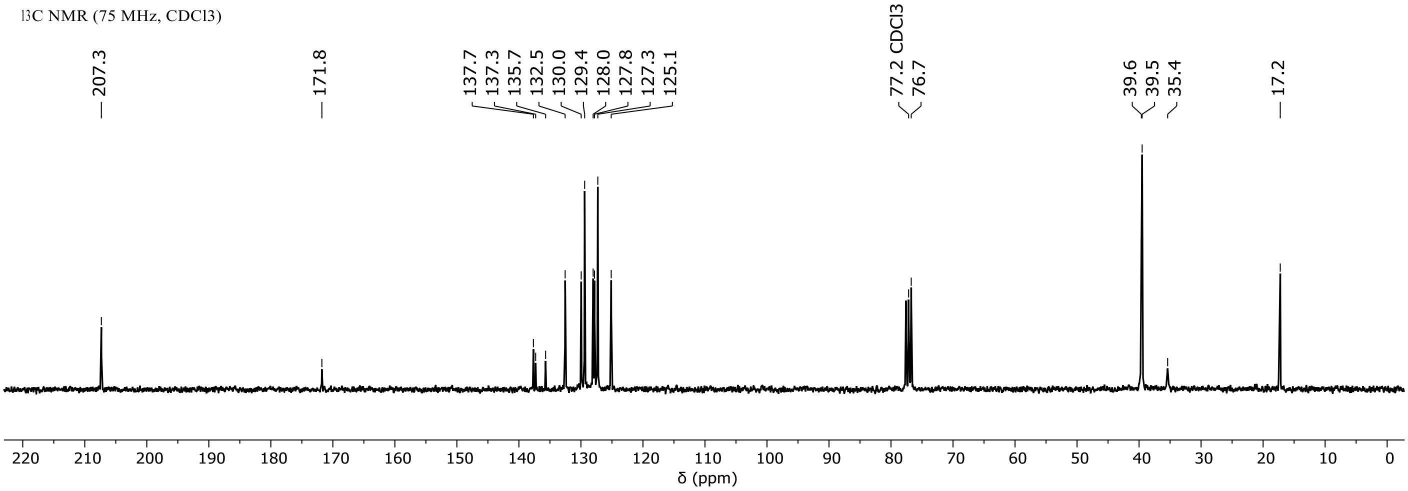

1H NMR (300 MHz, CDCl3)

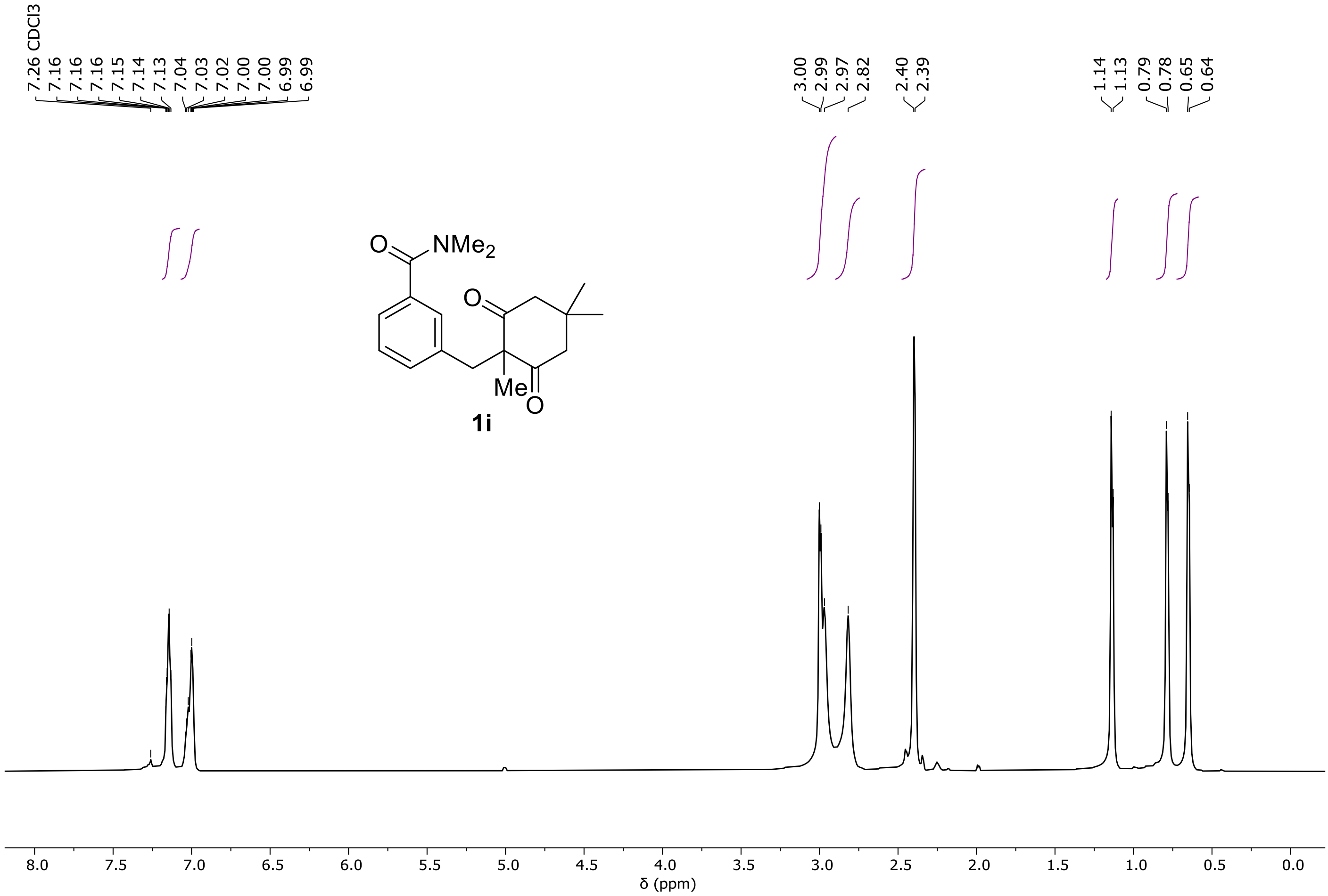

DEPT-135 NMR (75 MHz, CDCl3)

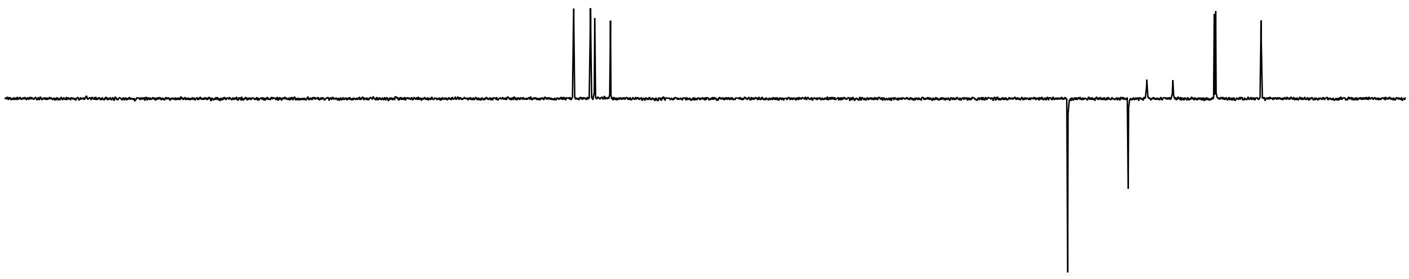

13C NMR (75 MHz, CDCl3)

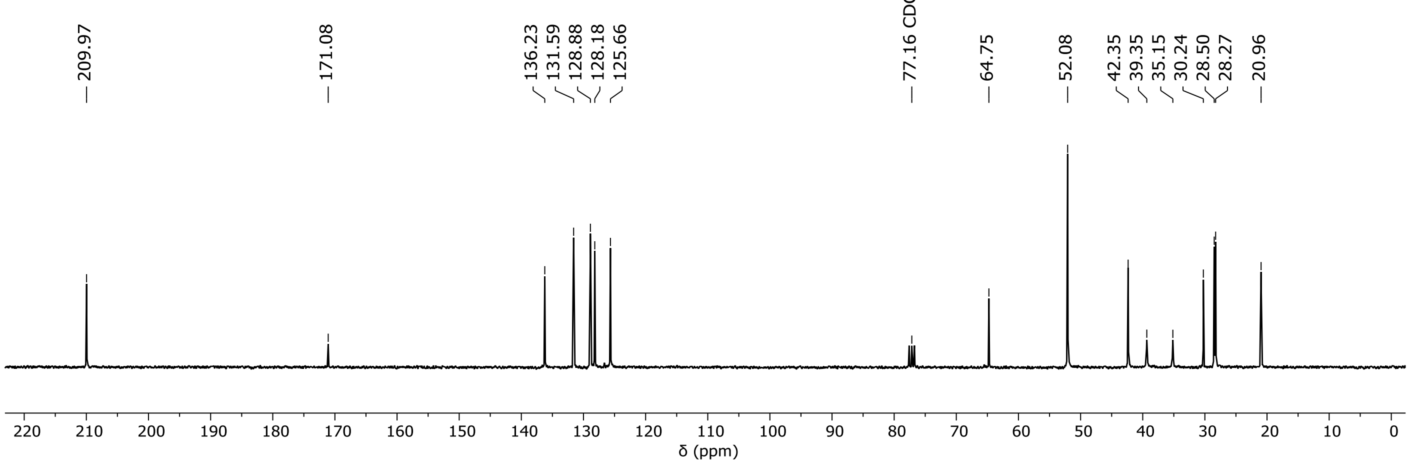

1H NMR (300 MHz, CDCl3)

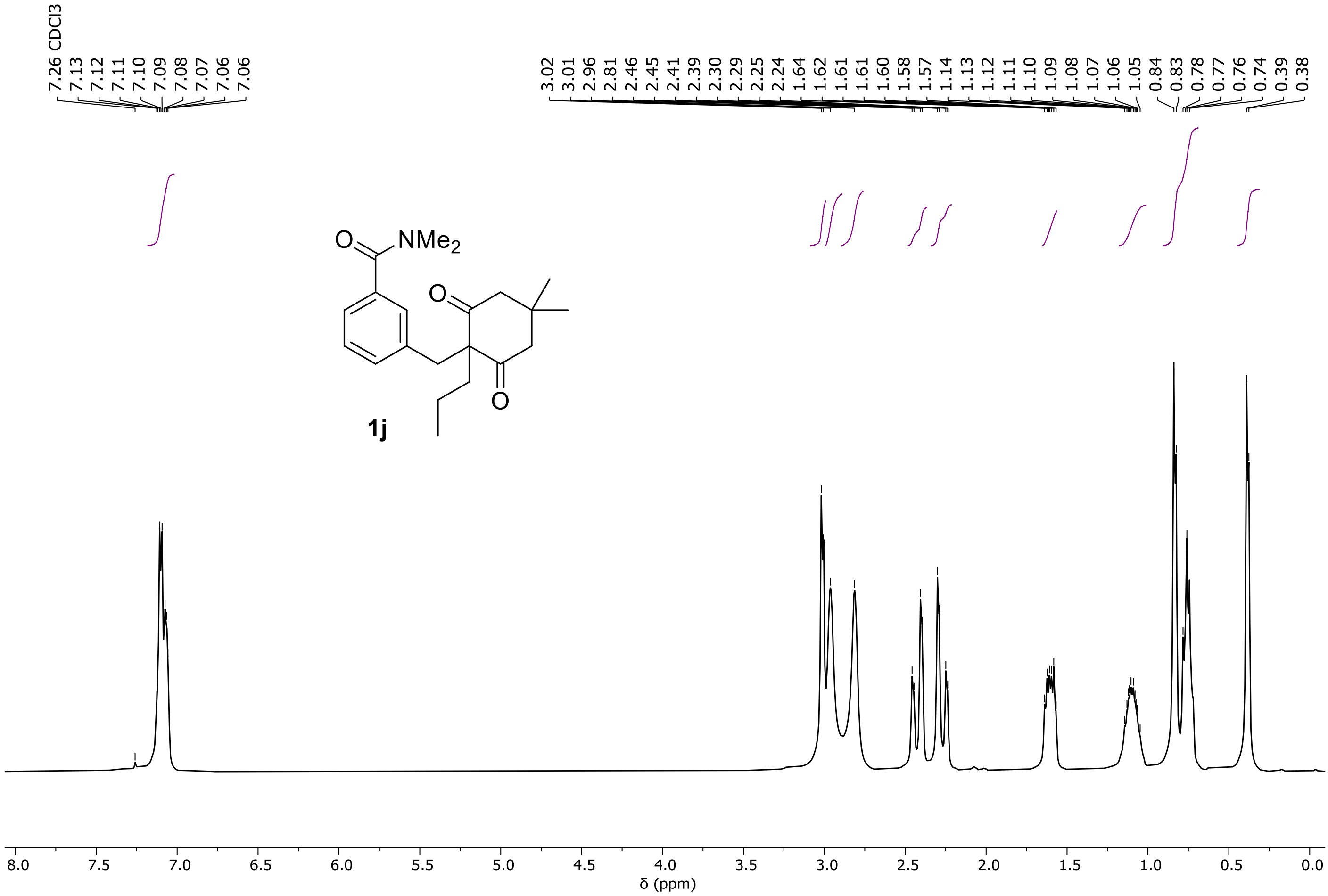

DEPT-135 NMR (75 MHz, CDCl3)

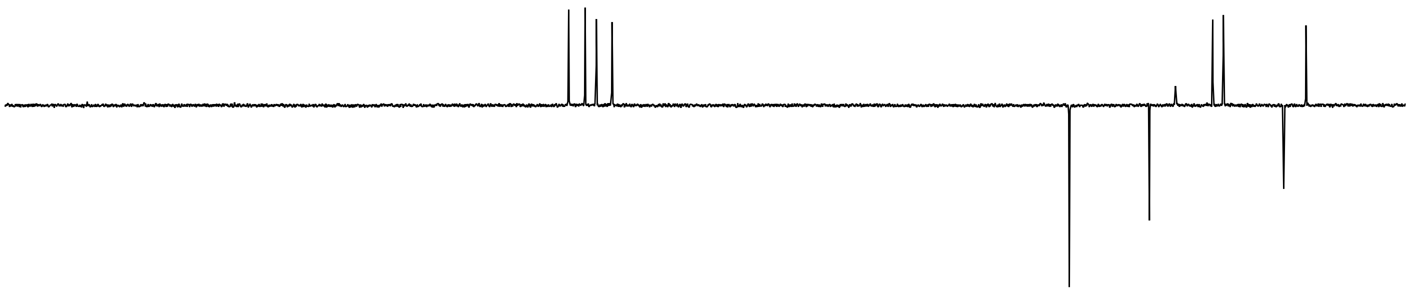

13C NMR (75 MHz, CDCl3)

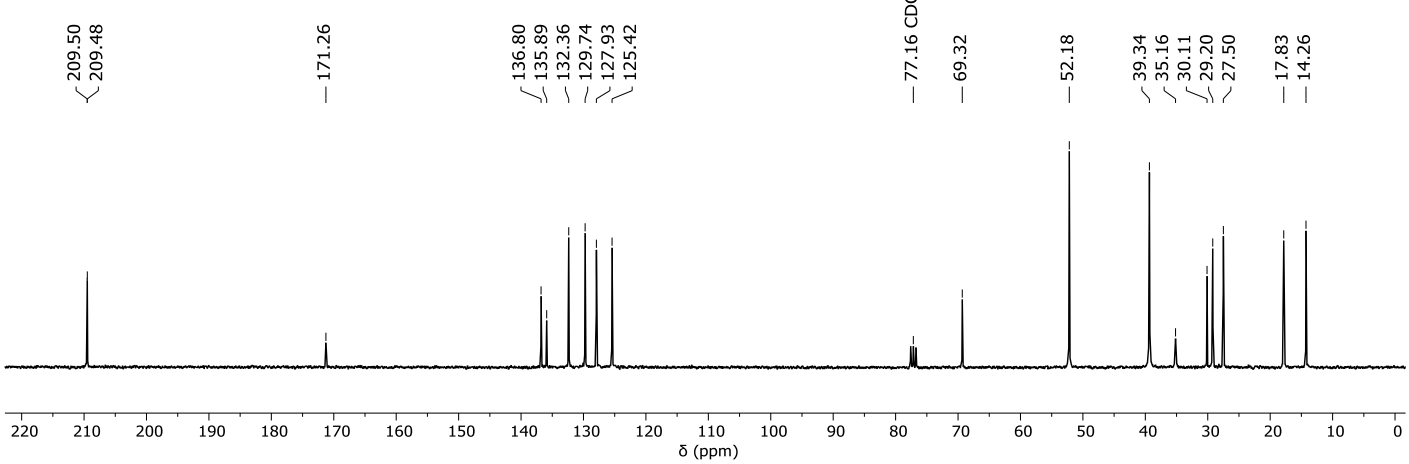

1H NMR (300 MHz, CDCl3)

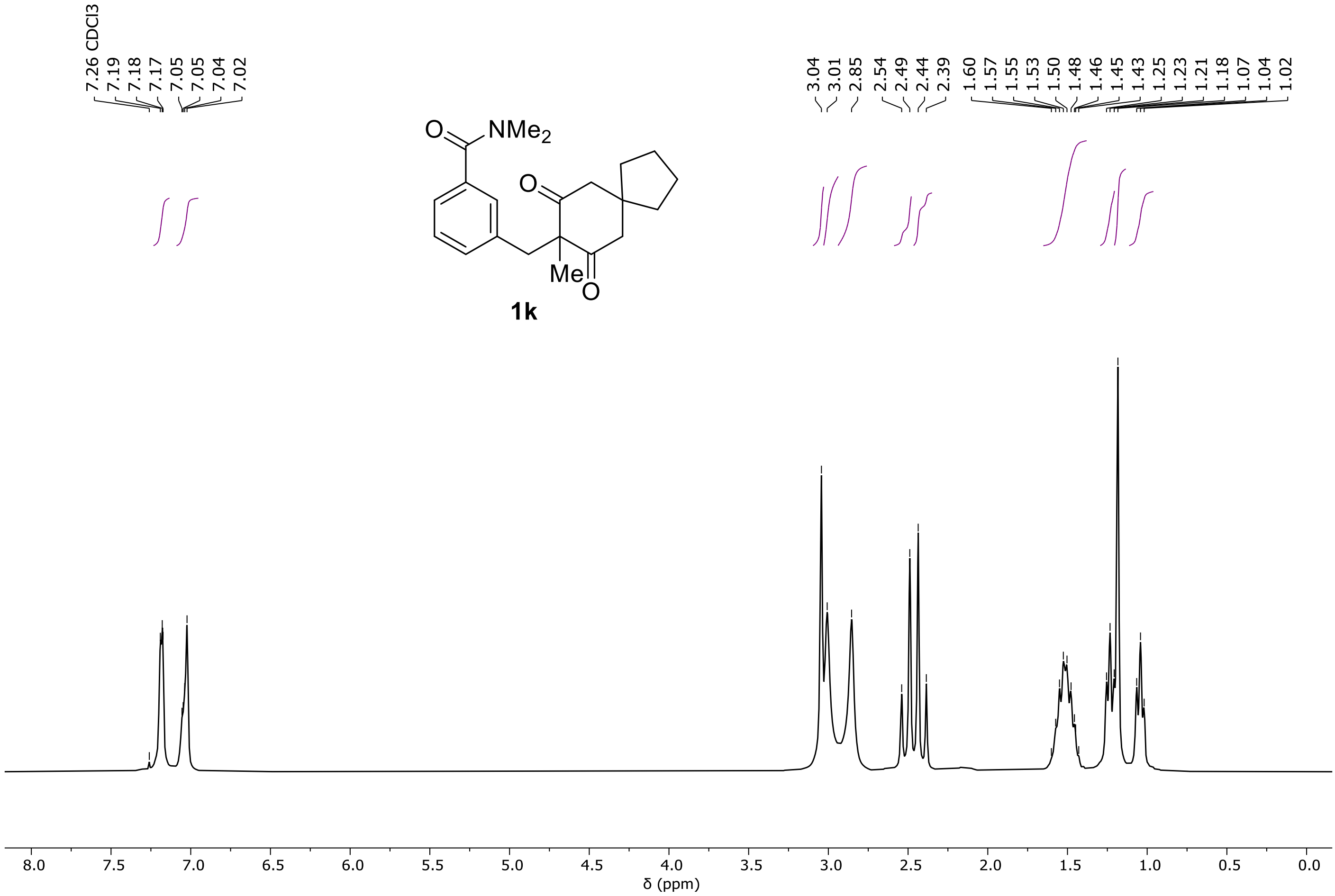

DEPT-135 NMR (75 MHz, CDCl3)

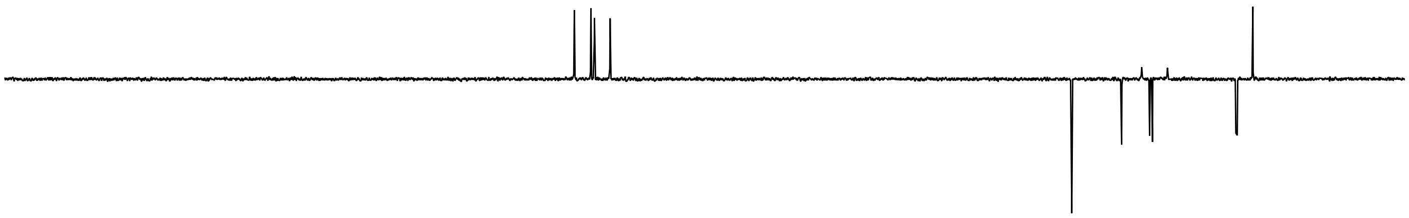

13C NMR (75 MHz, CDCl3)

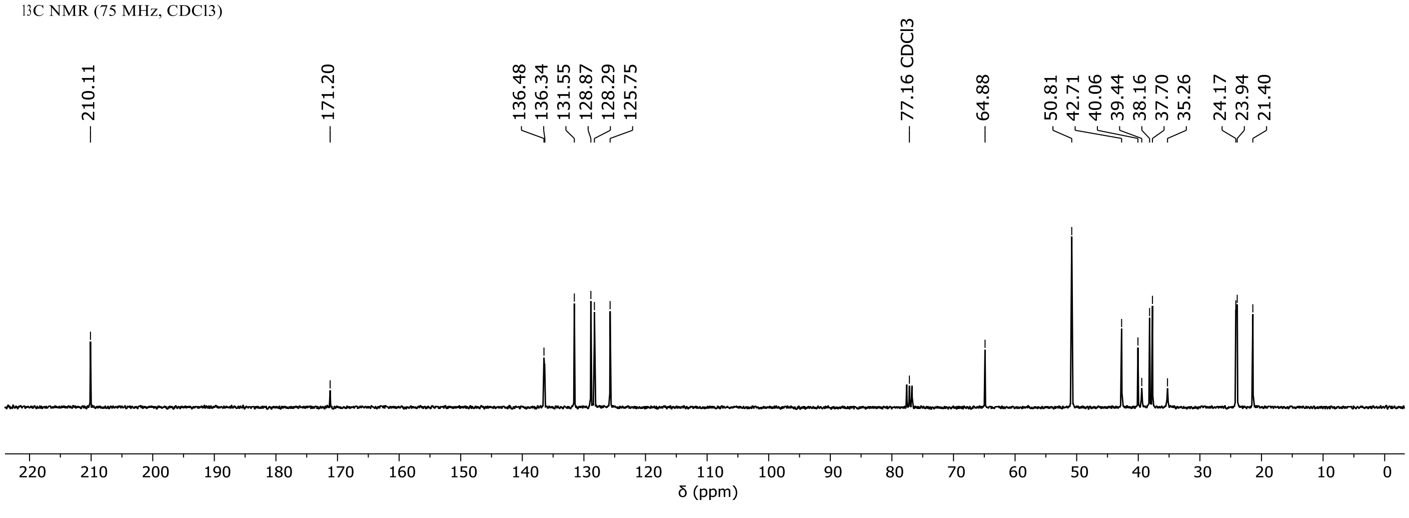

1H NMR (300 MHz, CDCl3)

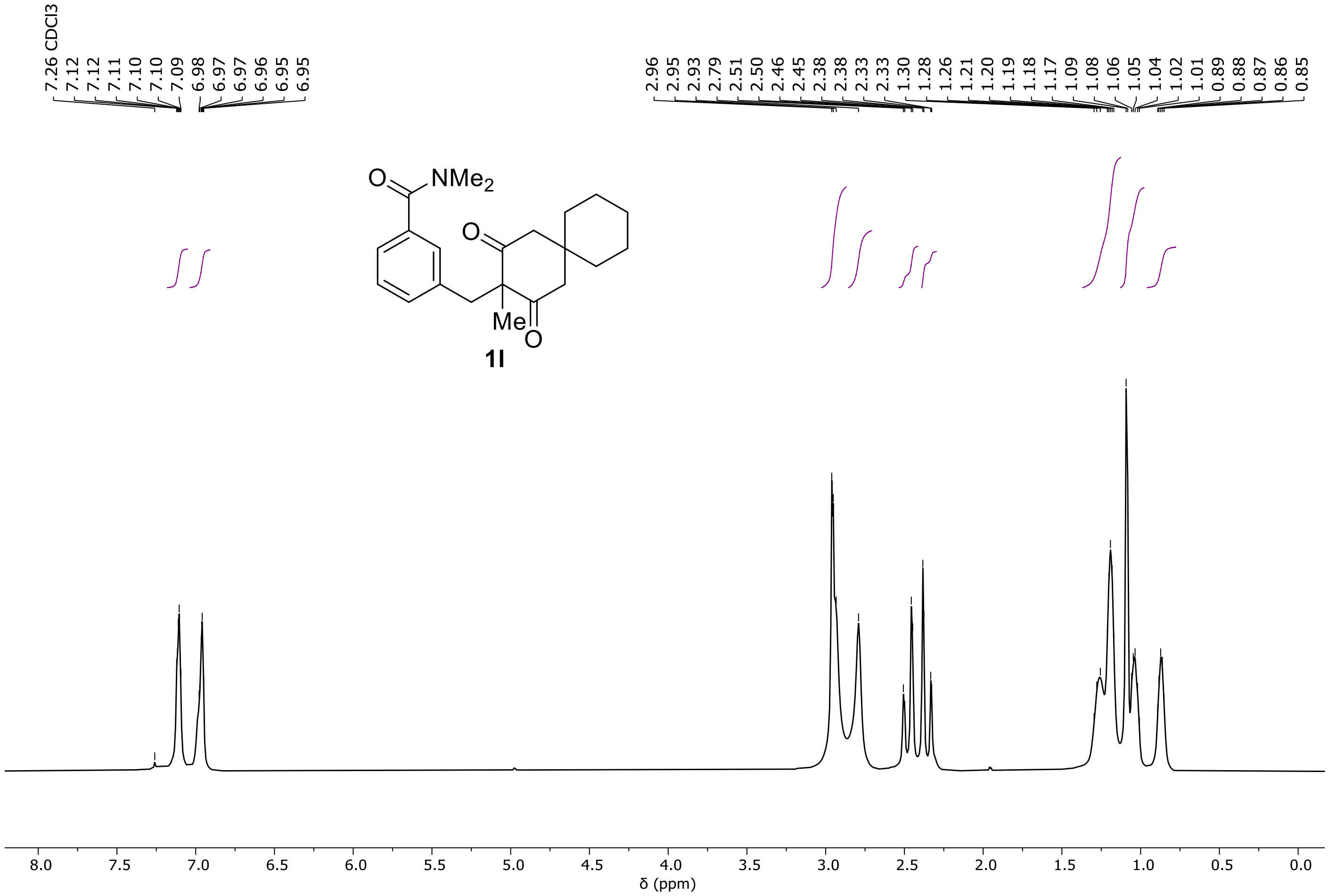

1l

DEPT-135 NMR (75 MHz, CDCl3)

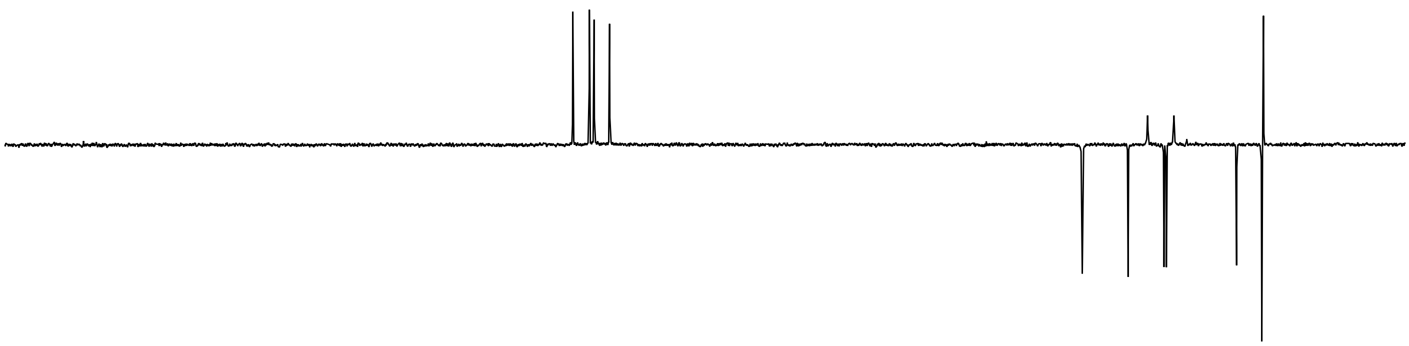

13C NMR (75 MHz, CDCl3)

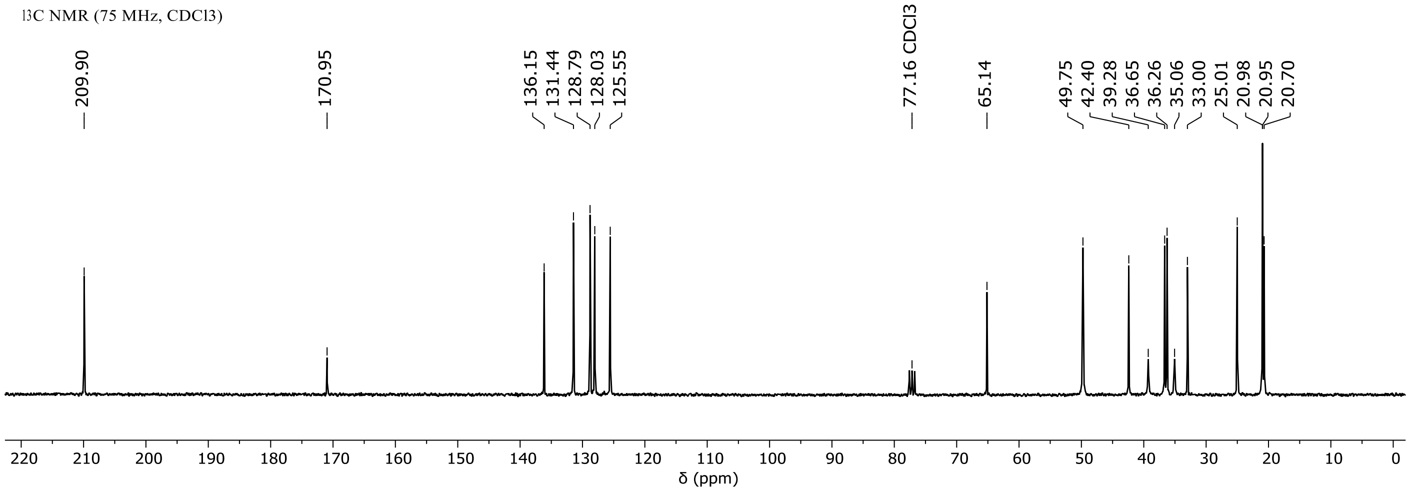

1H NMR (300 MHz, CDCl3)

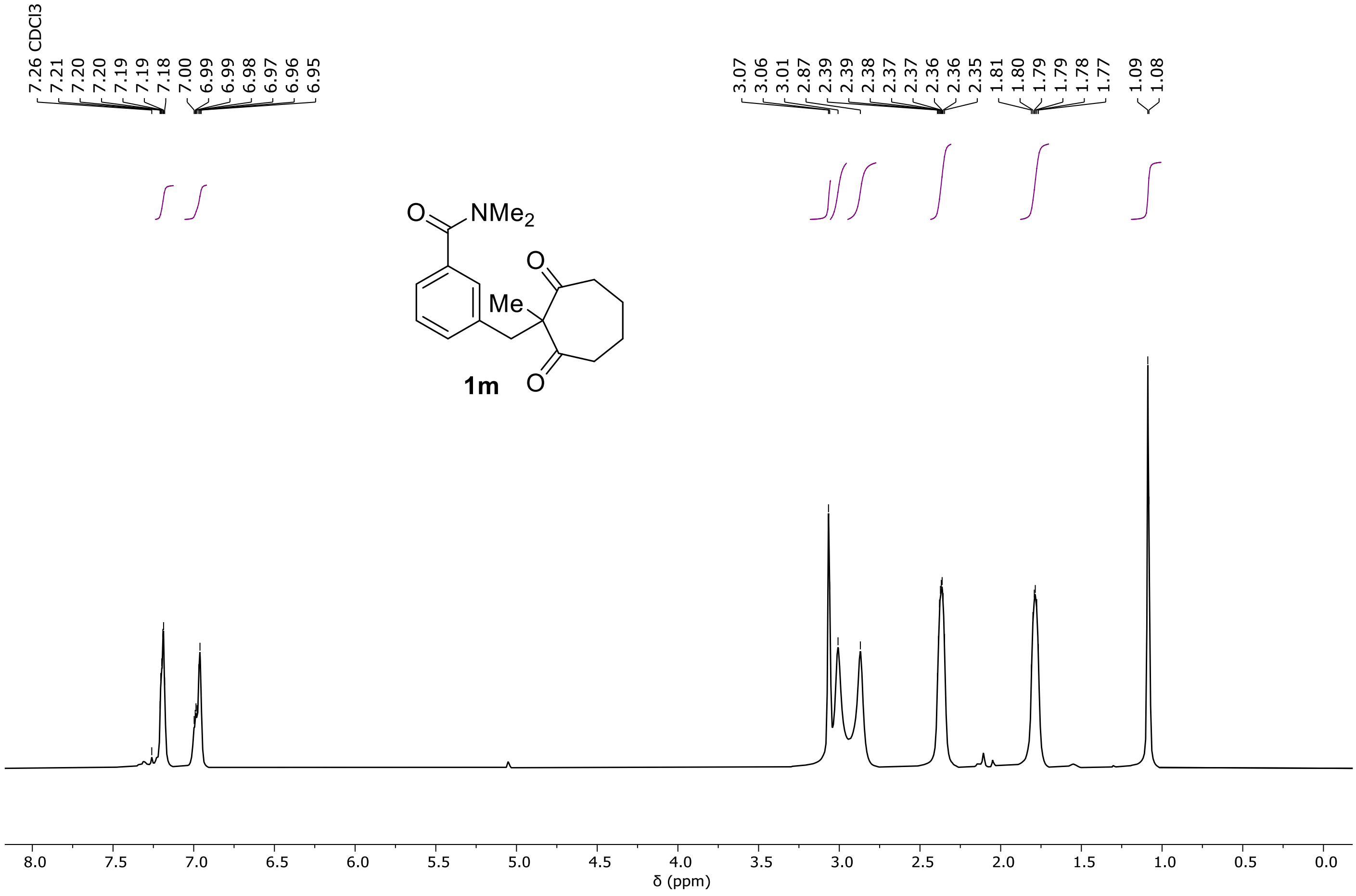

DEPT-135 NMR (75 MHz, CDCl3)

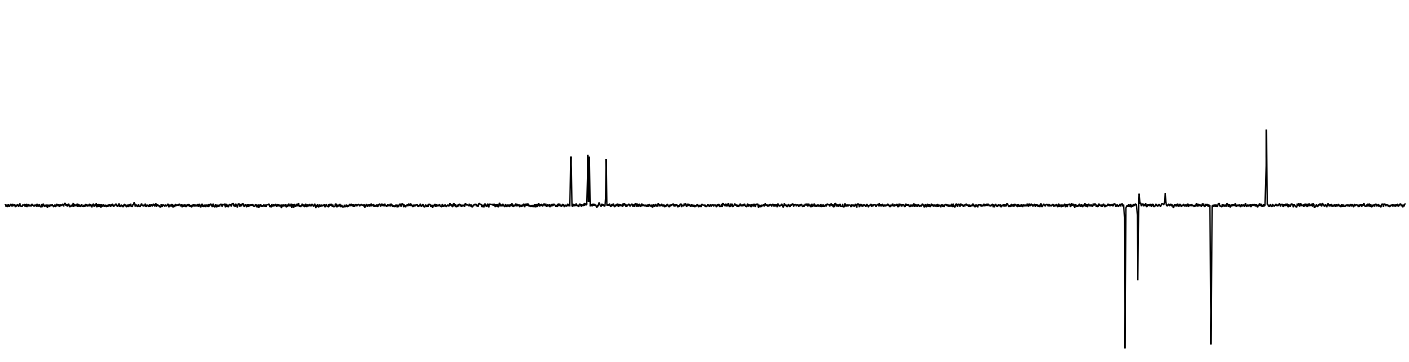

13C NMR (75 MHz, CDCl3)

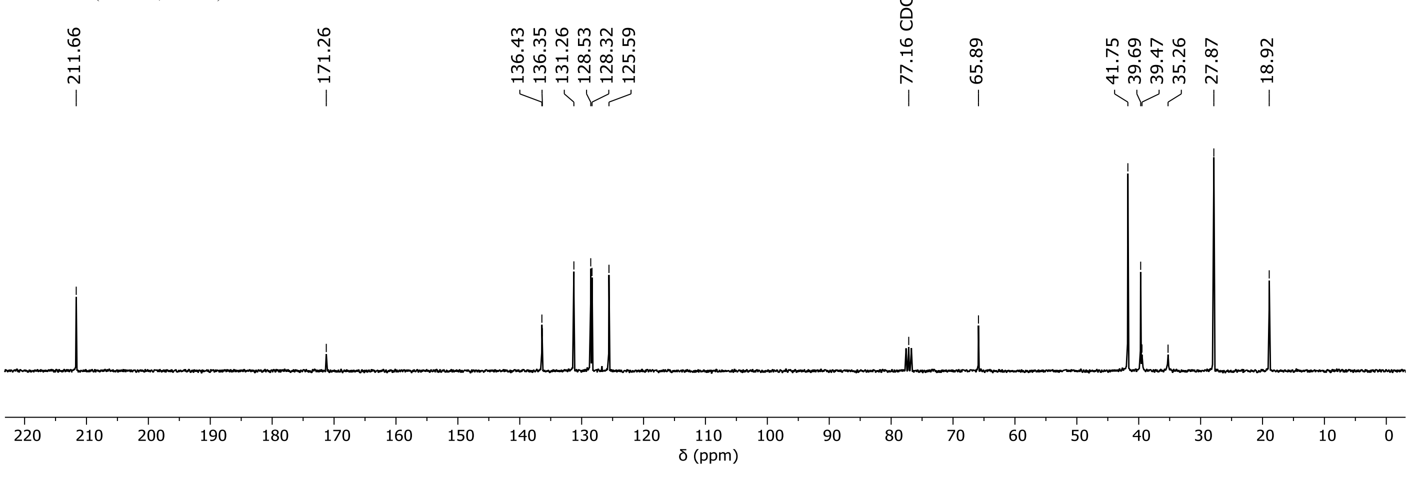

1H NMR (300 MHz, CDCl3)

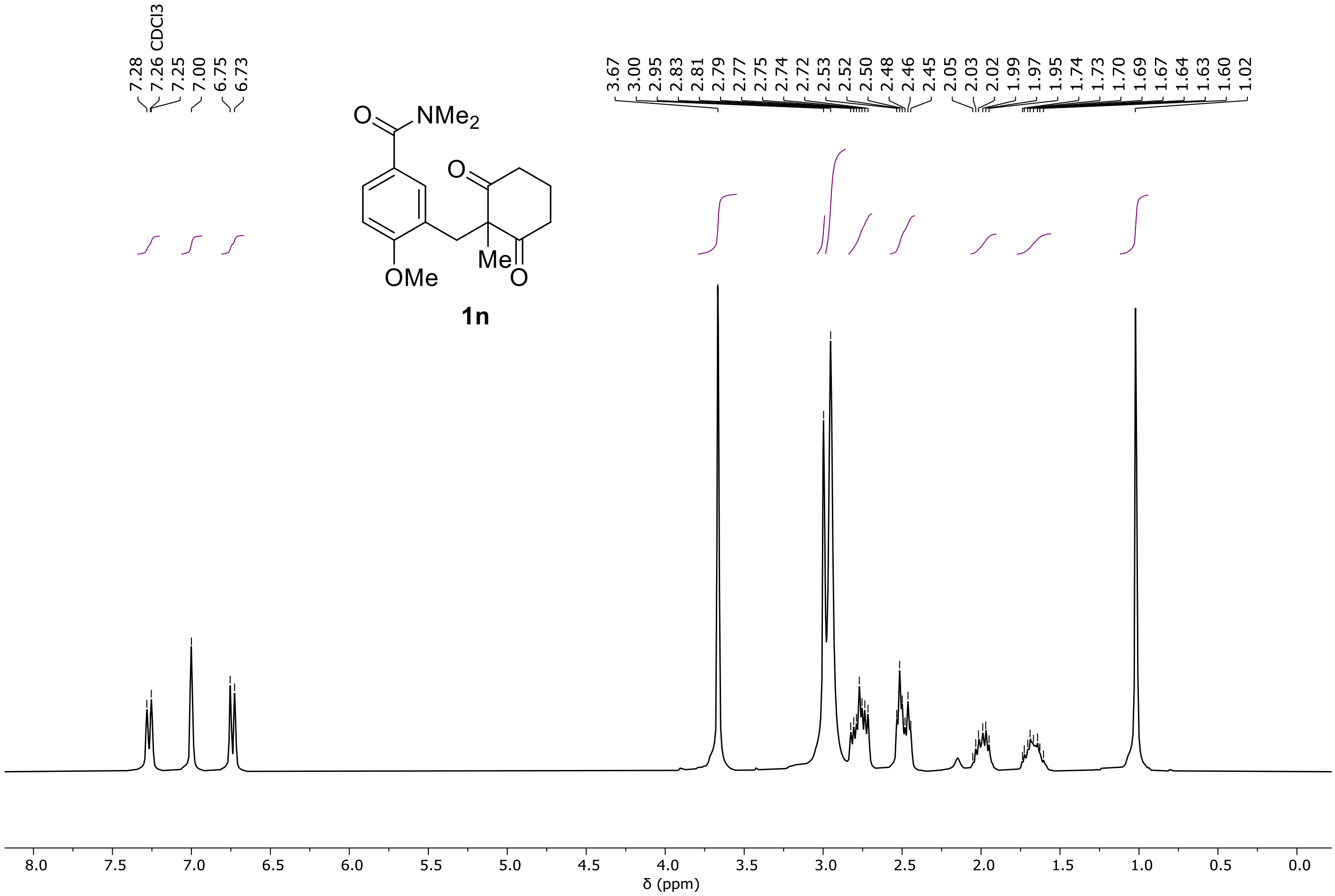

DEPT-135 NMR (75 MHz, CDCl3)

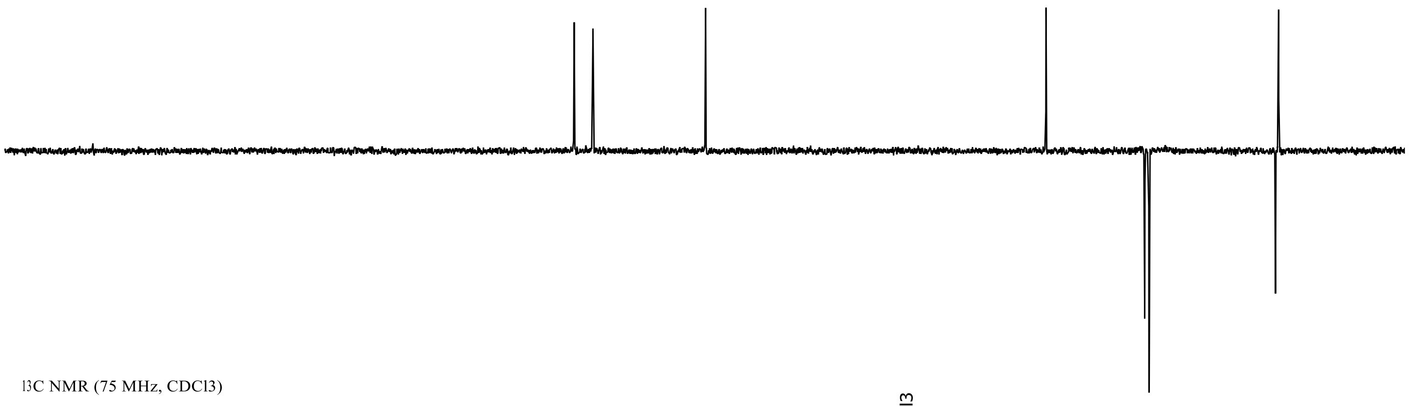

13C NMR (75 MHz, CDCl3)

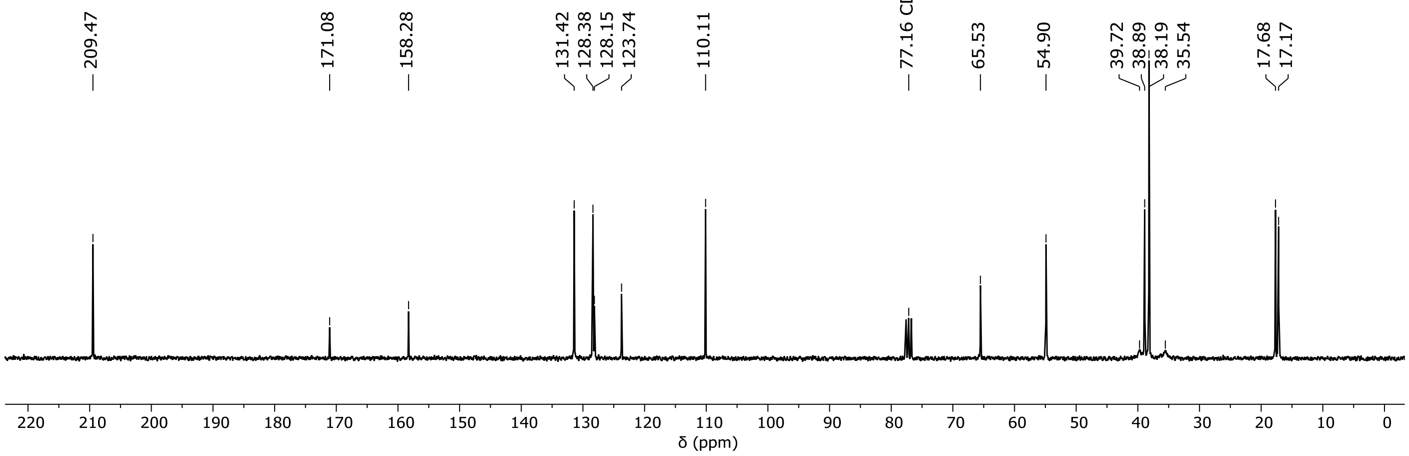

1H NMR (300 MHz, CDCl3)

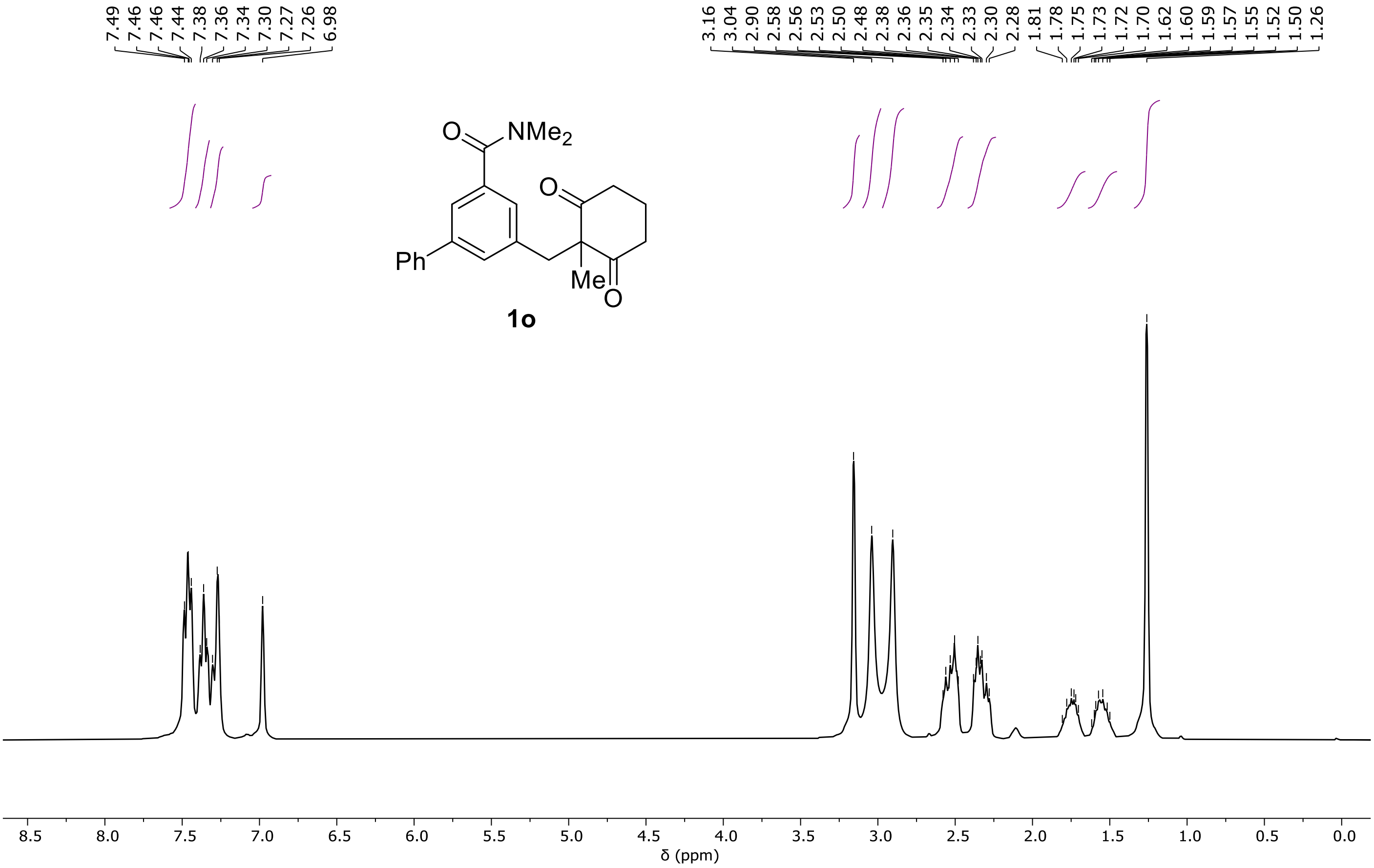

DEPT-135 NMR (75 MHz, CDCl3)

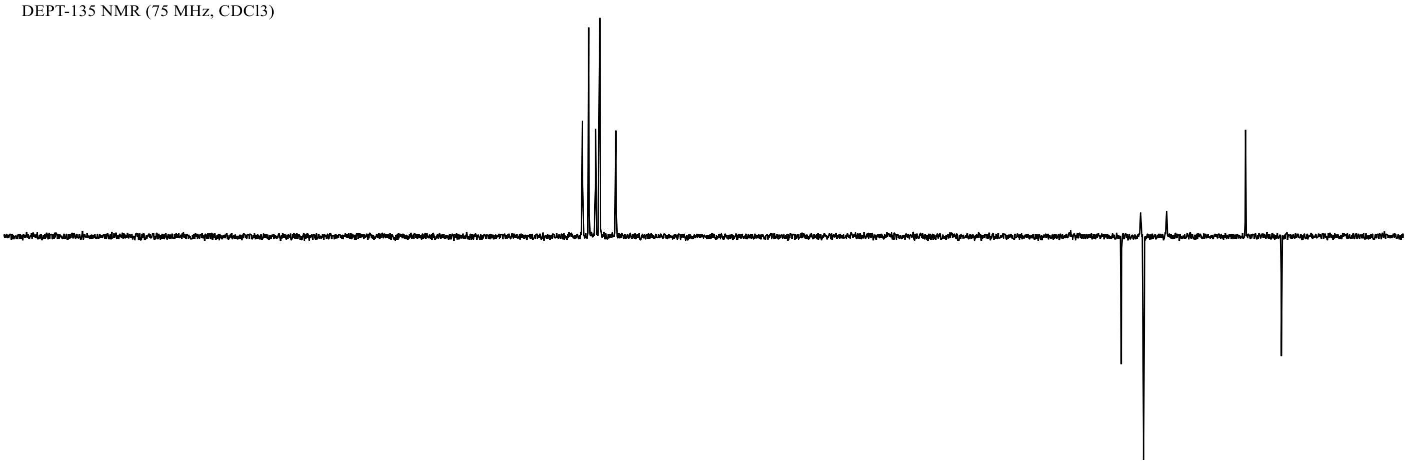

13C NMR (75 MHz, CDCl3)

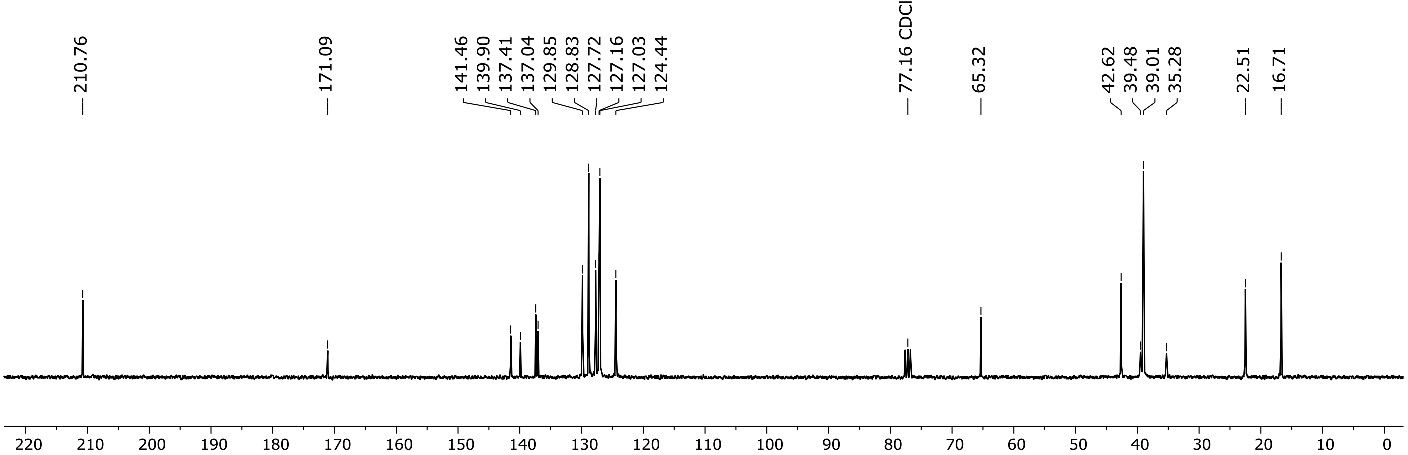

1H NMR (300 MHz, CDCl3)

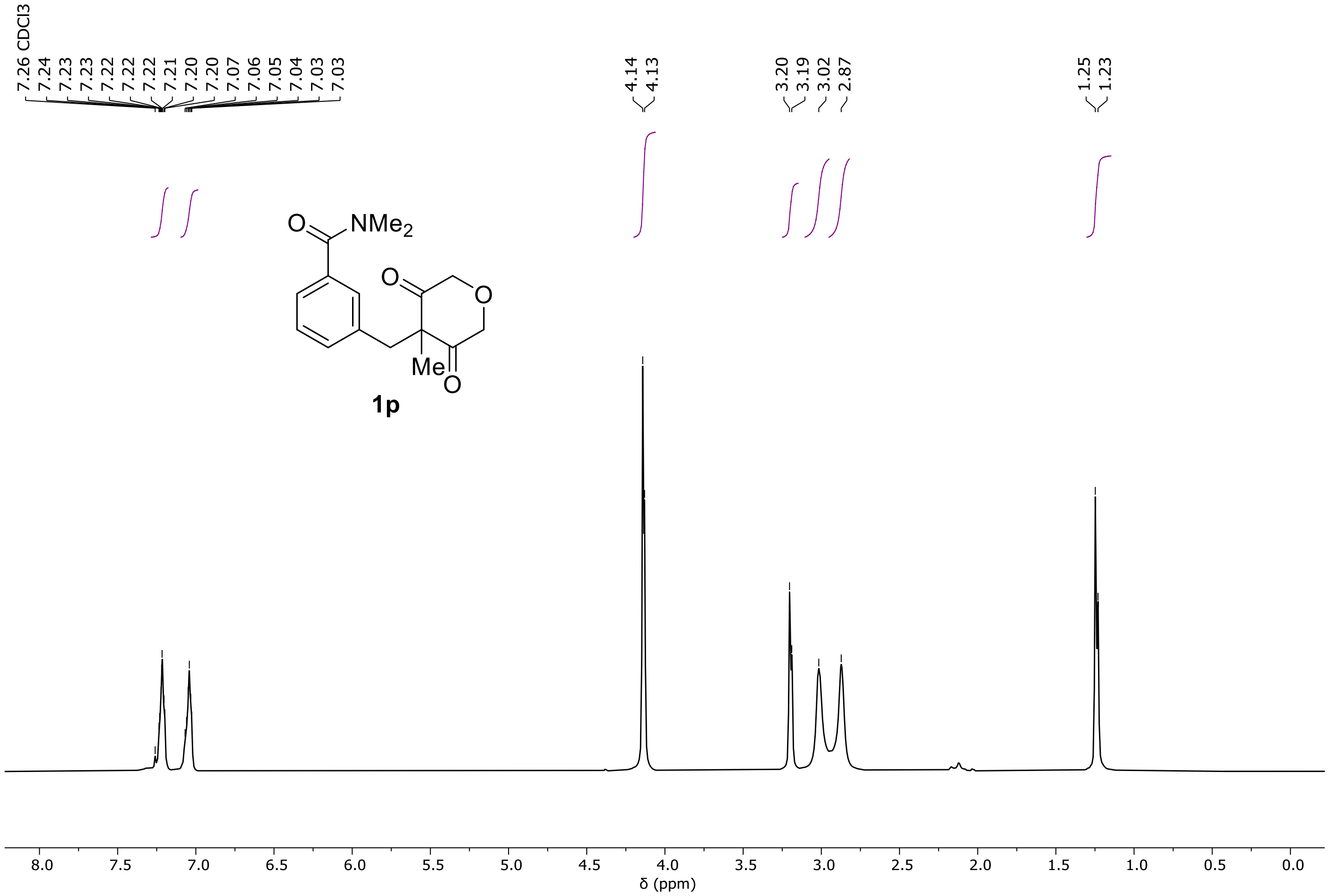

DEPT-135 NMR (75 MHz, CDCl3)

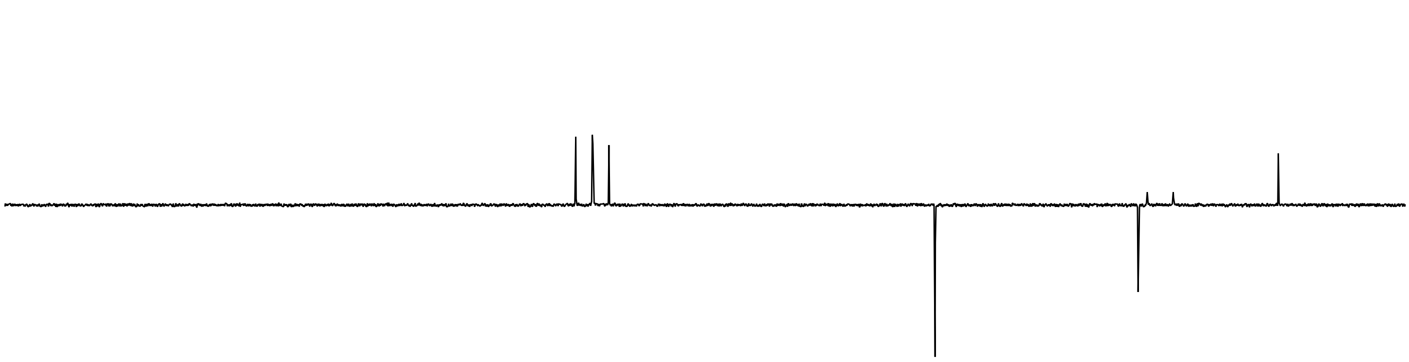

13C NMR (75 MHz, CDCl3)

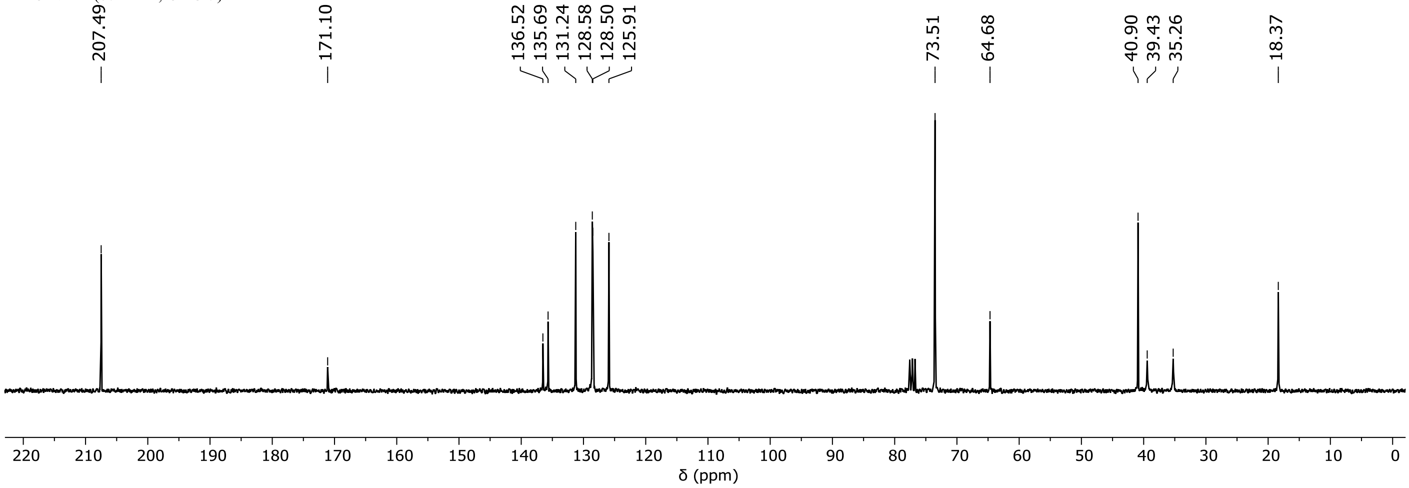

1H NMR (300 MHz, CDCl3)

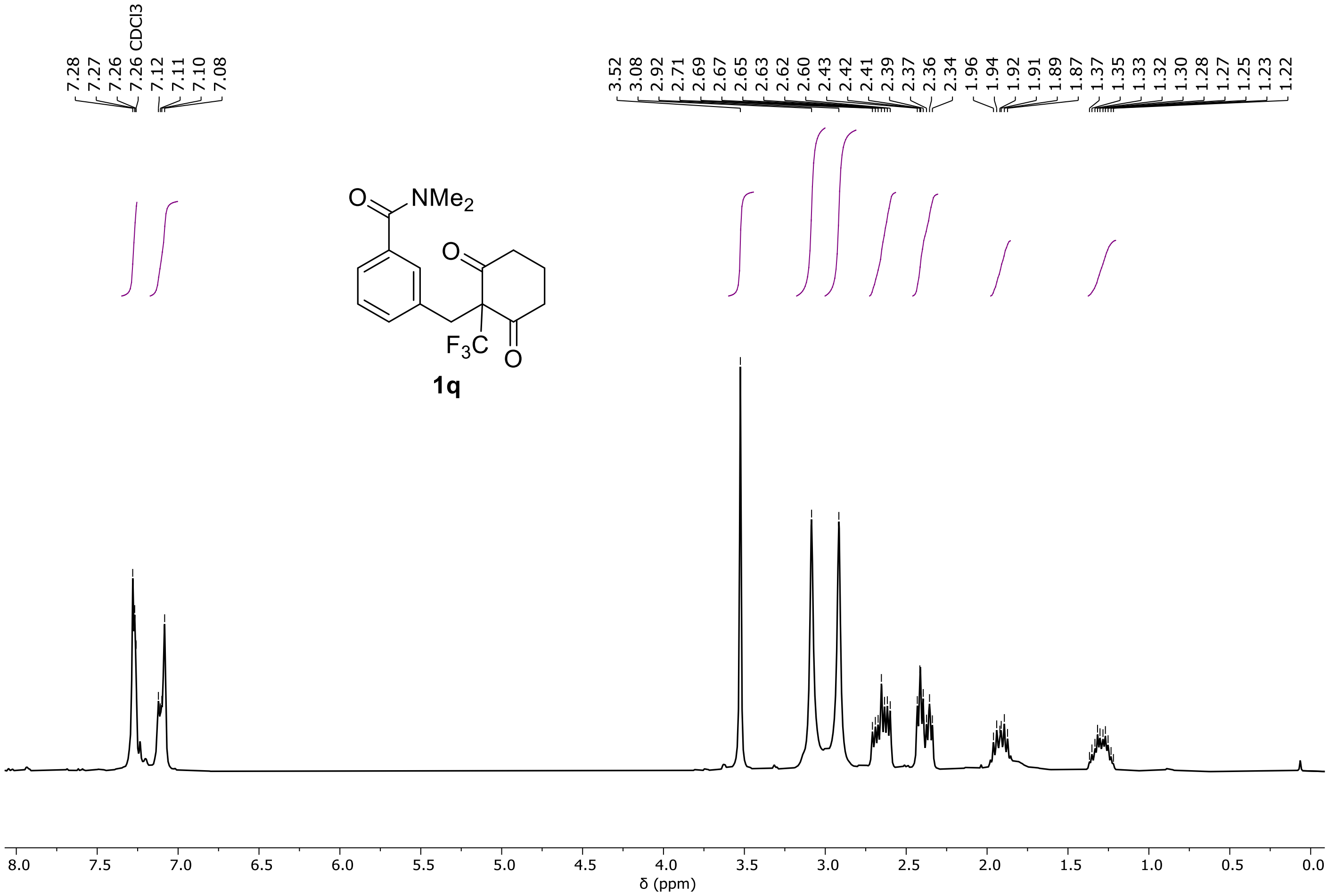

DEPT-135 NMR (75 MHz, CDCl3)

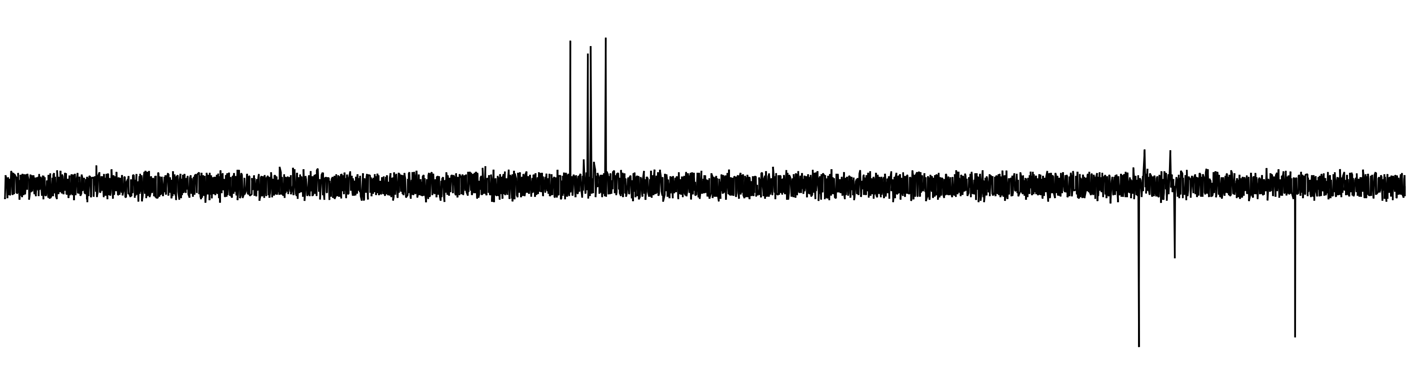

13C NMR (75 MHz, CDCl3)

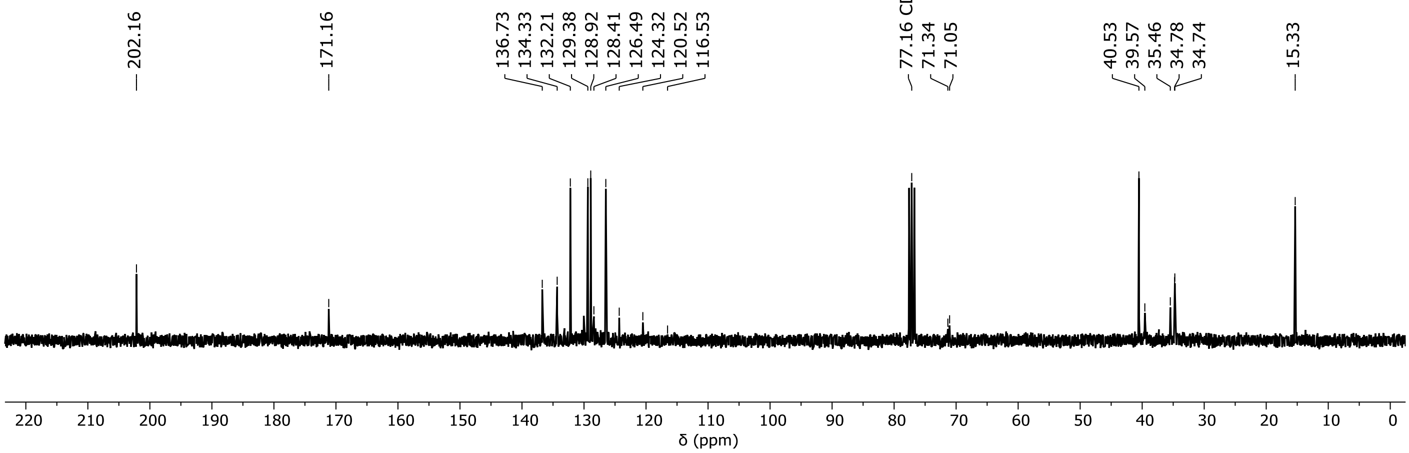

1H NMR (300 MHz, CDCl3)

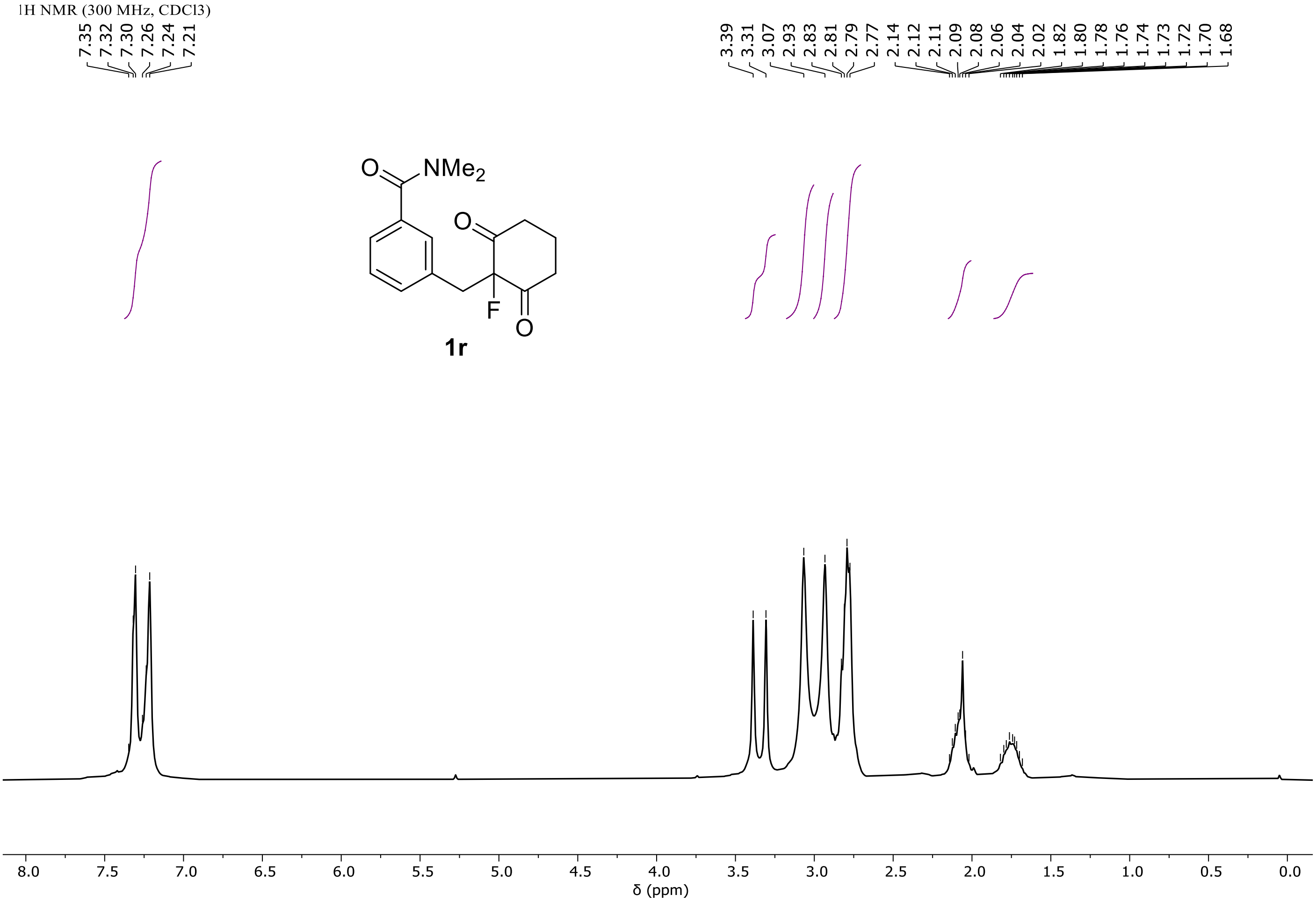

DEPT-135 NMR (75 MHz, CDCl3)

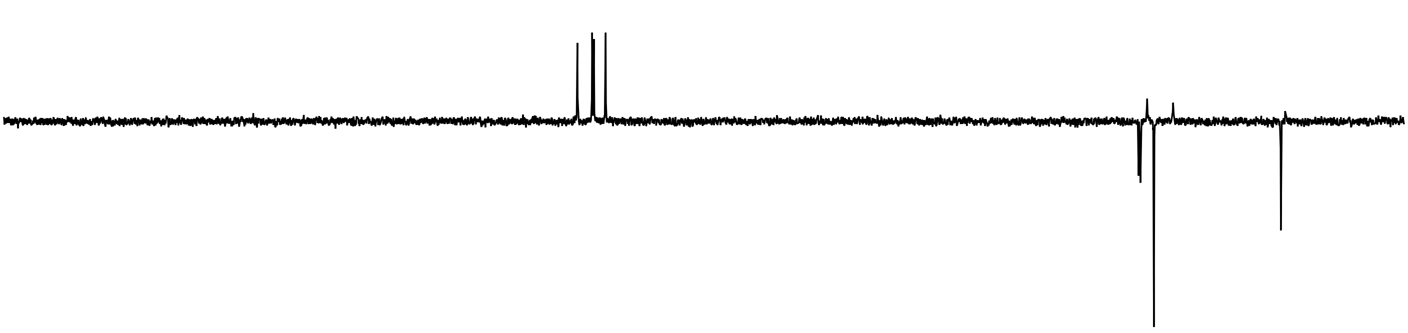

13C NMR (75 MHz, CDCl3)

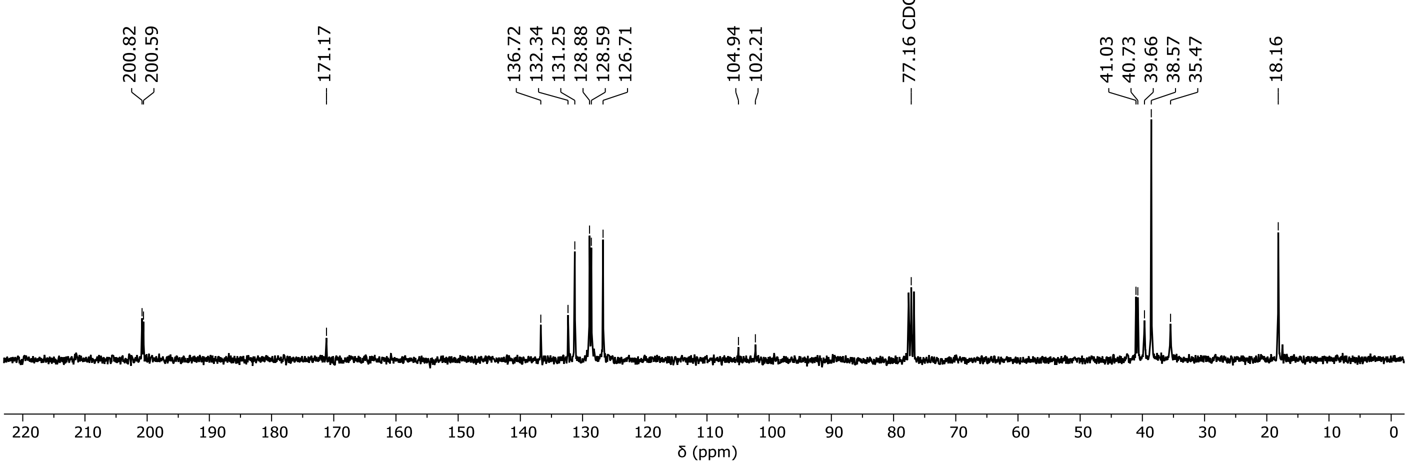

1H NMR (500 MHz, CDCl3)

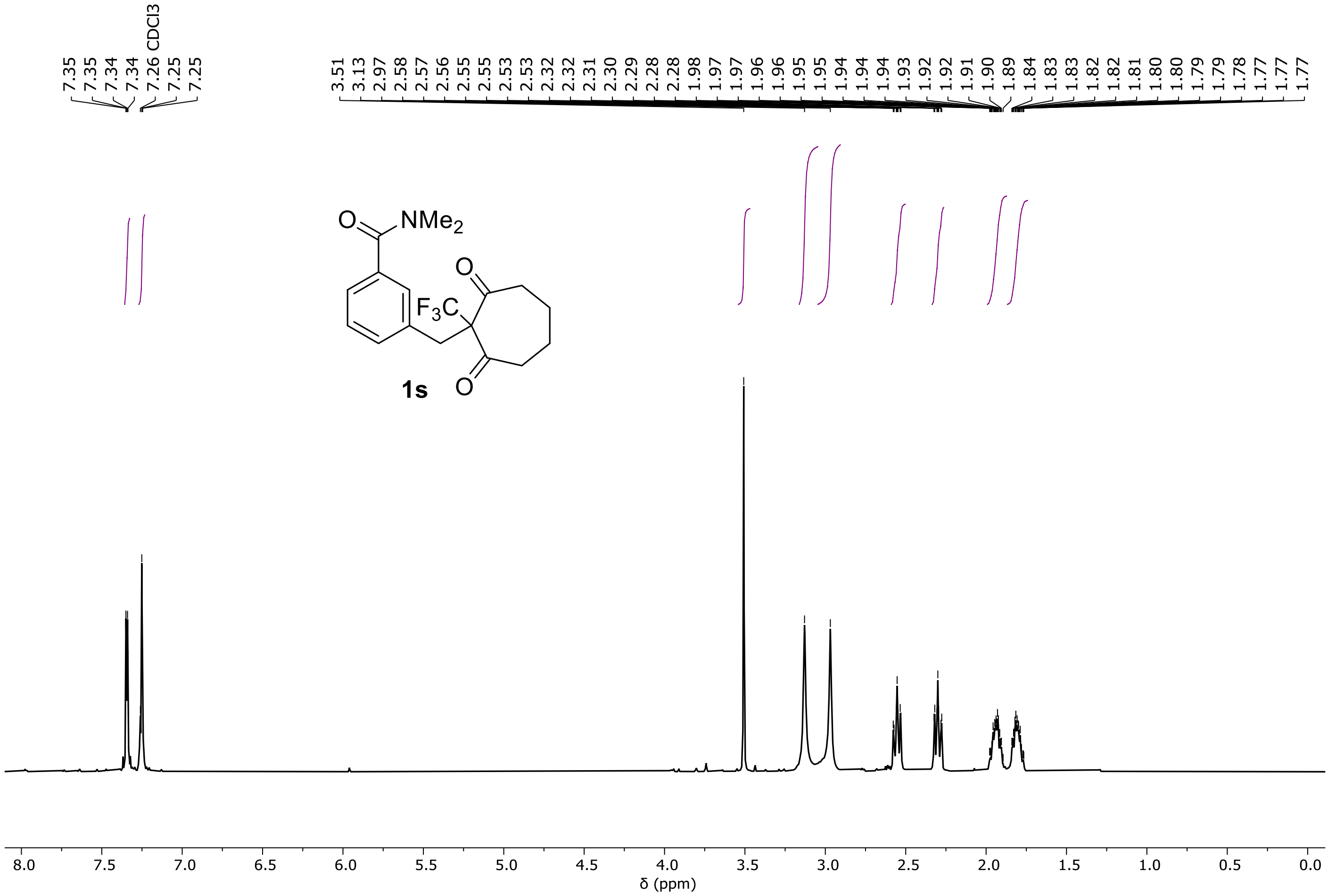

DEPT-135 NMR (126 MHz, CDCl3)

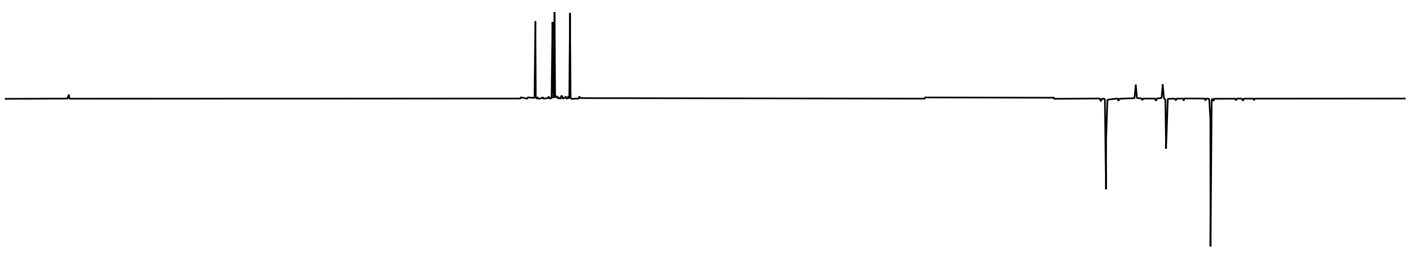

13C NMR (126 MHz, CDCl3)

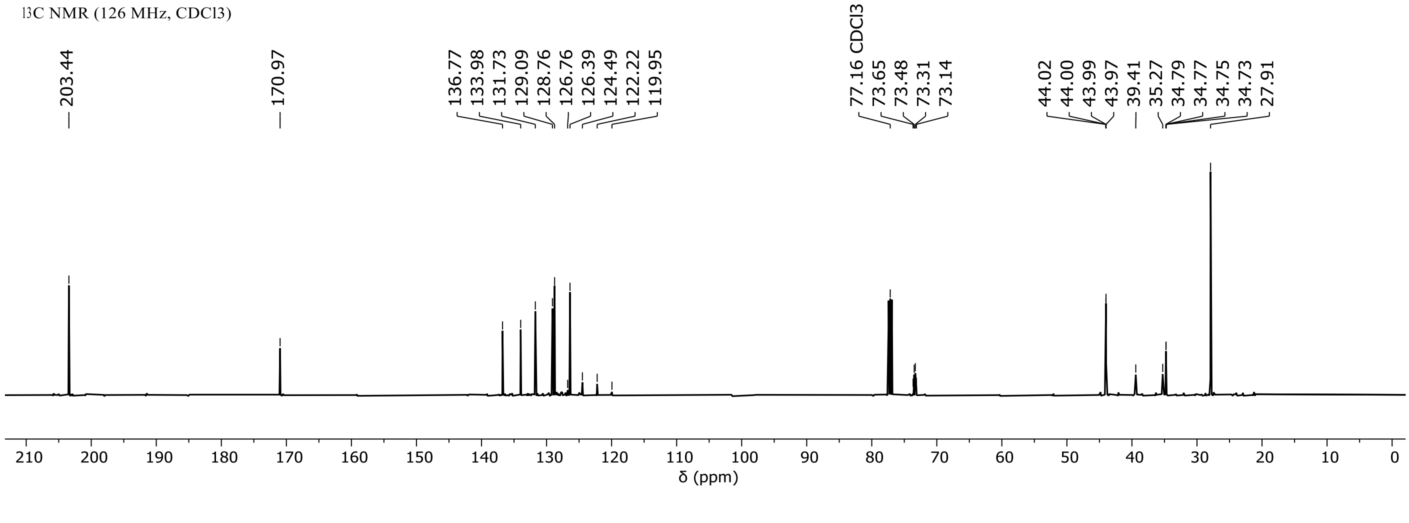

1H NMR (300 MHz, CDCl3)

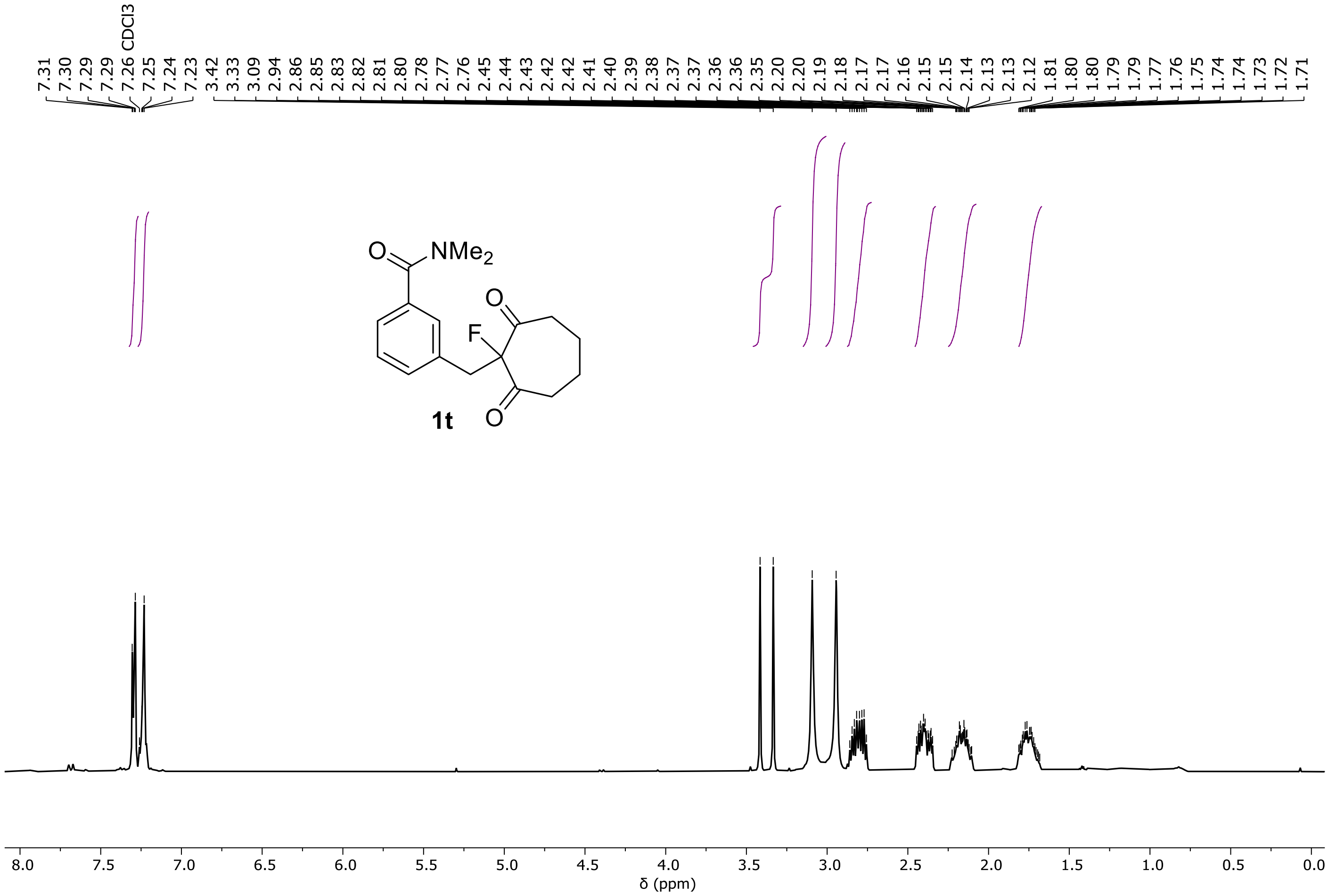

DEPT-135 NMR (75 MHz, CDCl3)

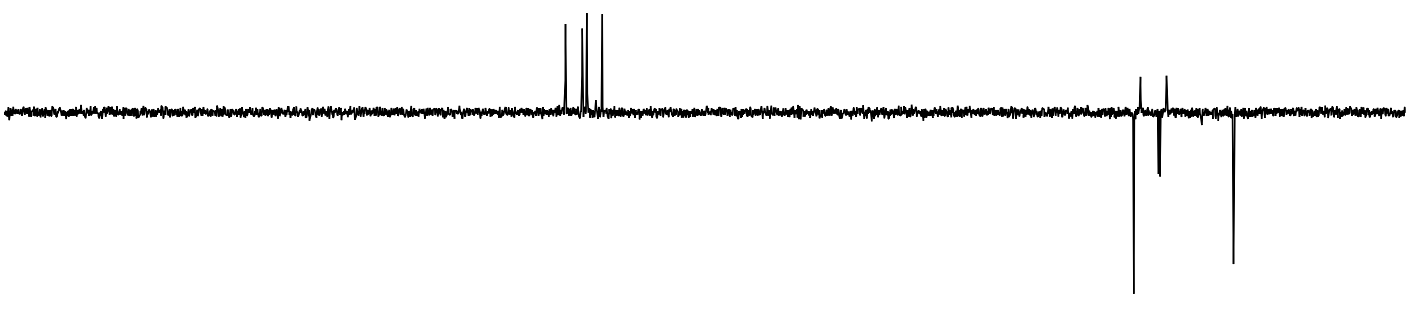

13C NMR (75 MHz, CDCl3)

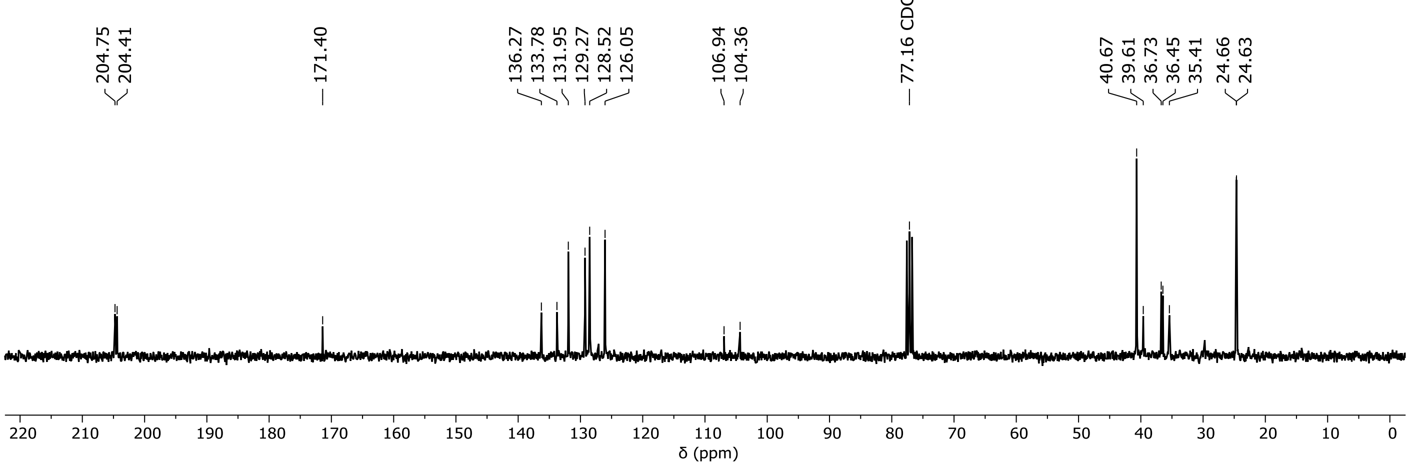

1H NMR (300 MHz, CDCl3)

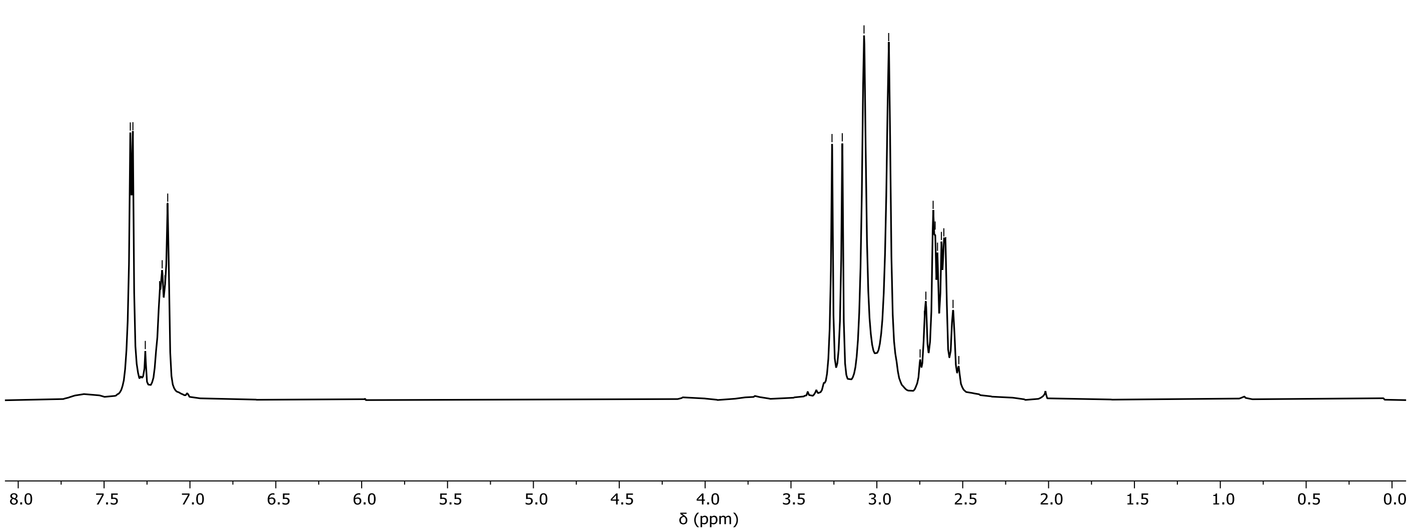

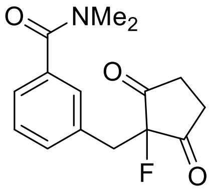

1u

DEPT-135 NMR (75 MHz, CDCl3)

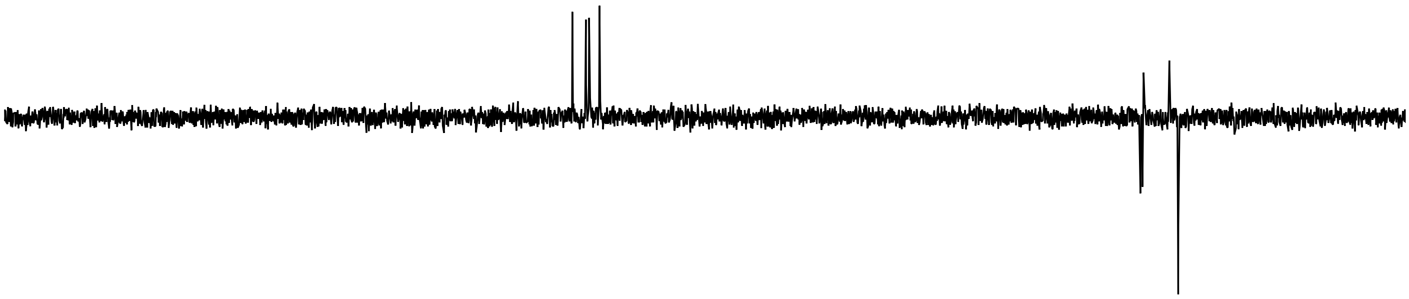

13C NMR (75 MHz, CDCl3)

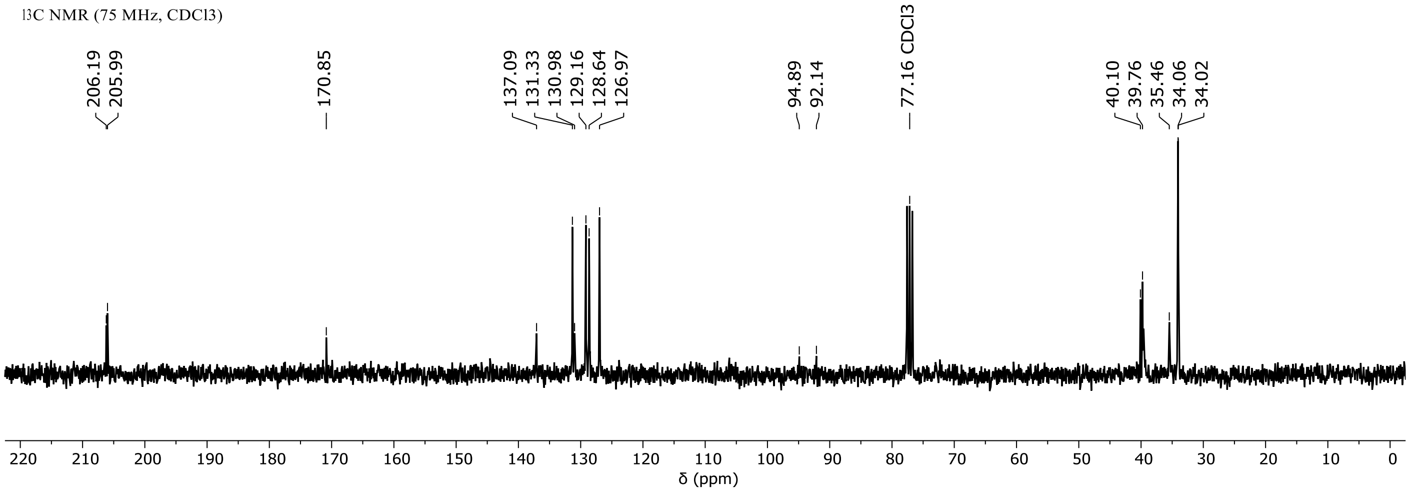

1H NMR (300 MHz, CDCl3)

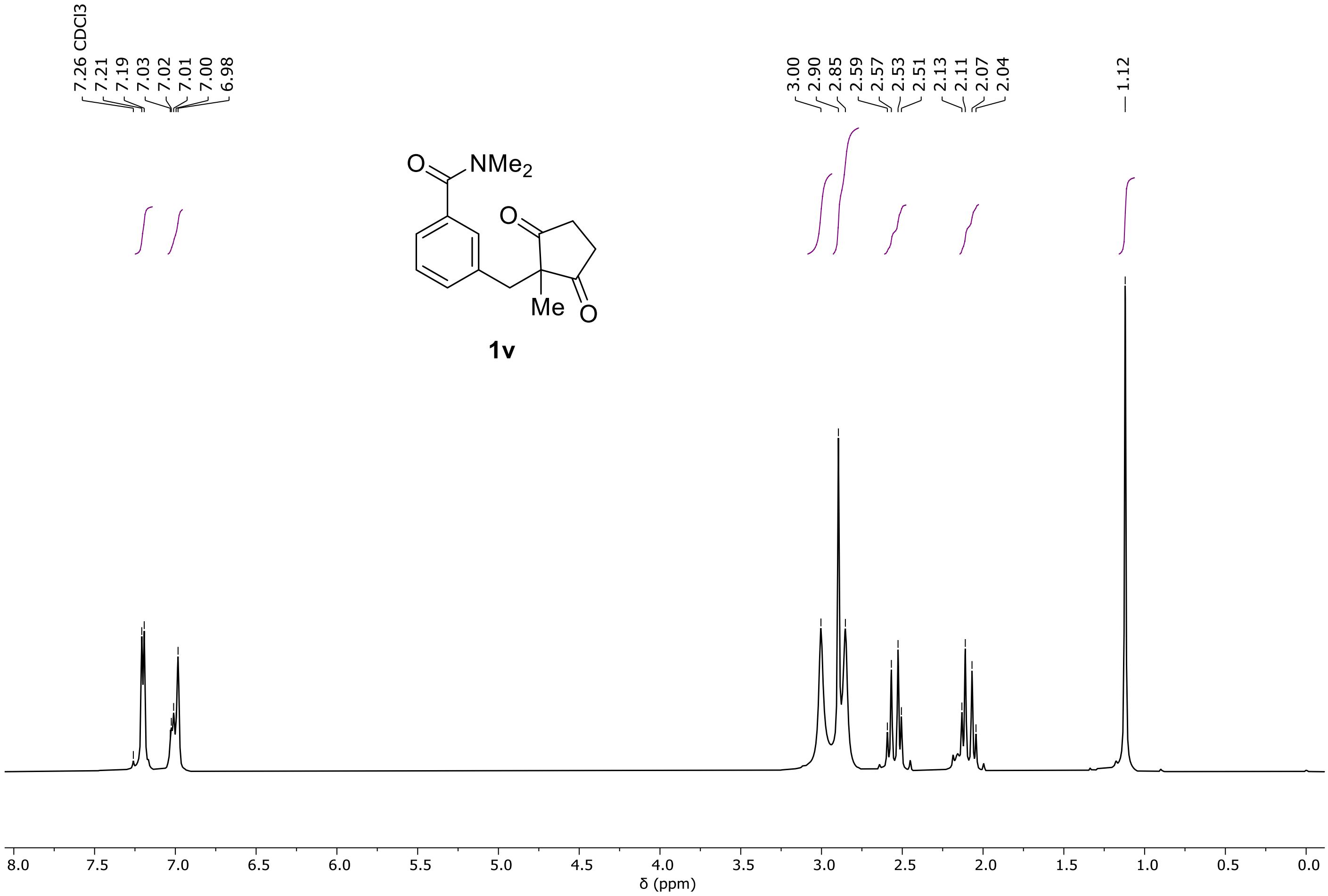

DEPT-135 NMR (75 MHz, CDCl3)

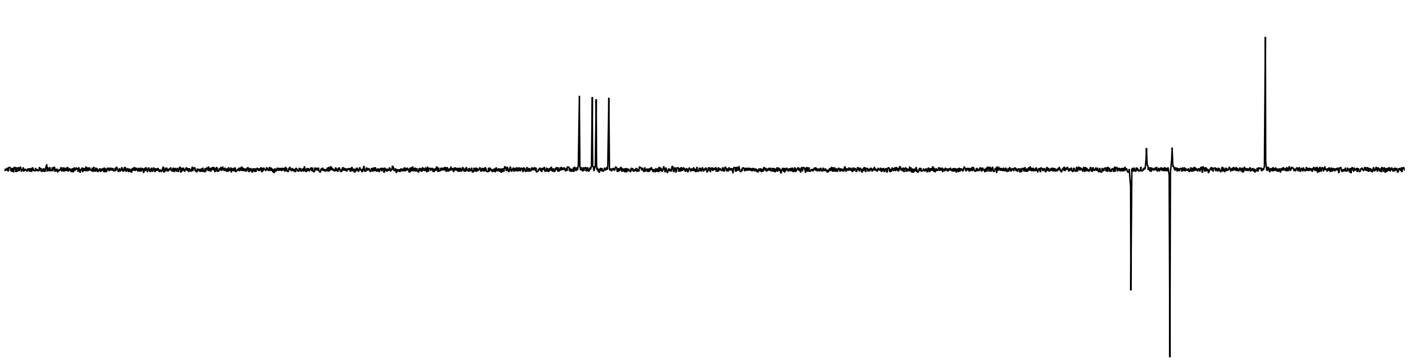

13C NMR (75 MHz, CDCl3)

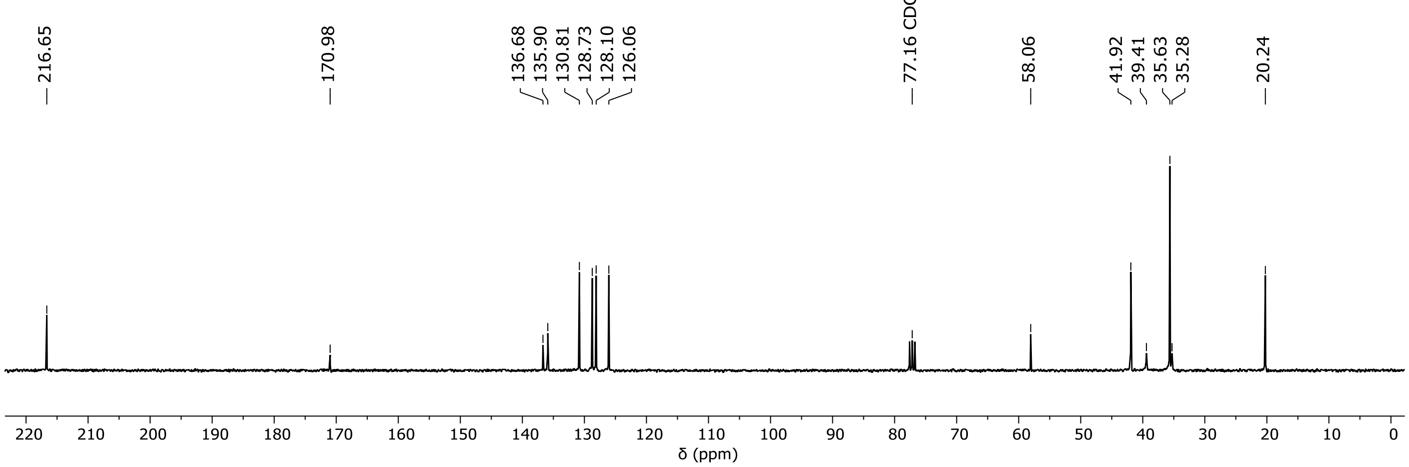

1H NMR (300 MHz, CDCl3)

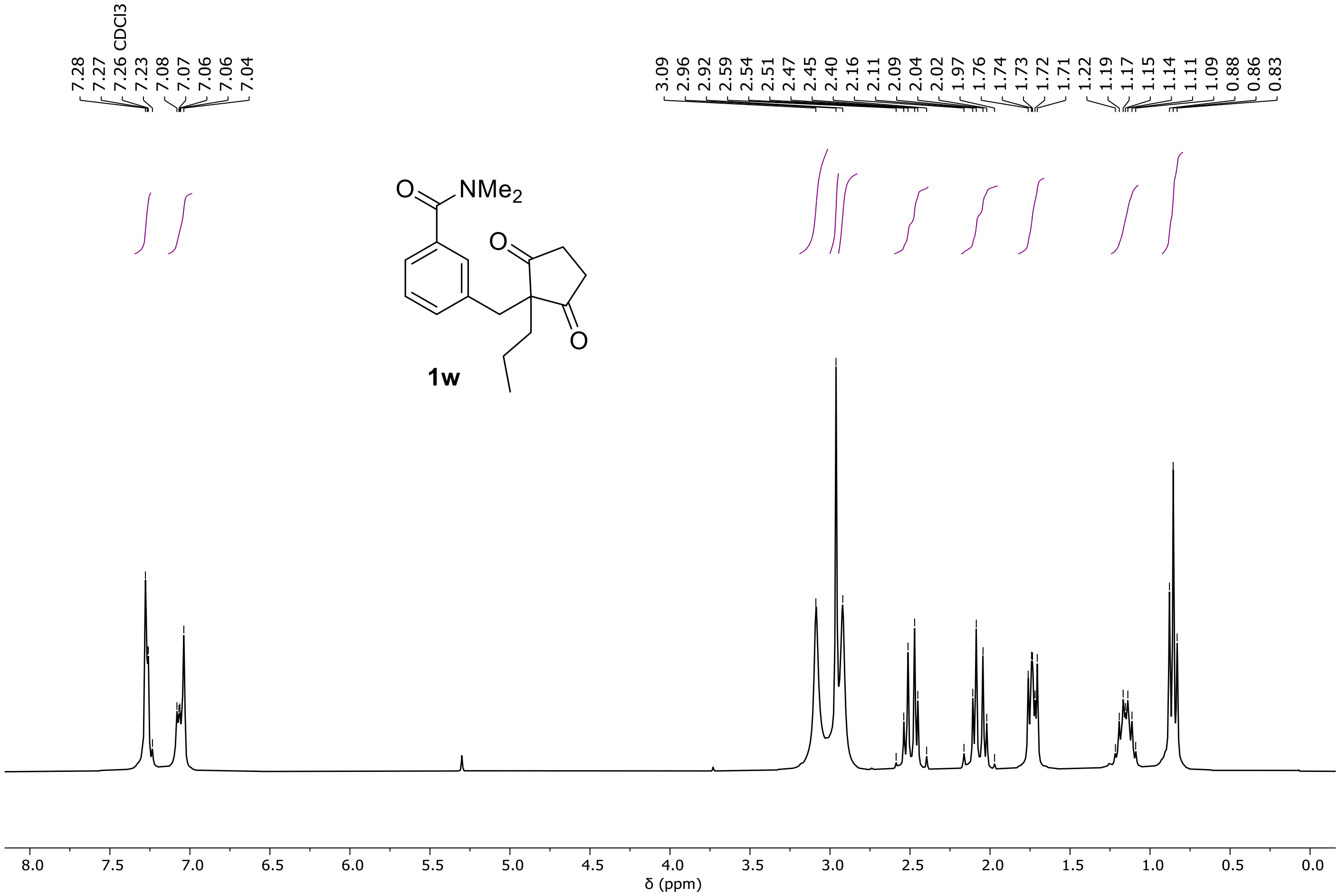

DEPT-135 NMR (75 MHz, CDCl3)

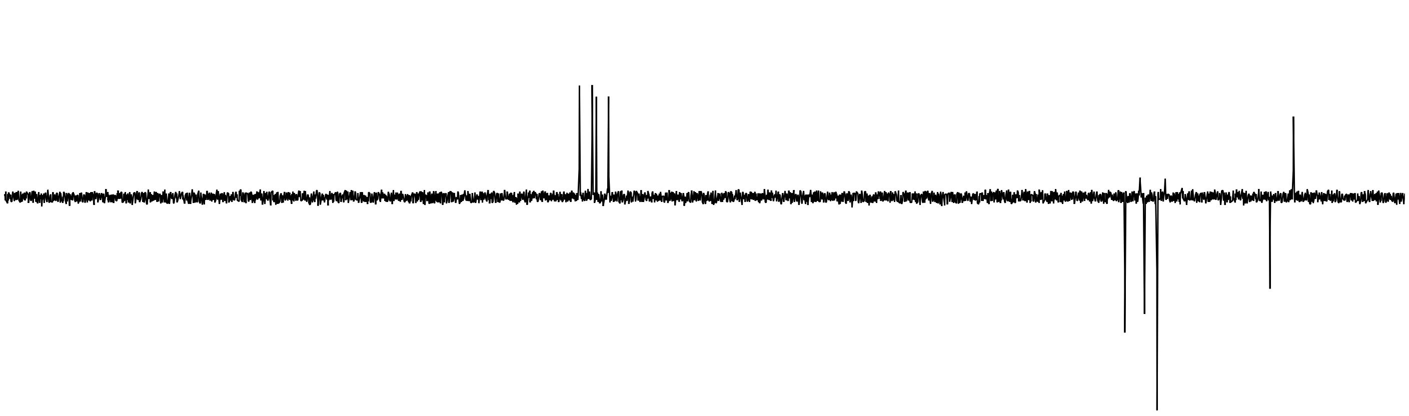

13C NMR (75 MHz, CDCl3)

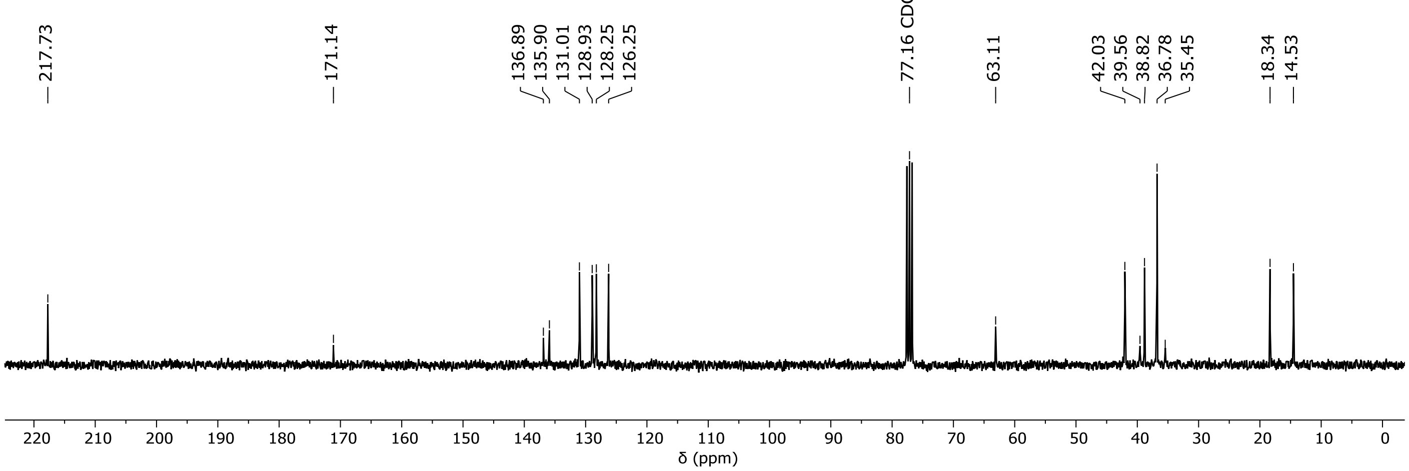

1H NMR (300 MHz, CDCl3)

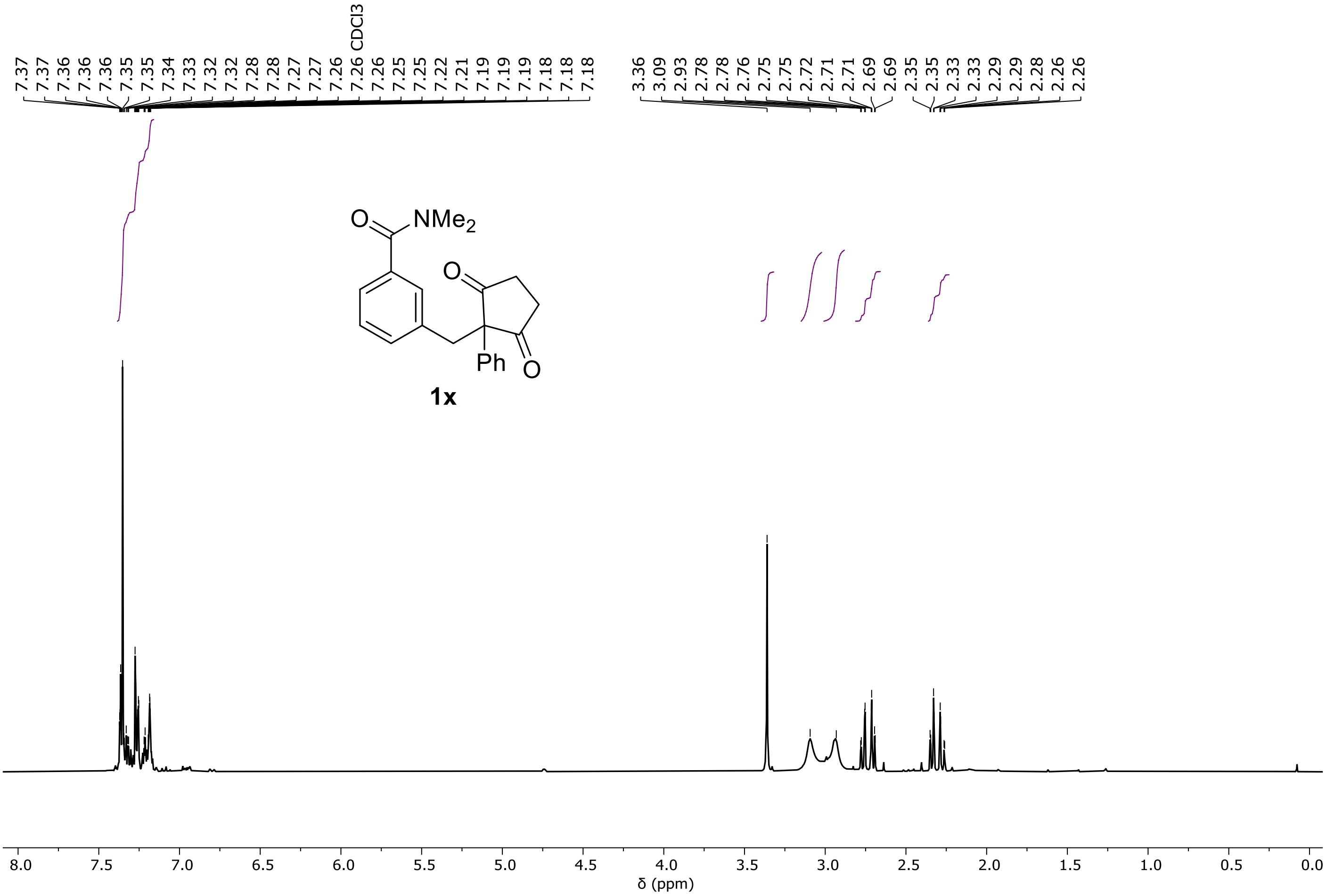

DEPT-135 NMR (75 MHz, CDCl3)

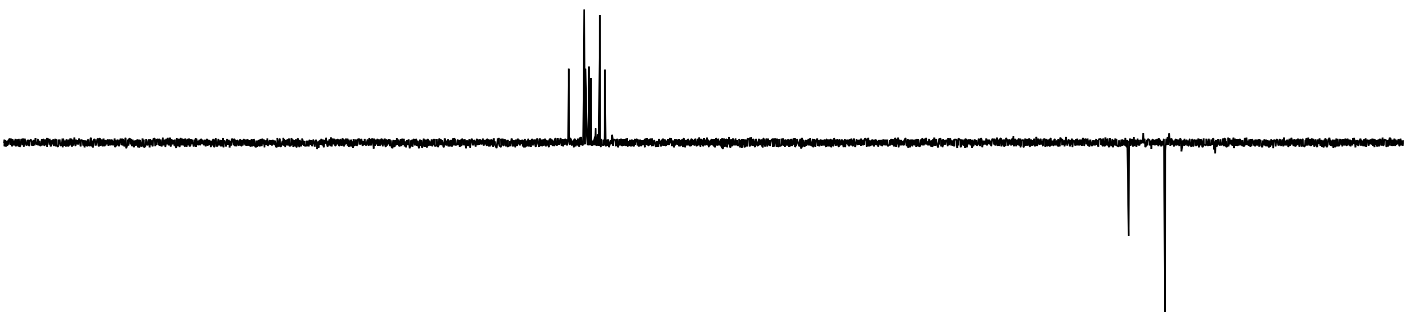

13C NMR (75 MHz, CDCl3)

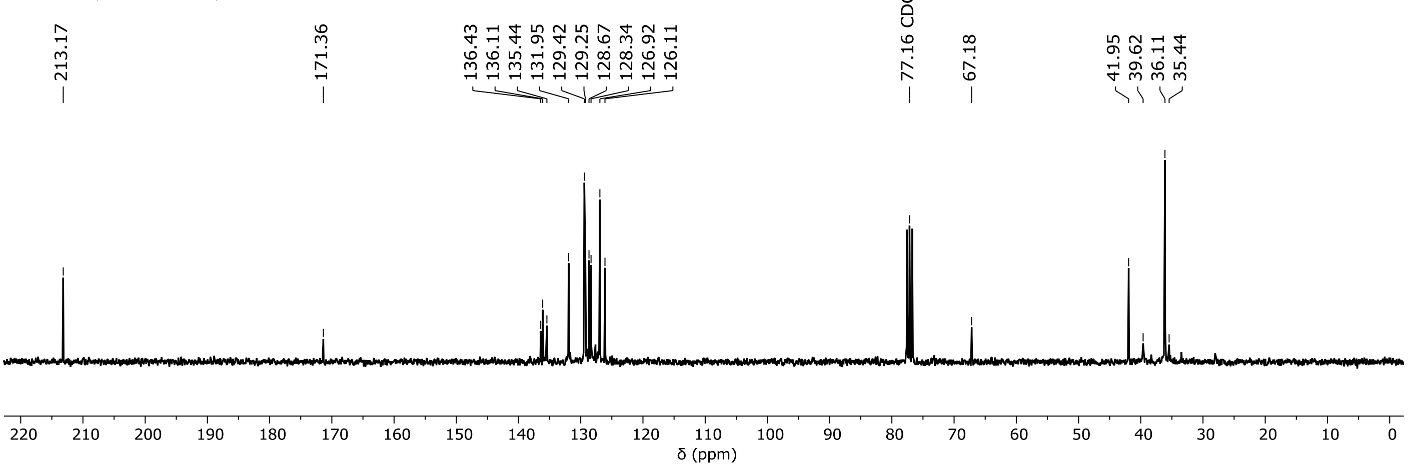

1H NMR (300 MHz, CDCl3)

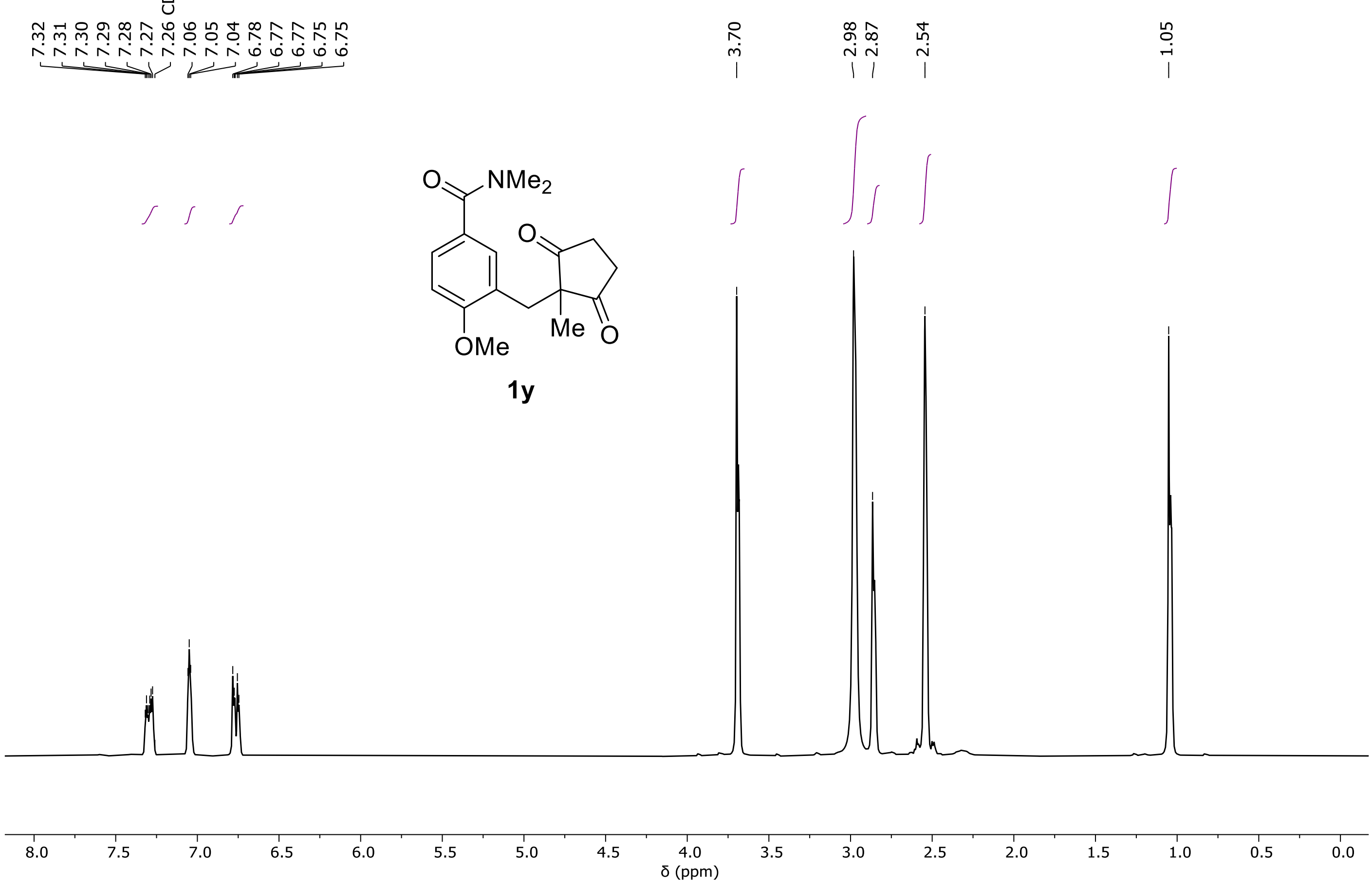

DEPT-135 NMR (75 MHz, CDCl3)

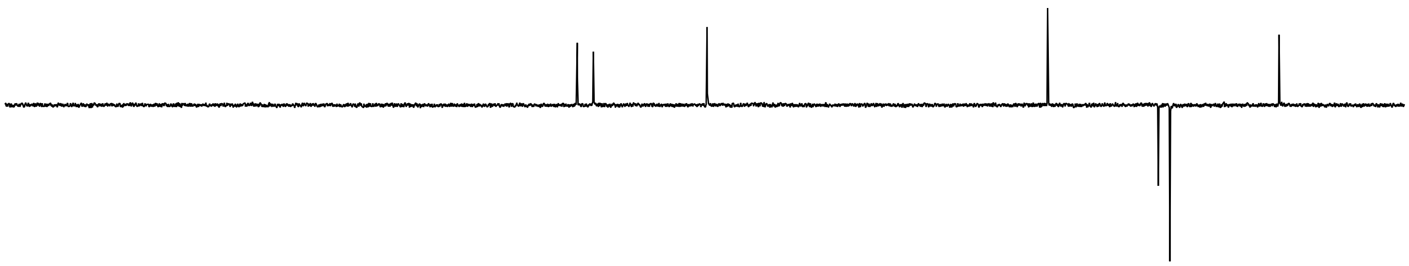

13C NMR (75 MHz, CDCl3)

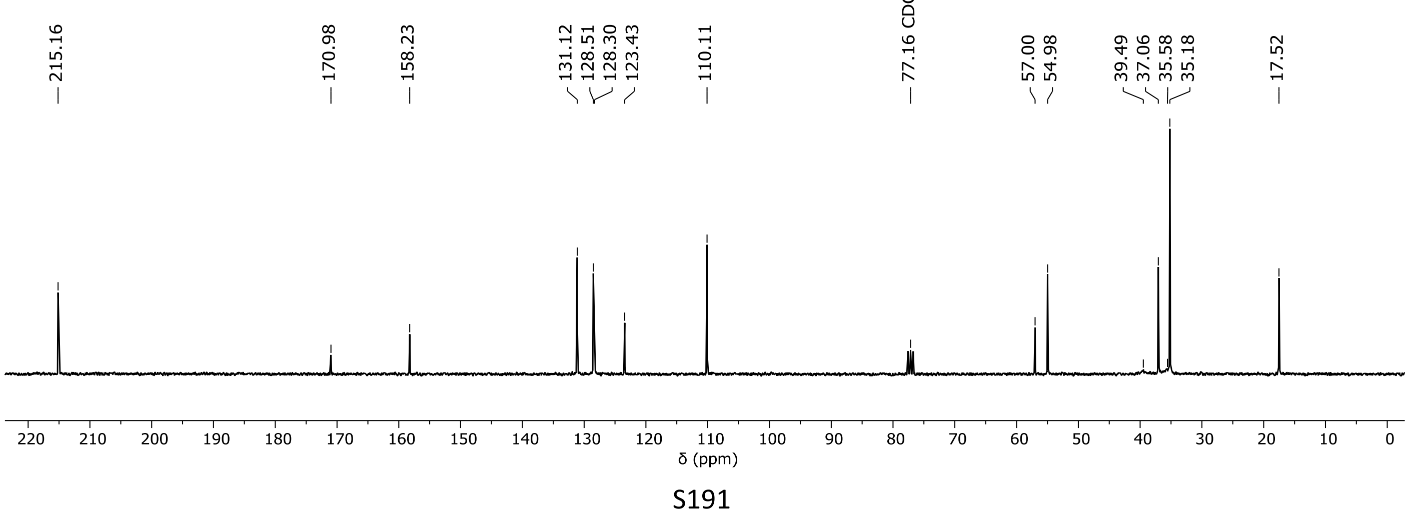

1H NMR (300 MHz, CDCl3)

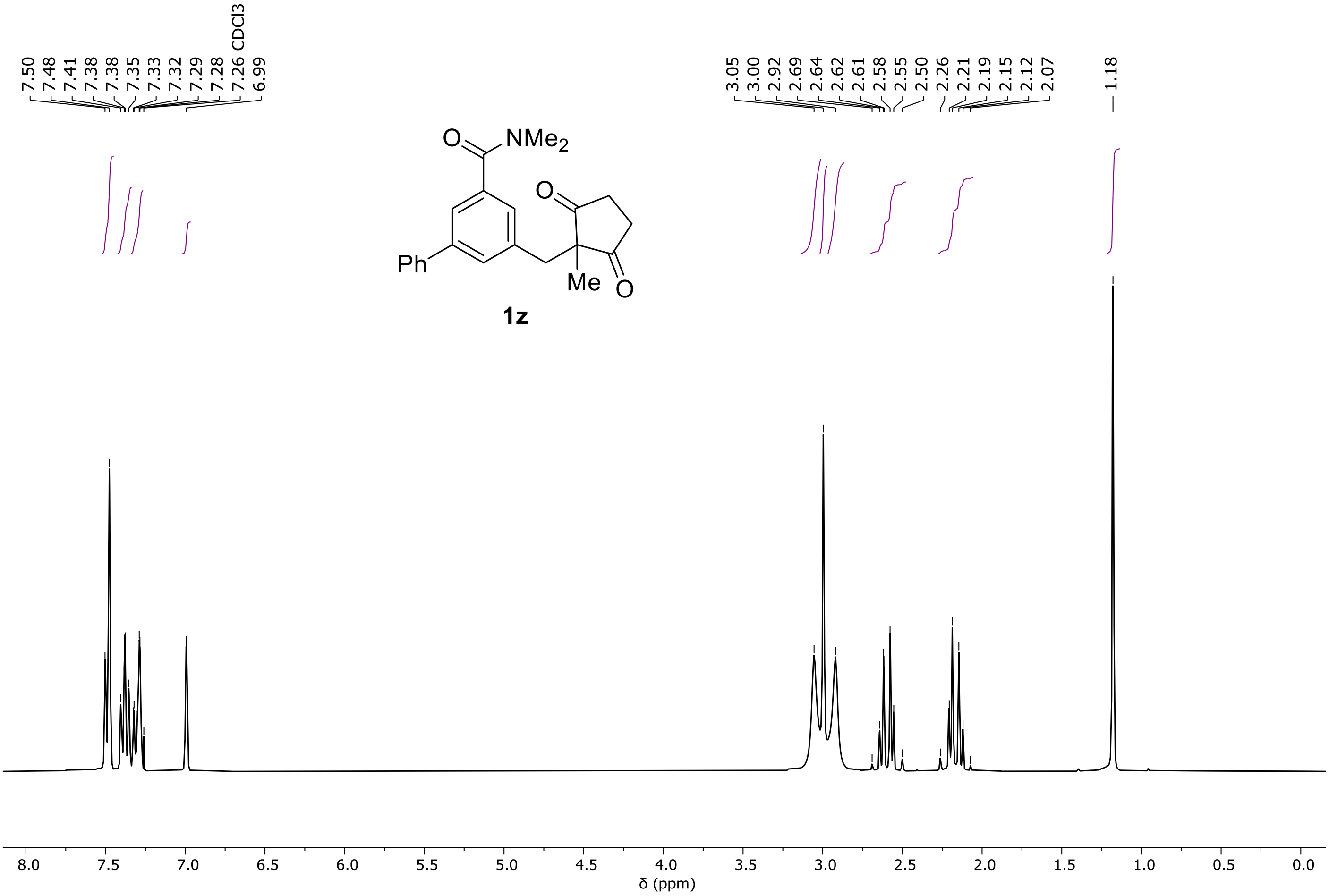

1z

DEPT-135 NMR (75 MHz, CDCl3)

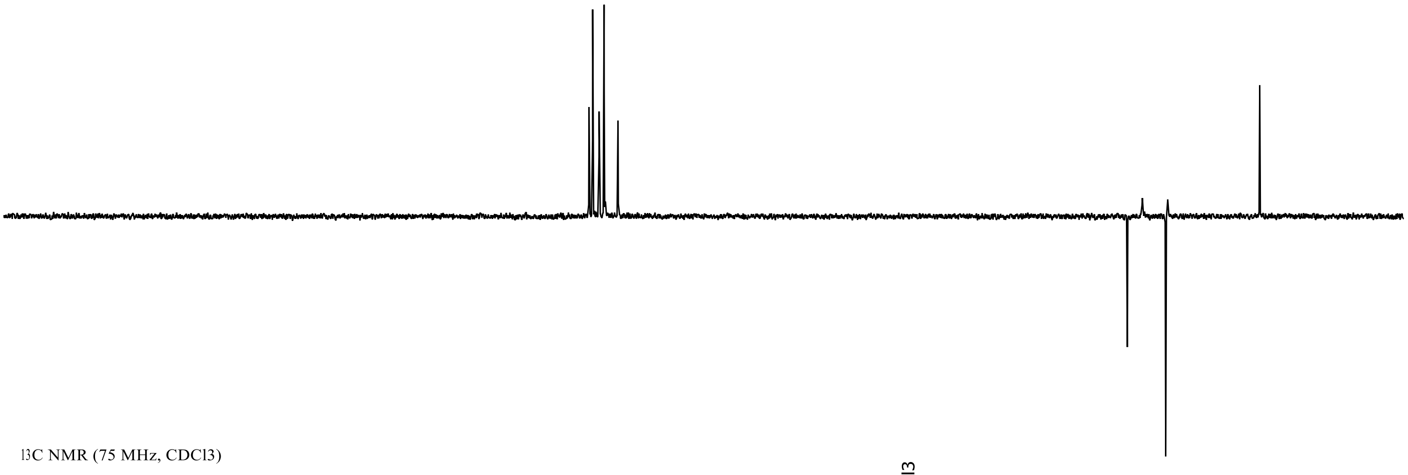

13C NMR (75 MHz, CDCl3)

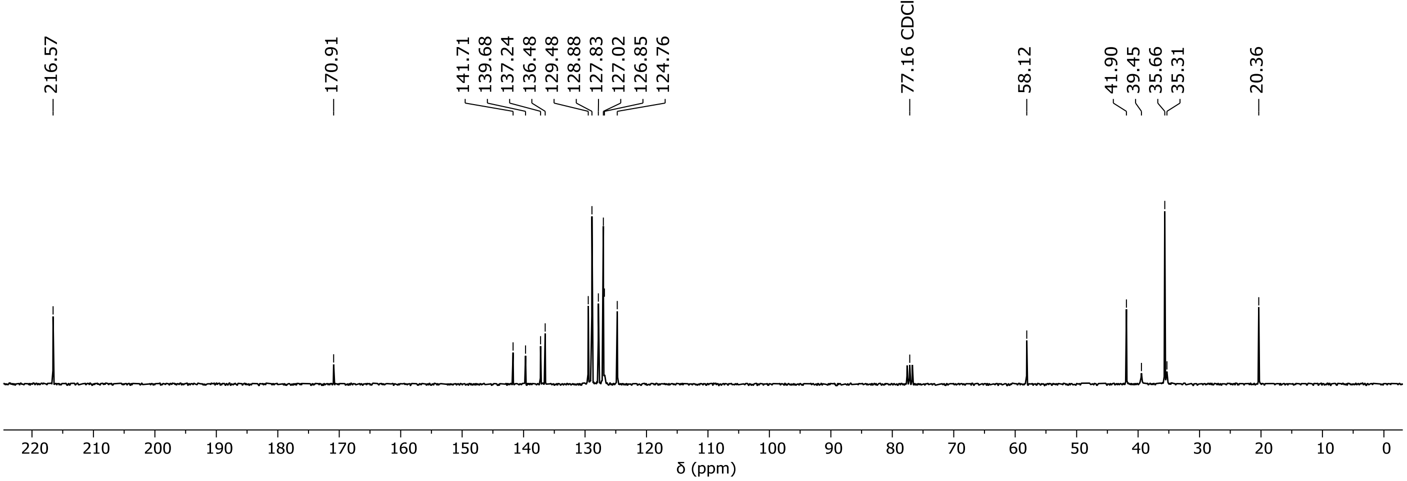

1H NMR (300 MHz, CDCl3)

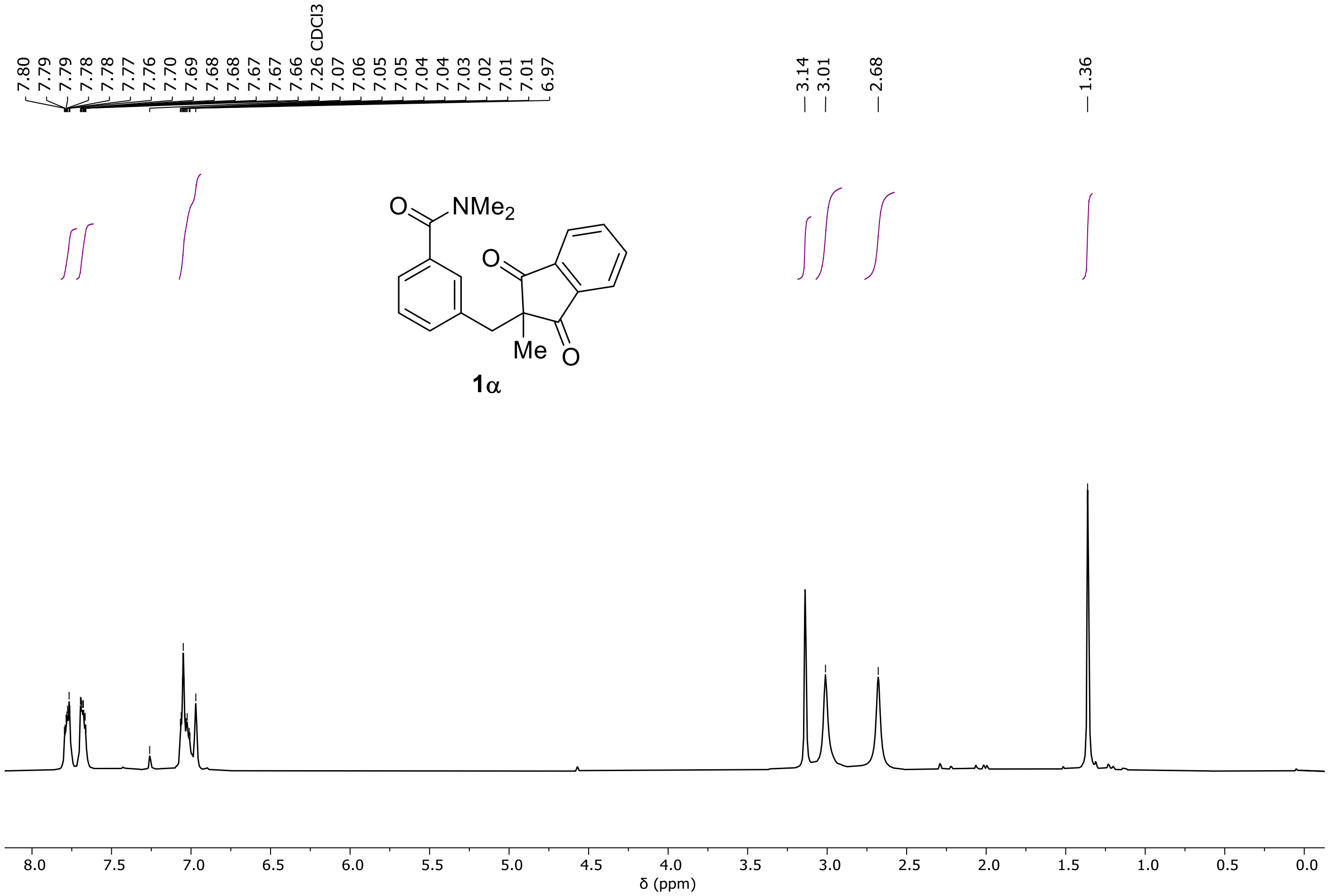

DEPT-135 NMR (75 MHz, CDCl3)

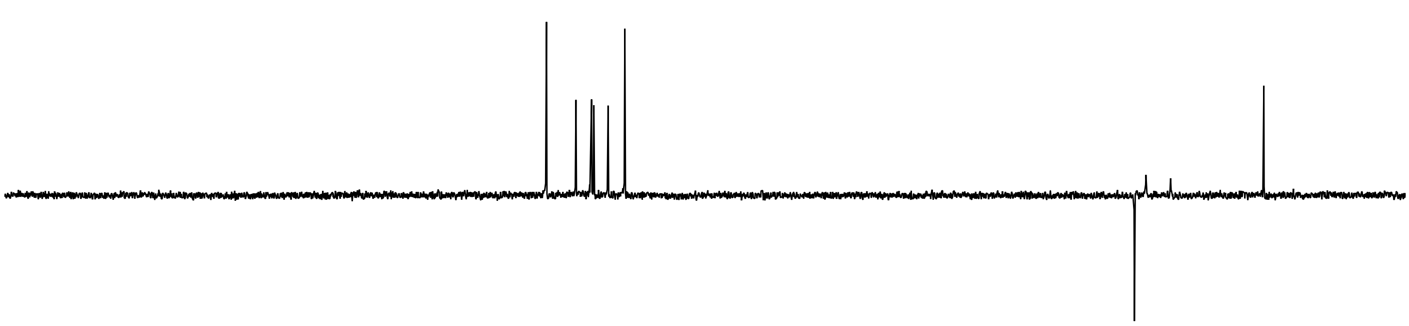

13C NMR (75 MHz, CDCl3)

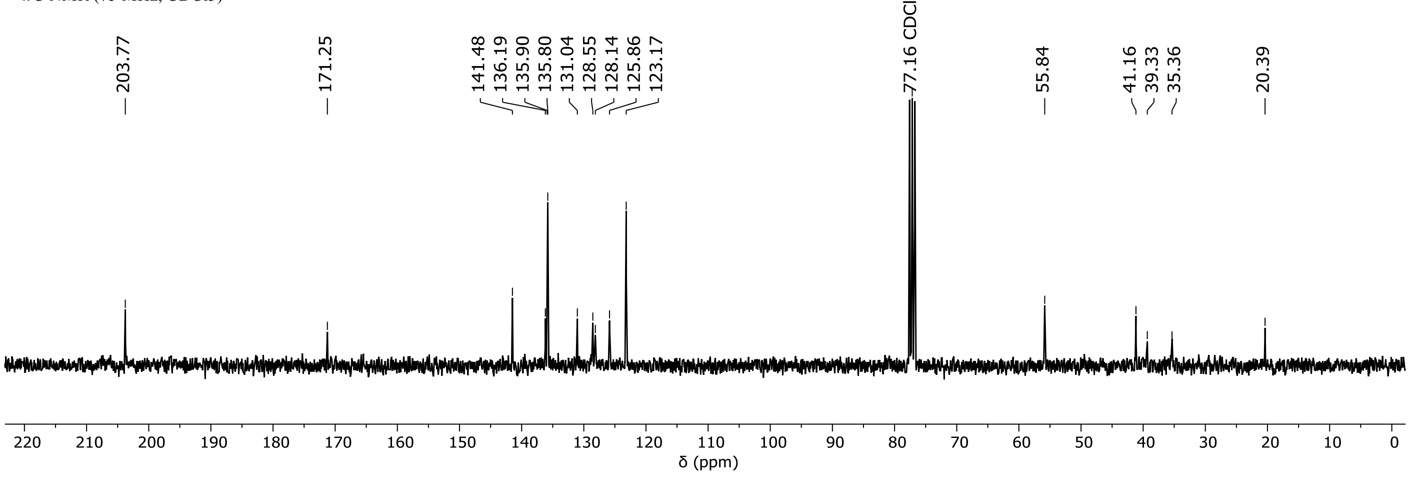

1H NMR (300 MHz, CDCl3)

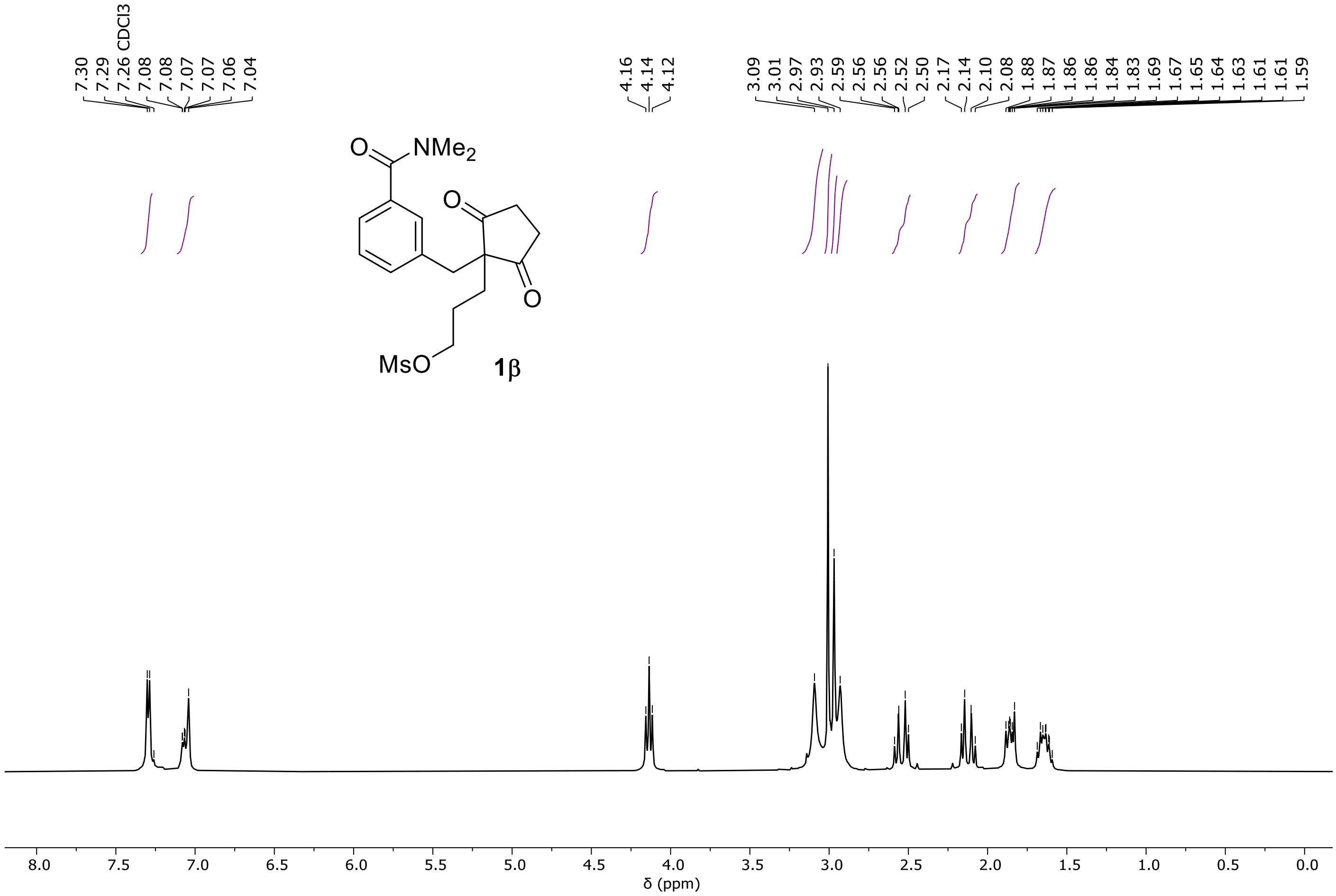

DEPT-135 NMR (75 MHz, CDCl3)

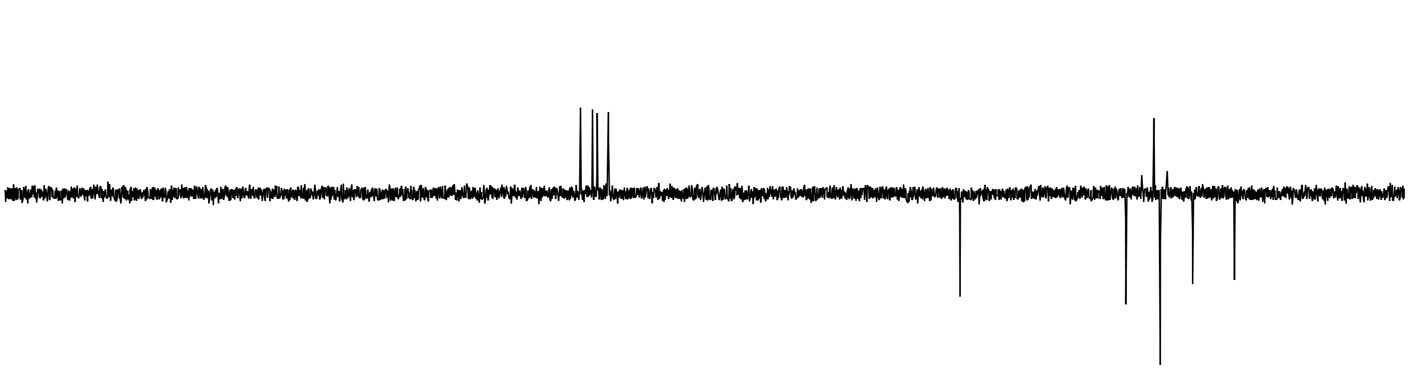

13C NMR (75 MHz, CDCl3)

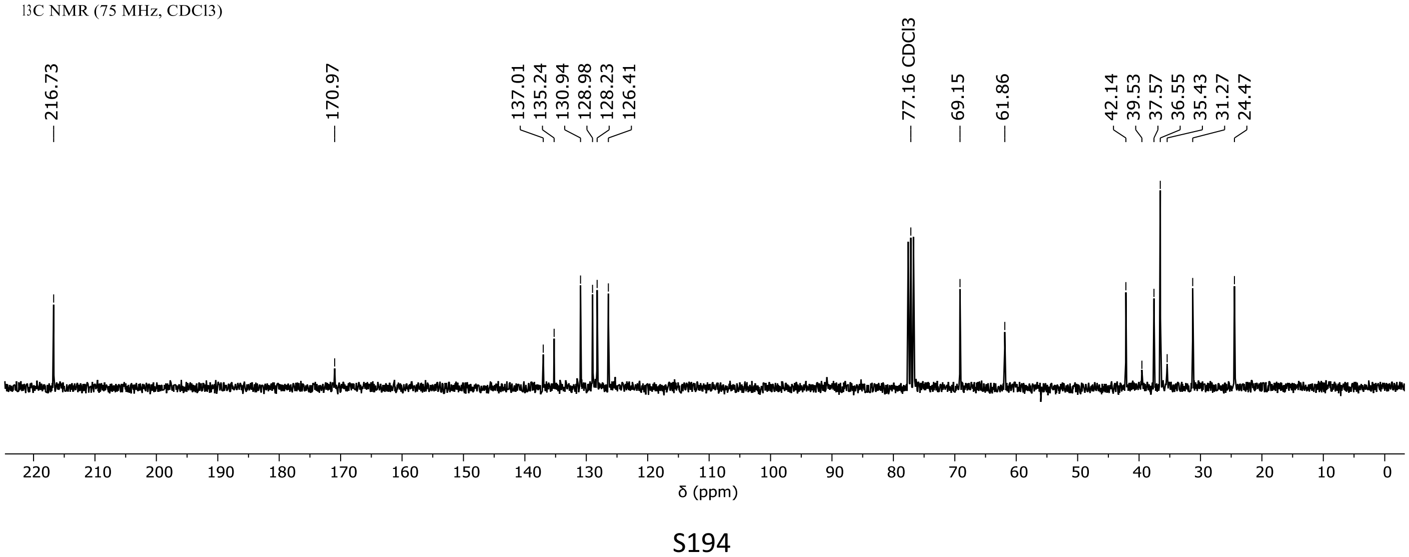

1H NMR (300 MHz, CDCl3)

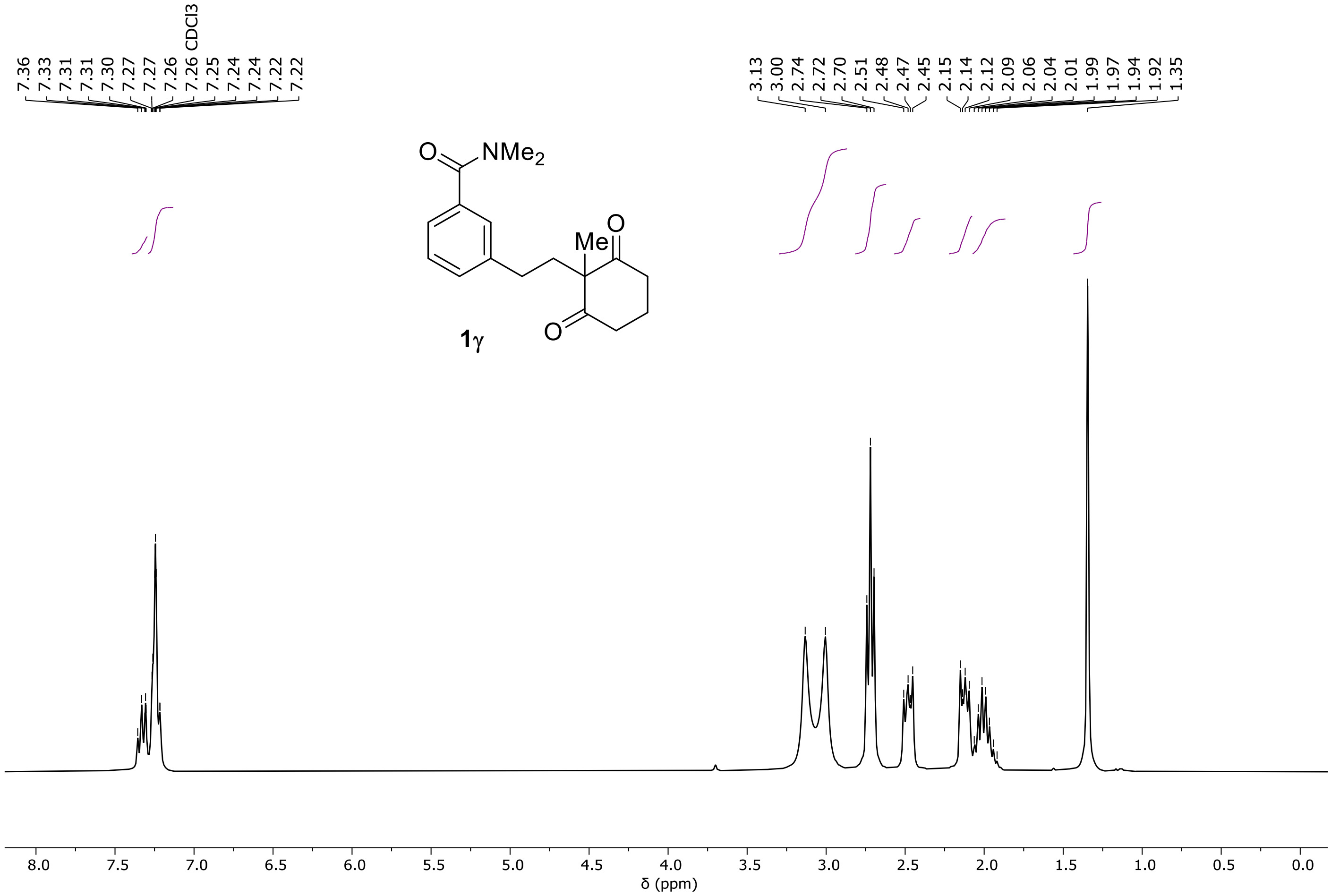

DEPT-135 NMR (75 MHz, CDCl3)

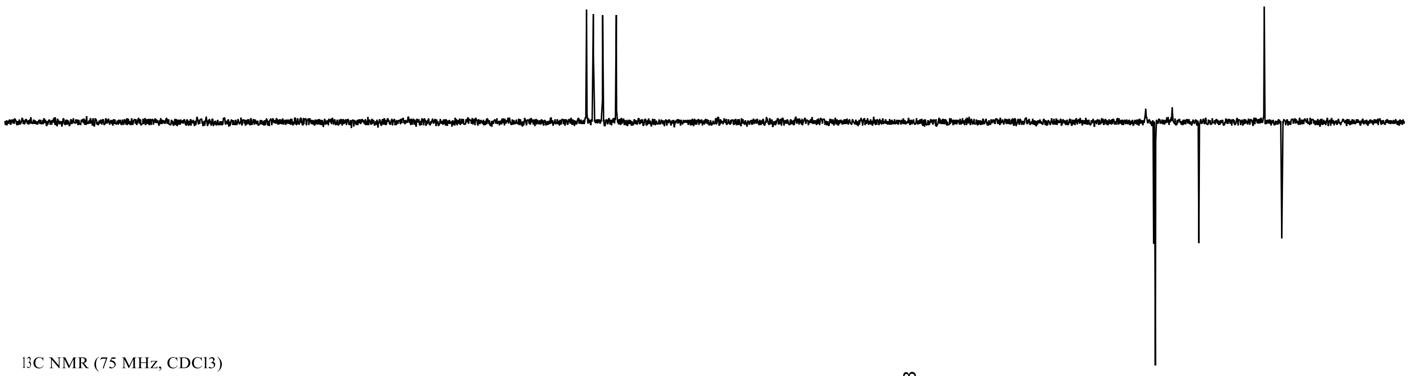

13C NMR (75 MHz, CDCl3)

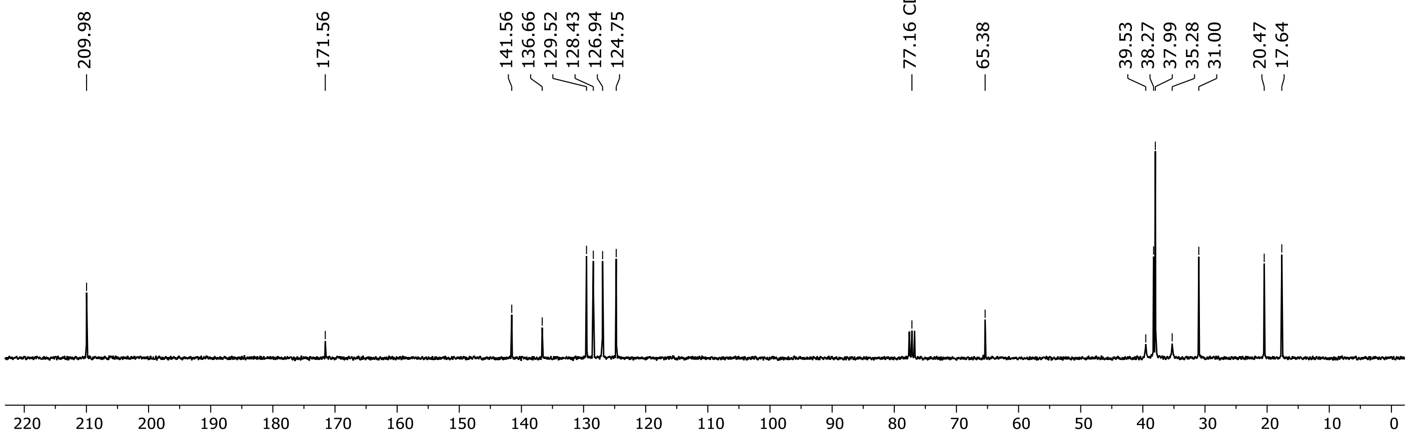

1H NMR (300 MHz, CDCl3)

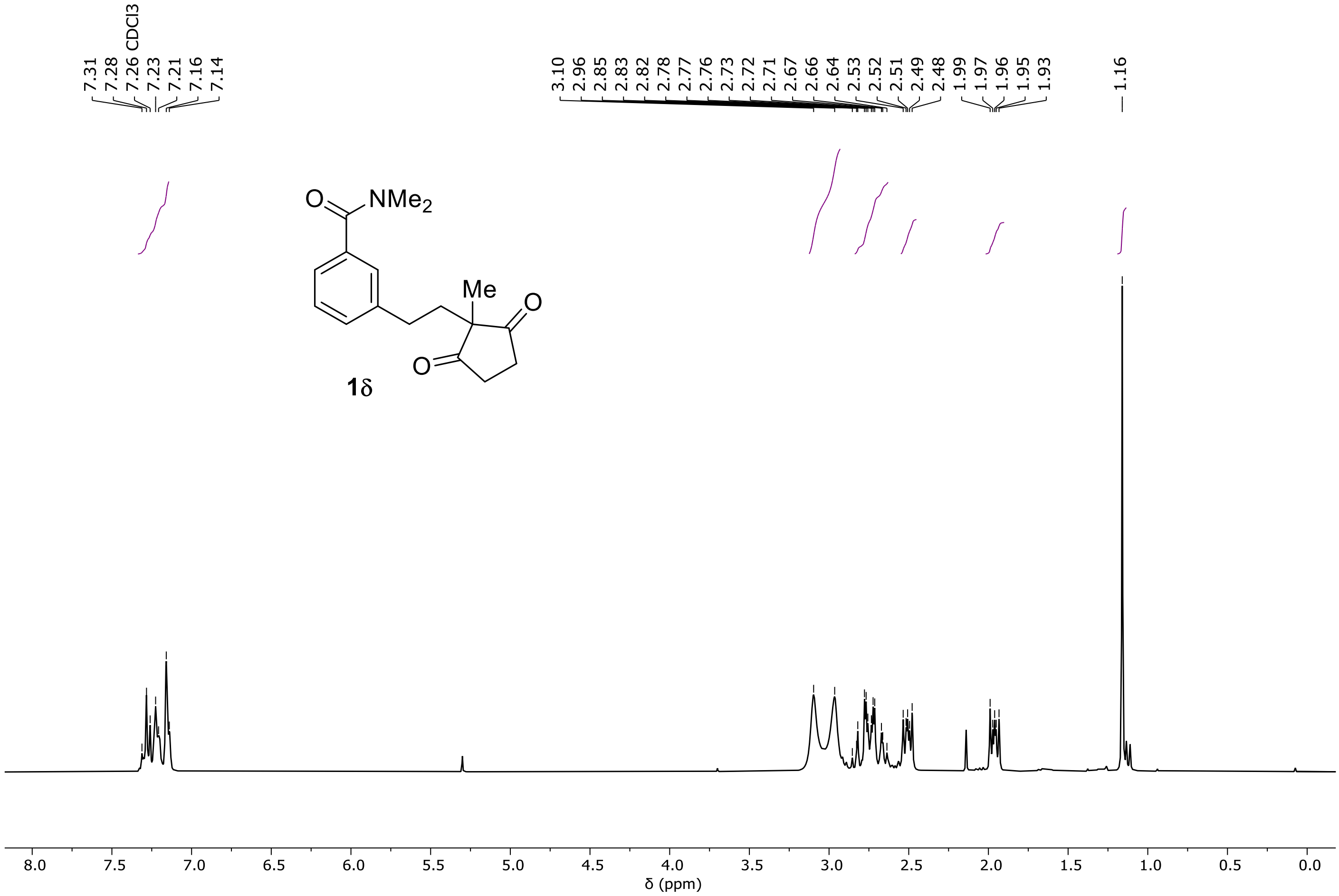

DEPT-135 NMR (75 MHz, CDCl3)

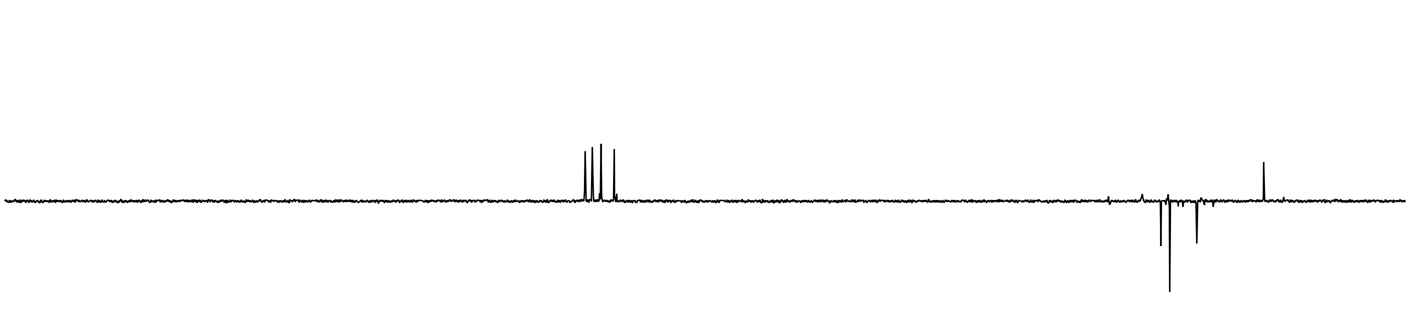

13C NMR (75 MHz, CDCl3)

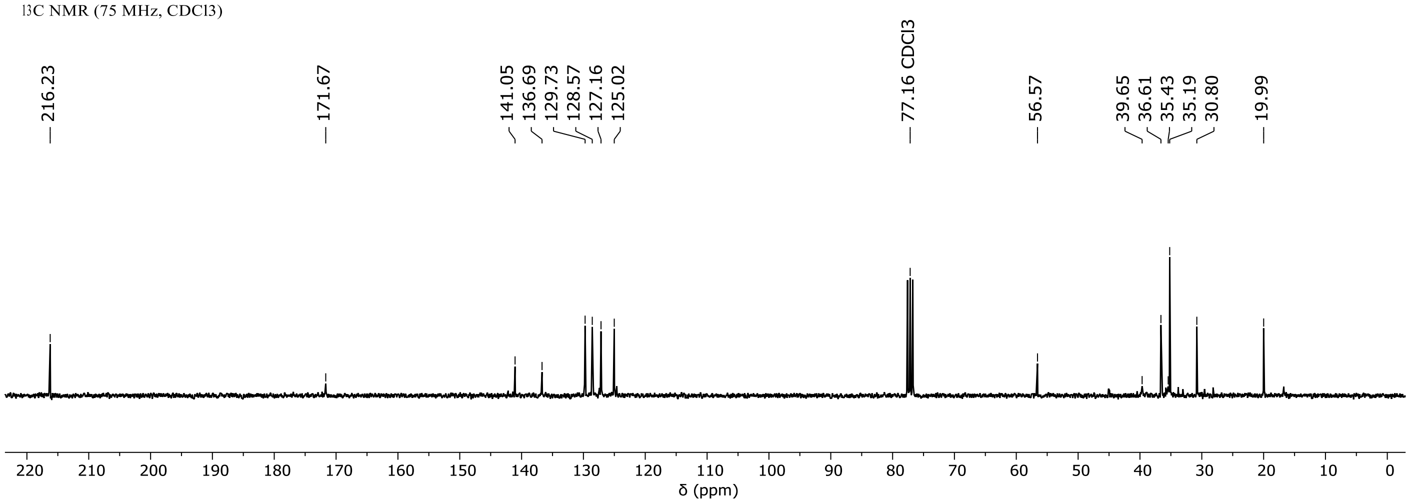

1H NMR (300 MHz, CDCl3)

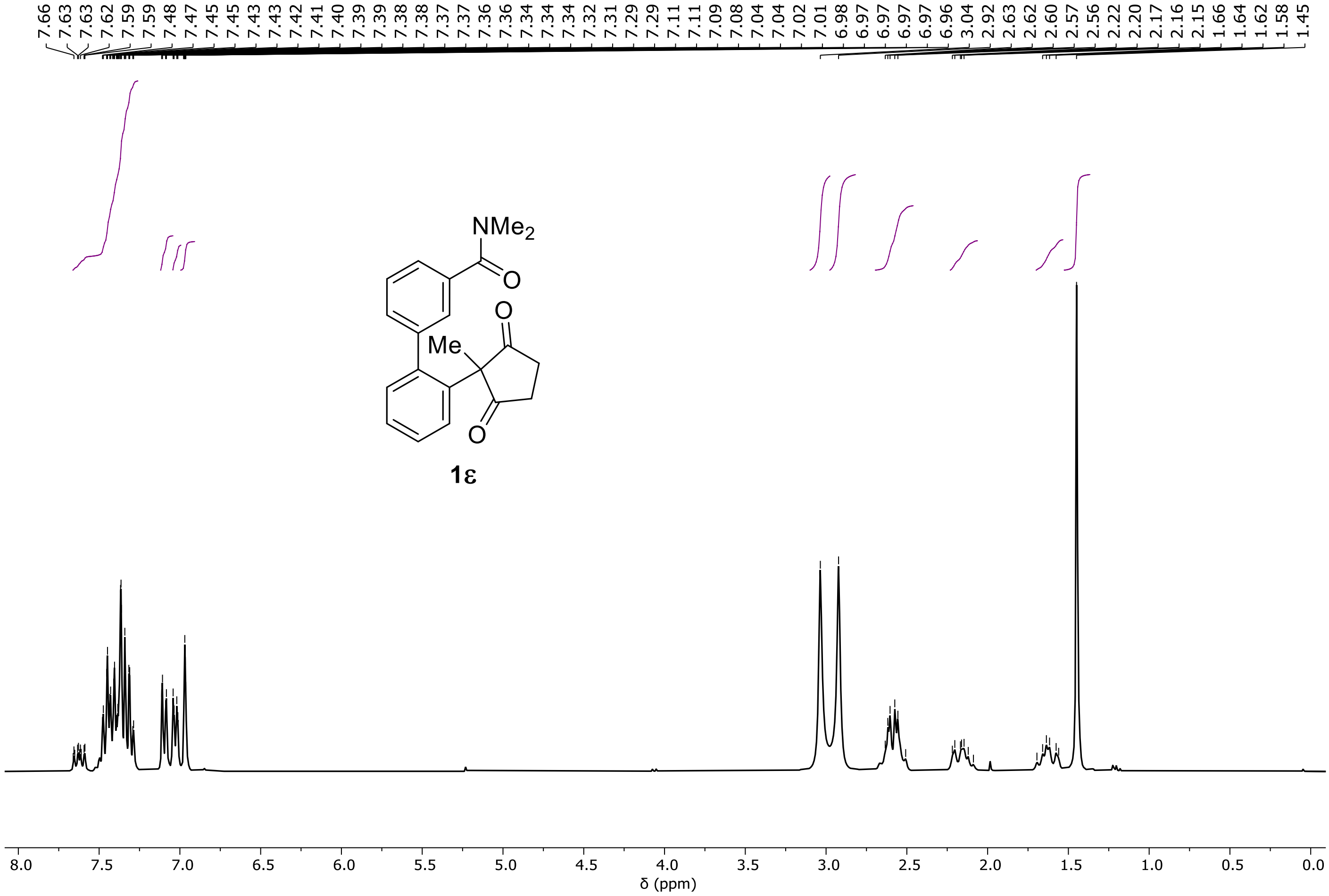

DEPT-135 NMR (75 MHz, CDCl3)

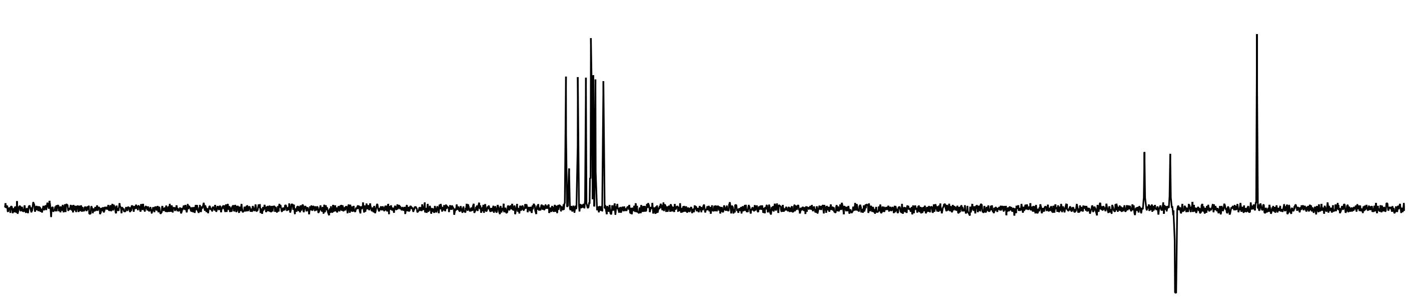

13C NMR (75 MHz, CDCl3)

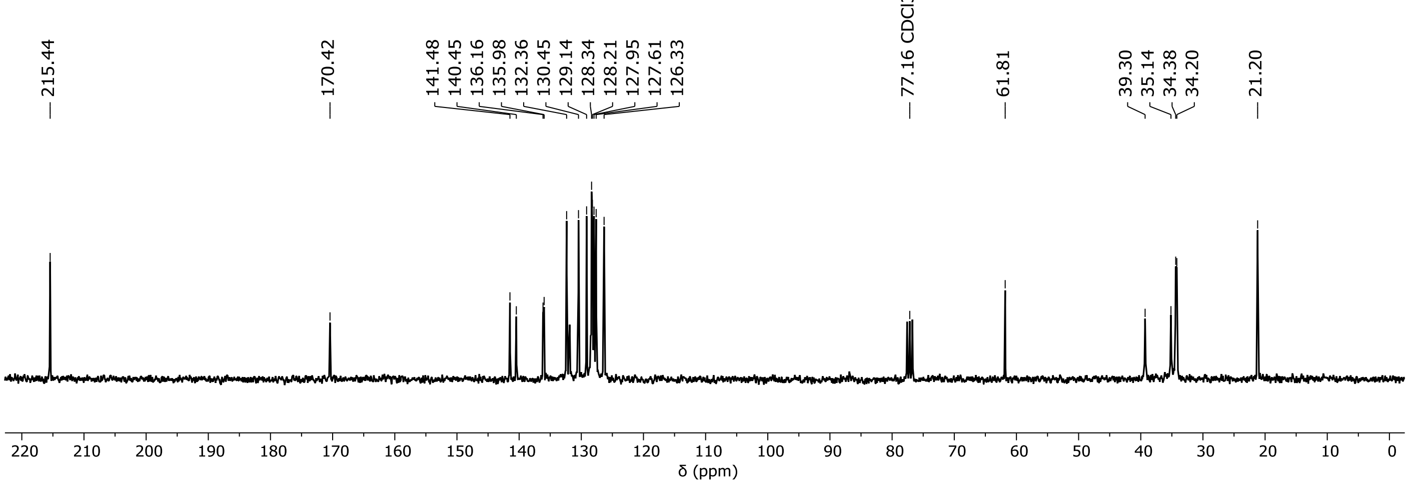

1H NMR (300 MHz, CDCl3)

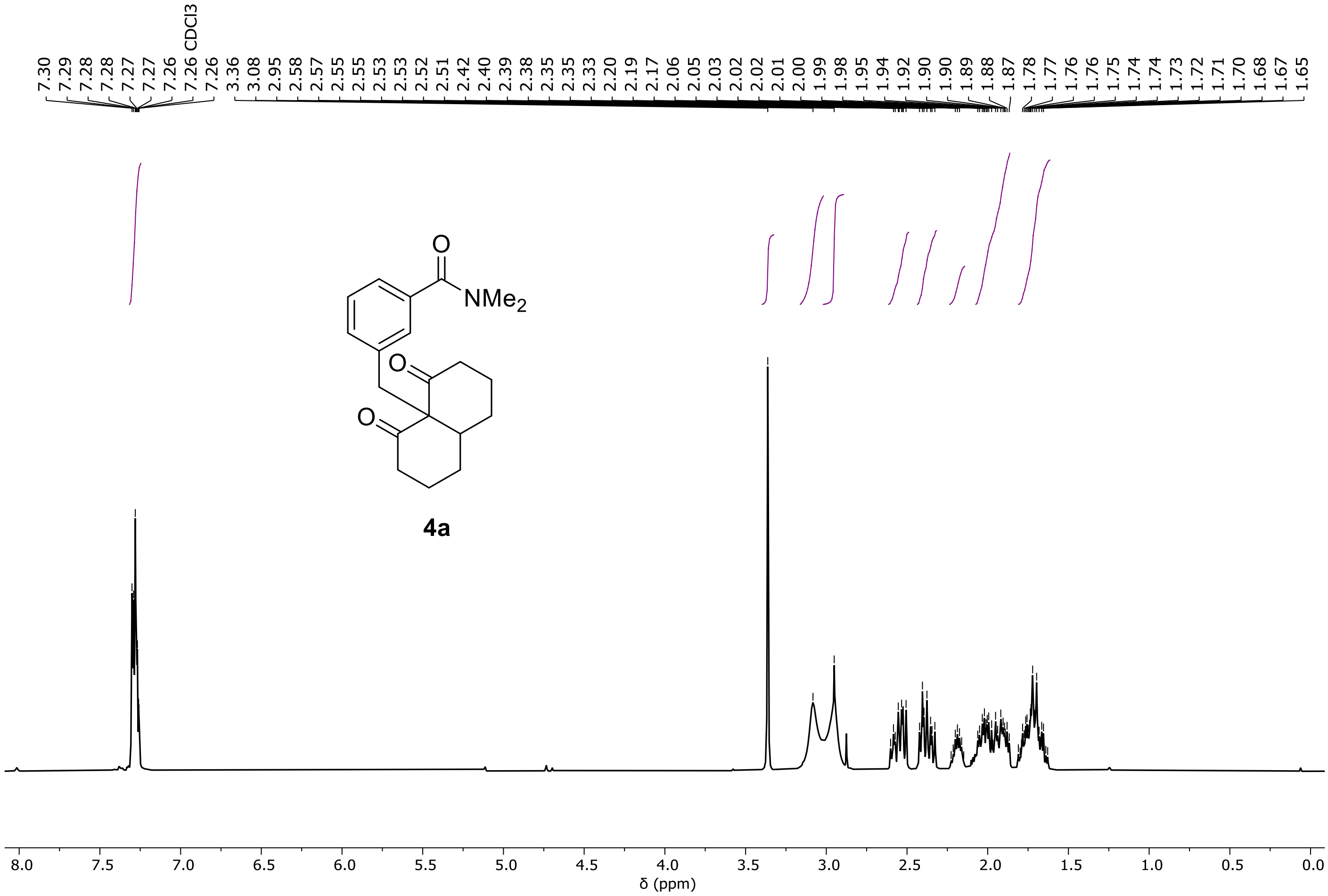

4a

DEPT-135 NMR (75 MHz, CDCl3)

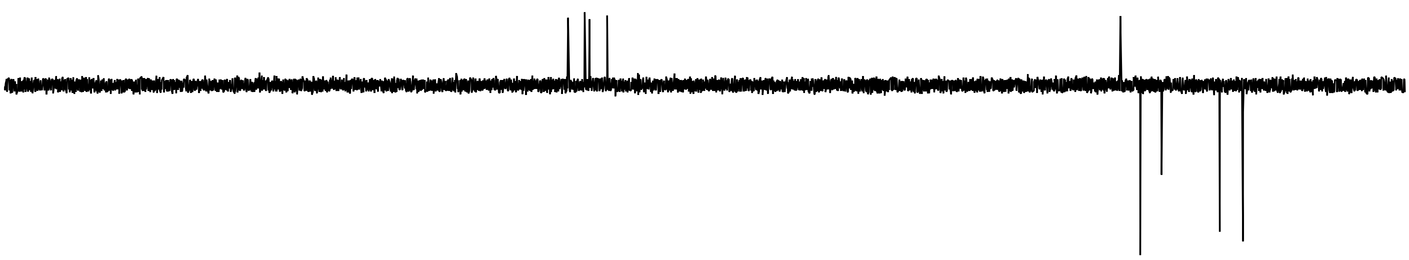

13C NMR (75 MHz, CDCl3)

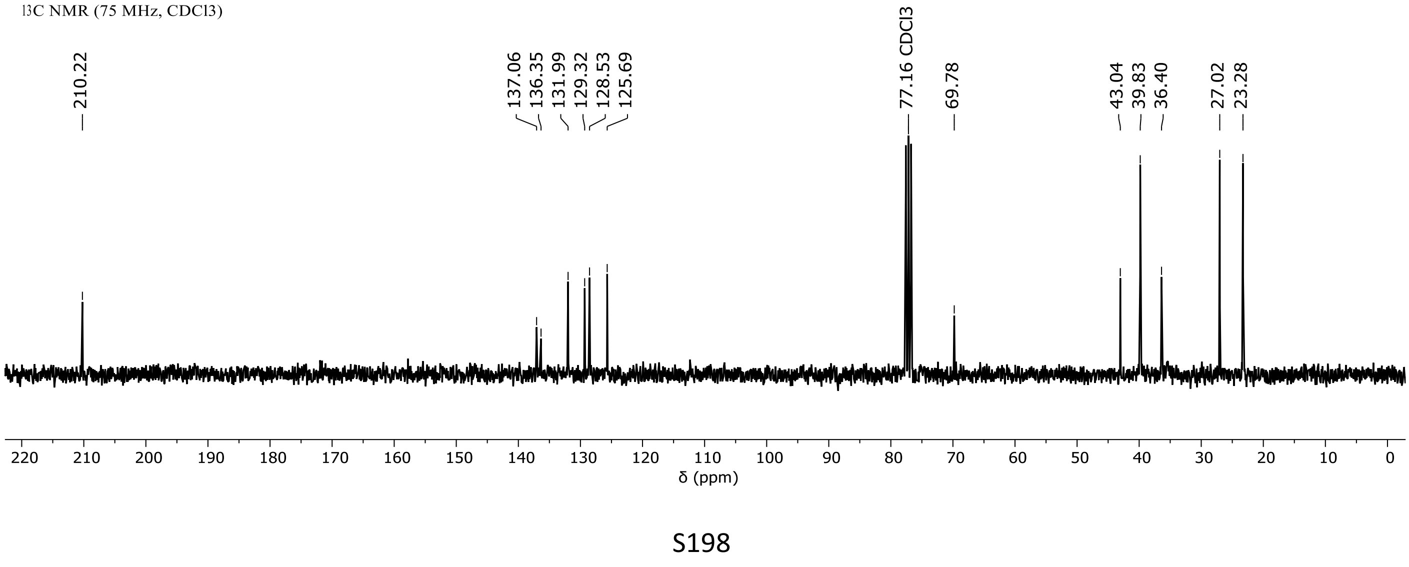

1H NMR (300 MHz, CDCl3)

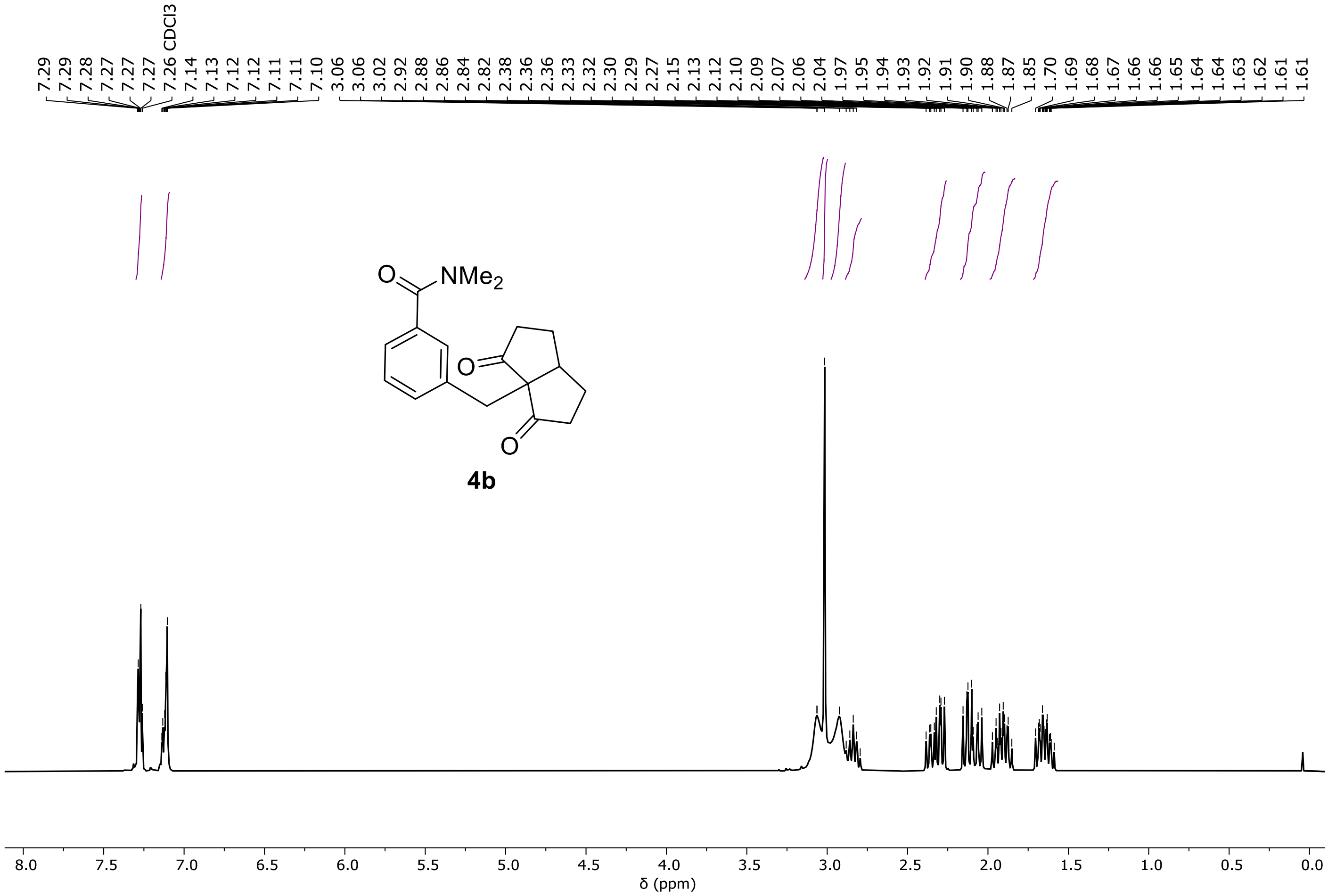

DEPT-135 NMR (75 MHz, CDCl3)

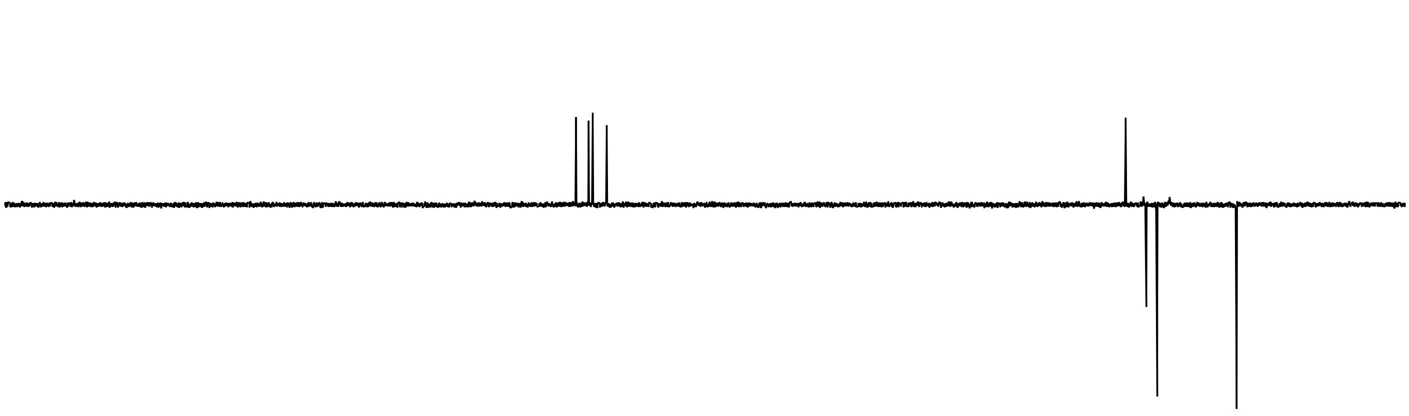

13C NMR (75 MHz, CDCl3)

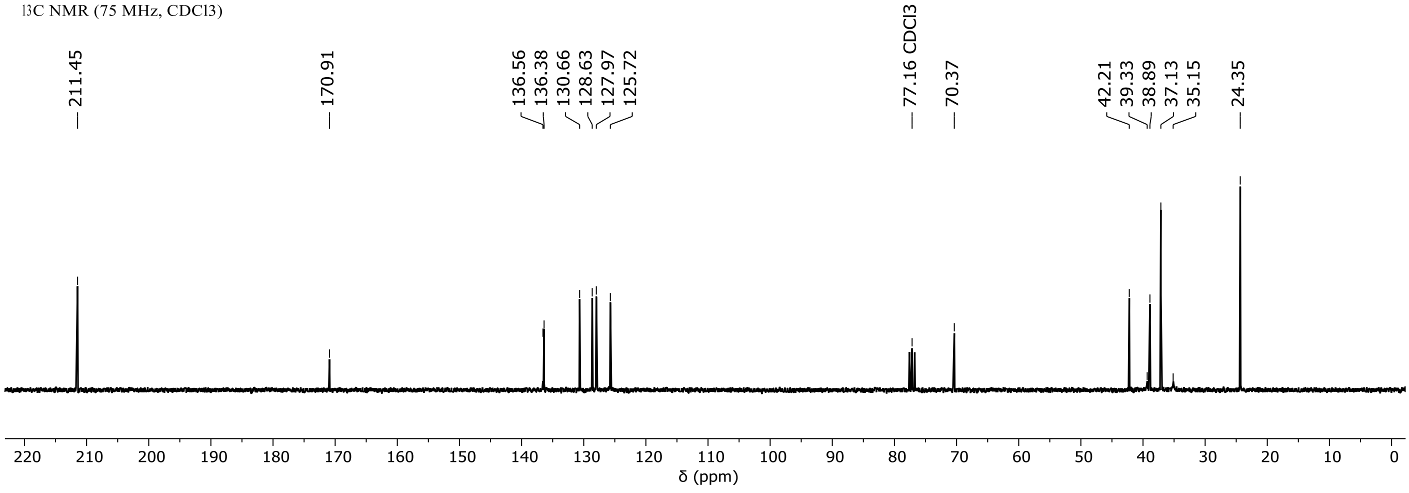

1H NMR (300 MHz, CDCl3)

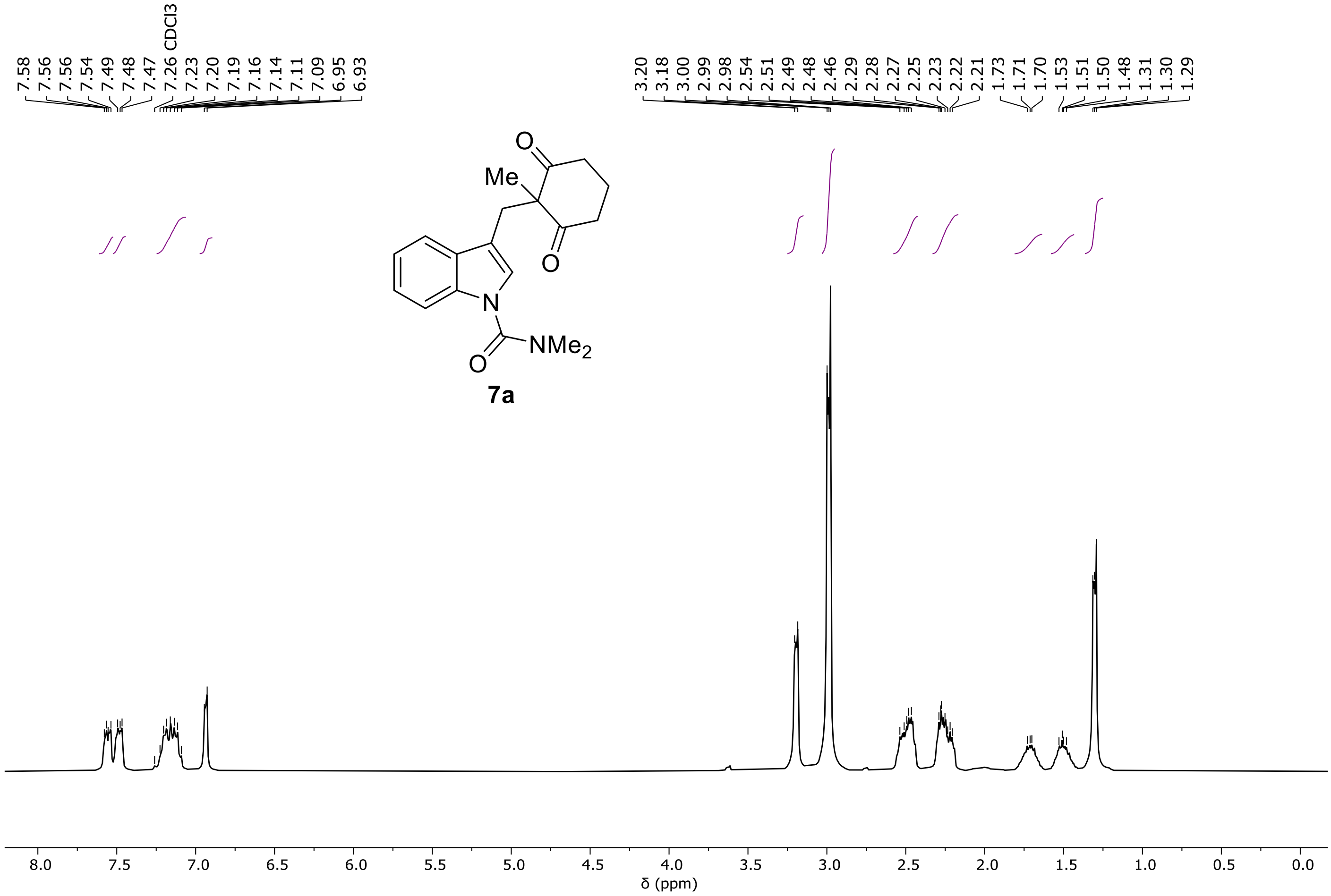

DEPT-135 NMR (75 MHz, CDCl3)

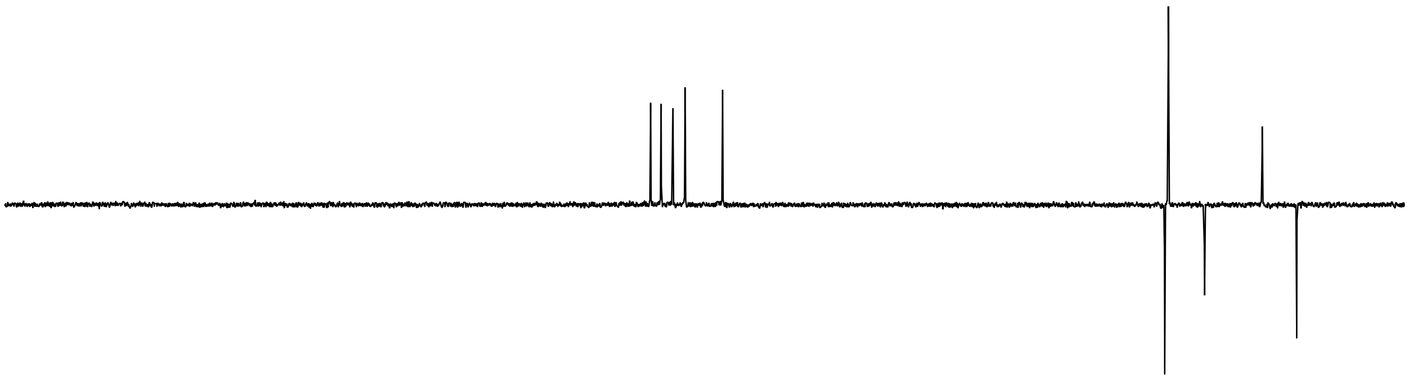

13C NMR (75 MHz, CDCl3)

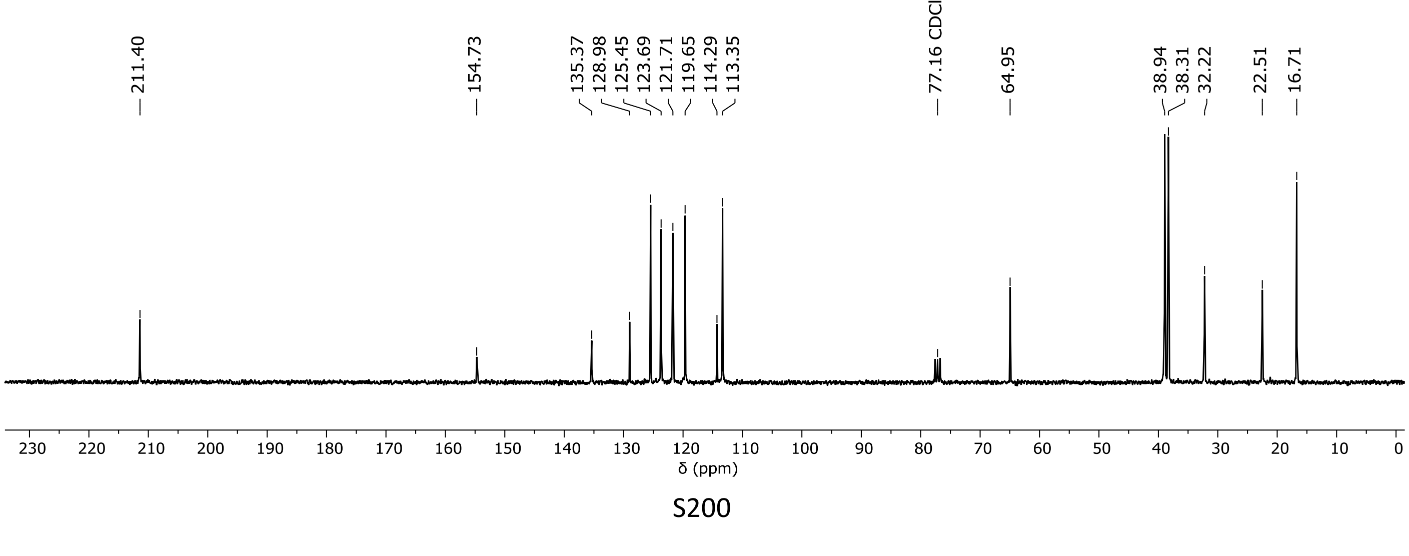

1H NMR (300 MHz, CDCl3)

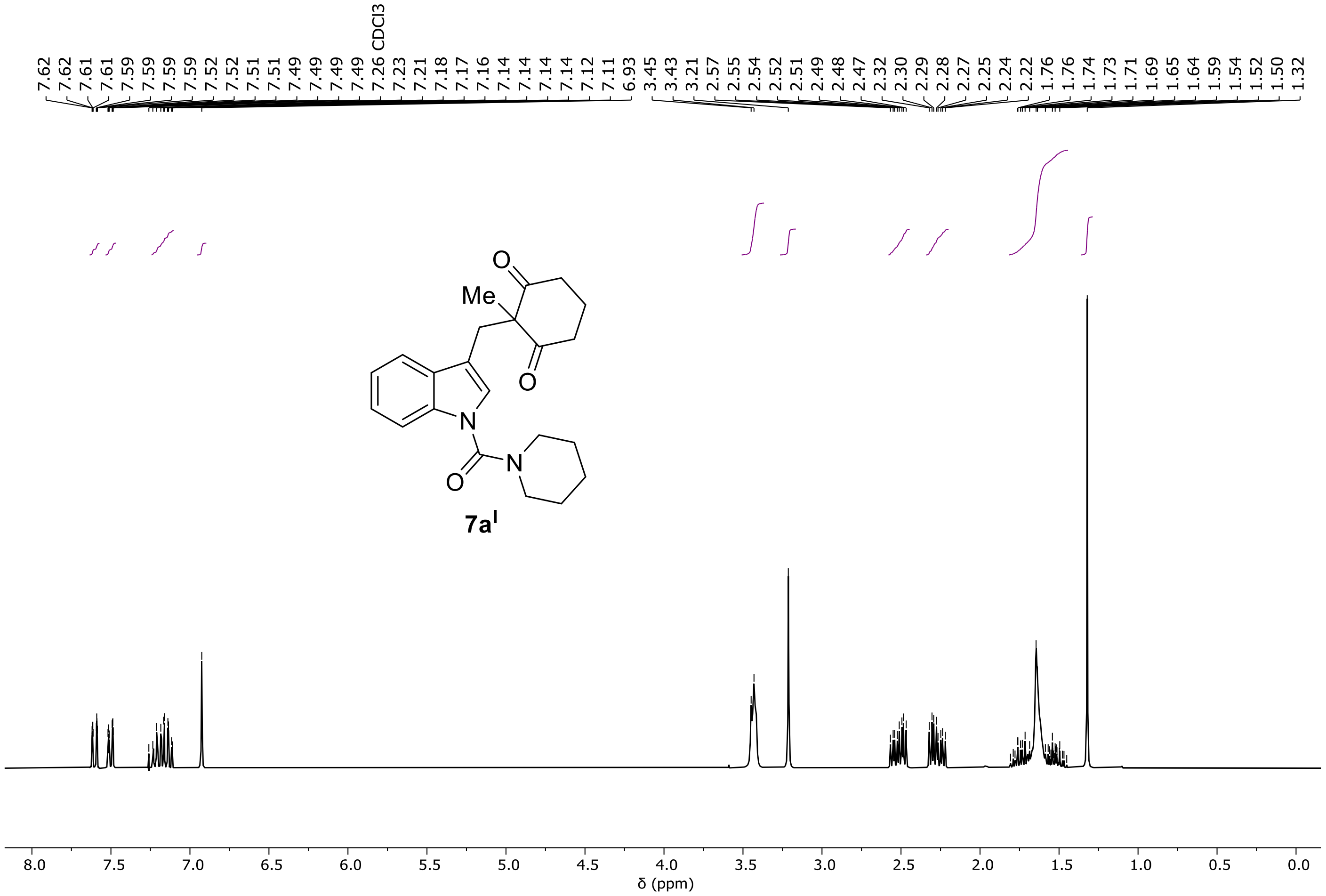

DEPT-135 NMR (75 MHz, CDCl3)

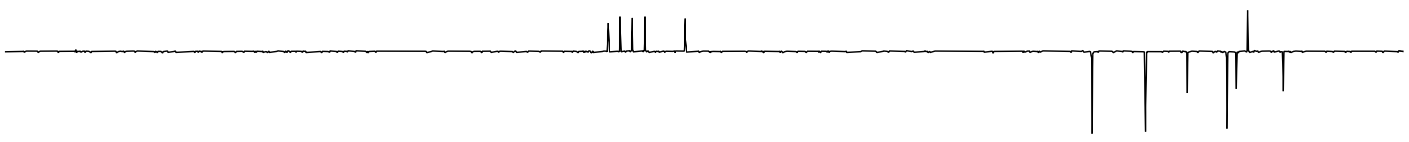

13C NMR (75 MHz, CDCl3)

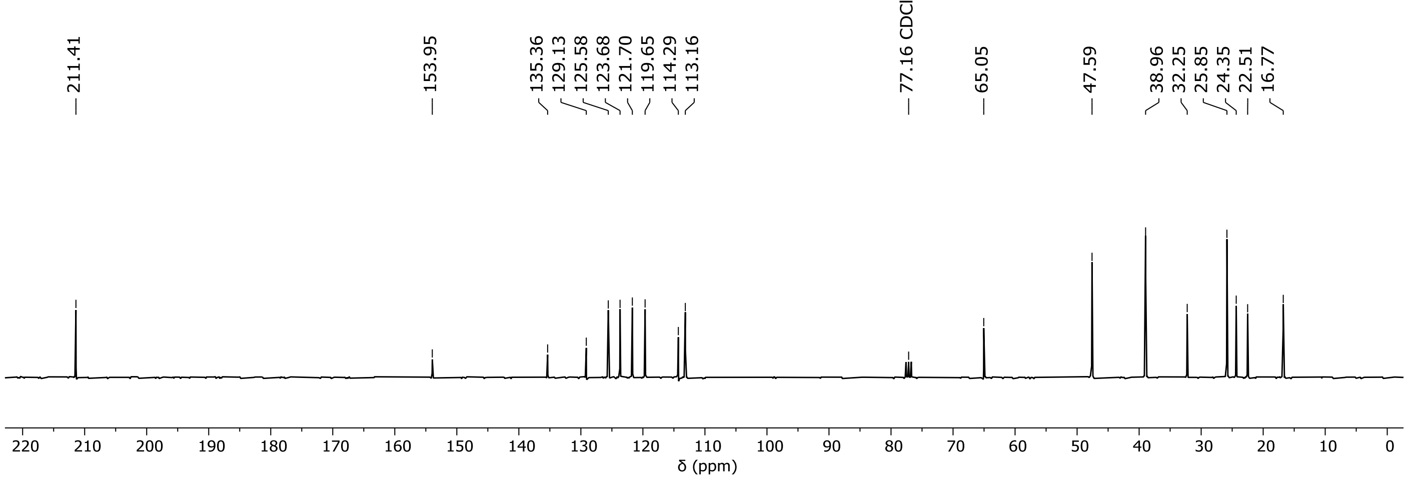

1H NMR (300 MHz, CDCl3)

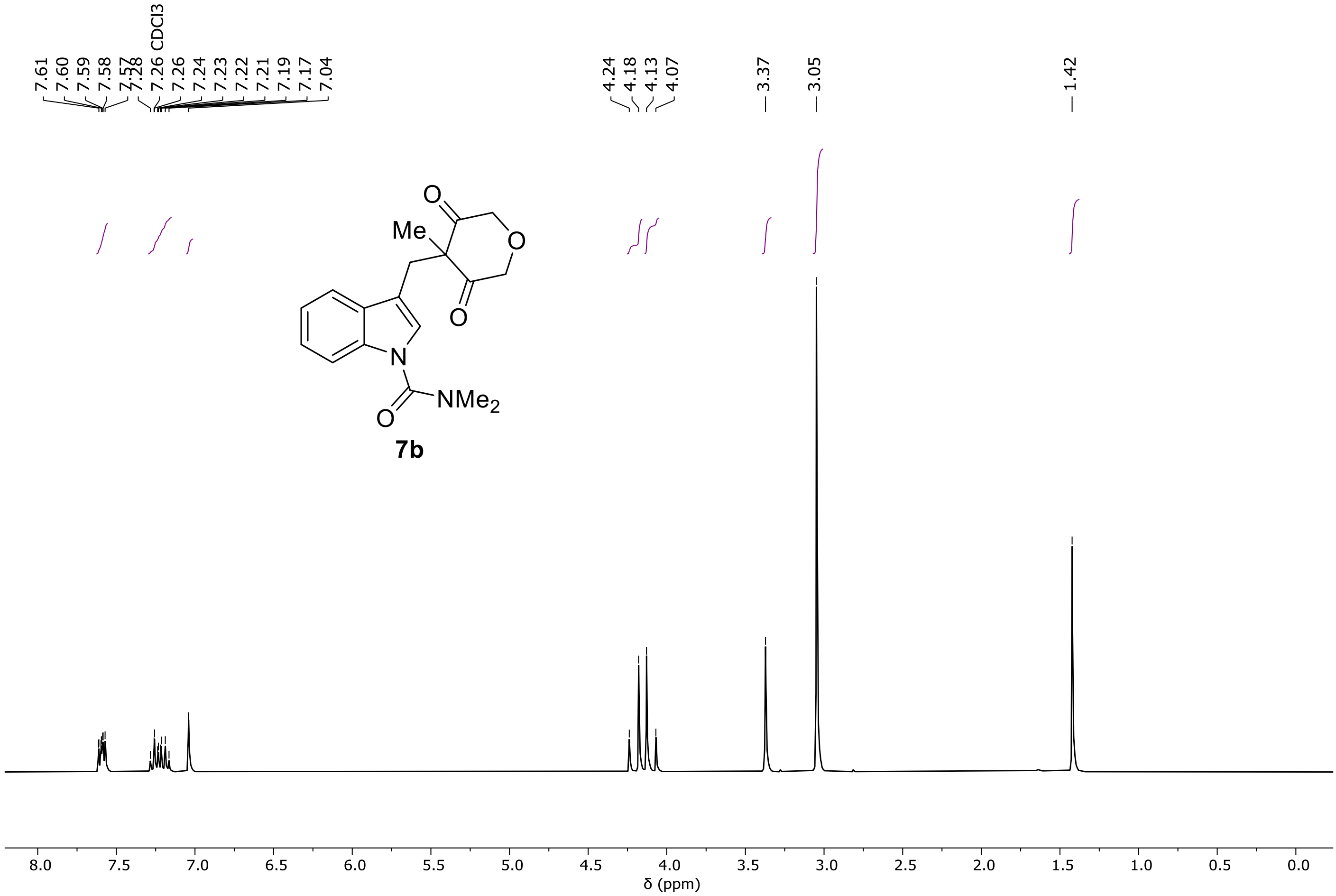

DEPT-135 NMR (75 MHz, CDCl3)

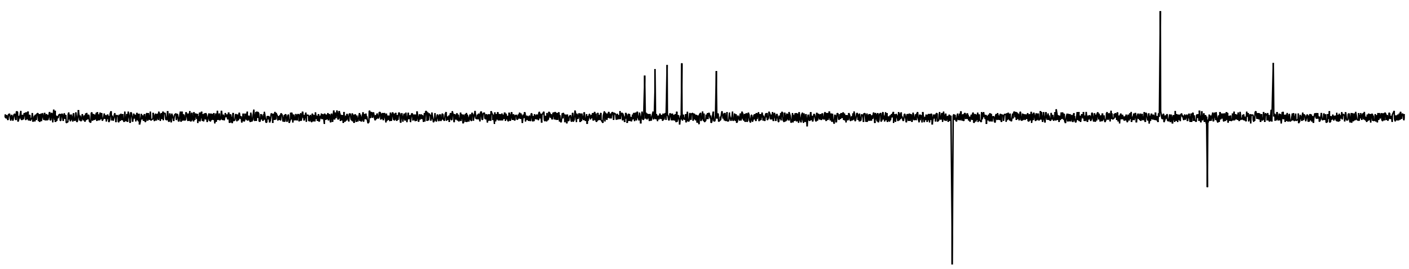

13C NMR (75 MHz, CDCl3)

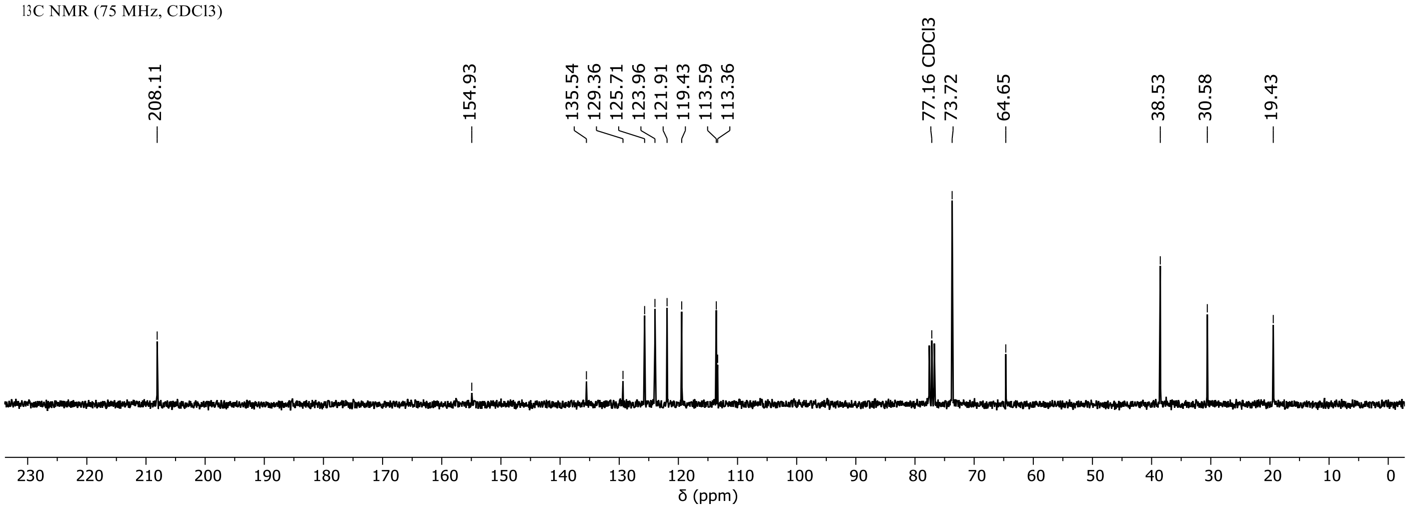

1H NMR (300 MHz, CDCl3)

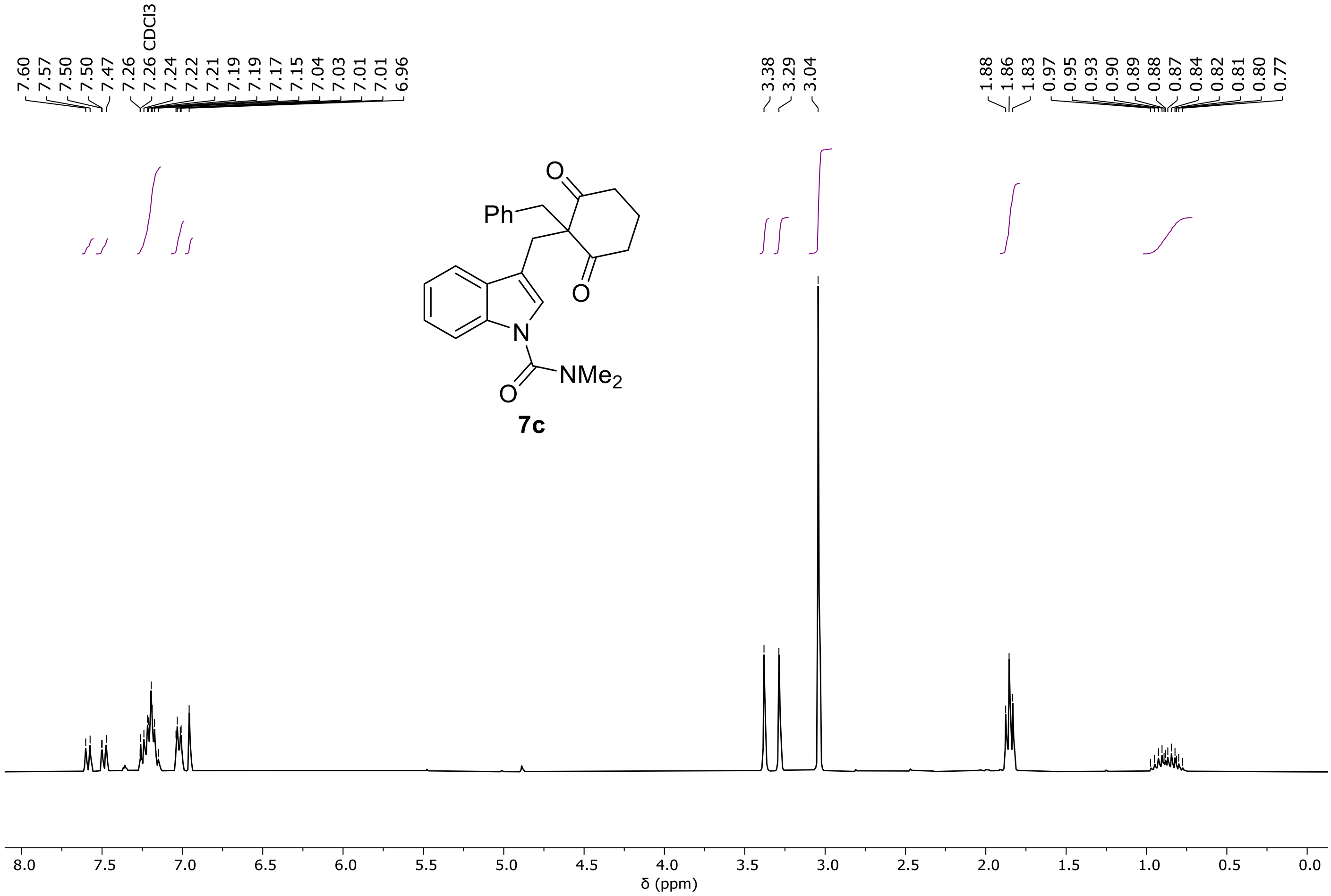

DEPT-135 NMR (75 MHz, CDCl3)

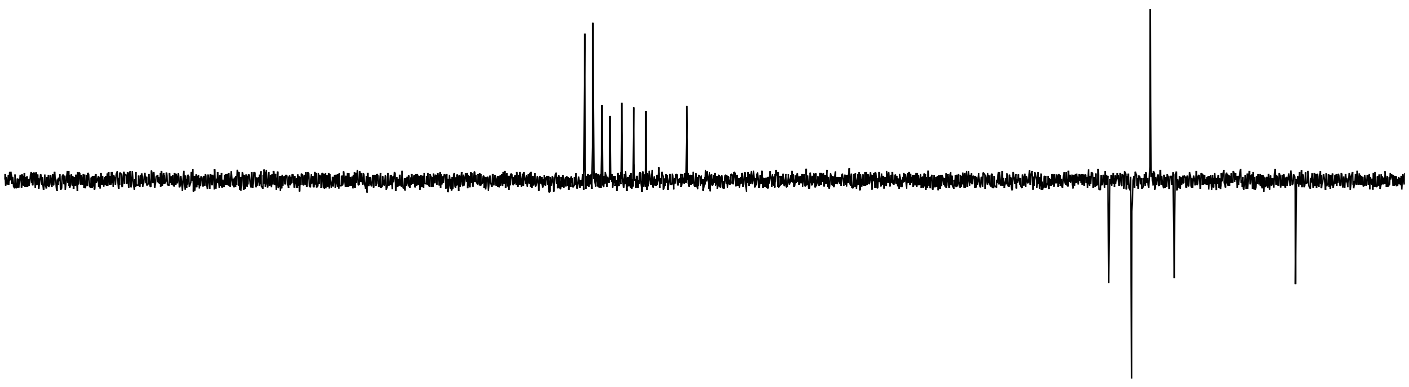

13C NMR (75 MHz, CDCl3)

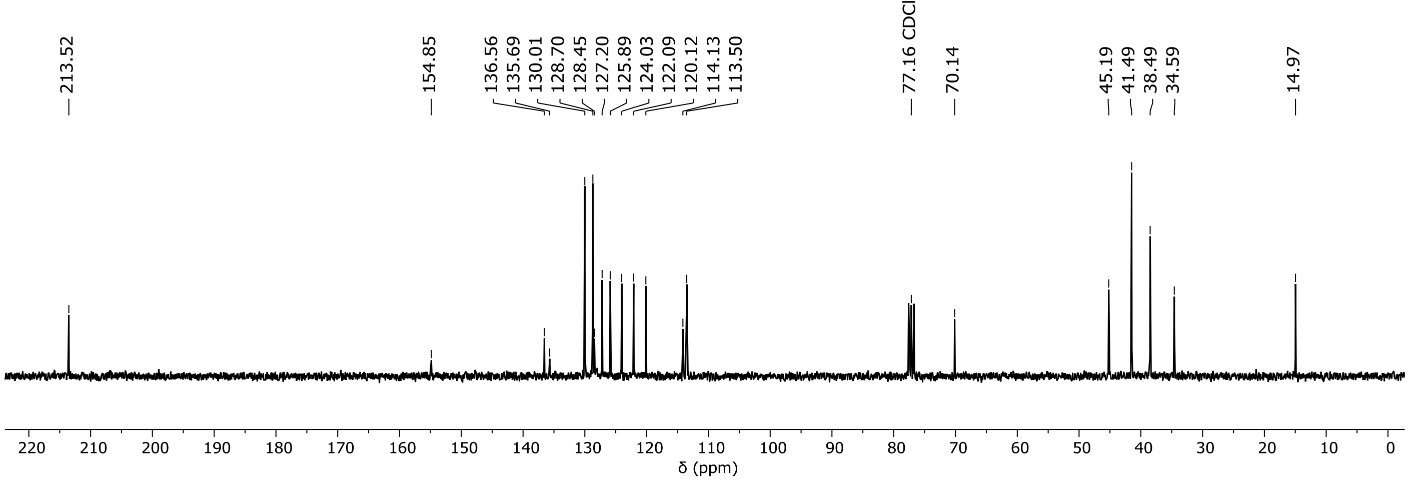

1H NMR (300 MHz, CDCl3)

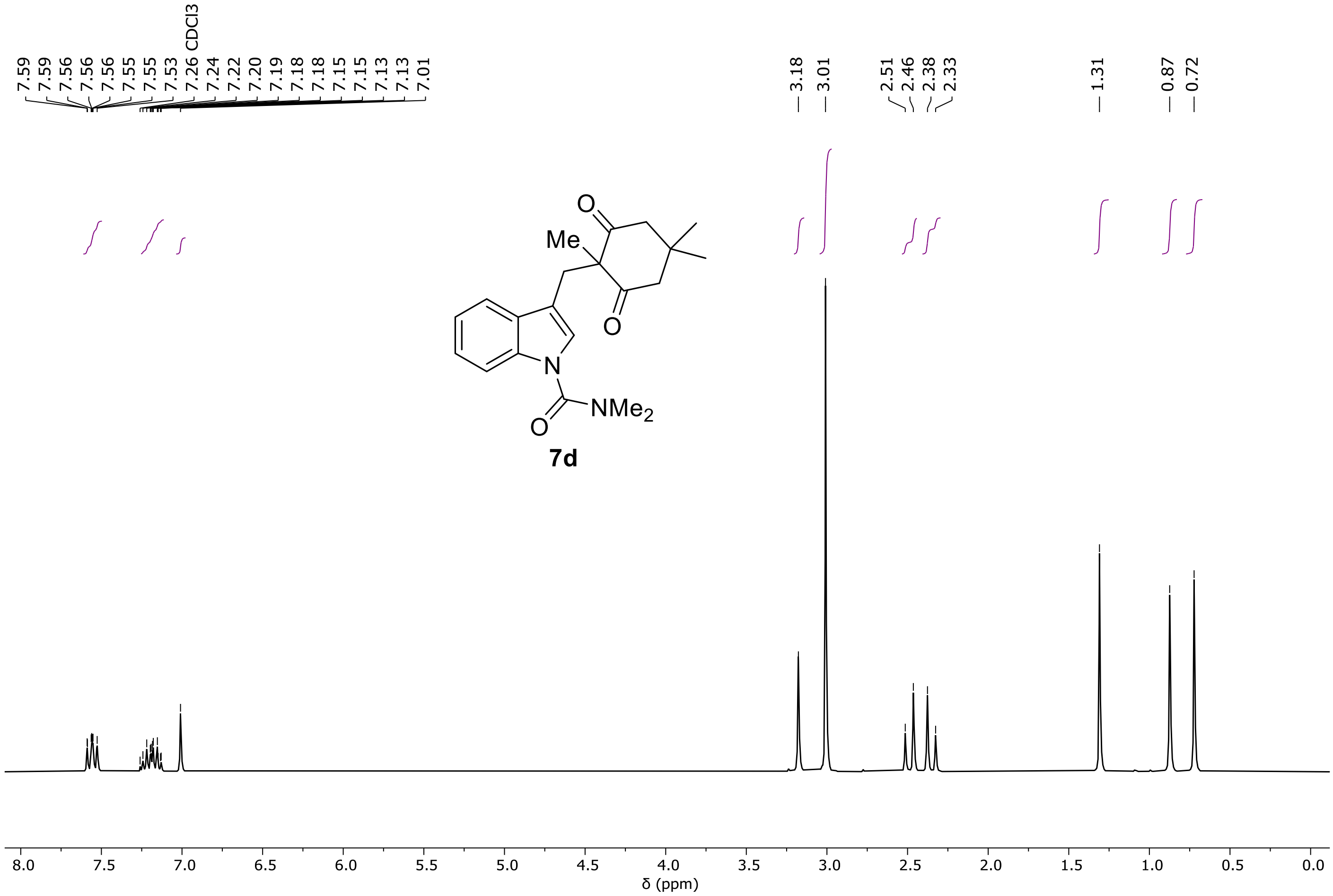

DEPT-135 NMR (75 MHz, CDCl3)

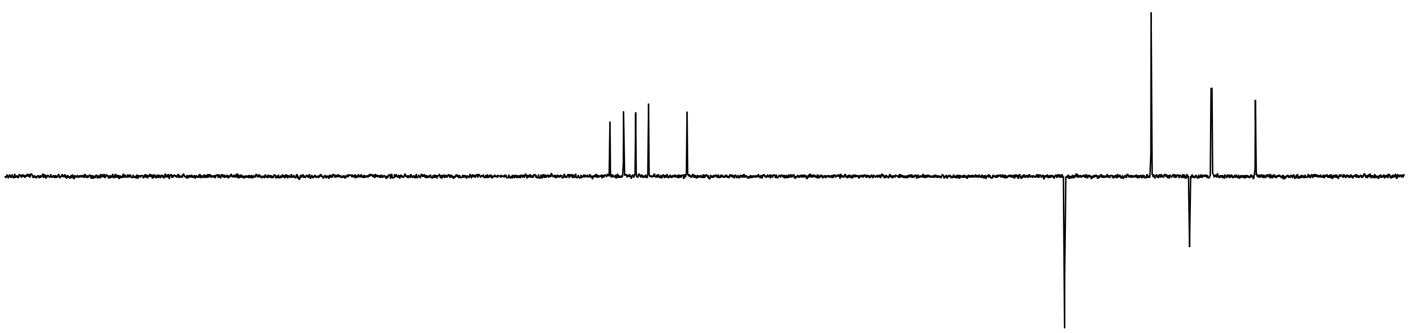

13C NMR (75 MHz, CDCl3)

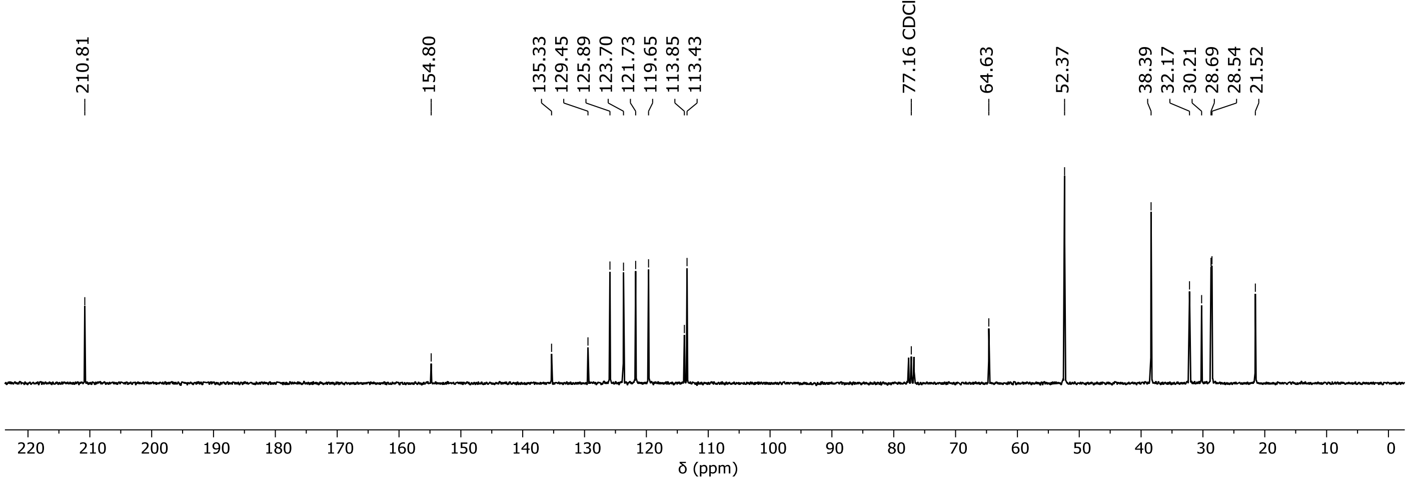

1H NMR (300 MHz, CDCl3)

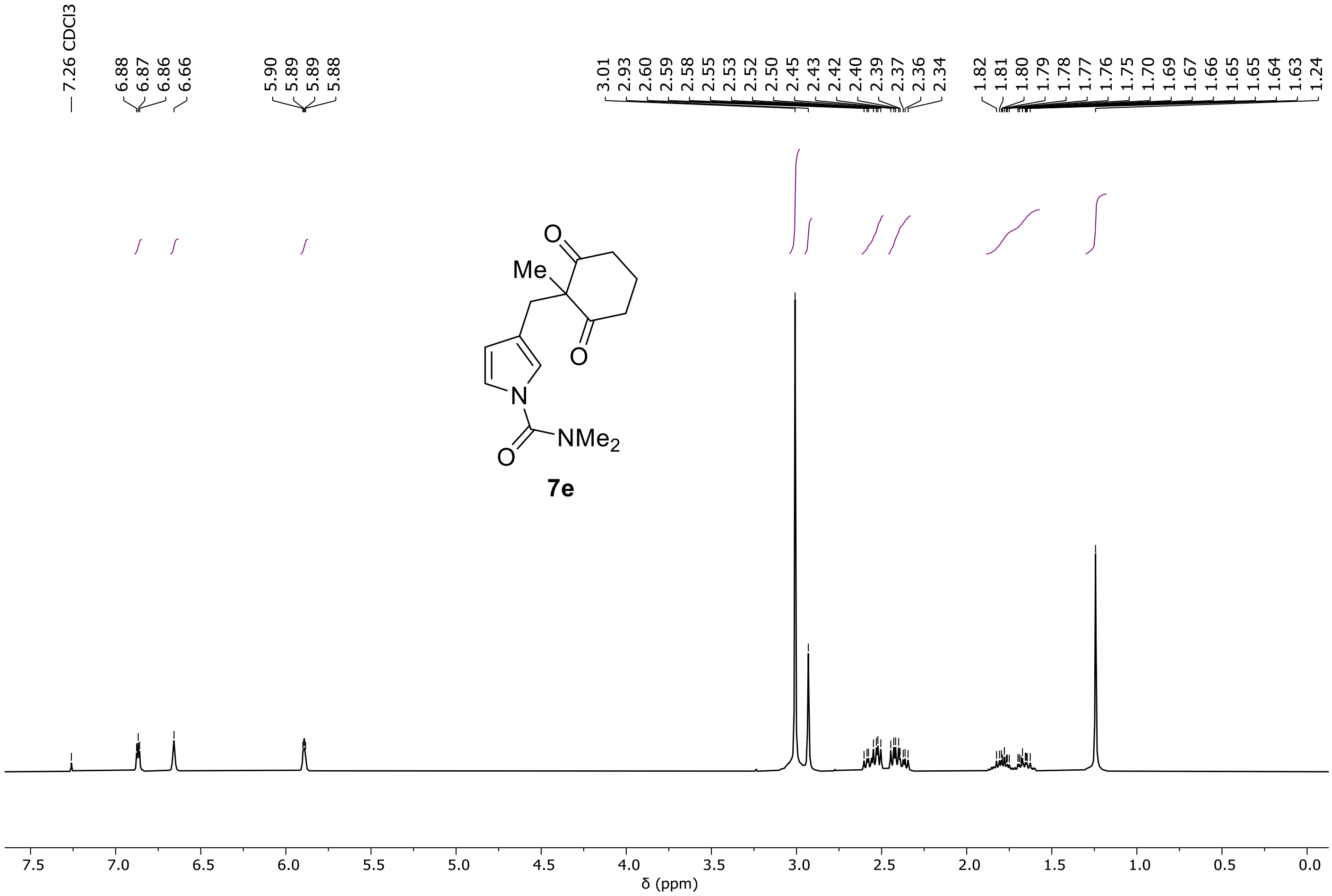

DEPT-135 NMR (75 MHz, CDCl3)

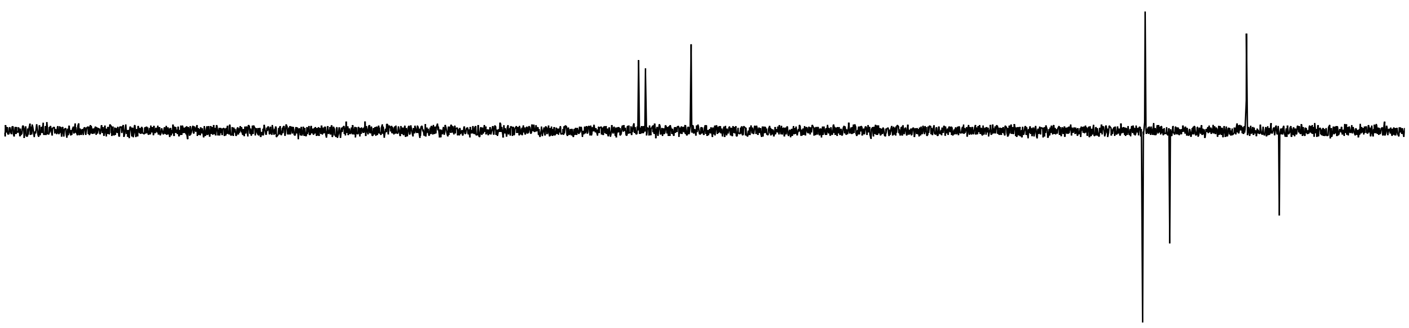

13C NMR (75 MHz, CDCl3)

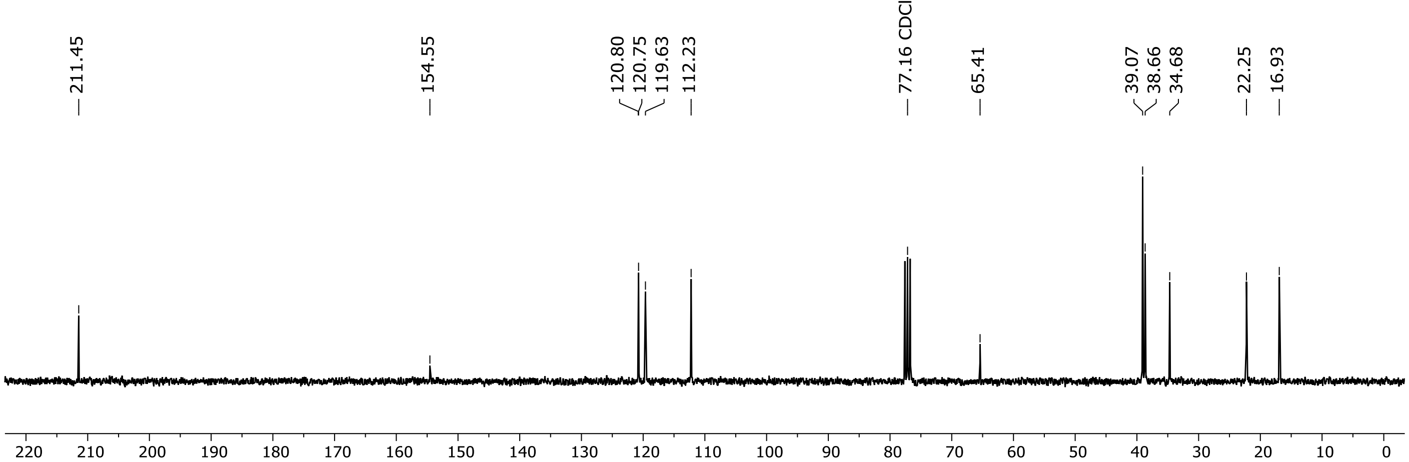

1H NMR (500 MHz, CDCl3)

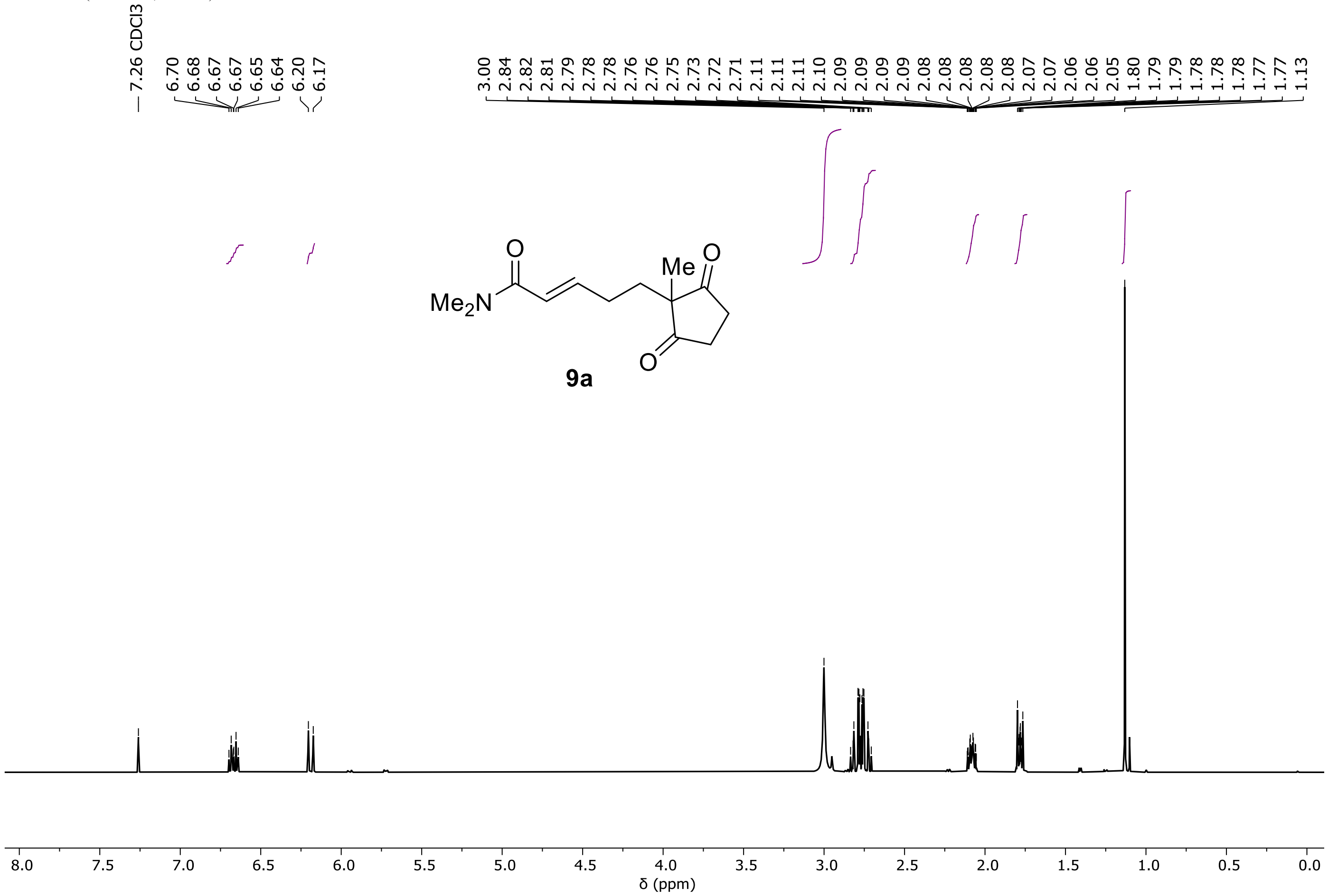

DEPT-135 NMR (126 MHz, CDCl3)

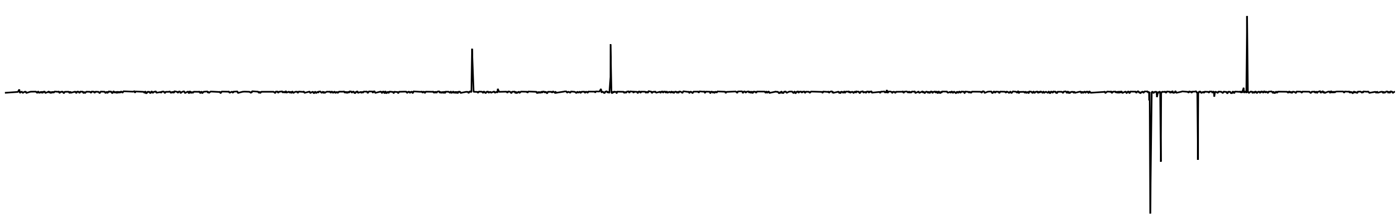

13C NMR (126 MHz, CDCl3)

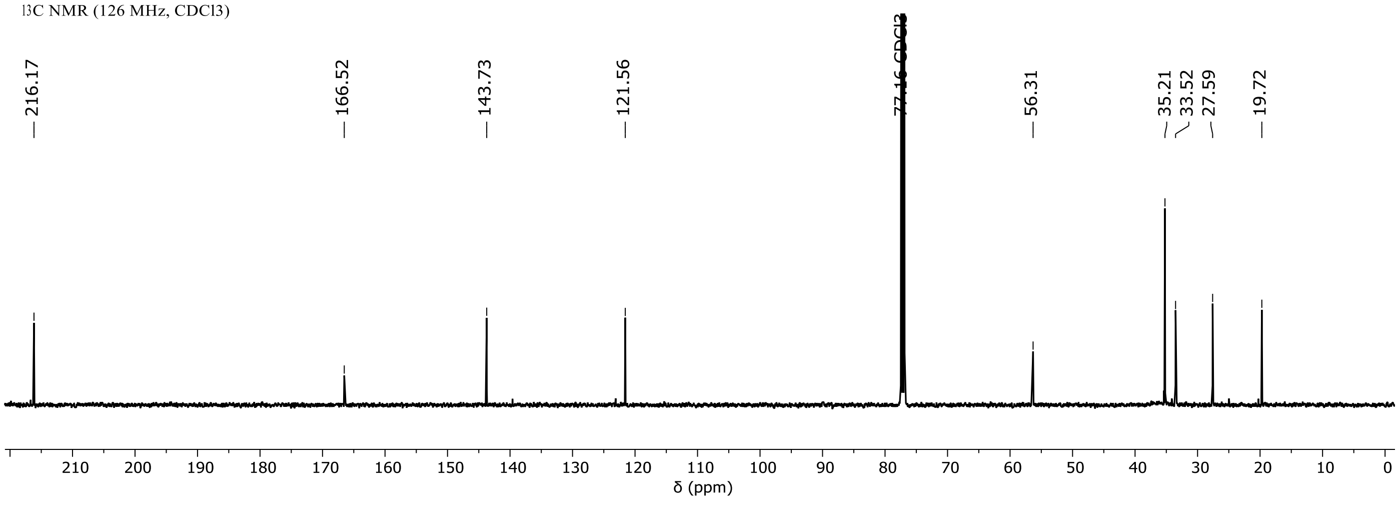

1H NMR (500 MHz, CDCl3, 0 ºC)

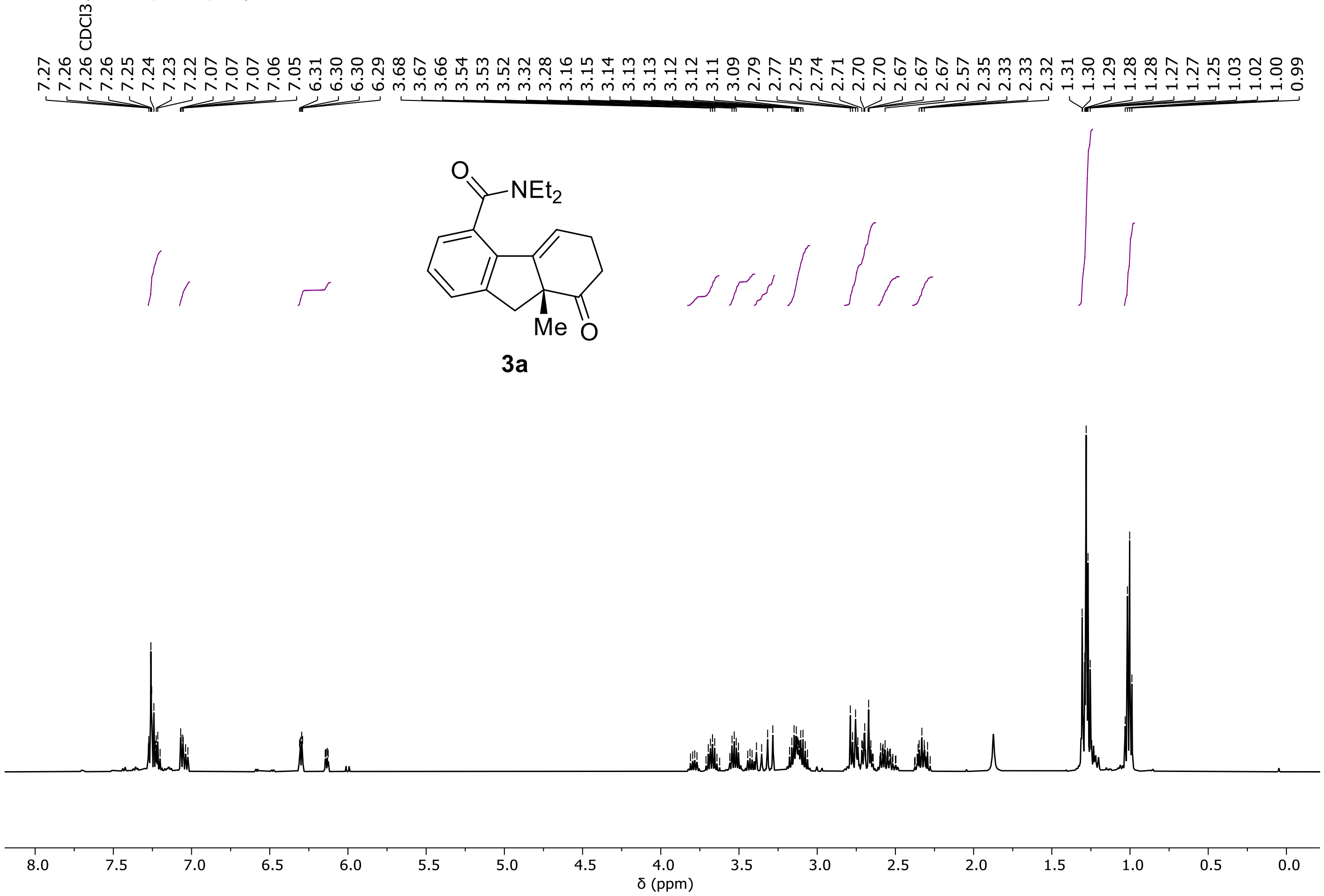

DEPT-135 NMR (126 MHz, CDCl3, 0 ºC)

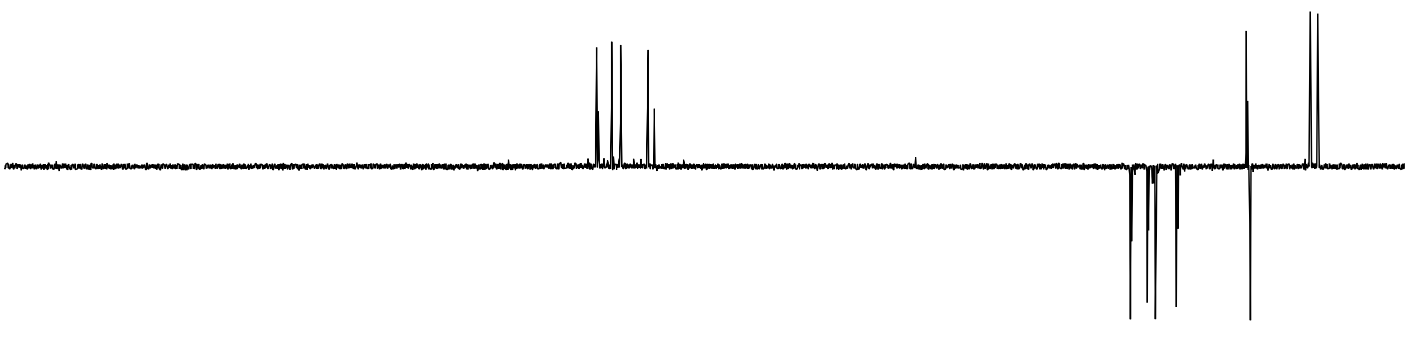

13C NMR (126 MHz, CDCl3, 0 ºC)

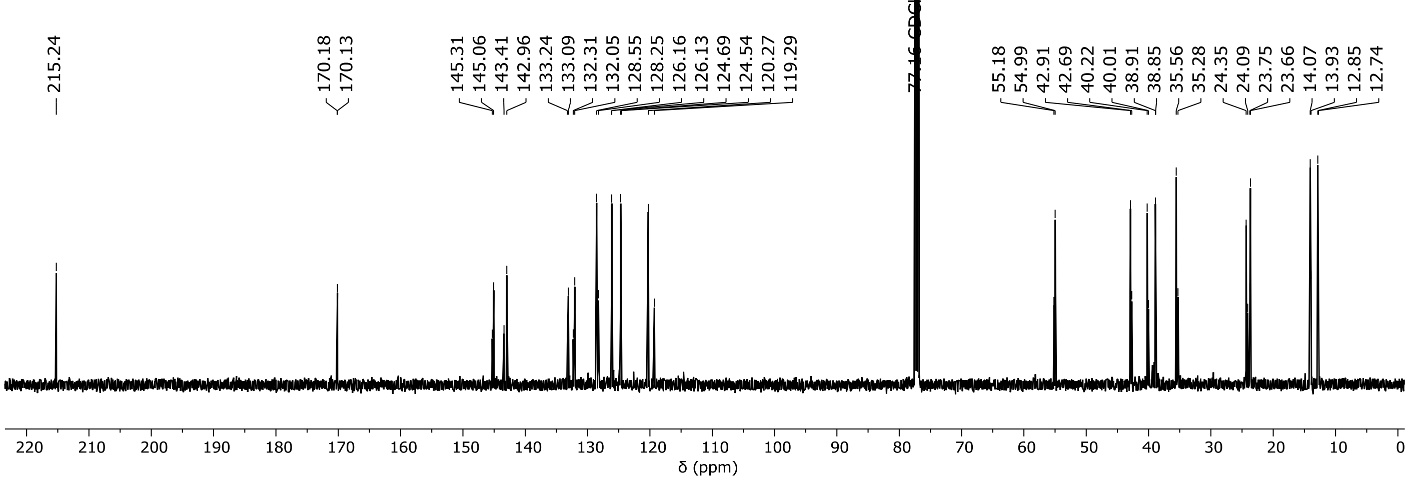

1H NMR (300 MHz, CDCl3)

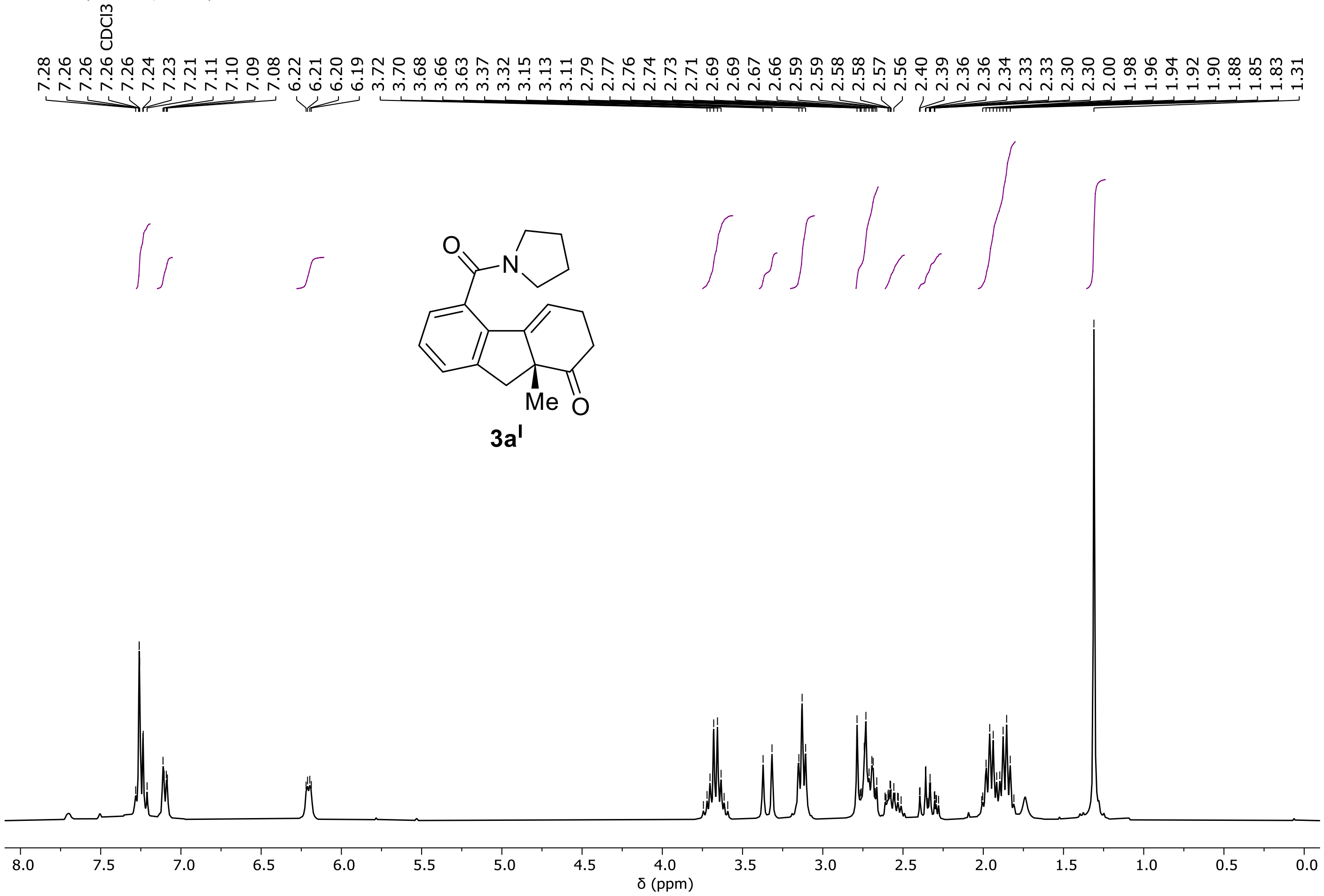

DEPT-135 NMR (75 MHz, CDCl3)

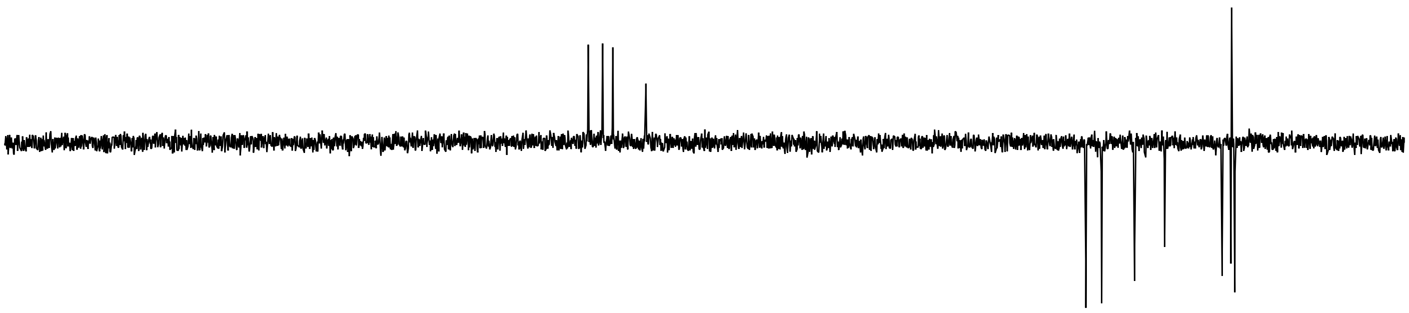

13C NMR (75 MHz, CDCl3)

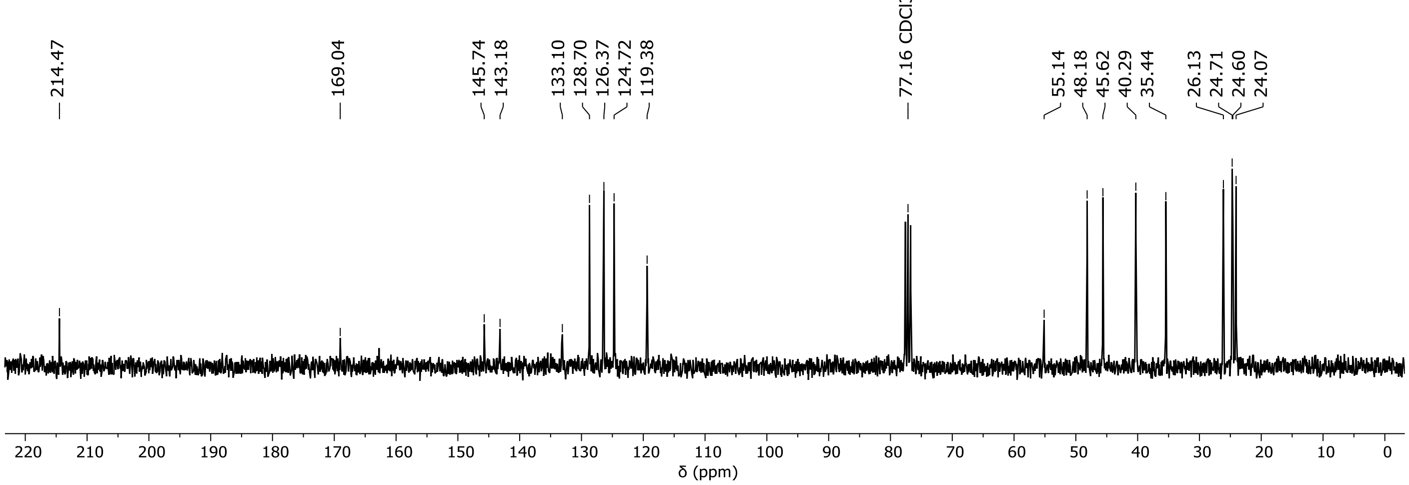

1H NMR (500 MHz, CDCl3)

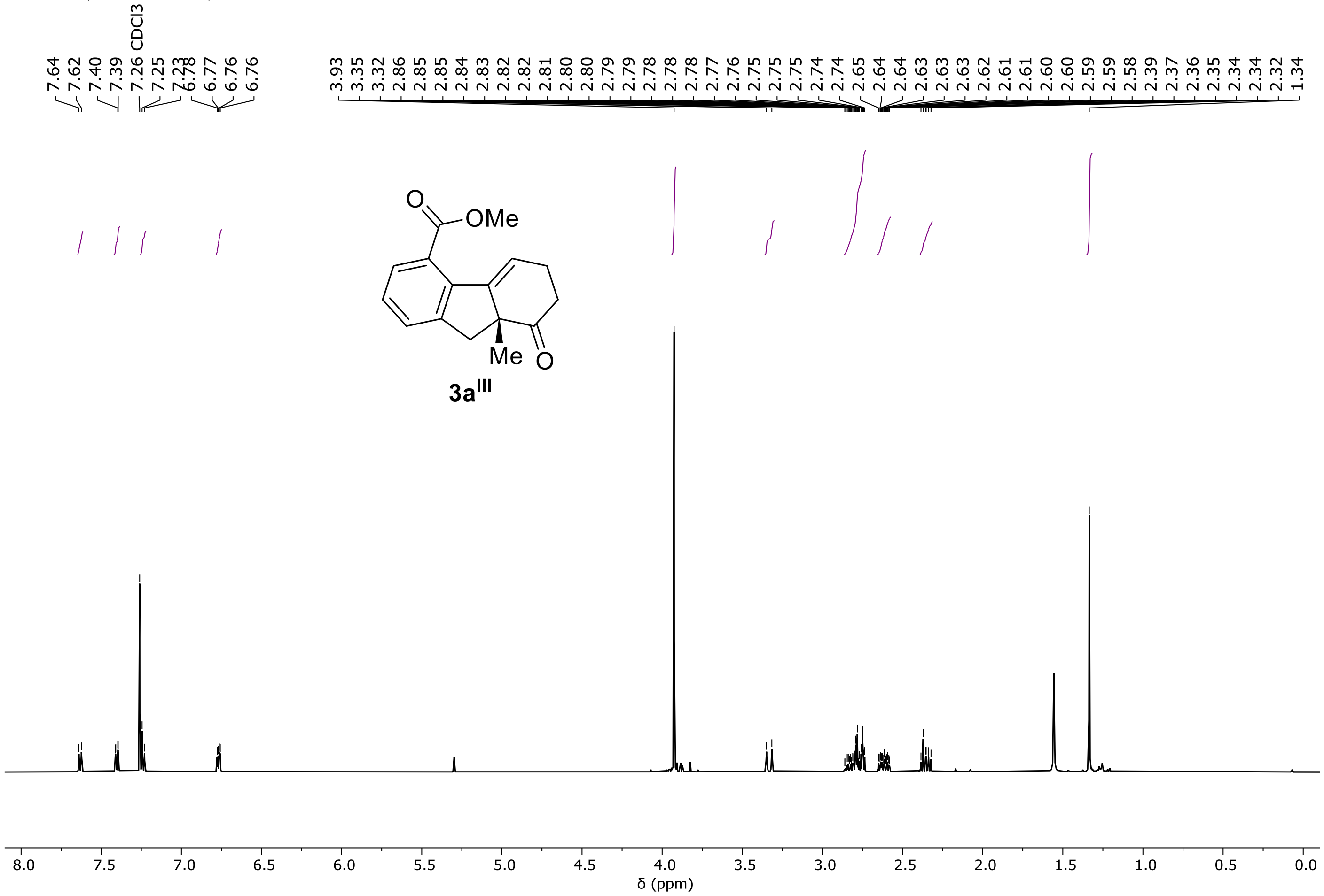

DEPT-135 NMR (126 MHz, CDCl3)

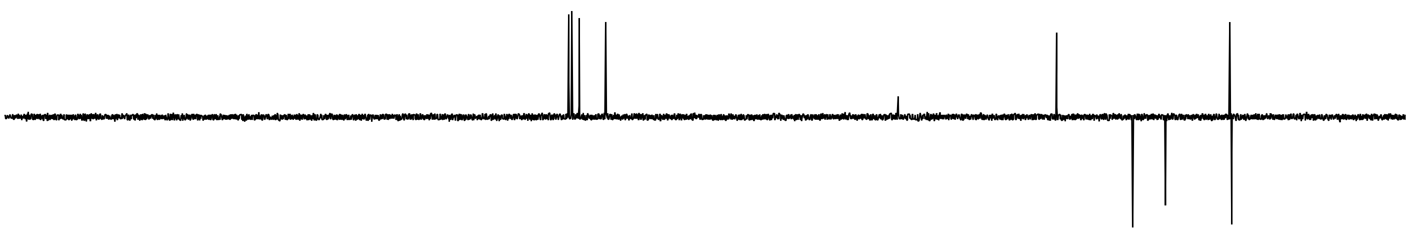

13C NMR (126 MHz, CDCl3)

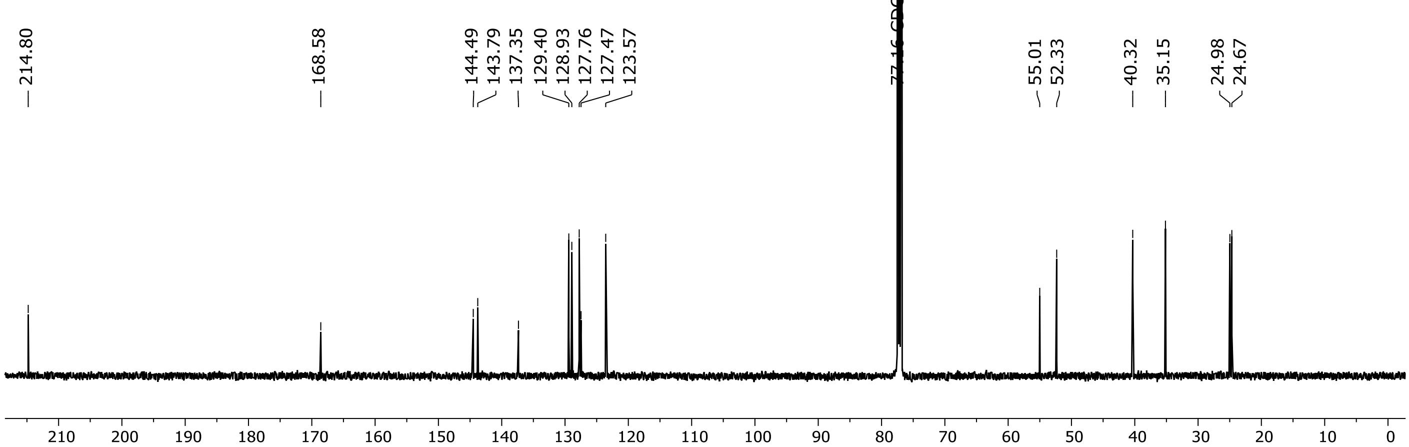

1H NMR (500 MHz, CDCl3)

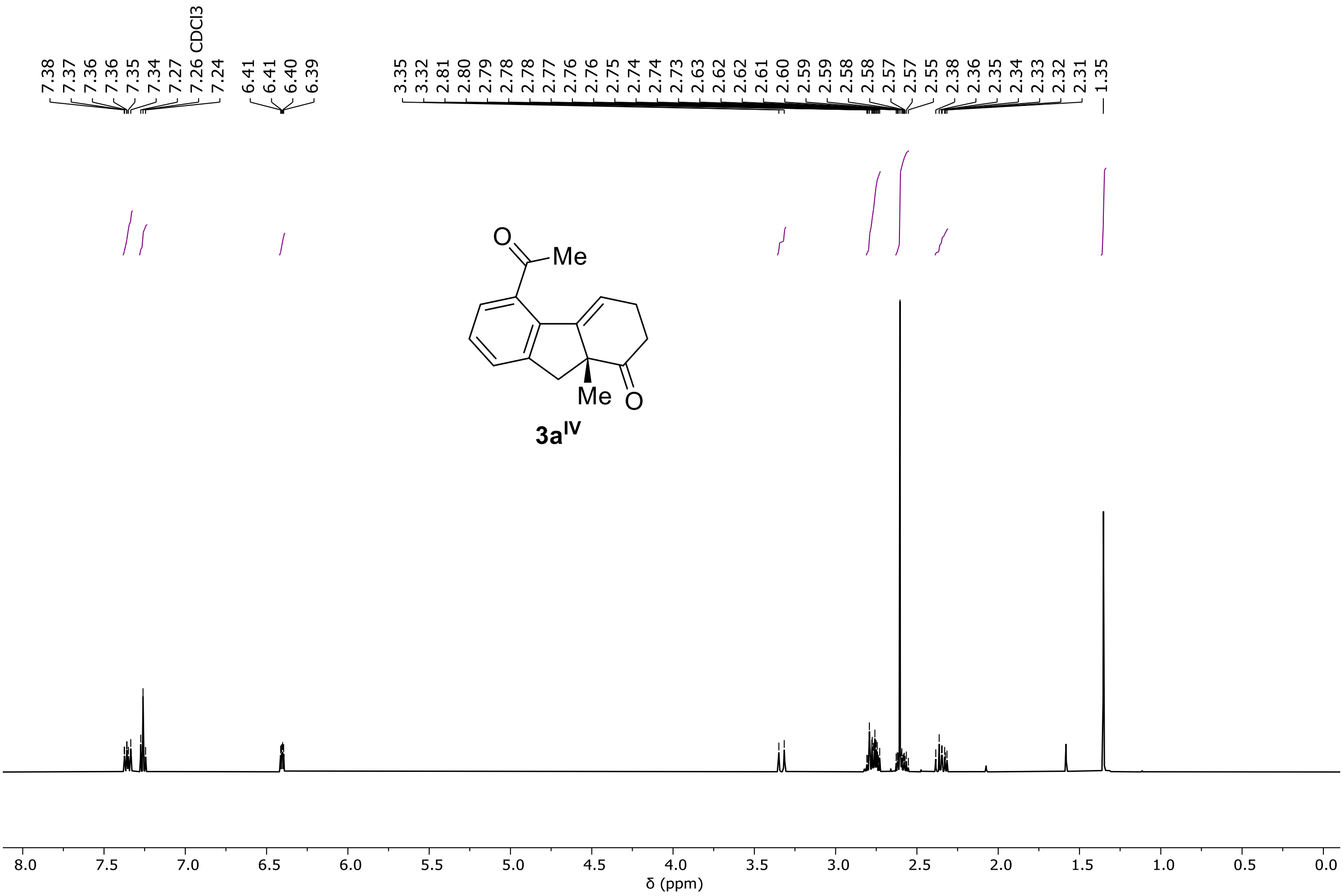

DEPT-135 NMR (126 MHz, CDCl3)

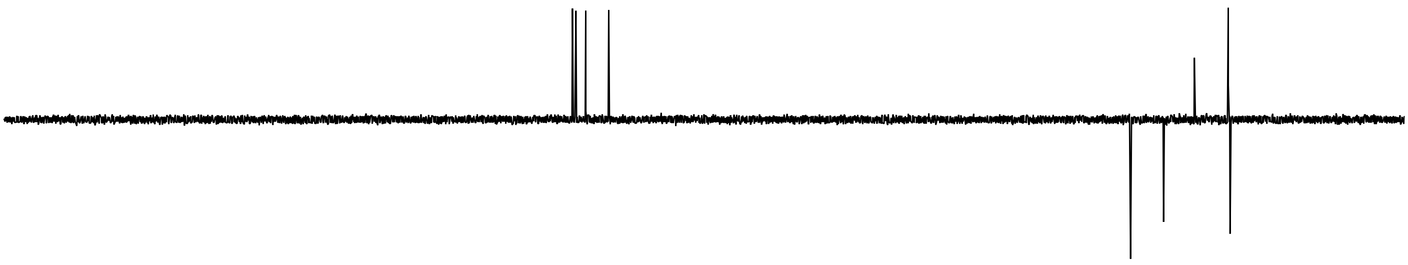

13C NMR (126 MHz, CDCl3)

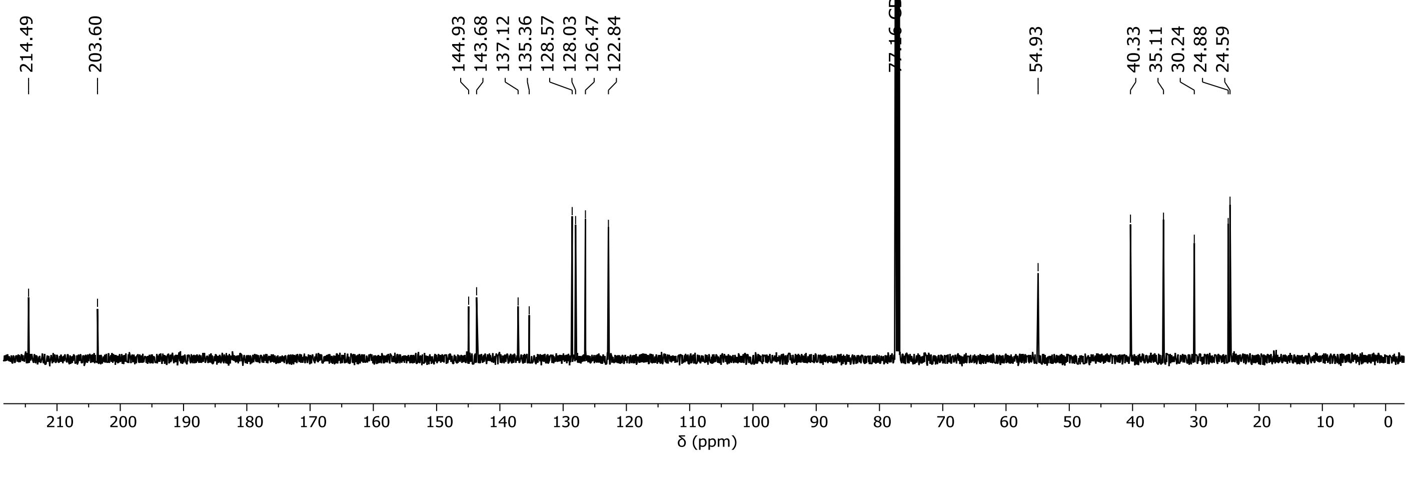

1H NMR (500 MHz, CDCl3)

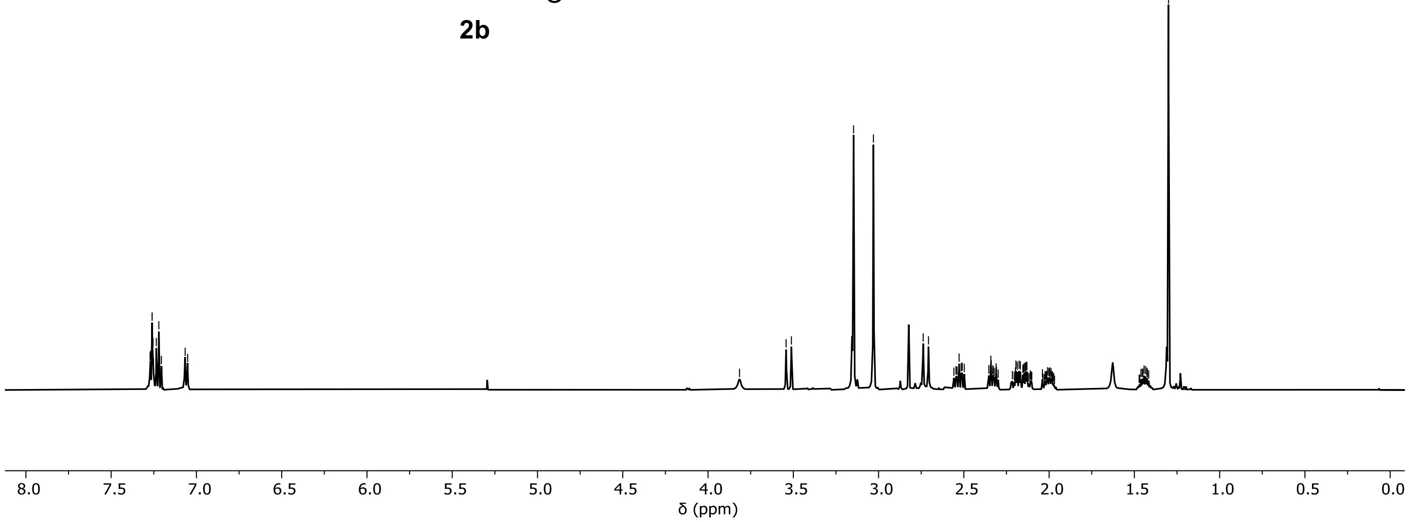

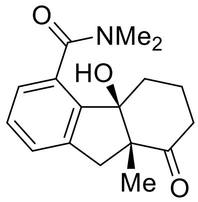

2b

DEPT-135 NMR (126 MHz, CDCl3)

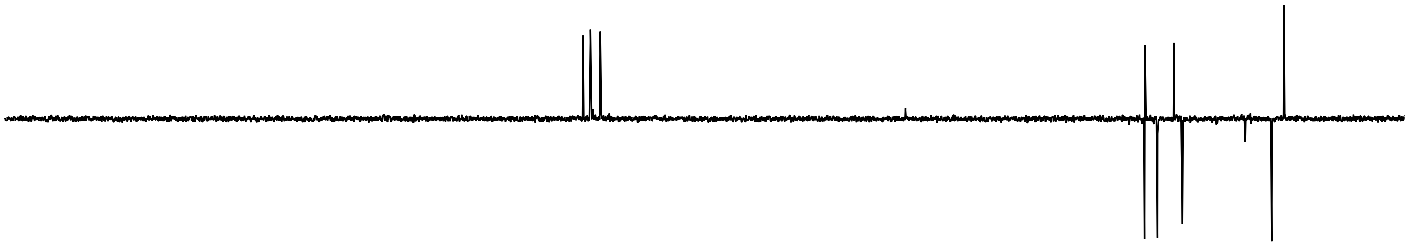

13C NMR (126 MHz, CDCl3)

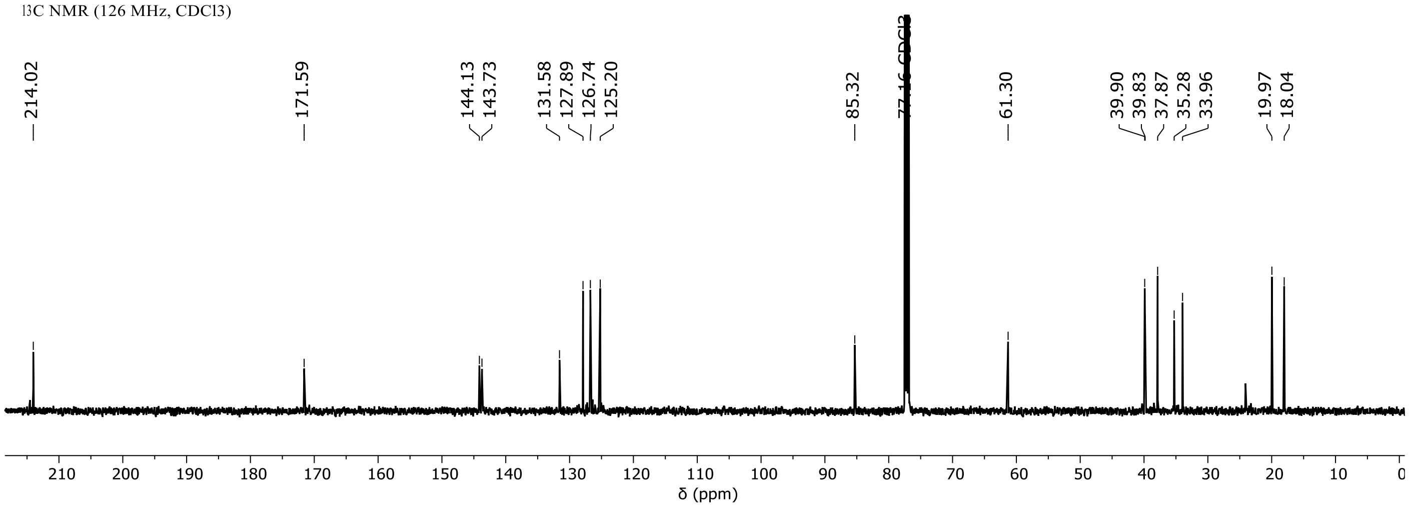

1H NMR (500 MHz, CDCl3)

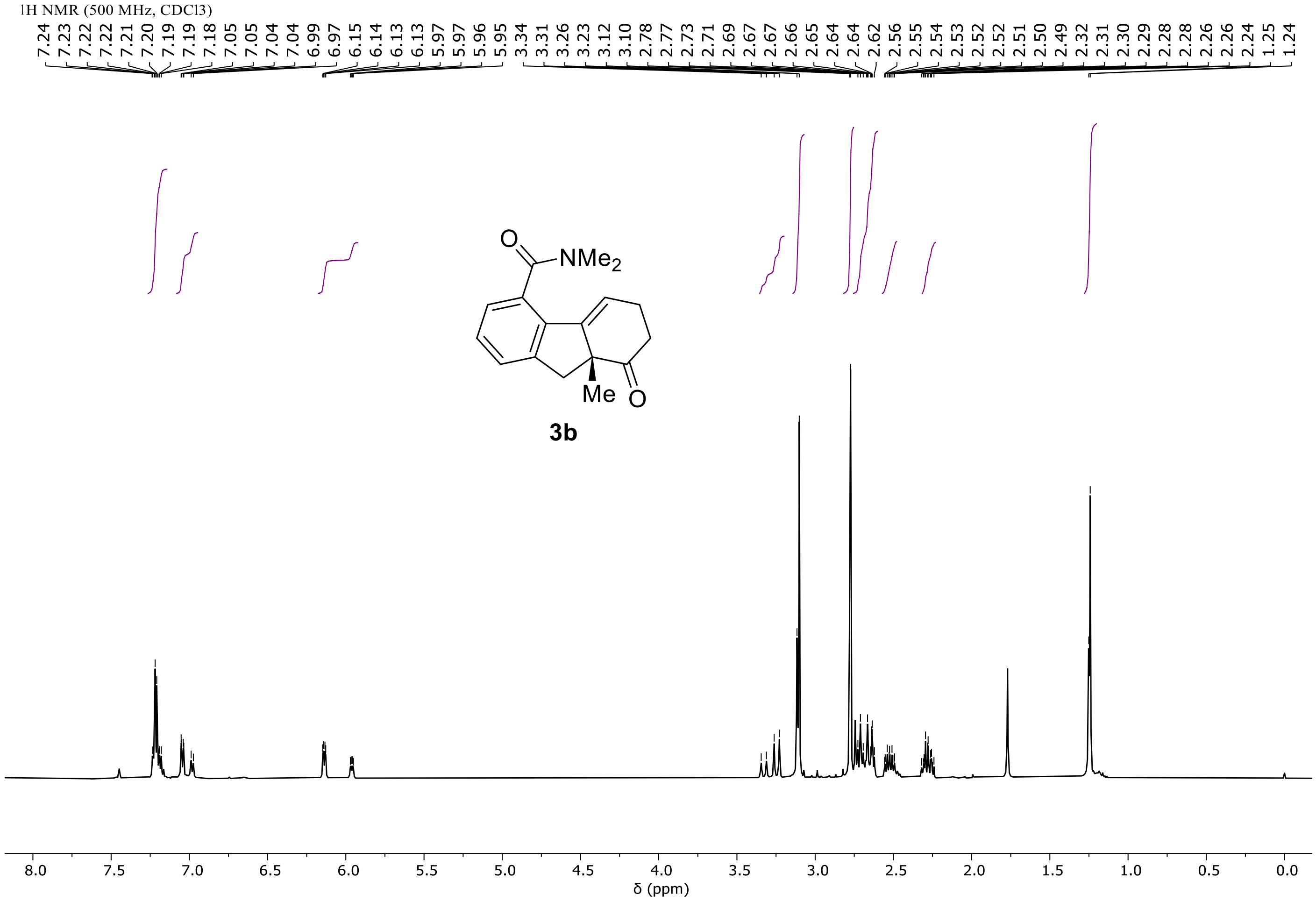

DEPT-135 NMR (126 MHz, CDCl3)

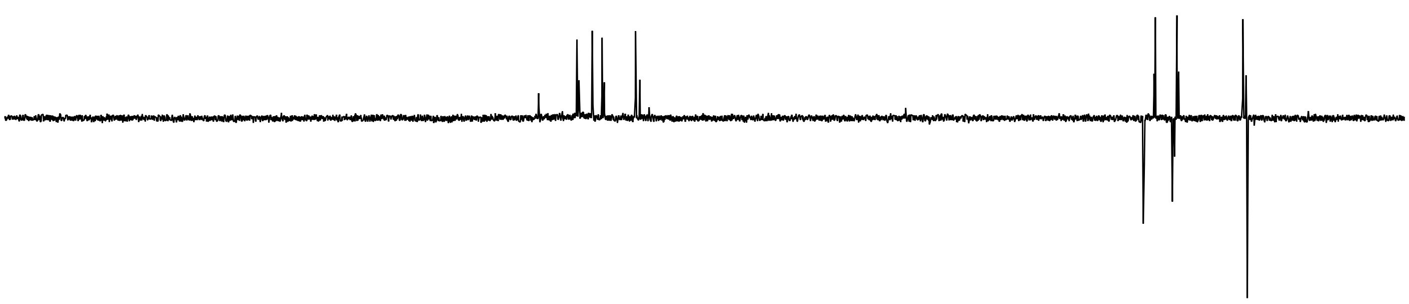

13C NMR (126 MHz, CDCl3)

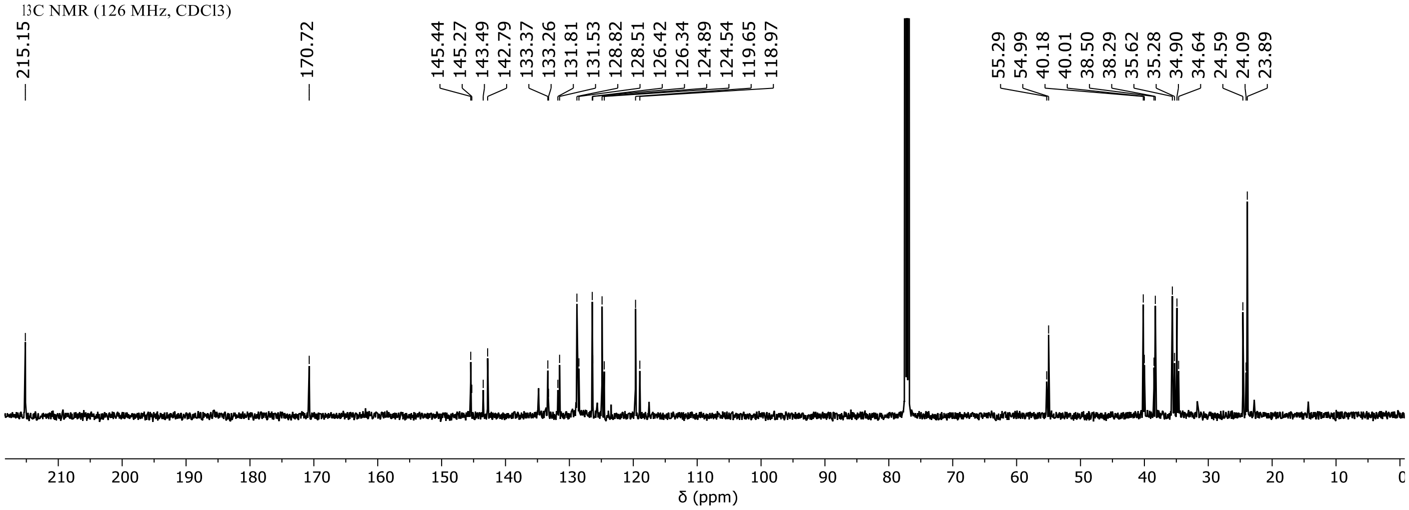

1H NMR (500 MHz, CDCl3)

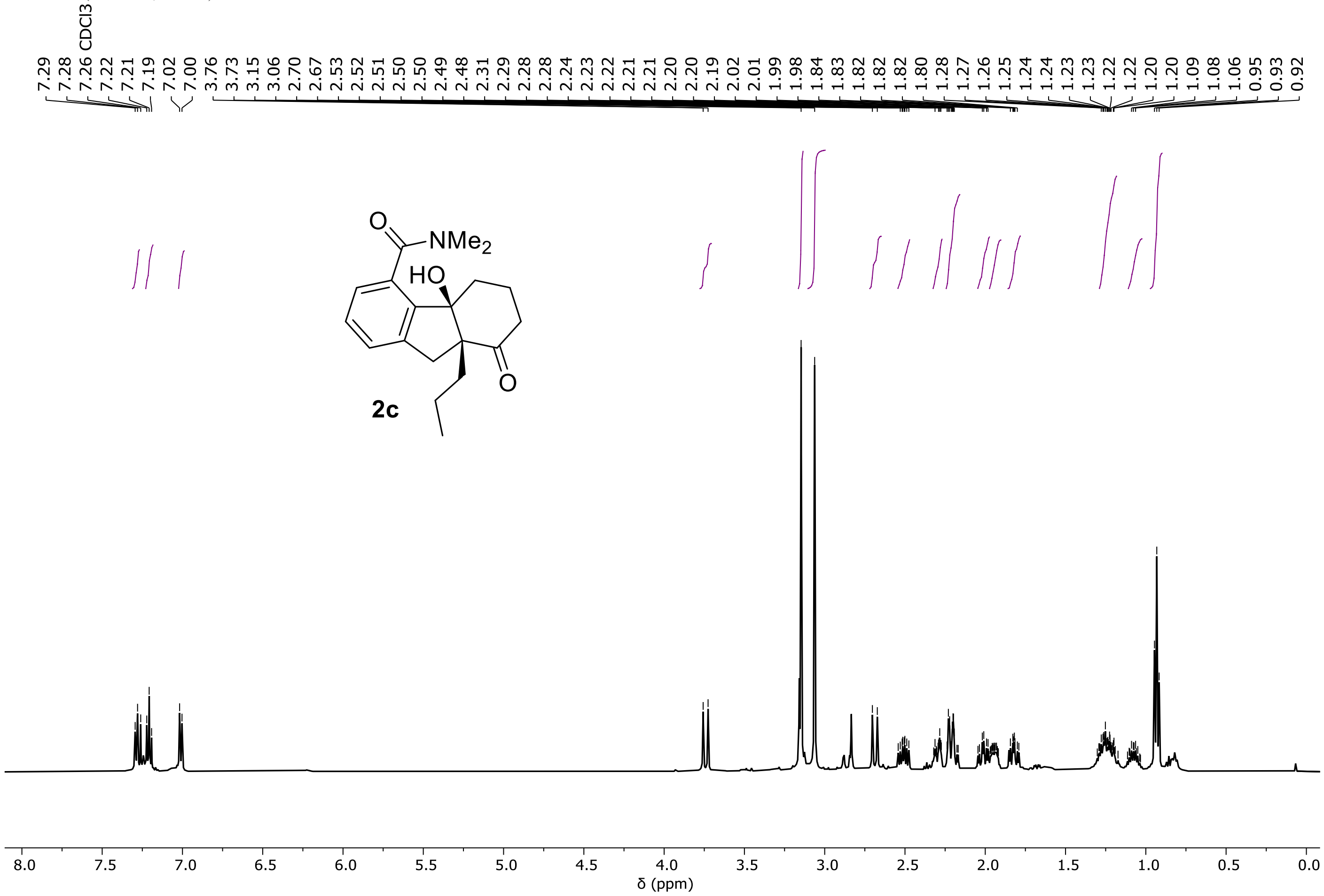

DEPT-135 NMR (126 MHz, CDCl3)

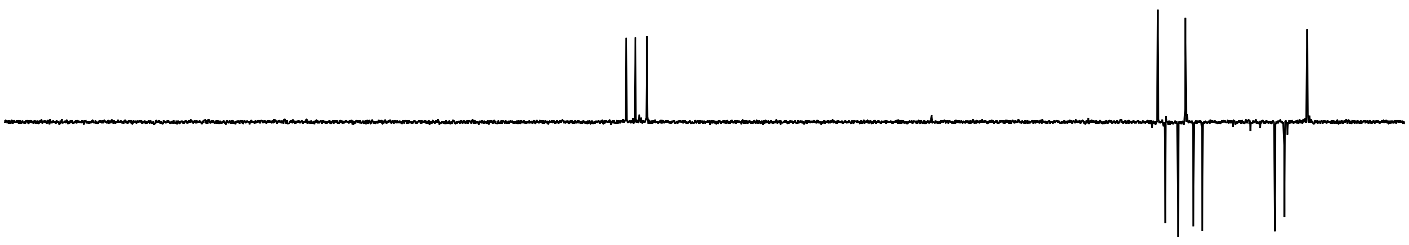

13C NMR (126 MHz, CDCl3)

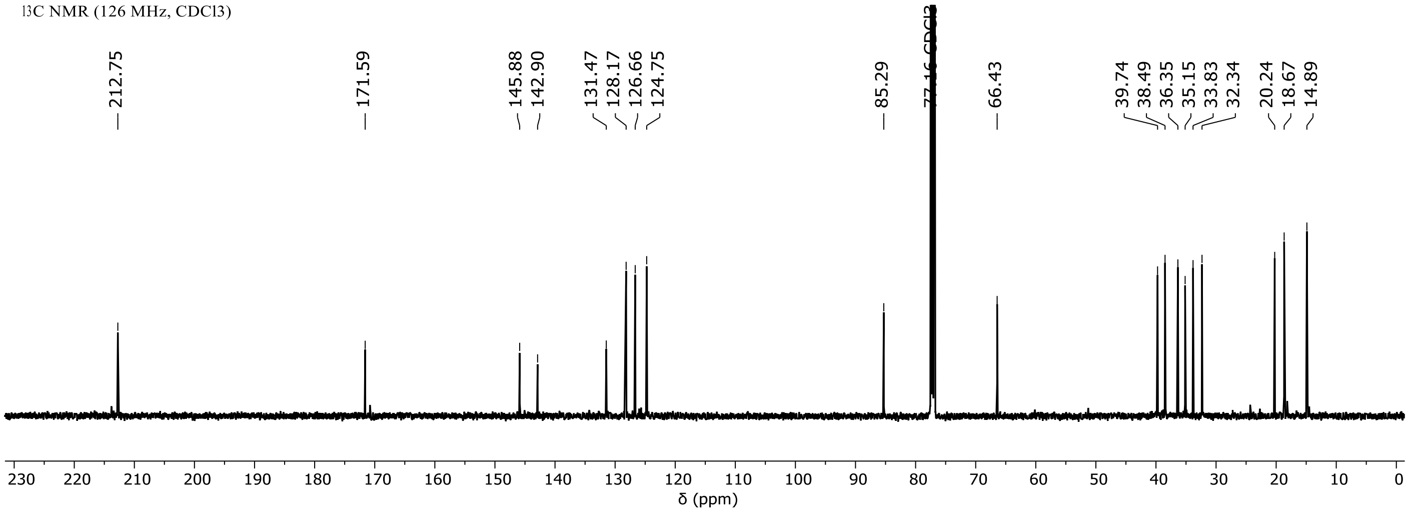

1H NMR (300 MHz, CDCl3)

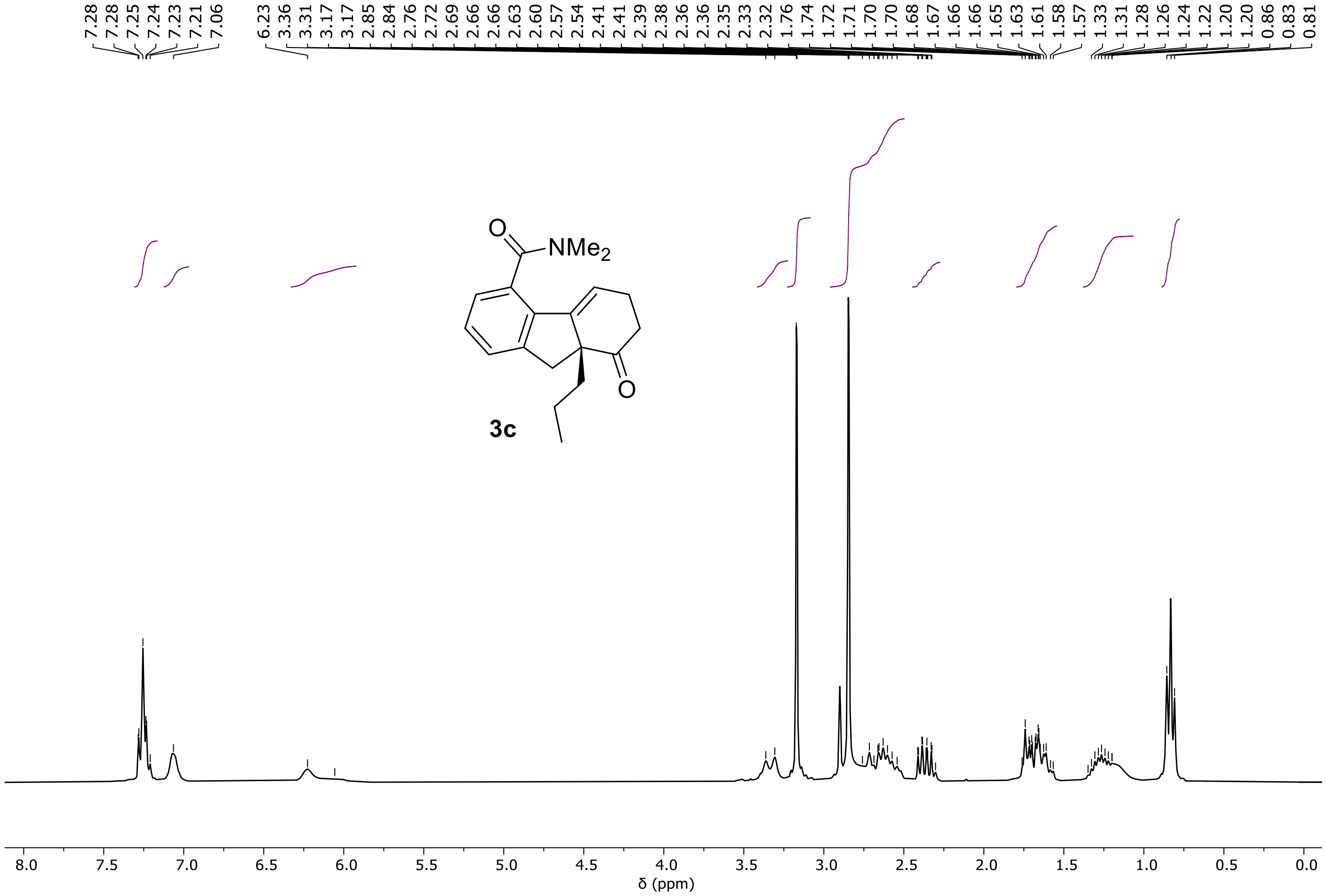

DEPT-135 NMR (75 MHz, CDCl3)

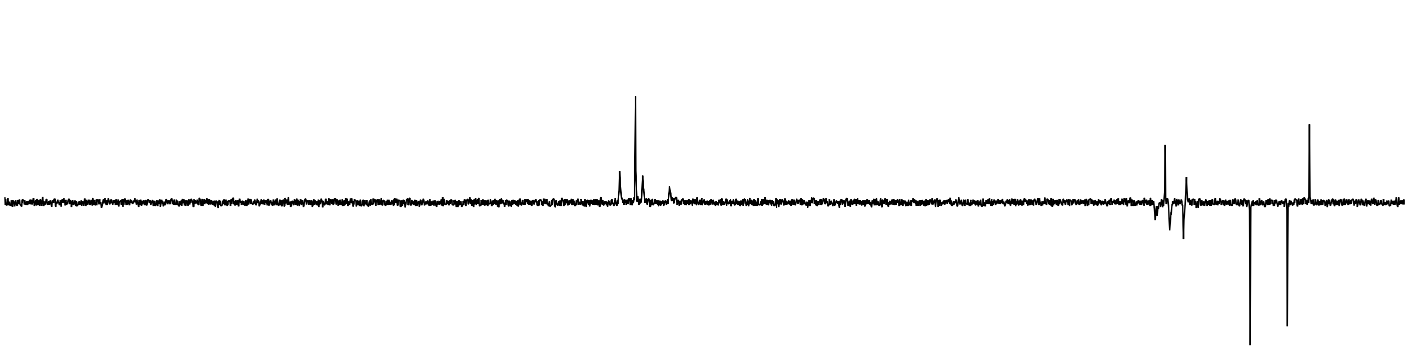

13C NMR (75 MHz, CDCl3)

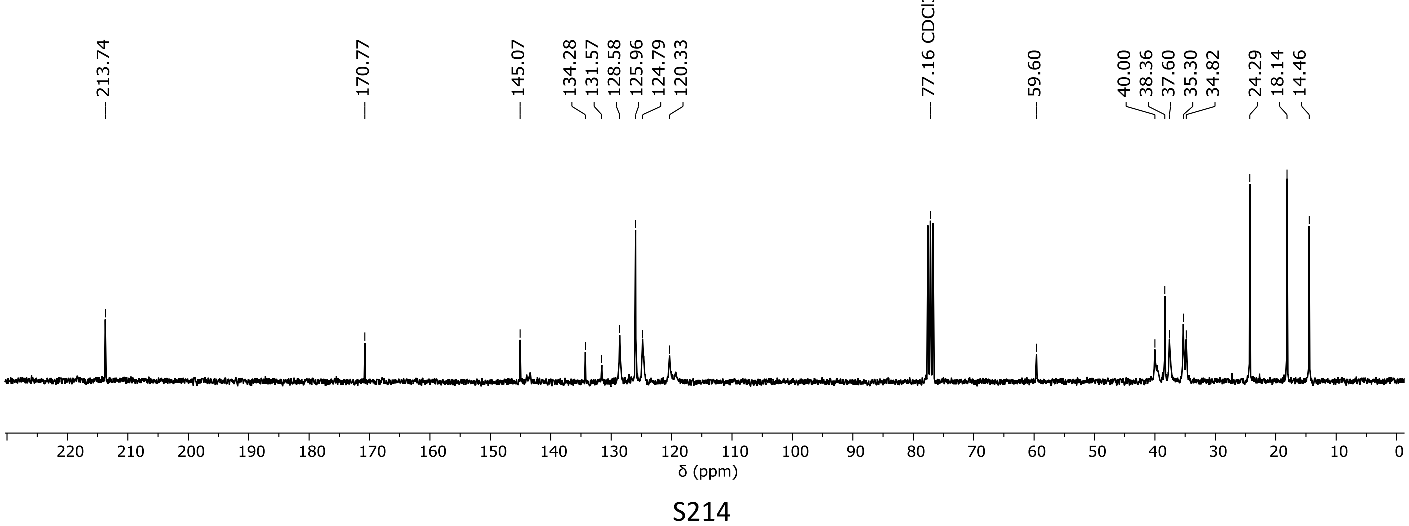

1H NMR (500 MHz, CDCl3, 55 ºC)

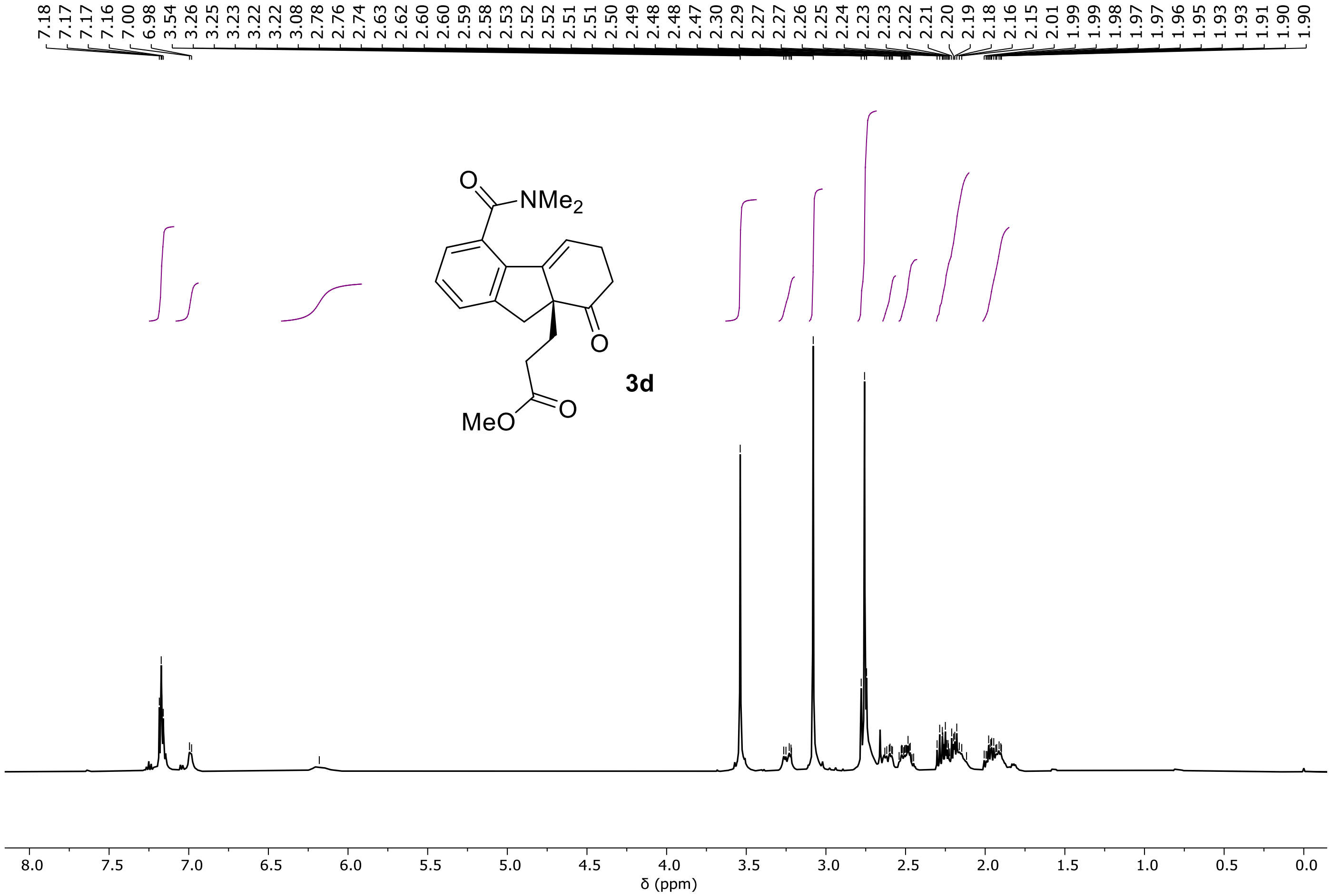

DEPT-135 NMR (126 MHz, CDCl3, 55 ºC)

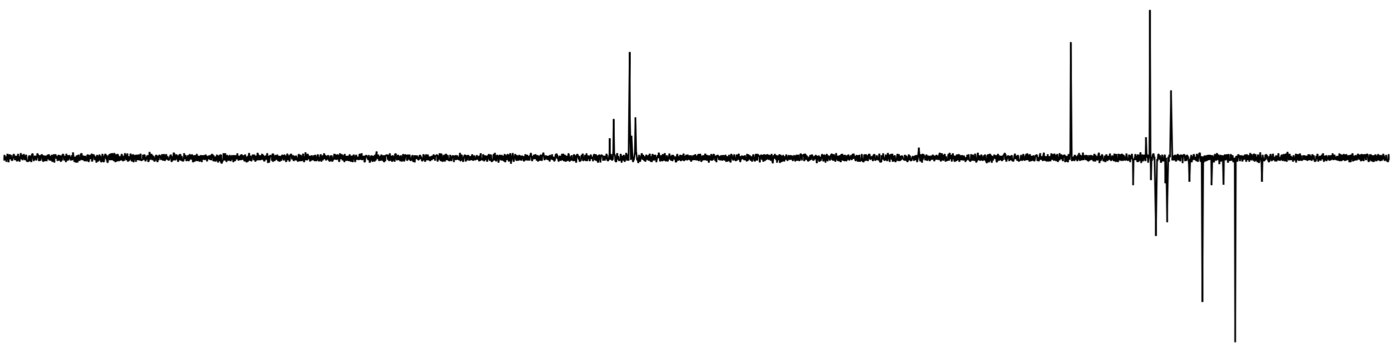

13C NMR (126 MHz, CDCl3, 55 ºC)

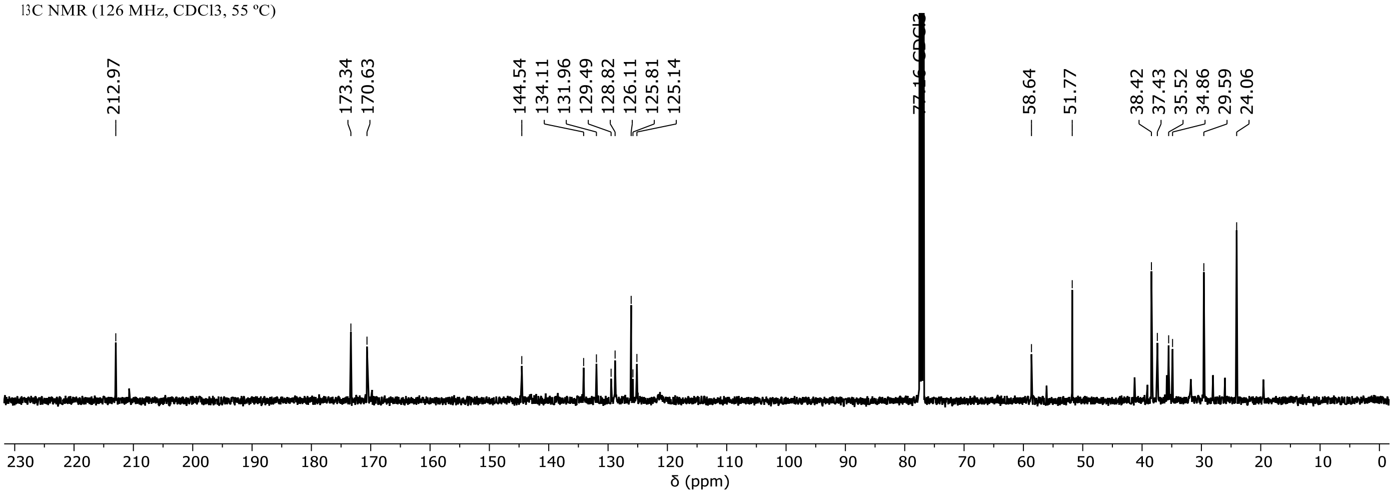

1H NMR (300 MHz, CDCl3)

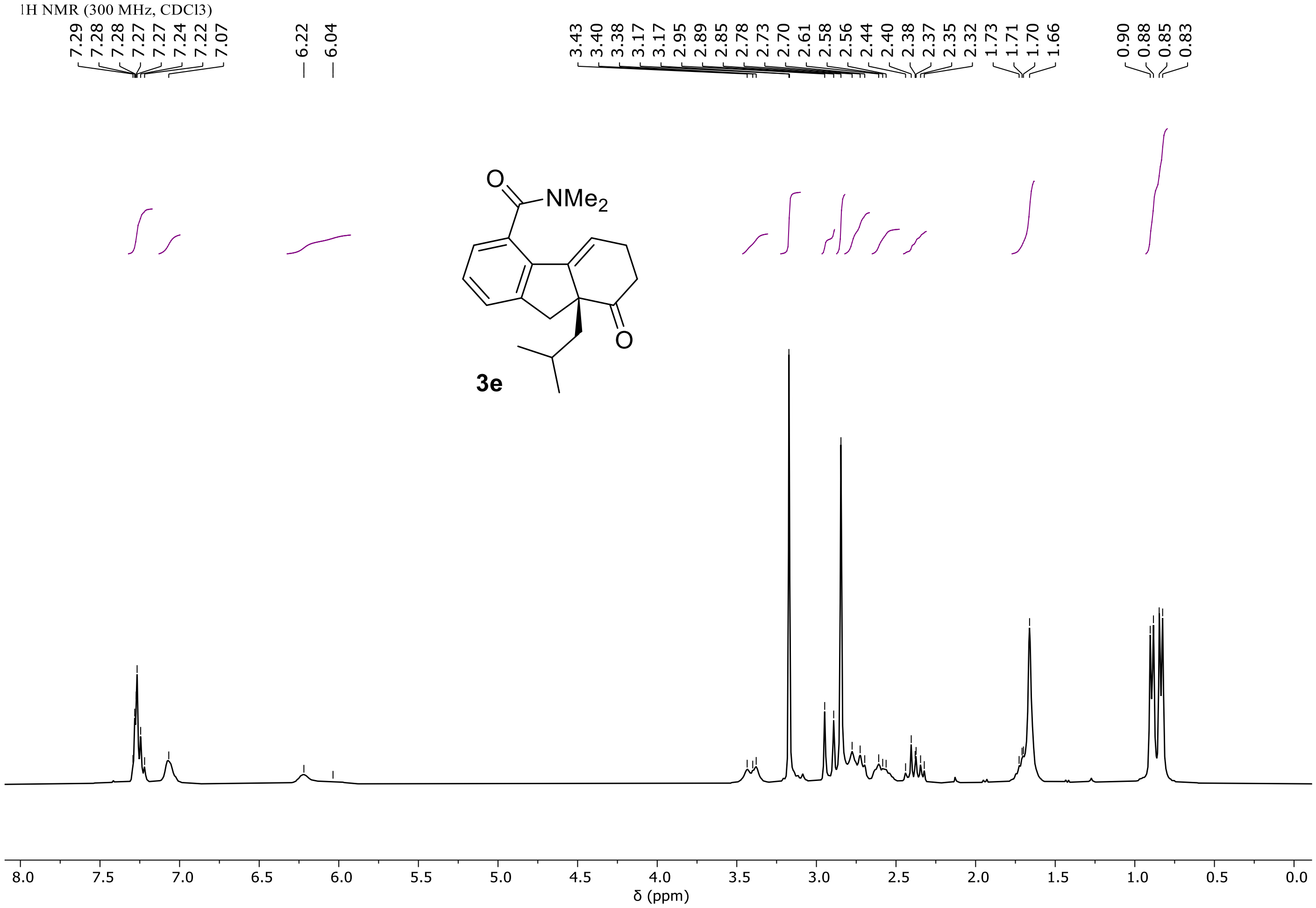

DEPT-135 NMR (75 MHz, CDCl3)

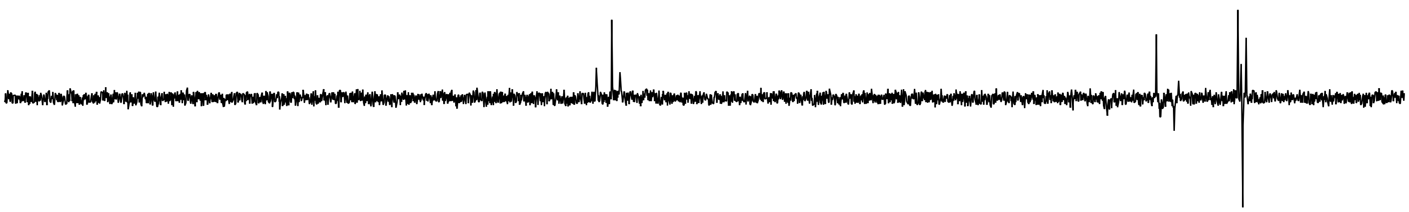

13C NMR (75 MHz, CDCl3)

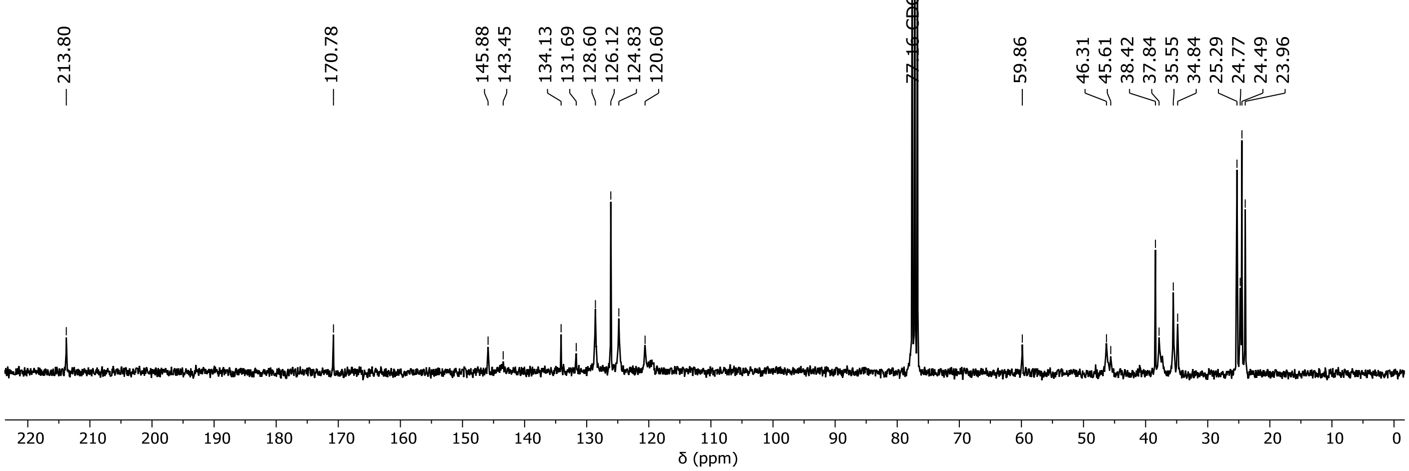

1H NMR (500 MHz, CDCl3)

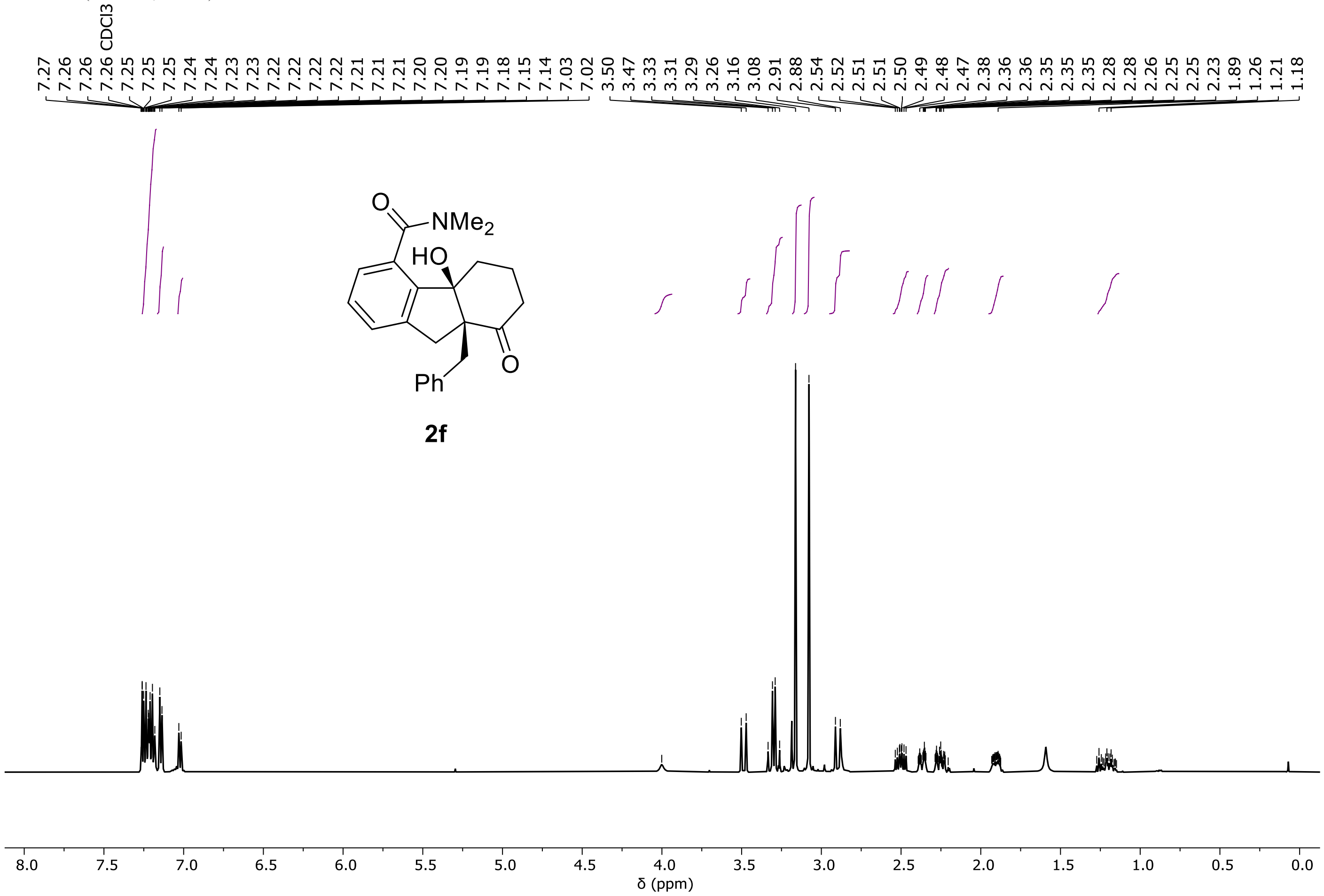

DEPT-135 NMR (126 MHz, CDCl3)

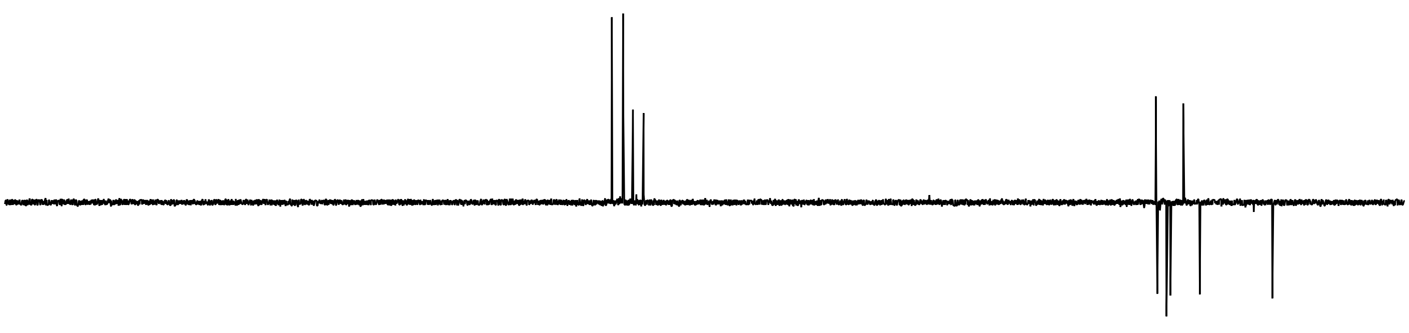

13C NMR (126 MHz, CDCl3)

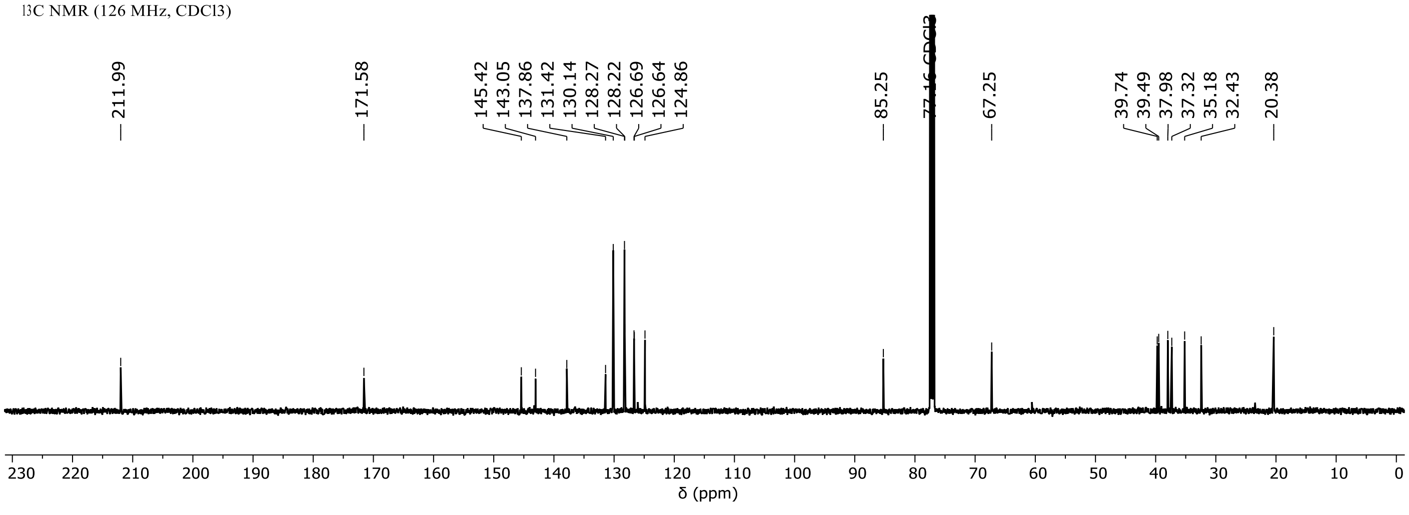

1H NMR (500 MHz, CDCl3)

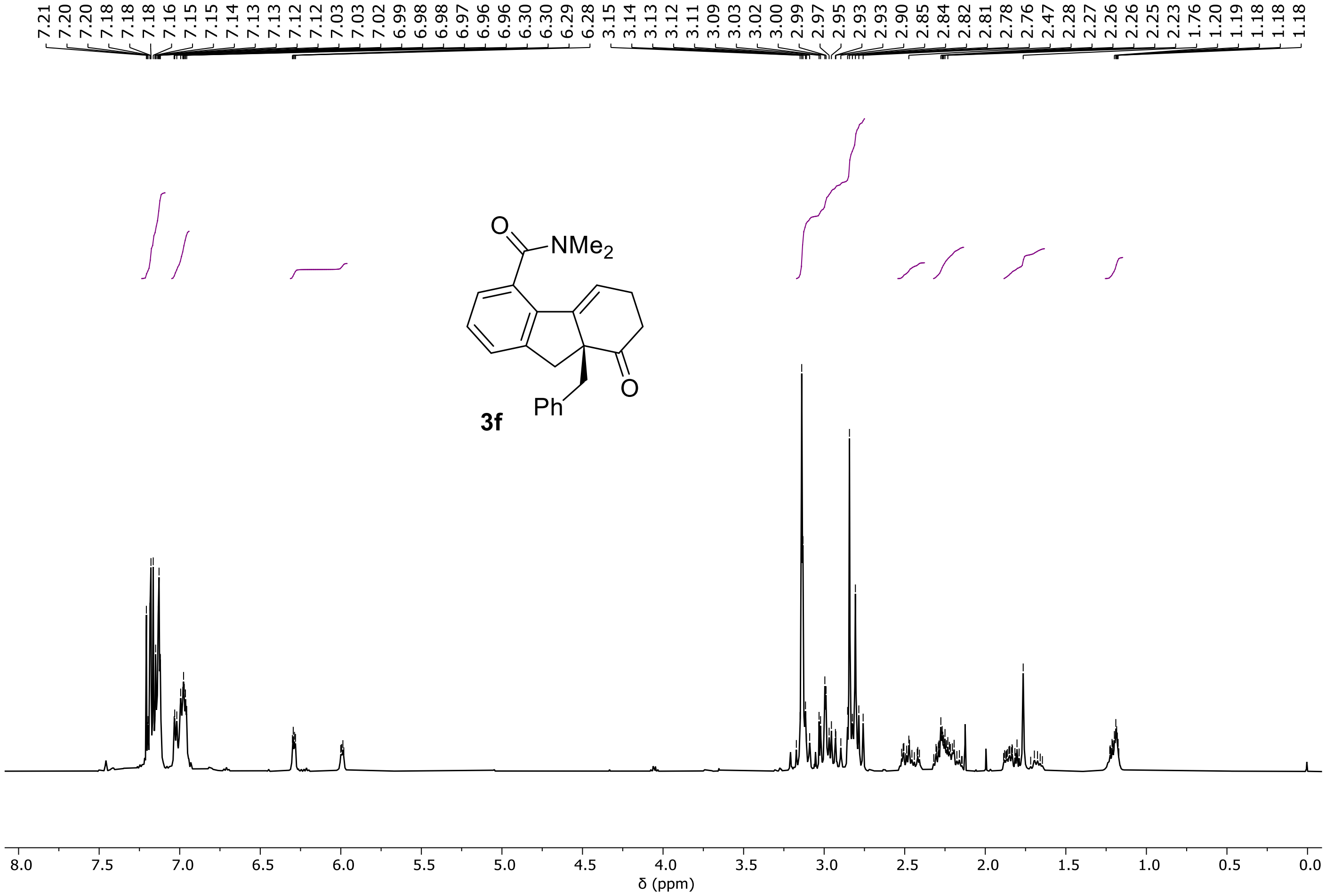

DEPT-135 NMR (126 MHz, CDCl3)

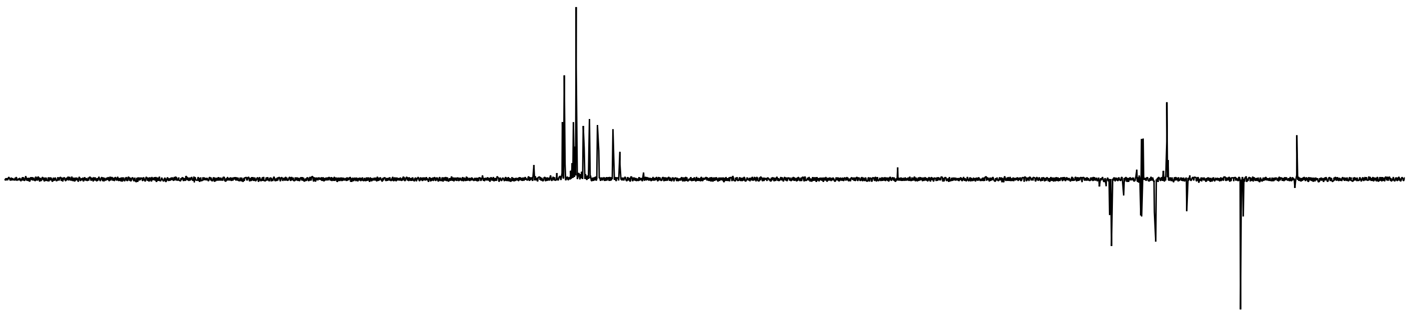

13C NMR (126 MHz, CDCl3)

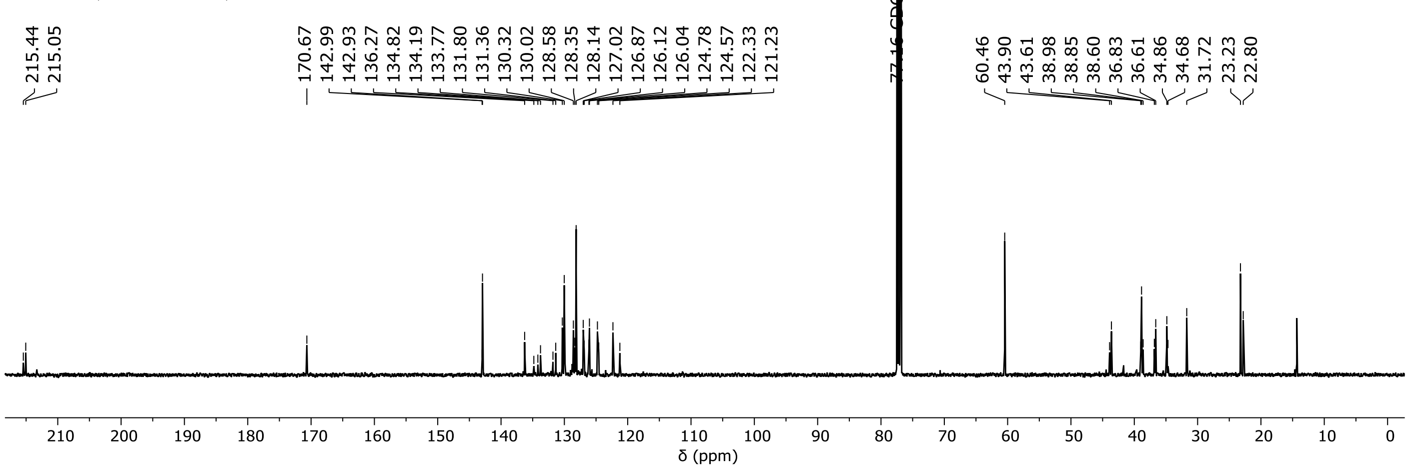

1H NMR (300 MHz, CDCl3)

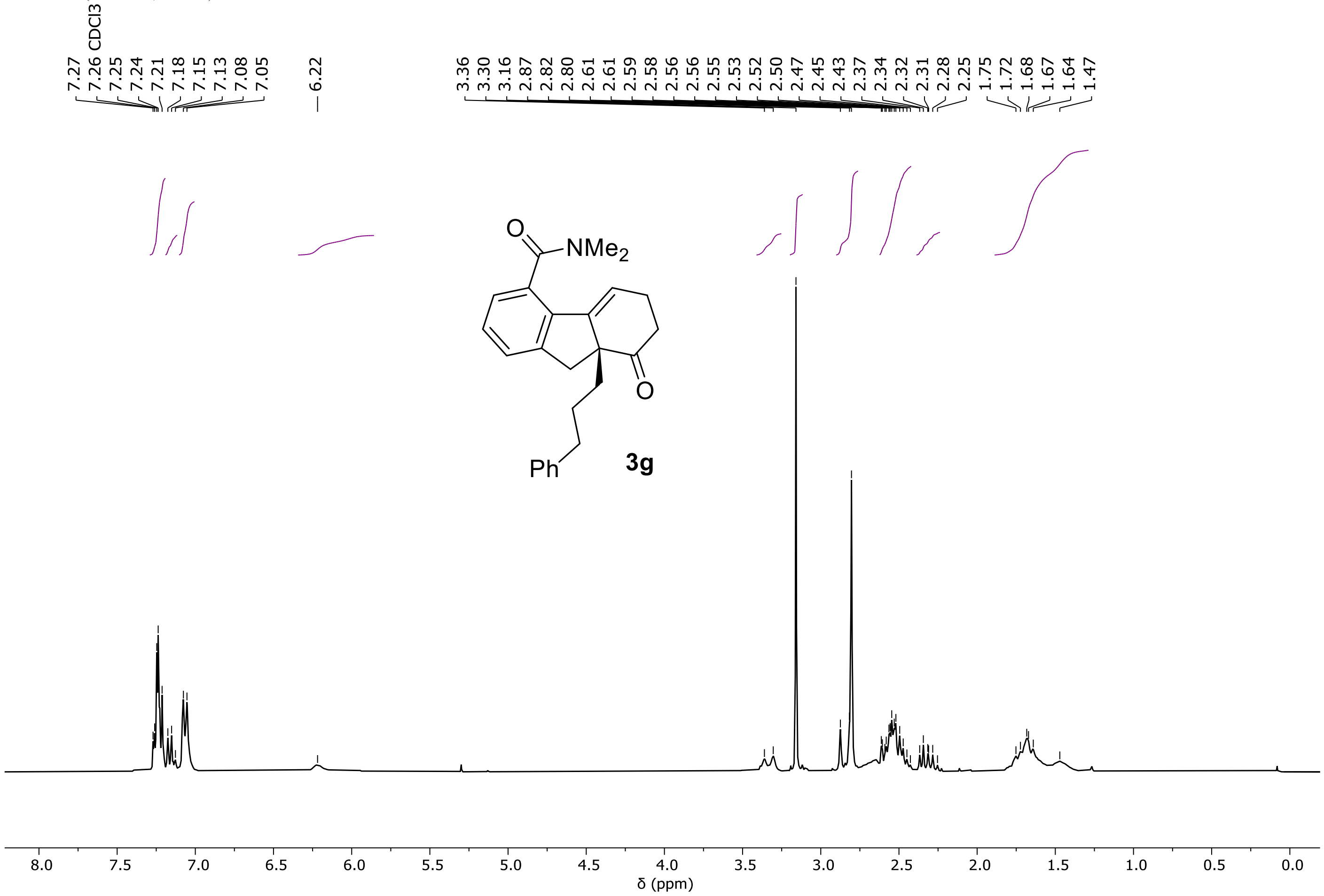

DEPT-135 NMR (75 MHz, CDCl3)

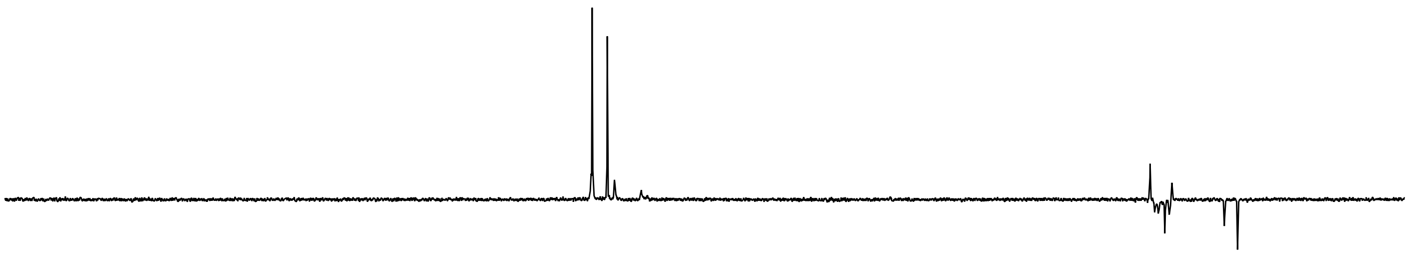

13C NMR (75 MHz, CDCl3)

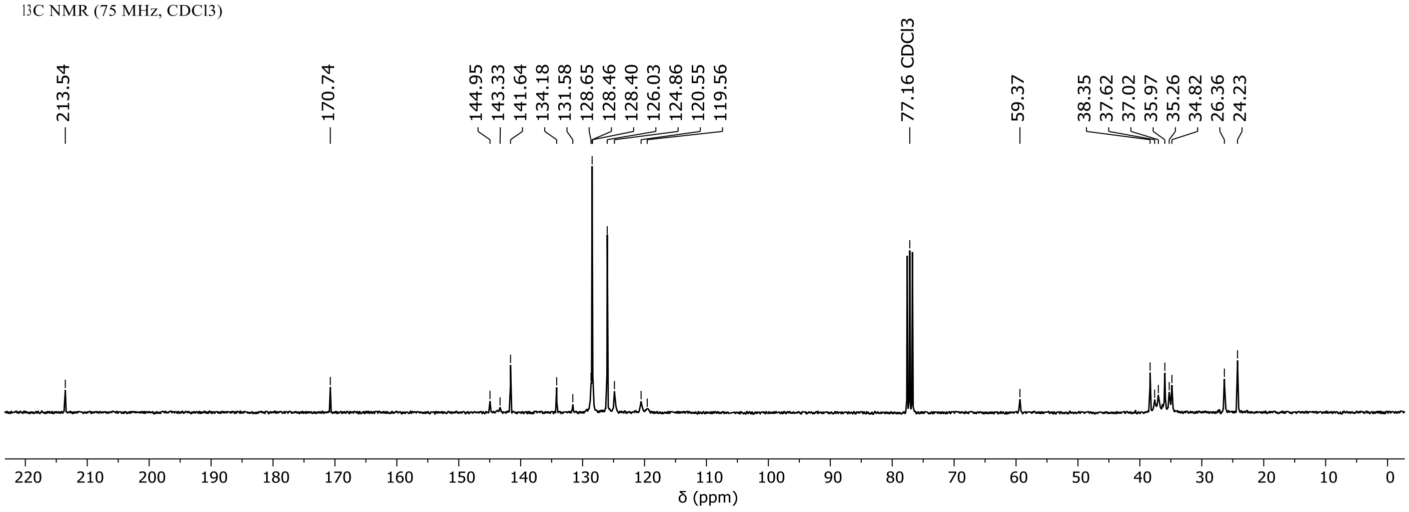

1H NMR (500 MHz, CDCl3)

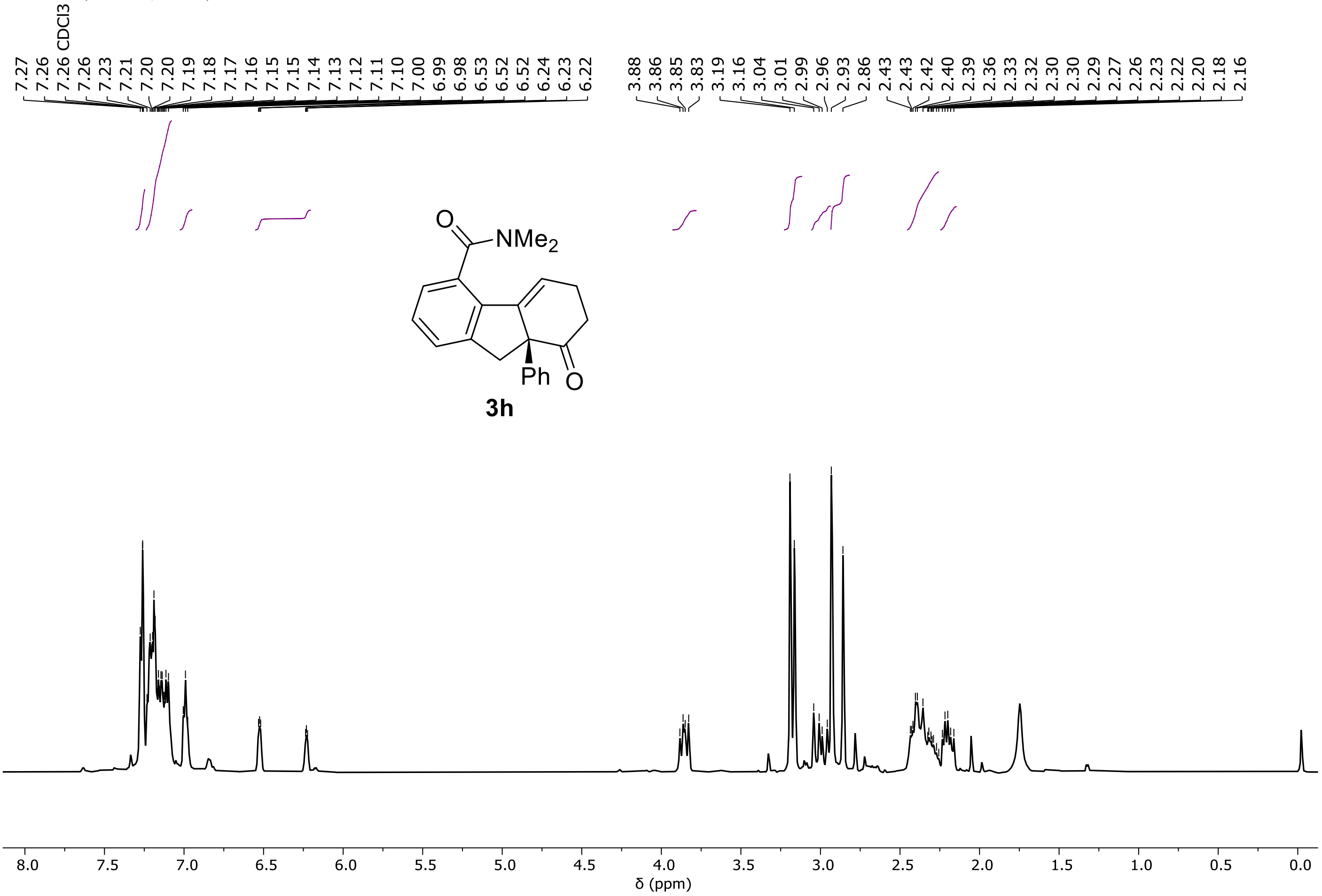

DEPT-135 NMR (126 MHz, CDCl3)

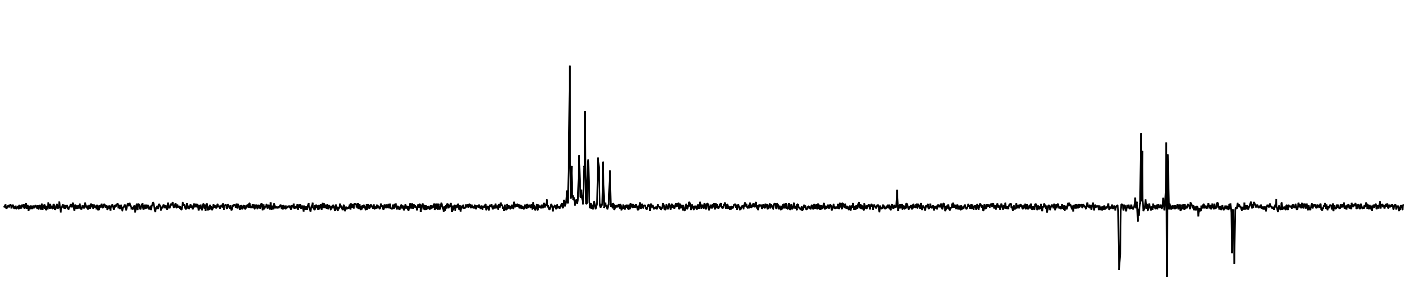

13C NMR (126 MHz, CDCl3)

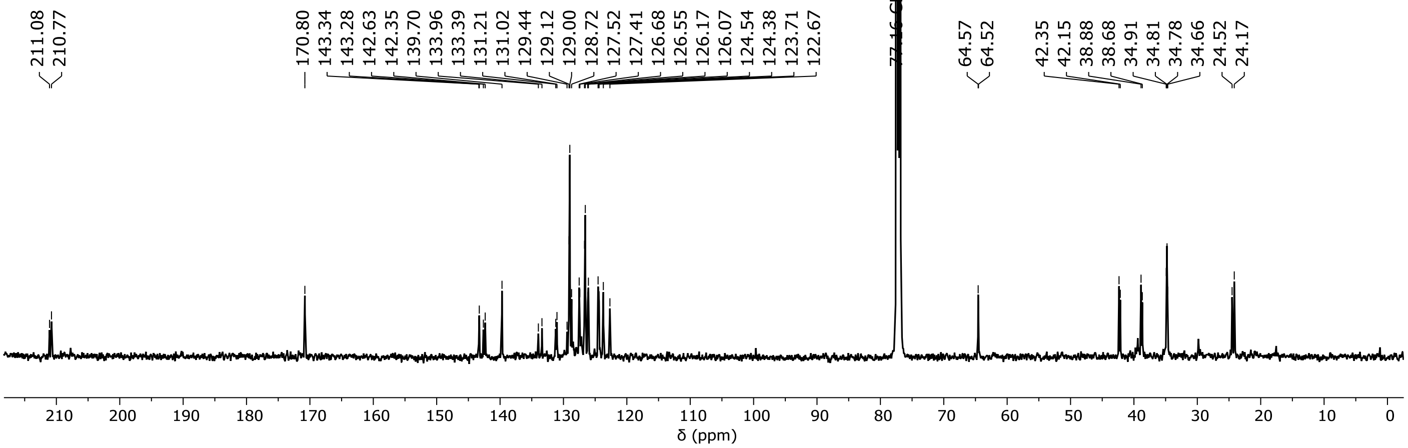

1H NMR (300 MHz, CDCl3)

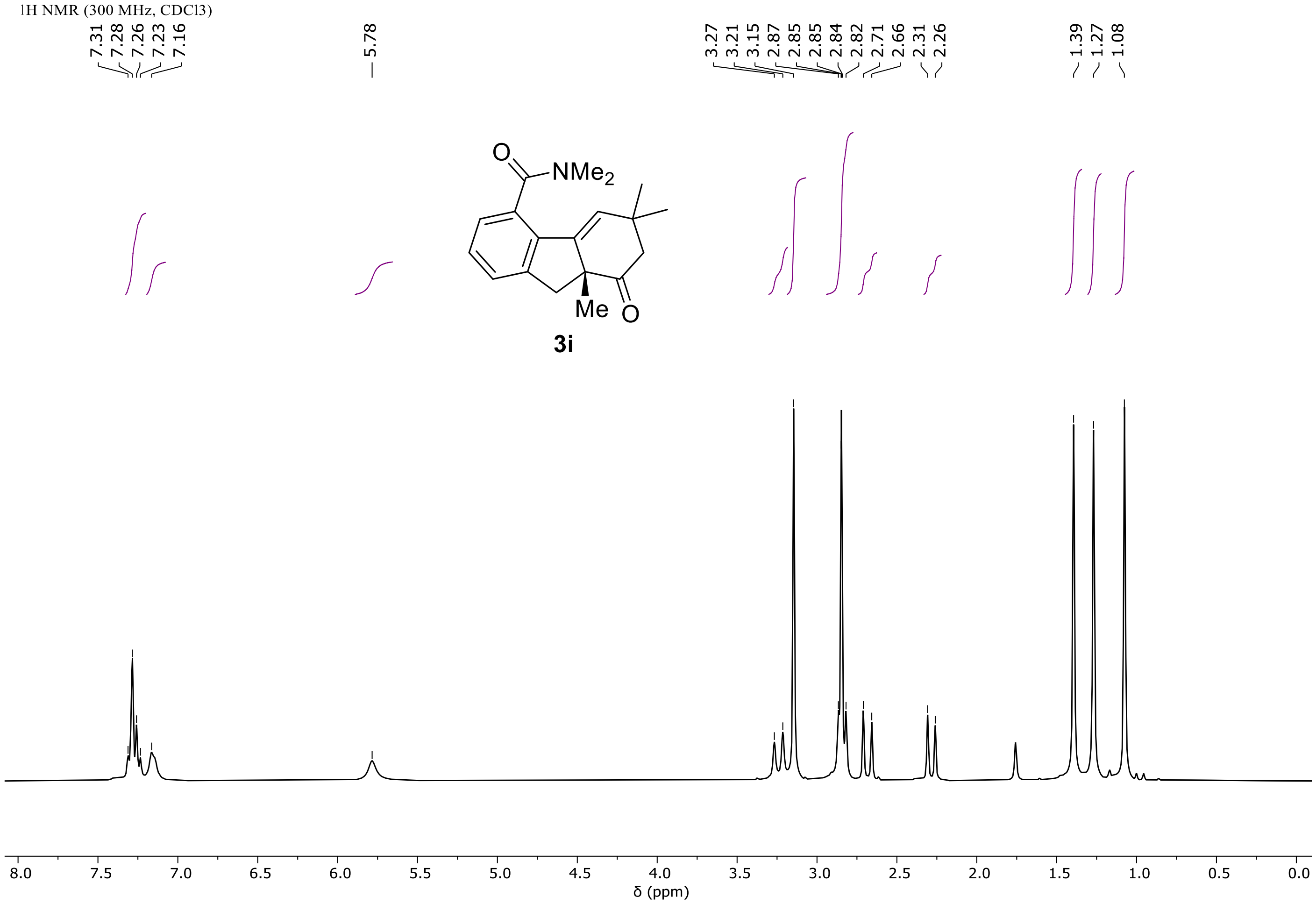

DEPT-135 NMR (75 MHz, CDCl3)

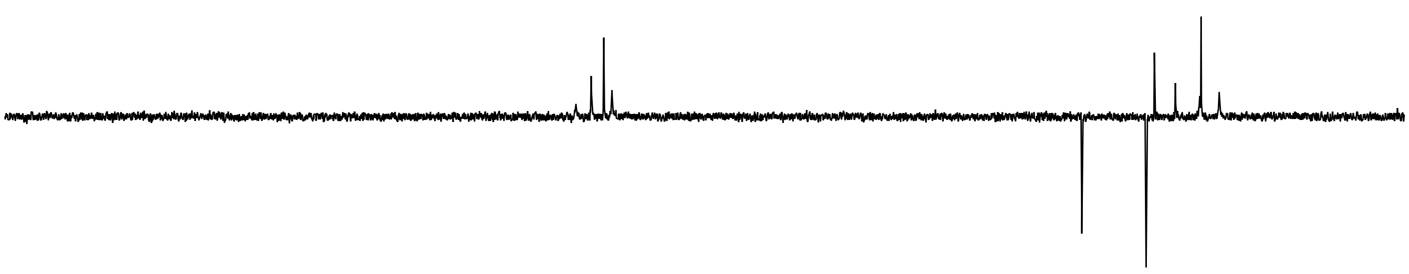

13C NMR (75 MHz, CDCl3)

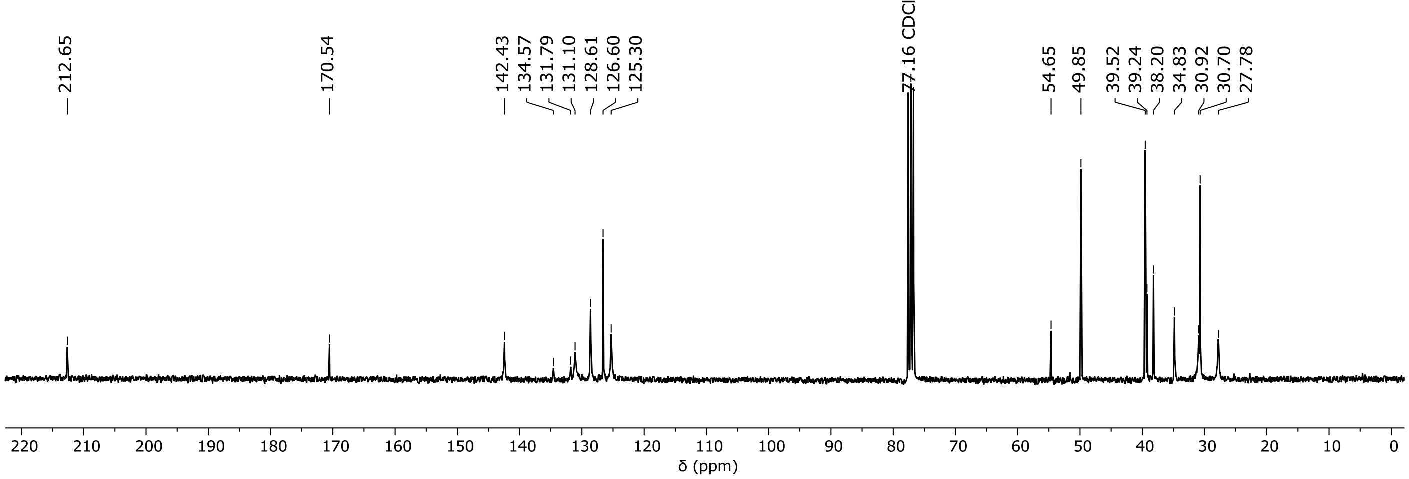

1H NMR (300 MHz, CDCl3)

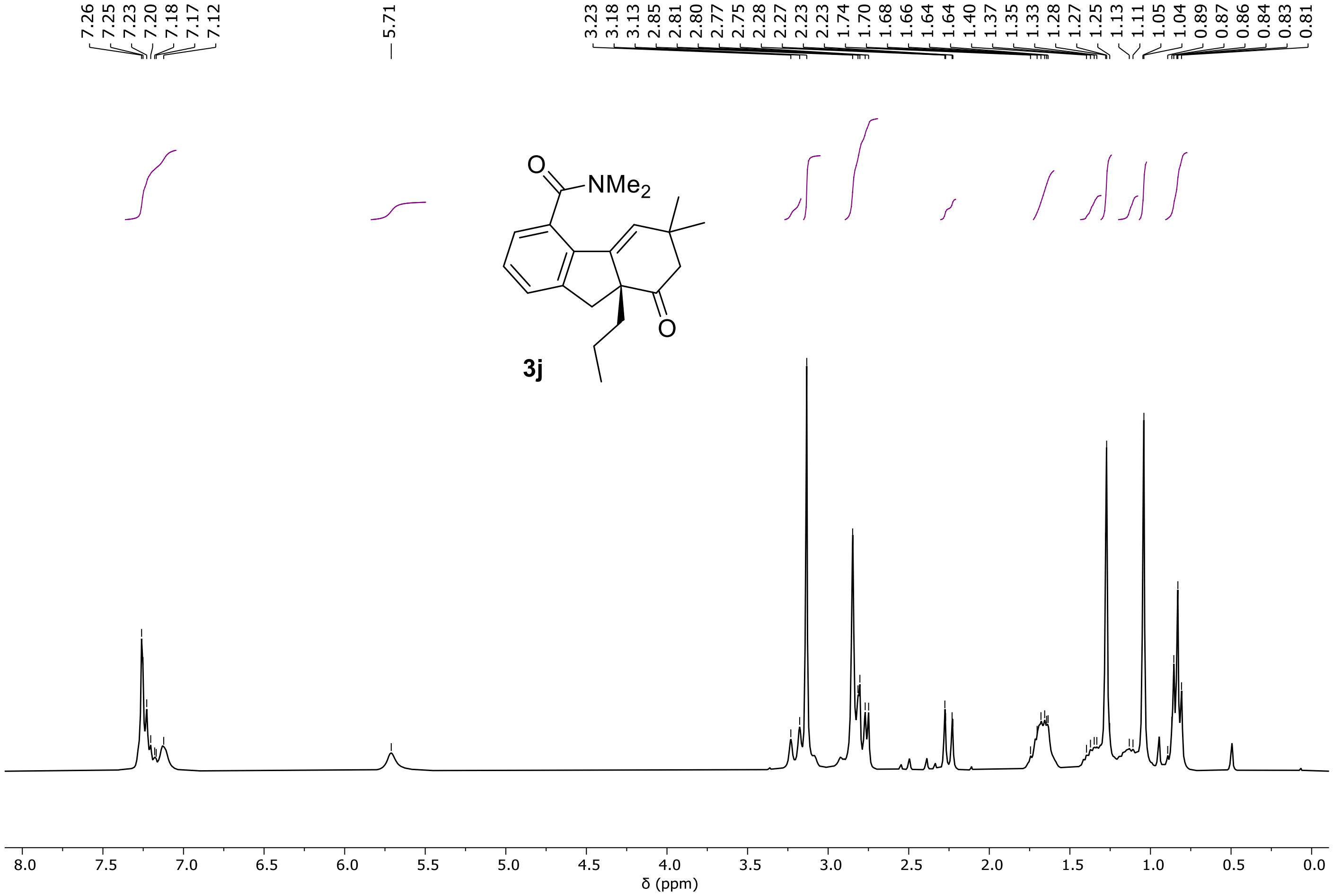

DEPT-135 NMR (75 MHz, CDCl3)

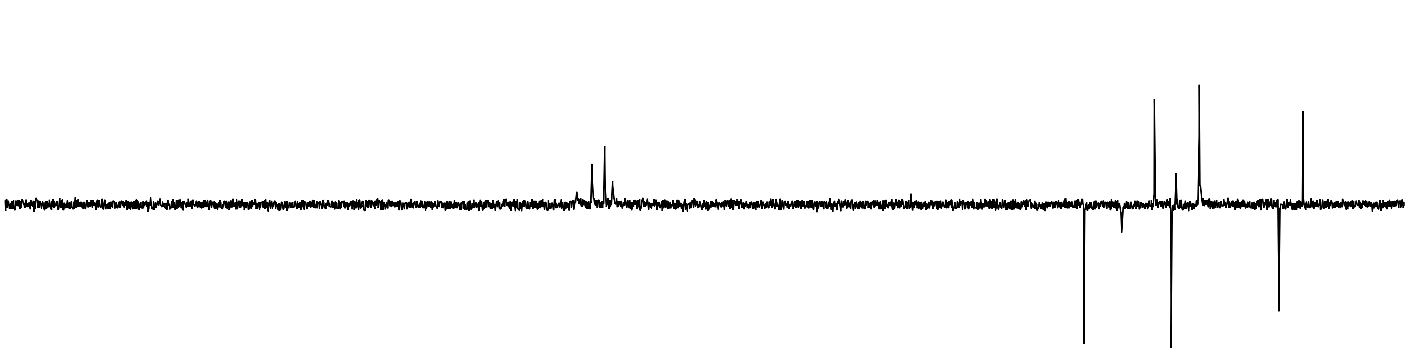

13C NMR (75 MHz, CDCl3)

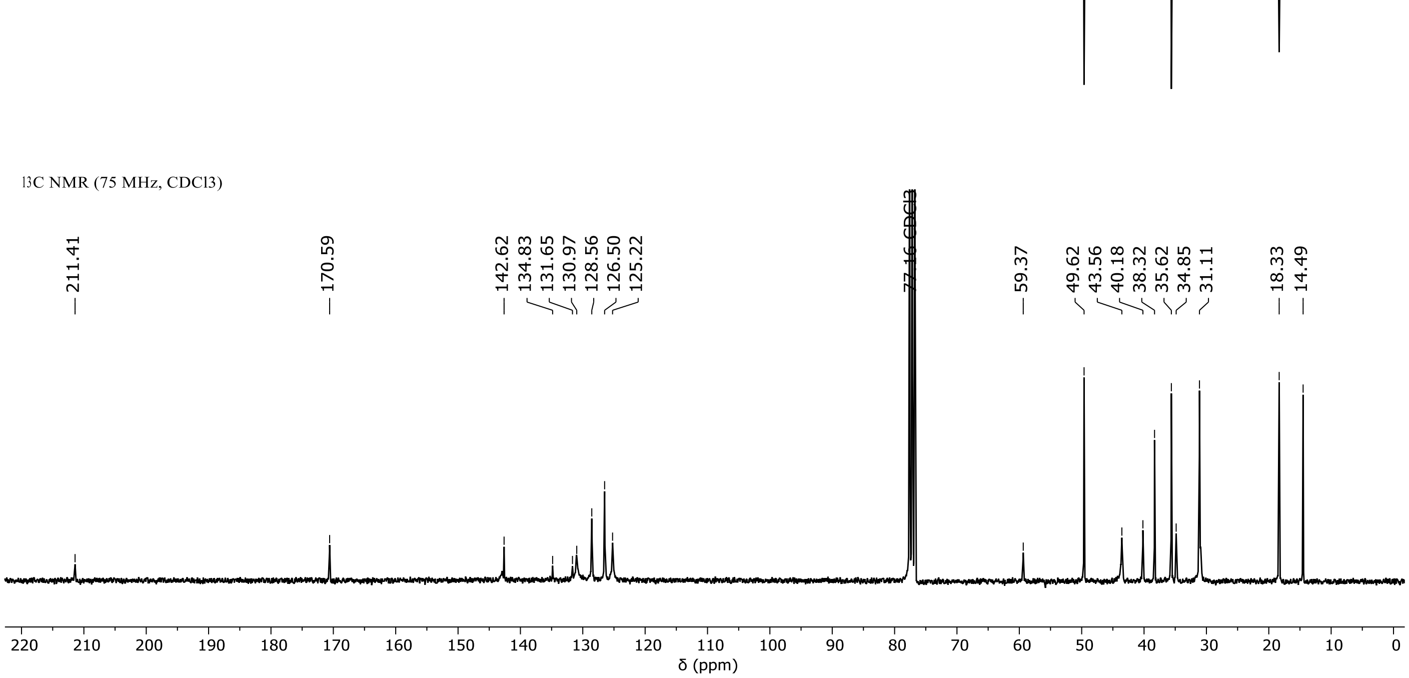

1H NMR (500 MHz, CDCl3)

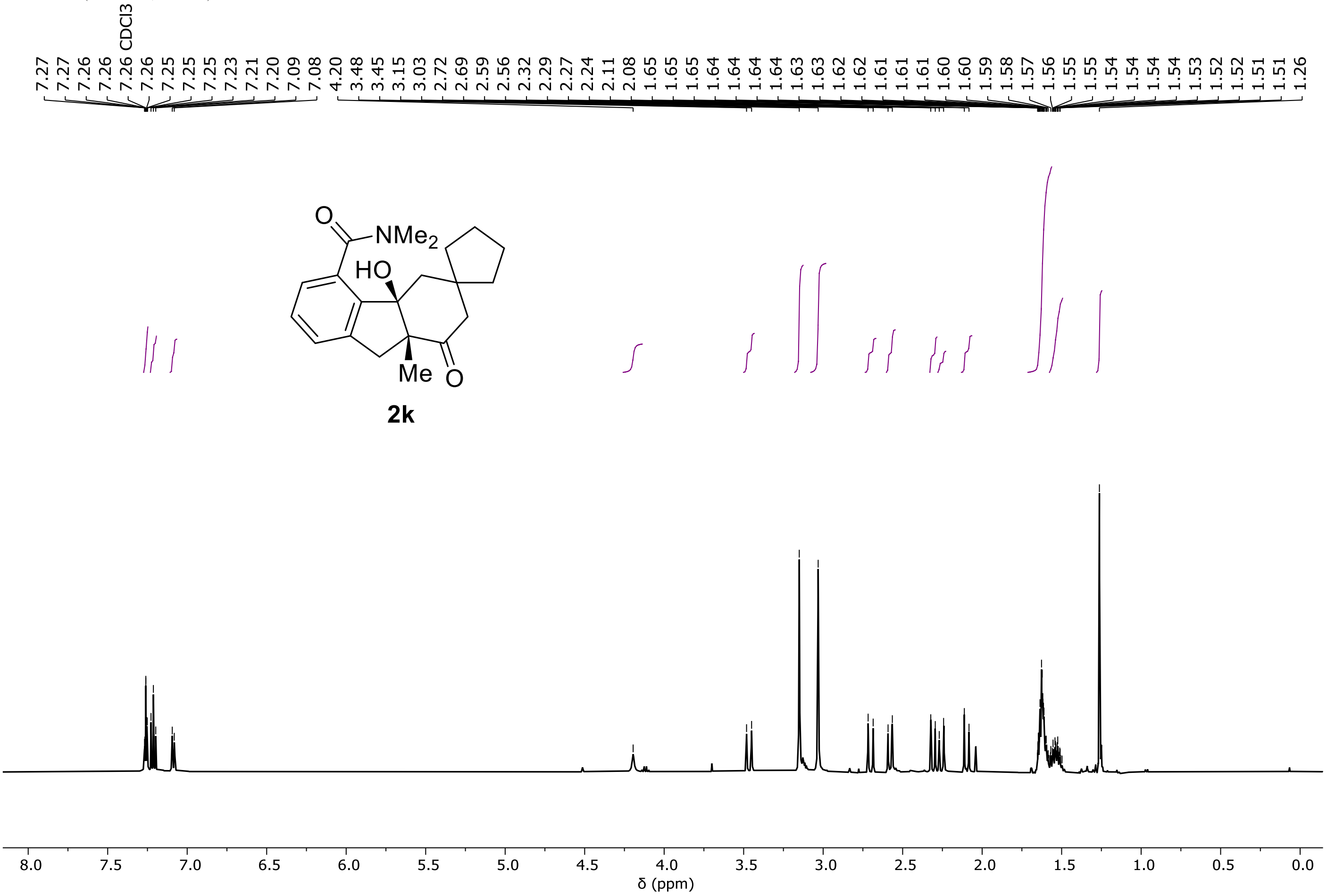

DEPT-135 NMR (126 MHz, CDCl3)

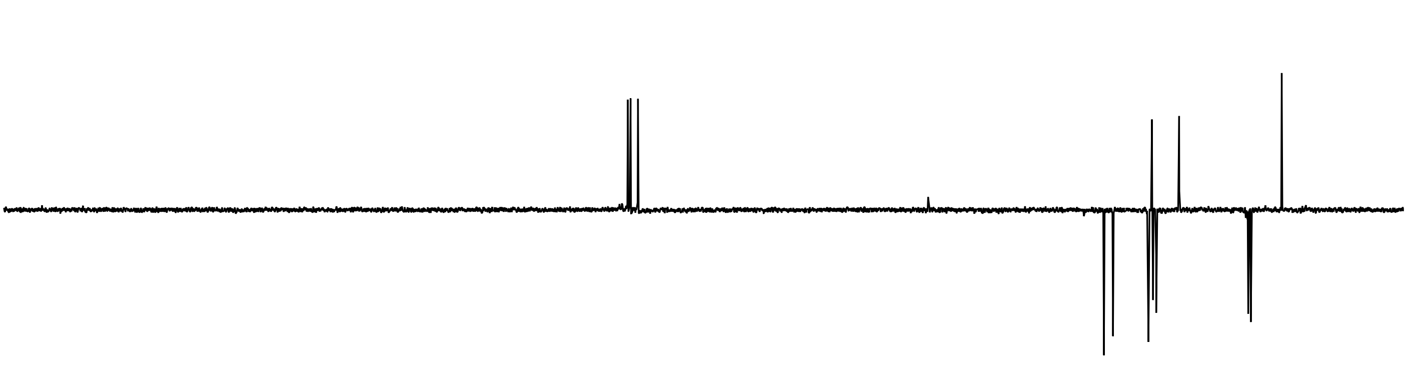

13C NMR (126 MHz, CDCl3)

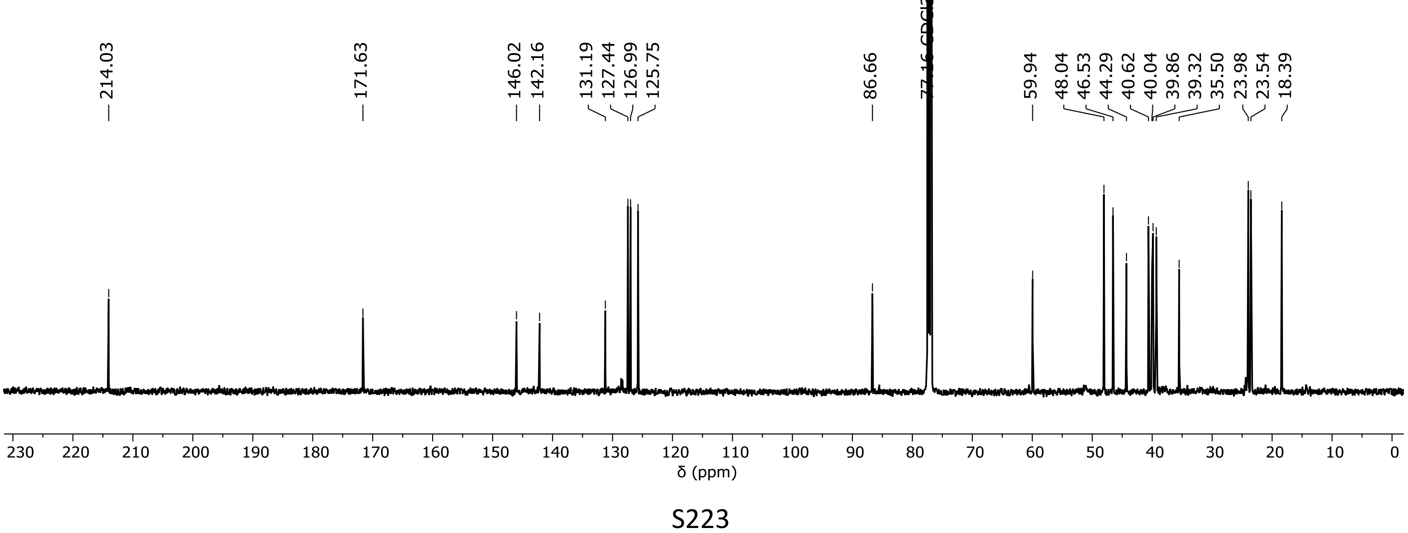

1H NMR (300 MHz, CDCl3)

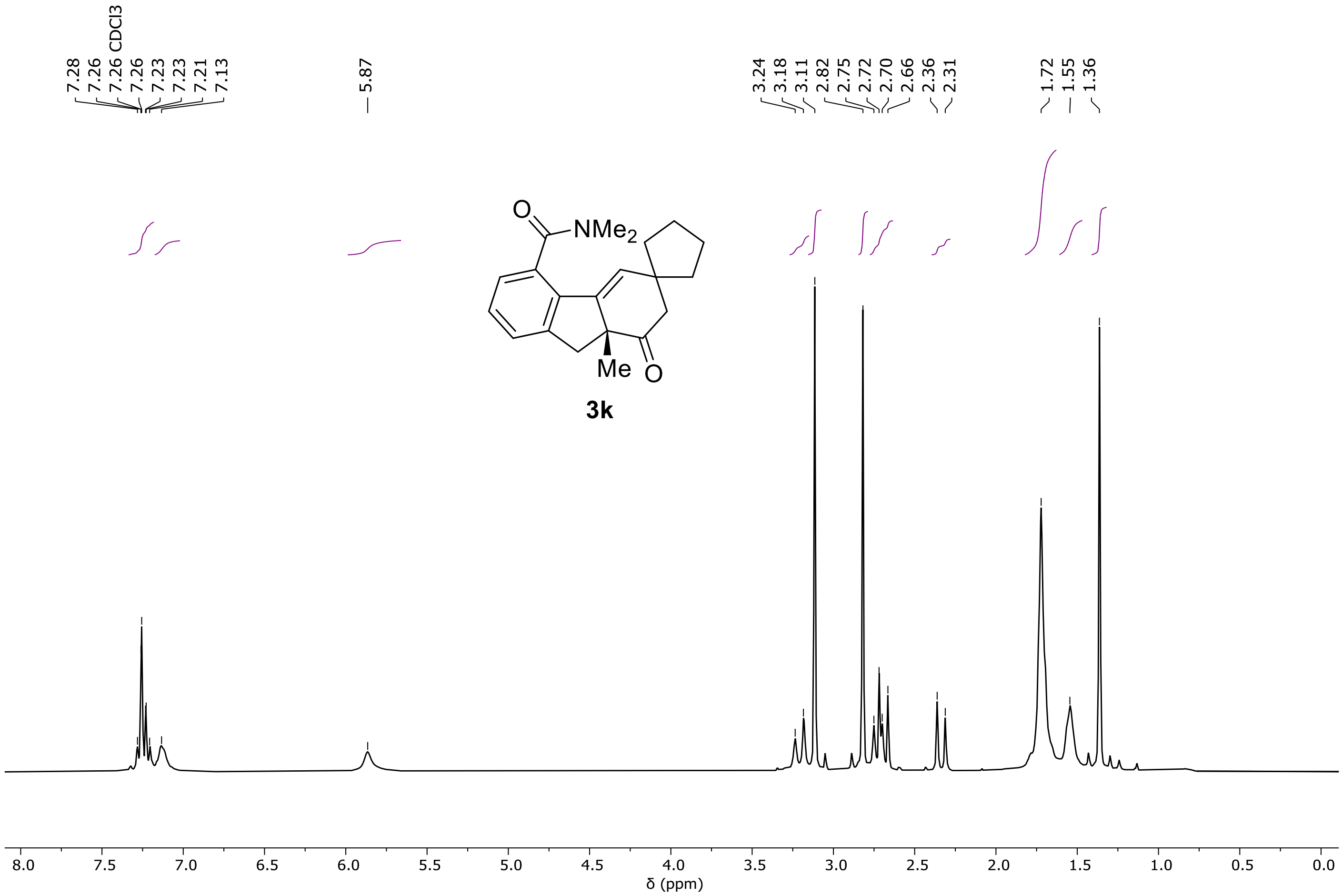

DEPT-135 NMR (75 MHz, CDCl3)

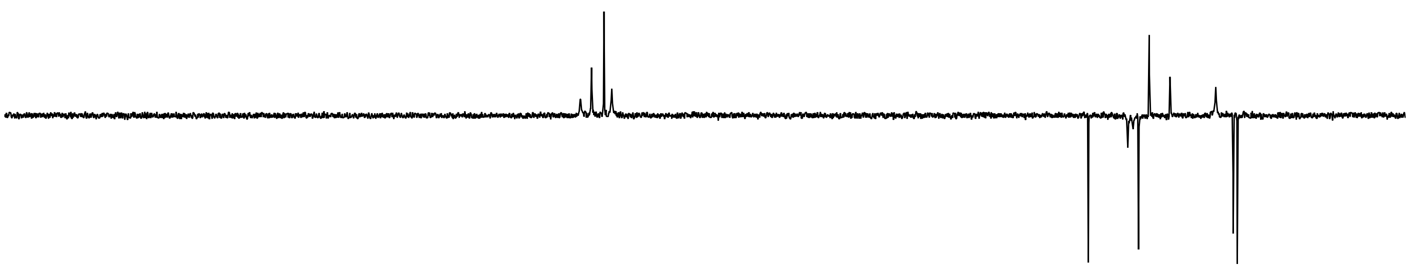

13C NMR (75 MHz, CDCl3)

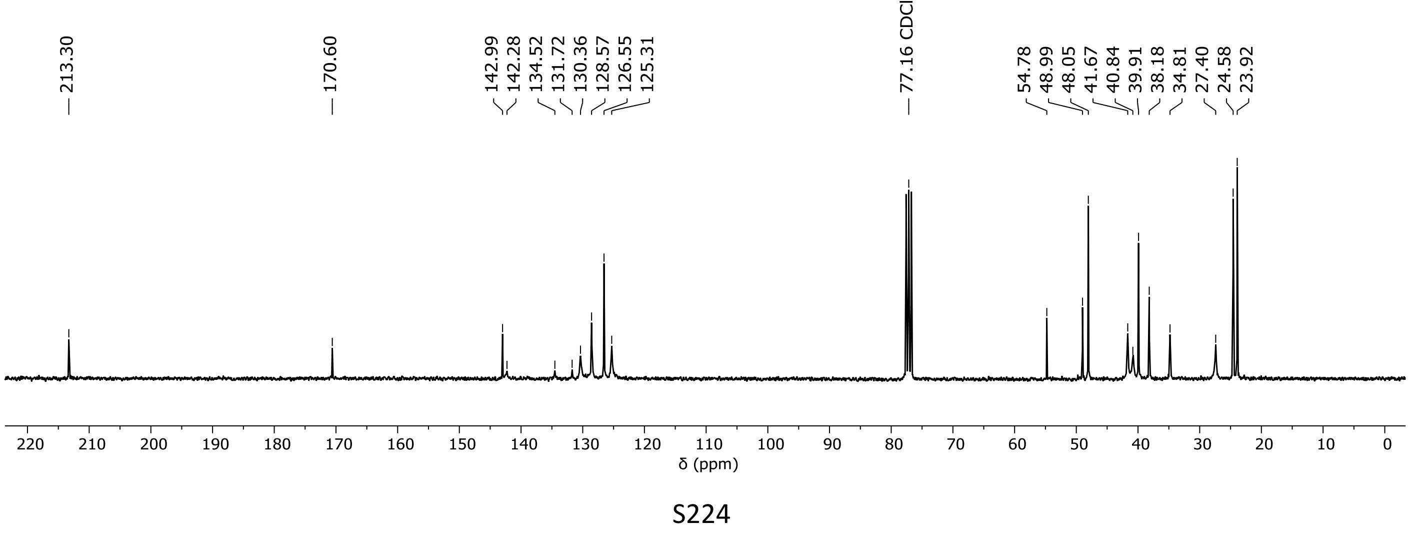

1H NMR (300 MHz, CDCl3)

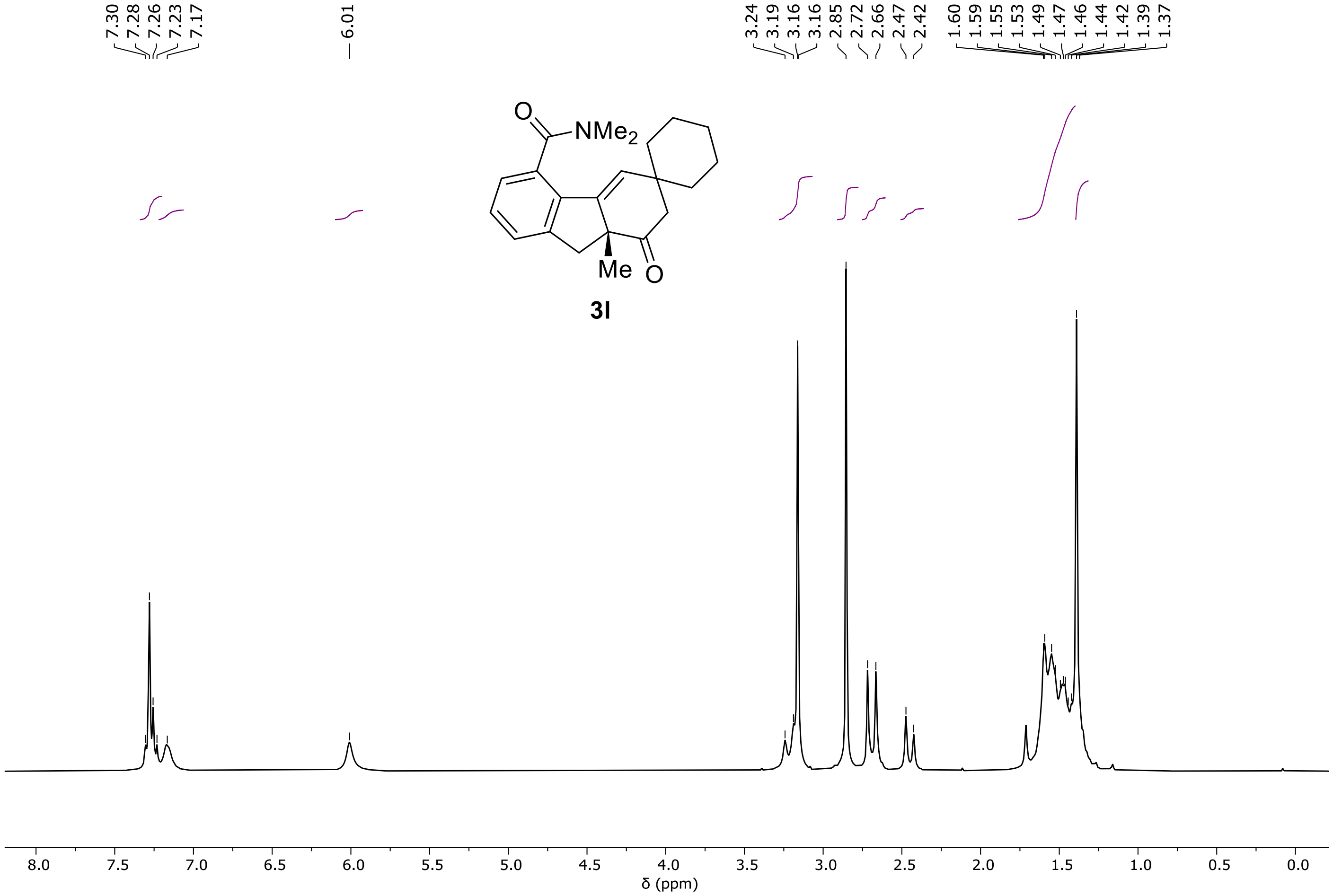

DEPT-135 NMR (75 MHz, CDCl3)

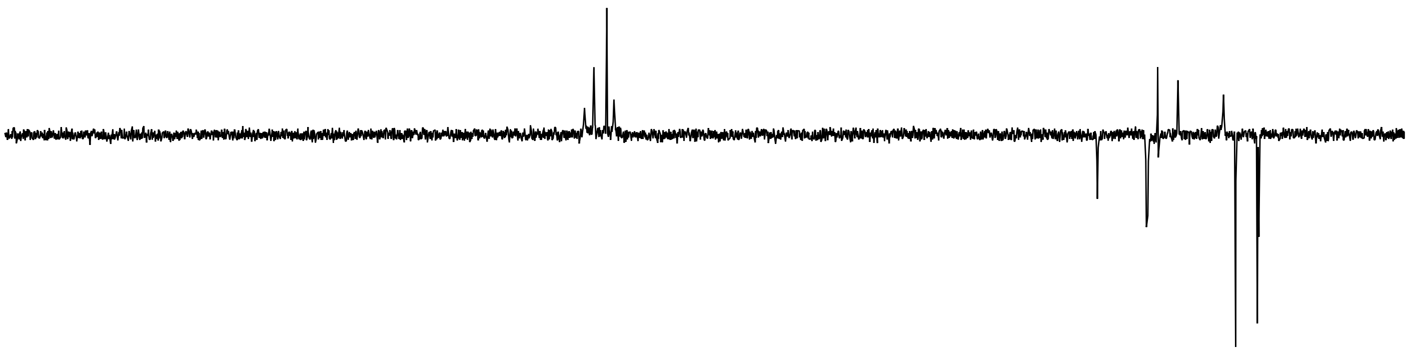

13C NMR (75 MHz, CDCl3)

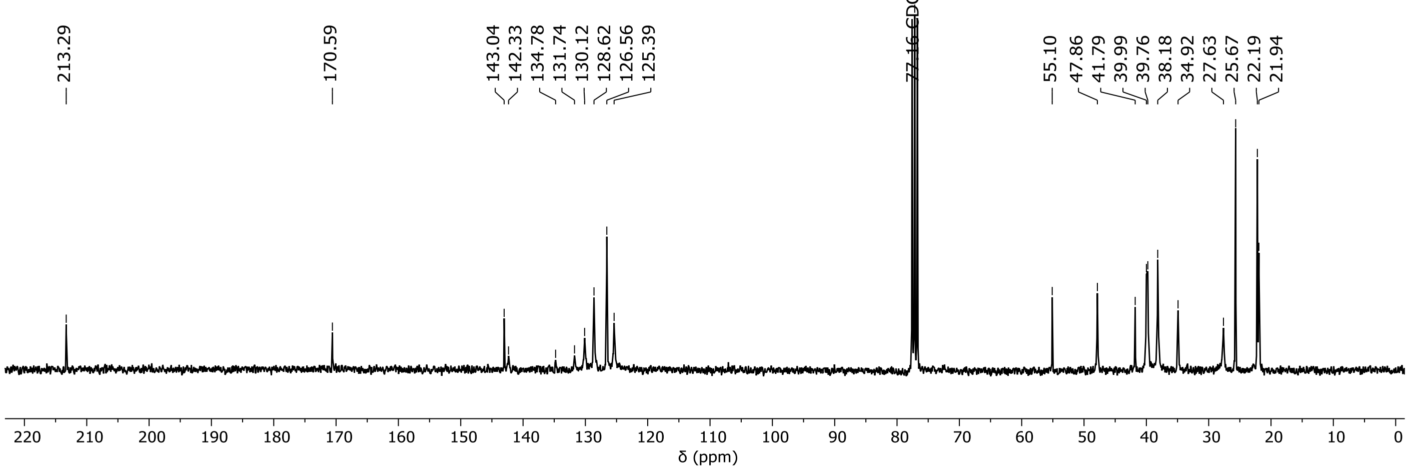

1H NMR (500 MHz, CDCl3)

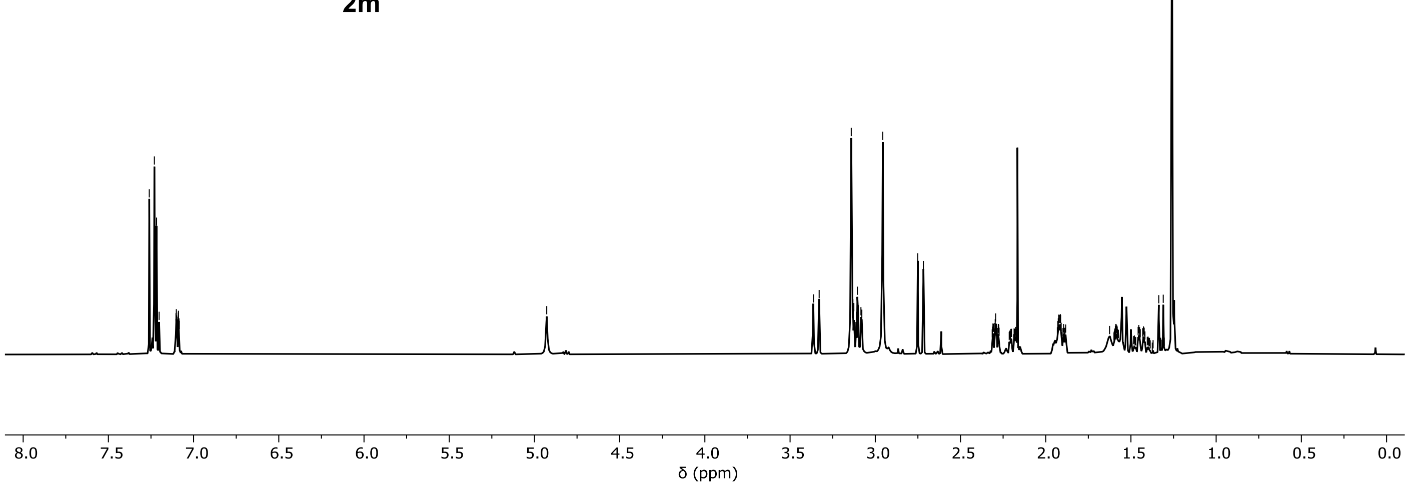

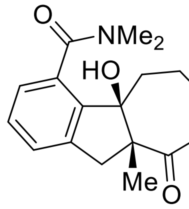

2m

DEPT-135 NMR (126 MHz, CDCl3)

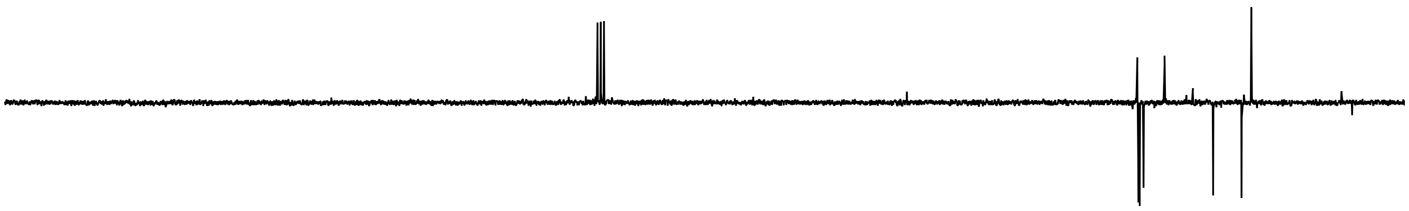

13C NMR (126 MHz, CDCl3)

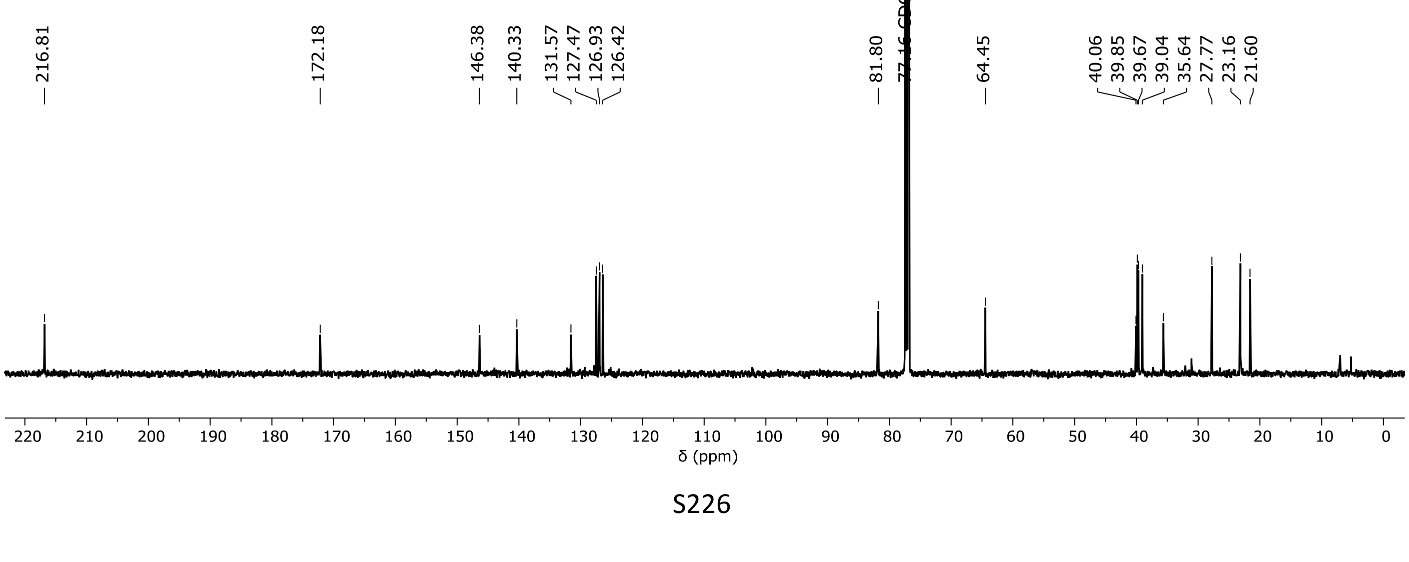

1H NMR (500 MHz, CDCl3)

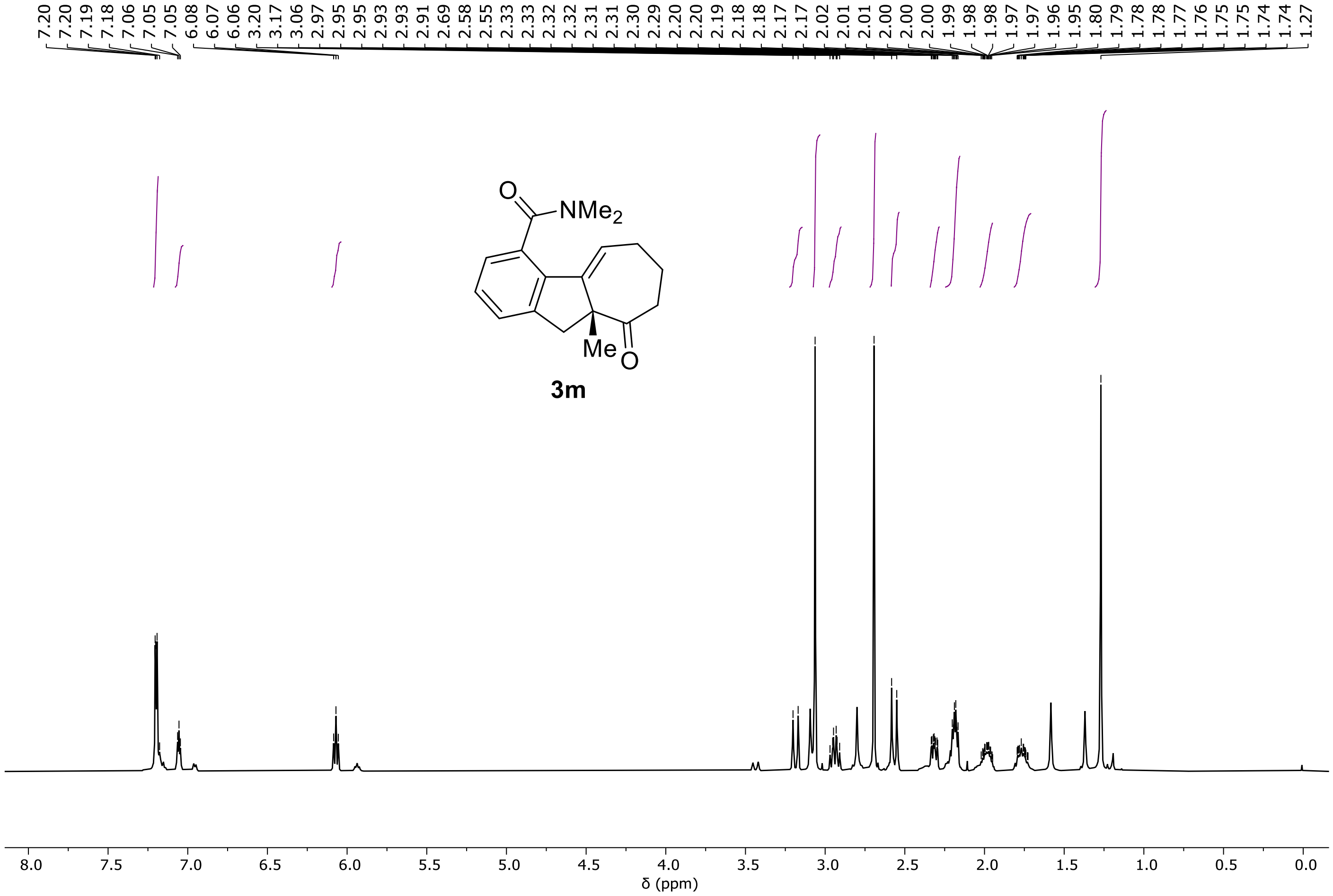

DEPT-135 NMR (126 MHz, CDCl3)

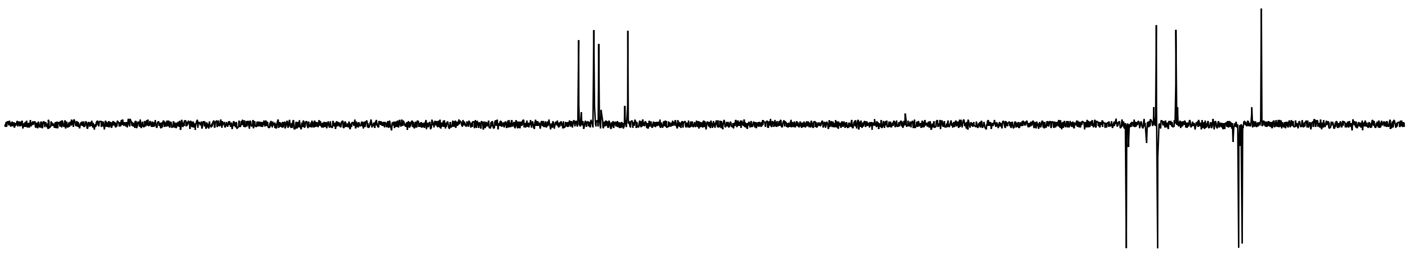

13C NMR (126 MHz, CDCl3)

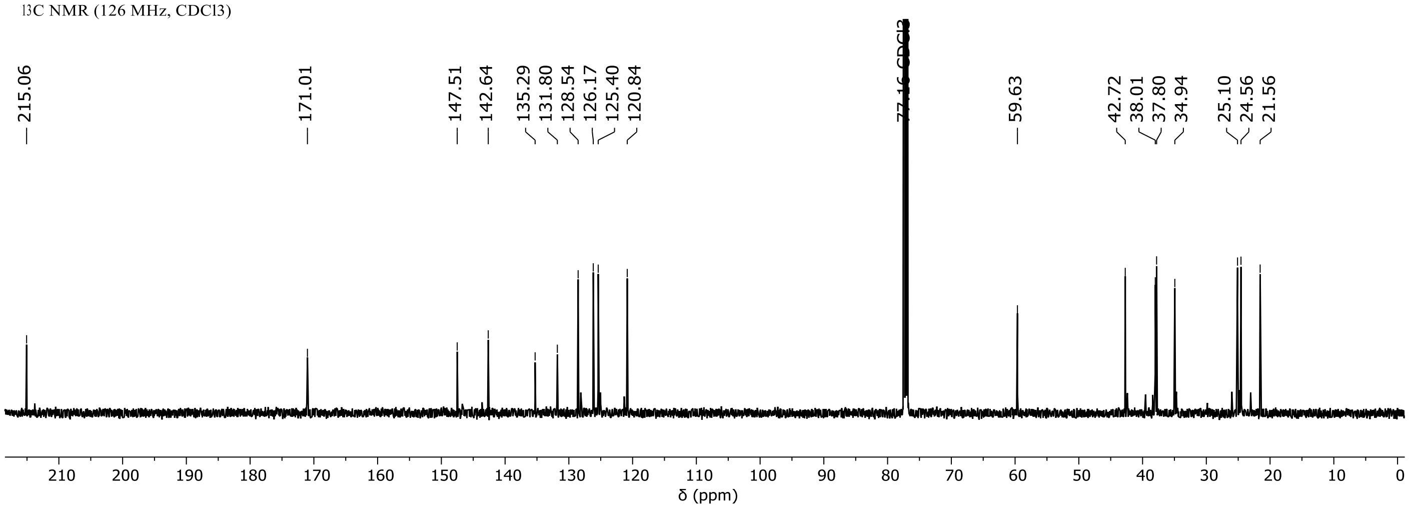

1H NMR (300 MHz, CDCl3)

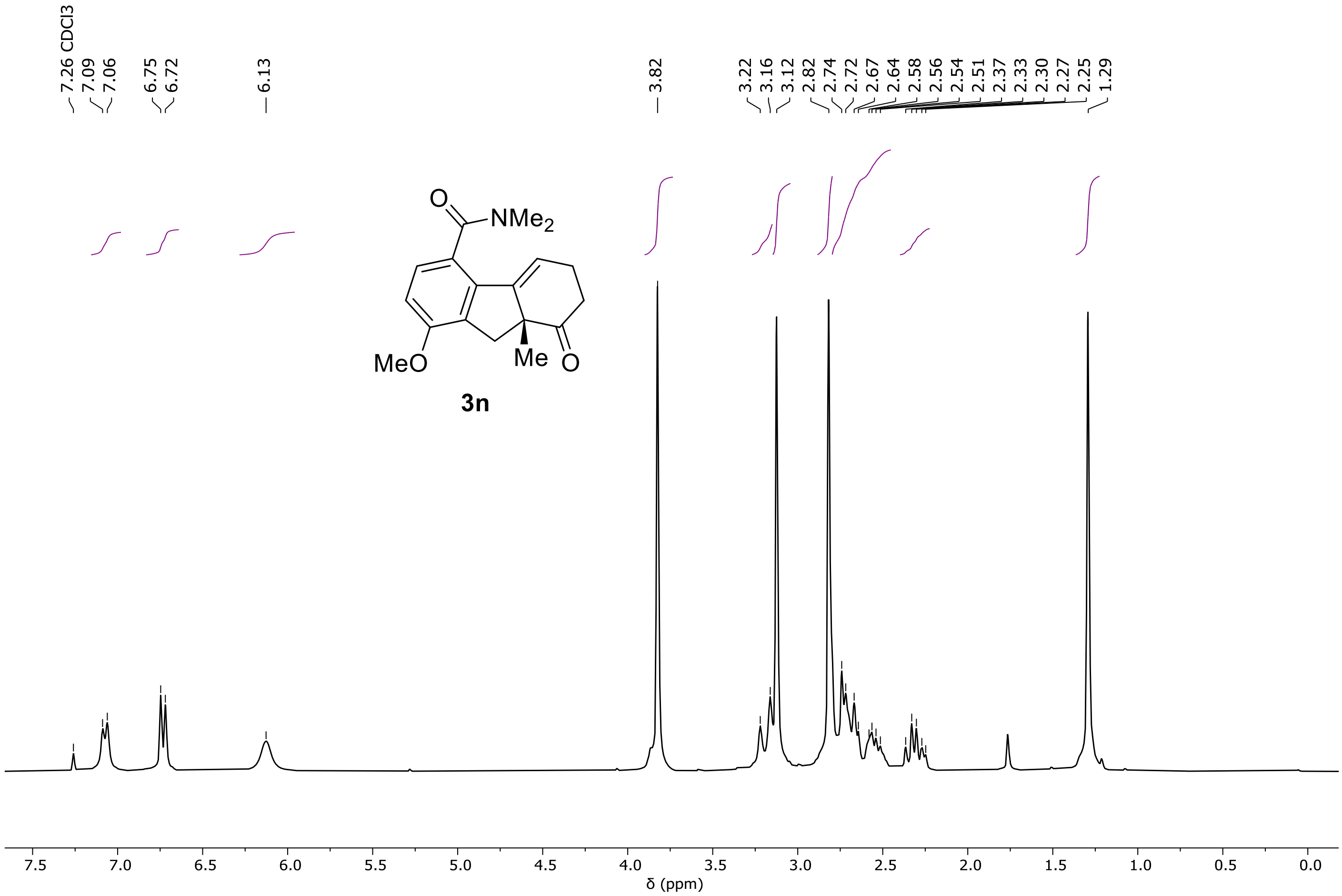

DEPT-135 NMR (75 MHz, CDCl3)

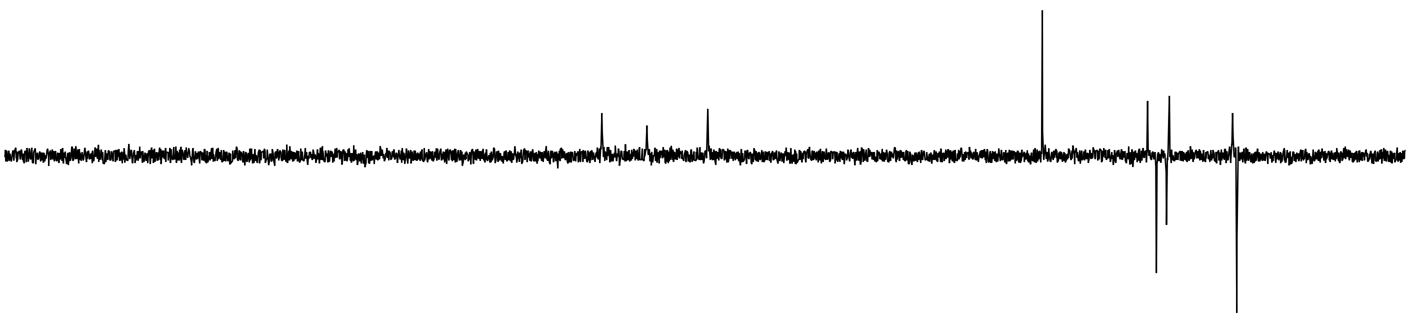

13C NMR (75 MHz, CDCl3)

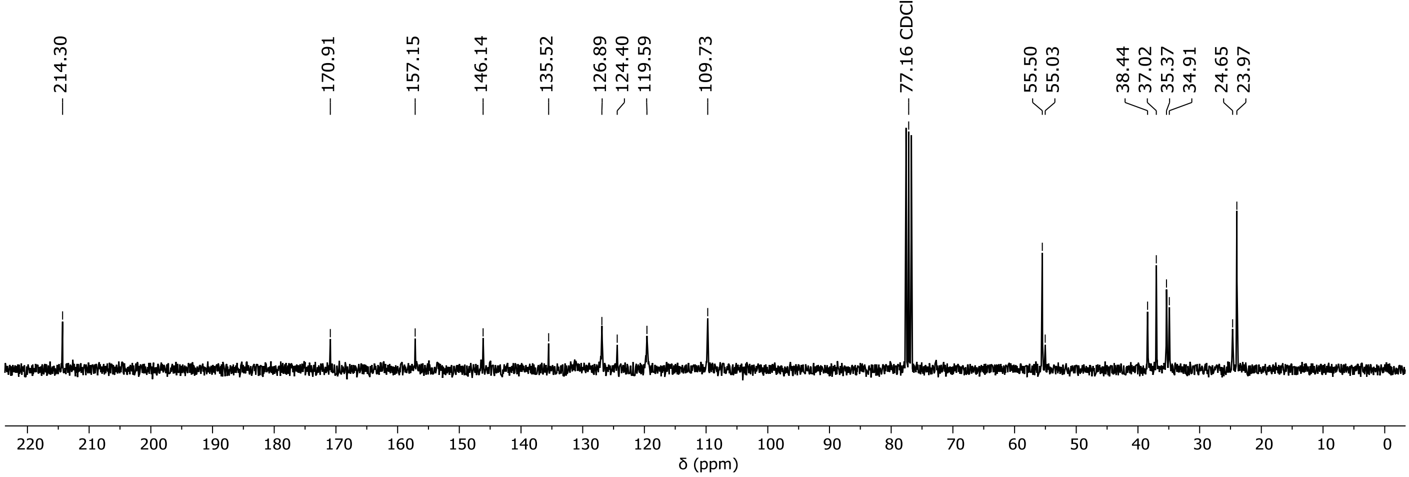

1H NMR (300 MHz, CDCl3)

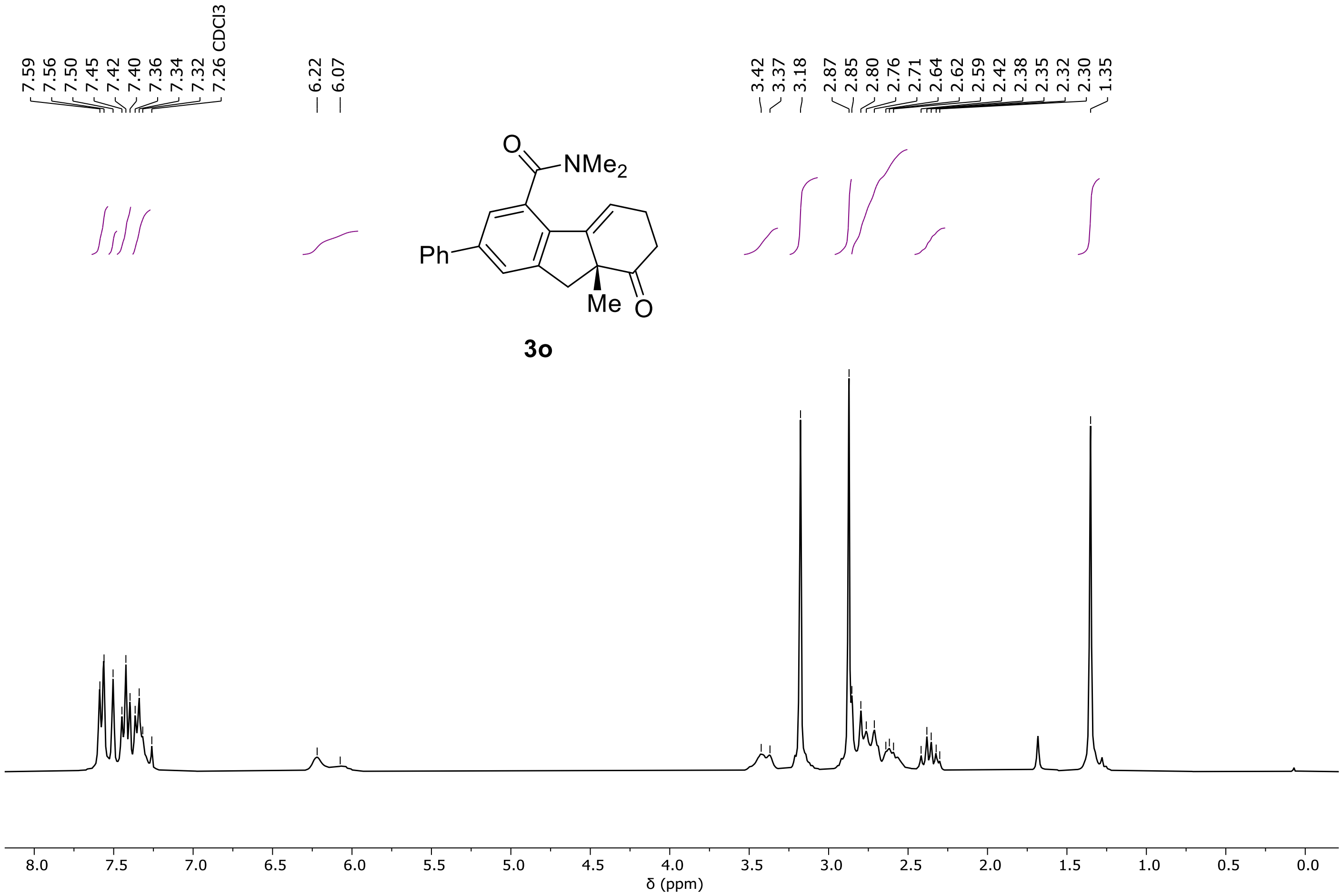

3o

DEPT-135 NMR (75 MHz, CDCl3)

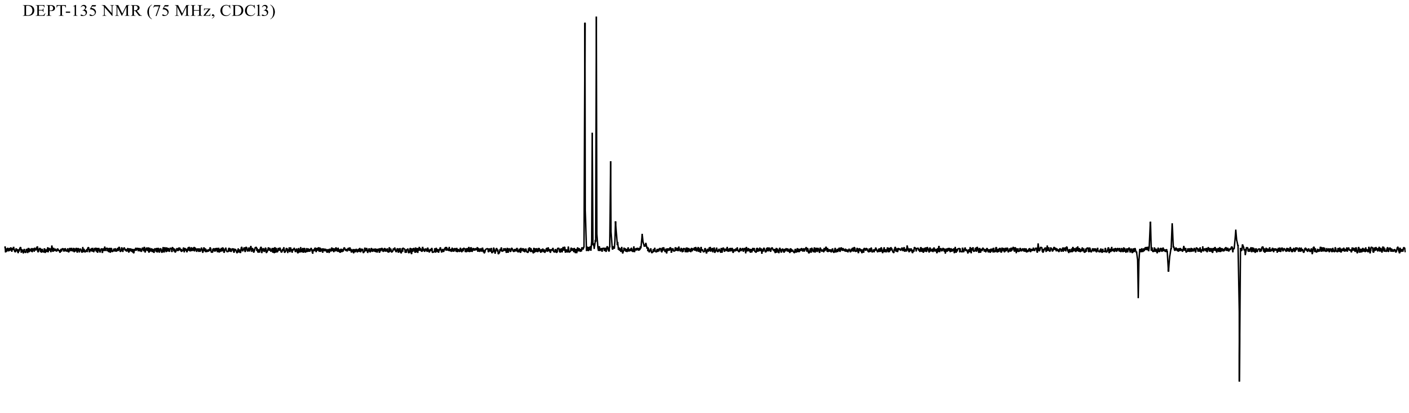

13C NMR (75 MHz, CDCl3)

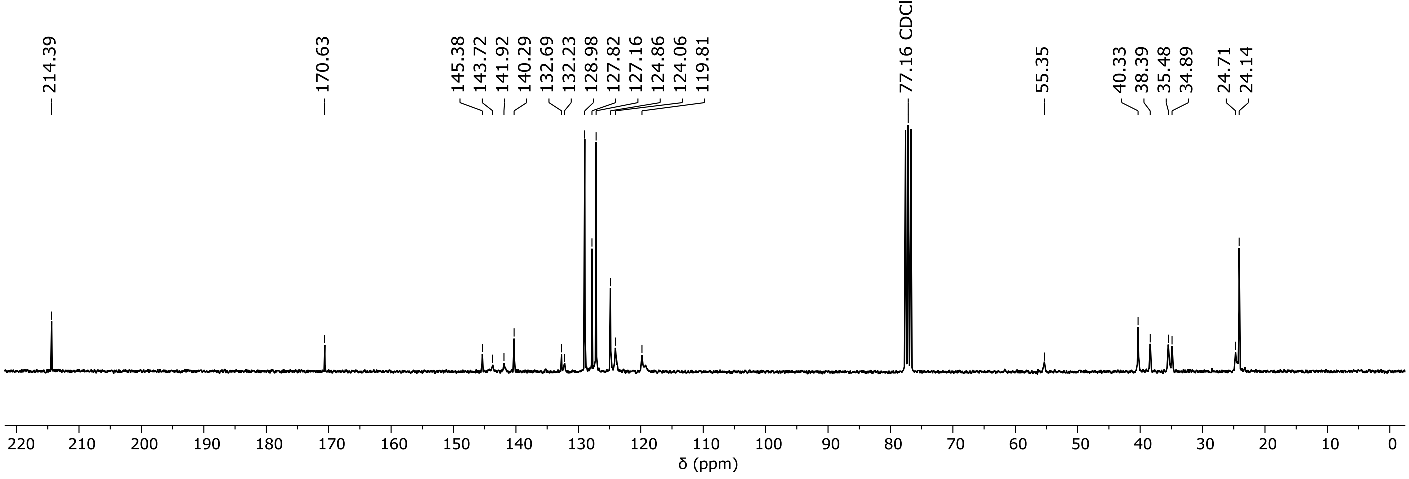

1H NMR (300 MHz, CDCl3)

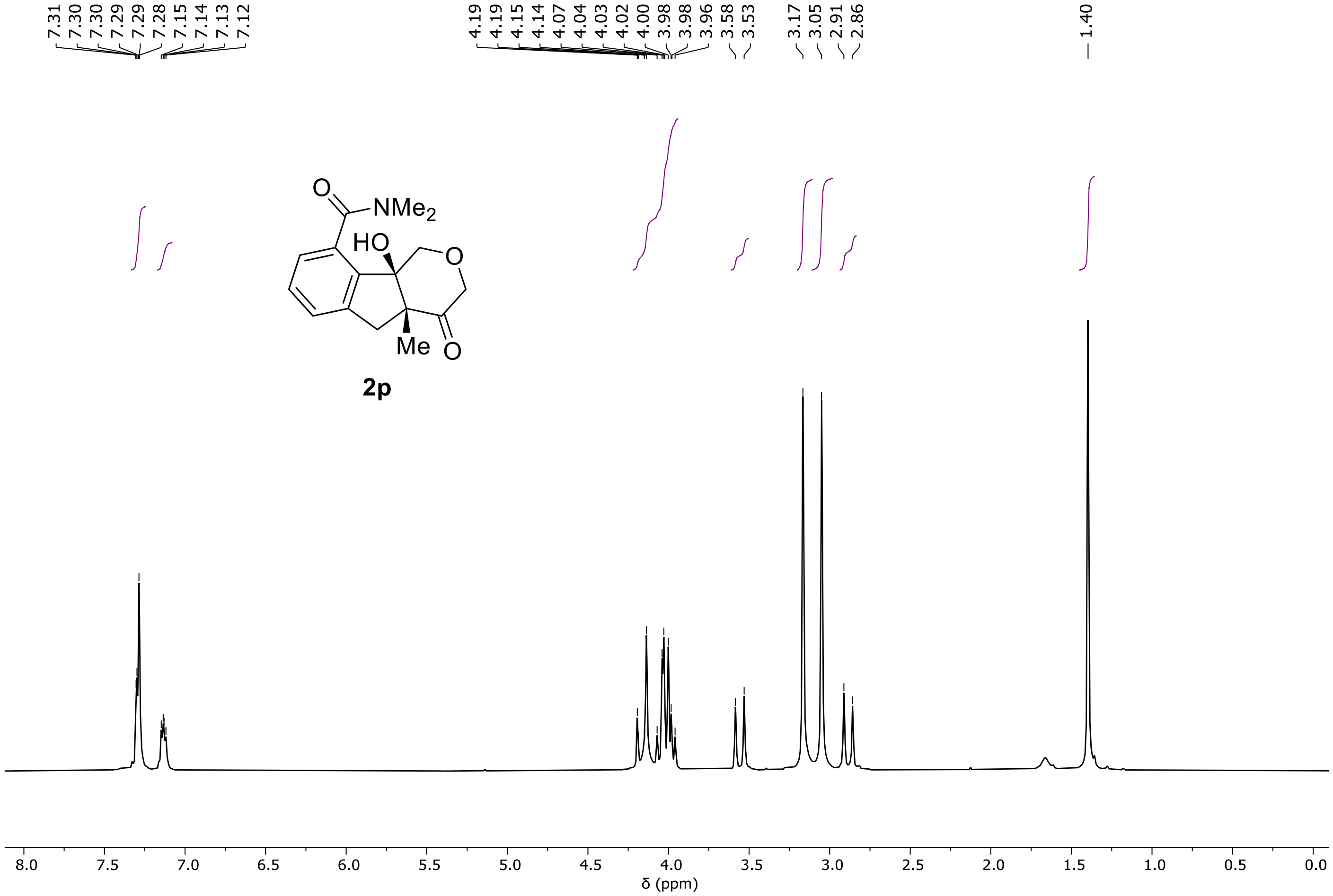

DEPT-135 NMR (75 MHz, CDCl3)

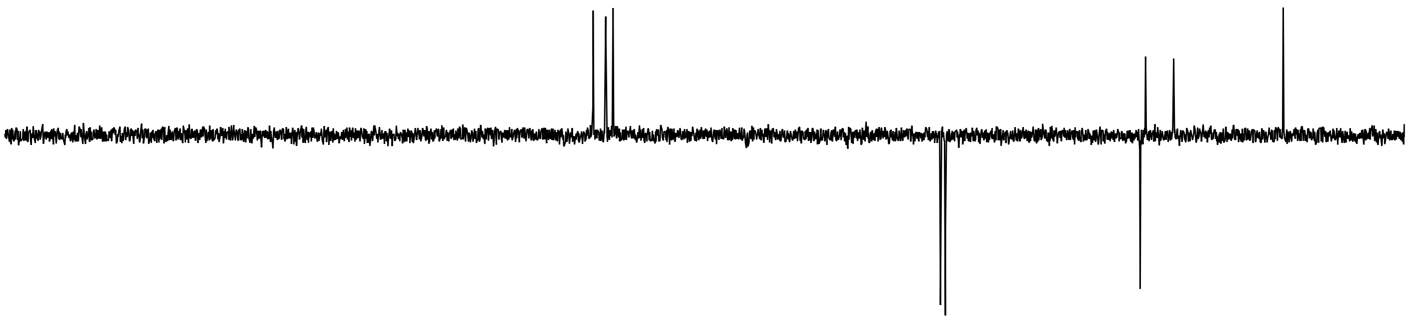

13C NMR (75 MHz, CDCl3)

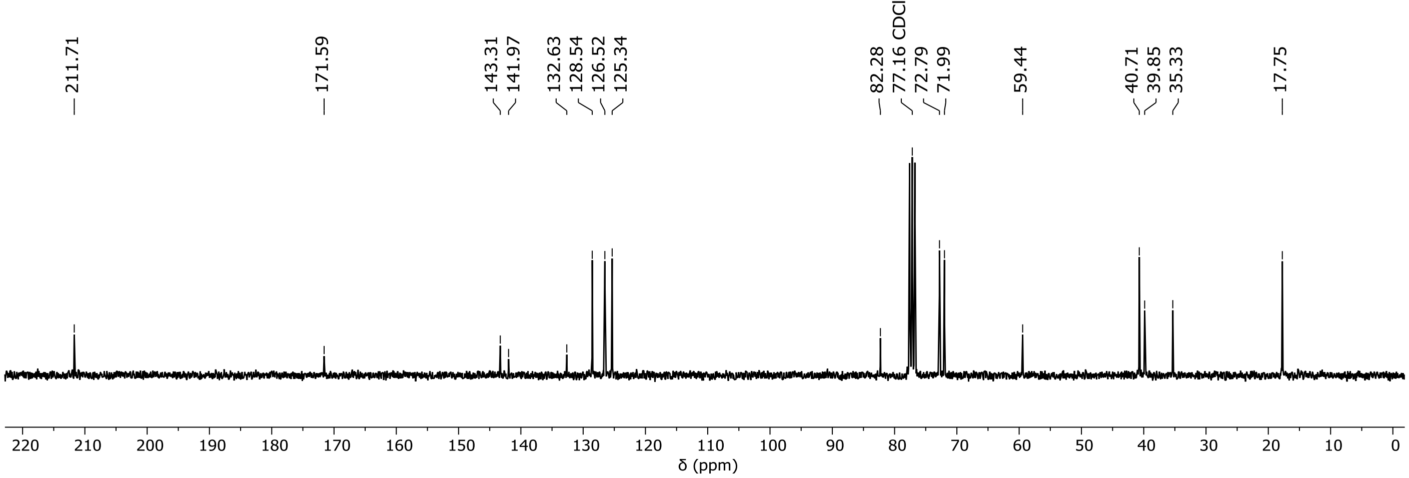

1H NMR (300 MHz, CDCl3)

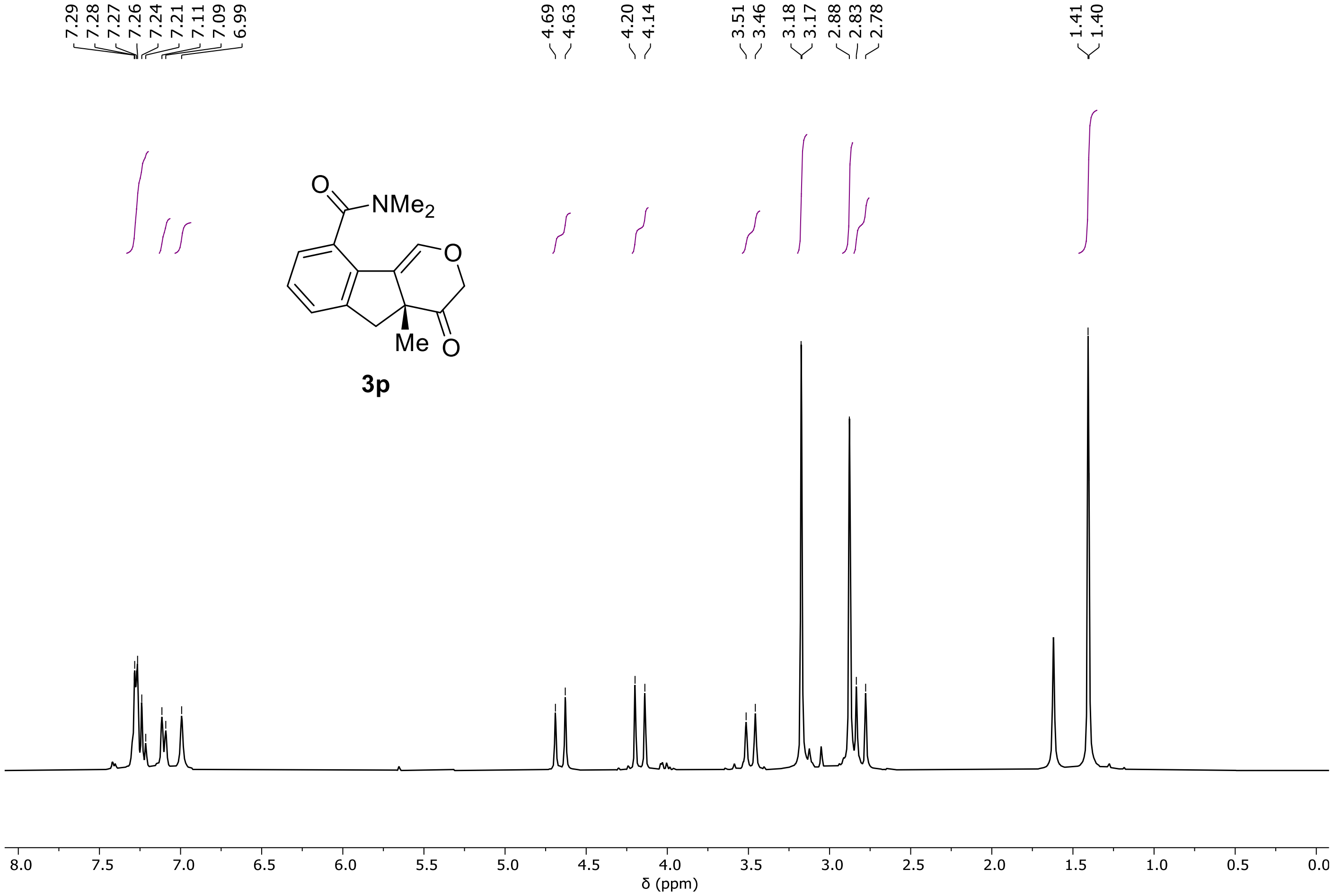

3p

DEPT-135 NMR (75 MHz, CDCl3)

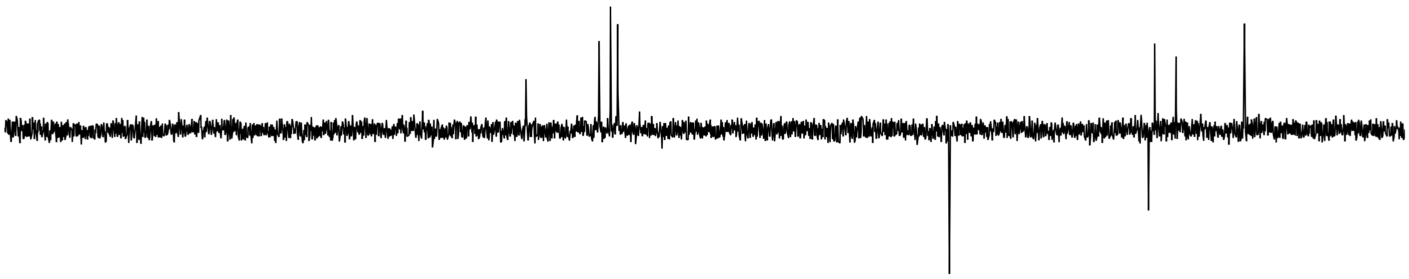

13C NMR (75 MHz, CDCl3)

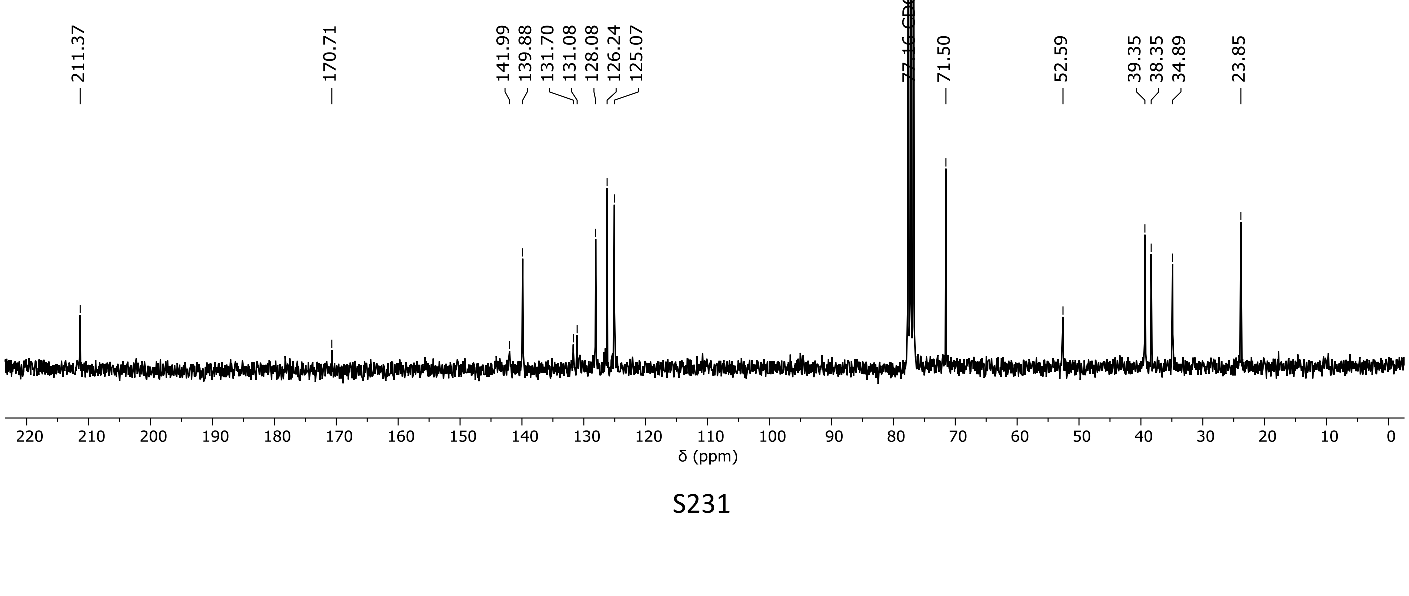

1H NMR (500 MHz, CDCl3)

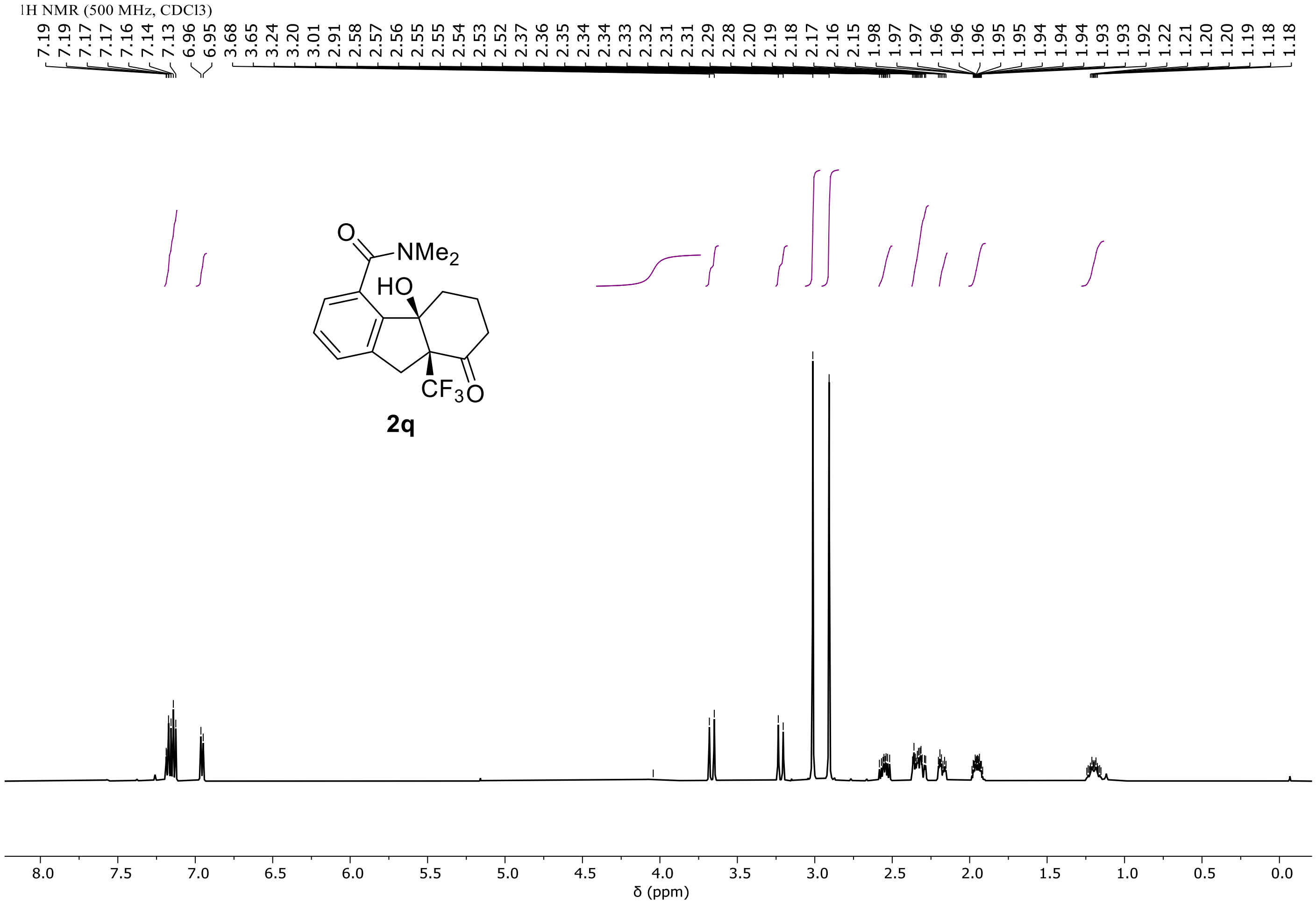

DEPT-135 NMR (126 MHz, CDCl3)

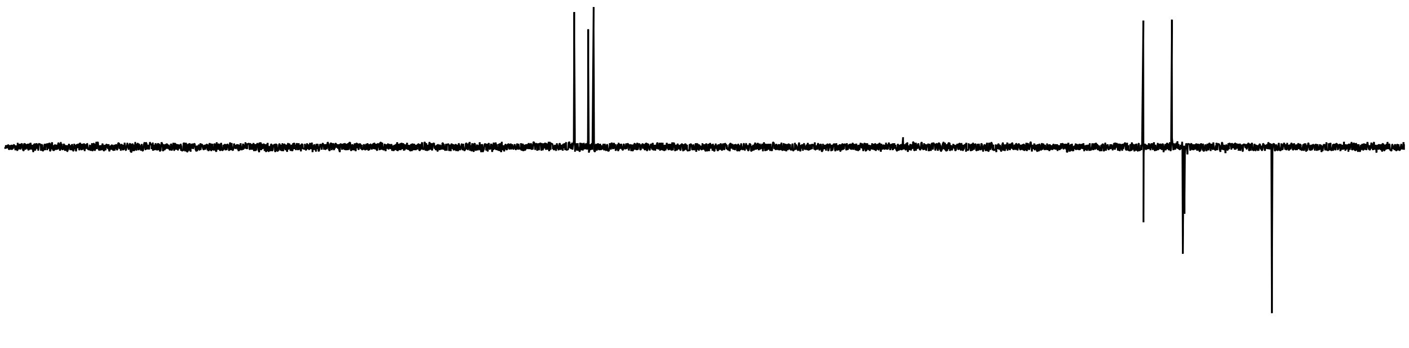

13C NMR (126 MHz, CDCl3)

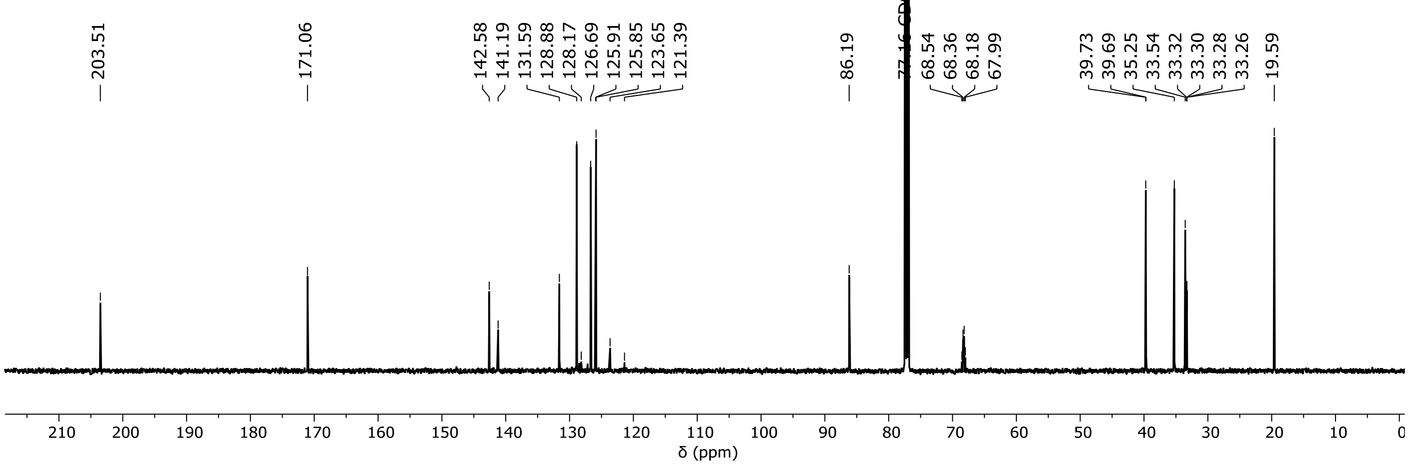

1H NMR (500 MHz, CDCl3)

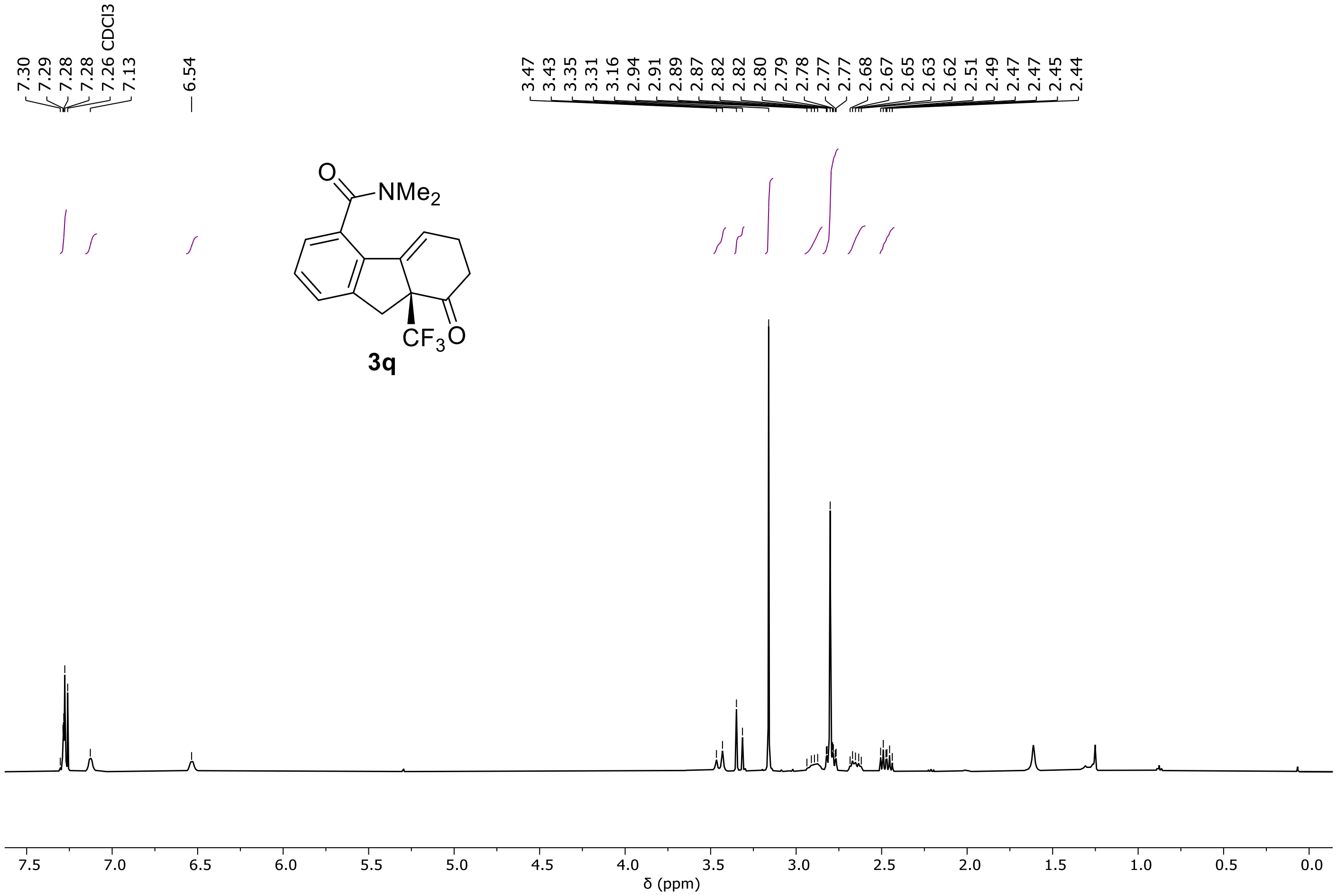

DEPT-135 NMR (126 MHz, CDCl3)

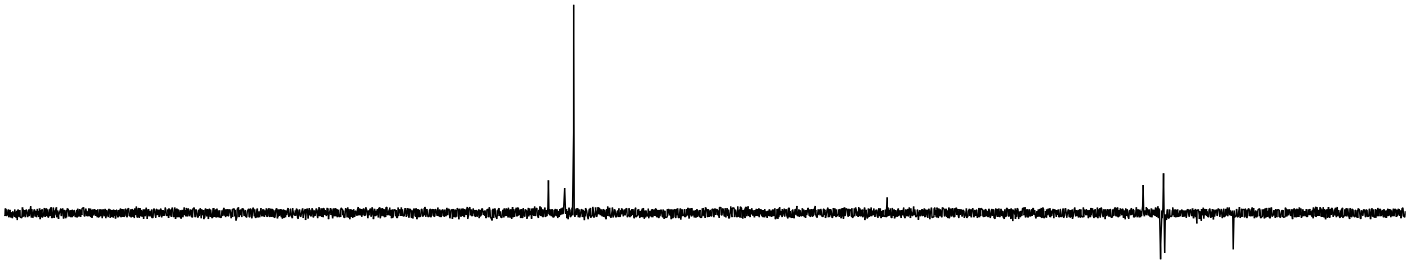

13C NMR (126 MHz, CDCl3)

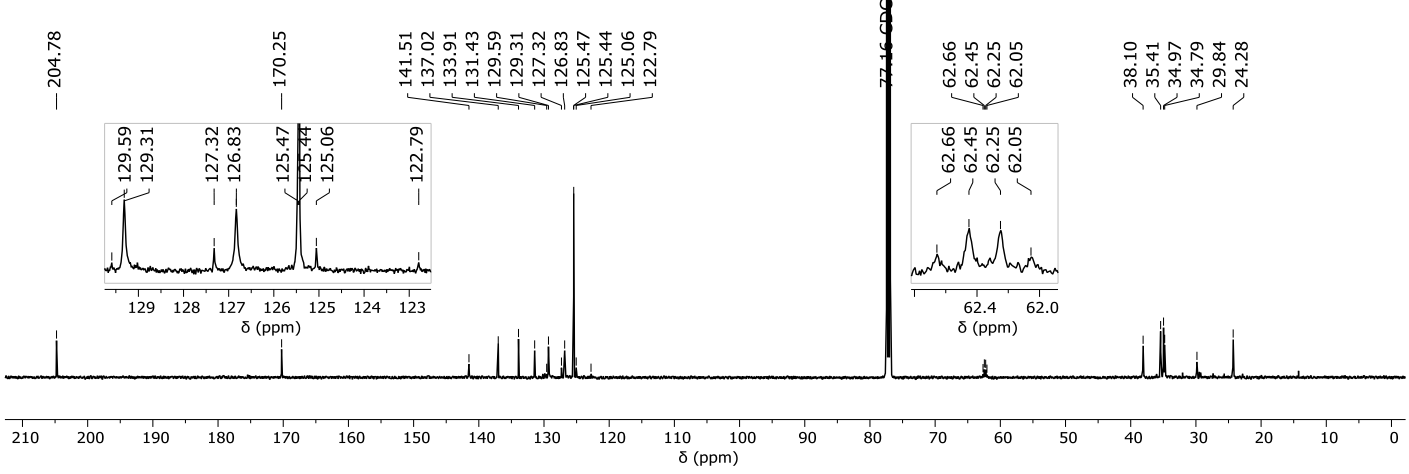

1H NMR (500 MHz, CDCl3)

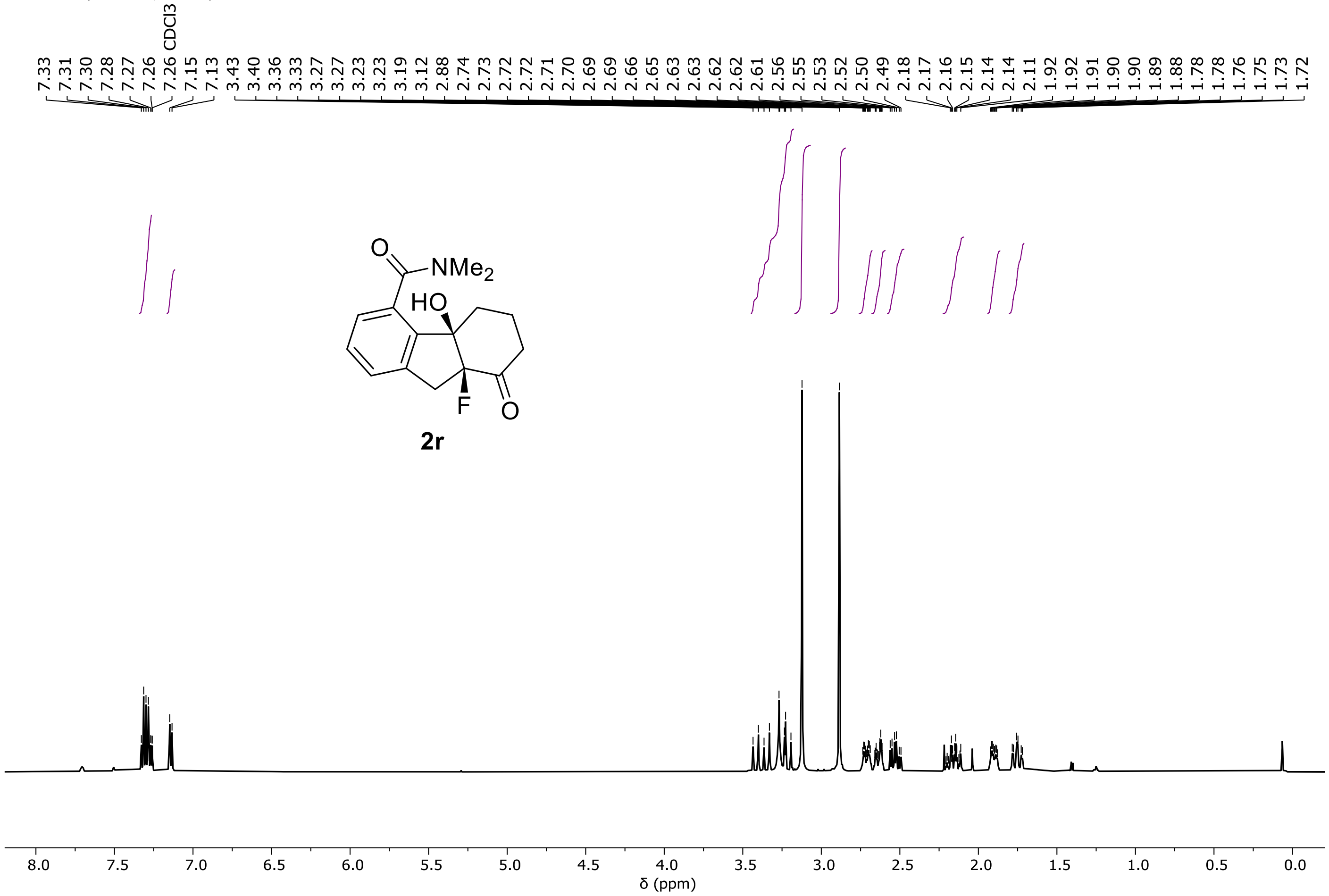

DEPT-135 NMR (126 MHz, CDCl3)

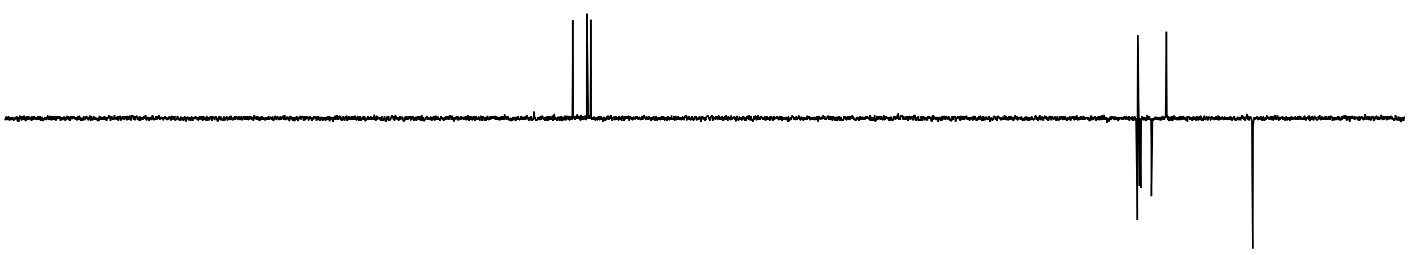

13C NMR (126 MHz, CDCl3)

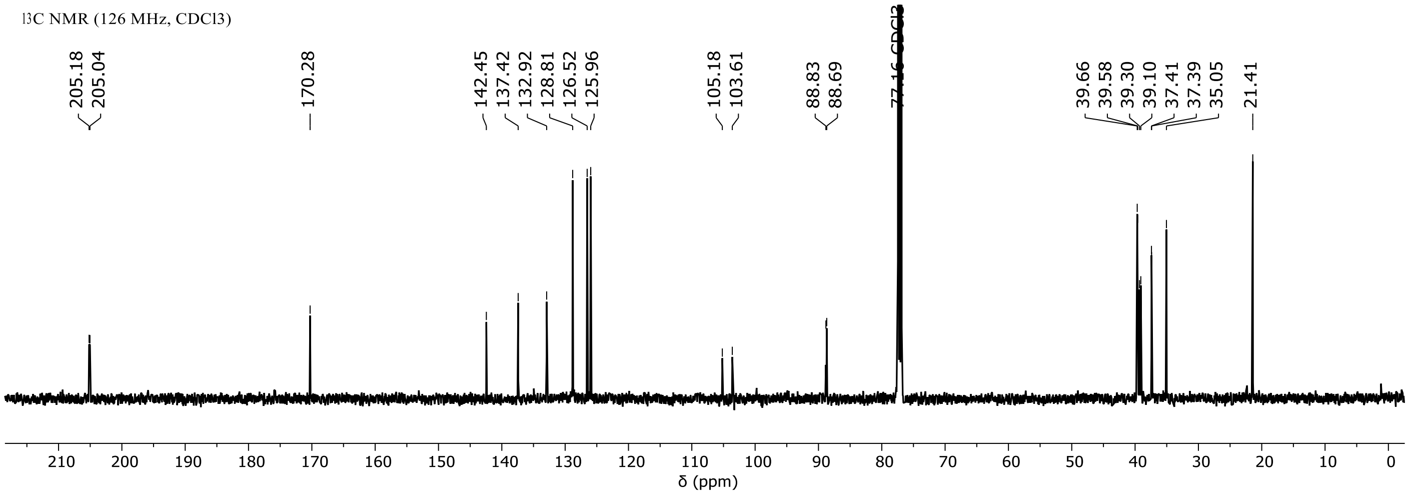

1H NMR (500 MHz, CDCl3)

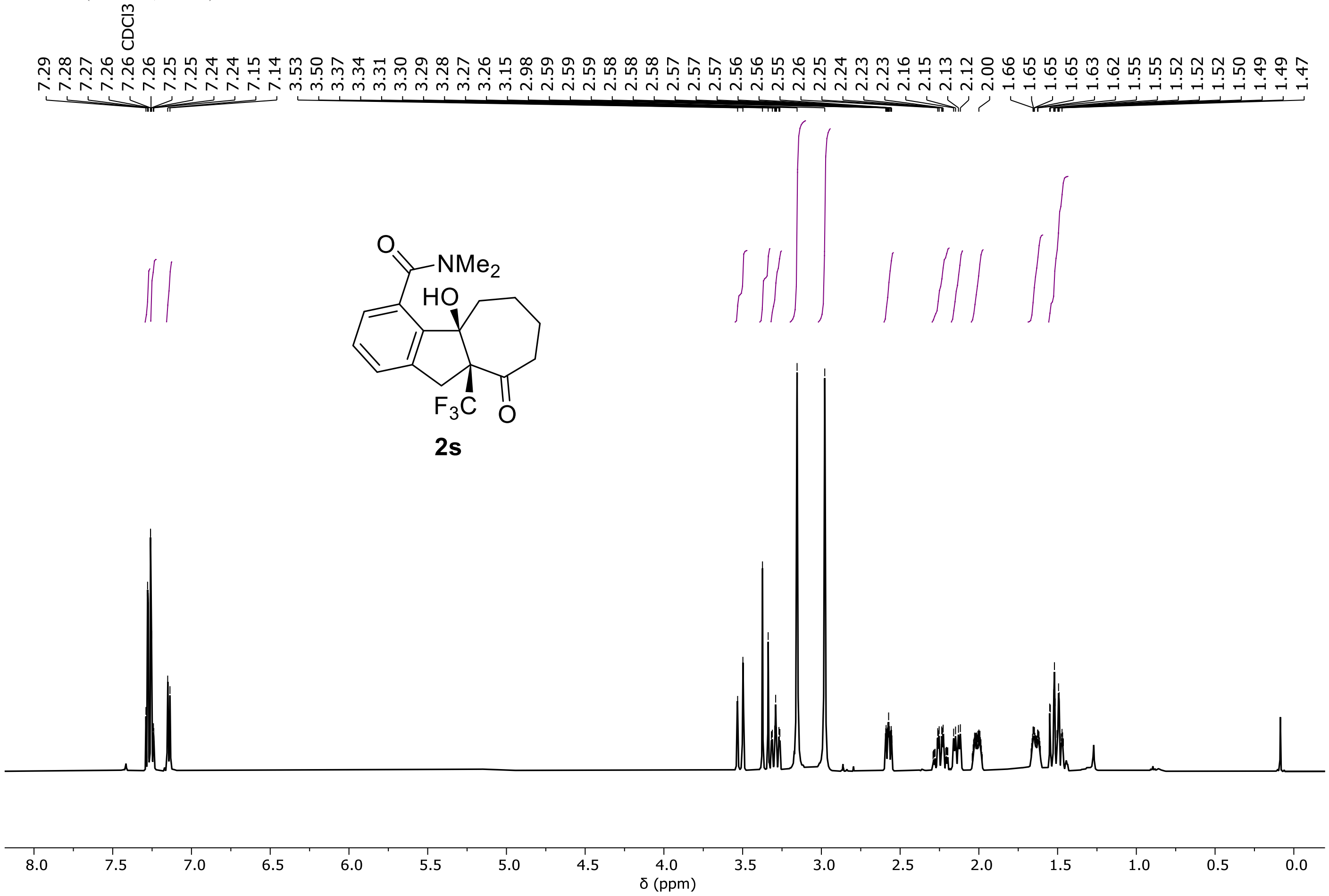

DEPT-135 NMR (126 MHz, CDCl3)

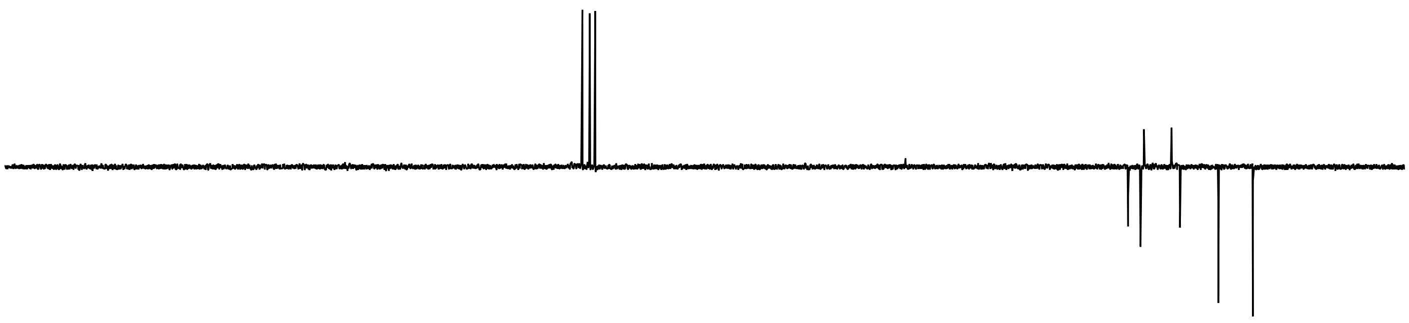

13C NMR (126 MHz, CDCl3)

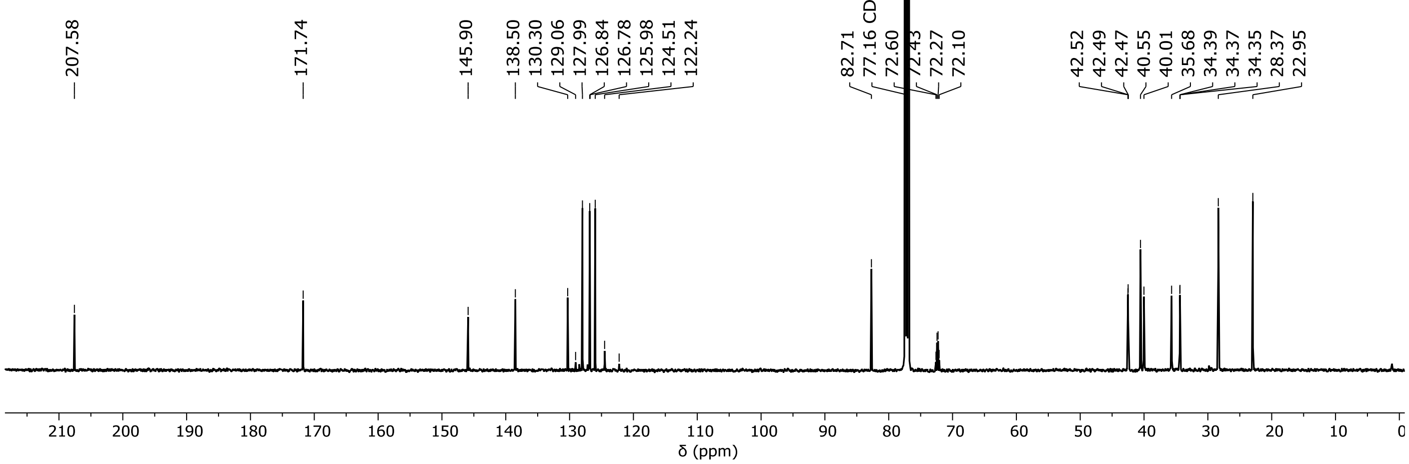

1H NMR (500 MHz, CDCl3)

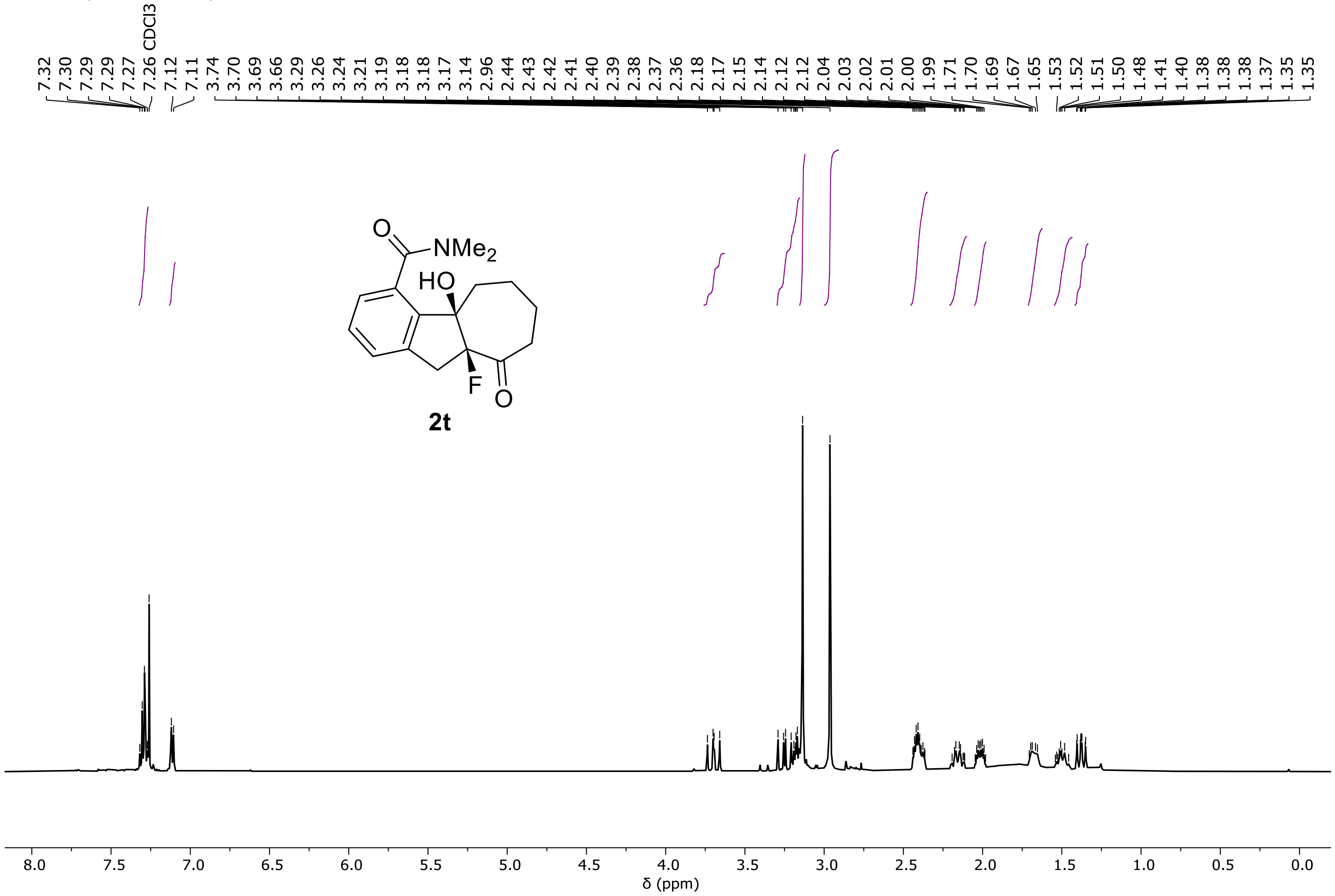

DEPT-135 NMR (126 MHz, CDCl3)

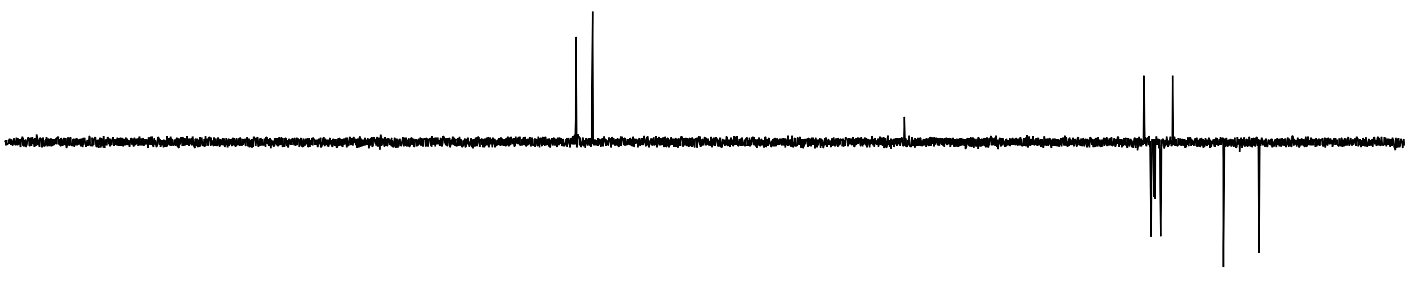

13C NMR (126 MHz, CDCl3)

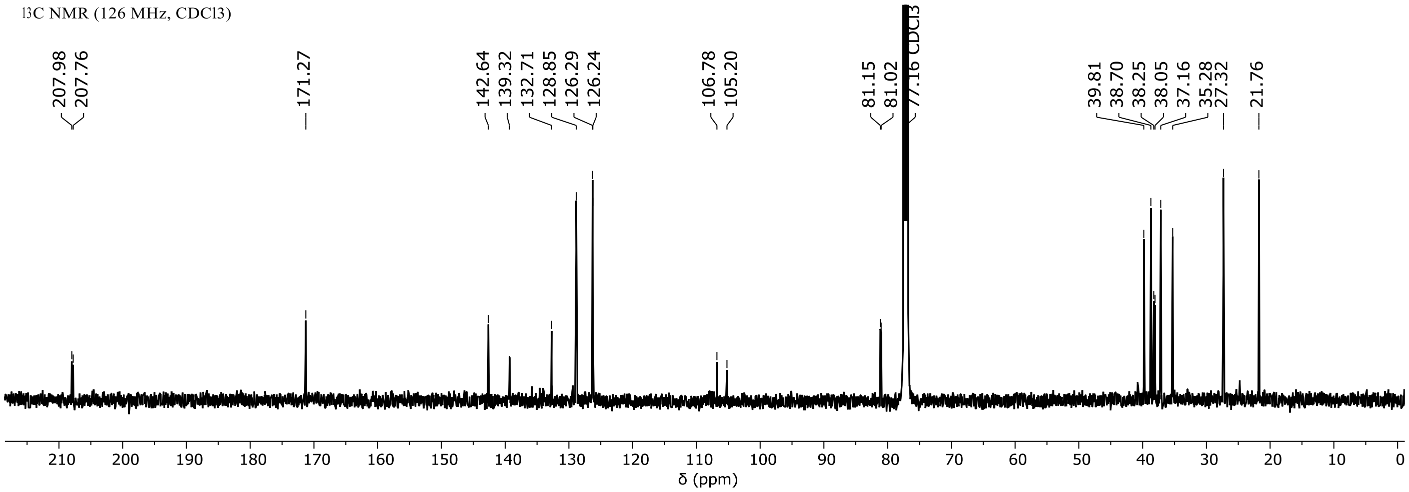

1H NMR (500 MHz, CDCl3)

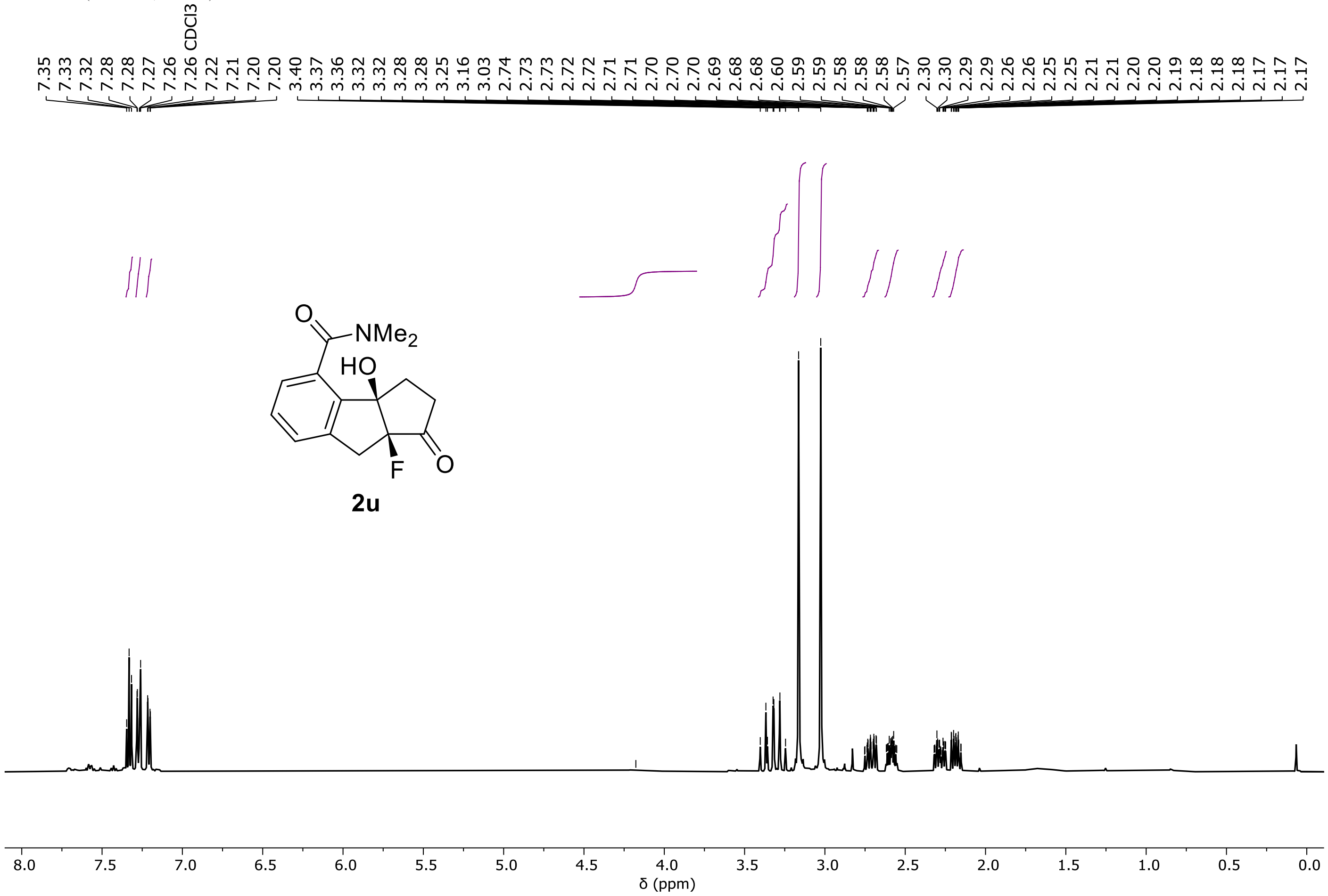

DEPT-135 NMR (126 MHz, CDCl3)

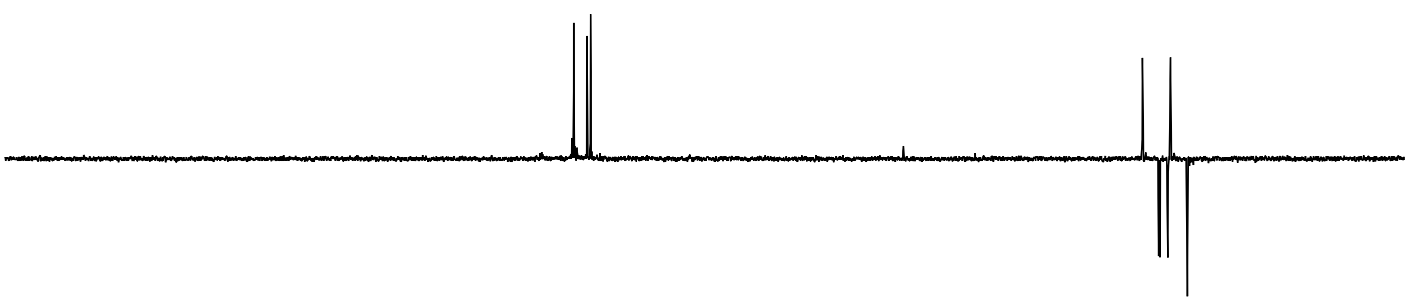

13C NMR (126 MHz, CDCl3)

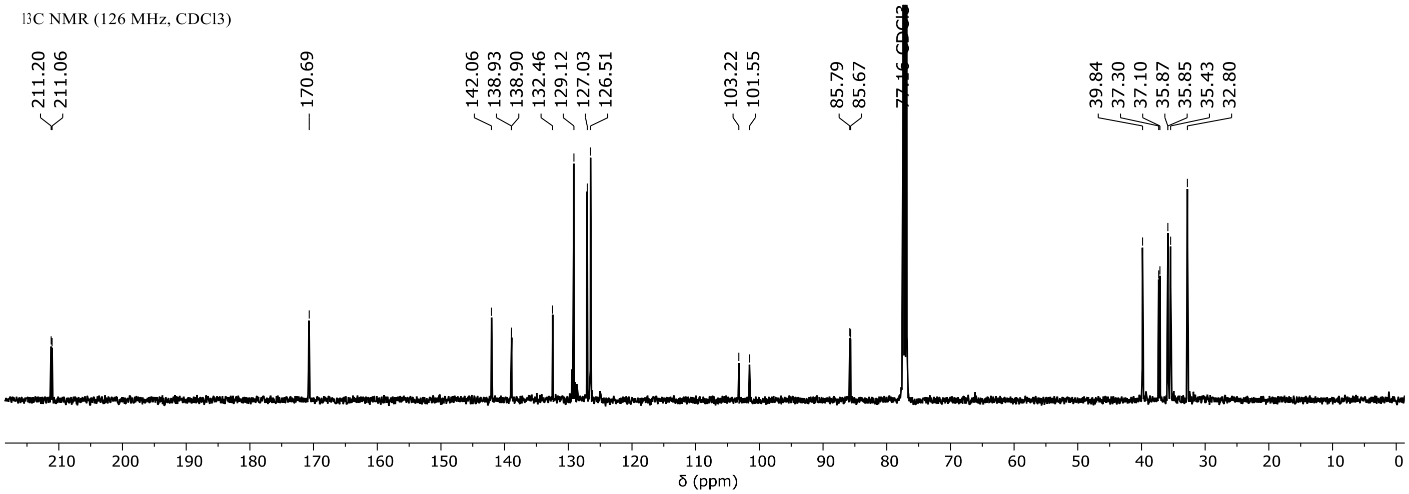

1H NMR (300 MHz, CDCl3)

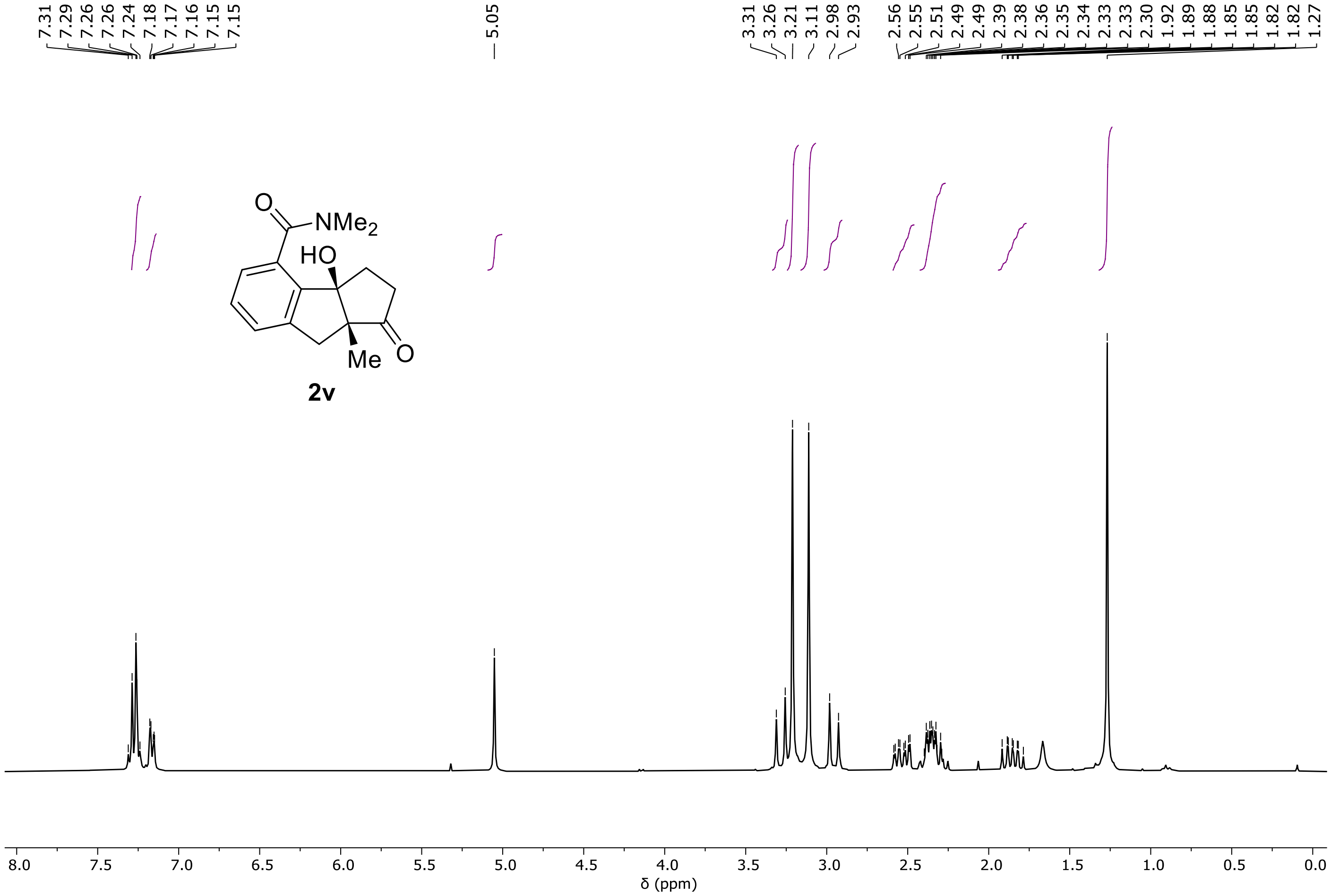

DEPT-135 NMR (75 MHz, CDCl3)

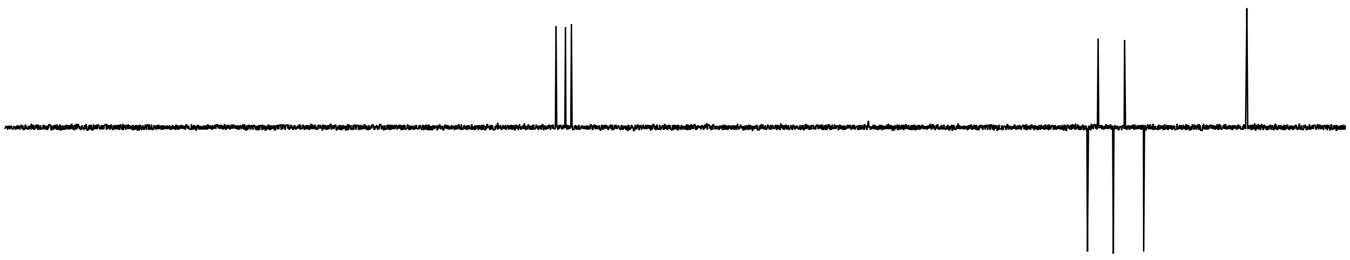

13C NMR (75 MHz, CDCl3)

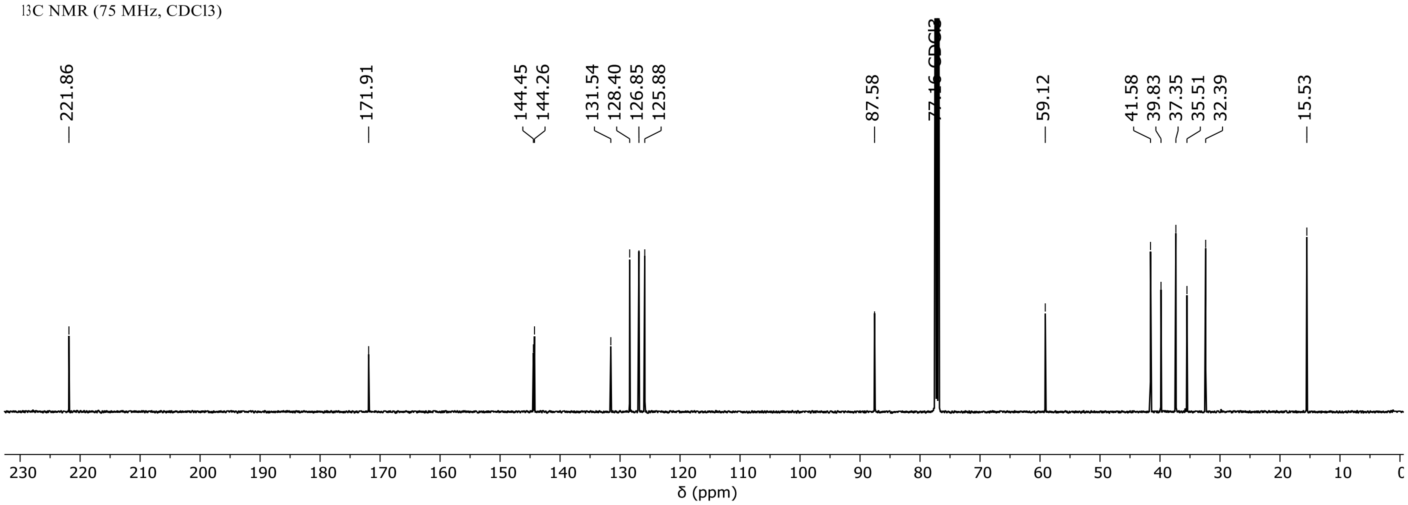

1H NMR (500 MHz, CDCl3)

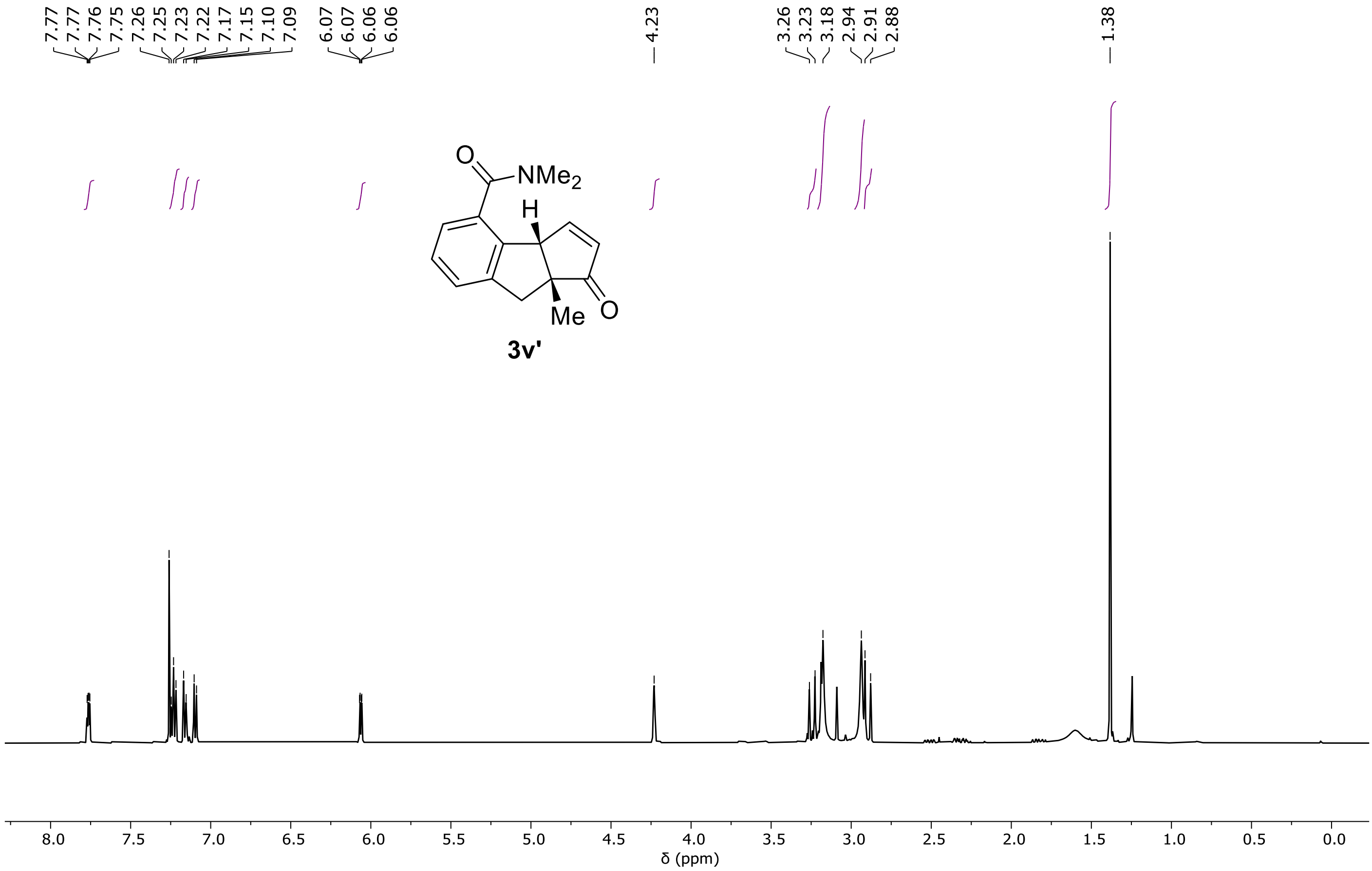

DEPT-135 NMR (126 MHz, CDCl3)

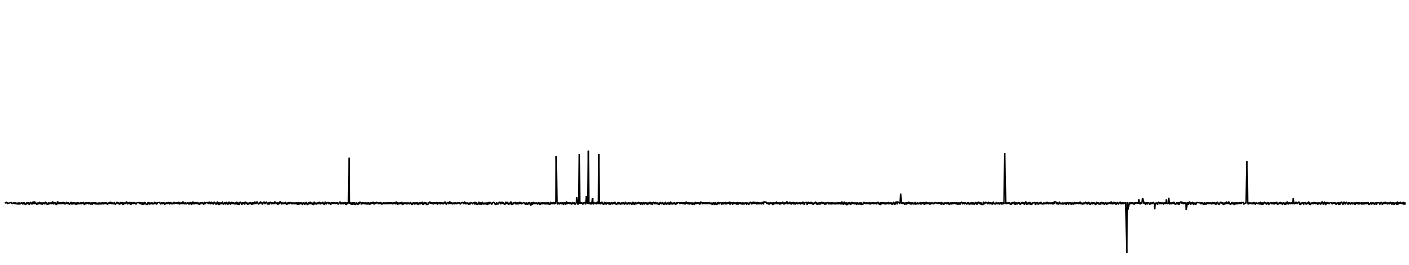

13C NMR (126 MHz, CDCl3)

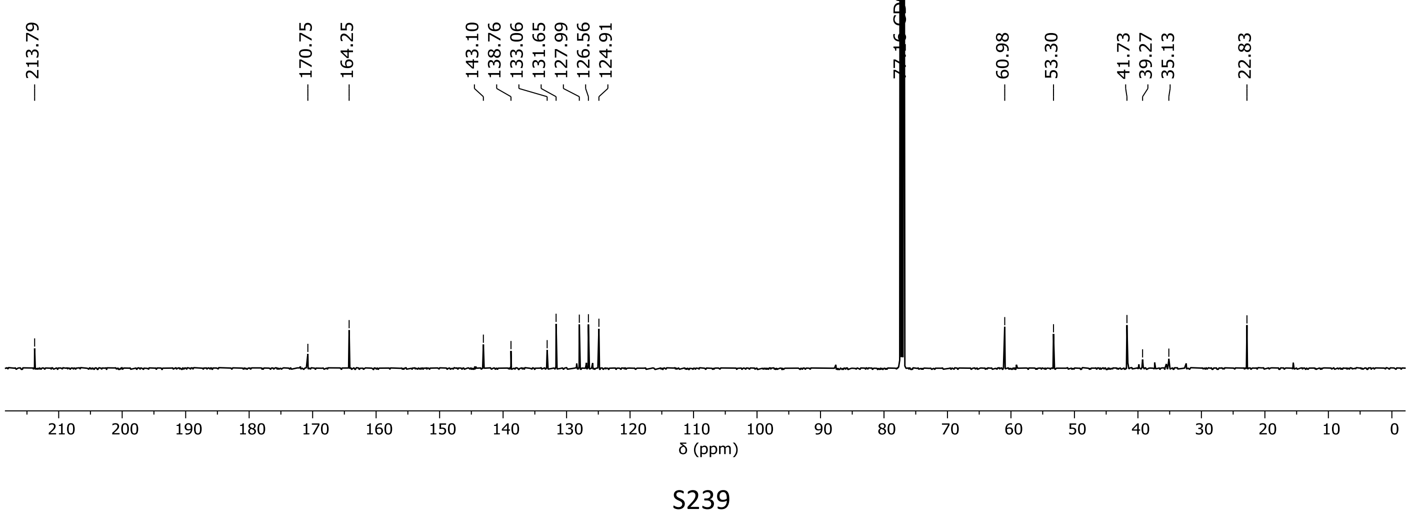

NOE experiment (500 MHz, CDCl3). Irradiation: CH3

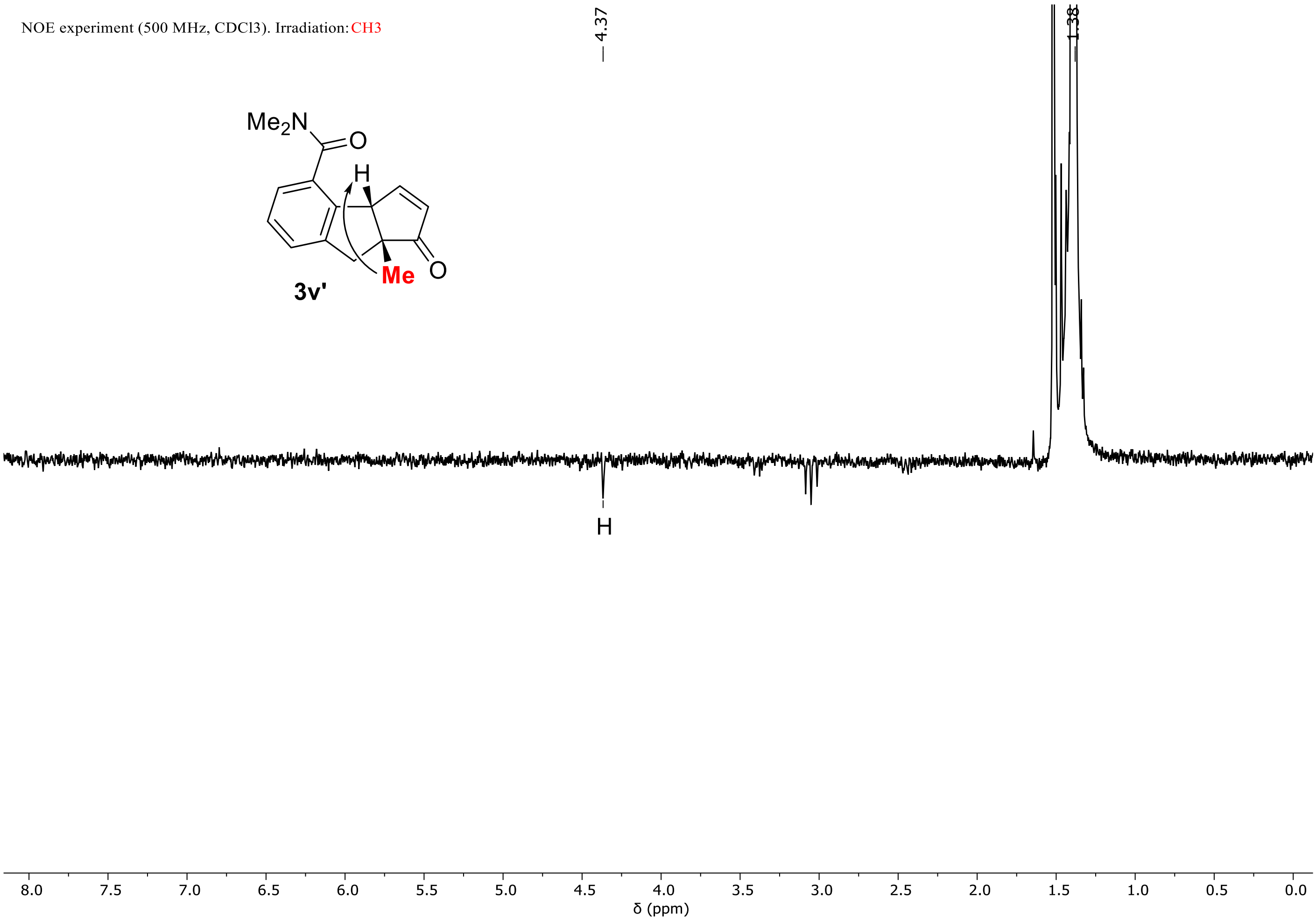

NOE experiment (500 MHz, CDCl3). Irradiation: CH

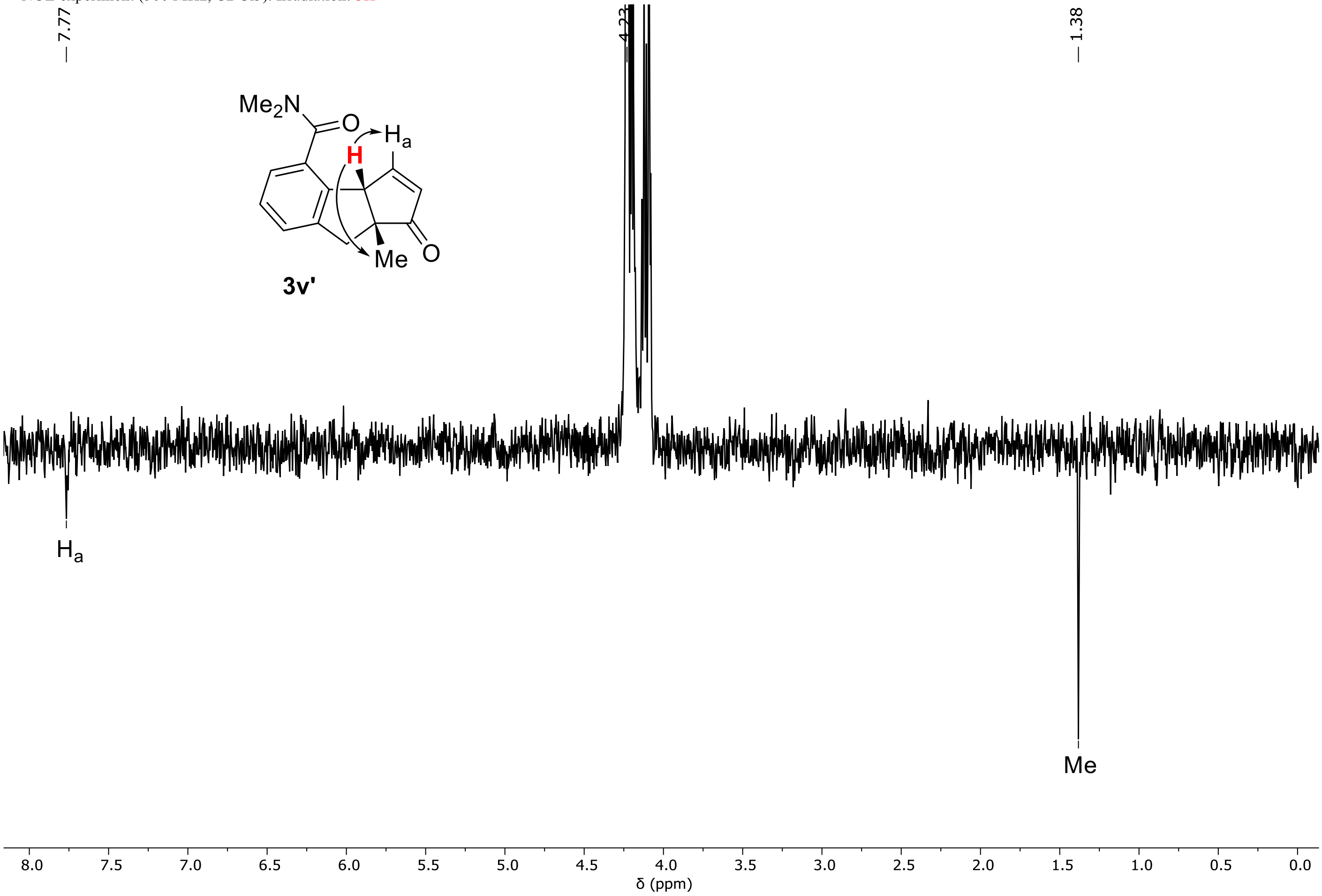

1H NMR (300 MHz, CDCl3)

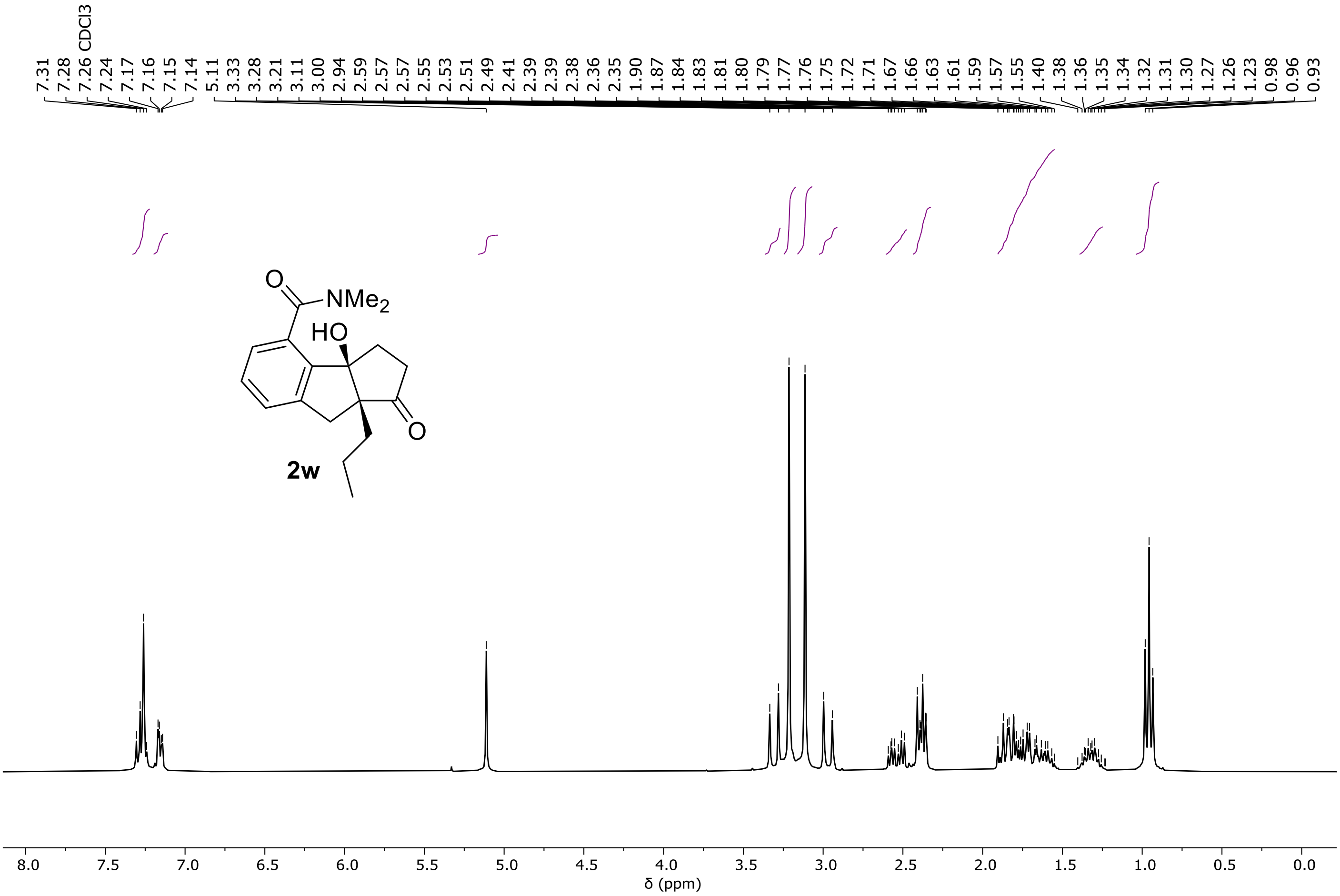

DEPT-135 NMR (75 MHz, CDCl3)

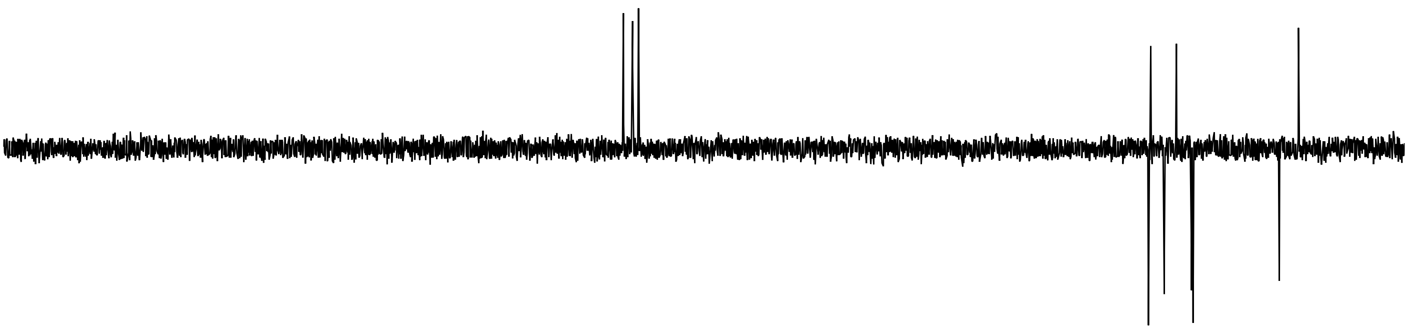

13C NMR (75 MHz, CDCl3)

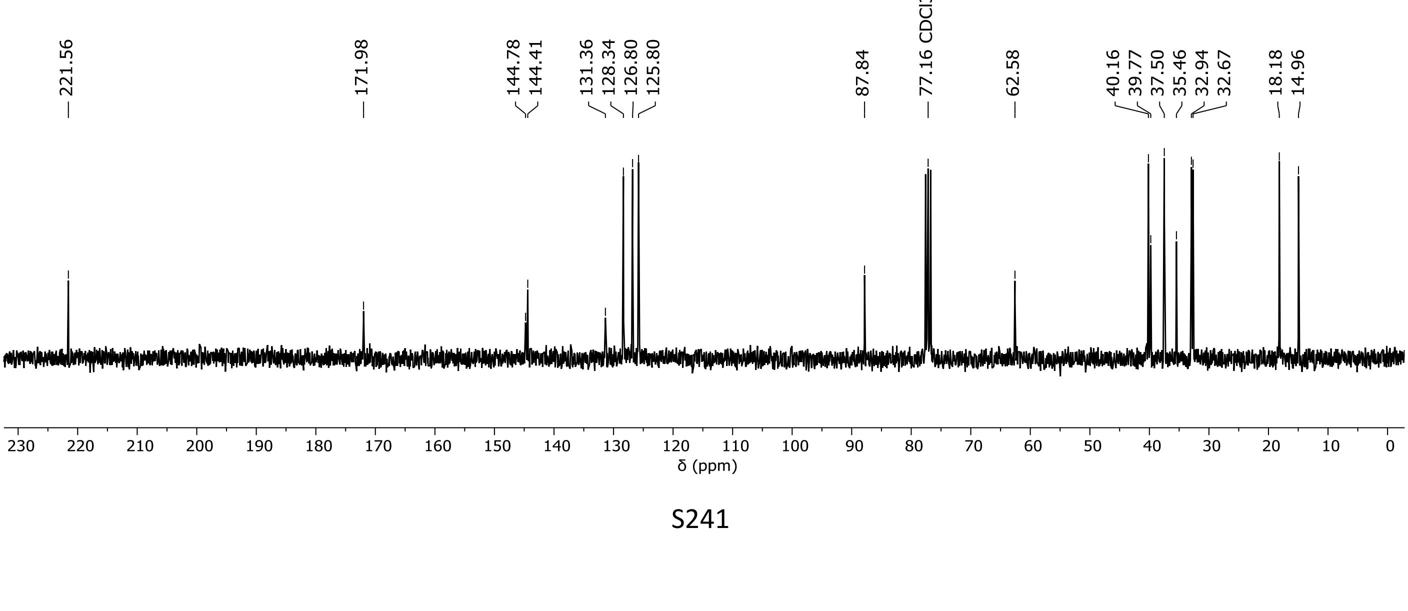

1H NMR (500 MHz, CDCl3)

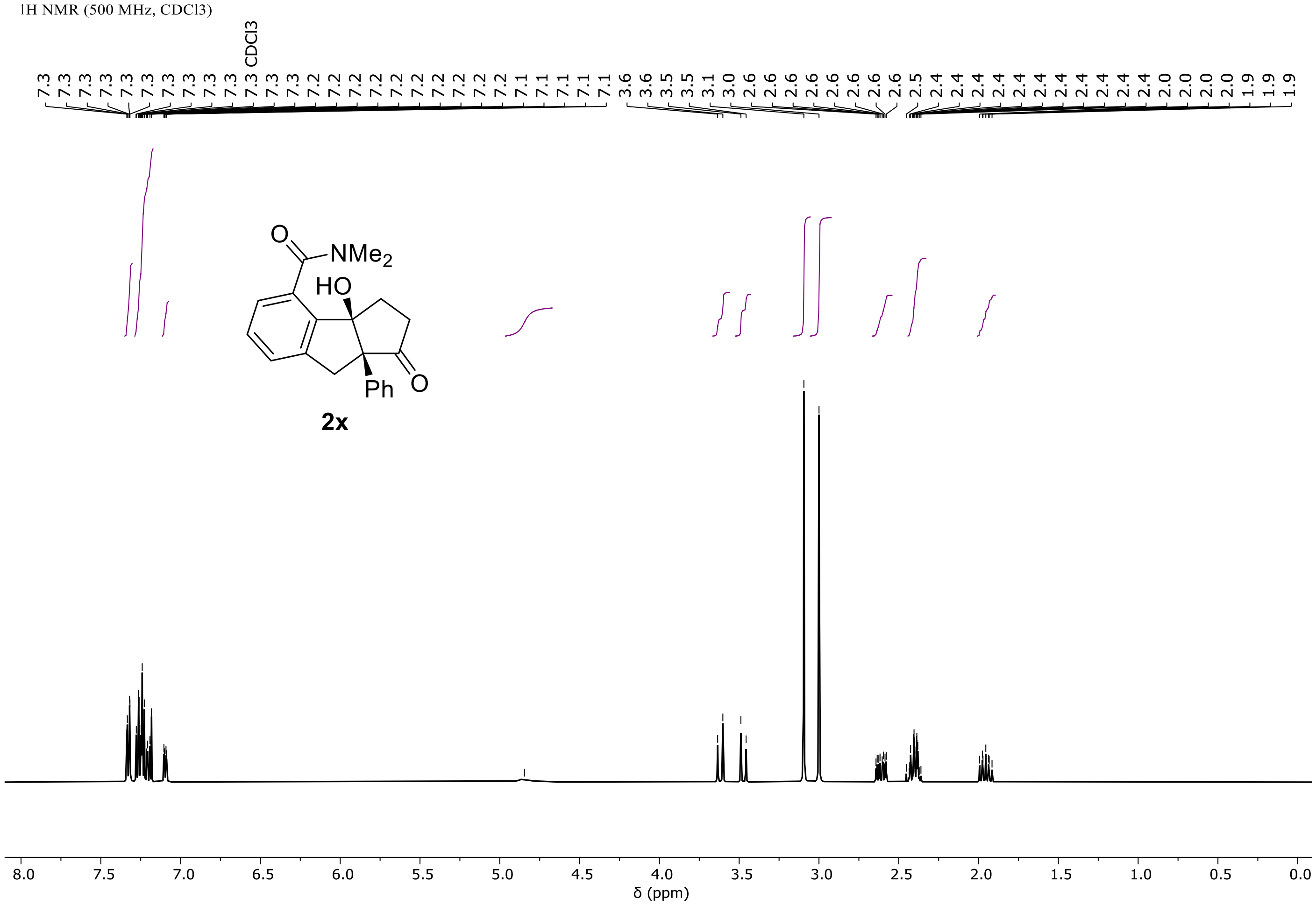

DEPT-135 NMR (126 MHz, CDCl3)

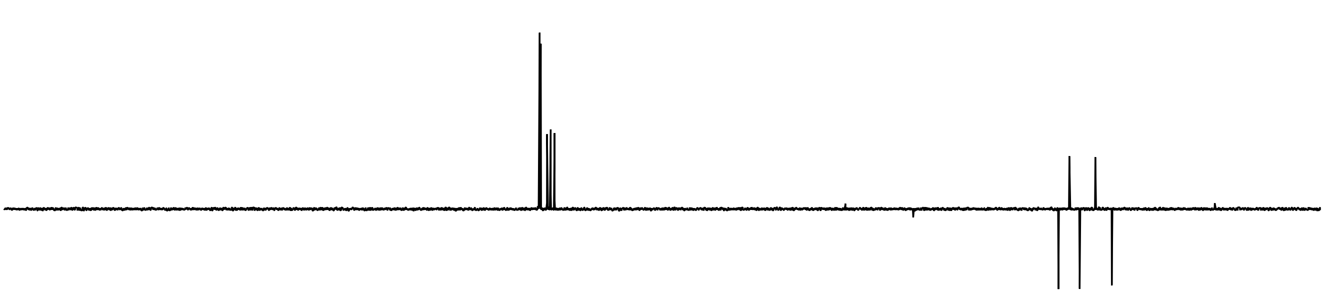

13C NMR (126 MHz, CDCl3)

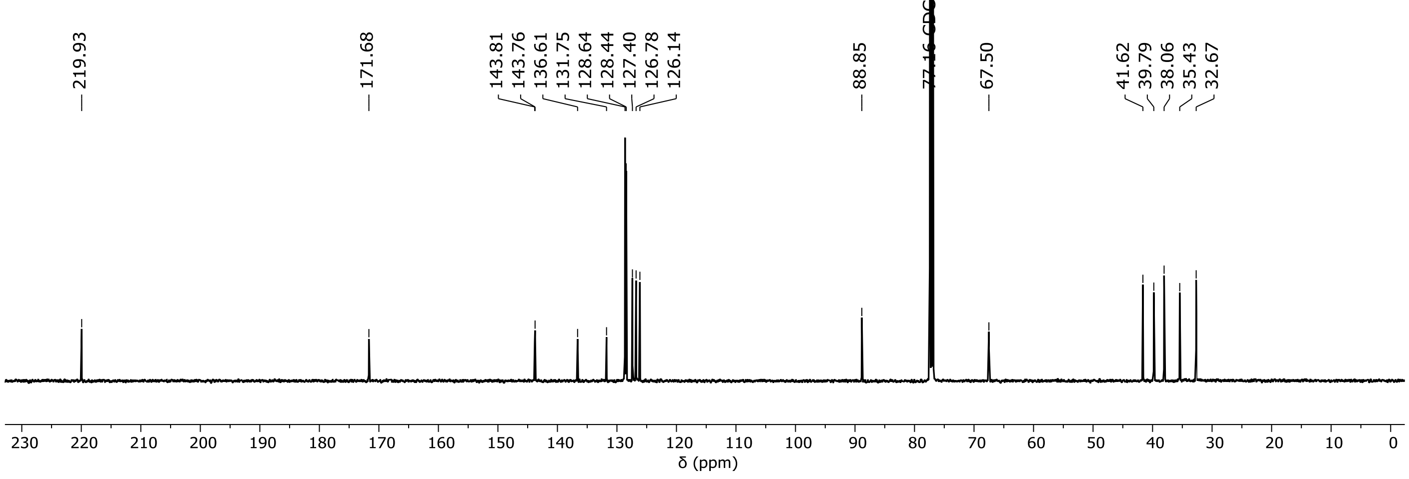

1H NMR (300 MHz, CDCl3)

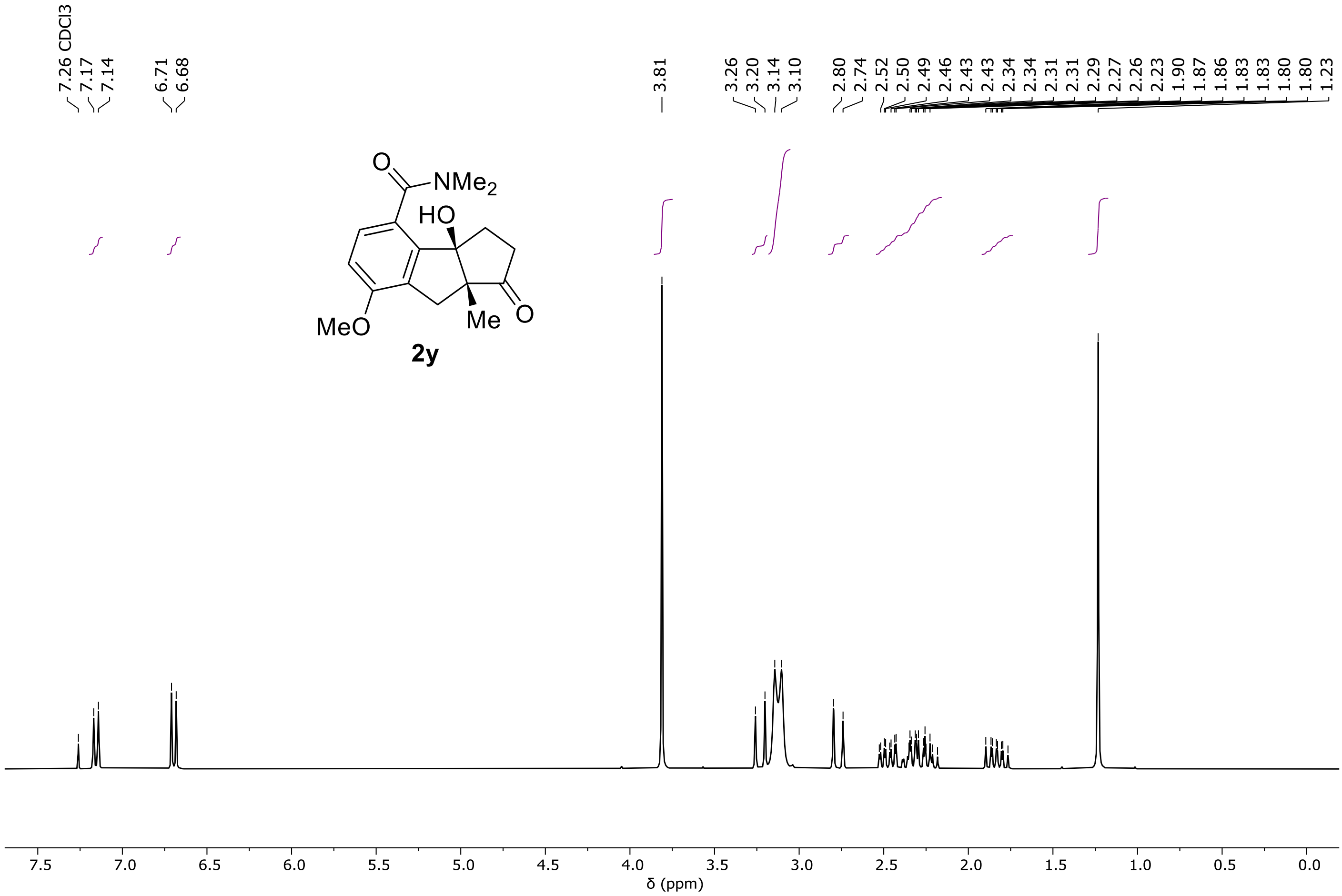

DEPT-135 NMR (75 MHz, CDCl3)

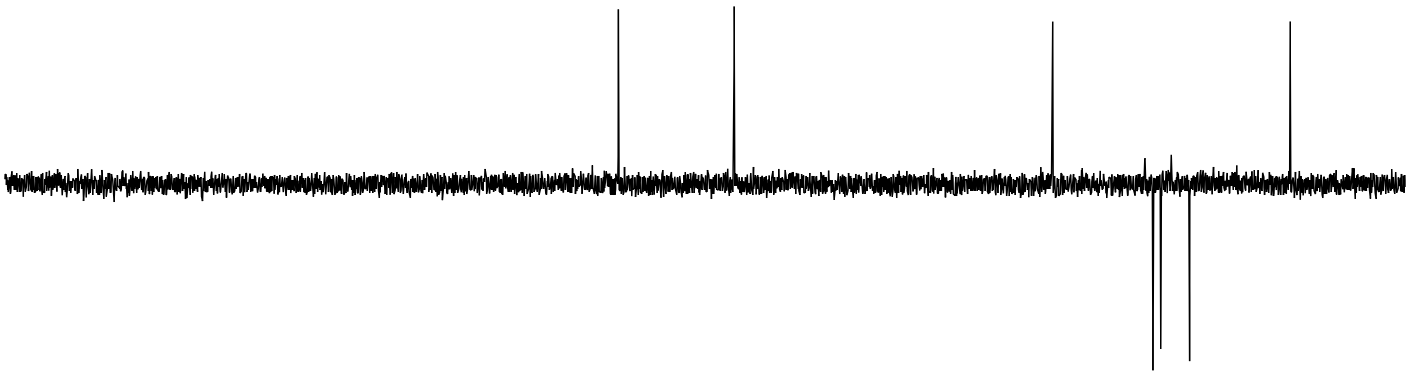

13C NMR (75 MHz, CDCl3)

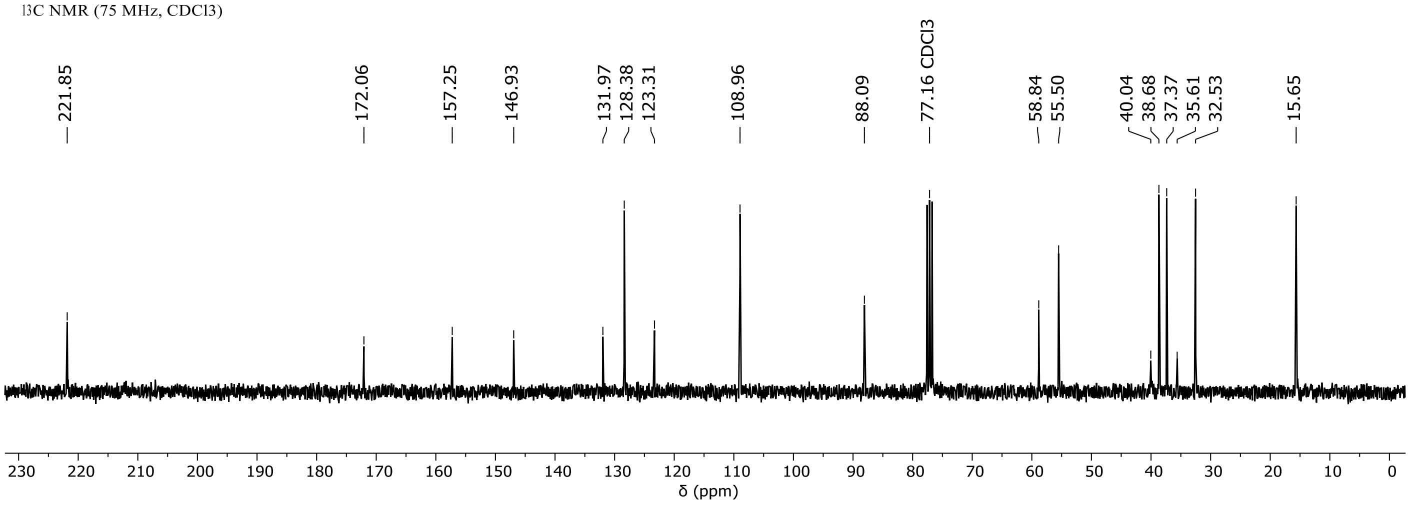

1H NMR (300 MHz, CDCl3)

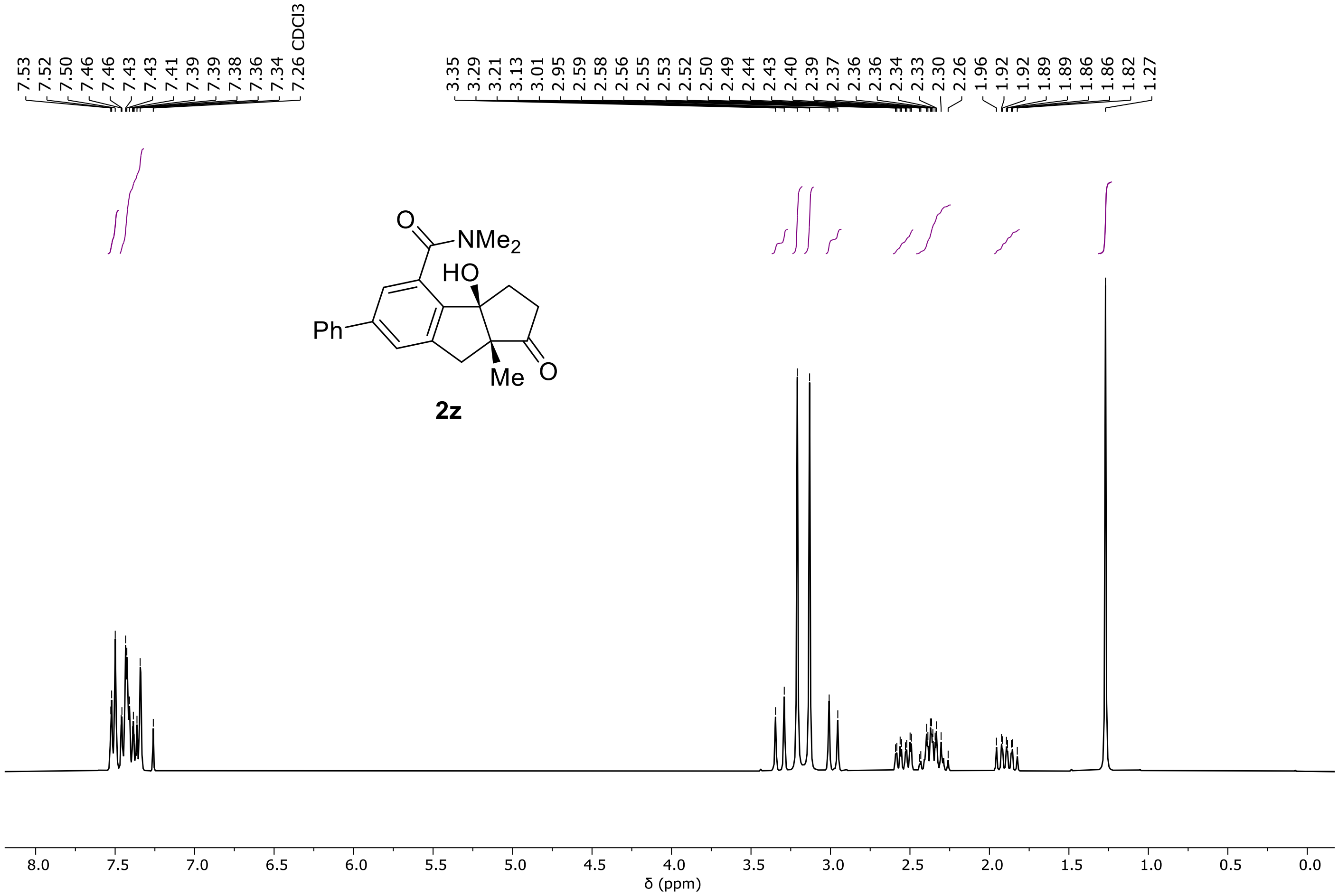

DEPT-135 NMR (75 MHz, CDCl3)

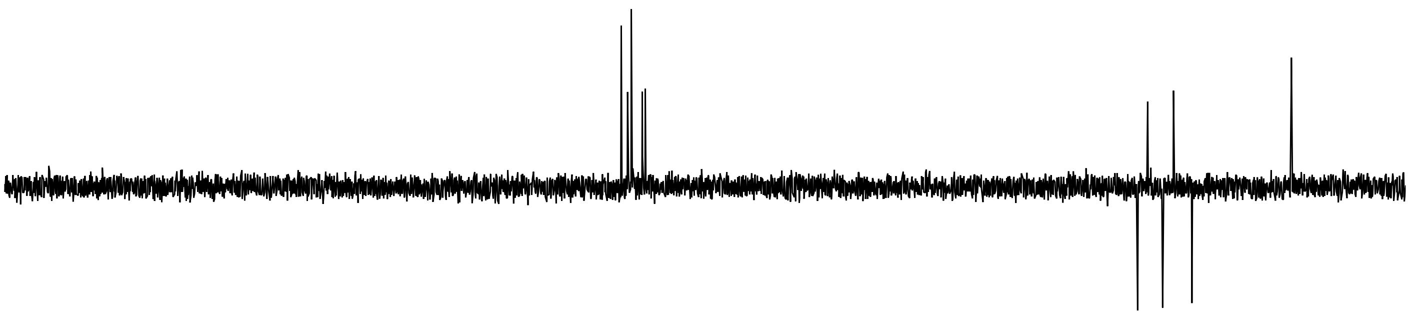

13C NMR (75 MHz, CDCl3)

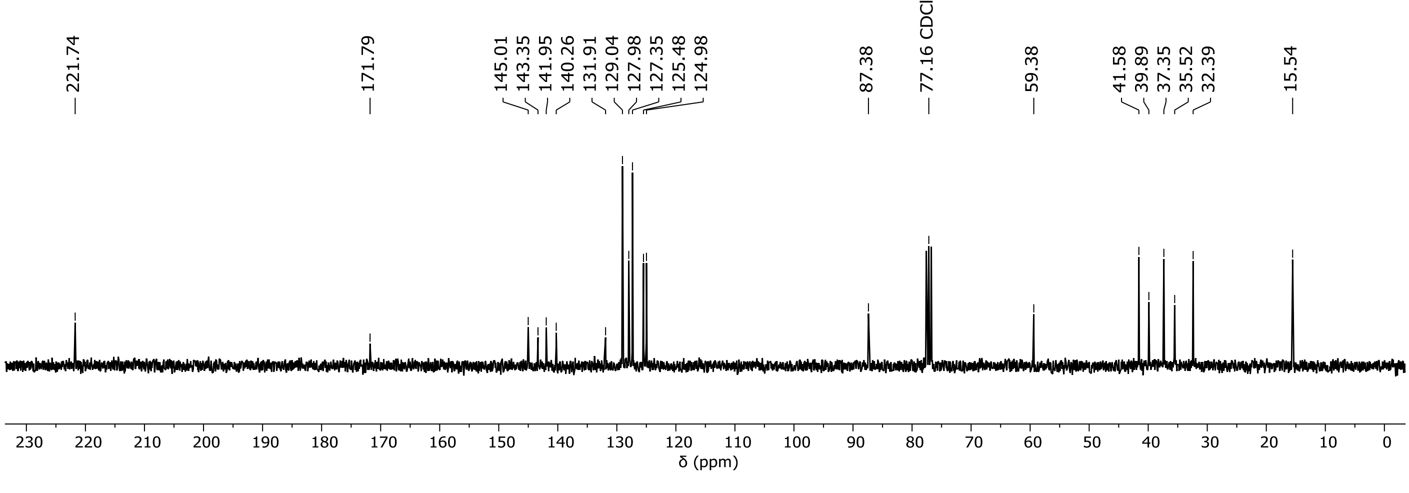

1H NMR (500 MHz, CDCl3)

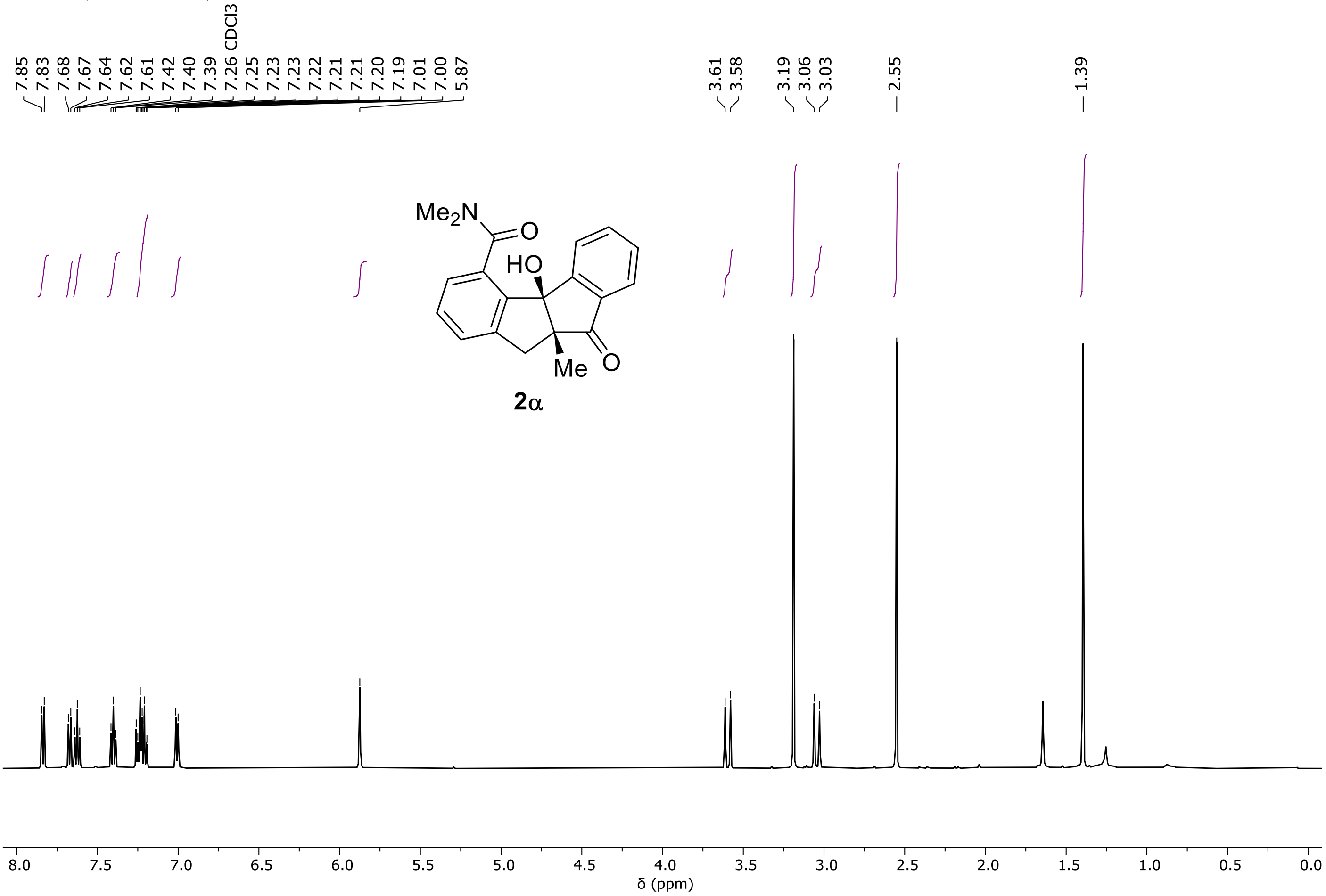

DEPT-135 NMR (126 MHz, CDCl3)

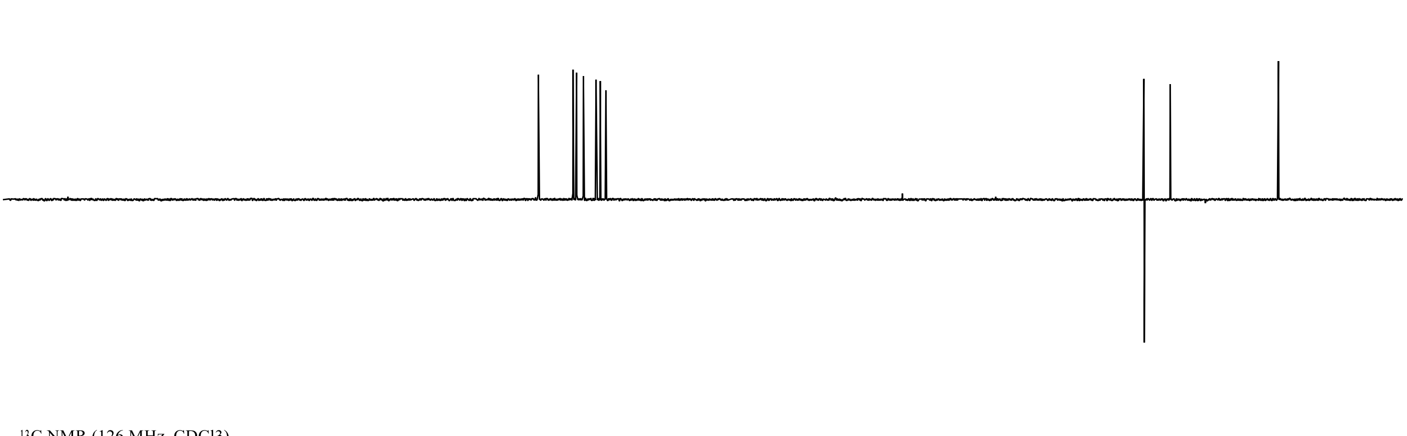

13C NMR (126 MHz, CDCl3)

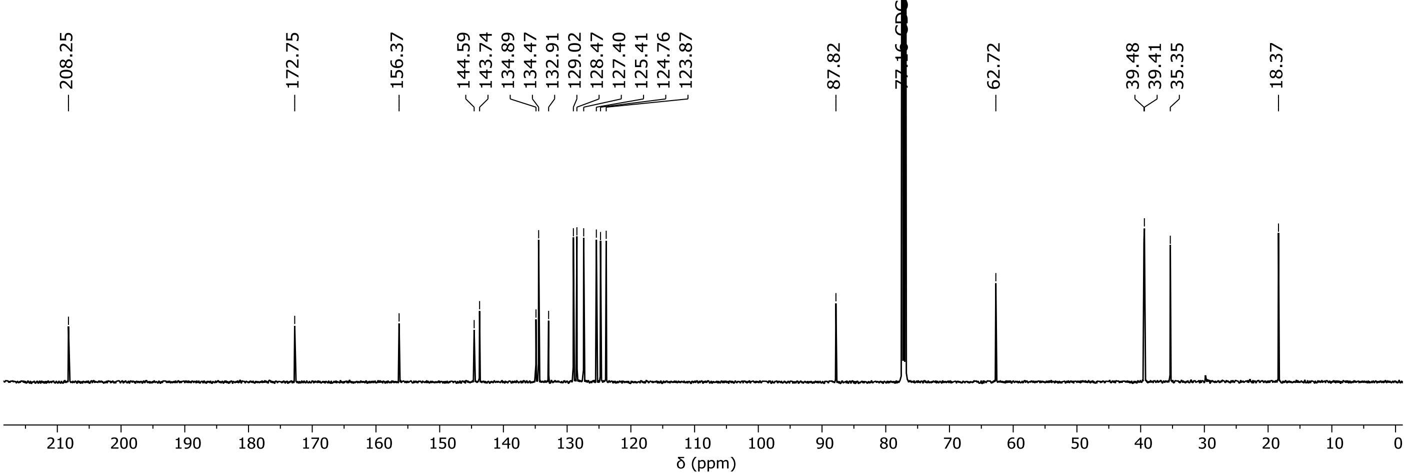

1H NMR (500 MHz, DMSO, 100 ºC)

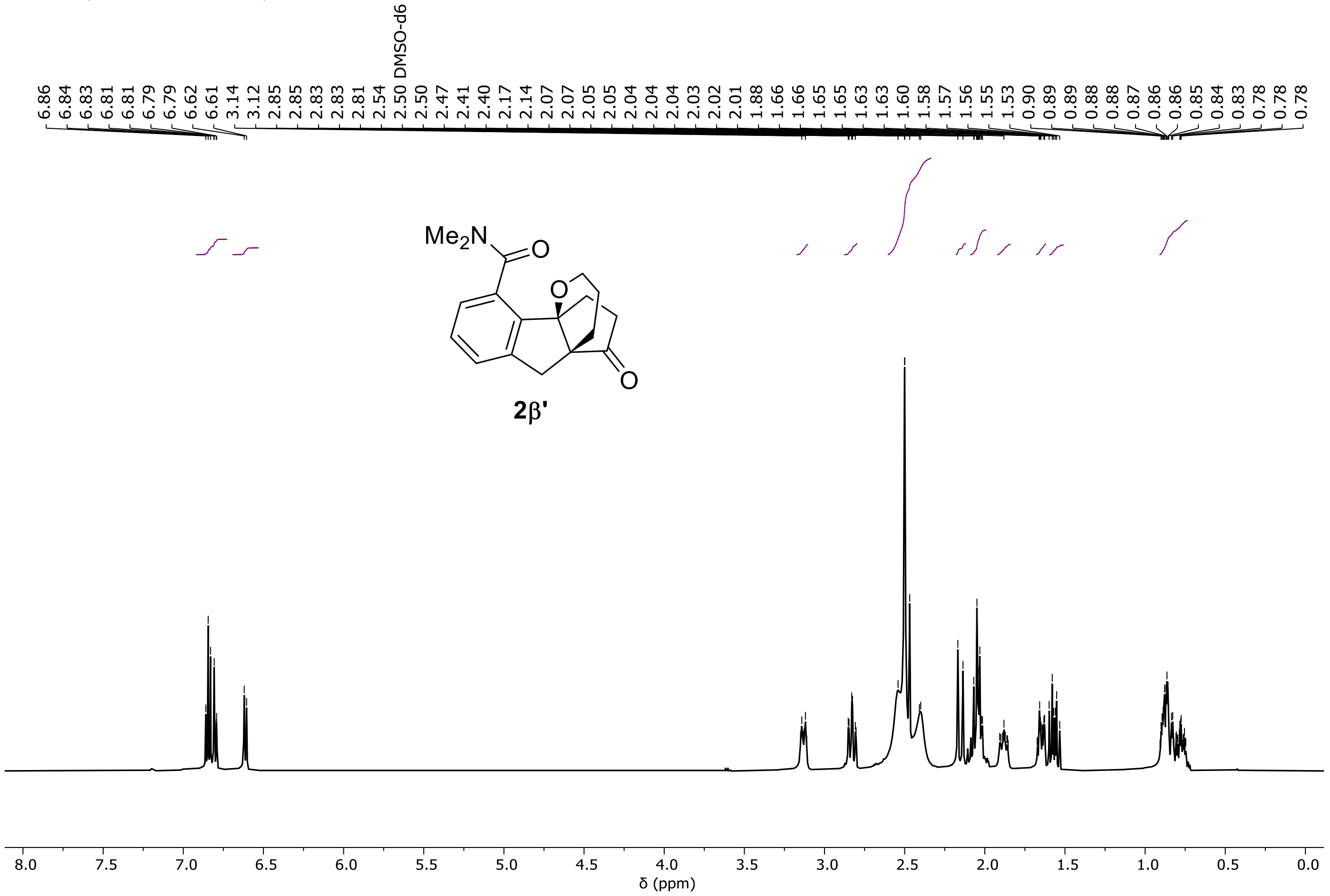

DEPT-135 NMR (126 MHz, DMSO, 100 ºC)

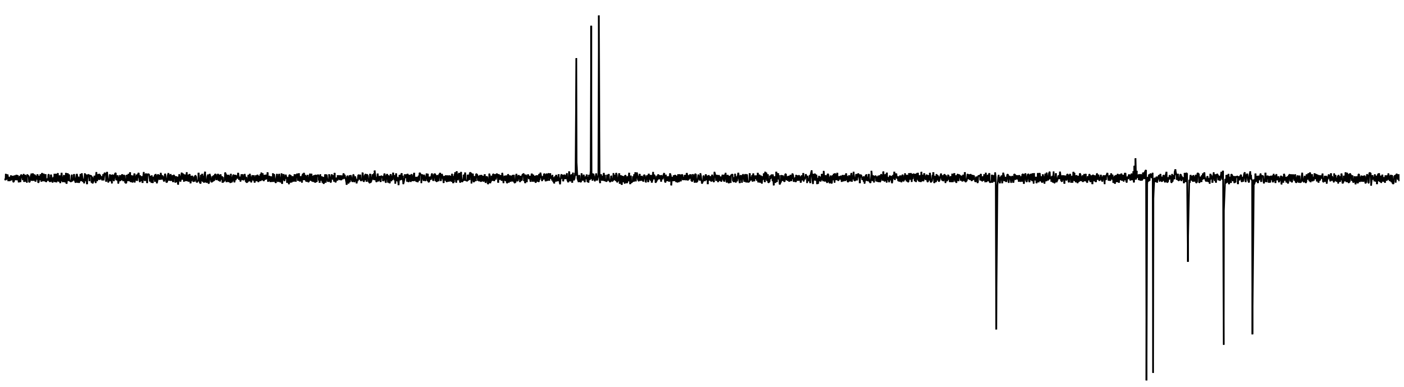

13C NMR (126 MHz, DMSO, 100 ºC)

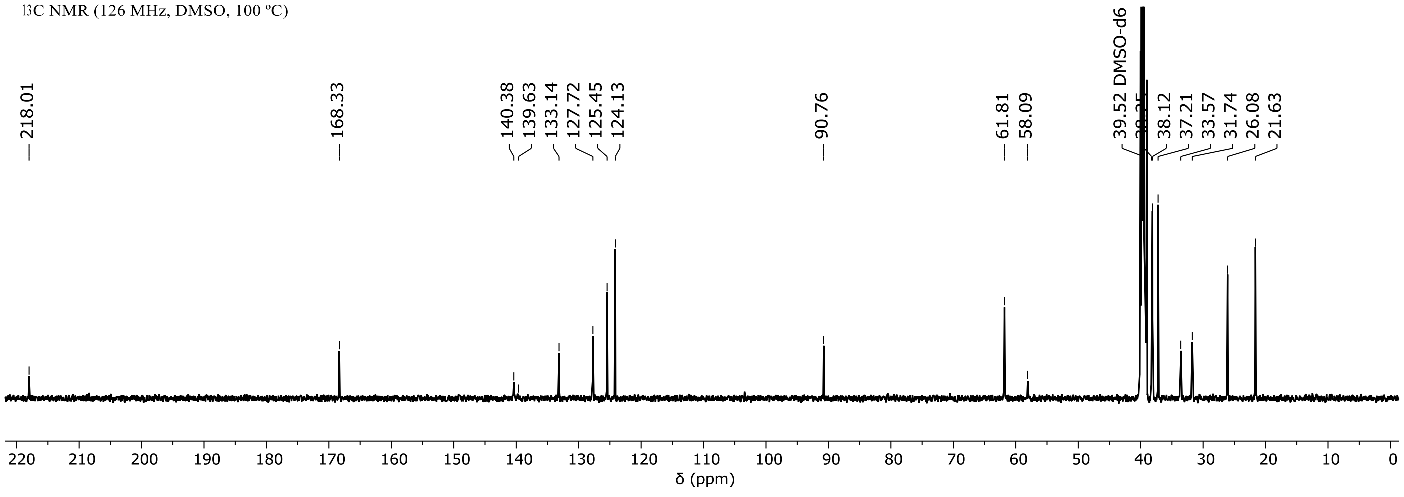

1H NMR (500 MHz, CDCl3)

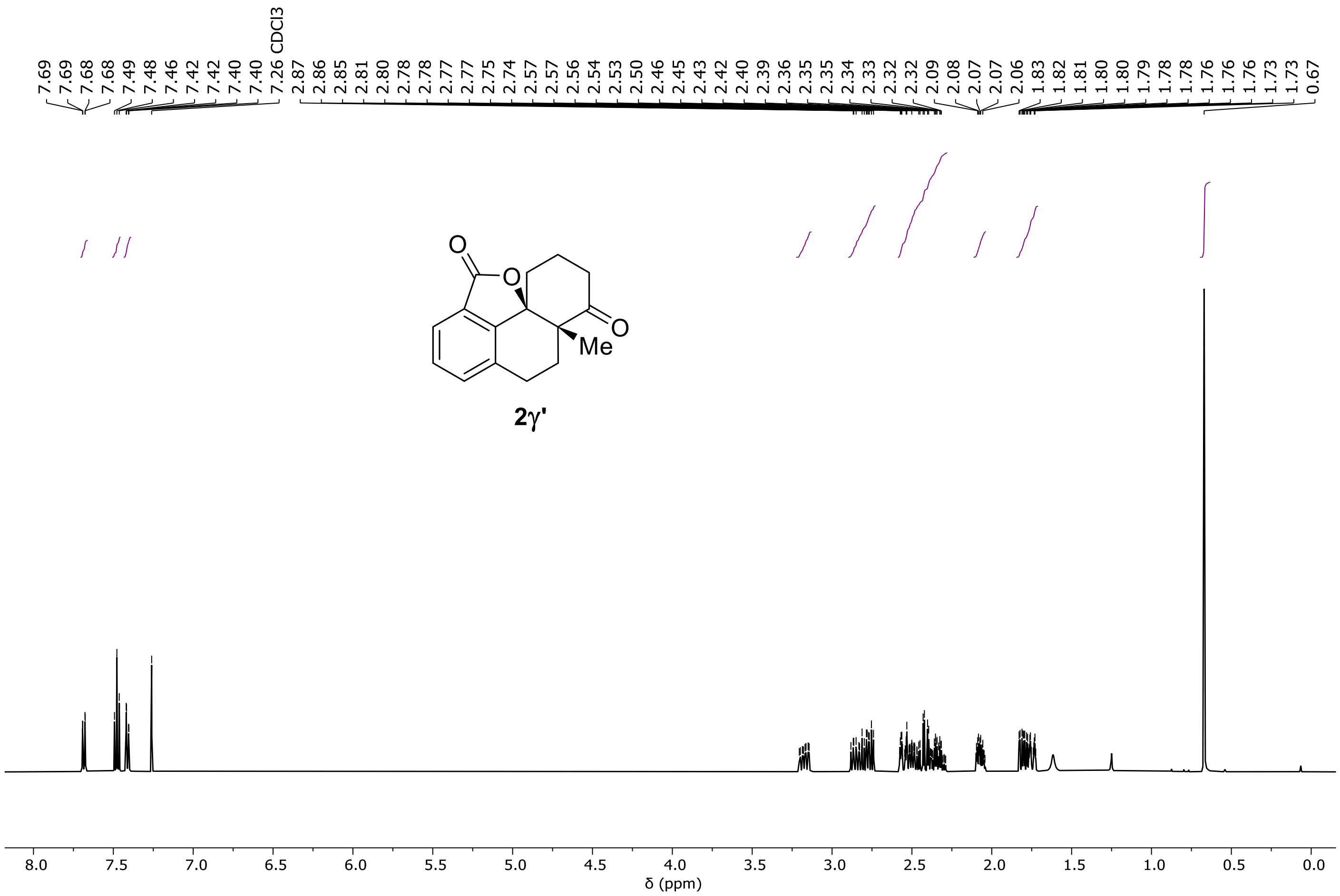

DEPT-135 NMR (126 MHz, CDCl3)

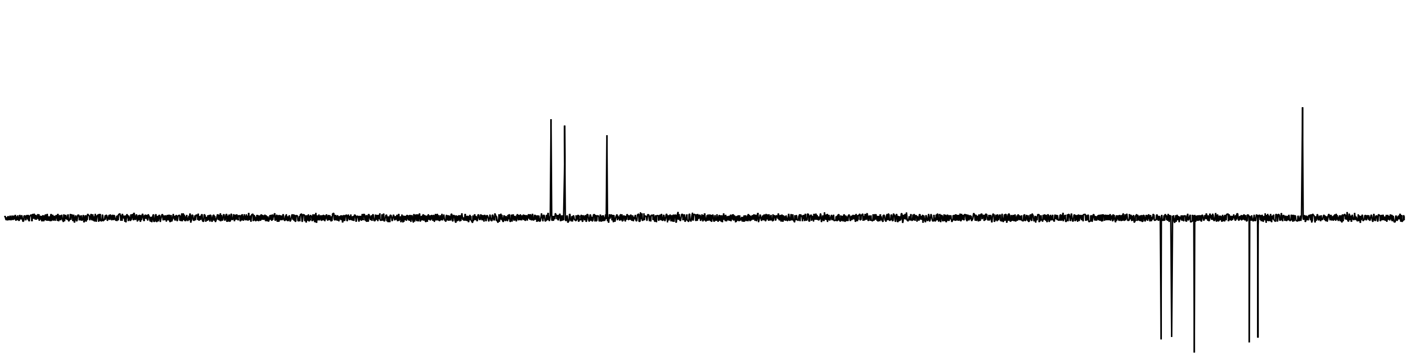

13C NMR (126 MHz, CDCl3)

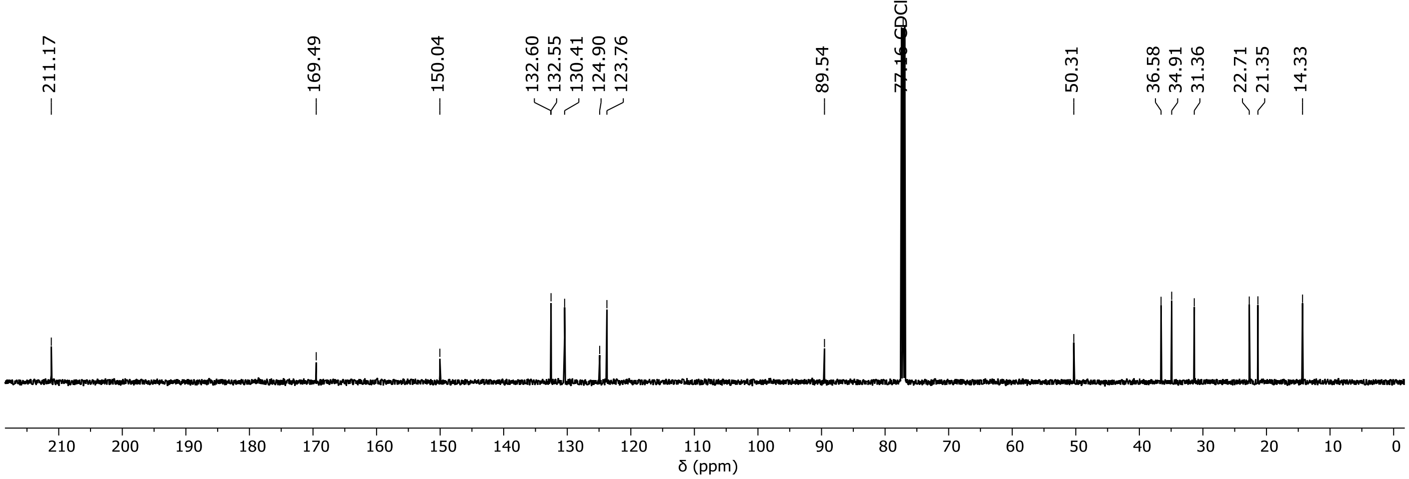

1H NMR (500 MHz, CDCl3)

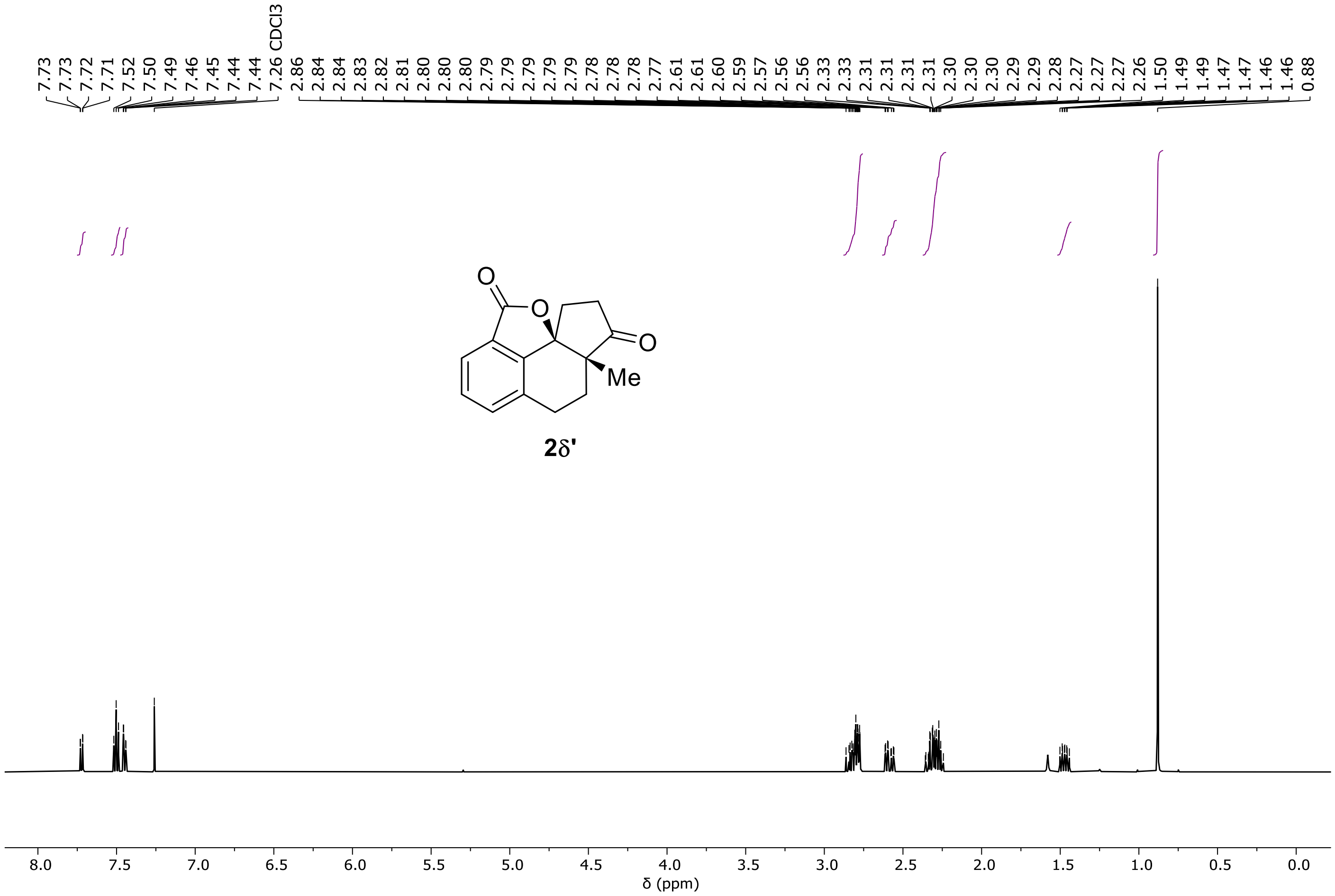

DEPT-135 NMR (126 MHz, CDCl3)

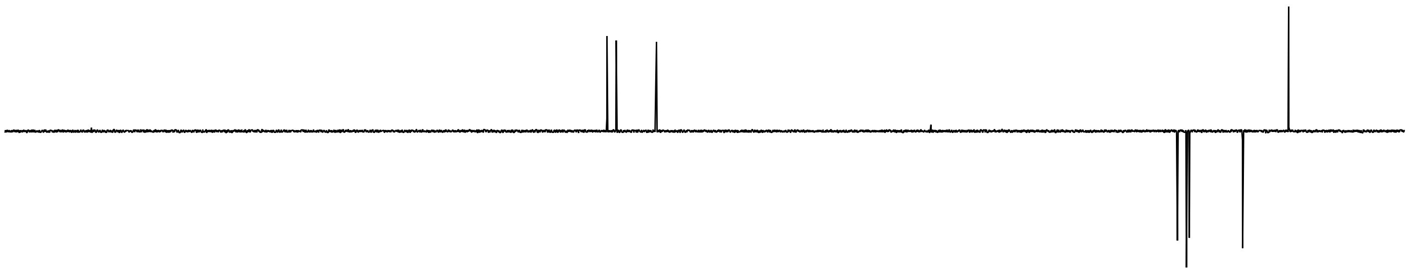

13C NMR (126 MHz, CDCl3)

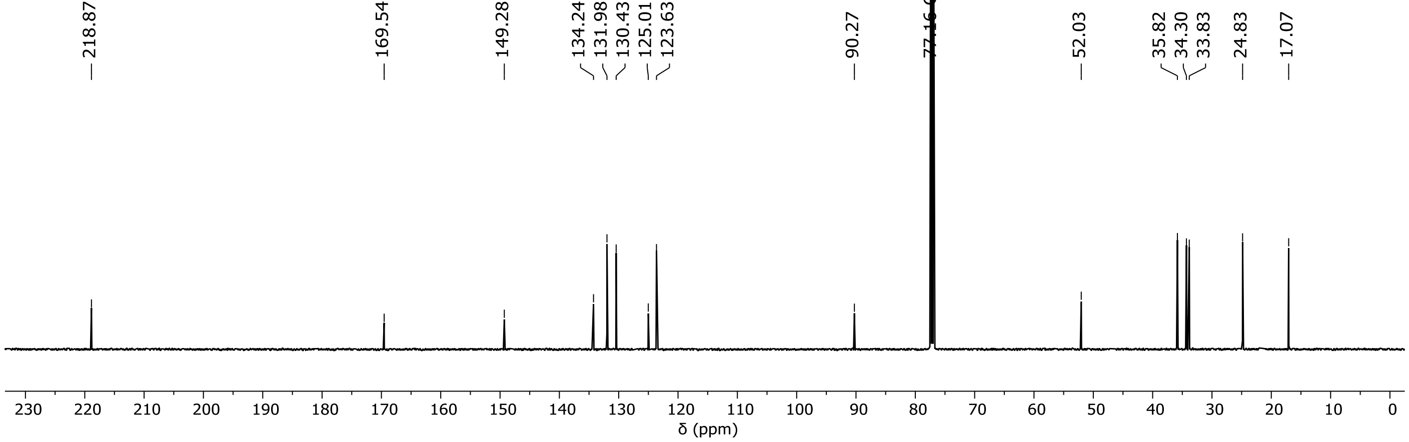

1H NMR (500 MHz, CDCl3)

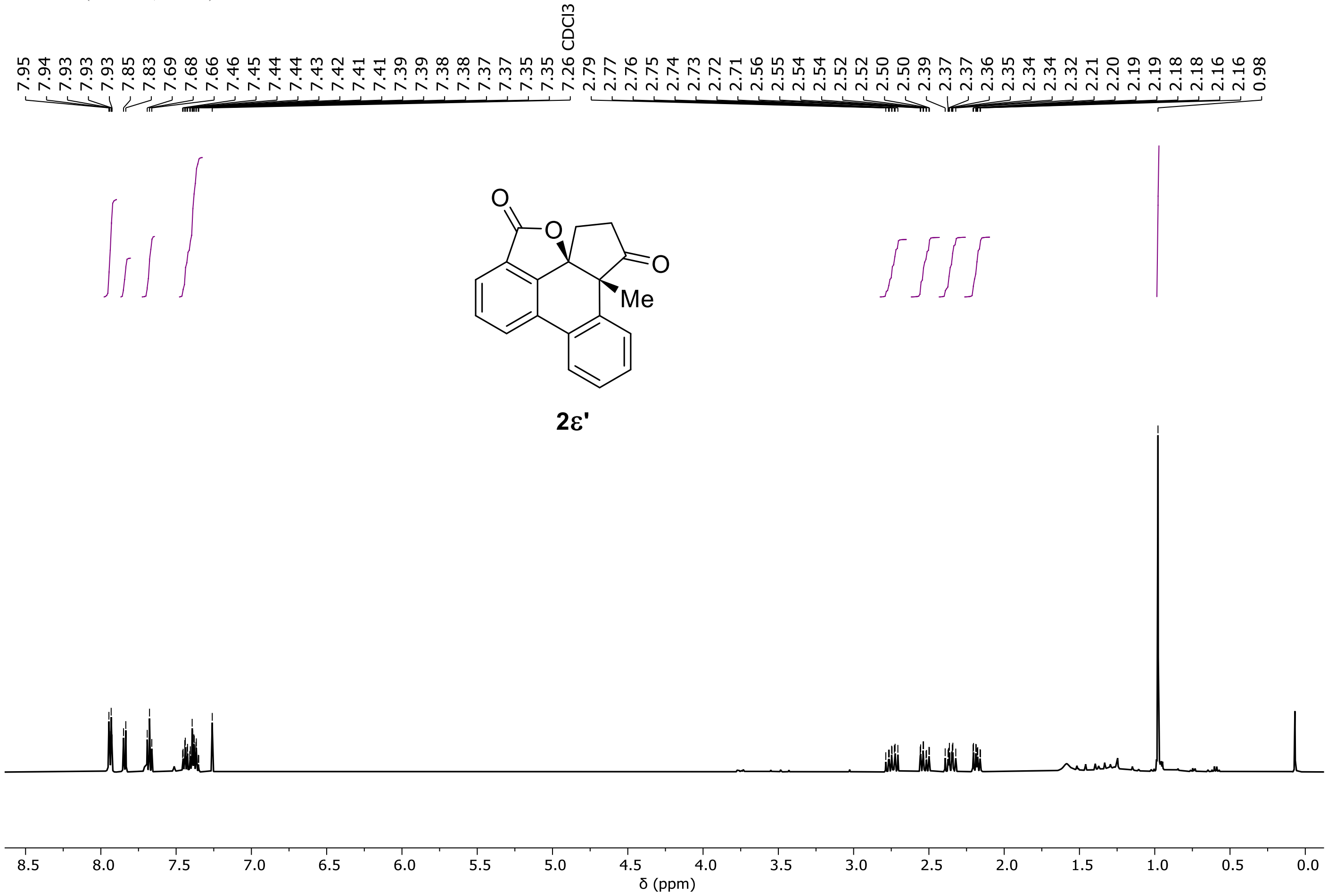

2ε'

DEPT-135 NMR (126 MHz, CDCl3)

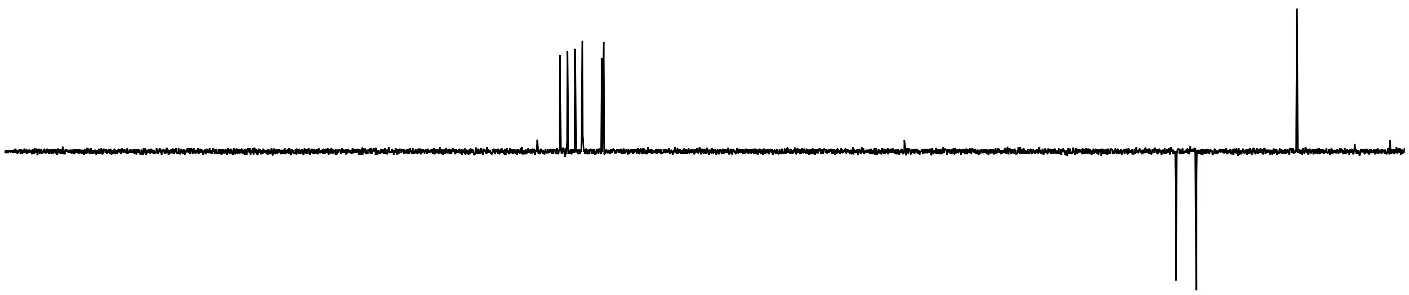

13C NMR (126 MHz, CDCl3)

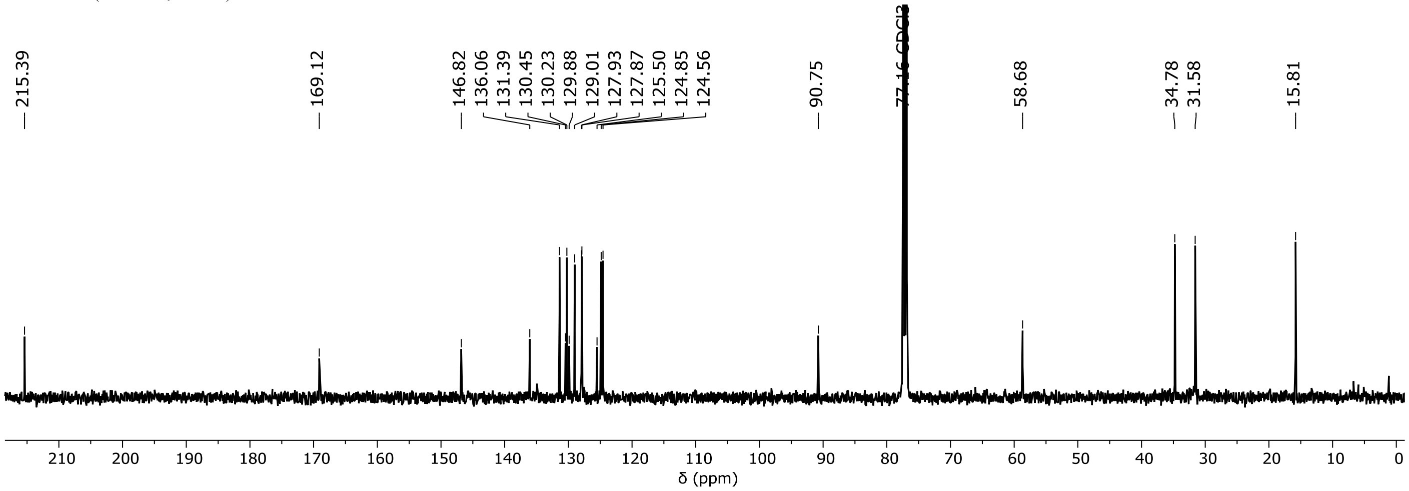

1H NMR (500 MHz, CDCl3)

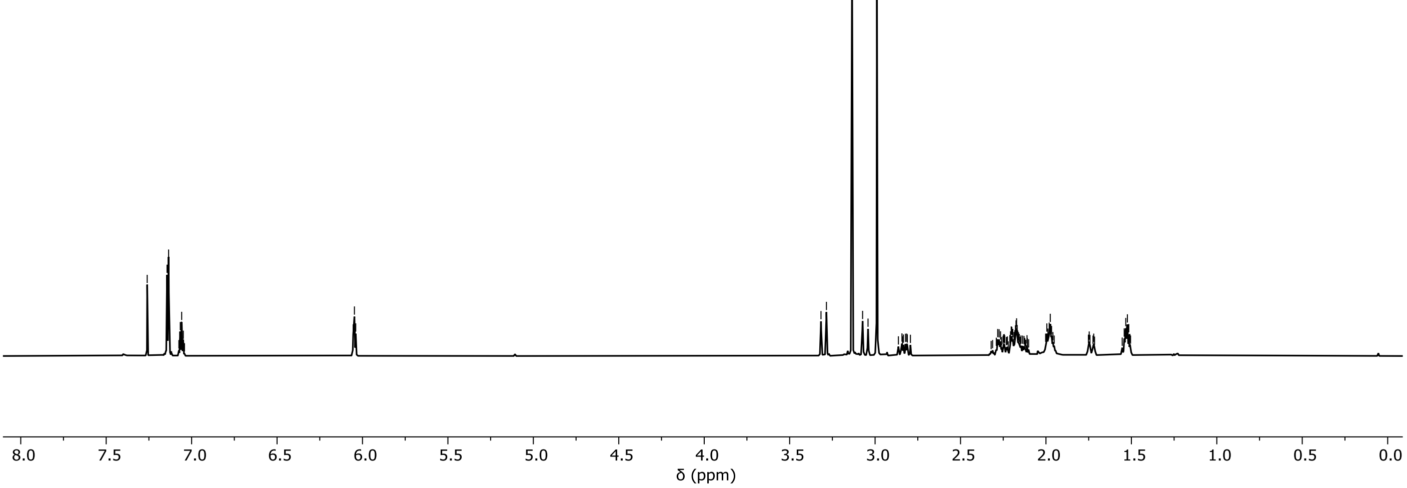

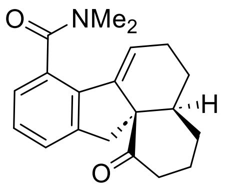

5a

DEPT-135 NMR (126 MHz, CDCl3)

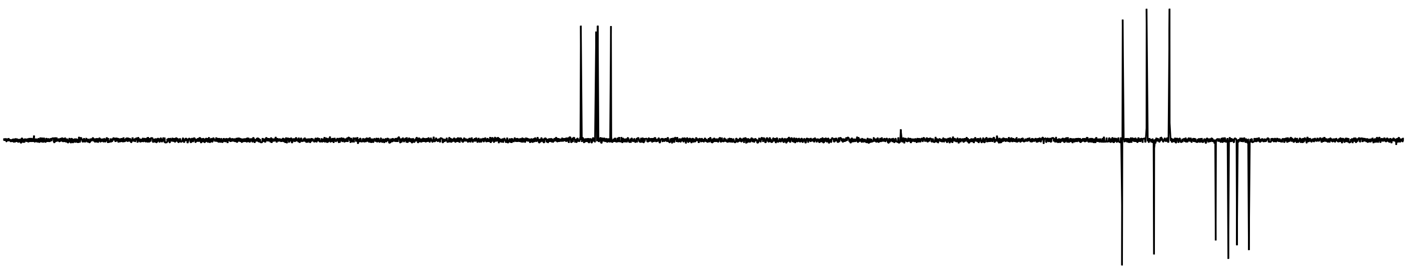

13C NMR (126 MHz, CDCl3)

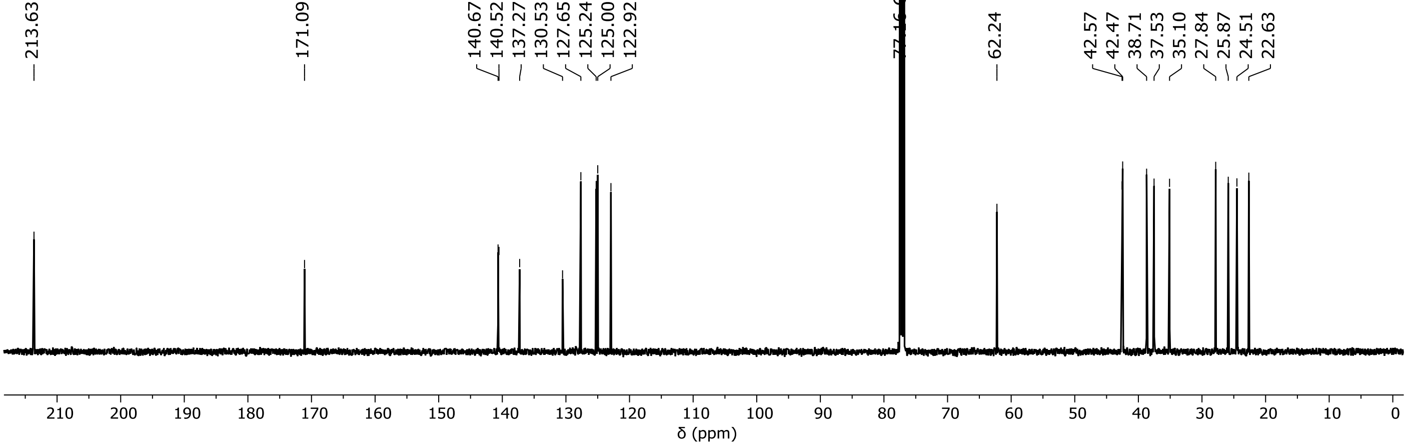

1H NMR (500 MHz, CDCl3)

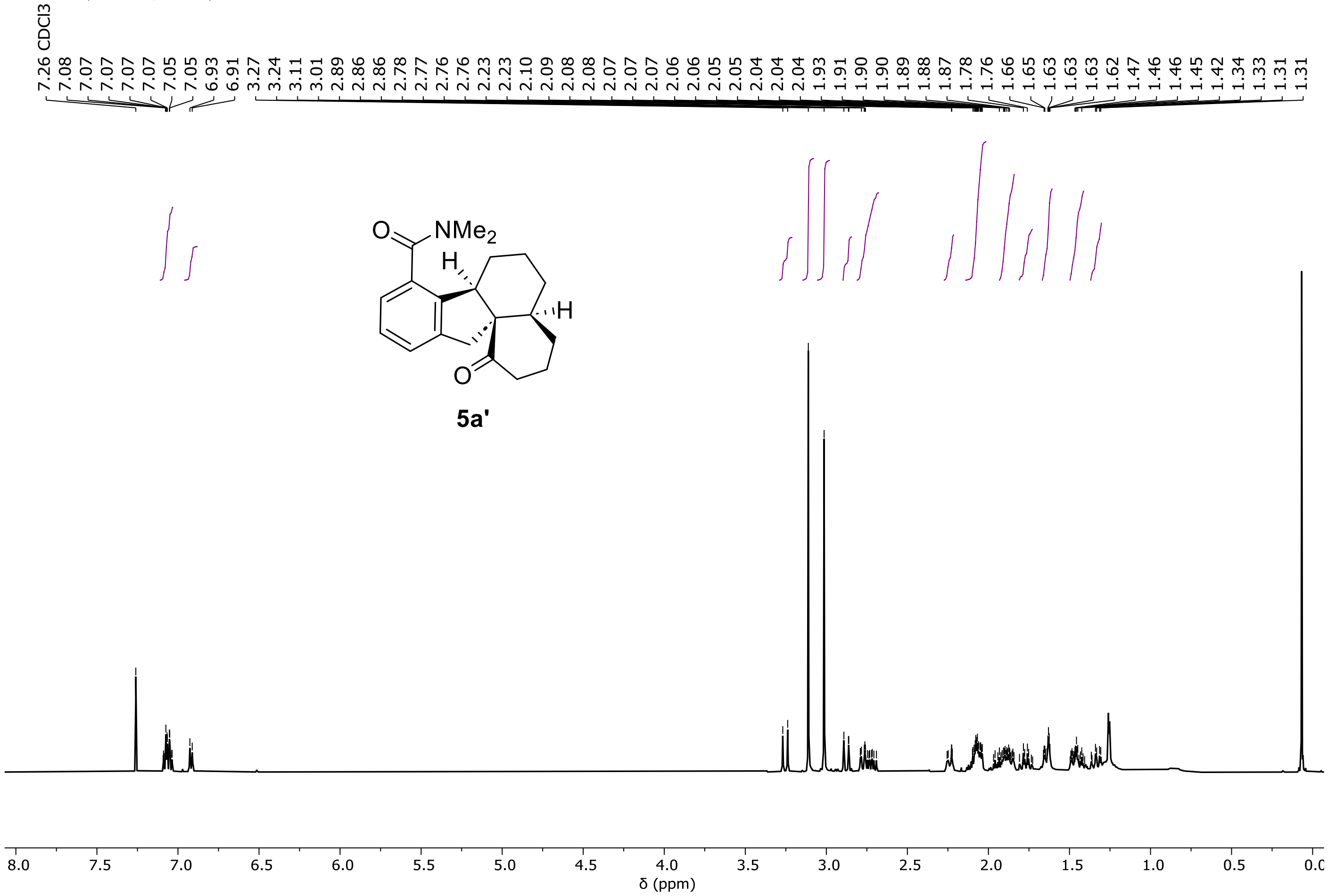

DEPT-135 NMR (126 MHz, CDCl3)

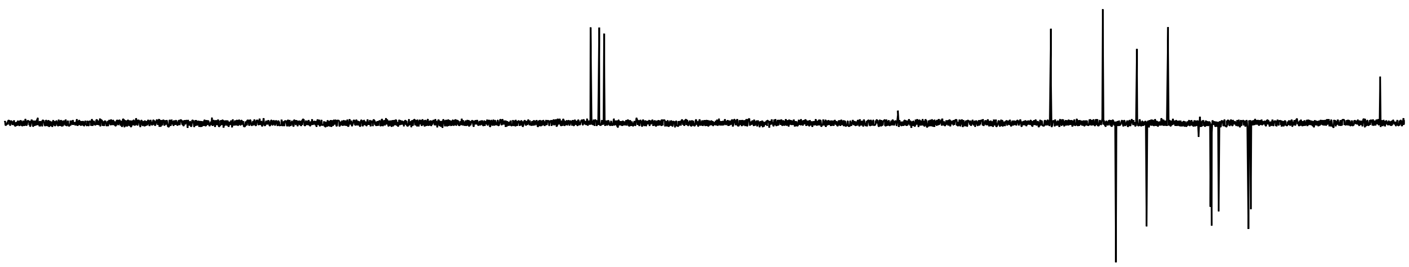

13C NMR (126 MHz, CDCl3)

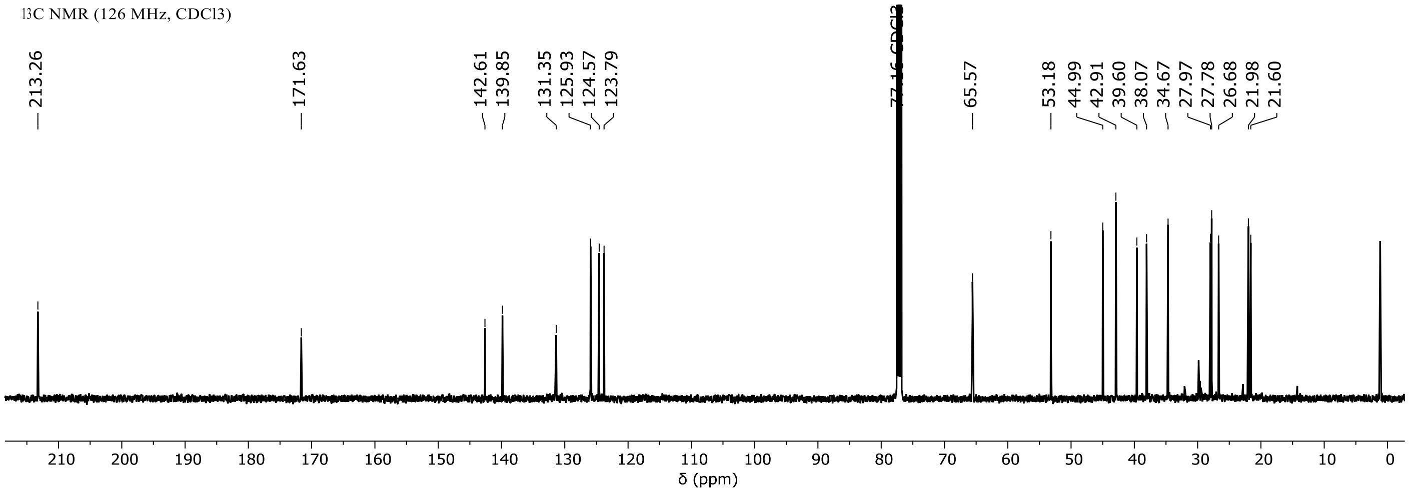

1H NMR (500 MHz, CDCl3)

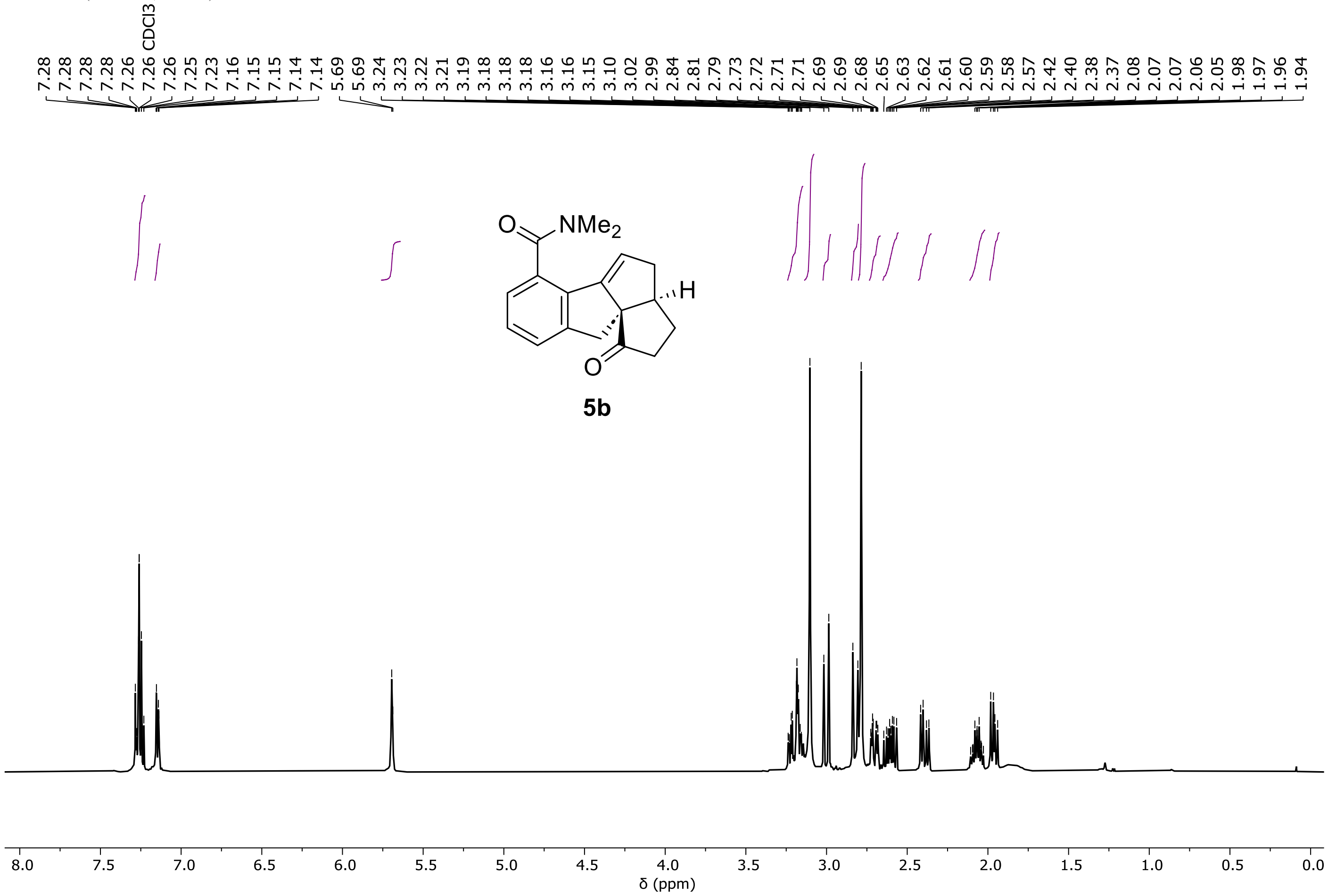

DEPT-135 NMR (126 MHz, CDCl3)

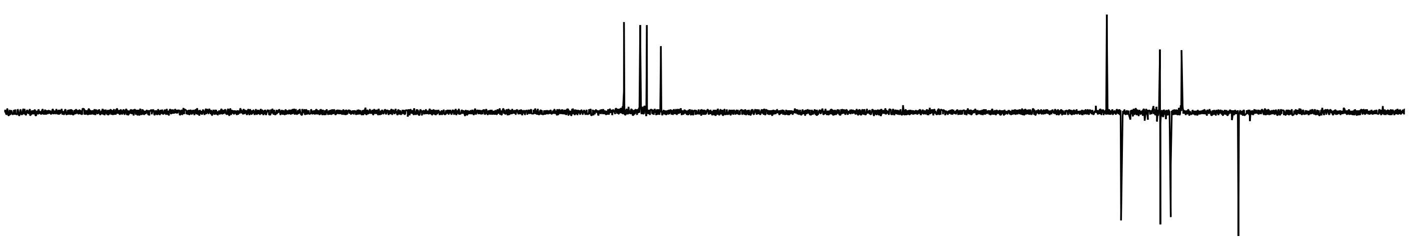

13C NMR (126 MHz, CDCl3)

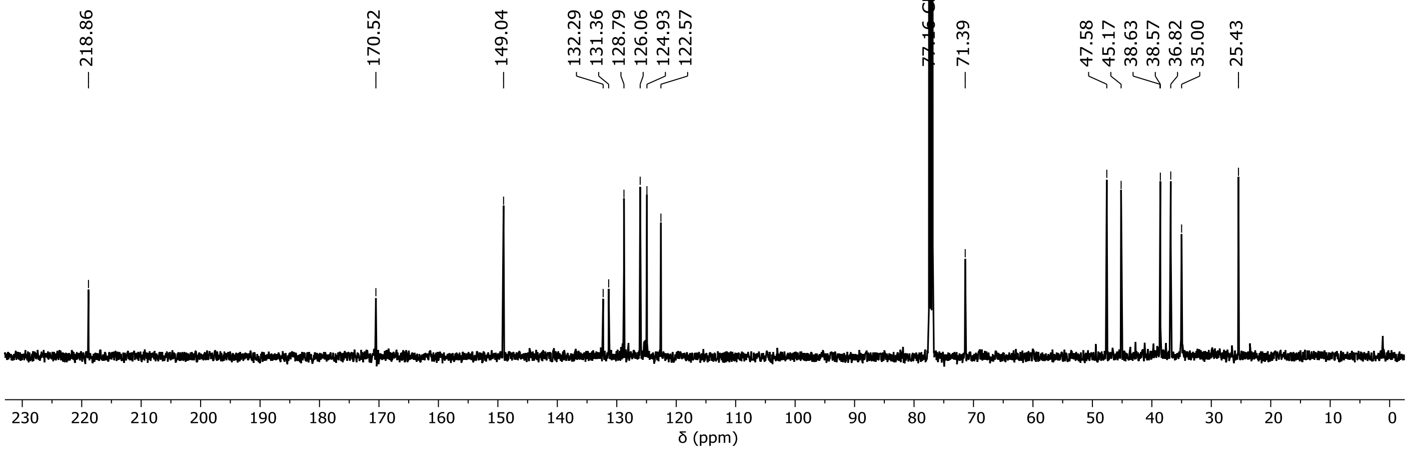

1H NMR (500 MHz, CDCl3)

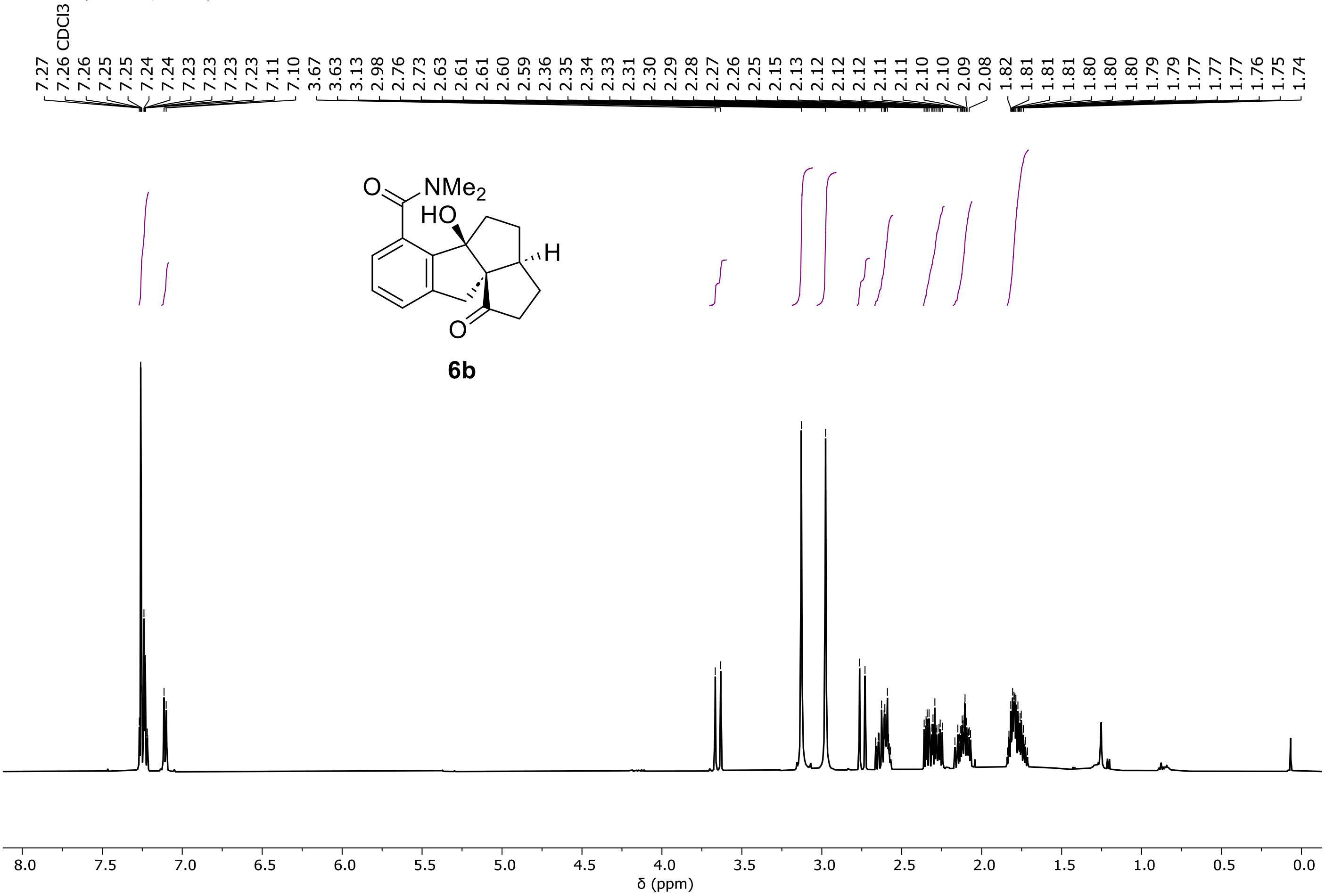

DEPT-135 NMR (126 MHz, CDCl3)

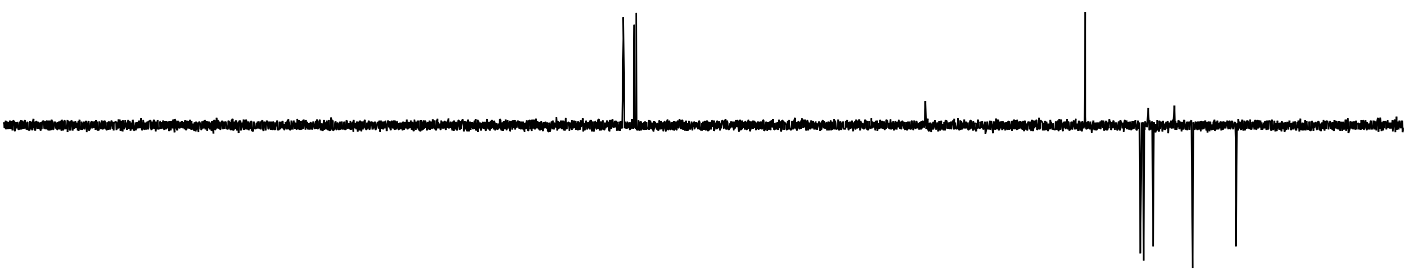

13C NMR (126 MHz, CDCl3)

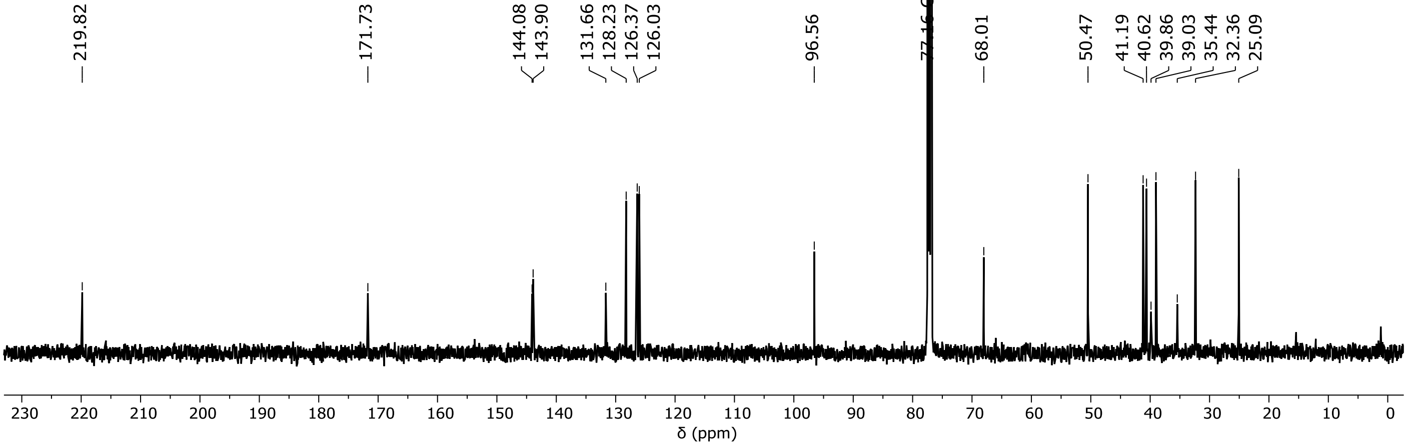

1H NMR (300 MHz, CDCl3)

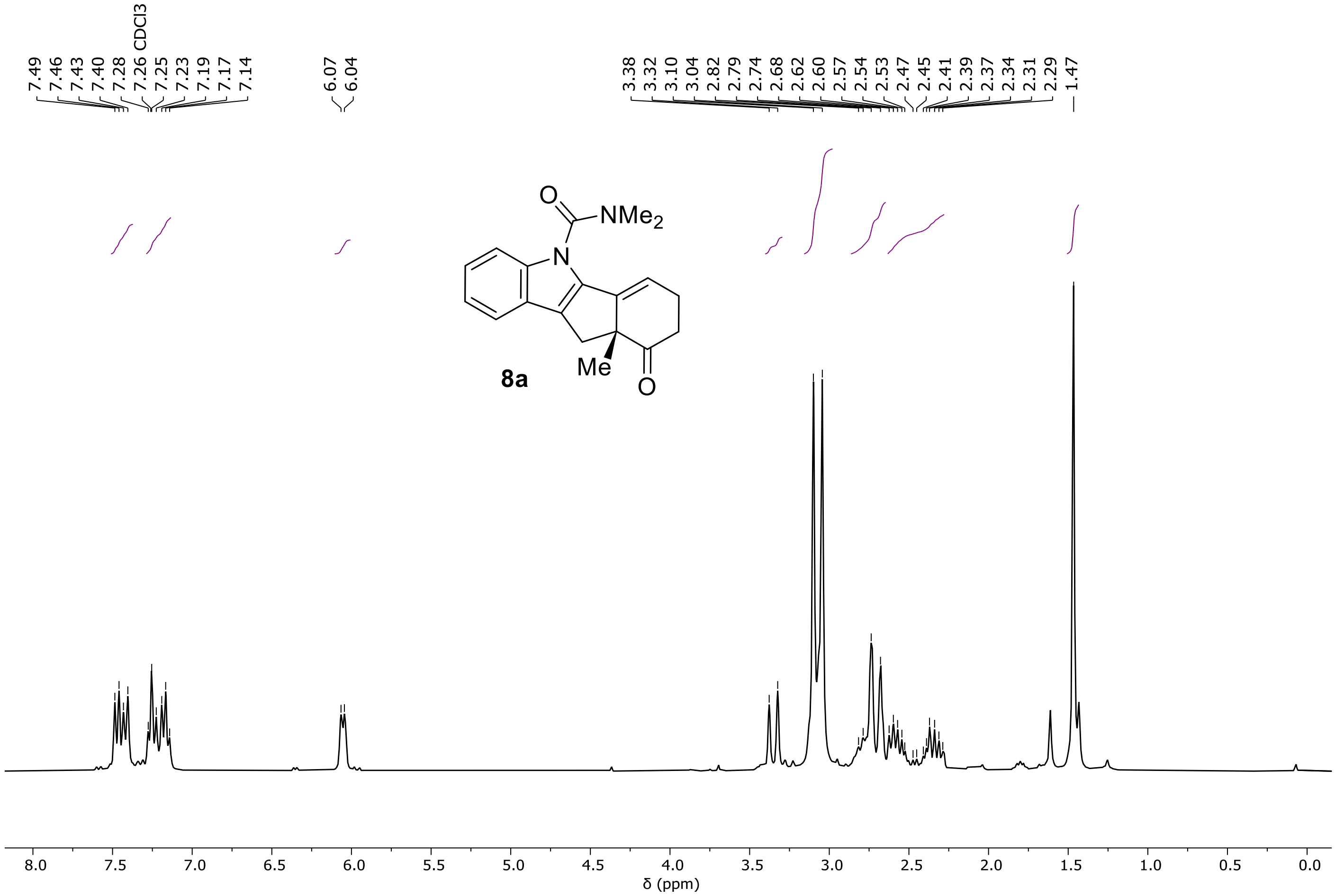

DEPT-135 NMR (75 MHz, CDCl3)

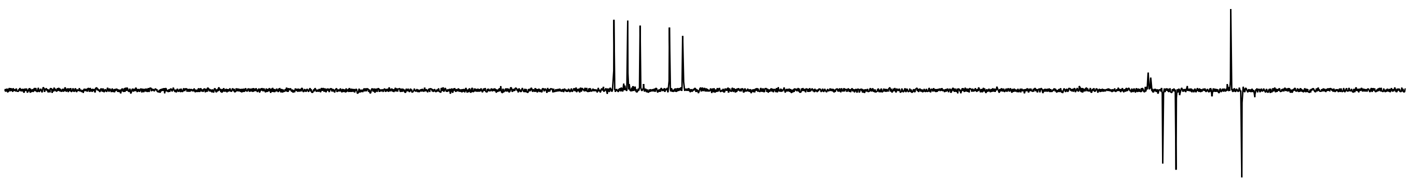

13C NMR (75 MHz, CDCl3)

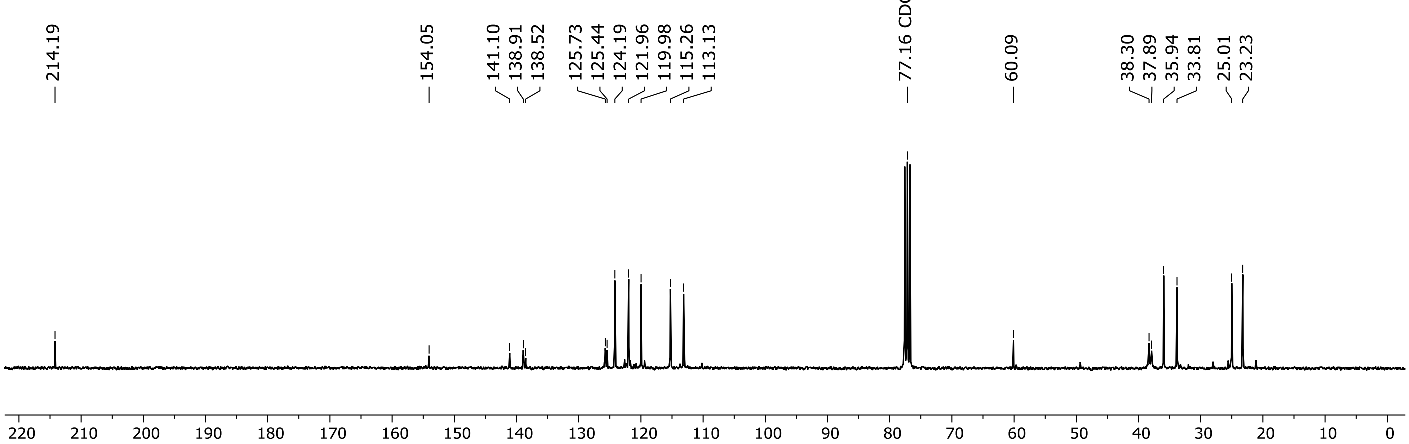

1H NMR (500 MHz, CD2Cl2)

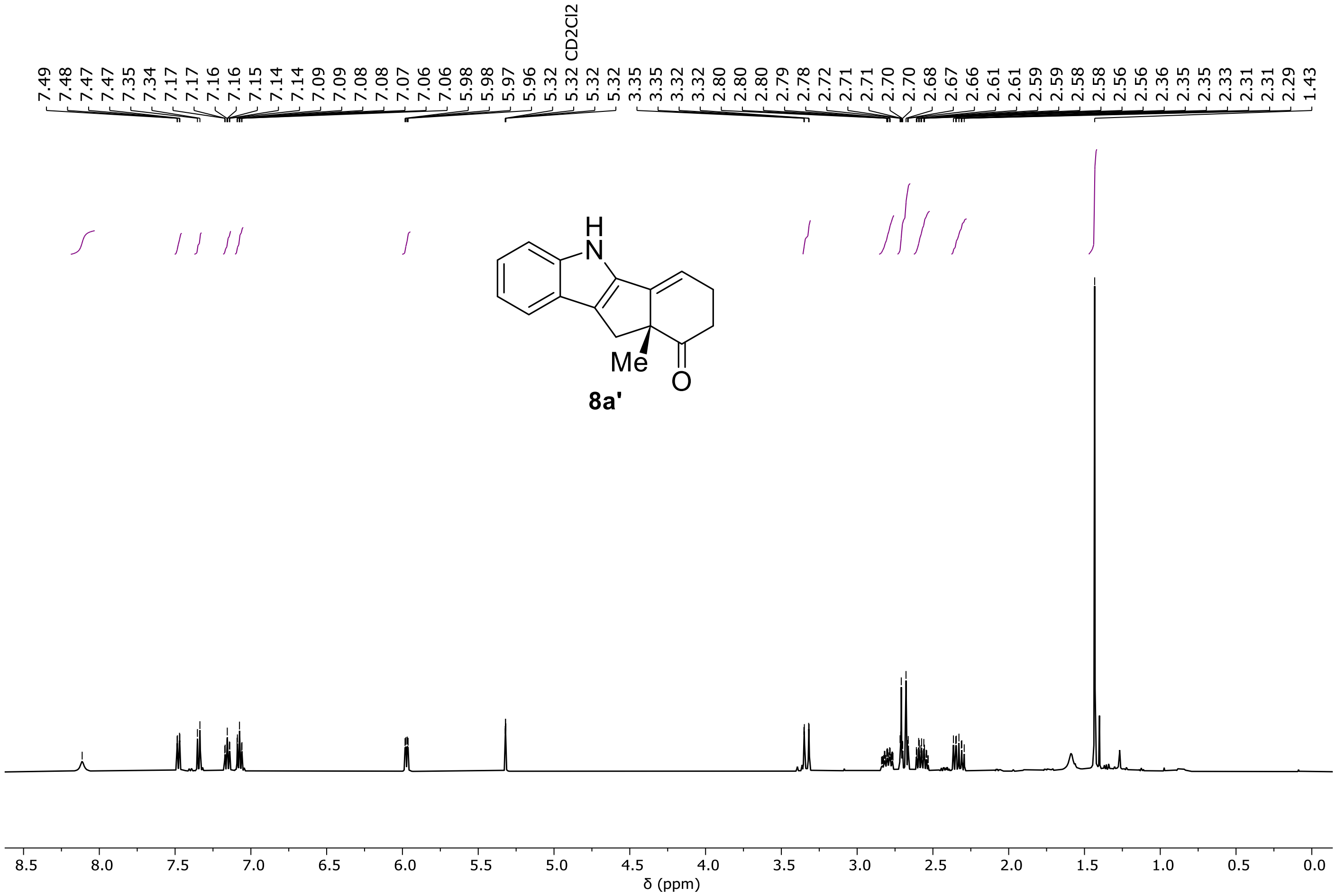

DEPT-135 NMR (126 MHz, CD2Cl2)

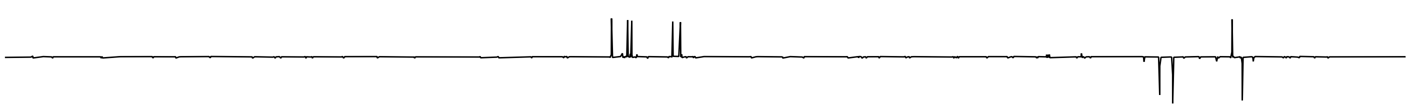

13C NMR (126 MHz, CD2Cl2)

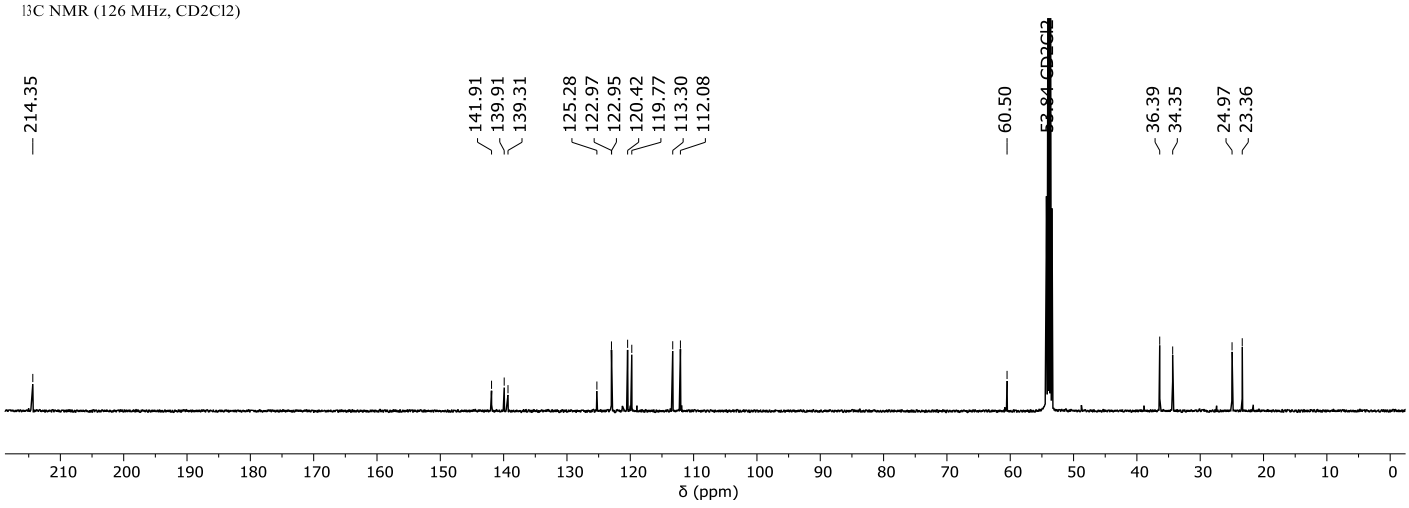

1H NMR (300 MHz, CDCl3)

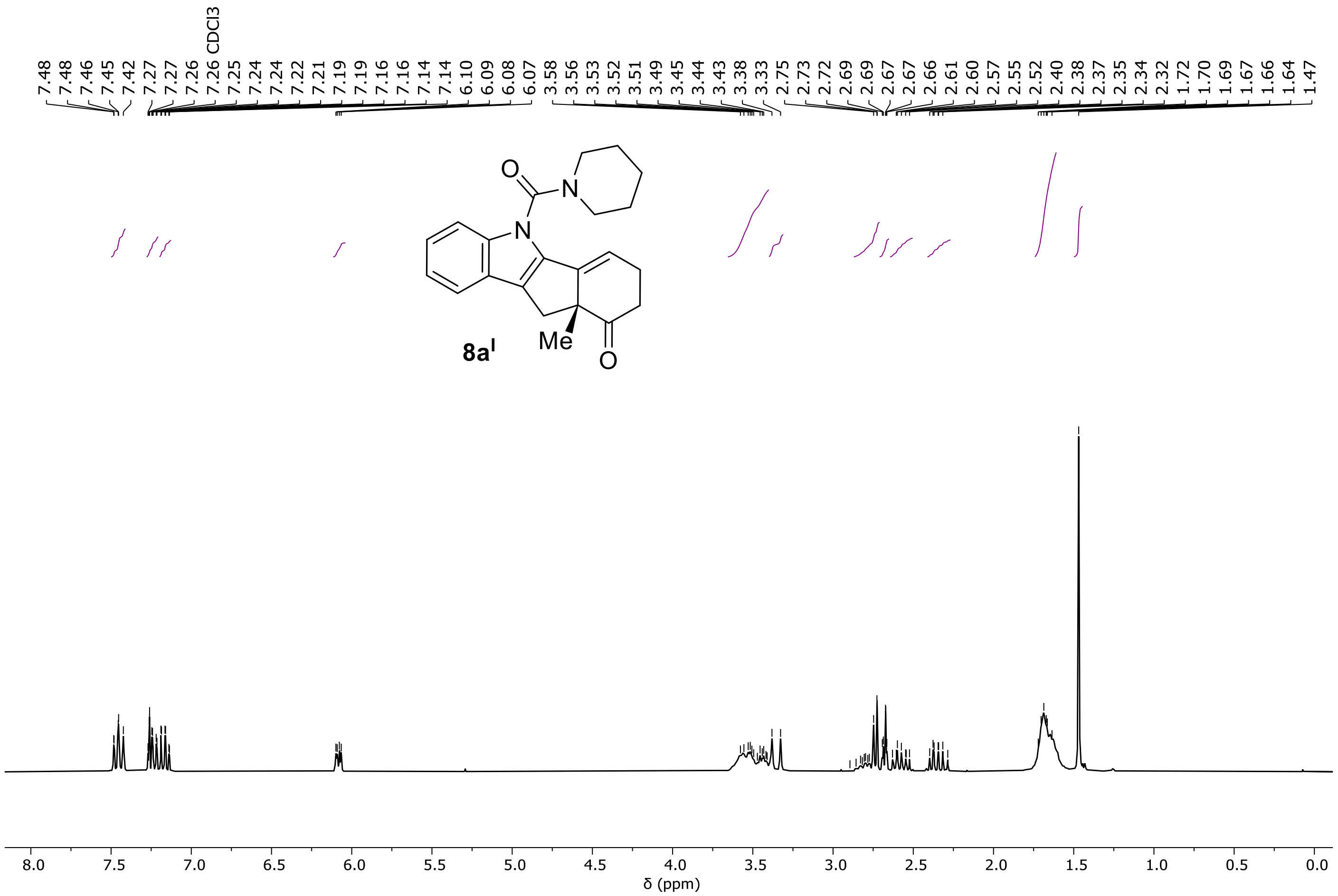

DEPT-135 NMR (75 MHz, CDCl3)

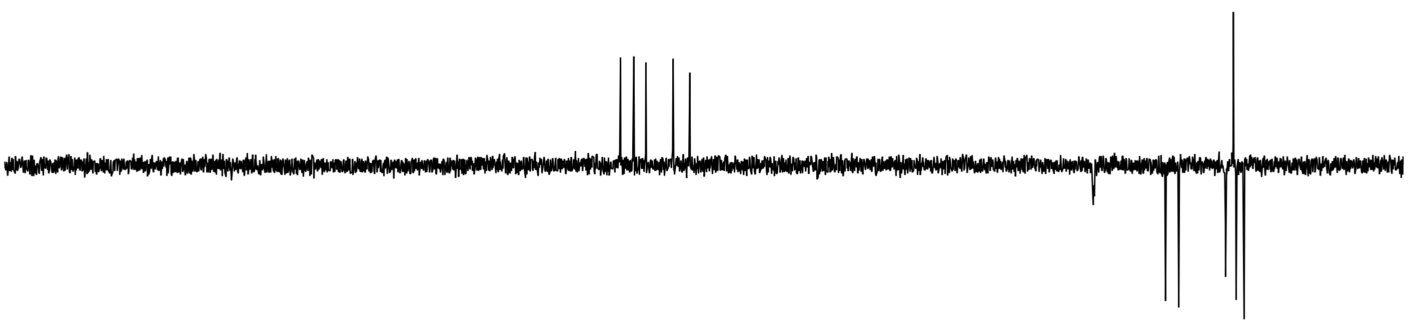

13C NMR (75 MHz, CDCl3)

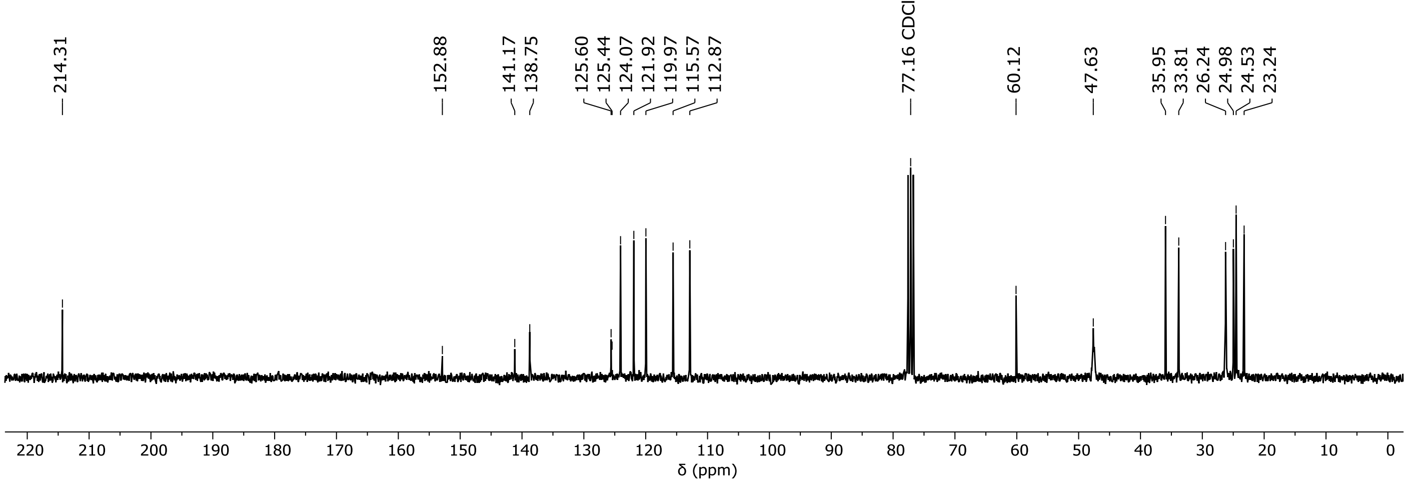

1H NMR (300 MHz, CDCl3)

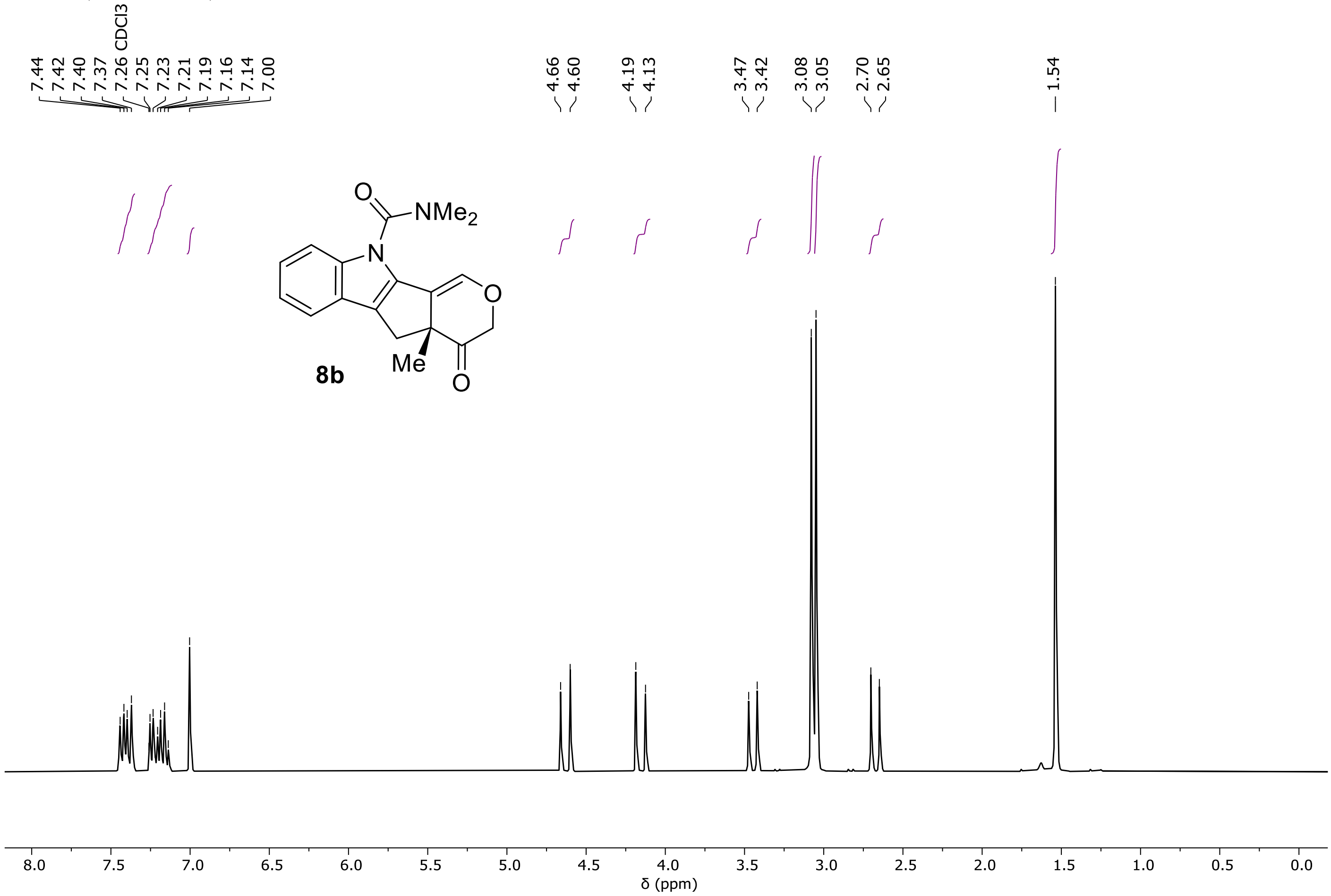

DEPT-135 NMR (75 MHz, CDCl3)

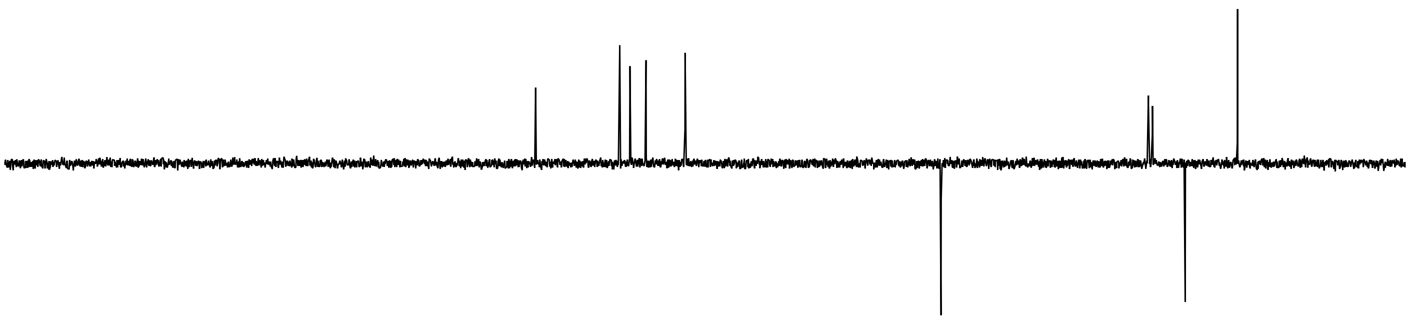

13C NMR (75 MHz, CDCl3)

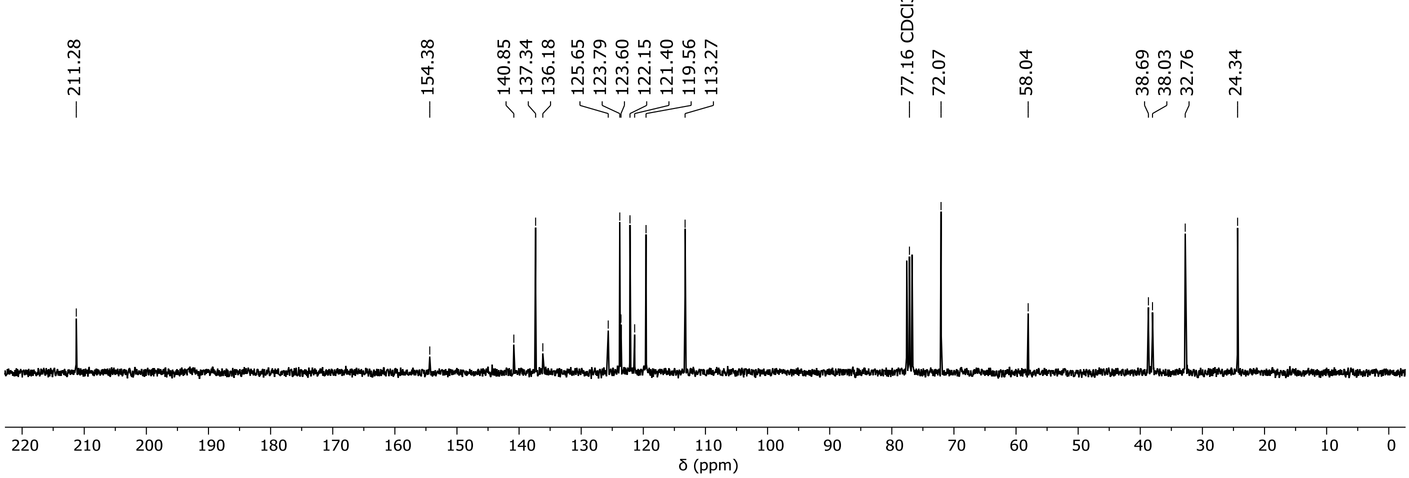

1H NMR (300 MHz, CDCl3)

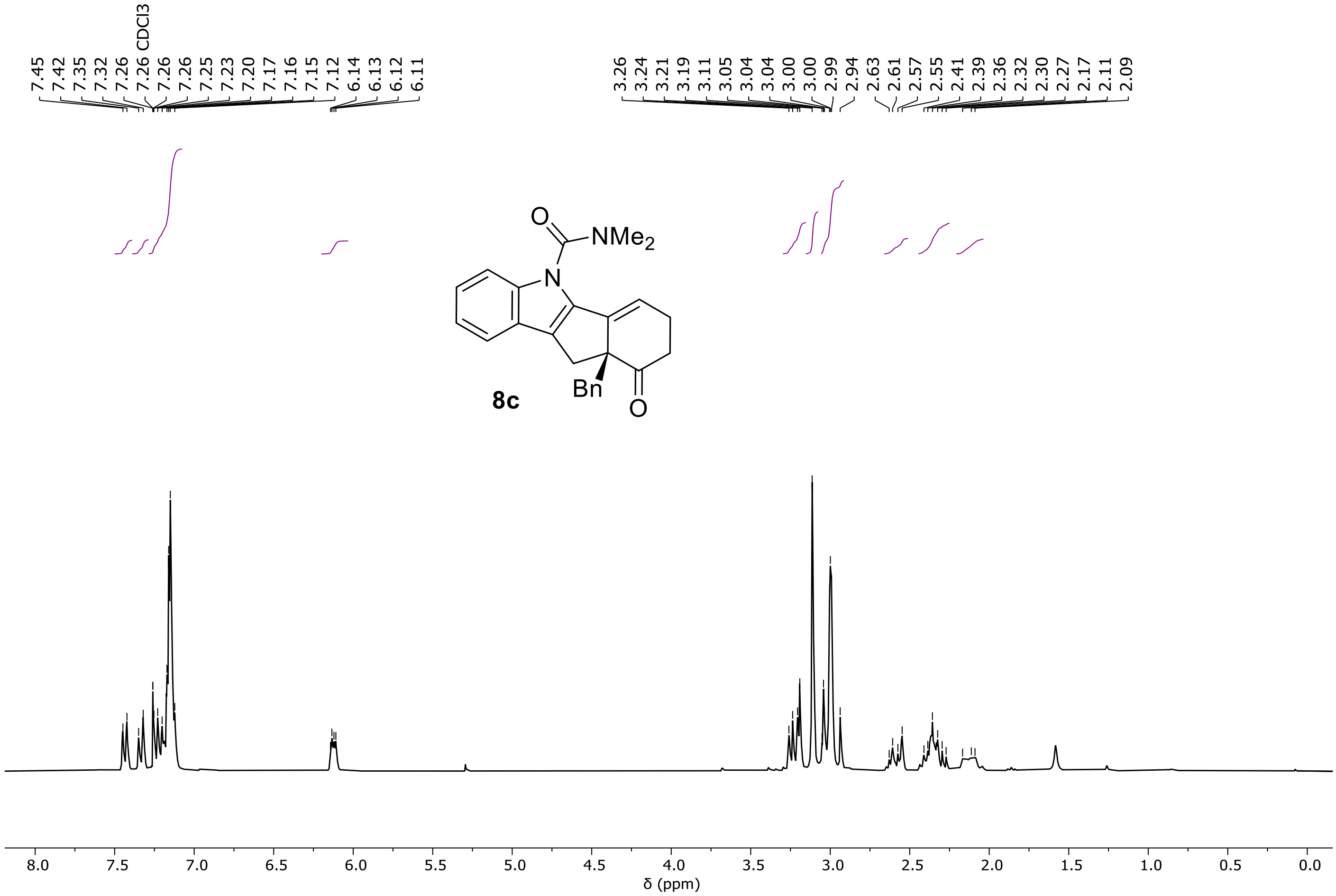

DEPT-135 NMR (75 MHz, CDCl3)

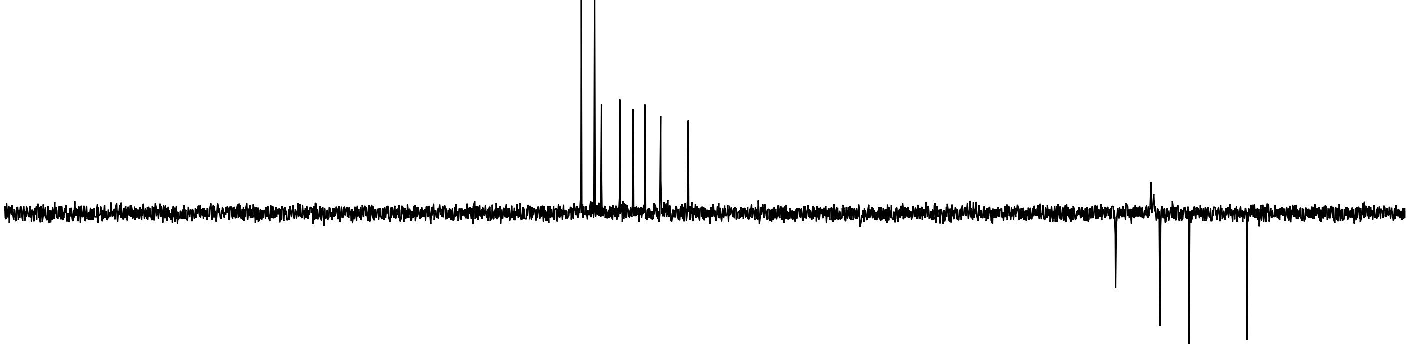

13C NMR (75 MHz, CDCl3)

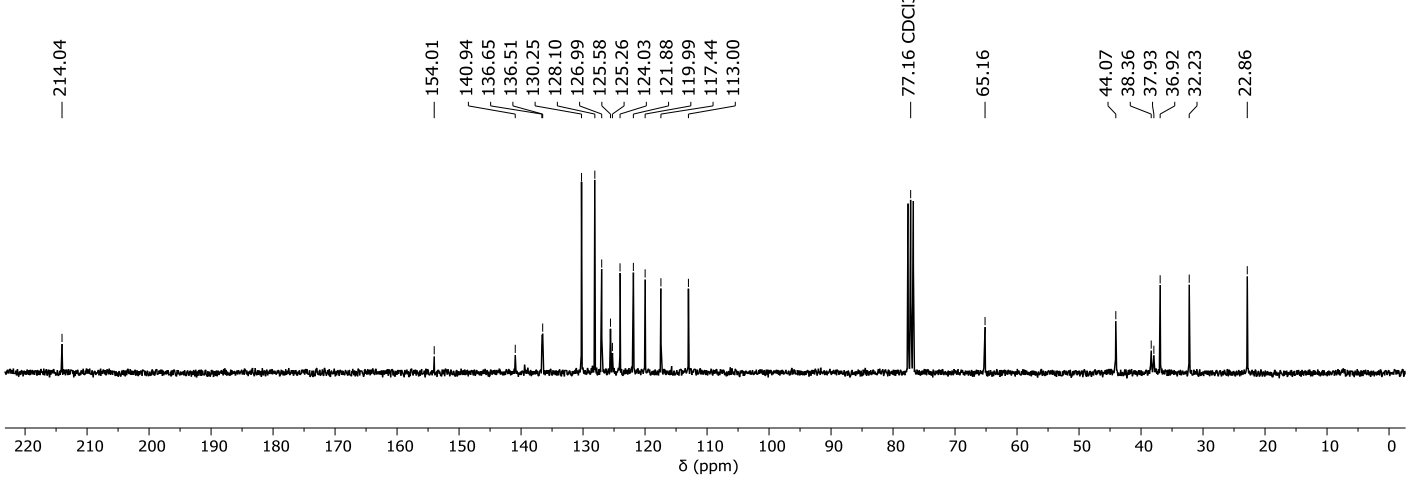

1H NMR (300 MHz, CDCl3)

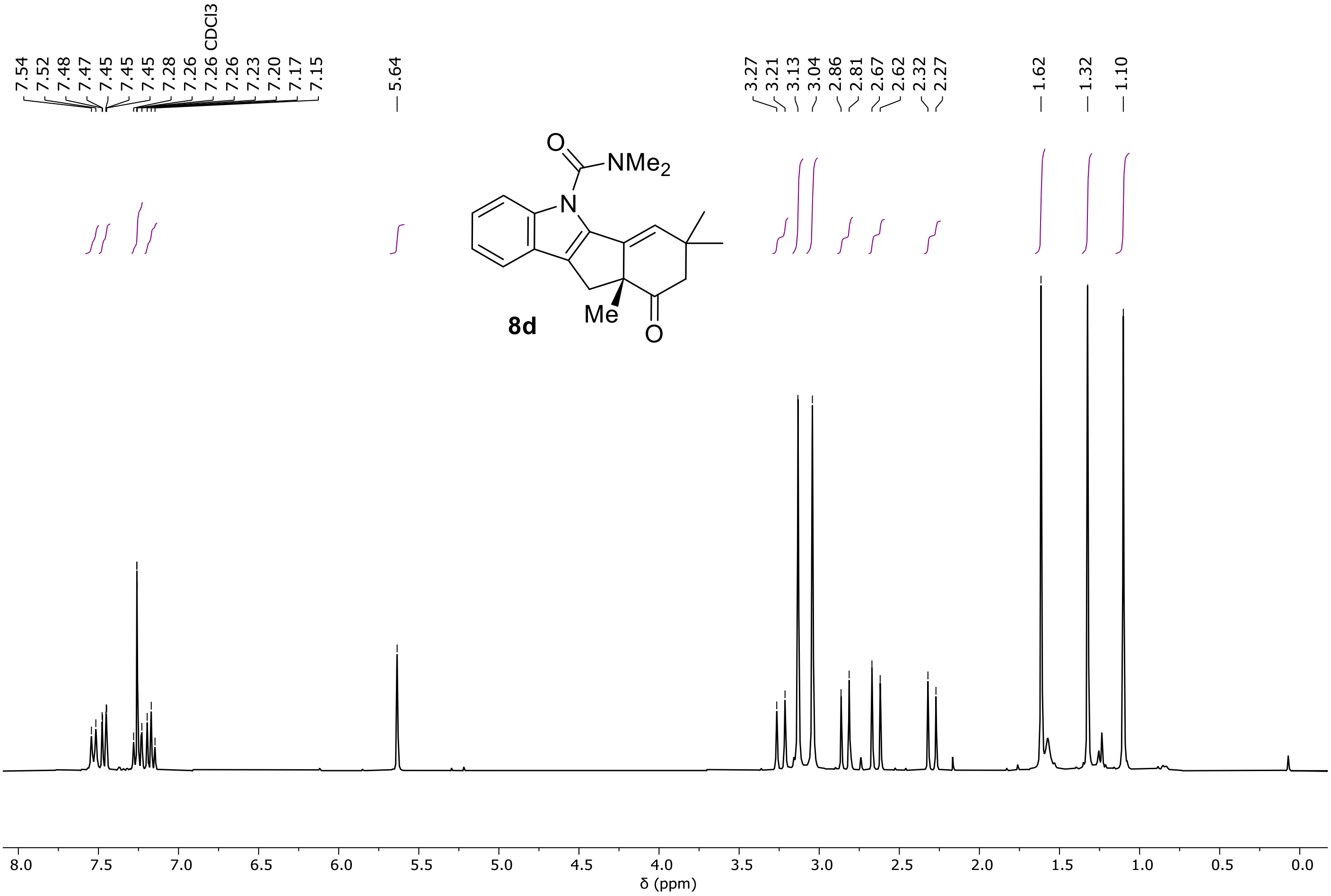

DEPT-135 NMR (75 MHz, CDCl3)

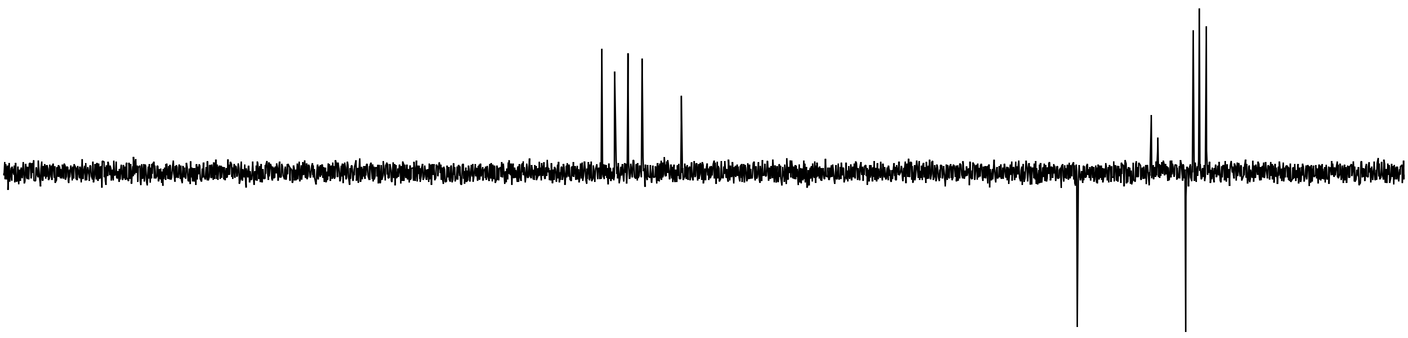

13C NMR (75 MHz, CDCl3)

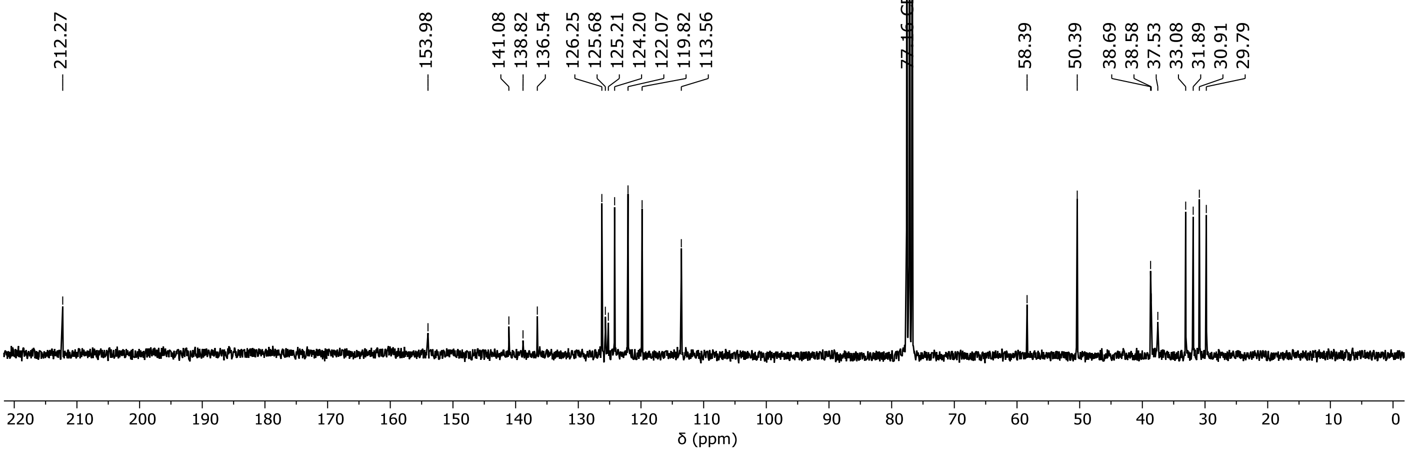

1H NMR (300 MHz, C6D6)

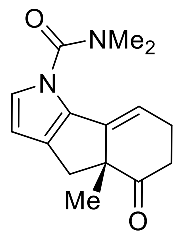

8e

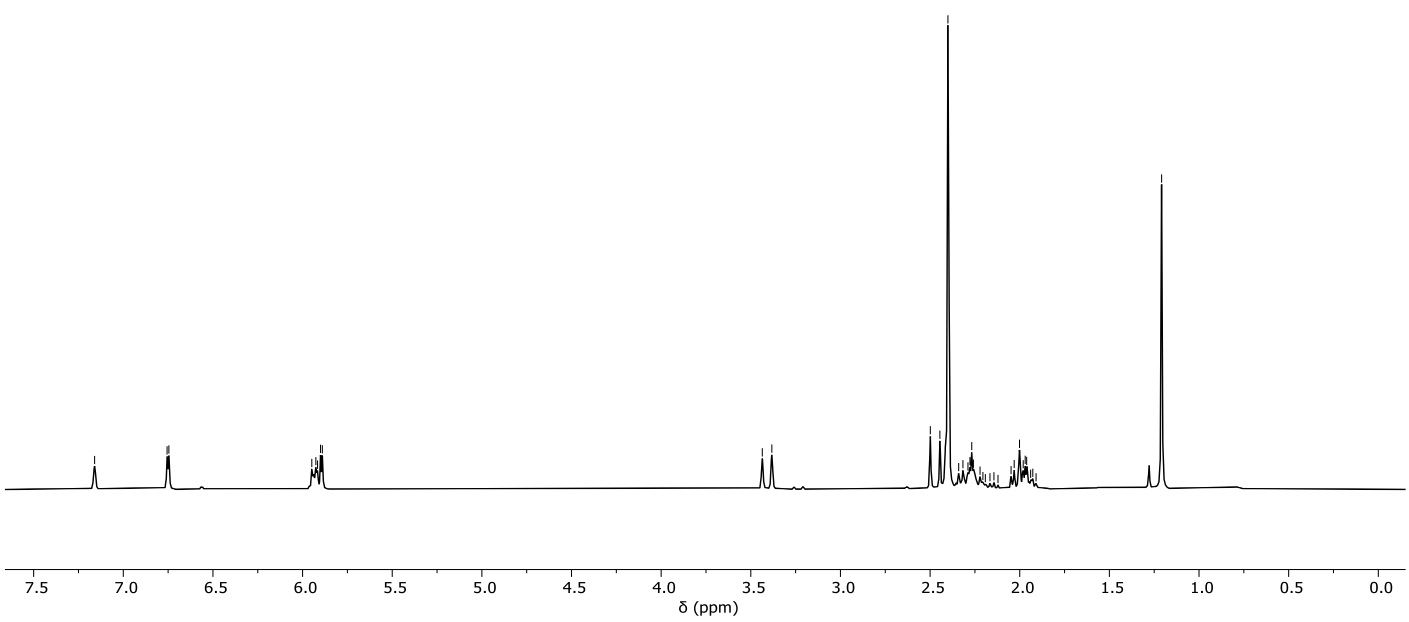

DEPT-135 NMR (75 MHz, C6D6)

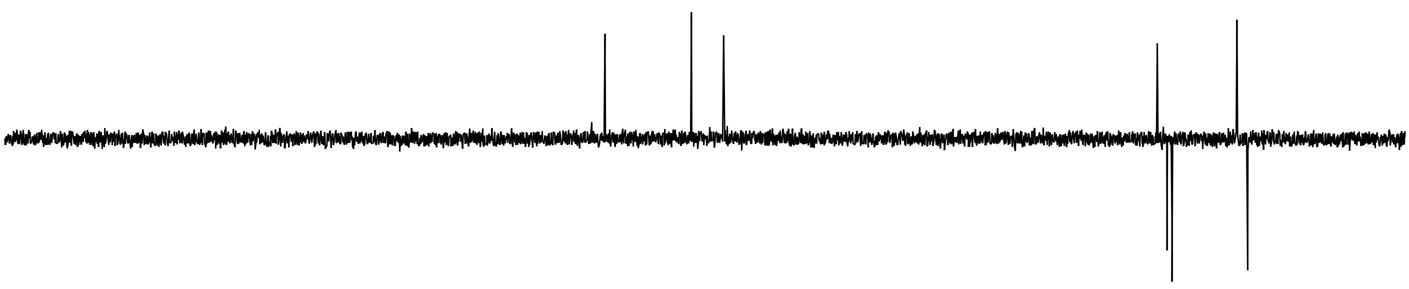

13C NMR (75 MHz, C6D6)

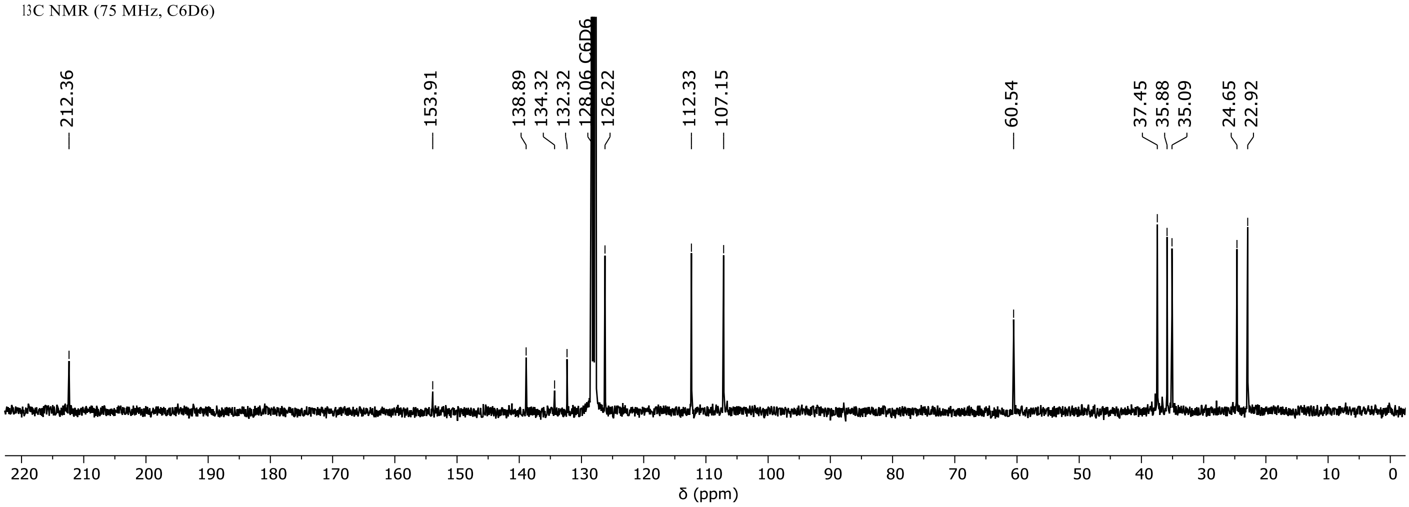

1H NMR (300 MHz, CDCl3)

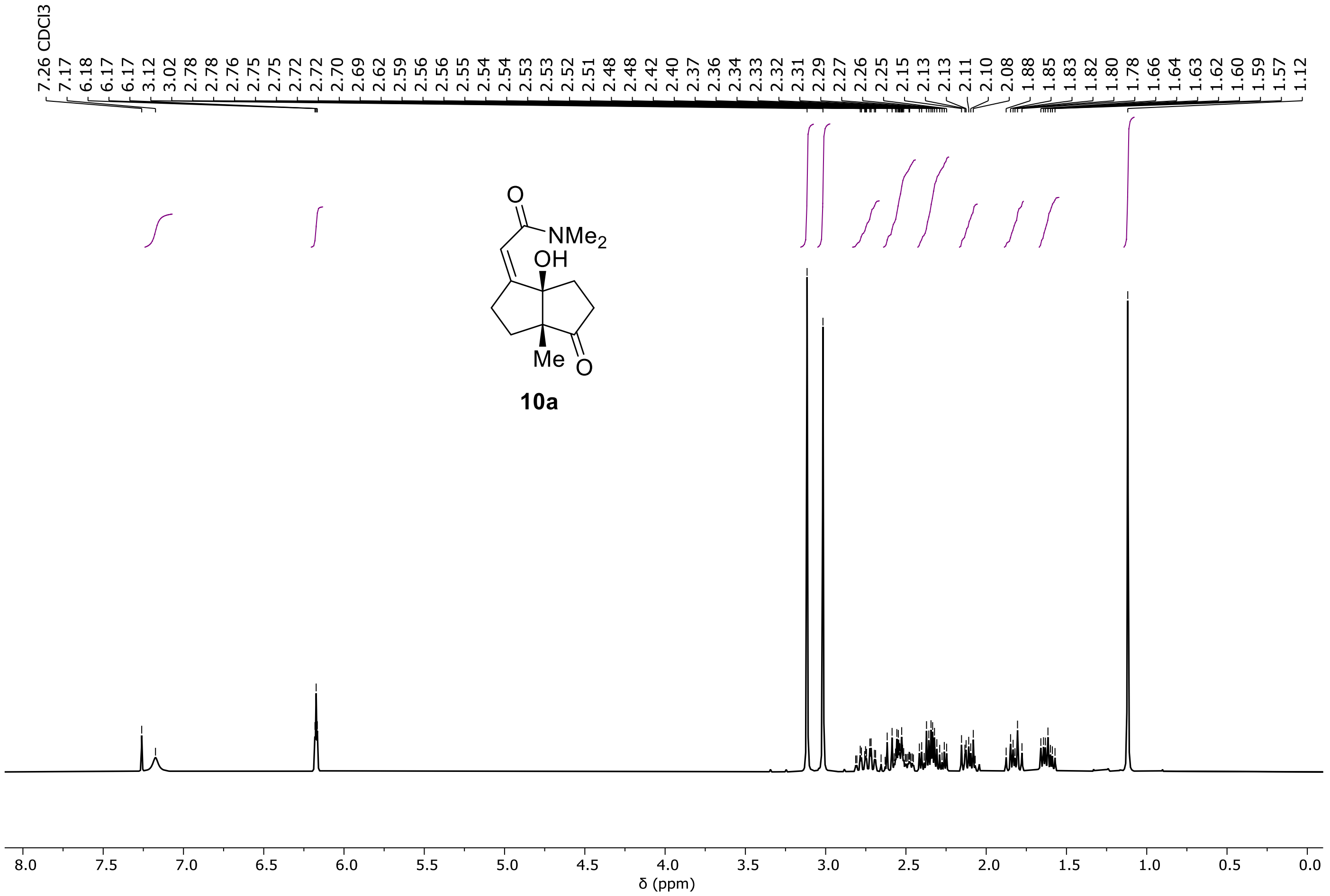

DEPT-135 NMR (75 MHz, CDCl3)

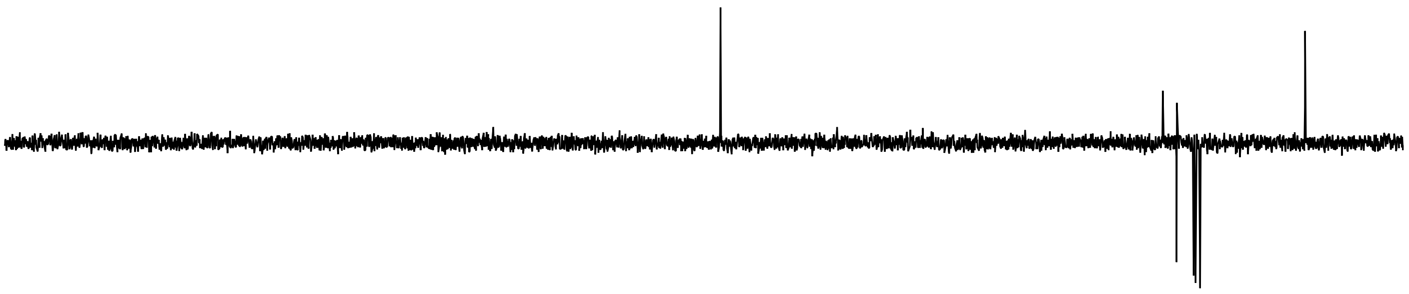

13C NMR (75 MHz, CDCl3)

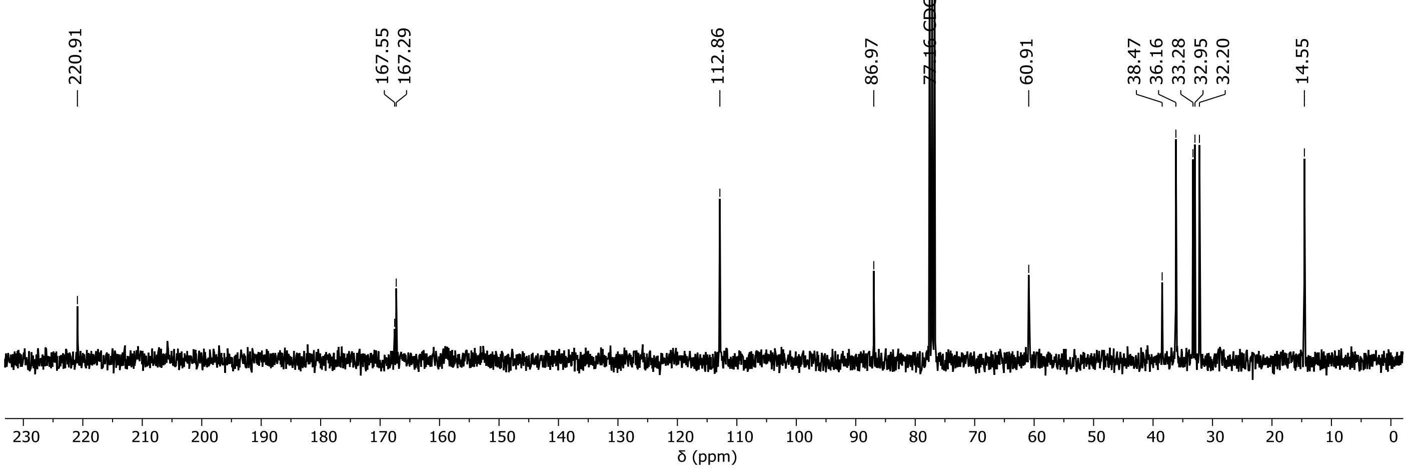

1H NMR (500 MHz, CDCl3)

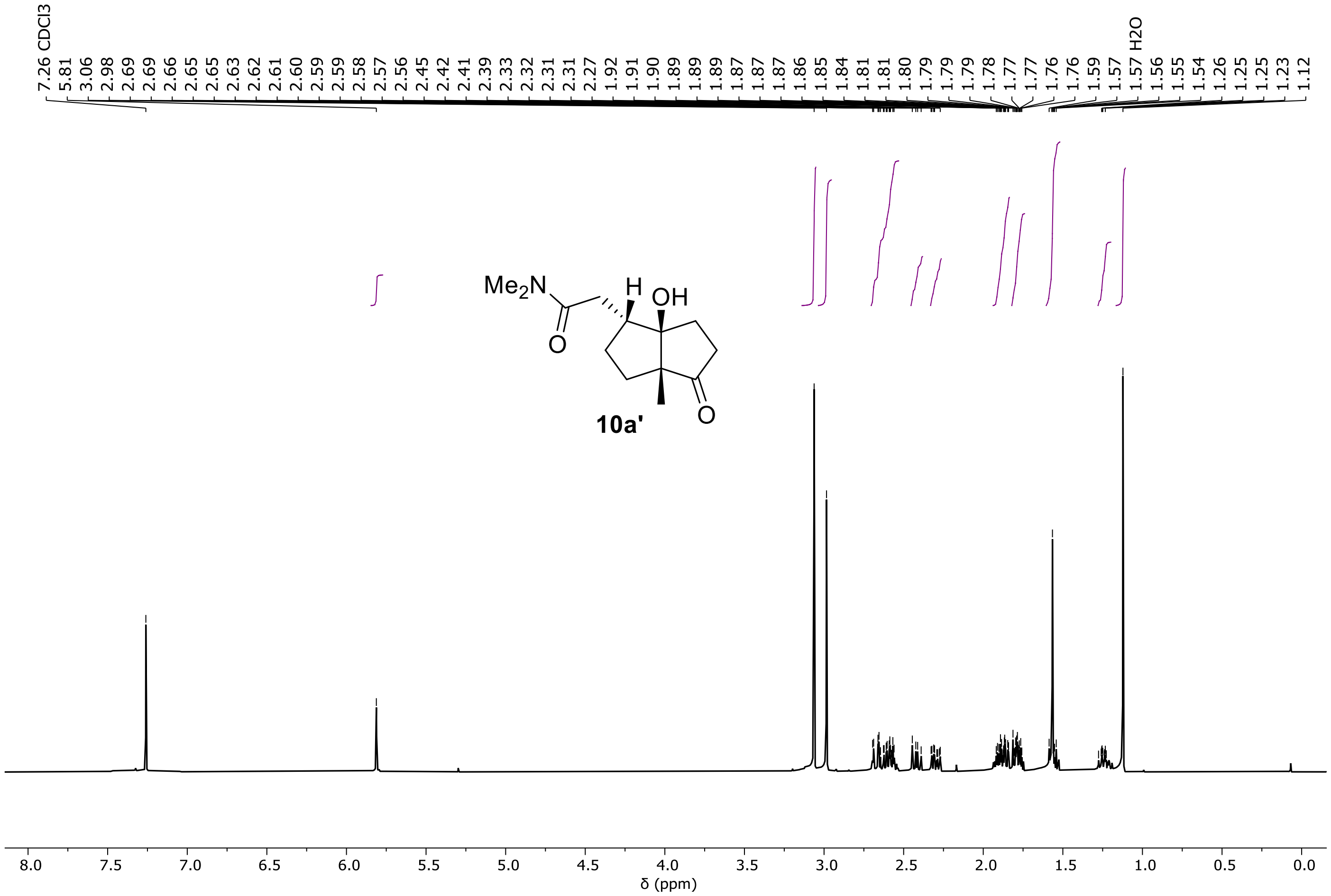

DEPT-135 NMR (126 MHz, CDCl3)

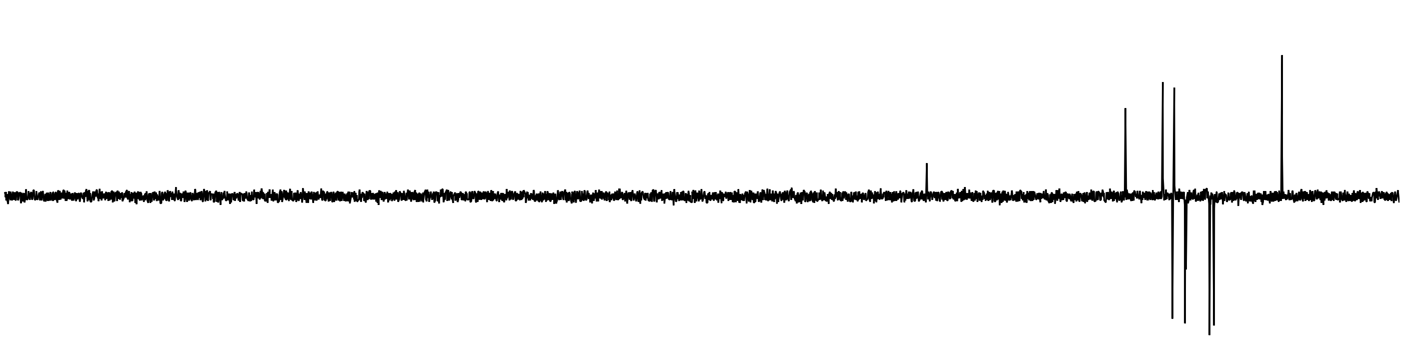

13C NMR (126 MHz, CDCl3)

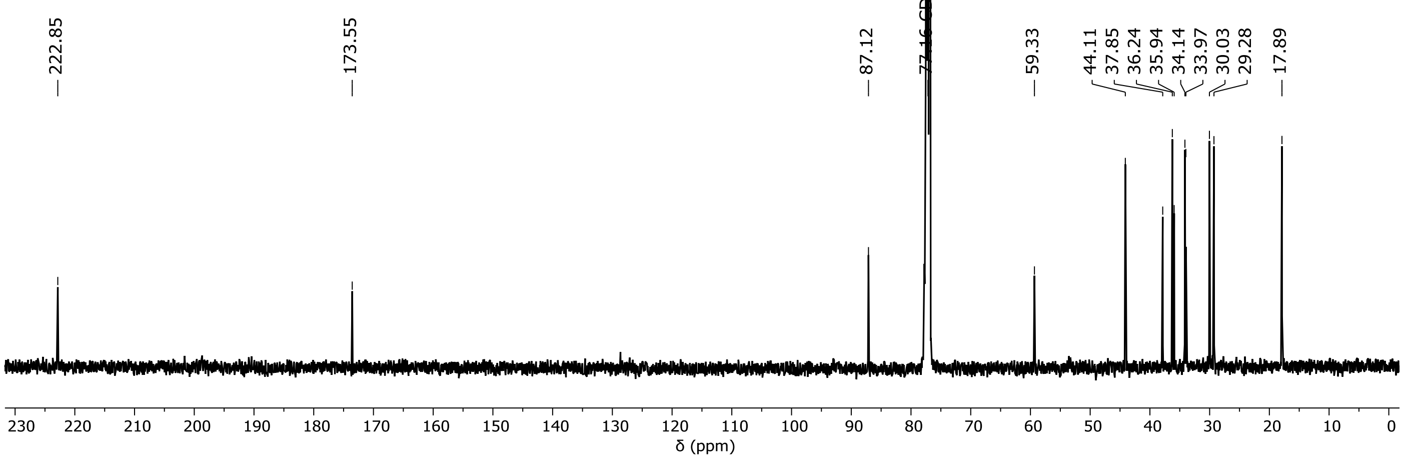

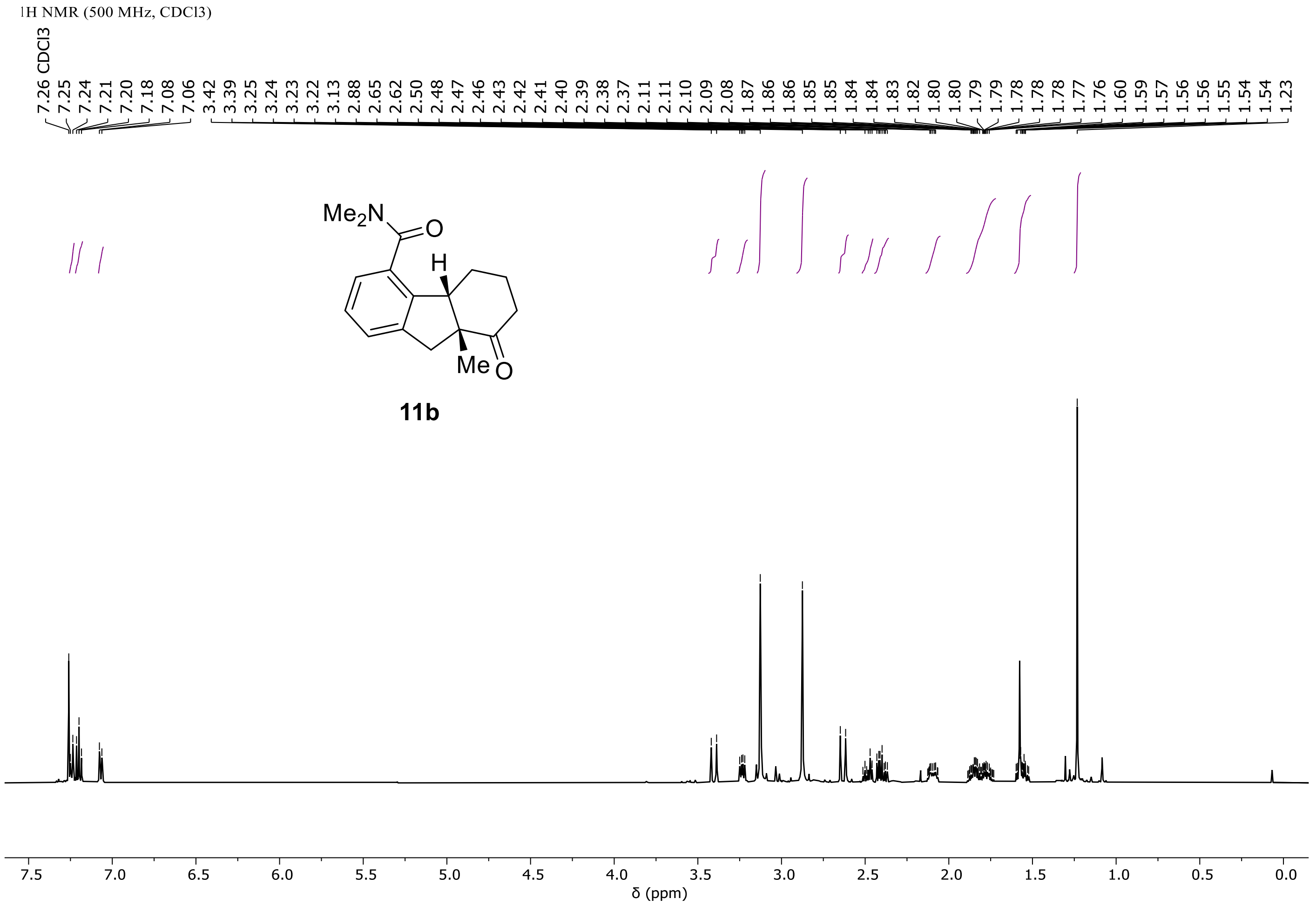
1H NMR (500 MHz, CDCl3)
7.26 CDCl3
7.25
7.24
7.21
7.20
7.18
7.08
7.06
3.42
3.39
3.25
3.24
3.23
3.22
3.13
2.88
2.65
2.62
2.50
2.48
2.47
2.46
2.43
2.42
2.41
2.40
2.39
2.38
2.37
2.11
2.11
2.10
2.09
2.08
1.87
1.86
1.86
1.85
1.85
1.84
1.84
1.83
1.82
1.80
1.79
1.79
1.78
1.78
1.78
1.77
1.76
1.60
1.59
1.57
1.56
1.56
1.55
1.54
1.54
1.23
Me2N
O
H
Me
O
11b
δ (ppm)

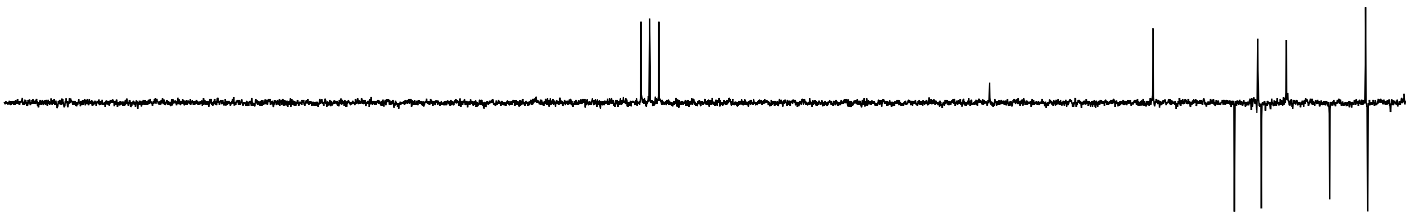

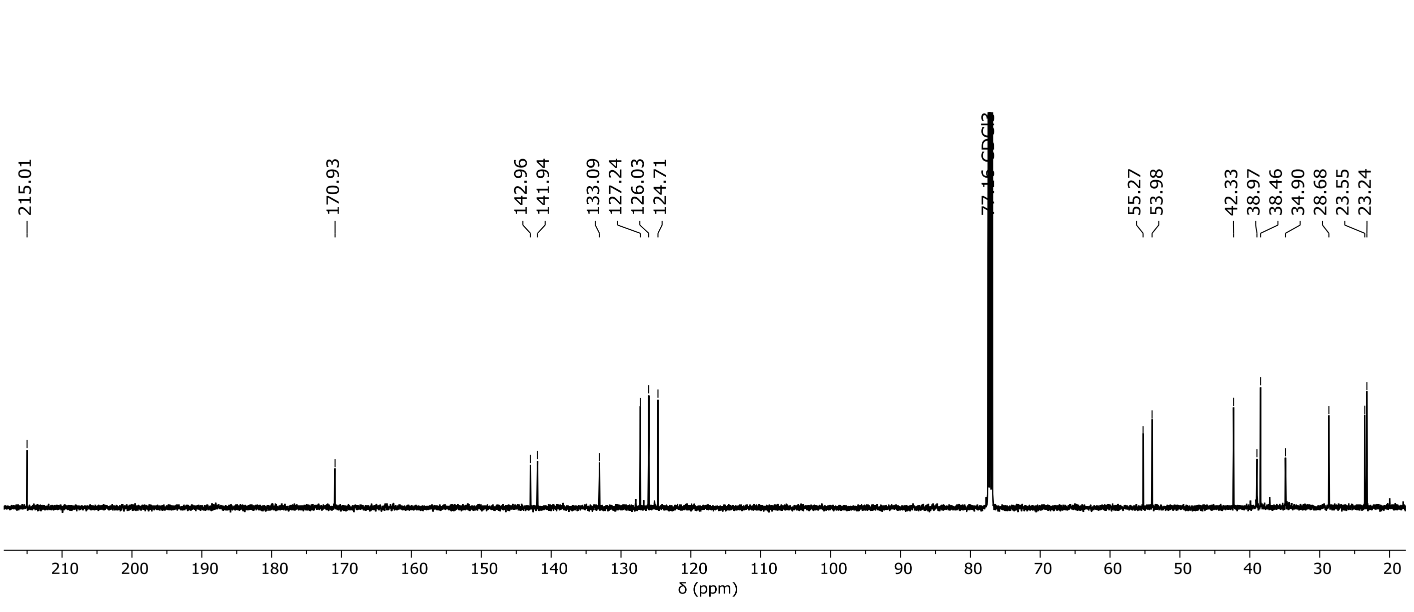
215.01
170.93
142.96
141.94
133.09
127.24
126.03
124.71
77.16 CDCl3
55.27
53.98
42.33
38.97
38.46
34.90
28.68
23.55
23.24
δ (ppm)

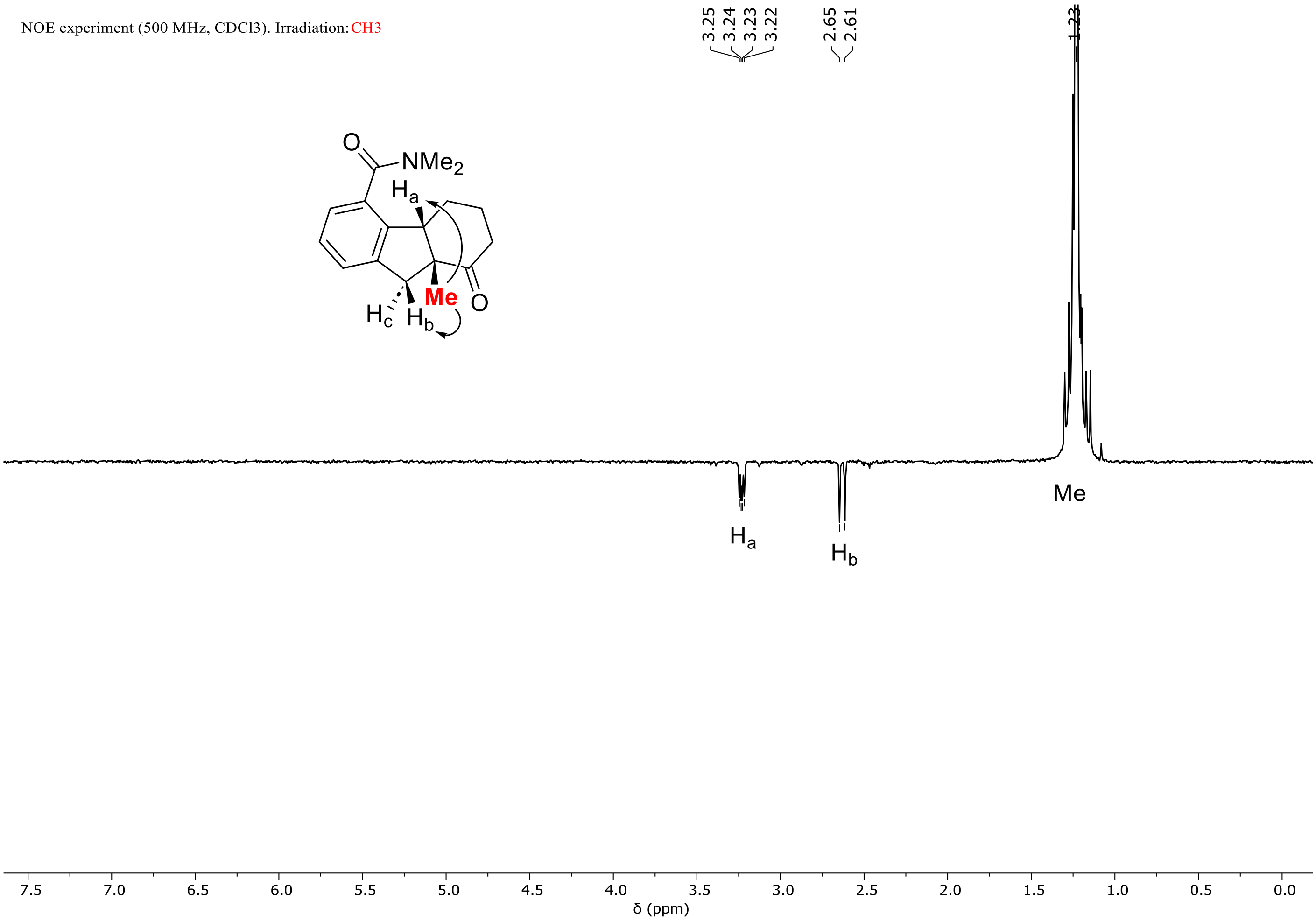
NOE experiment (500 MHz, CDCl3). Irradiation: CH3
3.25
3.24
3.23
3.22
2.65
2.61
1.23
O
NMe2
Ha
Me
O
Hc
Hb
Ha
Hb
Me
7.5
7.0
6.5
6.0
5.5
5.0
4.5
4.0
3.5
3.0
2.5
2.0
1.5
1.0
0.5
0.0
δ (ppm)

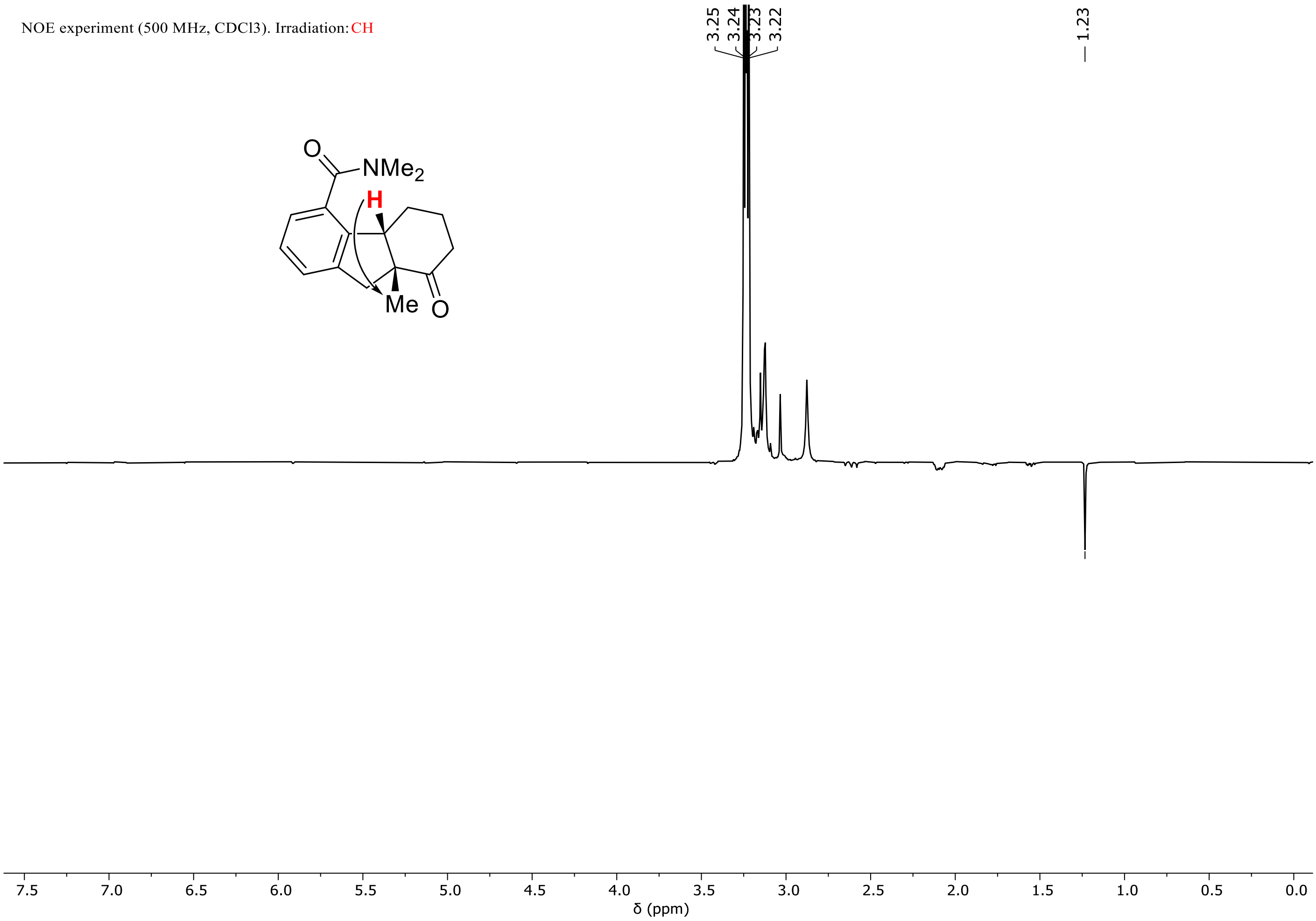
NOE experiment (500 MHz, CDCl3). Irradiation: CH
3.25
3.24
3.23
3.22
1.23
O
NMe2
H
Me
O
7.5
7.0
6.5
6.0
5.5
5.0
4.5
4.0
3.5
3.0
2.5
2.0
1.5
1.0
0.5
0.0
δ (ppm)

1H NMR (300 MHz, CDCl3)

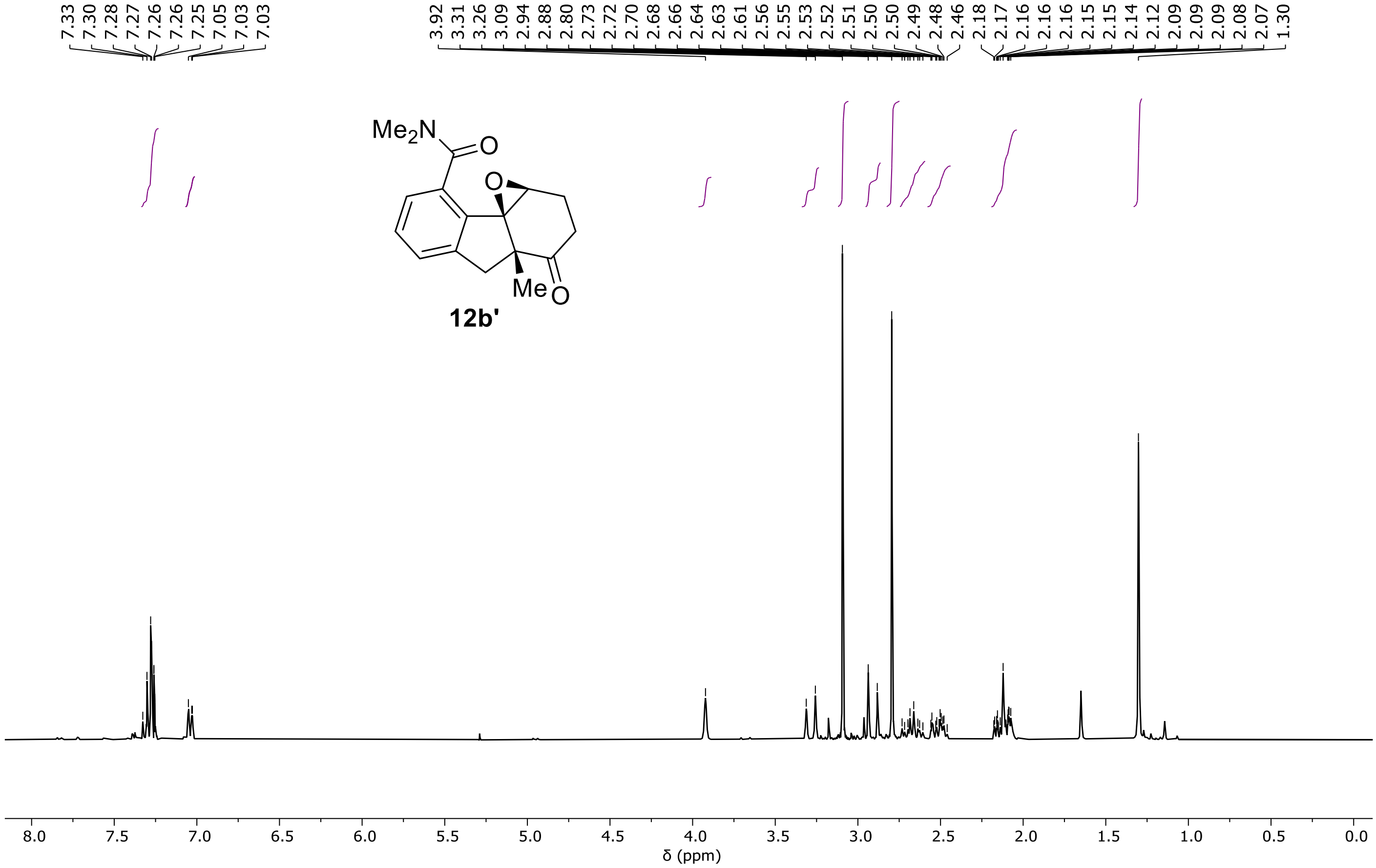

DEPT-135 NMR (75 MHz, CDCl3)

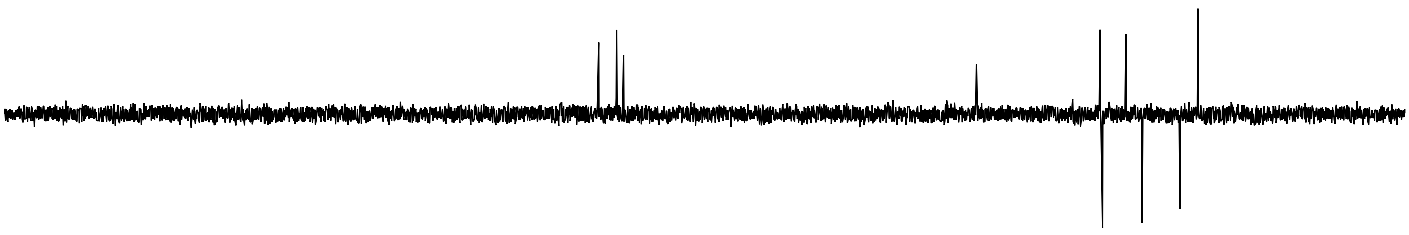

13C NMR (75 MHz, CDCl3)

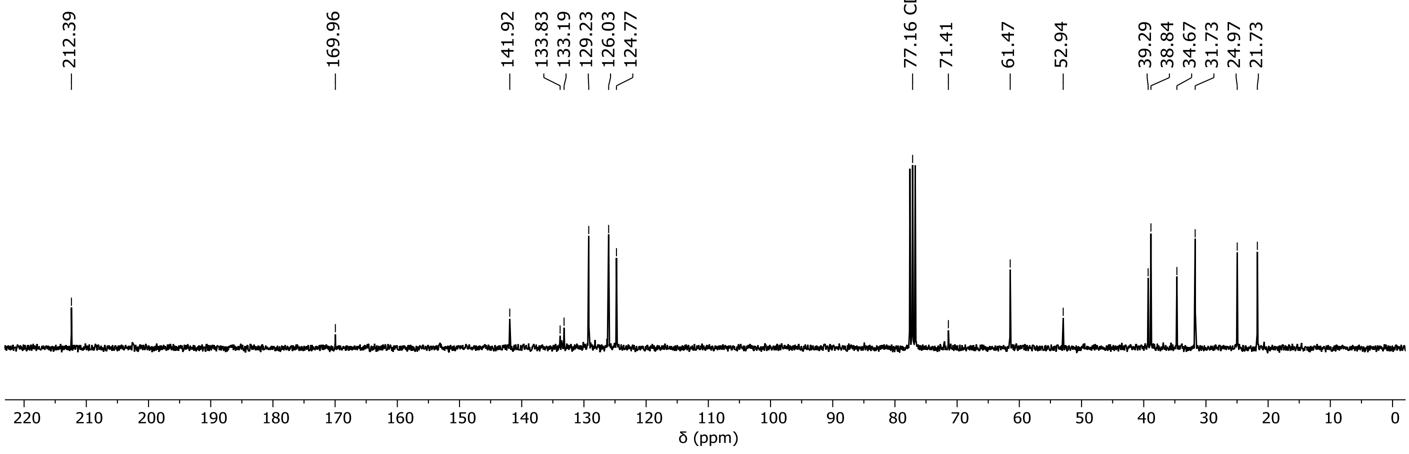

1H NMR (500 MHz, CDCl3)

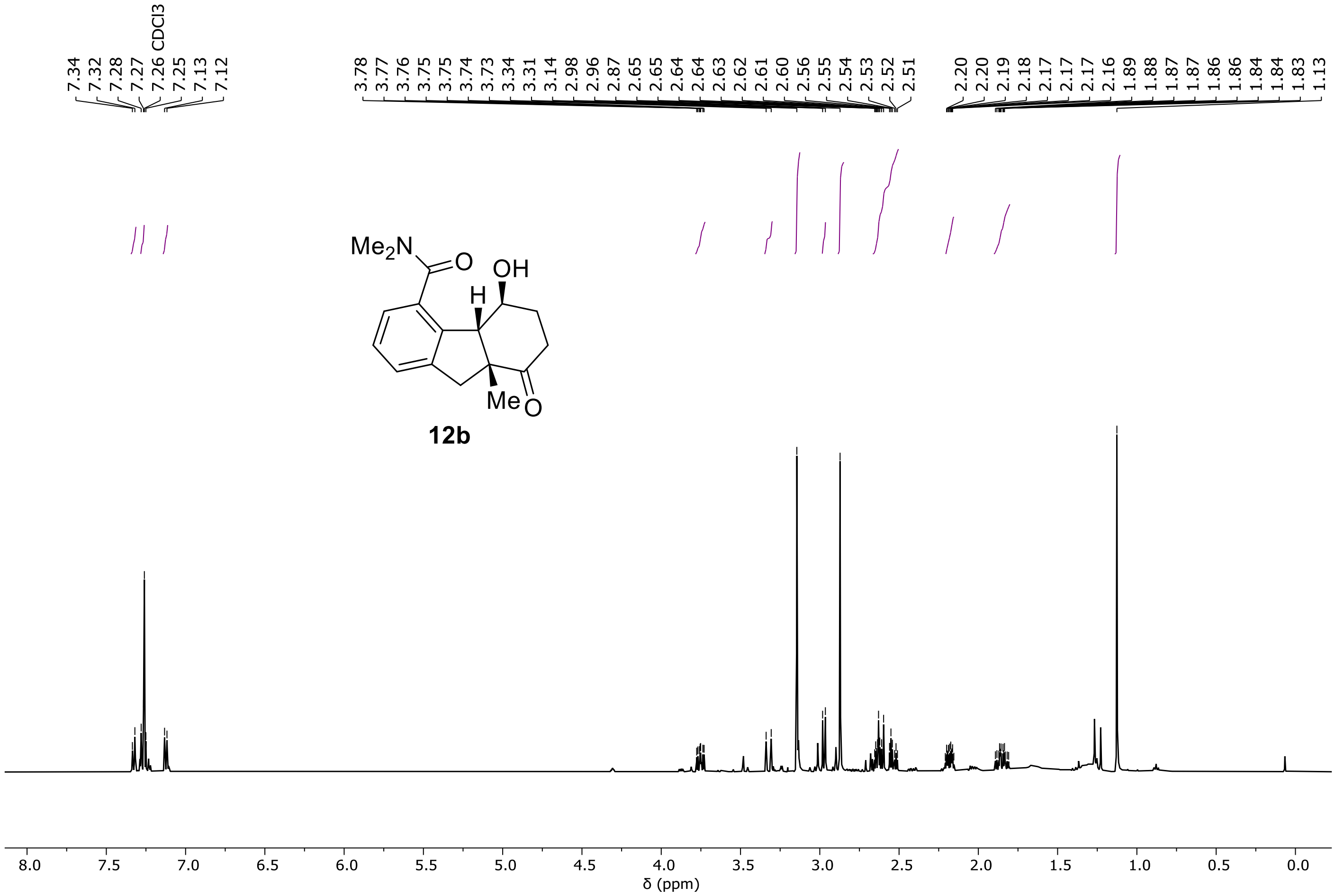

DEPT-135 NMR (126 MHz, CDCl3)

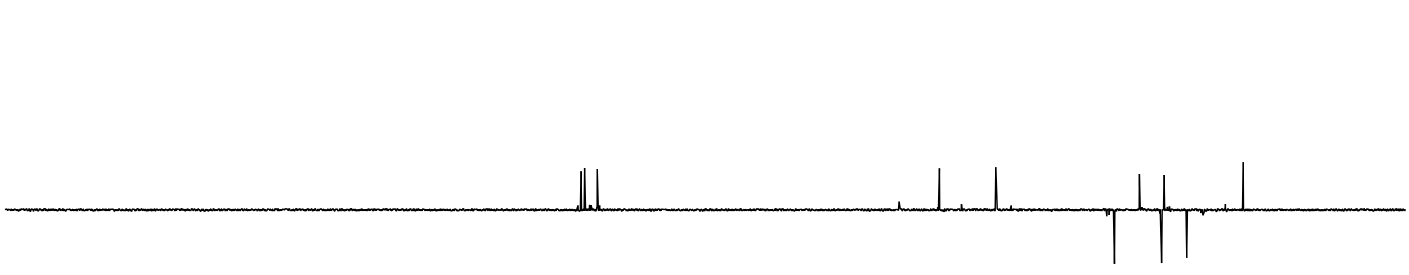

13C NMR (126 MHz, CDCl3)

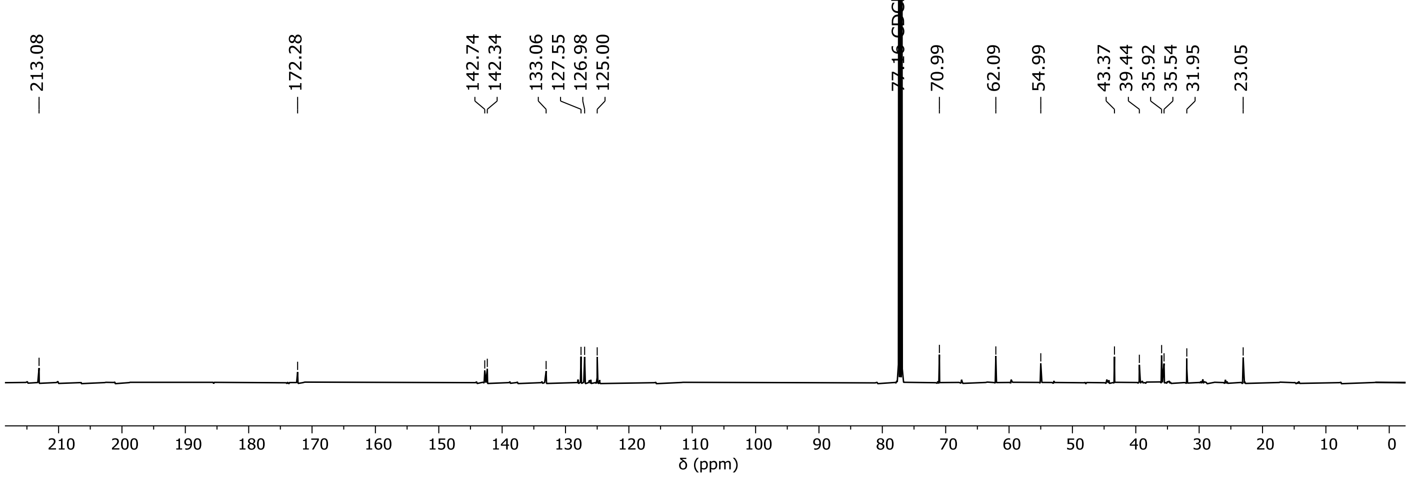

NOE experiment (500 MHz, CDCl3). Irradiation: CH3

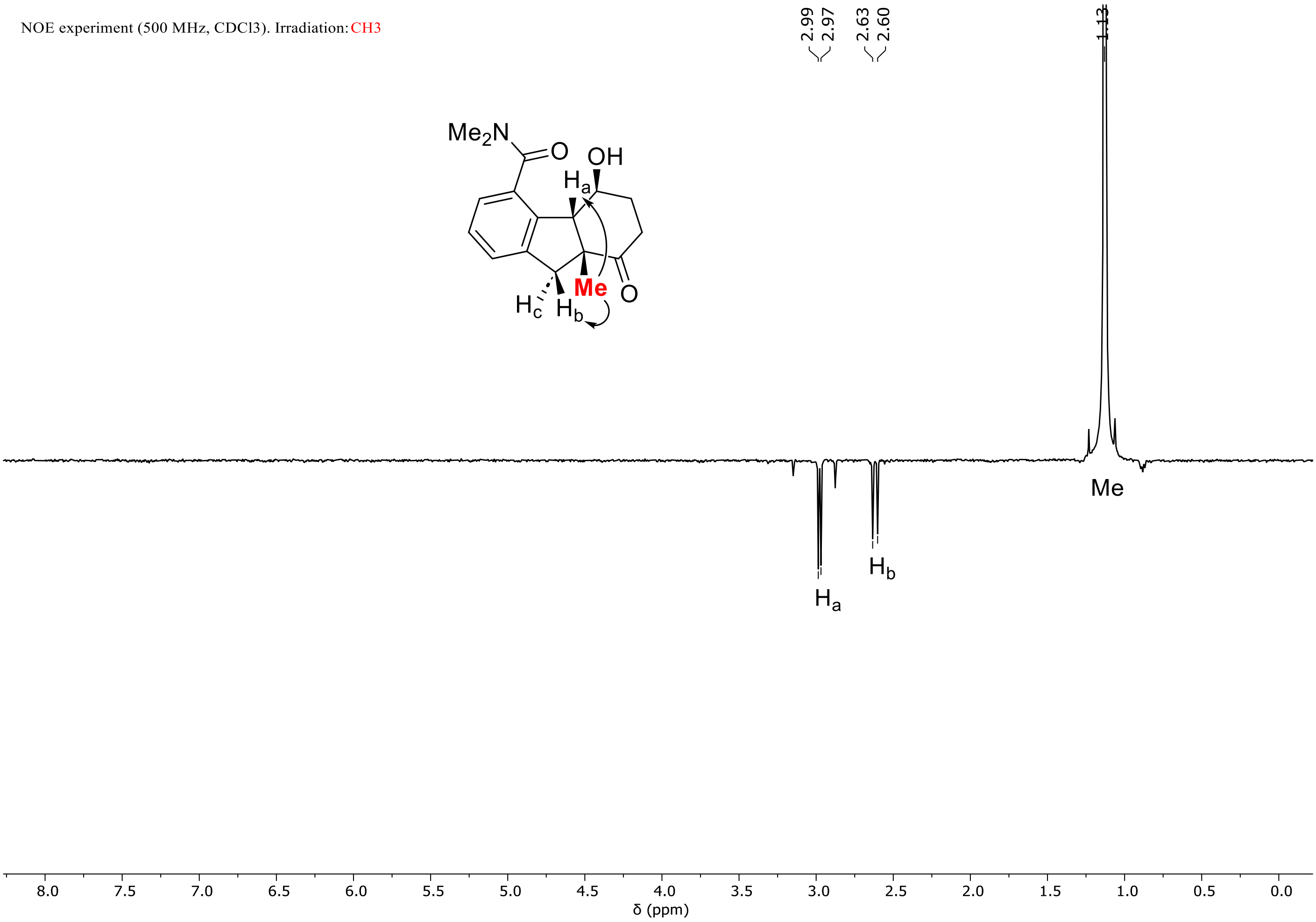

NOE experiment (500 MHz, CDCl3). Irradiation: CH

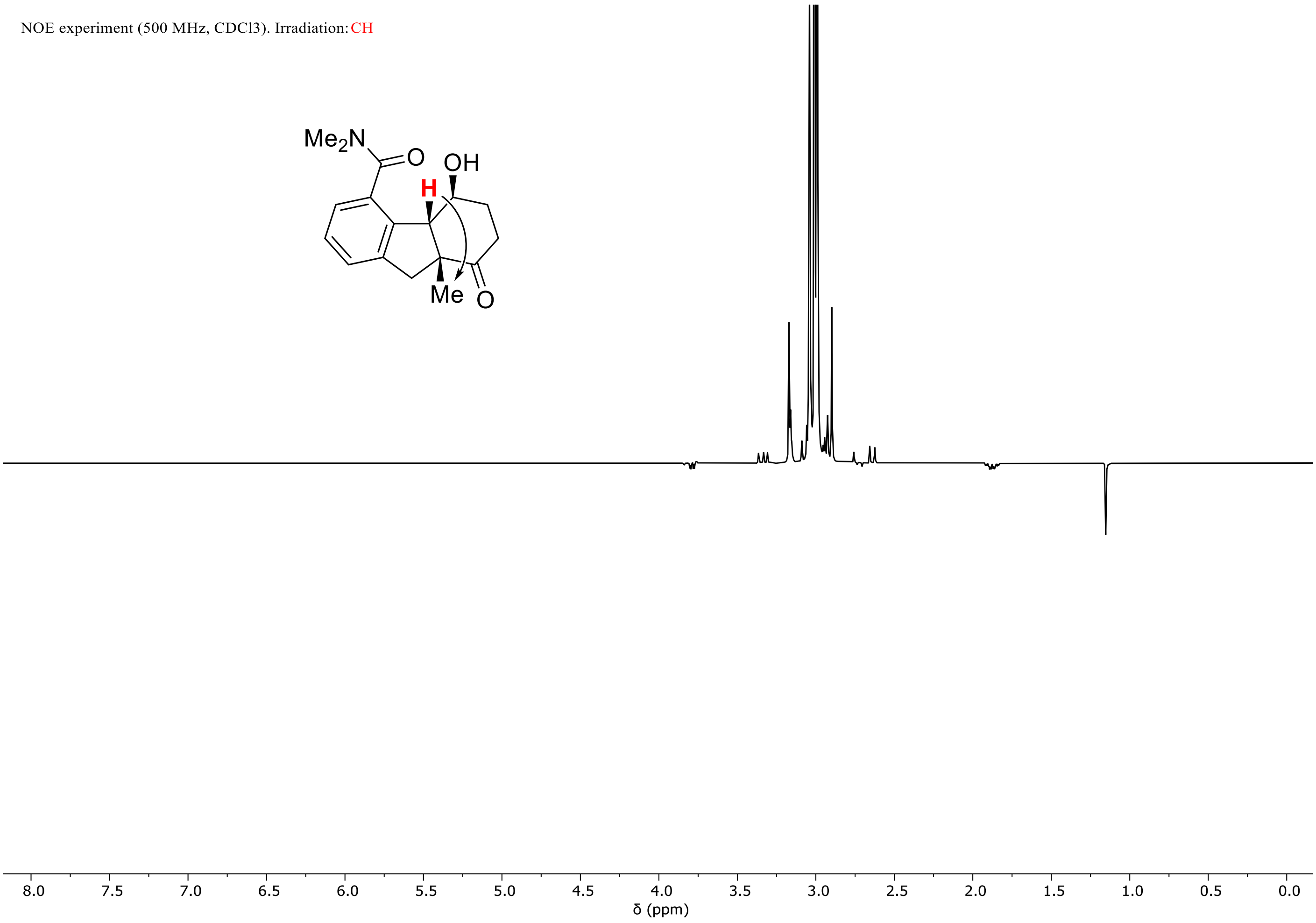

NOE experiment (500 MHz, CDCl3). Irradiation: CHOH

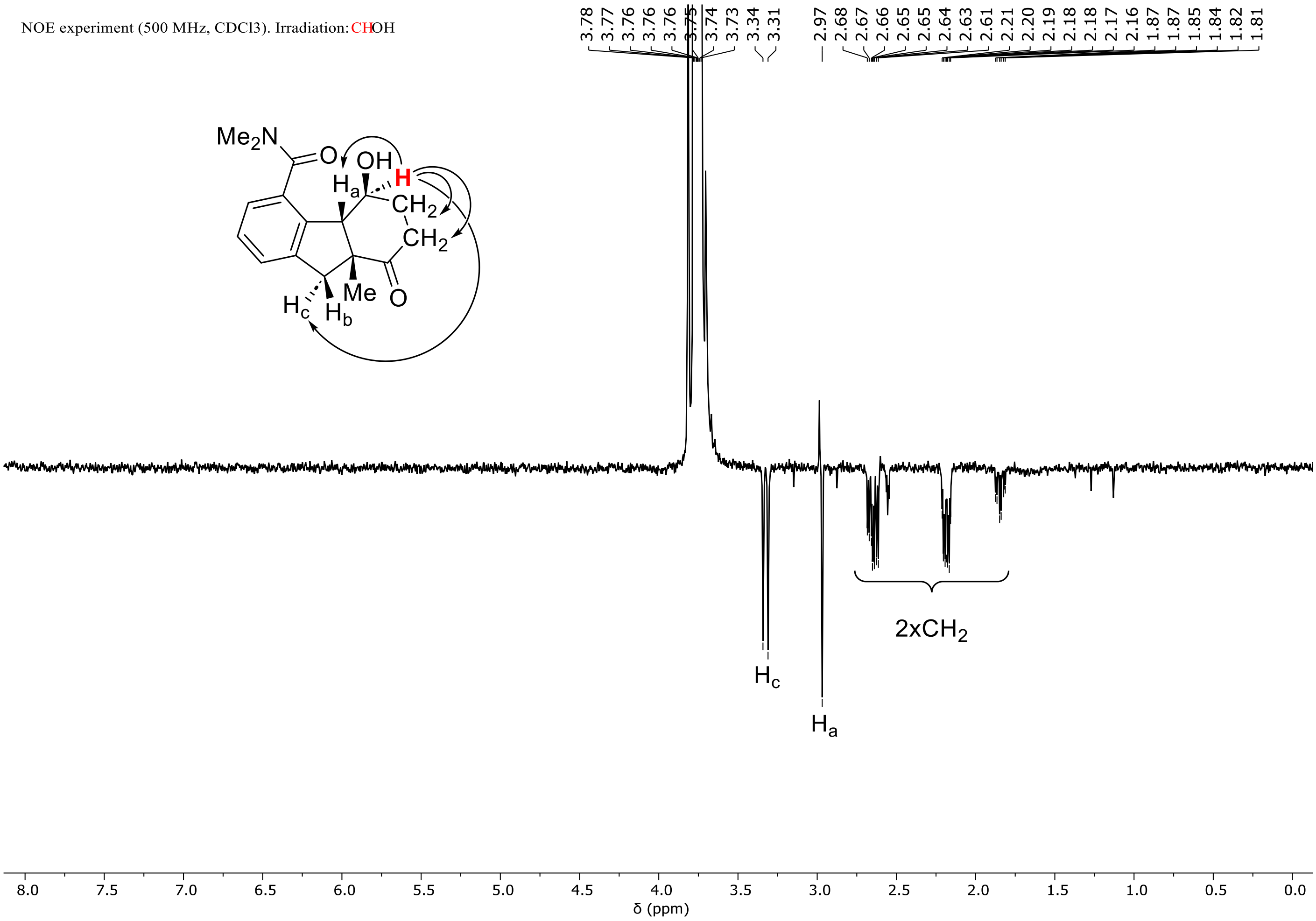

1H NMR (500 MHz, CDCl3)

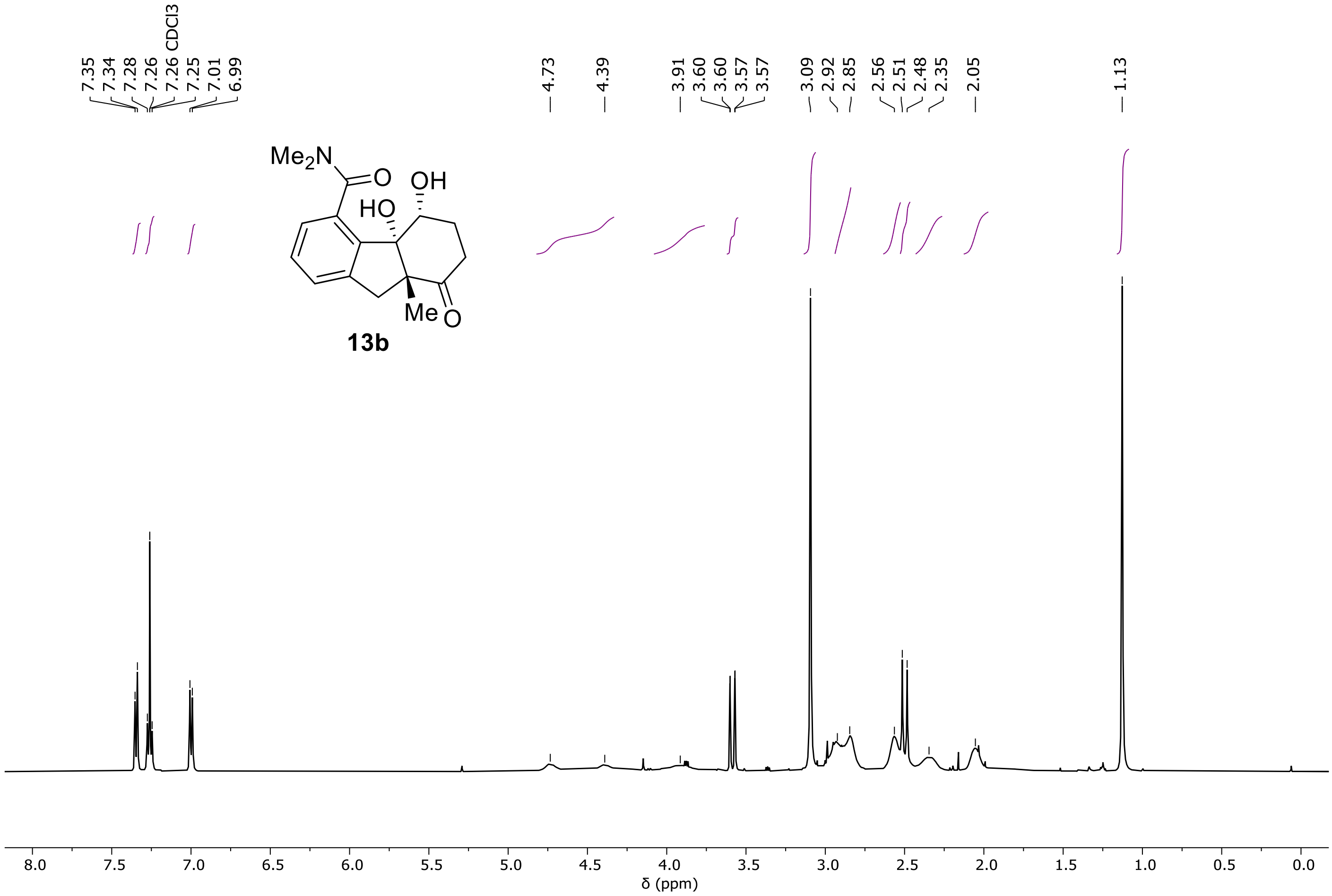

DEPT-135 NMR (126 MHz, CDCl3)

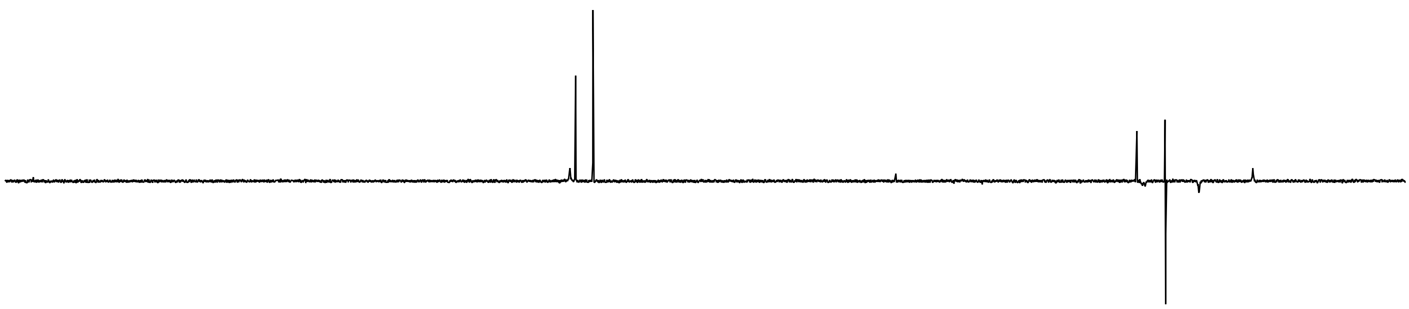

13C NMR (126 MHz, CDCl3)

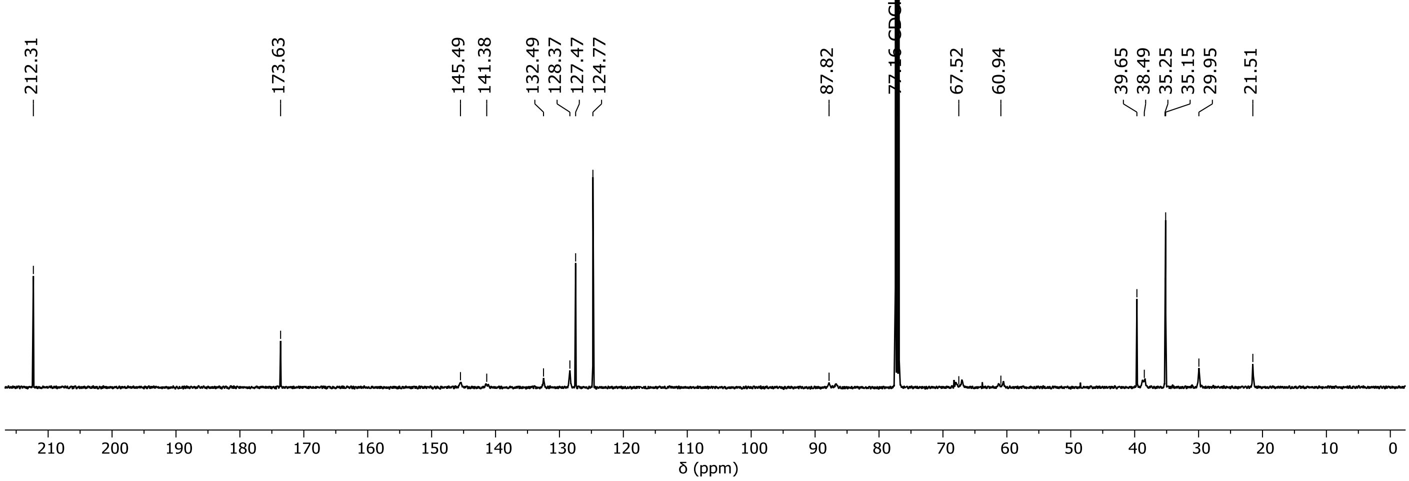

1H NMR (300 MHz, CDCl3)

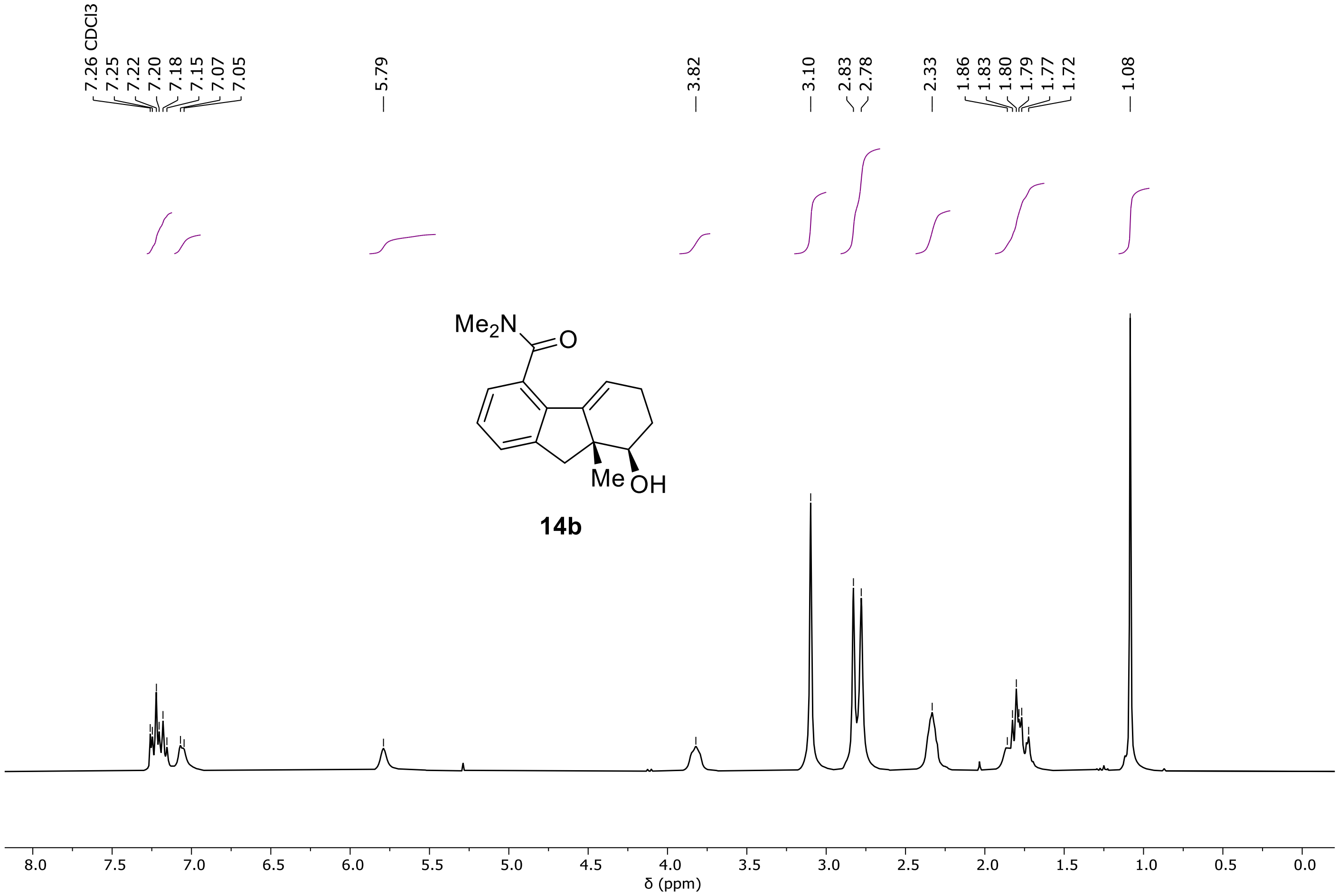

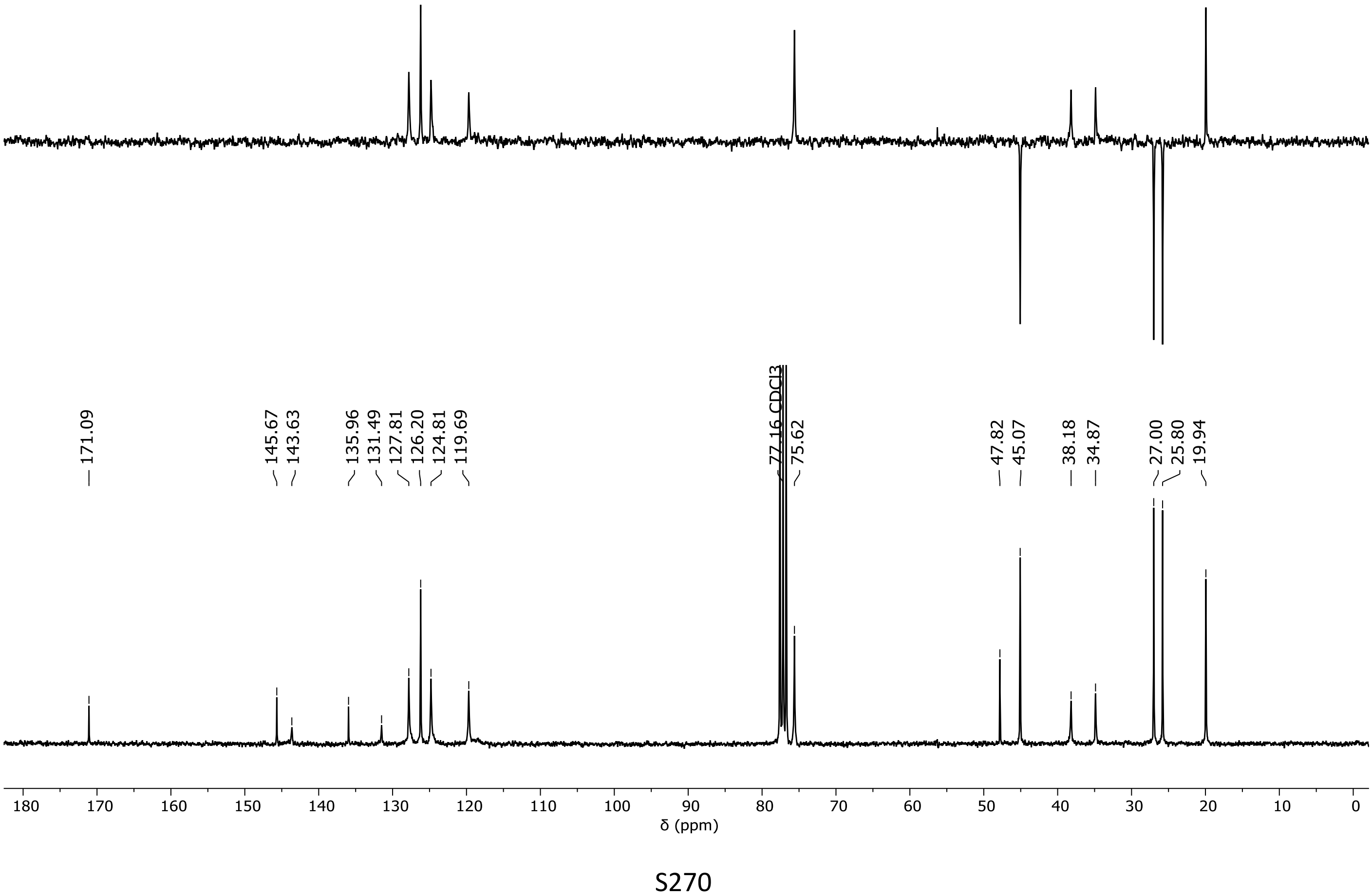

1H NMR (300 MHz, CDCl3)

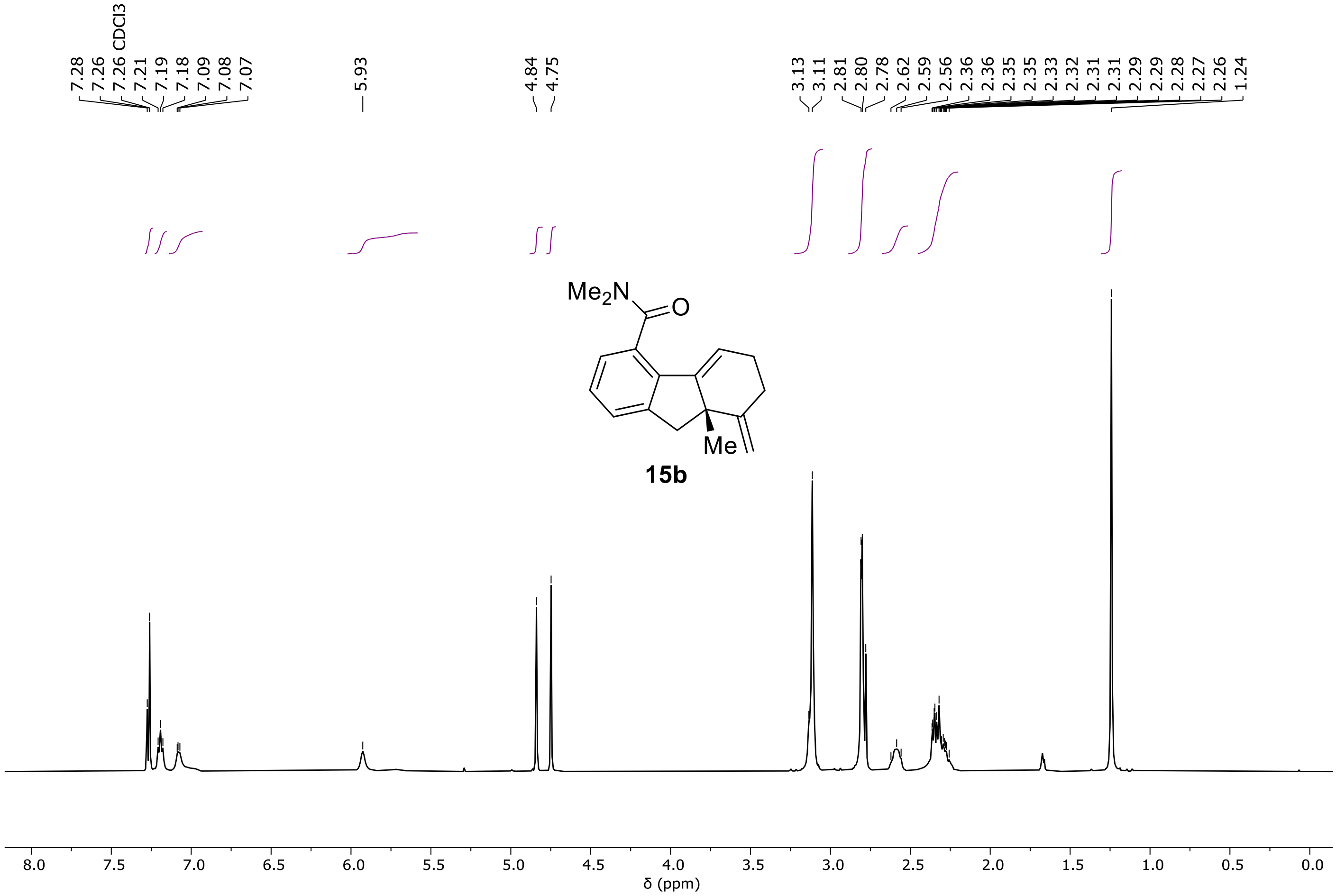

DEPT-135 NMR (75 MHz, CDCl3)

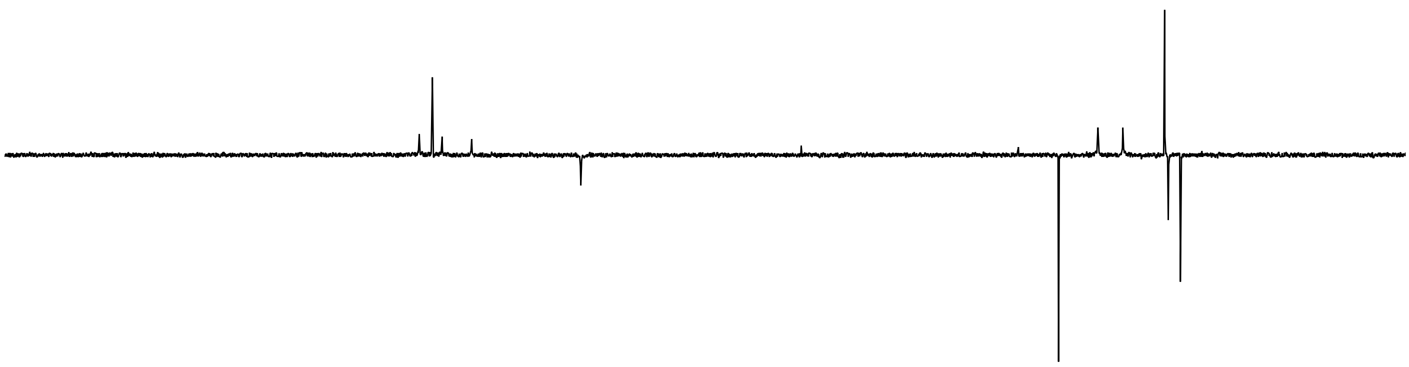

13C NMR (75 MHz, CDCl3)

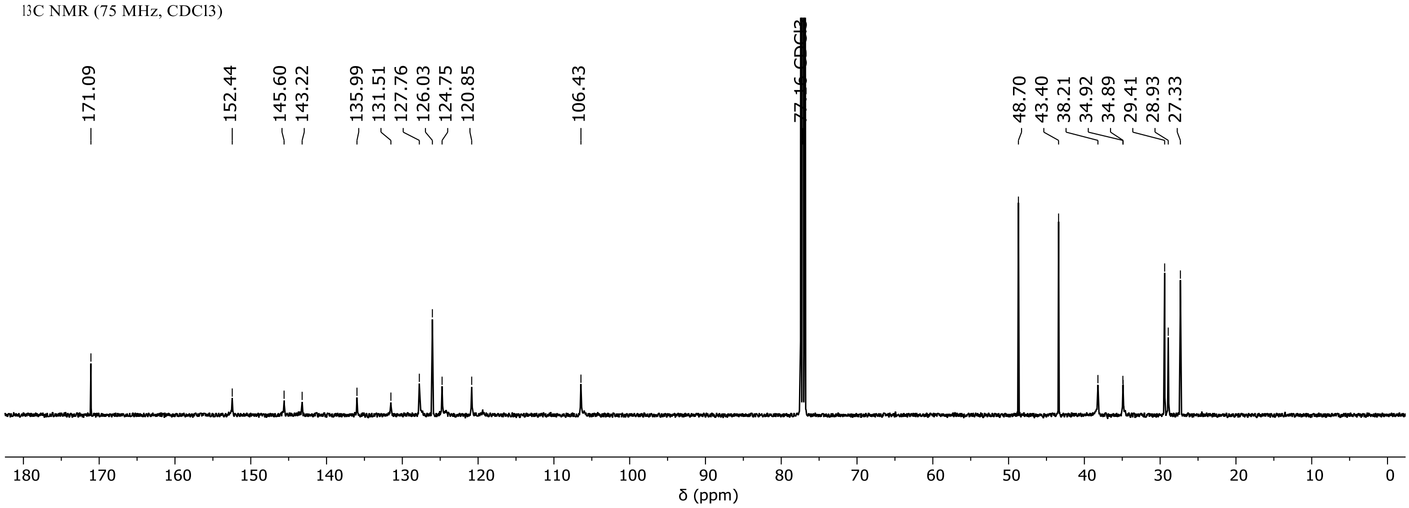

1H NMR (500 MHz, CDCl3)

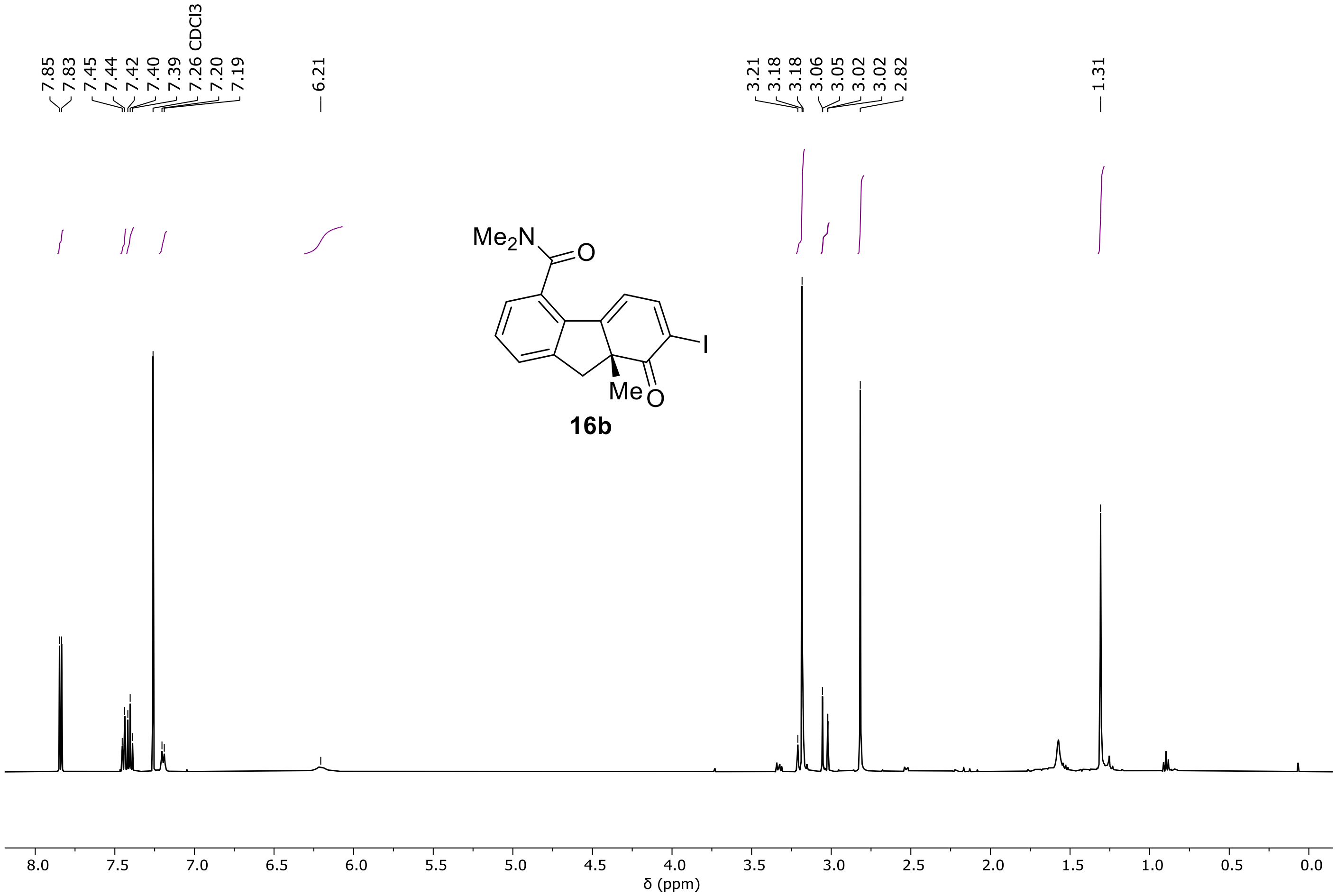

DEPT-135 NMR (126 MHz, CDCl3)

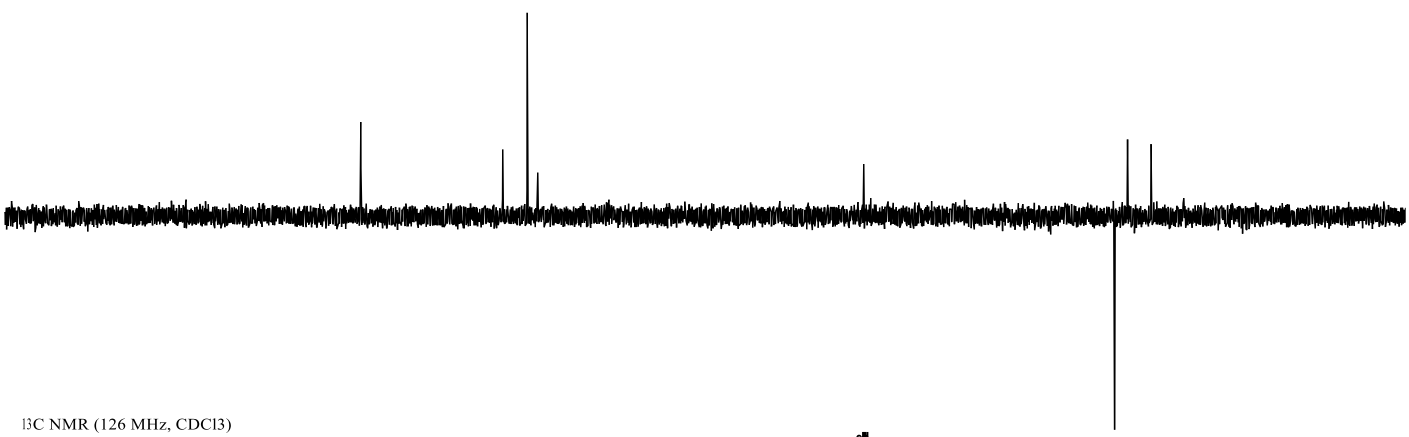

13C NMR (126 MHz, CDCl3)

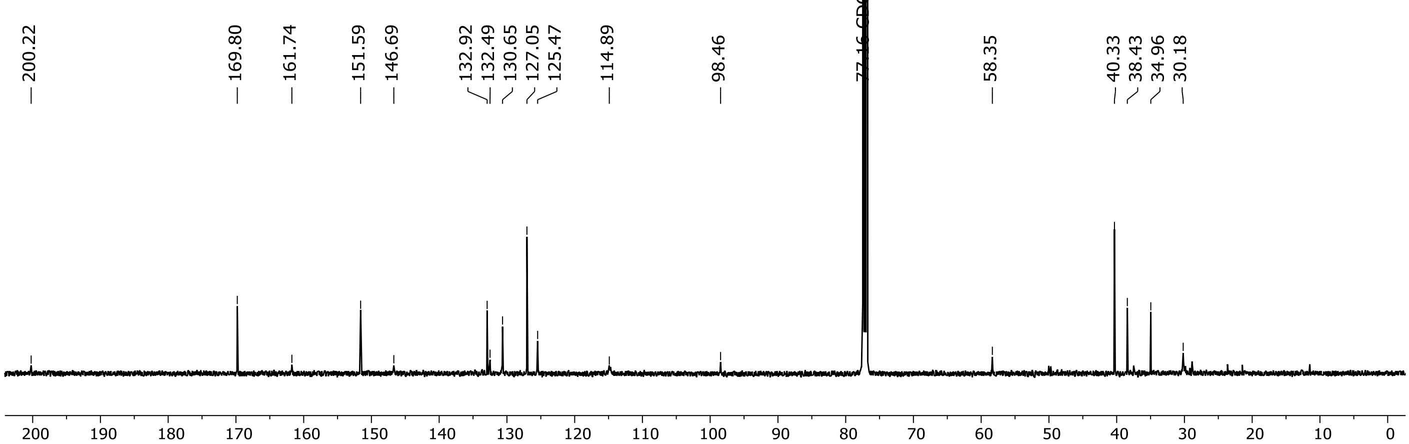

1H NMR (300 MHz, CDCl3)

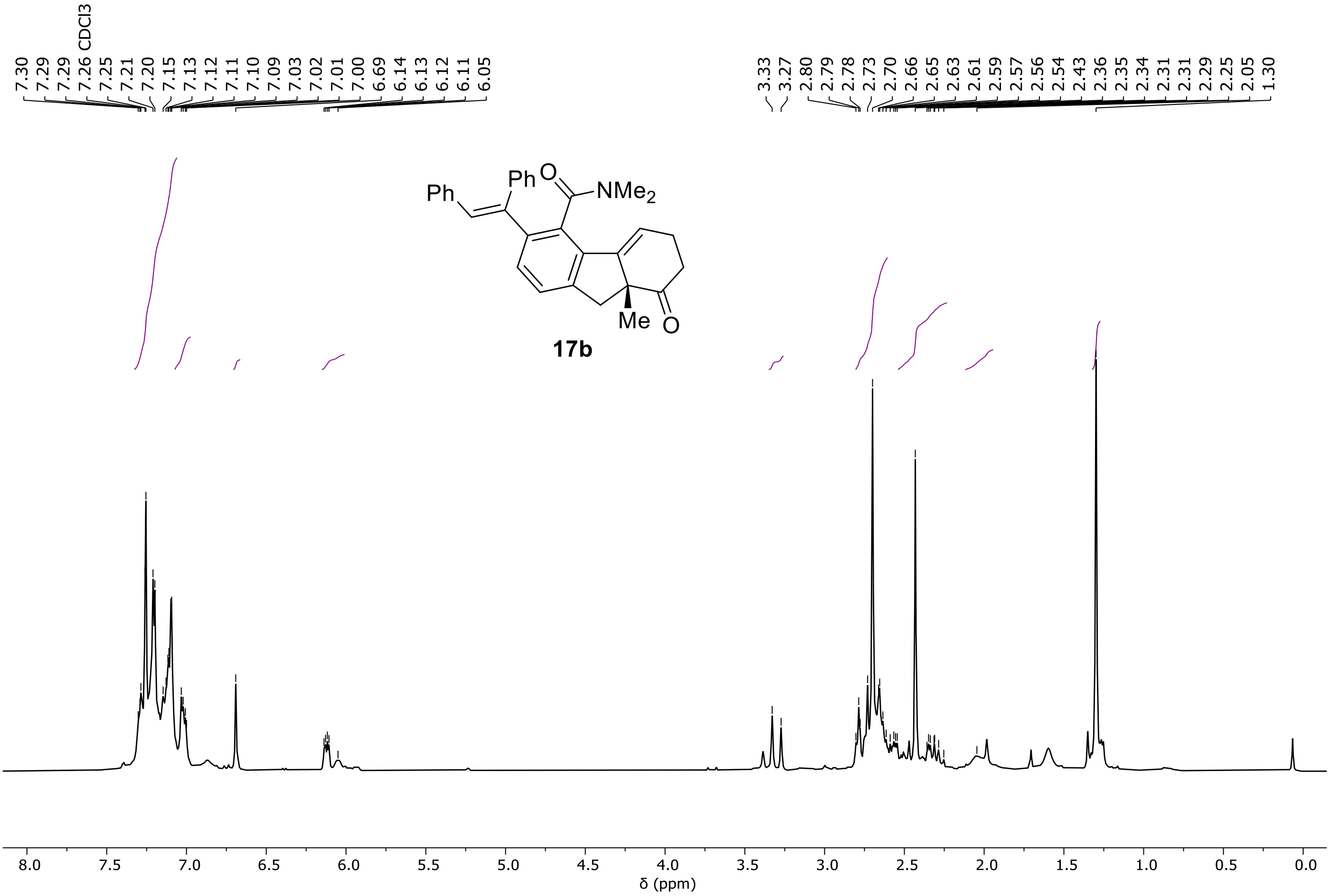

DEPT-135 NMR (75 MHz, CDCl3)

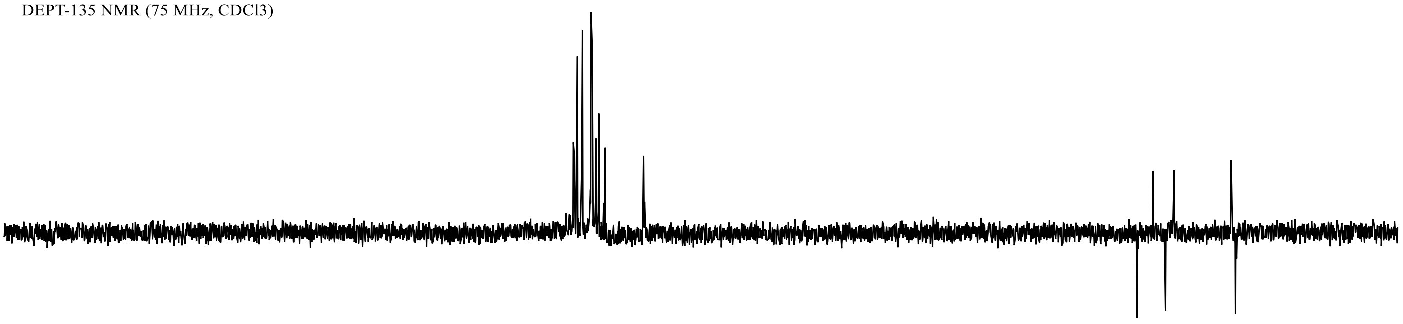

13C NMR (75 MHz, CDCl3)

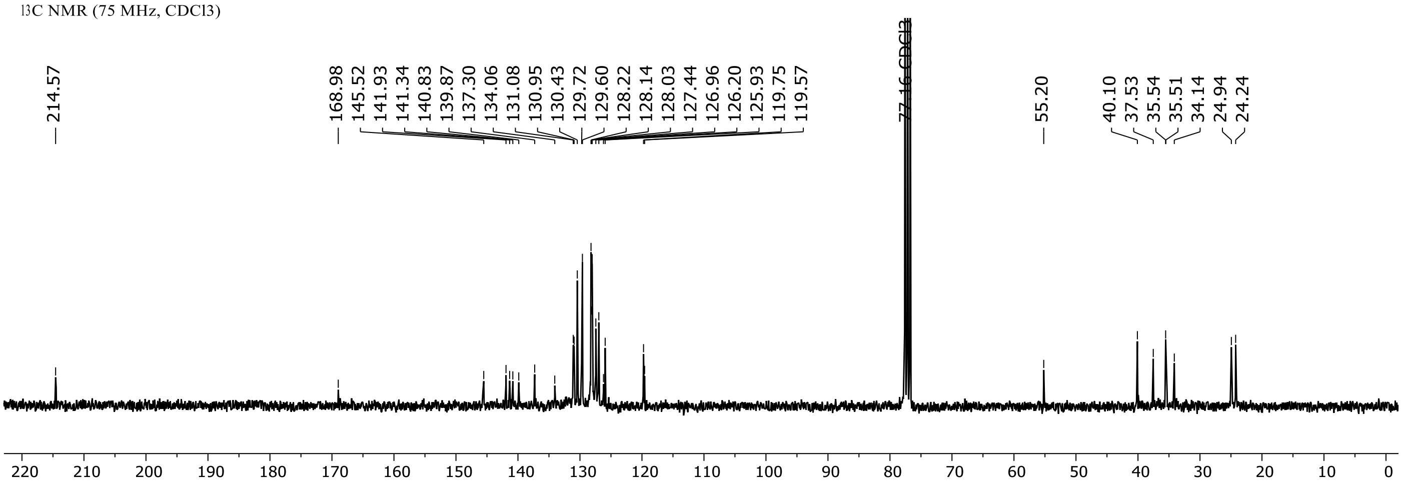

1H NMR (300 MHz, CDCl3)

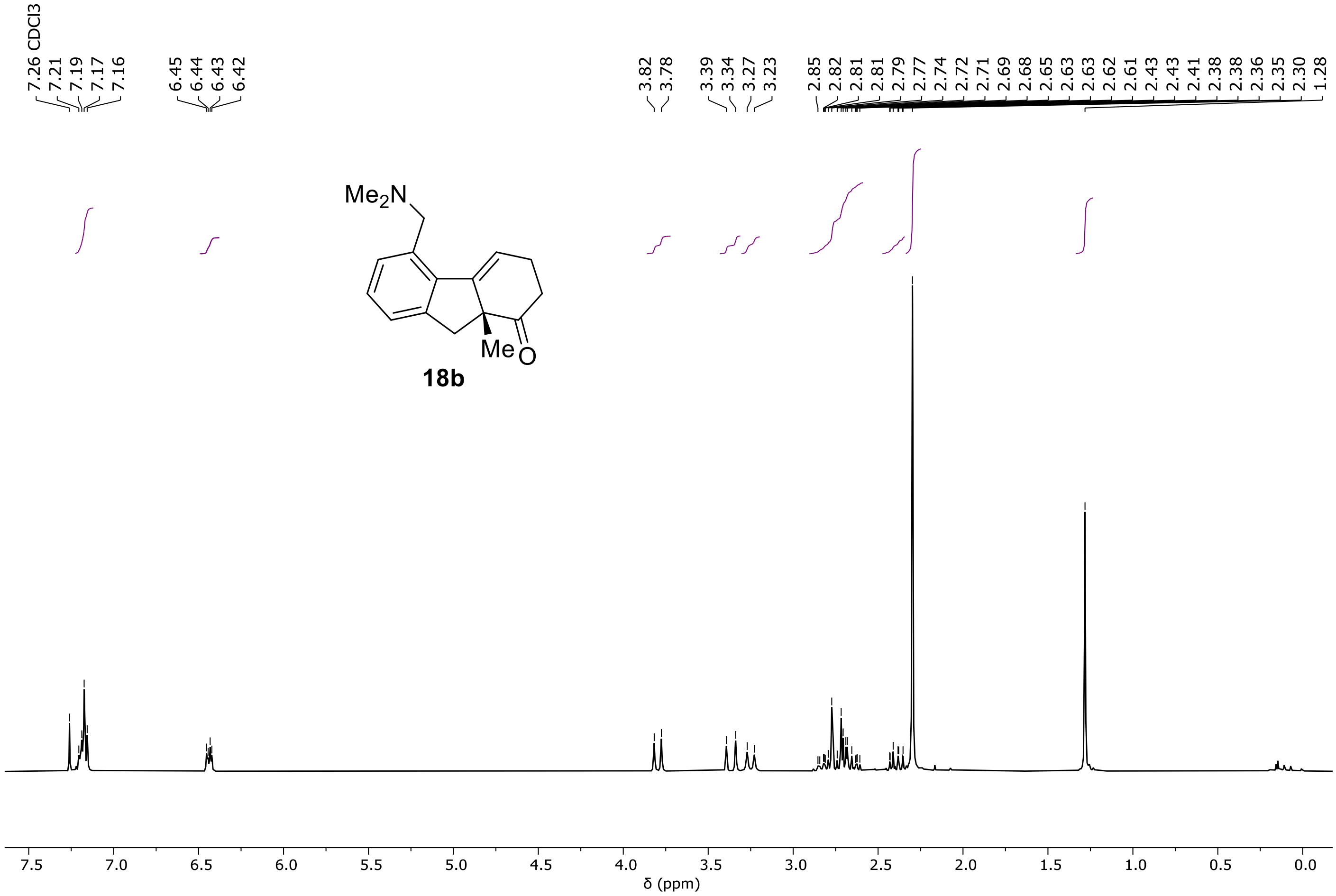

DEPT-135 NMR (75 MHz, CDCl3)

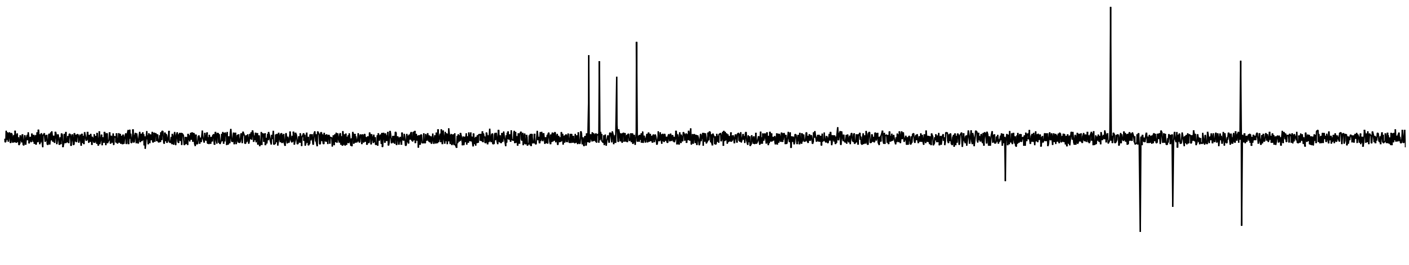

13C NMR (75 MHz, CDCl3)

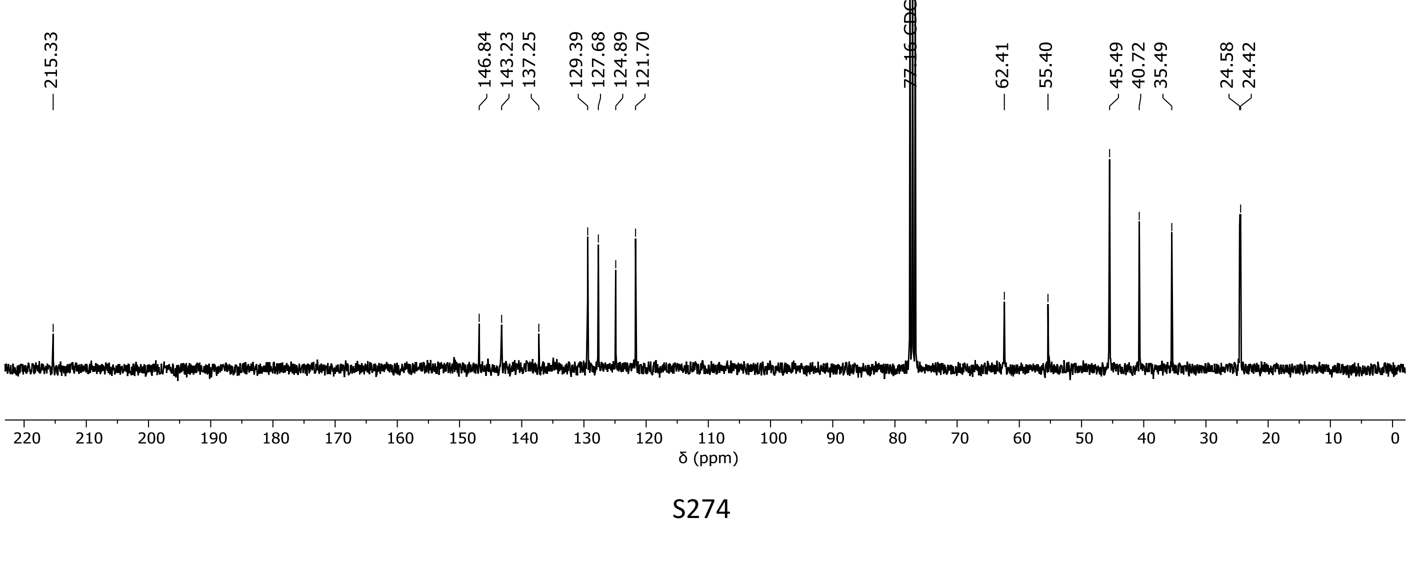

1H NMR (500 MHz, CDCl3)

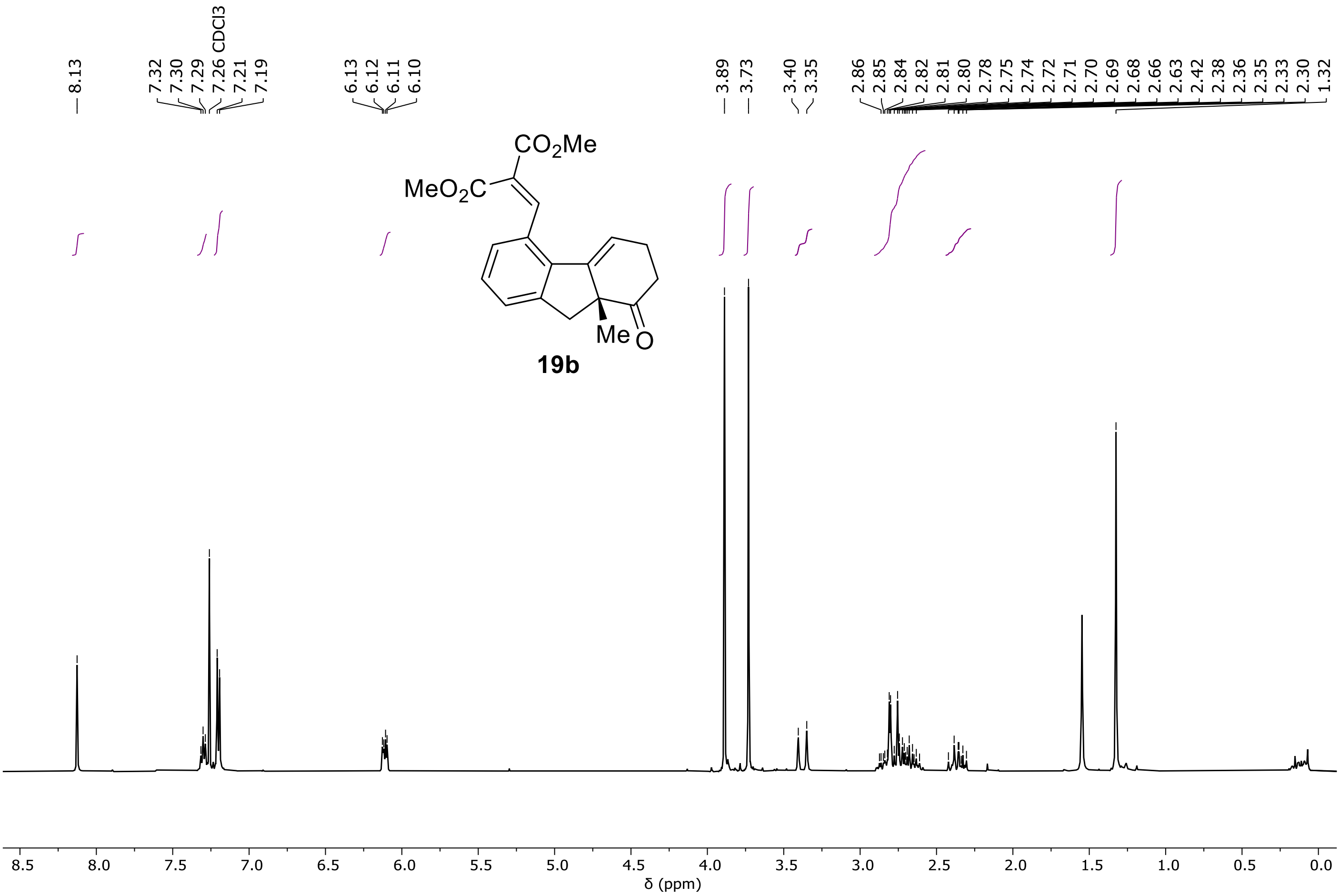

DEPT-135 NMR (126 MHz, CDCl3)

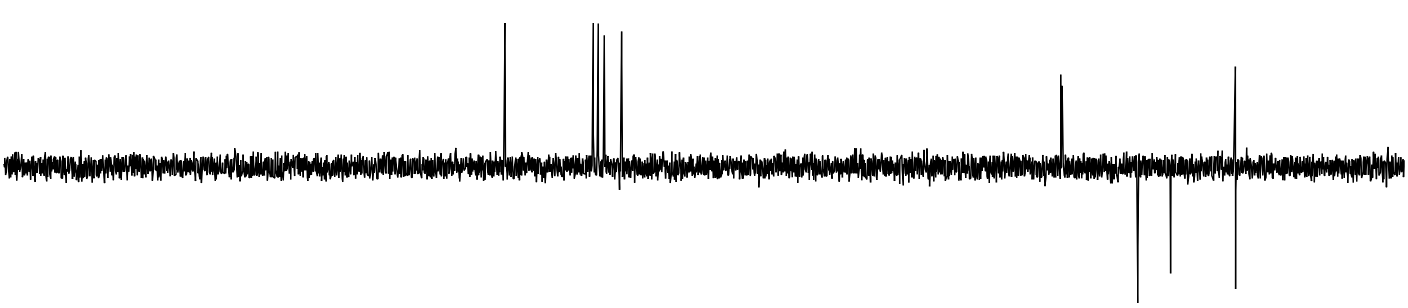

13C NMR (126 MHz, CDCl3)

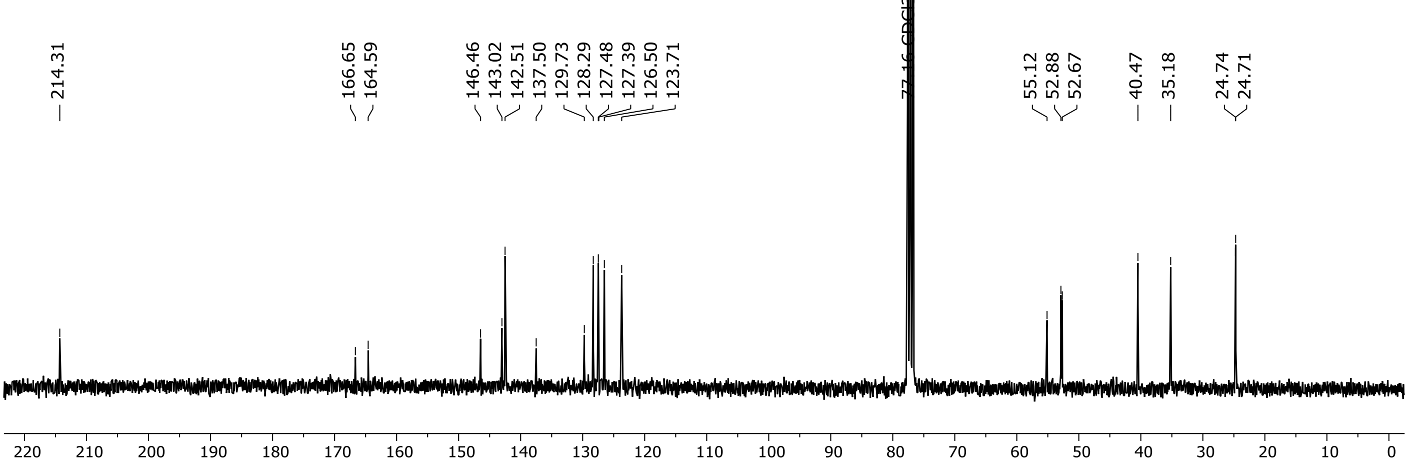

1H NMR (300 MHz, CDCl3)

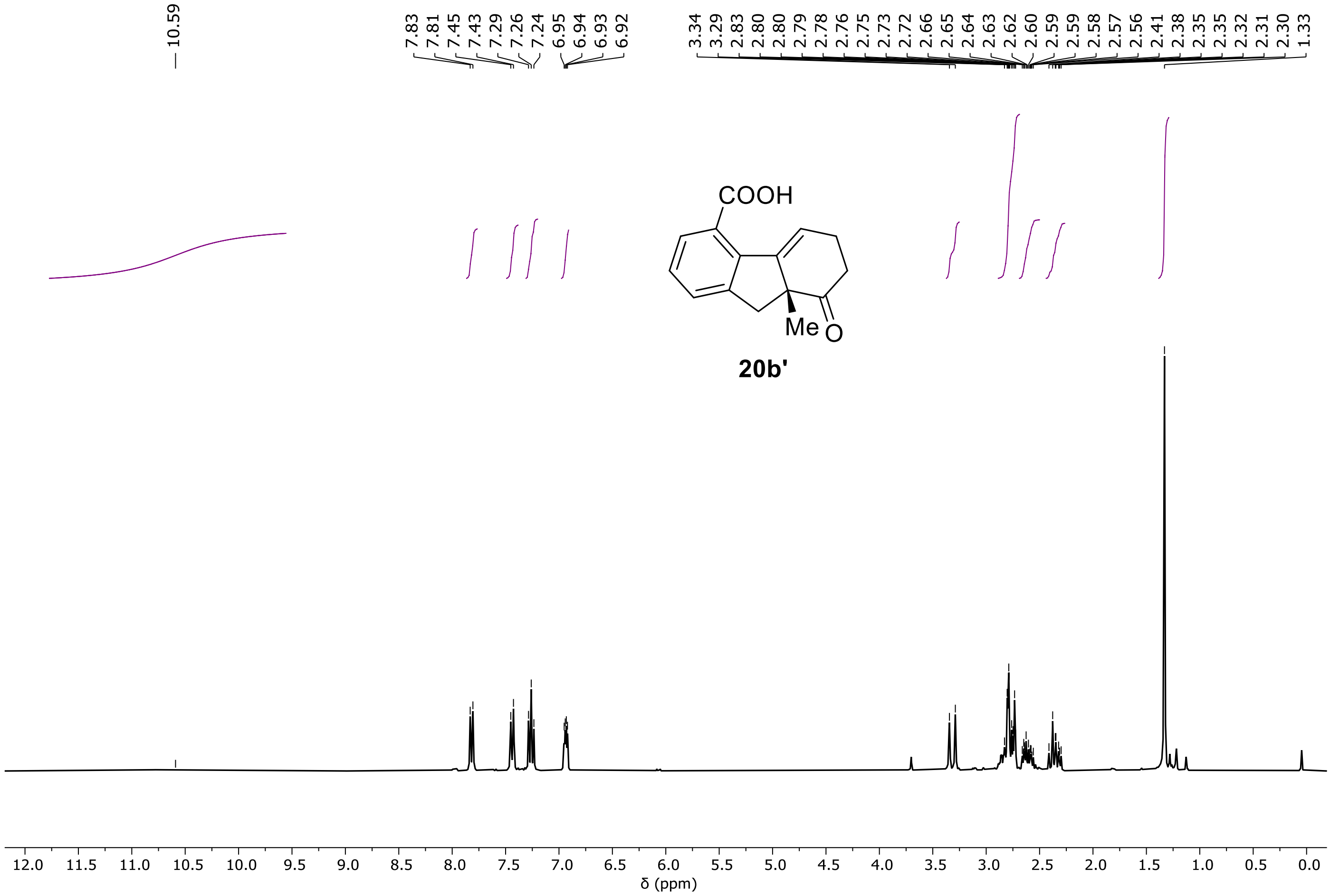

DEPT-135 NMR (75 MHz, CDCl3)

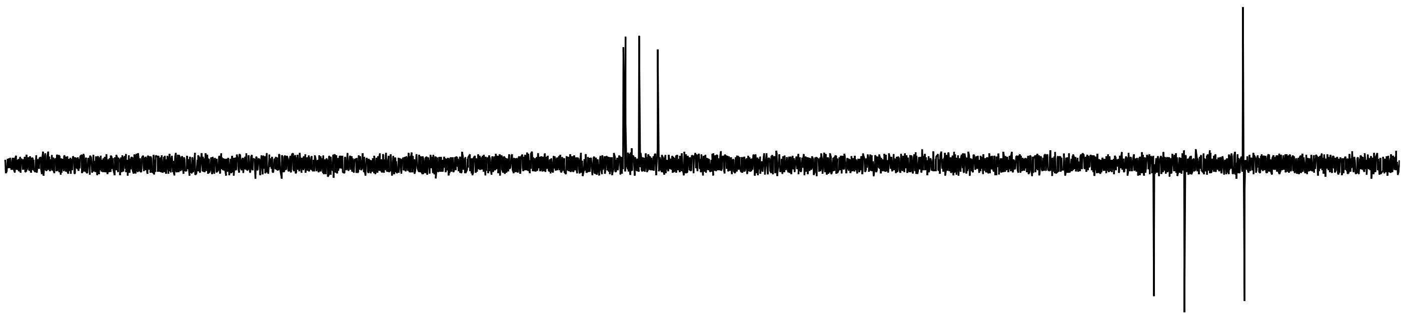

13C NMR (75 MHz, CDCl3)

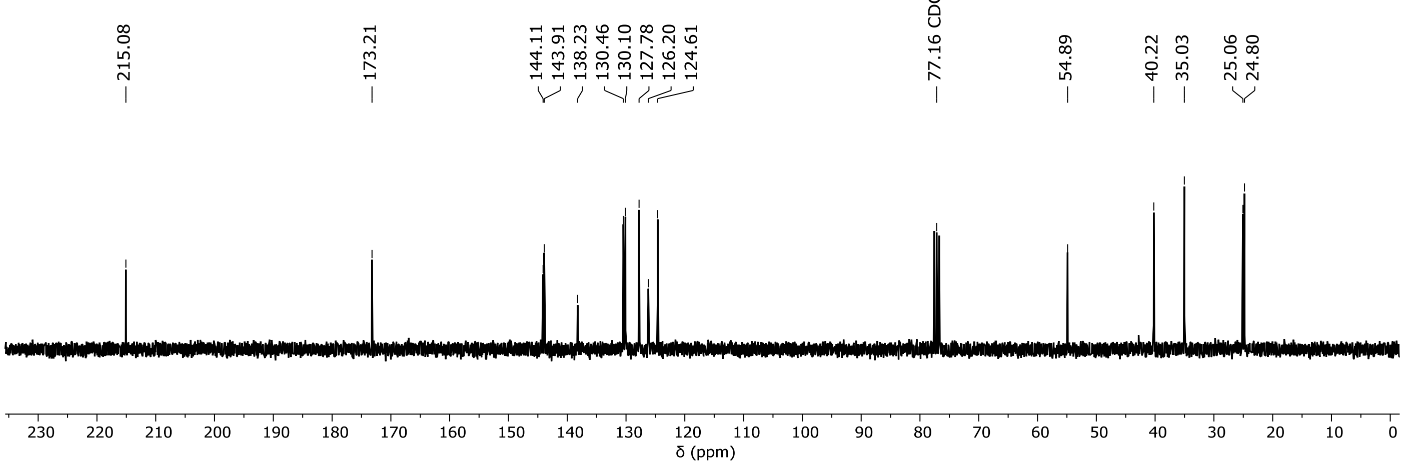

1H NMR (500 MHz, CDCl3)

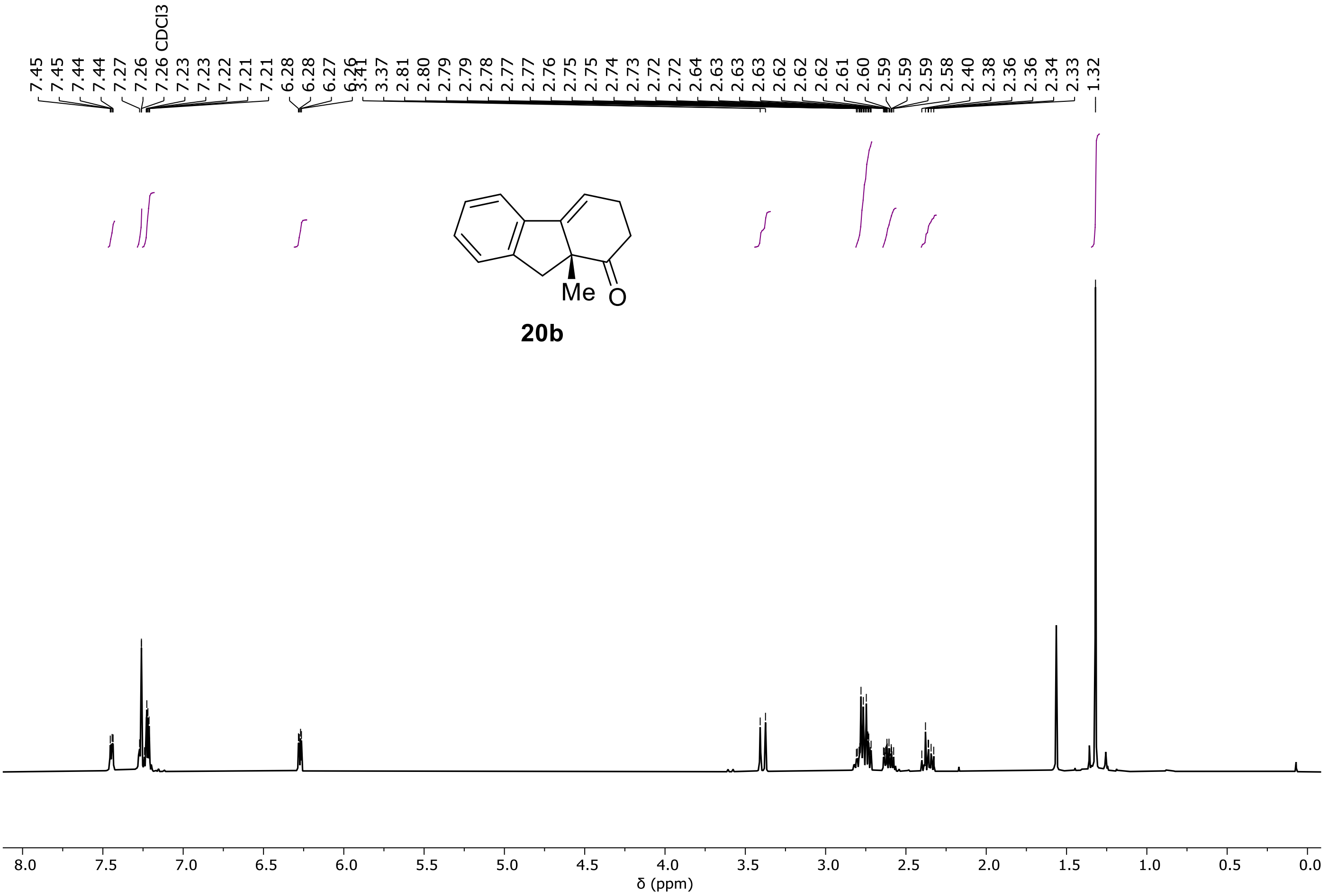

20b

DEPT-135 NMR (126 MHz, CDCl3)

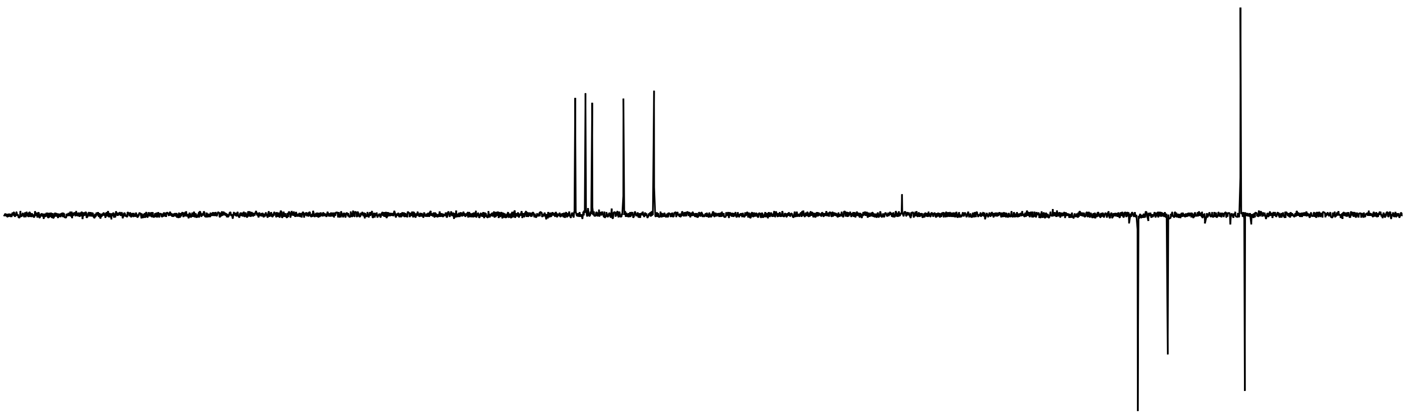

13C NMR (126 MHz, CDCl3)

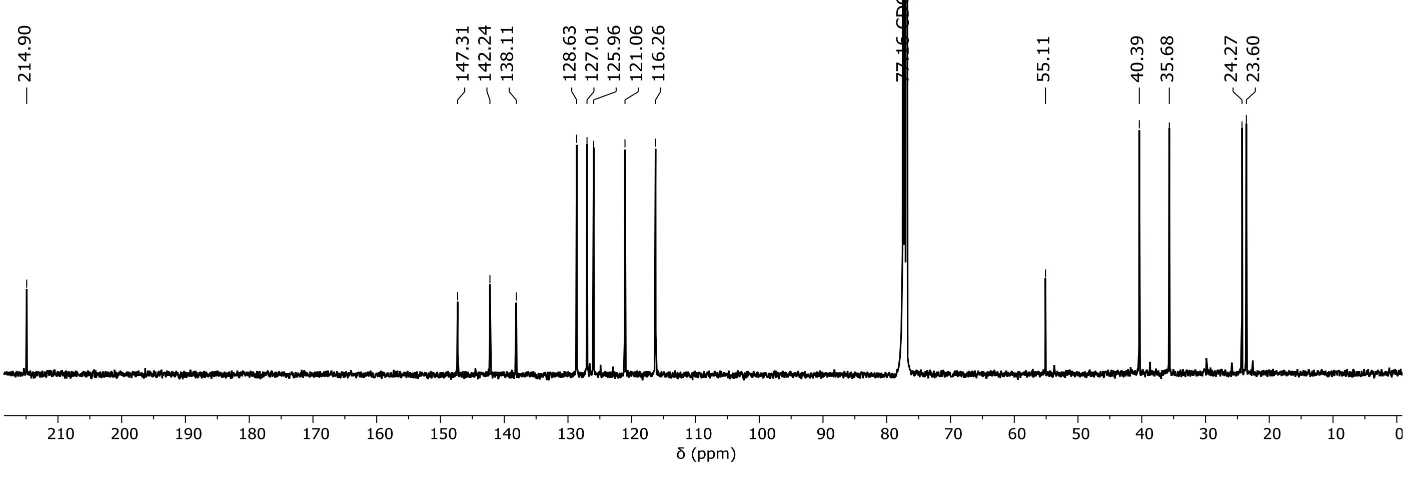

1H NMR (500 MHz, CDCl3)

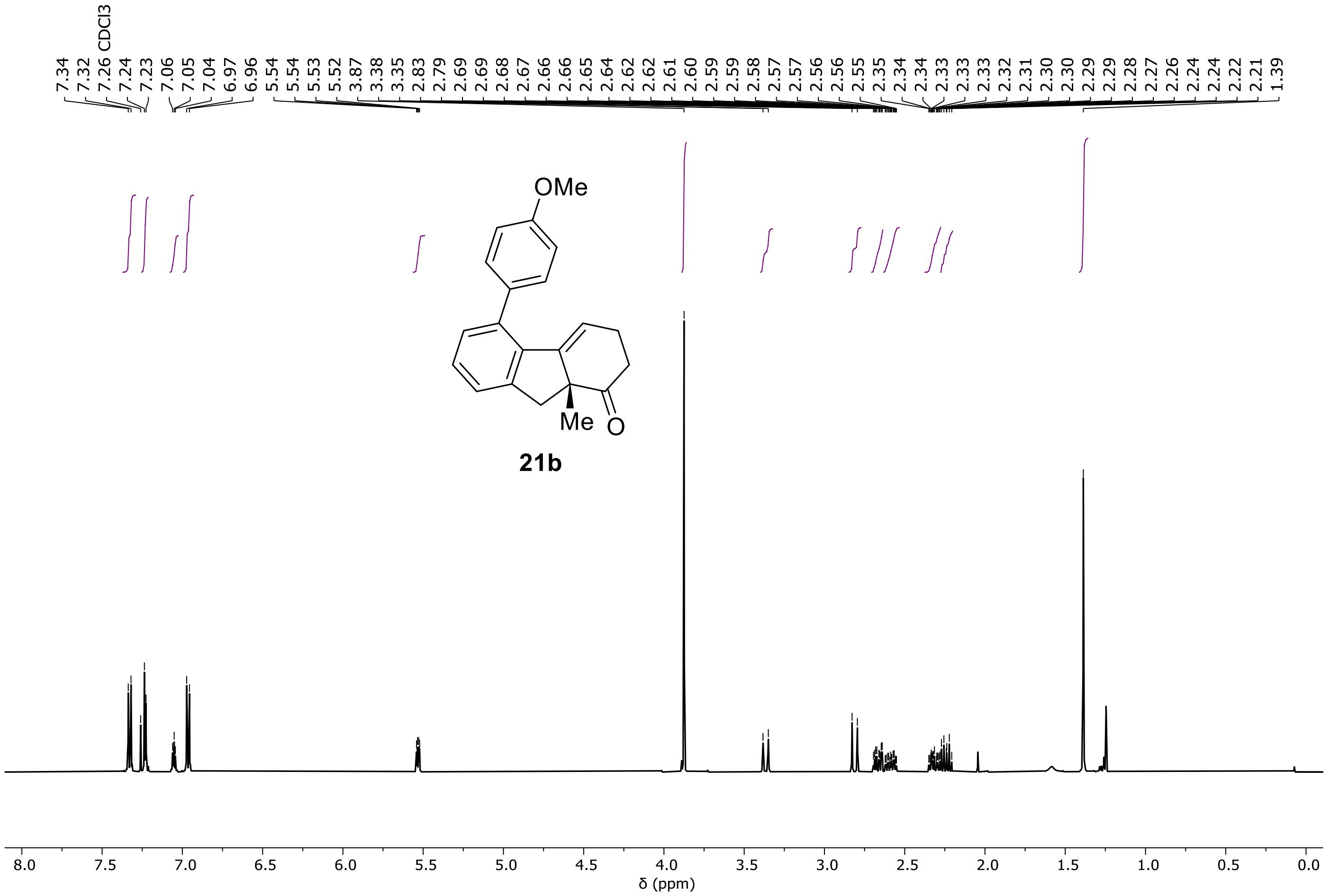

DEPT-135 NMR (126 MHz, CDCl3)

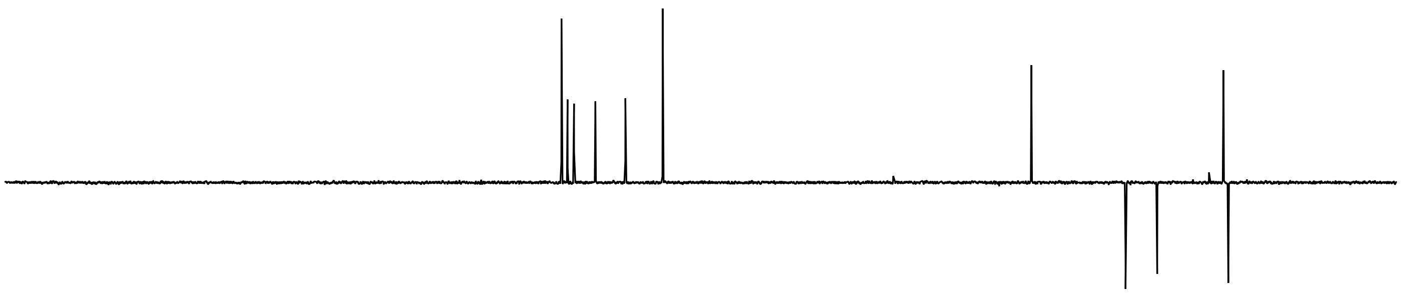

13C NMR (126 MHz, CDCl3)

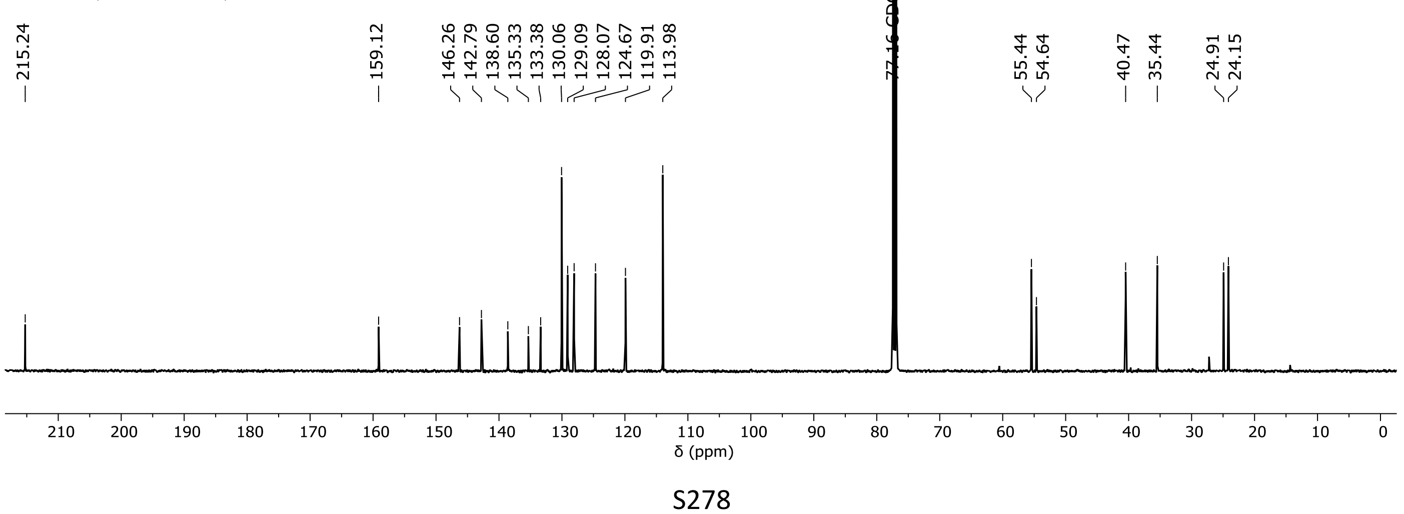

12. References

[1] Dolomanov, O. V.; Bourhis, L. J.; Gildea, R. J.; Howard, J. A. K.; Puschmann, H. OLEX2: A Complete Structure Solution, Refinement and Analysis Program. *J. Appl. Cryst.* **2009**, *42*, 339 – 341.

[2] Precatalyst $[Ir(COD)_2]BAr^F_4$ was prepared following the reported procedure: Woodmansee, D. H.; Müller, M. A.; Neuburger, M.; Pfaltz, A. Chiral Pyridyl Phosphinites with Large Aryl Substituents as Efficient Ligands for the Asymmetric Iridium-Catalyzed Hydrogenation of Difficult Substrates. *Chem. Sci.* **2010**, *1*, 72 – 78.

[3] Procedure adapted from: Zhoua, P.; Xu, T. Nickel-Catalyzed Intramolecular Desymmetrization Addition of Aryl Halides to 1,3-Diketones. *Chem. Commun.* **2020**, *56*, 8194 – 8197.

[4] Procedure adapted from: Zhu, C.; Wang, D.; Zhao, Y.; Sun, W.-Y.; Shi, Z. Enantioselective Palladium-Catalyzed Intramolecular α-Arylative Desymmetrization of 1,3-Diketones. *J. Am. Chem. Soc.* **2017**, *139*, 16486 – 16489.

[5] Procedure adapted from: Morgan, J.; Pinhey, J. T. Reaction of Arylboronic Acids and their Derivatives with Lead Tetra-Acetate. The Generation of Aryl-Lead Triacetates, and Meta- and Para-Phenylenebis(Lead Triacetate), In Situ for Electrophilic Arylation. *J. Chem. Soc., Perkin Trans.* **1990**, *1*, 715 – 720.

[6] Procedure adapted from: Armanino, N.; Koller, R.; Togni, A. Electrophilic Trifluoromethylation of Primary Phosphines: Synthesis of a P-Bis(trifluoromethyl) Derivative of BINAP, *Organometallics* **2010**, *29*, 1771 – 1777.

[7] Procedure adapted from: Han, X.; Shan, L.-X.; Zhu, J.-X.; Zhang, C.-S.; Zhang, X.-M.; Zhang, F.-M.; Wang, H.; Tu, Y.-Q.; Yang, M.; Zhang, W.-S. Copper-Nitrene-Catalyzed Desymmetric Oxaziridination/1,2-Alkyl Rearrangement of 1,3-Diketones toward Bicyclic Lactams. *Angew. Chem. Int. Ed.* **2021**, *60*, 22688 – 22692.

[8] Procedure adapted from: Jenkins, T. J.; Burnell, D. J. Lewis Acid Catalyzed Geminal Acylation Reaction of Ketones with 1,2-Bis((trimethylsilyl)oxy)cyclobutene: Direct Formation of 2,2-Disubstituted 1,3-Cyclopentanedione. *J. Org. Chem.* **1994**, *59*, 1485 – 1491.

[9] Procedure adapted from: Liu, S.; Cheng, L.; Liu, L. Synthesis of Biaryl Carboxylic Acids through a Cascade Suzuki–Miyaura Coupling/Friedel–Crafts Alkylation/Lewis-Acid-Catalyzed Rearrangement/Aromatization Process. *Org. Lett.* **2024**, *26*, 1902 – 1907.

[10] Procedure from: Wu, Y.; Arenas, I.; Broomfield, L. M.; Martin, E.; Shafir, A. Hypervalent Activation as a Key Step for Dehydrogenative ortho C-C Coupling of Iodoarenes. *Chem. Eur. J.* **2015**, *21*, 18779 – 18784.

[11] Ohyoshi, T.; Tano, H.; Kigoshi, H. Synthetic Studies toward Aplysiasecosterol A: Concise Synthesis of the Tricyclic Core and Its Reactions for Introduction of the D Ring Fragment. *Bull. Chem. Soc. Jpn.* **2021**, *94*, 1179 – 1184.

[12] Procedure adapted from: Wu, N.; Song, F.; Yan, L.; Li, J.; You, J. Transition-Metal-Catalyzed C-H Bond Functionalizations: Feasible Access to a Diversity-Oriented β-Carboline Library. *Chem. Eur. J.* **2014**, *20*, 3408 – 3414.

[13] Procedure adapted from: Nelson, R.; Gulías, M.; Mascareñas, J. L.; López, F. Concise, Enantioselective, and Versatile Synthesis of (−)-Englerin A Based on a Platinum-Catalyzed [4C+3C] Cycloaddition of Allenedienes. *Angew. Chem. Int. Ed.* **2016**, *55*, 14359 – 14363.

[14] Procedure adapted from: Danishefsky, S. J.; Masters, J. J.; Young, W. B.; Link, J. T.; Snyder, L. B.; Magee, T. V.; Jung, D. K.; Isaacs, R. C. A.; Bornmann, W. G.; Alaimo, C. A.; Coburn, C. A.; Di Grandi, M. J. Total Synthesis of Baccatin III and Taxol. *J. Am. Chem. Soc.* **1996**, *118*, 2843 – 2859.

[15] Procedure adapted from: Wang C.-Q.; Feng C.; Loh T.-P. Rh(III)-Catalyzed Hydroarylation of Internal Alkynes through C-H Bond Activation. *Asian J. Org. Chem.* **2016**, 5, 1002 – 1007.

[16] Procedure adapted from: Tinnis, F.; Volkov, A.; Slagbrand, T.; Adolfsson, H. Chemoselective Reduction of Tertiary Amides under Thermal Control: Formation of either Aldehydes or Amines. *Angew. Chem. Int. Ed.* **2016**, *55*, 4562 – 4566.

[17] Procedure adapted from: Wu, D.-P.; Ou, W.; Huang, P.-Q. Ir-Catalyzed Chemoselective Reductive Condensation Reactions of Tertiary Amides with Active Methylene Compounds. *Org. Lett.* **2022**, *24*, 5366 – 5371.

[18] Procedure adapted from: Goossen, L. J.; Manjolinho, F.; Khan, B. A.; Rodríguez, N. Microwave-Assisted Cu-Catalyzed Protodecarboxylation of Aromatic Carboxylic Acids. *J. Org. Chem.* **2009**, 74, 2620 – 2623.

[19] Procedure adapted from: Liu, C.; Ji, C.-L.; Qin, Z.-X.; Hong, X.; Szostak, M. Synthesis of Biaryls via Decarbonylative Palladium-Catalyzed Suzuki-Miyaura Cross-Coupling of Carboxylic Acids. iScience 2019, 19, 749 – 759.

[20] Fast, C. D.; Jones, C. A. H.; Schley N. D. Selectivity and Mechanism of Iridium-Catalyzed Cyclohexyl Methyl Ether Cleavage. *ACS Catal.* **2020**, *10*, 6450 – 6456.

[21] Jones, C. A. H.; Schley N. D. Selective Demethylation of O-aryl Glycosides by Iridium-Catalyzed Hydrosilylation. *Chem. Commun.* **2021**, *57*, 5953 – 5956.

[22] Frisch, M. J.; Trucks, G. W.; Schlegel, H. B.; Scuseria, G. E.; Robb, M. A.; Cheeseman, J. R.; Scalmani, G.; Barone, V.; Petersson, G. A.; Nakatsuji, H.; Li, X.; Caricato, M.; Marenich, A. V.; Bloino, J.; Janesko, B. G.; Gomperts, R.; Mennucci, B.; Hratchian, H. P.; Ortiz, J. V.; Izmaylov, A. F.; Sonnenberg, J. L.; Williams-Young, D.; Ding, F.; Lipparini, F.; Egidi, F.; Goings, J.; Peng, B.; Petrone, A.; Henderson, T.; Ranasinghe, D.; Zakrzewski, V. G.; Gao, J.; Rega, N.; Zheng, G.; Liang, W.; Hada, M.; Ehara, M.; Toyota, K.; Fukuda, R.; Hasegawa, J.; Ishida, M.; Nakajima, T.; Honda, Y.; Kitao, O.; Nakai, H.; Vreven, T.; Throssell, K.; Montgomery, J. A., Jr.; Peralta, J. E.; Ogliaro, F.; Bearpark, M. J.; Heyd, J. J.; Brothers, E. N.; Kudin, K. N.; Staroverov, V. N.; Keith, T. A.; Kobayashi, R.; Normand, J.; Raghavachari, K.; Rendell, A. P.; Burant, J. C.; Iyengar, S. S.; Tomasi, J.; Cossi, M.; Millam, J. M.; Klene, M.; Adamo, C.; Cammi, R.; Ochterski, J. W.; Martin, R. L.; Morokuma, K.; Farkas, O.; Foresman, J. B.; Fox, D. J. Gaussian 16, Revision C.01; Gaussian, Inc.: Wallingford, CT, **2016**.

[23] Kohn, W.; Becke, A. D.; Parr, R. G. Density Functional Theory of Electronic Structure. *J. Phys. Chem.* **1996**, *100*, 12974 – 12980.

[24] Hay, P. J.; Wadt, W. R. *Ab initio* Effective Core Potentials for Molecular Calculations. Potentials for K to Au Including the Outermost-Core Orbitals. *J. Chem. Phys*. **1985**, *82*, 299 – 310.

[25] Zhao, Y.; Truhlar, D. G. Theor. The M06 Suite of Density Functionals for Main Group Thermochemistry, Thermochemical Kinetics, Noncovalent Interactions, Excited States, and Transition Elements: Two New Functionals

and Systematic Testing of Four M06-Class Functionals and 12 other Functionals. *Theor. Chem. Acc.* ***2008***, *120*, 215 – 241.

[26] Dolg, M.; Wedig, U.; Stoll, H.; Preuss, H. Energy-Adjusted *ab initio* Pseudopotentials for the First-Row Transition Elements. *J. Chem. Phys.* **1987**, *86*, 866 – 872.

[27] Marenich, A. V.; Cramer, C. J.; Truhlar, D. G. Universal Solvation Model Based on Solute Electron Density and a Continuum Model of the Solvent Defined by the Bulk Dielectric Constant and Atomic Surface Tensions. *J. Phys. Chem. B.* **2009**, *113*, 6378 – 6396.

[28] Lu, T. and Chen, F., Multiwfn: A multifunctional wavefunction analyzer. *J. Comput. Chem.*, **2012**, *33*, 580 – 592.

[29] Pracht, P.; Grimme, S.; Bannwarth, C.; Bohle, F.; Ehlert, S.; Feldmann, G.; Gorges, J.; Müller, M.; Neudecker, T.; Plett, C.; Spicher, S.; Steinbach, P.; Wesołowski, P. A.; Zeller, F., CREST—A program for the exploration of low-energy molecular chemical space. *J. Chem. Phys.* **2024,** *160*, 114110.
